# Understanding varenicline function via key receptor and ligand interactions

**DOI:** 10.1016/j.xcrp.2025.102992

**Published:** 2025-12-17

**Authors:** Sheenagh G. Aiken, Daniele Fiorito, Matthew Harper, Grzegorz Pikus, Juno Underhill, Jacob Murray, Joshua Rawlinson, AnnMarie C. O’Donoghue, Cecilia Gotti, Sarah C.R. Lummis, Teresa Minguez Viñas, Franco Viscarra, Isabel Bermudez, Timothy Gallagher, A. Sofia F. Oliveira

**Affiliations:** 1 School of Chemistry, University of Bristol, Bristol BS8 1TS, UK; 2 Department of Chemistry, Durham University, South Road, Durham DH1 3LE, UK; 3 CNR, Institute of Neuroscience, University of Milan, 20129 Milan, Italy; 4 Department of Biochemistry, University of Cambridge, Cambridge CB2 1QW, UK; 5 Department of Biological and Medical Sciences, Oxford Brookes University, Oxford OX3 0BP, UK; 6 Centre for Computational Chemistry, School of Chemistry, University of Bristol, Bristol BS8 1TS, UK

**Keywords:** nicotinic acetylcholine receptor, receptor-agonist interactions, ligand selectivity, ligand function, binding profile, functional profile, functional mutations, molecular dynamics simulations, serotonin 5-HT receptor

## Abstract

Approved by the US Food and Drug Administration in 2006, varenicline was the first nicotinic-based therapy for smoking cessation, targeting the α4β2 nicotinic acetylcholine receptor (nAChR). While inspired by cytisine, varenicline has distinct effects at both target and off-target receptors; however, despite being widely used clinically, the precise molecular interactions underpinning varenicline’s mode of action remain unclear. Using a multidisciplinary approach, the interactions that set varenicline apart from related compounds such as nicotine and cytisine have been identified. In particular, the binding-site residues α4T139, α4T183, and especially β2S133 were shown to be key modulators for varenicline’s function. Substituting β2S133 with valine significantly reduced efficacy, pinpointing it as a crucial determinant. Additionally, a set of novel varenicline variants showed that the positioning of the quinoxaline moiety in varenicline is essential for receptor activation. These insights reveal a unique interaction network at α4β2 that underlies varenicline’s function, offering a deeper understanding of the ligand’s working mechanism.

## Highlights

• Varenicline interacts with serine and threonine donors to drive α4β2 nAChR partial agonism
• β2S133 and α4T183 are key in the α-β binding site, with β2S133 the critical determinant
• β2S133V reduces varenicline efficacy, underscoring β2S133 functional importance
• New varenicline variants pinpoint the quinoxaline moiety’s role for receptor activation

## Graphical abstract

**Figure:**
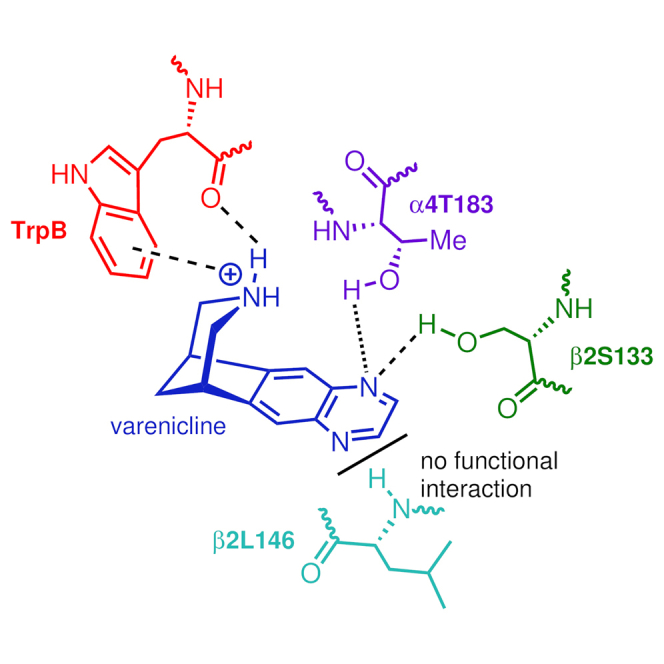

## Teaser

Aiken et al. reveal that serine and threonine hydrogen-bond donors, alongside cation-π and hydrogen-bond acceptor interactions, shape varenicline's partial agonism at α4β2 nicotinic receptors. Structural variants show the heteroaryl hydrogen-bond acceptor’s position is crucial for activating α4β2 and 5-HT_3_ receptors across multiple related binding contexts and functional receptor assays *in vitro*.

## Introduction

Tobacco consumption, with the World Health Organization estimating >8 million deaths annually, is a leading cause of preventable disease and death worldwide. Of this total, 7 million deaths are attributable to direct smoking, and approximately 1.3 million are due to second-hand smoke exposure.^1^^,^^2^ As a result, smoking cessation still represents a major but frustratingly challenging and increasingly ephemeral global health objective.^3^^,^^4^^,^^5^^,^^6^^,^^7^ Further, recent declines in the prevalence of tobacco consumption have slowed, legislation to limit tobacco sales has failed to keep pace (or been reversed), and this situation has been exacerbated by a sharp increase in nicotine consumption via electronic vapes^8^^,^^9^^,^^10^^,^^11^ and pouches.^12^^,^^13^ Consequently, this major public health threat should be viewed as one of nicotine, as opposed to solely tobacco, addiction.^14^^,^^15^^,^^16^^,^^17^

**Figure 1:**
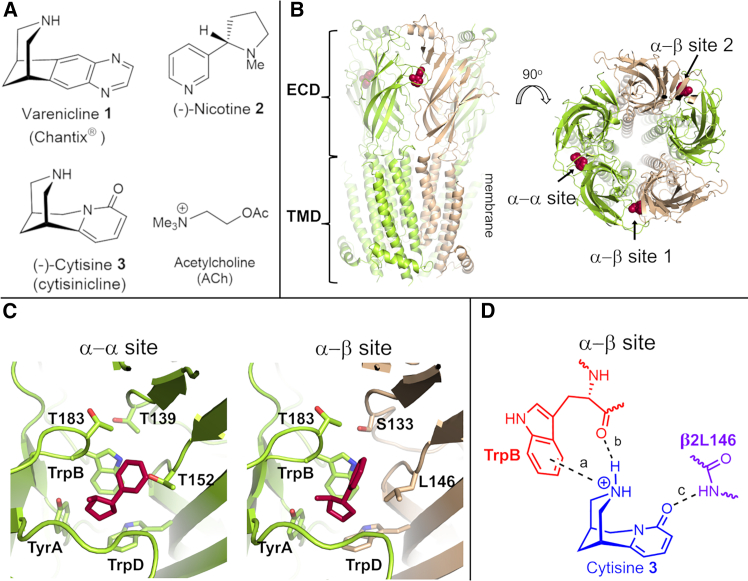
The α4β2 nAChR: structure, agonists, and key agonist-receptor interactions (A) Chemical structures of varenicline **1**, nicotine **2**, cytisine **3**, and ACh. (B) Cryo-EM structure of the human LS isoform of the α4β2 nAChR (i.e., (α4)_3_(β2)_2_) with nicotine bound (PDB: 6CNK).^22^ nAChRs are composed of three domains: an extracellular domain (ECD), a transmembrane domain (TMD), and an intracellular domain (ICD); the ICD is absent in the cryo-EM 6CNK structure.^22^ The LS isoform of the α4β2 nAChR contains one α-α pocket (at the interface between the two α4 subunits) and two α-β sites (formed by an α4 and a β2 subunit). (C) Close-up view of the α-α and α-β binding pockets in 6CNK.^22^ The side chains of the several conserved residues, namely TyrA (Y126 in the principal α4 side), TrpB (W182 in the principal α4 side), and TrpD (W88 in the complementary α4 side of the α-α pocket and W82 in the complementary β2 side of the α-β pocket) as well as the residues that are the focus of the current work, notably α4T183 (principal α4 side), α4T139 (complementary α4 side of the α-α pocket), β2S133 (complementary β2 side of the α-β pocket), α4T152 (complementary α4 side of the α-α pocket), and β2L146 (complementary β2 side of the α-β pocket), are shown with sticks. The residue numbers refer to UniProt sequences P43681 and P17787 for the human α4 and β2 subunits, respectively. Here and in (B), the α4 and β2 subunits are colored yellow and light brown, respectively. Nicotine **2** is highlighted in red. (D) Dougherty-Lester nAChR functionally important binding model for the α-β pocket illustrated for cytisine **3** showing the three key functional interactions identified: (a) cation-π and (b) backbone C=O as H-bond acceptor associated with TrpB within the α4 subunit; and (c) backbone NH as H-bond donor associated with β2L146 (in the complementary β2 subunit).^23^^,^^24^^,^^25^^,^^26^

A key part of the smoking cessation toolkit is varenicline **1** (Figure 1A), which was launched in 2006 as Chantix (Champix in Europe)^18^ to support smoking cessation.^17^^,^^18^^,^^19^^,^^20^ Available (since 2022) in generic form, varenicline **1** is estimated to have been used by >24 million smokers and represents the first nicotinic acetylcholine receptor (nAChR) therapeutic^21^ approved by the US Food and Drug Administration.

As a partial agonist, varenicline **1** targets the α4β2 subtype of the nAChRs found in the central nervous system.^19^^,^^27^^,^^28^^,^^29^ Due to its high affinity for nicotine **2**, this subtype emerged as the primary focus for nicotine addiction^14^^,^^30^^,^^31^ and, consequently, the primary target receptor for smoking cessation.^14^^,^^30^^,^^31^ Besides the α4β2 subtype, varenicline **1** also activates (as a full agonist) the α7 subtype,^29^^,^^32^^,^^33^ although the α7 nAChR role in smoking cessation remains undetermined. Significantly, varenicline **1** also activates the 5-HT_3_ serotonin receptor,^34^ a structurally related member of the Cys loop superfamily.^15^^,^^35^

The genesis of varenicline **1** as a novel (and consequently patentable) smoking cessation agent lies in the known profile of cytisine (**3** [the generic drug name cytisinicline was designated by the USAN Council in 2018]) (Figure 1A).^36^^,^^37^ Cytisine **3**, isolated from laburnum^38^ and used in eastern Europe as a smoking cessation agent since the 1960s,^39^^,^^40^^,^^41^^,^^42^^,^^43^ is both a high-affinity partial agonist for the α4β2 nAChR and a full agonist at the α7 subtype.^29^^,^^32^^,^^44^^,^^45^ However, varenicline **1** and cytisine **3** diverge in terms of their functional profile at the α4β2 and 5-HT_3_ receptors. First, varenicline **1** and cytisine **3** have substantially different profiles in α4β2 nAChR,^29^^,^^32^^,^^44^^,^^45^ which presents in two receptor stoichiometries: (α4)_2_(β2)_3_, the high-sensitivity (to activation by ACh; HS) complex, which contains two α-β binding sites; and (α4)_3_(β2)_2_, the low-sensitivity (toward ACh; LS) complex, which exhibits two α-β sites plus an α-α pocket (Table 1; Figures 1B and 1C).^29^^,^^32^ Varenicline **1** is efficacious in both α4β2 isoforms, while cytisine **3** only activates the LS stoichiometry; the efficacy at both isoforms of varenicline **1**, nicotine **2**, and cytisine **3** (relative to ACh, which is a full agonist) in the α4β2 subtype are shown in Table 1.^21^ Varenicline **1**, nicotine **2**, and cytisine **3** are all partial agonists (with an efficacy relative to ACh of 0.18, 0.31, and 0.025 for the HS isoform and 0.41, 0.53, and 0.23 for the LS isoform), whereas ACh is a full agonist of both LS and HS isoforms of the α4β2 receptor (Table 1).

**Table 1:** Potency and relative efficacy of nicotinic ligands at wild-type and targeted mutants of both HS and LS isoforms of the α4β2 nAChR

Mutation	ACh		Varenicline **1**		Nicotine **2**		Cytisine **3**	
	EC_50_ (μM)	RE	EC_50_ (μM)	RE	EC_50_ (μM)	RE	EC_50_ (μM)	RE
**(α4)**_**2**_**(β2)**_**3**_**(HS isoform)**								

WT	3.2 ± 0.7	1	0.090 ± 0.01	0.18 ± 0.02	1.77 ± 0.4	0.31 ± 0.01	ND	0.025 ± 0.004
β2S133V	4 ± 1.3	1	3.10 ± 0.4^a^	0.059 ± 0.017^a^	3.0 ± 0.5	0.23 ± 0.02^a^	ND	0.016 ± 0.002
α4T183V	3.4 ± 0.4	1	0.57 ± 0.01^a^	0.099 ± 0.004^a^	2.9 ± 0.5	0.29 ± 0.01	ND	0.021 ± 0.003
α4T139V	2.8 ± 0.4	1	0.051 ± 0.001	0.18 ± 0.02	1.5 ± 0.4	0.29 ± 0.02	ND	0.023 ± 0.001
β2S133Vα4T183V	3.3 ± 1.0	1	2.47 ± 0.6^a^	0.07 5 ± 0.0031^a^	3.2 ± 0.5	0.20 ± 0.01^a^	ND	0.018 ± 0.003
β2S133Vα4T139V	3.5 ± 0.9	1	3.58 ± 0.4^a^	0.067 ± 0.018^a^	2.8 ± 0.9	0.22 ± 0.02^a^	ND	0.022 ± 0.009

**(α4)**_**3**_**(β2)**_**2**_**(LS isoform)**								

WT	99 ± 10	1	1.15 ± 6	0.41 ± 0.02	7.5 ± 1.6	0.53 ± 0.05	2.9 ± 0.8	0.23 ± 0.08
β2S133V	105 ± 15	1	33 ± 1.4^a^	0.02 ± 0.003^a^	11.2 ± 2	0.48 ± 0.06^a^	5.1 ± 1	0.21 ± 0.01
α4T183V	95 ± 10	1	8.0 ± 0.3^a^	0.20 ± 0.02^a^	6.9 ± 2	0.52 ± 0.03	4.6 ± 1	0.22 ± 0.02
α4T139V	88 ± 9	1	8.4 ± 5.4^a^	0.20 ± 0.02	7.4 ± 1	0.54 ± 0.05	2.5 ± 0.7	0.19 ± 0.02
β2S133Vα4T183V	101 ± 4	1	33.0 ± 5^a^	0.033 ± 0.005^a^	10.2 ± 2	0.47 ± 0.01^a^	6.9 ± 1.5	0.19 ± 0.01
β2S133Vα4T139V	98 ± 10	1	41.34 ± 2^a^	0.031 ± 0.005^a^	11.4 ± 2	0.46 ± 0.02^a^	5.8 ± 1.4	0.18 ± 0.01

Relative efficacy (RE) was determined by normalizing the maximal current responses elicited by varenicline **1** to the maximal current response to ACh (*I*/*I*_max_ACh). EC_50_ values were estimated as described in supplemental methods. Data shown represent the mean ± SEM of *n =* 8–10 experiments in 6–8 different batches of *Xenopus* oocytes. Statistical differences between wild-type (WT) and mutant receptors were determined by one-way ANOVA followed by a post hoc Dunnett’s test and/or a post hoc Bonferroni multiple comparison test to determine the level of significance between WT and mutants. ND, not determined due to low levels of functional expression (less than 50 nA of ACh maximal currents).

a Statistically significant difference (*p* < 0.05) between mutant and WT receptors.

Second, cytisine **3** is a weak antagonist at the 5-HT_3_ receptor, unlike varenicline **1**, which is an agonist at this receptor,^34^^,^^46^ with this difference linked to side effects experienced with varenicline **1** therapy.^47^ These differing profiles of otherwise two closely related compounds raise fundamental questions about their underlying mechanisms of action at the α4β2 nAChR. First, what are the details of the protein-ligand interactions that both mediate and differentiate the functional effects of varenicline **1** vs. cytisine **3** at the α4β2 nAChR? Second, how does varenicline **1** binding translate into efficacy, i.e., receptor activation? Clearly, this begs the question that if varenicline **1** differs from cytisine **3** in terms of how it binds to the receptor, does that difference regulate function? Further, if the specifics of the α4β2 interactions are different, does this shed light on why varenicline **1** is an agonist while cytisine **3** is an antagonist at the human 5-HT_3_ receptor? All these questions direct us toward the need to enhance our understanding of the network of receptor-ligand interactions that govern the wider functional profile of varenicline **1**.

The seminal work of Dougherty and Lester,^23^^,^^24^^,^^25^^,^^26^^,^^48^^,^^49^ together with structural studies of soluble acetylcholine-binding proteins (AChBP)^50^^,^^51^^,^^52^^,^^53^ and recent high-resolution structural data for complete nAChRs,^54^^,^^55^^,^^56^^,^^57^^,^^58^ including the α4β2-nicotine, acetylcholine, and varenicline complexes,^22^^,^^59^^,^^60^^,^^61^ has defined a binding model for nicotinic agonists (Figure 1D).

The salient features of the Dougherty-Lester model consist of a cation-π and H-bond donor (except for acetylcholine) associated with the piperidinium center and a highly conserved tryptophan residue in loop B (TrpB) in the principal face of the agonist binding site,^23^^,^^24^ and an H-bond acceptor component within the ligand (C=O, in the case of cytisine **3**) that interacts with a donor within the complementary face (Figure 1D).^25^ In addition to TrpB, the binding pockets are formed by several conserved aromatic residues, including a tyrosine in loop A (TyrA), two tyrosines in loop C (TyrC1 and TyrC2), and a tryptophan in loop D (TrpD). These residues play a critical role in ligand recognition and binding.^62^ For most agonists bound to the α4β2 nAChR, the H bond with the complementary face involves an interaction with the backbone NH of β2L146 (which corresponds to β2L119 in the work of Blum et al.^25^) in the complementary face.^25^^,^^26^^,^^49^ However, while this interaction mediates ACh, nicotine **2**, cytisine **3**, sazetidine-A, carbamylcholine, and epibatidine function,^25^^,^^26^^,^^49^ varenicline **1** was shown not to engage with β2L146, at least in terms of linking to receptor function, in the α4β2 subtype.^49^ Dougherty and co-workers used changes in EC_50_ to determine the relevance of a ligand-receptor interaction,^48^ and backbone mutation of β2L146 did not affect varenicline **1** function in either of the LS and HS α4β2 isoforms.^49^ Note, however, that these findings do not exclude the involvement of β2L146 in varenicline **1** recognition/binding. Moreover, other interactions within the agonist binding sites can contribute to subtype differentiation. For instance, we have shown how α7R101 and β2R106 can modulate functional outcomes across nAChR subtypes^32^^,^^63^ and how agonist-induced structural and dynamics changes are transmitted from the agonist binding site (in α4β2 and α7) to the ion channel,^64^^,^^65^ allowing gating to occur.

Given that Dougherty and Lester demonstrated that varenicline **1** retains the cation-π and H-bond donor components in the α4β2 nAChR (Figure 1D)^26^ but lacks the β2L146 H-bond donor interaction, we have focused here on uncovering the specific residue(s) that partner with varenicline (as an H-bond acceptor) in shaping the functional profile of this ligand.

To investigate the variation of receptor-ligand network that mediates function across different nicotinic agonists, including varenicline **1**, we have integrated computational and experimental approaches to identify networks of H-bond donor interactions within the primary and complementary faces of α4β2 nAChR. In addition, we have also designed a unique set of varenicline **1** variants that probe the ligand features that mediate binding to and function of the human α4β2 nAChR. These studies offer a nuanced explanation of how and why the profile of varenicline **1** deviates from that of other nicotinic ligands, including cytisine **3**, in the α4β2 nAChR. We demonstrate that varenicline **1** engages in functionally relevant interactions with β2S133 on the complementary β2 side and (to a lesser extent) with α4T183 on the principal α4 side of the agonist pocket. Notably, the interaction with β2S133 plays a substantial role in shaping the functional profile of varenicline **1**, emerging as a distinguishing feature of its association with the receptor. Varying varenicline **1** structure suggests that the size and shape of the ligand (which were both retained in all designed variants) are significant for binding at α4β2 nAChR, but that function is associated with the precise location of the H-bond acceptor (quinoxaline) moiety within varenicline **1**. A similar picture was observed regarding the location of the quinoxaline group at the 5-HT_3_ receptor, where it was also shown to be a critical determinant of varenicline’s profile.

## Results

### Identifying alternative H-bond networks in the agonist binding sites of α4β2 nAChR

Molecular dynamics (MD) simulations were performed for several α4β2 nAChR-agonist complexes to identify alternative H-bonding donor networks within the α4β2 agonist binding sites (see supplemental methods and Table S1). The complexes between the extracellular domain (ECD) of the human α4β2 LS and HS isoforms and varenicline **1**, nicotine **2**, cytisine **3**, and ACh were constructed (wild-type [WT] complexes) and optimized (Figures S2 and S3) as described in supplemental methods.

**Figure 2:**
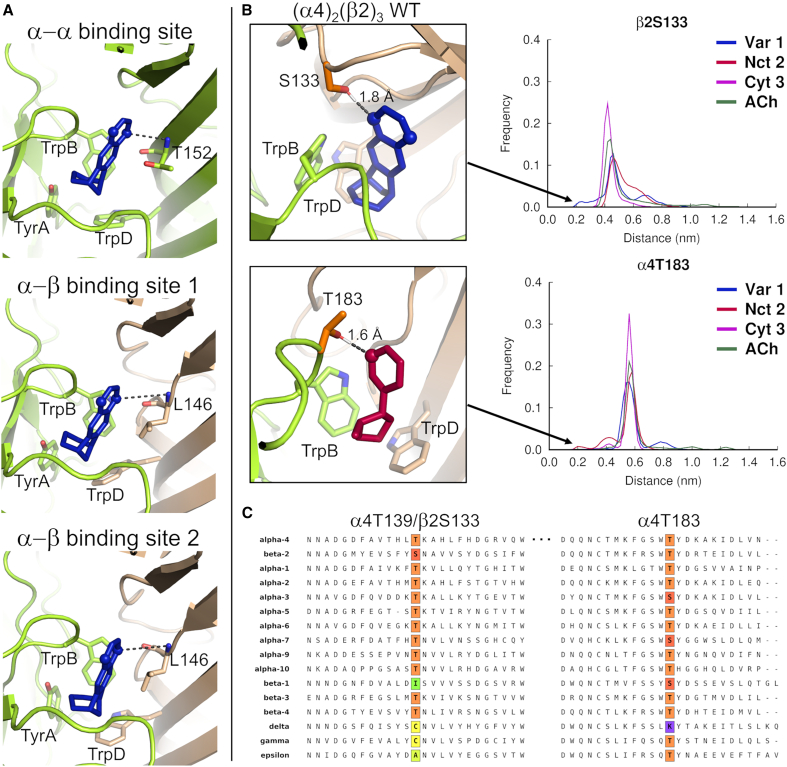
Varenicline interactions in the α-α and α-β binding sites in the wild-type complexes (A) Optimized binding mode for varenicline **1** in the α-α and α-β binding sites in the LS wild-type complex. The α4 and β2 subunits are colored yellow and brown, respectively. TyrA, TrpB, TrpD, α4T152, and β2L146 are shown as sticks. Varenicline **1** is colored blue, with the nitrogen atoms of the quinoxaline moiety highlighted by spheres. Note that the distance between the nearest H-bond acceptor in the quinoxaline ring and the backbone NH group of α4T152 and β2L146 (indicated by dashed lines) exceeds 3.8 Å (distances >3.8 Å are also observed for the α-β binding sites in the HS complex), suggesting that no H bond is formed between these two groups. (B) Distribution of the minimum distance between the agonist (specifically, the closer pyrazine nitrogen in the quinoxaline moiety of varenicline **1**, the pyridine nitrogen of nicotine **2**, the pyridone oxygen of cytisine **3**, and the carbonyl oxygen of ACh) and α4T183 and β2S133 in the α-β binding pockets of the HS wild-type complex (right panels). The left panels illustrate examples of conformations where a hydrogen bond between varenicline **1** and β2S133 and nicotine **2** and α4T183 are present (as indicated by the dashed lines). The α4 and β2 subunits are colored yellow and brown, respectively, whereas varenicline **1** and nicotine **2** are highlighted in blue and red. The side chains of TrpB, TrpD, α4T183, and β2S133 are represented by sticks. The nitrogen atoms of the quinoxaline ring of varenicline **1** and the pyridine nitrogen of nicotine **2** are highlighted by spheres. (C) Sequence alignment for the α4T183, α4T152/β2L146, and α4T139/β2S133 regions of various human nAChR subunits. The colored boxes highlight the locations of α4T183, α4T139, and β2S133 (the residues mutated in this work), with threonine, serine, isoleucine, cysteine, alanine, and lysine residues represented by orange, red, green, yellow, light green, and purple, respectively. The white box marks the location of α4T152 and β2L146, which were not mutated here. The sequences shown correspond to UNIPROT: P43681 (human α4), UNIPROT: P17787 (human β2), UNIPROT: P02708 (human α1), UNIPROT: Q15822 (human α2), UNIPROT: P32297 (human α3), UNIPROT: P30532 (human α5), UNIPROT: Q15825 (human α6), UNIPROT: P36544 (human α7), UNIPROT: Q9U6MI (human α9), UNIPROT: Q9GZZ6 (human α10), UNIPROT: P11230 (human β1), UNIPROT: Q05901 (human β3), UNIPROT: Q07001 (human δ), UNIPROT: P07510 (human γ), and UNIPROT: Q04844 (human ε). The sequence alignments were performed using the Muscle server.^66^

In the WT varenicline **1** models, the H-bond acceptor quinoxaline moiety does not directly engage with the backbone amide of either α4T152 (in the α-α pocket) or β2L146 (in the α-β pockets) (Figure 2A), which is consistent with Dougherty and co-workers’ observation that β2L146 does not modulate the function of varenicline **1**.^49^

MD simulations were performed for the WT HS and LS complexes to probe relevant differences in H-bond patterns within the receptor. These simulations provided a more complete picture of the dynamic behavior of the complexes and the nature of the ligand-receptor interactions (i.e., cation-π and H-bond interactions) within the α-β and α-α binding sites (Figures S6–S19). Besides the well-established interactions with TrpB, TyrA, and TrpD (Figures S17–S19), analysis of the distances between the H-bond acceptor in varenicline **1**, nicotine **2**, cytisine **3**, and ACh and H-bond donors within the binding pockets further identified three alternative residues that can transiently interact with some of the agonists (Figure S20). These involved α4T183 in the principal face of the pocket and β2S133 and α4T139 in the complementary face of the α-β and α-α pockets, respectively. As can be seen in Figures 2B and S20, the H-bond acceptor groups of the agonists can closely approach the side-chain H-bond donors of α4T183, β2S133, and α4T139, suggesting that a transient (either direct or mediated by a water molecule) interaction between these groups is feasible.

Within the α-β binding sites, the residues identified as potential H-bond partners for the agonists were the hydroxyl groups of α4T183 and β2S133. While α4T183 is positioned on the side of the pocket immediately after the key TrpB residue, β2S133 lines the back of the orthosteric site (Figure 1C). In our simulations, only one of the quinoxaline nitrogen atoms of (meso) varenicline **1** was able to closely contact β2S133, while the pyridine nitrogen of nicotine **2** was able to interact with the α4T183 (Figures 2B and S20). In the LS isoform, which contains an additional agonist site on the α-α subunit interface,^22^^,^^67^ the new interaction is associated with α4T139, which is located in the complementary face in a position analogous to that of β2S133 on the α-β interface (Figure S20). Note that the interactions involving α4T139/β2S133 and α4T183 do not involve backbone NH donors (as in the case of β2L146 in Figure 1D) but rather the hydroxyl-containing side chain and are potentially synergistic in terms of the network offered.

Sequence alignment of nAChR subunits indicates that the presence of an H-bond donor group at positions equivalent to α4T139/β2S133 and α4T183 is highly conserved across human receptors (Figure 2C; for further details, see Figure S21). All human neuronal subunits possess a residue with a hydroxyl-containing side chain at the α4T139/β2S133 position, with the α2-α7, α9-α10, and β3-β4 subunits featuring a threonine and the β2 subunit a serine. In contrast to the human neuronal subunits, the muscle subunits (i.e., α1, β1, γ, δ, and ε) exhibit greater diversity in the residue at position α4T139/β2S133, with less scope to participate in H bonding.^68^^,^^69^ At the α4T183 position, all human neuronal and muscle subunits have either a threonine or a serine, except for a lysine in the δ subunit. The highly conserved nature of residues at the α4T139/β2S133 and α4T183 positions in neuronal subunits suggests that the side-chain hydroxyl group, acting as an H-bond donor, may play a key role in defining the action of certain ligands.

In the WT simulations, varenicline **1** exhibits strong positively concerted motions with α4T139, α4T183, and β2S133 in both α-α and α-β sites, whereas the correlation profiles for nicotine **2**, cytisine **3**, and ACh vary between pockets, generally showing weaker correlations with the α-α pocket (Figure S22). Previous work comparing the α-α and α-β sites has shown that their differences stem primarily from the chemical characteristics of three residues located on the complementary face of the sites, namely the hydrophobic β2V136, β2F144, and β2L146 in the α-β pocket and the polar α4H142, α4Q150, and α4T152 in the α-α site.^49^^,^^67^^,^^70^^,^^71^ These substitutions alter the pocket properties, increasing its hydrophilicity and influencing agonist affinity and functional profile.

The distance between the H-bond acceptor in varenicline **1**, nicotine **2**, cytisine **3**, and ACh, and the backbone NH donor of β2L146 (in the α-β pockets) and α4T152 (at the equivalent position within the α-α pocket) and the α4T152 hydroxyl donor in the residue’s side chain, was also determined (Figure S23). These profiles clearly demonstrate that cytisine **3** (and, to a lesser extent, nicotine **2** and ACh) can directly interact with the α4T152 side chain in the α-α site. A role for residues α4H142 and α4Q150, both located on the complementary side of the α-α pocket, was also evaluated, but no interactions between these residues and the ligands were observed (Figure S23). The interaction observed with the side chain of α4T152, specific to the α-α pocket, highlights the distinct nature of this pocket compared to the α-β site and the unique interactions this region can form with agonists. The presence of an α-α pocket in the α4β2 nAChR lowers the overall receptor sensitivity to agonists like varenicline **1**, nicotine **2**, and cytisine **3** (Table 1) but increases their relative efficacy compared to the HS isoform.^72^^,^^73^ Also, the α-α pocket is a known binding site for allosteric modulators, such as NS9283, that increase agonist efficacy.^74^^,^^75^^,^^76^

### Receptor mutations to explore new H-bond interactions within the α4β2 binding sites

As MD simulations suggested that β2S133, α4T183, and α4T139 can sustain hydrogen-bonding interactions with certain agonists (Figures 2 and S20), we posited that removal of the hydroxyl donor(s) from these residues would affect agonist binding (particularly for varenicline **1**) to the α4β2 nAChR, resulting in a decrease in their functional potency (half-maximal effective concentration [EC_50_]) and/or relative efficacy (RE). To test this hypothesis, we employed a valine substitution approach to create the corresponding β2S133V, α4T183V, and α4T139V mutants.

**Figure 3:**
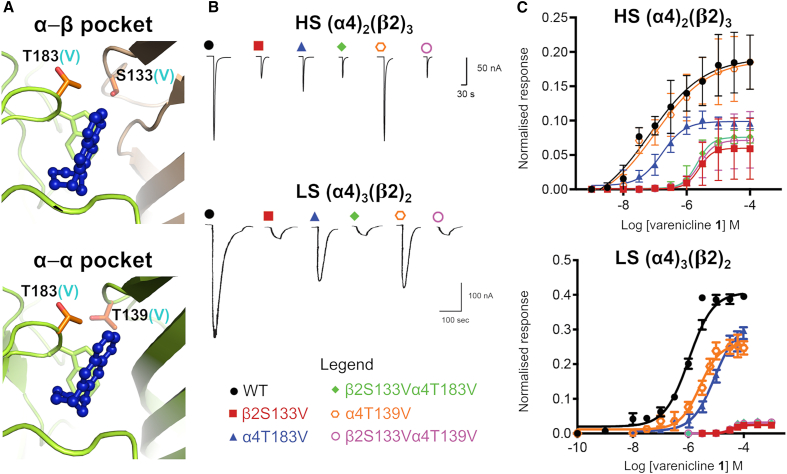
Functional effect of threonine-to-valine mutation with subsequent side-chain hydroxyl removal on varenicline **1** agonism at α4β2 nAChR (A) Location of α4T183, α4T139, and β2S133 in the α-β (top panel) and α-α (bottom panel) binding pockets of the wild-type α4β2 receptor. Residues α4T183, α4T139, and β2S133 were replaced with valine, as indicated by the cyan-colored “(V)” in the residue labels, resulting in the three single mutants β2S133V, α4T183V, and α4T139V, and the two double mutants β2S133Vα4T183V and β2S133Vα4T139V. The α4 and β2 subunits are colored yellow and light brown, respectively. Varenicline **1** is highlighted in dark blue. The side chains of α4T183, α4T139, and β2S133 are represented by orange sticks, whereas TrpB is shown as yellow sticks. (B) Representative current traces of the current responses elicited by varenicline **2** in oocytes expressing heterologously wild-type or mutant HS or LS α4β2 receptors. The current responses were concentration dependent. Full concentration-response curves are shown in (C). (C) Varenicline **1** concentration-response curves for wild-type HS and LS isoforms (data shown in black) and corresponding mutants β2S133V (data shown in red), α4T183V (data shown in blue), β2S133Vα4T183V (data shown in green), α4T139V (data shown in orange), and β2S133V α4T139V (data shown in magenta). Data points in the concentration-response curves represent the mean ± SEM of 8–10 experiments using 6–8 different *Xenopus* donors. Current responses were measured using two-electrode voltage clamping from *Xenopus* oocytes heterologously expressing wild-type or mutant HS/LS α4β2 nAChRs. Peak current amplitudes for all agonists were normalized to the maximal ACh response (1 mM), as described in supplemental methods. Estimated potency (EC_50_) and maximal relative efficacy (RE) parameters are shown in Table 1.

Using oocytes expressing heterologously HS or LS receptor mutants, concentration-response curves were obtained for varenicline **1**, nicotine **2**, cytisine **3**, and ACh. These are shown in Figures 3 and S34–S38, with data summarized in Table 1. The β2S133V significantly reduced the EC_50_ and RE of varenicline **1** at the α4β2 nAChR, with approximately 30-fold decrease in potency at both receptor isoforms. Changes in RE were more pronounced at the LS stoichiometry (with 95% of efficacy loss in LS relative to WT compared to a 67% reduction in the HS), although the difference between isoforms could be due to the challenges of measuring the very low efficacy of varenicline **1** in the β2S133V mutant. The α4T183V mutation also affected the EC_50_ and RE of varenicline **1** at both isoforms. Nonetheless, the effect was less marked than with the β2S133V mutation, with a potency and RE decrease of only 2-fold. Note that the β2S133V mutation is only present in the α-β binding pockets, whereas α4T183V affects both the α-β and α-α sites.

Within the α-α binding site, where α4T139 occupies a position homologous to that of β2S133 in the α-β agonist site, α4T139V reduced functional potency (by approximately 7-fold) and RE (by 50%) of varenicline **1** at the LS receptor. However, this mutation has no effect on agonist binding at the HS stoichiometry, i.e., α4T139 plays no role in the function of the orthosteric α-β site.

When β2S133V was co-expressed with α4T183V or α4T39V, the changes in potency and RE associated with varenicline **1** were generally no different from those of the single mutant β2S133, underscoring the essential functional role of the β2S133-varenicline interaction in α4β2 receptors.

As shown in Table 1 and Figures S36–S38, none of β2S133V, α4T183V, or α4T139V mutations had a significant impact on the agonist profiles of nicotine **2**, cytisine **3**, and ACh at either the HS or LS isoforms. This is consistent with our MD simulations showing that varenicline **1** is the only agonist that can approach closely the hydroxyl group of β2S133 and make a direct H bond with this residue (Figures 2B and S20).

To understand the structural and dynamic effect of the serine/threonine-to-valine mutations described above, MD simulations for the LS isoform incorporating the β2S133V, α4T183V, α4T139V, β2S133Vα4T183V, and β2S133Vα4T139V mutations with varenicline **1**, nicotine **2**, cytisine **3**, and ACh were carried out, with all complexes remaining stable throughout the simulation (Figures S7 and S8). To probe whether the mutations affect the dynamics of the receptor, C_α_ atom fluctuations were determined for both WT and mutant simulations, with all systems showing generally similar profiles, indicating comparable dynamics (Figure S10). Despite this, some regions (e.g., the Cys loop of the β2S133V complexes) showed discernible fluctuation differences compared to WT; however, these differences were not statistically significant (Figure S10).

The impact of the β2S133V, α4T183V, and α4T139V mutations on a range of other relevant interactions associated with ligand-receptor interactions was also assessed (Figures S17–S19 and S24–S26). In our mutant complexes, all agonists remained in their respective binding sites (Figures S11–S16), forming interactions with TrpB, TyrA, and TrpD, with varying frequencies depending on the mutant (Figures S17–S19). The only exception was the ACh molecule in the second α-β pocket of one β2S133Vα4T139V-ACh replicate, which exited the pocket after approximately 112 ns (Figure S13G).

An analysis of the distance between the H-bond acceptor groups within the agonists and H-bond donors present within the mutants’ binding pockets was performed to evaluate how the mutations alter the pattern of interactions with the receptor. As anticipated, the serine/threonine-to-valine mutations redefined the agonist H-bond network within the binding pockets to varying extents (Figures S24–S26). For instance, in the β2S133V mutant, changes in H-bonding profiles were observed in the α-β pockets, with an increase in the frequency of interaction between α4T183 and cytisine **3** (Figure S24). In the α4T183V and α4T139V mutants, a significant increase in the interactions between the ligands and the backbone NH and side-chain OH group of α4T152 in the α-α pocket was observed, with this increase being especially pronounced for varenicline **1** and cytisine **3** (Figure S25). Furthermore, mutations involving α4T183 and α4T139 within the α-α pocket generally resulted in enhanced interactions between varenicline **1** and the side chain of α4Q150 but not α4H142 (Figure S26).

### Exploring varenicline’s structural features that contribute to binding and function in α4β2 nAChR: The role of the heteroaryl moiety

While the ammonium center of varenicline **1** enables the well-characterized cation-π and H-bond donor contacts with the receptor,^26^ potential interactions (e.g., as an H-bond acceptor) involving the heteroaryl (quinoxaline) moiety of this ligand have also been identified from structural studies.^46^^,^^50^^,^^51^^,^^60^ However, given the weakly basic nature of a quinoxaline (p*K*_a_ = 0.6 vs. the pyridyl moiety of nicotine **2**; p*K*_a_ = 3.1 [p*K*_a_ here refers to the p*K*_a_ of the protonated form of the base, i.e., p*K*_a_H, and is used as defined in supplemental methods]), the functional contribution of any interaction involving this group remains unclear. To gain a more complete picture of the role of the quinoxaline moiety of varenicline **1**, we have explored the interactions involving the (hetero)aryl-based group in both receptor recognition (binding) and function (gating). Others, in particular the Pfizer group, have reported heteroaryl variants of varenicline.^28^^,^^37^^,^^77^^,^^78^^,^^79^ However, these generally differ significantly in geometry/size and may incorporate aryl/heteroaryl cores or peripheral substituents capable of enabling additional/different ligand-receptor interactions, which complicates an assessment of the functional role played by the quinoxaline.

**Figure 4:**
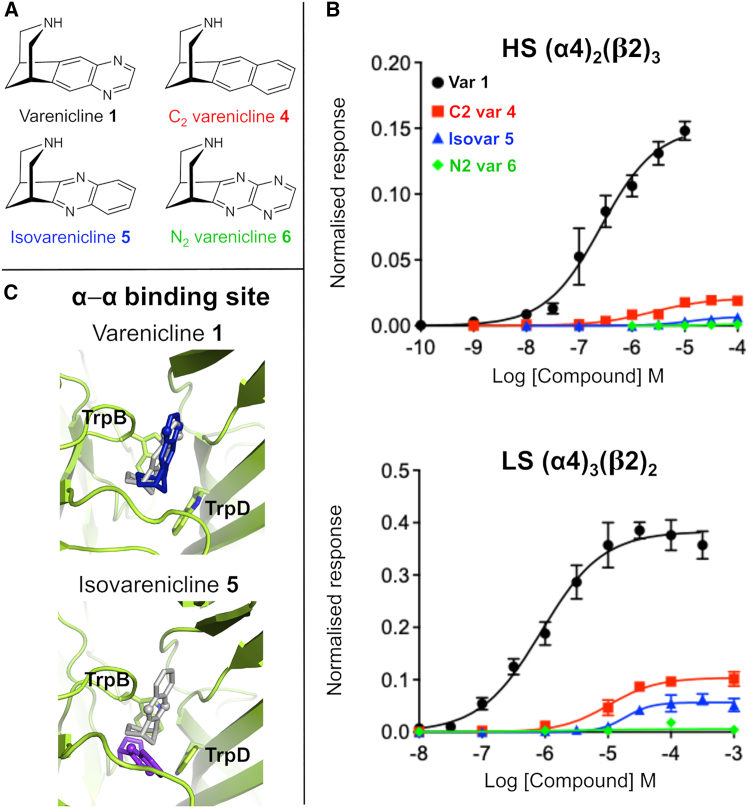
Functional profiles of varenicline **1** and variants **4**–**6** (A) Chemical structure of varenicline **1** and variants C_2_ varenicline **4**, isovarenicline **5**, and N_2_ varenicline **6**. (B) Concentration-response curves for varenicline **1**, C_2_ varenicline **4**, isovarenicline **5**, and N_2_ varenicline **6** for the HS and LS α4β2 isoforms. Concentration-response curves for varenicline **1** and its variants were obtained as described in Figure 3 and supplemental methods. Estimated parameters EC_50_ and maximal relative efficacy (RE) are shown in Table 3. (C) The different binding modes adopted by varenicline **1** and isovarenicline **5** in the α-α pocket after 300 ns of simulation. The gray sticks represent the starting binding mode (after energy minimization) for the agonists (see supplemental methods for a detailed description of how complexes were constructed). The final binding poses for varenicline **1** and isovarenicline **5** are colored blue and purple, respectively, with the nitrogen atoms of the quinoxaline moiety highlighted by spheres. TrpB and TrpD are shown as sticks.

Here, we aimed to focus only on the heteroaryl region of varenicline **1** with two considerations guiding ligand design: (1) retain overall ligand size and shape, as well as the crucial cation-π/H-bond donor components (Figure 1D) within the protonated piperidine unit; and (2) have the ability to include or exclude, or vary, the location of an H-bond acceptor moiety within an otherwise conserved ligand scaffold. These considerations led to the design of three new ligands C_2_ varenicline **4**, isovarenicline **5**, and N_2_ varenicline **6**, as shown in Figure 4A. Importantly, all three ligands retained essentially the same geometry and volume as the parent compound, varenicline **1**, and did not present any additional or significant interactions associated with the structural periphery.

**Scheme 1:**
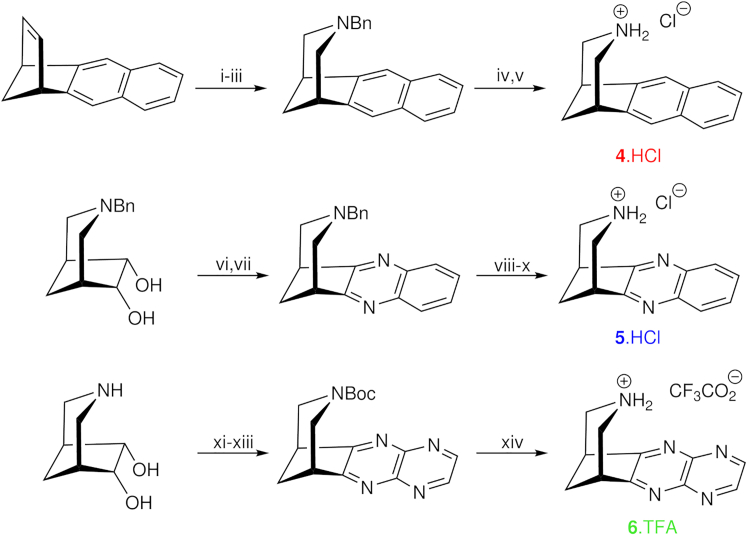
Reagents C_2_ varenicline **4**: i, NMNO, OsO_4_ (cat), acetone, water (98%); ii, NaIO_4_, THF/water; iii, NaBH(OAc)_3_, BnNH_2_ (65% over 2 steps); iv, H_2_, Pd(OH)_2_ (20 wt % on C), Boc_2_O, MeOH/EtOAc (74%); v, HCl in MeOH (quantitative). Isovarenicline **5**: vi, DCC, DMSO, Cl_2_CHCO_2_H, room temperature (rt), 20 h; vii, 1,2-phenylenediamine, rt, 18 h (70% over 2 steps); viii, chloroethyl chloroformate, ClCH_2_CH_2_Cl, 80°C, 18 h; ix, (A) MeOH, reflux 2 h (B) Boc_2_O, rt, 18 h (86% over 2 steps); x, HCl in MeOH, quantitative. N_2_ varenicline **6**: xi, Boc_2_O, Na_2_CO_3_, THF/water, rt, 16 h (90%); xii, TFAA, DMSO, CH_2_Cl_2_, then Et_3_N; xiii, 2,3-diaminopyrazine, MeOH, 65°C, 16 h (38% over 2 steps); xiv, 5% TFA in MeOH (69%).

The synthetic chemistry involved is outlined in Scheme 1, and full details and compound characterization are available in supplemental methods. Each new ligand was isolated as an ammonium salt, which was then used for affinity binding and functional pharmacology studies (Tables 2 and 3).

**Table 2:** Binding affinity constants *K*_i_ for varenicline **1** and ligands **4** and **5**

Ligand	*K*_i_				
	α4β2 (nM)	α3β4 (nM)	α3β4/α4β2	α7 (nM)	α7/α4β2
Varenicline **1**	0.46 ± 0.12	171 ± 41	372	75 ± 22.5	163
C_2_ varenicline **4**	14.3 ± 1.6	267 ± 73	18.7	1,325 ± 595	93
Isovarenicline **5**	5,460 ± 1,280 (5.46 μM)	67,700 ± 37,600 (67.7 μM)	12	126,800 ± 86,000 (126.8 μM)	23

The *K*_i_ values for the human α4β2, α3β4, and α7 nAChRs (as well as ratios relative to α4β2) are shown. The *K*_i_ value for N_2_ varenicline **6** was not determined, due to disruption (and subsequent closure) of the Milan lab owing to the COVID-19 pandemic.

**Table 3:** Agonist sensitivity of HS and LS α4β2 isoforms to varenicline **1**, C_2_ varenicline **4**, isovarenicline **5**, and N_2_ varenicline **6**

Ligand	HS (α4)_2_(β2)_3_		LS (α4)_3_(β2)_2_	
	EC_50_ (μM)	RE	EC_50_ (μM)	RE
Varenicline **1**	0.286 ± 0.1	0.14 ± 0.012	1.01 ± 0.4	0.38 ± 0.025
C_2_ varenicline **4**	2.64 ± 0.8^a^	0.022 ± 0.0033^a^	11.50 ± 1.74^a^	0.102 ± 0.012^a^
Isovarenicline **5**	13.27 ± 2.5^a^	0.0071 ± 0.00086^a^	26 ± 8.5^a^	0.054 ± 0.013^a^
N_2_ varenicline **6**	ND	0.0010 ± 0.0001^a^	ND	0.0032 ± 0.0023^a^

Potency (EC_50_) and relative efficacy (RE) were determined as described in Table 1 and supplemental methods. Statistical differences between varenicline **1** and ligands **4**, **5** and **6** were determined as described in Table 1. ND, not determined due to low levels of functional expression (less than 50 nA of ACh maximal currents). These data are also presented in a graphical format in Figure 4B.

a Statistically significant difference (*p* < 0.05) between varenicline **1** and ligands **4**, **5**, or **6**.

For each variant **4**–**6**, we have assessed (1) ligand affinity constants (*K*_i_) to the human WT α4β2 nAChR (as well as to the human α3β4 and α7 nAChR subtypes); (2) functional (full/partial agonist) profiles at the LS and HS α4β2 isoforms, and compared these to varenicline **1**, nicotine **2**, and cytisine **3**; (3) the effect of the aryl/heteroaryl moieties on the p*K*_a_ of the piperidine amine; and (4) using MD, the optimized binding modes of variants **4** and **5** and their dynamics in the LS and HS α4β2 isoforms relative to varenicline **1**.

(1) Given a requirement for initial recognition, ligand affinity constants for ligands **4** and **5** (together with varenicline **1**) to various nAChRs, including the human α4β2, are shown in Table 2. Comparative *K*_i_ data for human α3β4 and human α7 receptors have also been included, as varenicline also interacts with these subtypes. C_2_ varenicline **4**, while weaker than the parent compound, has a *K*_i_ value within the nanomolar (nM) range and is most potent at the α4β2 subtype; further support for binding of **4** to the α4β2 nAChR comes from the inhibition of ACh by **4** (see Figure S39 and Table S2). A marked decrease in *K*_i_ was observed for isovarenicline **5**, and the relative differences observed at α4β2 for **4** and **5** are replicated at the human α3β4 and α7 subtypes. Pfizer assessed the benzo variant (2,3,4,5-tetrahydro-1H-1,5-methanobenzo[*d*]azepine) of naphthyl-based C_2_ varenicline **4**, which had similar affinity at α4β2 (20 nM vs. 14.3 nM for **4**), although **4** showed higher affinity at α3β4 and α7 nAChR subtypes.^28^ Binding affinity data for N_2_ varenicline **6** could not be directly determined (see Table 2); however, this ligand’s inability to bind was characterized indirectly.^32^ Using α4β2 nAChRs expressed heterologously in *Xenopus* oocytes, N_2_ varenicline **6** displayed very poor efficacy at both HS and LS α4β2 nAChRs (Table 3 and Figure 4B). Further, N_2_ varenicline **6** does not affect ACh current response in oocytes heterologously expressing α4β2 nAChRs (Figure S39). Taken together, these data suggest that N_2_ varenicline **6** does not bind appreciably to α4β2 nAChR.
(2) To determine the functional profile of the new varenicline variants, we have evaluated **1** and **4**–**6** against both the LS and HS stoichiometries of α4β2, with their concentration-response curves shown in Figure 4B and their EC_50_ and RE values in Table 3. These data show a similar trend to that associated with *K*_i_, with similar patterns within the two α4β2 isoforms: C_2_ varenicline **4** shows a (very) weak agonist profile, which reduces significantly further in the case of isovarenicline **5**, and N_2_ varenicline **6** is essentially inactive.
(3) Given the trends that emerged in Figure 4B and, in particular, the obvious discontinuity between the profiles of **1** and **4** vs. **5**, we have determined the p*K*_a_ values of the basic piperidinyl amine center. This was done using a spectrophotometric titration method (see supplemental methods and Figures S42–S49), with results shown in Table 4. As anticipated, the basicity of the ligands decreased in the following order: C_2_ varenicline **4**, varenicline **1**, isovarenicline **5**, and N_2_ varenicline **6**, correlating with an increasingly electron-deficient heteroarene. Amine protonation is a prerequisite for enabling both the cation-π and H-bond donor interactions (Figure 1D) with the conserved aromatic residues within the receptor’s binding sites.^26^ However, the range of p*K*_a_ values observed indicates that amine protonation at physiological pH should not be an issue for C_2_ varenicline **4**, varenicline **1**, and isovarenicline **5** but may be relevant to N_2_ varenicline **6** (see detailed discussion below).

**Table 4:** Experimentally determined p*K*_a_ values for the piperidine amine center of varenicline **1**, C_2_ varenicline **4**, isovarenicline **5**, and N_2_ varenicline **6**

Ligand (salt used)	p*K*_a_	Literature values
Varenicline **1** (tartrate^−^)	8.90 ± 0.1	9.2 (Pfizer^80^), 9.3 (Rollema et al.^81^), 9.22 ± 0.13 (Unal et al.^82^)
C_2_ varenicline **4** (Cl^−^)	9.63 ± 0.08	–
Isovarenicline **5** (Cl^−^)	8.44 ± 0.09	–
N_2_ varenicline **6** (CF_3_CO_2_^−^)	7.31 ± 0.05	–

The specific salt involved is indicated (based on the final deprotection method used in Scheme 1), and commercially available varenicline tartrate was used.
(4) To assess the stability of the ligand-receptor complexes, models for the complexes between ECD of the WT HS and LS α4β2 isoforms and C_2_ varenicline **4** and isovarenicline **5** were constructed and energy minimized. Given the extremely low level of activity and potency observed for ligand **6**, this system was not modeled. For each complex, one ligand molecule was placed in the α-α and α-β binding sites in poses corresponding to those observed in the cryo-electron microscopy (cryo-EM) structure of the human α4β2 nAChR-varenicline complex (PDB: 6UR8^60^). Energy minimized structures of the protonated variant **4** and **5** complexes show that both ligands were optimally located within the receptor’s binding sites in a similar orientation as varenicline **1** (Figures S4 and S5).

Equilibrium MD simulations were performed to assess the dynamics of ligands **4** and **5** when bound to the WT HS and LS α4β2 nAChR (Figures S27 and S28). Like varenicline **1**, variants **4** and **5** remained bound to both the α-β and α-α pockets, forming a stable cation-π interaction with TrpB and sporadic interactions with TyrA and TrpD (Figures S27 and S29). Varenicline **1** and variant **4** exhibited limited mobility within the binding sites, consistently maintaining similar orientations throughout the simulation (Figures S30 and S31). However, variant **5** exhibited (1) notable differences in dynamics compared to varenicline **1** (Figures S27 and S30) and (2) varying levels of mobility between the α-β and α-α pockets, with the ligand displaying high mobility in the latter (Figure S30) and adopting binding modes that were different from the initial one (Figures 4D and S31).

### Role of varenicline heteroarene for agonist profile at 5-HT_3_ receptor

The agonist activity of varenicline **1** vs. the antagonist profile of cytisine **3** at 5-HT_3_ receptors is a key characteristic differentiating these ligands.^34^ Although a full analysis of varenicline’s interactions at 5-HT_3_ receptors falls outside the scope of this study, we used variants **4**–**6** to assess the structural features of varenicline **1** that link to this profile.

The potency of variants **4**–**6** at 5-HT_3_A was assessed using the receptor antagonist [^3^H]GR65630. Compared to varenicline **1**, C_2_ varenicline **4** showed a marked decrease in potency, and a similar trend was observed for both isovarenicline **5** and N_2_ varenicline **6**, with half-maximal inhibitory concentration (IC_50_) values (against [^3^H]GR65630) increased by 44-, 165-, and 250-fold, respectively (Figure S40). Furthermore, none of the variants **4**–**6** elicited a significant functional response at concentrations up to 30 μM in cells expressing 5-HT_3_A receptors (Figure S41).

## Discussion

An attractive pharmacological profile and strong track record as a smoking cessation agent^41^ made cytisine **3** an attractive lead for the development of a proprietary therapeutic, which in 2006 led to the launch of varenicline **1** (Chantix).^17^^,^^18^^,^^19^^,^^20^ However, accumulating evidence now suggests that these two ligands are perhaps not as closely related as initially thought, particularly in terms of their functional mechanisms.^29^^,^^32^^,^^34^^,^^46^ While structurally similar, this relationship may be superficial, and pharmacologically varenicline **1** and cytisine **3** should be considered as “cousins” rather than “siblings.” Auerbach and co-workers have recently shown that varenicline **1** stands out as the first ligand in the “low-efficiency” category (*η* = 33%), whereas typical nAChR ligands, such as ACh, nicotine **2**, and cytisine **3** (as well as anatoxin-a, epibatidine, and epiboxidine) usually show higher efficiencies (*η* = 41%–51%).^83^ Efficiency (*η*), which is a measure of the proportion of binding energy converted to gating, likely reflects the relationship between ligand structure and the specifics of the receptor-ligand interactions that drive binding and receptor activation.^84^^,^^85^

Structurally, varenicline **1** and cytisine **3** both feature a rigid bicyclic core incorporating a piperidyl unit; however, the adjacent heteroaryl moieties (quinoxaline and 2-pyridone, respectively) are quite different. Functionally, these two molecules are also distinct; for instance, although both ligands have similar full agonist profiles at the α7 nAChR,^29^^,^^32^ their profiles differ at the α4β2 subtype, mainly at the HS isoform.^29^^,^^32^ While varenicline **1** acts as a weak partial agonist of the HS isoform, achieving approximately 13%–18% of the maximum efficacy relative to ACh, cytisine **3** is essentially inactive (Table 1).^29^^,^^32^ Another differentiating feature is that varenicline **1** is a potent agonist at the human 5-HT_3_ serotonin receptor, with an efficacy of 80% relative to 5-HT (serotonin), whereas cytisine **3** is an antagonist. While the pharmacological requirements for an effective smoking cessation drug (e.g., roles of the HS vs. LS isoforms; profile at α7) are still to be fully elucidated,^86^^,^^87^ off-target interactions (and associated side effects), such as binding to the 5-HT_3_ receptor, are a factor in reducing end-user compliance.^88^^,^^89^ Therefore, understanding the structure and dynamic basis underpinning functional differences between related ligands, such as varenicline **1** and cytisine **3**, and various receptors is paramount. The work of Dougherty, Lester, and co-workers has demonstrated that, at the molecular level, varenicline **1** does not utilize the same critical set of three receptor-ligand interactions as nicotine **2** or the related compound cytisine **3** to enable receptor activation.^25^^,^^26^^,^^49^ Based on an insightful and selective modification of the protein scaffold, Dougherty showed that, unlike nicotine **2** and cytisine **3**, varenicline **1** does not make a functional interaction with β2Leu146 (via the backbone NH).^49^ This important finding prompted our studies and led us to seek and identify alternative interaction(s) to that “missing” third component associated with varenicline **1** function.

Our efforts have aimed to provide a comprehensive description of the receptor-ligand interaction patterns involving varenicline **1** in order to enhance our understanding of the mode of action of this ligand and ultimately to shed light on the differences associated with varenicline **1** vs. cytisine **3**, such as, e.g., profiles at HS vs. LS 4β2 isoforms and at the 5-HT_3_ receptor. To this end, we have employed a multidisciplinary strategy based on known and novel ligands, integrating biomolecular modeling and simulation; chemical synthesis and physiochemical characterization; receptor binding; and comprehensive functional studies.

Using the experimental structural data available, we constructed models for the complexes between the ECDs of the LS and HS isoforms of the human α4β2 nAChR and varenicline **1**, nicotine **2**, cytisine **3**, and ACh (Figures 2A, S2, and S3). This was followed by extensive MD simulations to uncover potential new functional interactions involving the agonists, particularly varenicline **1**, within the α-β and α-α binding pockets of the α4β2 nAChR. The simulations revealed two new potential interactions associated with the H-bond hydroxyl donor in the side chain of α4T183 and β2S133 within the principal and complementary faces of the α-β binding site (Figure 2B). In the simulations, these residues can form transient H bonds with some ligands, including varenicline **1** and nicotine **2**. Note that, unlike α4T183 (which is present on the principal side of both binding sites), the β2S133 on the complementary face of the α-β site is replaced by α4T139 in the α-α pocket (Figure 1C). These three residues all had the potential to interact with the quinoxaline moiety of varenicline **1** and, thereby, provide a (necessary) third H-bond (receptor donor/ligand acceptor) interaction (in addition to the cation-π and H bond associated with the ammonium center of the ligand) characterized in the Dougherty-Lester model (Figure 1D). The interactions with α4T183, β2S133, and/or α4T139 also require participation of the ligand (as the H-bond acceptor), and, using synthetic chemistry, we have explored further those structural features of varenicline **1** that mediate its binding and function.

Targeted mutagenesis, together with electrophysiological assays, allowed us to assess the relative importance of each of these hydroxyl-containing residues across ACh and varenicline **1** as well as nicotine **2** and cytisine **3**. For this, the polar residues α4T183, β2S133, and α4T139 were mutated to valine, thereby eliminating their side-chain hydrogen-bonding potential. Note that by removing the hydroxyl group (an H-bond donor) from their side chains, the serine/threonine-to-valine mutations prevent α4T183, β2S133, and α4T139 from participating in H-bond interactions with the ligands and/or water molecules. However, the backbone amide NH in these residues, which also serves as an H-bond donor, may still engage in hydrogen bonding. The profiles of varenicline **1**, nicotine **2**, cytisine **3**, and ACh were then determined in the mutant receptors (Figures 3 and S34–S38; Table 1). While the α4T139V and β2S133V mutations, both situated on the α4/β2 complementary face, affect the α-α and the α-β sites individually, α4T183V impacts all binding sites simultaneously due to its position on the principal side of the pocket (Figure 1C).

These studies (Figure 3 and Table 1) demonstrated that, overall, none of the mutations introduced (whether individually or in clusters) affect the ACh profile at either the HS or LS isoforms of the α4β2 receptor. This confirms that α4T183, β2S133, and α4T139 do not play a role in ACh function and further supports Dougherty’s observation that β2L146 (or α4T152 in the α-α pocket) likely accounts for the receptor H-bond donor interaction with ACh.^25^^,^^26^

In contrast to the scenario described above for ACh, varenicline **1** exhibited a significantly different response at the β2S133V mutant, displaying a marked reduction in relative efficacy and showing a 3-fold and 21-fold decrease at the HS and LS β2S133V mutant, respectively, along with concomitant shifts toward higher EC_50_ values (Figure 3 and Table 1). Note that at the HS WT receptor, varenicline **1** is already a relatively weak partial agonist, with an efficacy of only 18% relative to ACh; however, upon introducing the serine-to-valine (β2S133V) mutation in the complementary face of the α-β sites, varenicline **1** shows negligible efficacy (Table 1).

Additionally, the impact of the α4T183V and α4T139V mutations on the functional profile of varenicline **1** was generally less pronounced than that observed for the β2S133V mutation. At the α4T183V receptor, varenicline **1** had a modest loss of efficacy and potency, with about a 2-fold and 6-fold decrease, respectively, at the HS and LS isoforms (Table 1). For the HS form of the α4T139V nAChR, as expected, no effect on the functional profile of varenicline **1** (or any other ligands for that matter) was observed, as this isoform lacks an α-α site; in contrast, the α4T139V mutation had a moderate effect on the efficacy and potency of varenicline **1** in LS isoform, with a level of reduction similar to that observed for the LS α4T183V mutant (Table 1). At the two double mutants, β2S133Vα4T183V and β2S133Vα4T139V, varenicline **1** exhibited again a dramatic reduction in its functional profile (both efficacy and potency), with the magnitude of the changes generally on the same order as those observed in the single-point β2S133V mutant (Table 1).

Based on all the data presented above, we conclude that β2S133 (rather than α4T183 or α4T139), via the H bond formed by its side-chain hydroxyl group (as opposed to the backbone NH, which is still present in the mutants), is the dominant and (up until now unidentified) “third component” required to mediate the function of varenicline **1**. Regarding the roles of β2S133, α4T183, and α4T139 in the function of nicotine **2** and cytisine **3**, the situation is less clear than for varenicline **1**. The effects of the β2S133V, α4T183V, and α4T139V mutations, when present, are markedly more muted compared to those observed for varenicline **1** (Table 1). However, for both nicotine **2** and cytisine **3**, the most impactful interaction still appears to be associated with β2S133, although the changes in the ligand functional profiles in the β2S133V-containing mutants were modest (Table 1). We interpret these results as an ability of nicotine **2** and cytisine **3** to access β2S133 in addition to (but not to the exclusion of) β2L146, which nevertheless remains the optimal partner for mediating the functional profiles of these two ligands. A potential role for β2S133 in mediating the binding of a series of 2-(2-pyrrolidinyl)-1,4-benzodioxane ligands was suggested previously^90^; however, in this case, the role of β2S133 was limited to ligand recognition, with the corresponding β2S133A mutation shown to have no effect on function.

As stated above, the α4T139V mutation, which is the residue associated with the complementary face of the α-α binding site, unsurprisingly had no effect on the functional profiles of the ligands studied at HS receptor isoform and, at best, showed only a minimal impact at the α-α-containing LS stoichiometry, depending on the ligand used (Table 1). This is consistent with the findings of, for example, Balle and co-workers, who have previously shown that α4T139A has only a modest impact on the maximal efficacy of NS9283.^74^

If we consider that, as with the α-β site, a third anchoring connection is required to potentiate activity via the α-α site, then the residue(s) responsible for mediating this interaction remain unclear. The α-α and α-β binding sites are structurally different on the complementary face, as the former is formed by an α4 subunit and the latter by a β2 subunit, with these differences resulting in distinct pharmacological profiles for each site.^22^^,^^67^ Examples of these differences include the substitution of β2S133, β2V136, β2F144, and β2L146 located in loop E of the α-β site by α4T139, α4H142, α4Q150, and α4T152 in the α-α site, respectively.^49^^,^^67^^,^^70^^,^^71^ The MD simulations performed in this study indicate that the hydroxyl moiety of α4T152 in the α-α site can directly interact with the H-bond acceptor groups of certain agonists (Figure S25), therefore suggesting that this residue may potentially serve as the third interaction in this binding site. Previous experimental studies have demonstrated that the α4T152A and α4T152V mutations can decrease the activity of NS9283 several-fold,^74^ supporting the hypothesis that α4T152 can indeed sustain interactions with some ligands. Additionally, given that α-α site residues, such as α4W88 and α4H142, have been identified as key for the gating efficiency of the LS form of the α4β2 receptor,^52^^,^^74^ it would be prudent to say that the precise role of α4T152 in modulating the functional profile of different agonists requires further investigation.

The structure of varenicline **1** and its comparison to cytisine **3** also merit comment. As explained above, the bicyclic piperidine moiety is common to both varenicline **1** and cytisine **3**, but their adjacent heteroaryl units, quinoxaline and 2-pyridone, respectively, differ in terms of the spatial relationships associated with these H-bond acceptor elements. However, given the relatively non-basic nature of the quinoxaline moiety, to comprehend how varenicline **1** exerts its action, two fundamental questions must be addressed: (1) is the quinoxaline unit, as present in varenicline **1**, required for both binding and function, or do those characteristics rely primarily on rigidity and overall shape?; and (2) if the quinoxaline group is essential for function, what evidence is available to support the role of the quinoxaline as an H-bond acceptor within the context of nAChRs?

To answer these questions, we have designed a targeted set of novel varenicline variants, namely C_2_ varenicline **4**, isovarenicline **5**, and N_2_ varenicline **6**. These new ligands, which all incorporate a basic piperidine center to retain the critical cation-π and H-bond donor characteristics, maintain the same size, shape, and molecular volume as the parent compound, varenicline **1** (Figure 4A). This enabled us to avoid added complications associated with additional peripheral substituents to probe receptor-ligand recognition and function and the role played by the aryl moiety in these two connected processes. C_2_ varenicline **4**, which differs from varenicline **1** in containing a naphthalene (lacking an H-bond acceptor) instead of a quinoxaline group (Figure 4A), displays a *K*_i_ of 14.3 nM, corresponding to an approximately 30-fold decrease in affinity compared to the parent compound (Table 2). In terms of function, variant **4** is, however, a very weak partial agonist showing a 10-fold increase in EC_50_ at both HS and LS isoforms of α4β2, together with reduced efficacy, with a 6- and 4-fold reduction at HS and LS, respectively (Table 3). We conclude from these results that while the quinoxaline moiety (as present in varenicline) enhances binding, it is not a prerequisite, with size, rigidity, and shape (and the interactions associated with the ammonium center) being primary determinants for recognition. Further, the MD simulations performed suggest that when bound to the α4β2 nAChR, variant **4** exhibits comparable dynamics and adopts similar binding modes to those of varenicline **1** (Figures S30 and S31), with the biggest differences between the two ligands arising from the lack of the H bonds associated with the naphthalene group. However, the presence of a quinoxaline unit within varenicline **1** is an essential requirement for function (Table 3), therefore showing that this structural component contributes to additional interaction(s) unavailable to C_2_ varenicline **4**. In this regard, it is noteworthy that C_2_ varenicline **4** exhibits an identical pattern of functional responses at both the HS WT and mutant β2S133V/α4T183V receptors (Tables 1 and 2). Both C_2_ varenicline **4** and these receptor variants preserve the capacity to form cation-π and hydrogen-bond interactions but lack the distinctive third interaction characteristic of varenicline **1**. This observation supports the conclusion that the quinoxaline moiety in varenicline mediates an additional, functionally critical interaction that is absent in variant **4**. For the LS isoform, where the α-α binding site present is not subject to mutation, EC_50_ values are, nevertheless, still very similar.

In the case of isovarenicline **5**, where the orientation of the quinoxaline moiety has been reversed (Figure 4A), a step change in both binding and function at α4β2 was observed (Tables 2 and 3). Variant **5** binds very weakly to α4β2 (high μM, which was also observed at both the α3β4 and α7 subtypes) and also shows markedly reduced efficacy, with a 20-fold and 7-fold decrease at HS and LS isoforms, respectively. MD simulations associated with variant **5** align with these findings, revealing the ligand to be less stable when bound to α4β2, adopting configurations markedly different from varenicline **1** (Figures 4C, S30, and S31) and, importantly, forming alternative interaction networks within the binding pockets (Figures S32 and S33). A clear distinction between the interaction networks for varenicline **1** vs. isovarenicline **5** is the loss of H bonding to the side chain of β2S133 and the emergence of a frequent and stable interaction with the side chain of α4T152 in the α-α pocket (Figures S32 and S33). As discussed, the interaction between varenicline **1** and β2S133 is essential for the ligand functional activity (Table 1), and its absence for variant **5** likely contributes to its diminished efficacy. Taken together, the experimental and computational findings allow us to answer the first of the two questions posed above, namely, whether the quinoxaline in varenicline **1** is necessary for binding and function. Our results indicate that both the incorporation and the specific orientation of the quinoxaline unit within varenicline **1** is essential for function.

The results reported here for C_2_ varenicline **4** and isovarenicline **5** also shed light on the second question raised above regarding the role of the quinoxaline as an H-bond acceptor. Our findings demonstrate that H bonding, either direct or water mediated, between the quinoxaline group and the protein is crucial for varenicline function. Further compelling experimental evidence that this key third varenicline **1** interaction is based on quinoxaline acting as an H-bond acceptor comes from crystallographic data for the *Capitella teleta* AChBP-varenicline **1** complex (PDB:4AFG),^51^ a complex analogous with that of the serotonin binding protein from *Aplysia californica* (PDB: 5AIN)^46^ as well as the structure of varenicline **1** bound to the iCytSnFR cytisine sensor precursor binding protein (PDB: 7S7X).^91^ Although they are not functionally significant, in all of these cases, water (and also tyrosine)-varenicline **1** interactions involving the quinoxaline N atoms of varenicline are evident. Furthermore, our findings underscore the importance of the precise spatial positioning of the quinoxaline unit in varenicline **1**, as its interactions with the protein must occur within a specific, well-defined region of the binding site. Altering the orientation of the quinoxaline group (as in isovarenicline **5**) substantially disrupts this interaction network and, subsequently, receptor function.

N_2_ varenicline **6** incorporates a 1,4,6,8-tetraazanaphthalene moiety, which we anticipated to be much less available as an H-bond acceptor than the quinoxaline of varenicline **1** (Figure 4A).^92^ Variant **6** showed no evidence of binding (Figure S39), nor was activity detected at either HS or LS isoforms (Table 3). A likely explanation for this lack of binding is, we suggest, related to the p*K*_a_ of the piperidine group in N_2_ varenicline **6** (Table 4). Given that piperidine protonation in varenicline **1** is necessary for the cation-π and H-bond donor/acceptor interactions with TrpB in the principal side of the α-β pockets,^26^ the affinity constant (*K*_i_) and p*K*_a_ values for varenicline **1** and variants **4**–**6** were determined (Tables 2 and 4, respectively). A significant decrease in *K*_i_ was observed between C_2_ varenicline **4** (p*K*_a_ = 9.63) and isovarenicline **5** (p*K*_a_ = 8.44), despite no meaningful differences in the protonation state of their ammonium centers at physiological pH; both ligands remain ≥90% protonated at pH 7.5. Moreover, isovarenicline **5** is more basic than either nicotine **2** (pyrrolidine p*K*_a_ = 7.80)^93^ or cytisine **3** (p*K*_a_ = 8.20),^94^ further supporting that protonation differences are not linked to reduced binding affinity. Rather, the functional profile of variant **4** is attributed to its inability to act as an H-bond acceptor. In contrast, the decrease in functional profile behavior of isovarenicline **5** is associated with distinct interaction patterns that it forms within the binding sites compared to varenicline **1**. These encompass the formation of contacts with α4T152 and the loss of those involving β2S133 (Figures S32 and S33). This finding highlights the importance of the precise H-bond acceptor position within varenicline **1**, where an interaction with β2L146 is precluded by distance, yet the ligand can compensate by engaging in an alternative functional mechanism via β2S133.

Finally, the very poor H-bond acceptor characteristics of the 1,4,5,8-tetraazanaphthyl group in N_2_ varenicline **6** would suggest a functional profile resembling that of naphthyl-based C_2_ varenicline **4**. However, this is not the case, with the inactive profile of variant **6** likely associated with the low p*K*_a_ of the piperidine amine (p*K*_a_ = 7.31), indicating that protonation may be impaired.^95^

**Figure 5:**
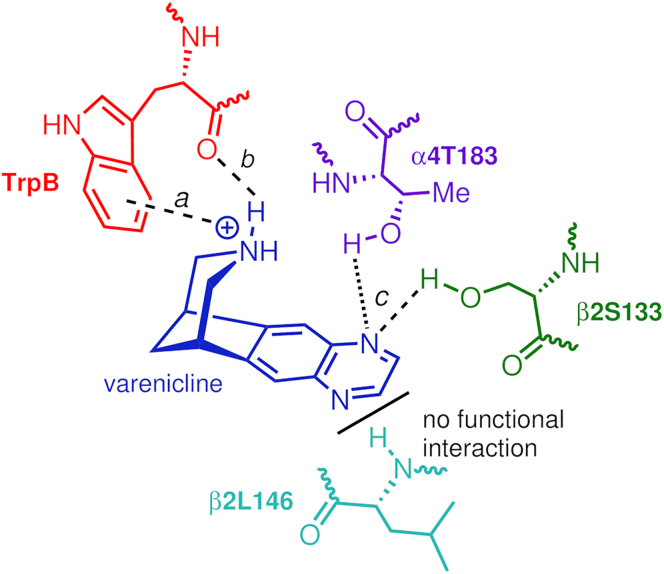
Expanded version of the Dougherty-Lester nAChR binding model for varenicline **1** in the α-β pocket of α4β2 nAChR, highlighting the three key functional interactions (a) cation-π and (b) backbone C=O as H-bond acceptor associated with TrpB within the α4 subunit,^23^^,^^24^^,^^25^ and (c) the newly identified H-bond donor-acceptor interaction between the quinoxaline moiety of varenicline **1** and the side-chain OH group of β2S133 (in the complementary subunit). The less prominent interaction involving the OH of α4T183 is also included in the scheme, as it can occur in the absence of a functional connection involving the backbone NH of β2L146. Note that although the new ligand-protein contacts involving β2S133 and α4T183 are depicted as being direct, the possibility of a bridging water molecule mediating these interactions cannot be excluded.

Given that the viability of varenicline **1** to participate as an H-bond acceptor within similar receptor binding environments is now firmly established, and based on the data for the mutants above (Table 1), we propose that the key interactions of the quinoxaline moiety of varenicline **1** involve β2S133 in the complementary side and (to a lesser degree) α4T183 in the principal side of the α-β binding sites within the α4β2 nAChR. Furthermore, building on previous findings by Dougherty and colleagues showing that varenicline **1** does not rely on β2L146 to enable function,^49^ we posit that the binding model of varenicline **1** to the α-β sites of the α4β2 subtype, which retains both the cation-π and H-bond donor components derived from the protonated secondary amine center in the ligand, can now be expanded to include an H-bond donor-acceptor interaction (as the “missing” third component) and that this previously unrecognized interaction involves the quinoxaline heterocycle with chiefly β2S133, as illustrated in Figure 5.

We have also probed the ligand requirements of the 5-HT_3_ receptor: compared to varenicline **1**, ligands **4**–**6** exhibit far weaker interactions and fail to activate 5-HT_3_ (Figures S40 and S41). From these findings, we conclude that the precise location of the quinoxaline moiety within the varenicline scaffold is again a critical determinant of varenicline’s profile at 5-HT_3_ receptors.

In conclusion, we have characterized the “missing interaction” associated to date with modulation of the partial agonist profile of varenicline **1** at the α4β2 receptor, the primary target in nicotine addiction. We have demonstrated that the quinoxaline moiety within varenicline **1** engages in functionally relevant interactions with α4β2 nAChR. In its α-β binding sites, these interactions are highly specific and well defined, involving the hydroxyl side chains of primarily β2S133 and (to a lesser degree) α4T183. Given that H-bond donor groups at positions equivalent to β2S133 and α4T183 are highly conserved across human neuronal nAChR subunits, it is plausible to suggest that these newly characterized interactions contribute to the functional profile of varenicline **1** (and potentially other agonists) across a range of neuronal subtypes, such as α7 and α3β4. This, in our opinion, warrants further investigation, as uncovering the functional interactions of each agonist will strengthen the foundation for rational drug design targeting these proteins. The ability of varenicline **1** to exploit alternative and functionally relevant binding patterns within neuronal receptors, distinct from that of its progenitor cytisine **3**, may directly account for the divergence in functional profiles observed for these two ligands. This is particularly relevant for the human 5-HT_3_ receptor, where cytisine **3** is 2,000-fold less potent than varenicline **1**.^96^

## Methods

Full details of synthetic chemistry, computational chemistry, nAChR ligand binding, nAChR functional studies, 5-HT_3_ functional studies, and p*K*_a_ determinations are provided in supplemental methods.

## Resource availability

### Lead contact

Requests for further information and resources should be directed to and will be fulfilled by the lead contact, A. Sofia F. Oliveira (sofia.oliveira@bristol.ac.uk).

### Materials availability

Subject to availability, ligands **4**, **5**, and **6** generated in this study are available upon request from Timothy Gallagher (t.gallagher@bristol.ac.uk).

### Data and code availability

All MD data (including input and trajectory files) are publicly available via the University of Bristol Research Data Repository (https://data.bris.ac.uk/).

## Acknowledgments

A.S.F.O. was supported at the University of Bristol by a BBSRC Discovery Fellowship (BB/X009831/1). F.V. was supported by ANID Becas Chile 72210124. We thank Achieve Life Sciences for a gift of (−)-cytisine and 10.13039/501100000266EPSRC (EP/N024117/1) for financial support. T.G. thanks Dr. Jack Rogers for support and Professor Varinder Aggarwal for access to laboratory facilities at the University of Bristol. We thank EPSRC for providing ARCHER2 time via HECBioSim (hecbiosim.ac.uk). Data analysis was conducted using the facilities of the Advanced Computing Research Center at the University of Bristol (https://www.bris.ac.uk/acrc/).

## Author contributions

A.S.F.O. and T.C.G. conceptualized the project. S.G.A., D.F., M.H., G.P., and T.G. performed the synthetic chemistry. J.M., J.R., and A.C.O.’D. carried out the pKa determinations. A.S.F.O. conducted the molecular dynamics simulations, and both A.S.F.O. and J.U. performed the analysis. C.G. performed the nACh receptor binding experiments, and I.B., F.V., and T.M.V. carried out the nACh receptor functional assays and functional data analysis. S.C.R.L. performed the 5-HT_3_ receptor functional assays and functional data analysis. All authors contributed to the preparation of the manuscript.

## Declaration of interest

The authors declare no competing interests.

## Supplemental information

Document S1. Figures S1–S49, Tables S1–S4, Schemes S1–S3, Data S1, and supplemental methods

Document S2. Article plus supplemental information
