# Supplementary material for: Understanding varenicline function via key receptor and ligand interactions
Source: Cell Rep Phys Sci. 2025 Dec 17;6(12):102992. doi: 10.1016/j.xcrp.2025.102992 (PMC12711630; doi:10.1016/j.xcrp.2025.102992)
Supplement: Document S1. Figures S1–S49, Tables S1–S4, Schemes S1–S3, Data S1, and supplemental methods [file mmc1.pdf]

Cell Reports Physical Science, Volume *6*

Supplemental information

Understanding varenicline function

via key receptor and ligand interactions

Sheenagh G. Aiken, Daniele Fiorito, Matthew Harper, Grzegorz Pikus, Juno Underhill, Jacob Murray, Joshua Rawlinson, AnnMarie C. O'Donoghue, Cecilia Gotti, Sarah C.R. Lummis, Teresa Minguez Viñas, Franco Viscarra, Isabel Bermudez, Timothy Gallagher, and A. Sofia F. Oliveira

Supplemental Methods

A. Synthetic Chemistry — **SI 2 – SI 39**

- *(i)* General information
- *(ii)* Synthetic procedures and characterization data; x-ray crystallographic details of N-Boc isovarenicline **S18**
- *(iii)* Data S1: ^{1}H and ^{13}C NMR spectra of key intermediates and final products

B. Computational Modelling — **SI 40 – SI 83**

- *(i)* Molecular dynamics (MD) simulations
- *(ii)* Analysis of MD simulations
- *(iii)* Supporting figures and tables

C. nAChR Ligand Binding Measurements — **SI 84 – SI 86**

- *(i)* Expression of human α4β2, α3β4 and α7 nAChR
- *(ii)* Radioligand binding assays
- *(iii)* Competition binding assays
- *(iv)* Statistical analysis

D. nAChR Methods and Functional Studies — **SI 87 – SI 96**

- *(i)* Animals
- *(ii)* Human α4β2 nAChR expression in *Xenopus* oocytes
- *(iii)* Single and double mutations
- *(iv)* Electrophysiological recordings
- *(v)* Statistical analysis
- *(vi)* Supporting figures and tables

E. 5-HT_3 Methods and Functional Studies — **SI 97 – SI 99**

- *(i)* Cell culture
- *(ii)* Radioligand binding
- *(iii)* FlexStation analysis
- *(iv)* Data analysis
- *(v)* Supporting figures

F. pK_a Determinations — **SI 100 – SI 106**

- *(i)* Experimental description of the materials and assays
- *(ii)* Theoretical background
- *(iii)* Spectrophotometric method validation: 4-dimethylaminopyridine (DMAP) and nicotine **2**
- *(iv)* Spectrophotometric titration of varenicline **1**, nicotine **2**, cytisine **3** and varenicline variants **4**-**6**

G. Supplemental Methods References — **SI 107 – SI 109**

A. Synthetic Chemistry

(i) General information

Reactions requiring inert conditions were conducted under an N_2 atmosphere using standard Schlenk-line techniques. Anhydrous solvents were obtained from an Anhydrous Engineering alumina column drying system or from distillation following standard procedures. All other reagents were purchased from commercial suppliers and used as received. Thin layer chromatography was performed using aluminum backed 60 F254 silica plates. Visualization was achieved by UV fluorescence or a basic $KMnO_4$ solution and heat.

Infrared spectra were recorded using a Perkin Elmer Spectrum Two FT-IR spectrometer.

NMR spectra were recorded on Bruker Advance III HD 500 Cryo, Varian 400-MR, Jeol ECS 400 or JEOL ECZ 400 spectrometers. Chemical shifts (δ) are quoted in parts per million (ppm) and are referenced to the residual solvent peak, coupling constants (*J*) are given in Hz. Multiplicities are abbreviated as: br (broad), s (singlet), d (doublet), t (triplet), q (quartet), m (multiplet) or combinations thereof. Assignments (when indicated) were made with the aid of COSY, HSQC, HMBC experiments.

Mass spectrometry was performed by the University of Bristol mass spectrometry service by either (EI^+) using a VG Micromass Autospec spectrometer or by electrospray ionization (ESI+) using a Bruker Daltonics MicrOTOF II spectrometer.

Numbering system (shown here for varenicline **SI0**) as used for $^1H/^{13}C$ NMR structural assignments of varenicline variants.

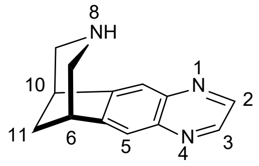

SI0 Varenicline

Structures are numbered based on the Schemes shown below except where a structure (varenicline variants) appears in the published manuscript, and the number used here corresponds to that used in the main paper.

(ii) Synthetic procedures and characterization data

1. C_2 Varenicline 4

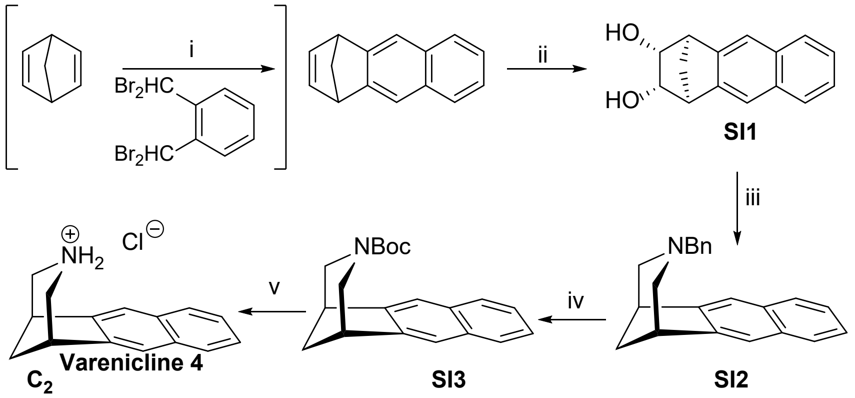

SI Scheme 1: *Reagents*: i, KI, xylene, 65 °C, 18h[1] (35%) [not included in main manuscript]; ii, NMNO, OsO_4 (cat), acetone, water (98%); iii, (a) $NaIO_4$, THF/water[2, 3] then (b) $NaBH(OAc)_3$, $BnNH_2$ (65% over 2 steps); iv, H_2, $Pd(OH)_2$ (20% wt on C), Boc_2O, MeOH/EtOAc (74%); v, HCl in MeOH (quantitative).

***N*-Bn C_2 Varenicline SI2**

exo-1,4-Methano-1,2,3,4-tetrahydro-2,3-dihydroxyanthracenedihydroxyanthracene **SI1** (222 mg, 0.98 mmol) was dissolved in THF:H_2O (2.5:1, 20 mL) then $NaIO_4$ (230 mg, 1.08 mmol) was added in a single portion. The mixture was stirred at rt for 30 minutes then H_2O (40 mL) was added. The aqueous phase was extracted with CH_2Cl_2 (4 × 10 mL), the extracts were dried ($MgSO_4$) and concentrated to give the crude dialdehyde as a yellow oily paste. The dialdehyde was dissolved in anhydrous CH_2Cl_2 (20 mL) under an N_2 atmosphere then cooled to 0 °C. Sodium triacetoxyborohydride (830 mg, 3.92 mmol) was added followed by benzylamine (118 µL, 1.08 mmol) dropwise. The mixture was allowed to slowly warm to rt overnight, quenched with saturated aq. Na_2CO_3 (15 mL) and H_2O (20 mL). The product was extracted with CH_2Cl_2 (3 × 20 mL), the combined extracts were washed with brine (30 mL), dried ($MgSO_4$) and concentrated. Purification by silica chromatography (Biotage; 5% to 15% EtOAc in hexane) gave the ***N*-Bn C_2 varenicline SI2** (190 mg, 65%) as a colorless oil. ^1H NMR (400 MHz, $CDCl_3$) δ 7.83 – 7.76 (m, 2 H, C10/13-H), 7.55 (s, 2 H, 5/12-H), 7.44 – 7.37 (m, 2 H, 2/3-H), 7.14 – 7.06 (m, 3 H, Ph), 6.90 – 6.81 (m, 2 H, Ph), 3.48 (s, 2 H, $PhCH_2$), 3.24 (t, *J* 4.4 Hz, 2 H, 6/10-H), 2.98 – 2.89 (m, 2 H, 7/9-H), 2.53 (d, *J* = 10.3 Hz, 2 H, 7/9-H), 2.27 (m, 1 H, 11-H), 1.79 (d, 1 H, *J* 10.5

Hz, 11-H); ^{13}C NMR (126 MHz, $CDCl_3$) δ 146.0 (C5a), 138.8 (Ph), 133.5 (C4a), 128.5 (Ph), 128.1 (Ph), 127.8 (C1), 126.6 (Ph), 124.8 (C2), 119.3 (C5), 61.8 ($PhCH_2$), 57.7 (C7), 43.6 (C11), 41.3 (C6); HRMS (ESI): calculated for $C_{22}H_{22}N$ $[M+H]^+$: 300.1747, found: 300.1733.

***N*-Boc C$_2$ Varenicline SI3**

To a solution of *N*-Bn C_2 varenicline **SI2** (176 mg, 0.588 mmol) in MeOH:EtOAc (1:1, 12 mL) was added Boc_2O (0.270 mL, 1.17 mmol) and $Pd(OH)_2$ (20 wt% on carbon, 83 mg). The mixture was stirred rapidly under an atmosphere of hydrogen at room temperature for 24 h, after which the mixture was filtered through Celite. The solids were washed with CH_2Cl_2 (50 mL), the filtrate was concentrated and purification by silica chromatography (Biotage; 2% to 30% EtOAc in hexane) gave ***N*-Boc C$_2$ varenicline SI3** (135 mg, 74%) as a colorless solid. FTIR v_{max} / cm^{-1} (neat): 1691; ^{1}H NMR (400 MHz, $CDCl_3$; broadening and splitting of some signals due to amide resonance was observed) δ 7.80 – 7.75 (m, 2 H, 1/4-H), 7.64 (s, 1 H, 5/12-H), 7.61 (s, 1 H, 5/12-H), 7.43 – 7.37 (m, 2 H, 2/3-H), 4.07 (d, *J* 12.5 Hz, 1 H, 7/9-H), 3.94 (d, *J* 12.5 Hz, 1 H, 11-H), 3.36 – 3.25 (m, 3 H, 2 × 6/10-H, 7/9-H), 3.21 (d, *J* = 12.4 Hz, 1 H, 7/9-H), 2.35 (m, 1 H, 11-H), 1.94 (d, *J* 10.8 Hz, 1 H, C11-H), 1.16 (s, 9 H); ^{13}C NMR (126 MHz, $CDCl_3$) δ 156.1 (C=O), 143.9/143.8 (C5a, rotamers), 133.64/133.59 (C4a, rotamers), 128.1/127.7 (C1, rotamers), 125.3/125.2 (C2, rotamers), 121.2/120.6 (C5, rotamers), 79.3 ($CCMe_3$), 50.5/49.4 (C7, rotamers), 41.6 (C11), 40.1/40.0 (C6, rotamers), 28.3 ($CCMe_3$); HRMS (ESI): calculated for $C_{20}H_{24}NO_2$ $[M+H]^+$: 310.1802, found: 310.1814.

C$_2$ Varenicline hydrochloride salt 4

N-Boc C_2 varenicline **SI3** (24 mg, 0.078 mmol) was dissolved in HCl (4 mL, 0.5 M in MeOH) and allowed to stand for 18 h at rt. The mixture was concentrated to afford the title compound **C$_2$ varenicline hydrochloride 4** (19 mg, quantitative) as an off-white solid. ^{1}H NMR (400 MHz, D_2O), δ 8.02 – 7.96 (m, 2H, 1-H), 7.94 (s, 2H, 5-H), 7.63 – 7.57 (m, 2H, 2-H), 3.67 – 3.60 (m, 2H, 7-H), 3.52 (d, *J* = 12.3 Hz, 2H, 6/10-H), 3.37 (d, *J* = 12.3 Hz, 2H, 6/10-H), 2.44 (m, 1H, 11-H), 2.20 (d, *J* = 11.6 Hz, 1H, 11-H); ^{13}C NMR (126 MHz, D_2O) δ 140.5 (C4), 133.6 (C6), 128.0 (C7), 126.2 (C8), 122.5 (C5), 47.8 (C1), 40.2 (C3), 38.0 (C2); HRMS (ESI+): Calculated for $C_{15}H_{16}N$ $[M+H]^+$: 210.1283, found: 210.1268.

2. Isovarenicline 5

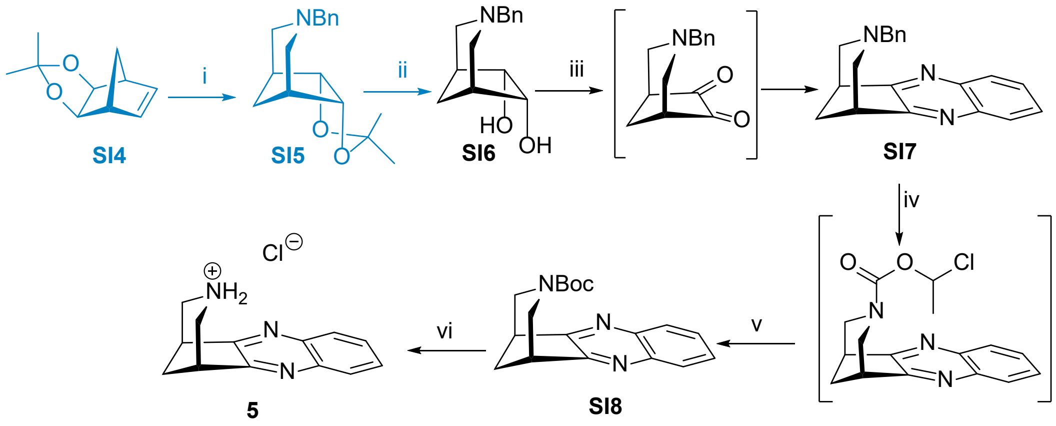

SI Scheme 2: *Reagents*: i, (a) O_3, DCM, -78 °C then Me_2S (b) $NaBH(OAc)_3$, $BnNH_2$, DCM, rt, 35% overall; ii, HCl, THF, 81%; iii, (a) DCC, DMSO, Cl_2CHCO_2H, rt, 20h; iv, (b) 1,2-phenylenediamine, rt, 18h, 70% over 2 steps; vi, chloroethyl chloroformate, $ClCH_2CHCl$ 80 °C, 18h; v, (a) MeOH, reflux 2h (b) Boc_2O, 86% over 2 steps; vi, HCl in MeOH, quantitative OR (+)-tartaric acid, EtOH/acetone 76%

Note that diol **SI6** was available commercially but synthetic details for conversion of S14 to S16 are provided here given the specialized nature of the commercial supply used.

N-Benzyl 3-azabicyclo[3.2.1]octane-6,7-diol acetonide S15

To a solution of acetonide **SI4**[4] (1.0 g, 6.0 mmol) in DCM (12 mL), ozone was bubbling at -78 °C until the blue colour appeared/persisted. The excess of ozone was removed by a stream of nitrogen at -78°C, and after that, dimethylsulfide (0.95 mL, 12.4 mmol) was added. The reaction mixture warm to rt and stirred for 16h. The solvent was removed to give a transparent oil, which was dissolved in dry DCM (80 mL) and sodium triacetoxyborohydride (5.10 g, 24.18 mmol) was added. The mixture was cooled to 0 °C and a solution of benzylamine (0.71 g, 0.73 mL) in dry DCM (20 mL) was added dropwise over 30 minutes. The mixture was warmed to rt, stirred for 18h, then washed with water (30 mL) and brine (30 mL) and dried ($MgSO_4$). The solvent was removed and the crude product was purified by silica gel chromatography (ethyl acetate:hexane) to give **acetonide SI5** (575 mg, 35%) as a colorless oil, which was used without additional purification. ^1H NMR (400 MHz, $CDCl_3$): δ 7.25-7.13 (m, 5H), 4.44 (d, *J*=1.6 Hz, 2H), 3.33 (s, 2H), 2.67-2.63 (m, 2H), 2.08 (t, *J* 4 Hz, 2H), 1.99 (d, *J* 12 Hz, 2H), 1.87 (m, 1H), 1.36 (s,

3H), 1.28 (s, 3H), 1.09 (d, *J* = 12 Hz, 1H); ^{13}C NMR (100 MHz, $CDCl_3$): δ 138.6, 128.6, 128.2, 126.9, 108.5, 83.4, 62.5, 56.1, 40.3, 31.6, 25.9, 23.8; R_f: 0.70 (30% ethyl acetate in hexane).

N-Benzyl 3-azabicyclo[3.2.1]octane-6,7-diol SI6

Acetonide **SI5** (663 mg, 2.43 mmol) was dissolved in THF (12 mL) and 4M HCl (12 mL) was added. The mixture was stirred at 80°C for 72h, after which time 10% aq. $NaHCO_3$(30 mL) was added and pH was adjusted to pH 10 by addition of aqueous Na_2CO_3 dropwise. The aqueous solution was extracted with ethyl acetate (3 x 30 mL), and the extracts were dried (Na_2SO_4) and concentrated. Purification of the residue by silica gel chromatography (hexane:ethyl acetate) gave **diol SI6** (459 mg, 81%) as a colorless oil that solidified, and could be further purified by recrystallization from ethyl acetate. ^{1}H NMR (400 MHz, $CDCl_3$): δ 7.23-7.12 (m, 5H), 4.11 (s, 2H), 3.32 (s, 2H), 3.19 (bs, 2H), 2.69-2.65 (m, 2H), 2.02-2.00 (m, 2H). 1.94 (d, *J* = 8Hz, 2H), 1.90-1.52 (m, 1H), 1.07 (d, *J* = 8 Hz, 1H); ^{13}C NMR (100 MHz, $CDCl_3$): δ 138.8, 128.6, 128.1, 126.9, 75.2, 62.4, 57.1, 43.6, 32.0; R_f: 0.41 (50% ethyl acetate in hexane); MS (ESI): calculated for $[C_{14}H_{20}NO_2]^+$: 234.1494, found $[M+H]^+$: 234.1490.

***N*-Benzyl isovarenicline SI7** (via Pfitzner–Moffatt oxidation).

A solution of N-benzyl 3-azabicyclo[3.2.1]octane-6,7-diol **SI6**[4] (250 mg, 1.07 mmol) in DMSO (2 mL) was added to a mixture of DCC (1.77 g, 8.57 mmol) in DMSO (10 mL) followed by dichloroacetic acid (221 mg, 140 μL, 1.72 mmol). The reaction mixture was stirred at rt for 20h, filtered through celite and the solids were washed with EtOAc (50 mL). EtOAc was removed under reduced pressure and to the resulting orange solution was added 1,2-phenylenediamine (116 mg, 1.07 mmol). The mixture was stirred at rt for 18h, water (150 mL) was then added and the product was extracted with EtOAc (3 x 50mL). The extracts were washed with water (150 mL) and brine (150 mL), dried (Na_2SO_4) and after removal of solvents, the residue was purified by chromatography (hexane: EtOAc 9:1 → 7:3) to give **N-benzyl isovarenicline SI7** (224 mg, 70%) as a pale orange solid. ^{1}H NMR (400 MHz, $CDCl_3$): δ 7.99-7.95 (m, 2 H), 7.63-7.59 (m, 2 H), 7.04-6.89 (m, 3 H). 6.72-6.68 (m, 2 H), 3.38 (s, 2 H), 3.26 (t, 2 H, = 8 Hz), 3.15- 3.11 (m, 2 H), 2.61 (d, 2 H, = 8 Hz), 2.34 (m, 1 H), 1.92 (d, 1 H, = 12 Hz); ^{13}C NMR

(100 MHz, $CDCl_3$): δ 163.3, 141.7, 137.4, 128.8, 128.4, 128.3, 128.0, 126.8, 61.5, 57.0, 41.1, 39.6; MS (ESI): calculated for $[C_{20}H_{20}N_3]^+$: 302.1652, found $[M+H]^+$: 302.1650.

[Using **SI6** and analogous Swern oxidation conditions (DMSO/TFAA/DCM at -78 °C), we isolated **SI7** in 44% yield.]

***N*-Boc isovarenicline SI8**

Chloroethyl chloroformate (251 μL, 2.32 mmol) was added to a solution of N-benzyl isovarenicline **SI7** (100 mg, 0.332 mmol) in 1,2-dichloroethane (7 mL) and the mixture was stirred at 80°C for 20 h. After cooling to rt and removal of solvent, the residue was dissolved in MeOH (7 mL) and heated under reflux for 2h.** After cooling, di-*tert*-butyl dicarbonate (87 mg, 0.4 mmol) was added and the mixture was stirred at rt for 18h. After concentration, purification by chromatography (hexane:EtOAc 7:3 → 5:5) gave **N-Boc isovarenicline SI8** (89 mg, 86%) as a colorless solid. ^1H NMR (400 MHz, $CDCl_3$; broadening due to amide resonance was observed): δ 7.99-7.96 (m, 2 H), 7.65-7.60 (m, 2 H), 4.23-4.10 (m 2 H), 3.40-3.25 (m, 4 H), 2.49-2.43 (m, 1 H), 2.05 (d, = 12 Hz, 1 H), 1.14 (s, 9 H); ^{13}C NMR (100 MHz, $CDCl_3$): δ 161.4, 155.6, 142.2, 129.1, 80.1, 49.3, 48.3, 40.0, 38.1, 28.2; MS (ESI): calculated for $[C_{18}H_{21}N_3O_2Na]^+$: 334.1527, found $[M+Na]^+$: 334.1526.

** At this point, the solvent can be evaporated, and the residue was filtered through a plug of silica (EtOAc as eluent) to provide isovarenicline **5** (as the free base) that was judged (by TLC and ^1H NMR) to be sufficiently pure to use directly to prepare the tartrate salt (see below). The advantage of the N-Boc intermediate **SI8** is its ease of purification.

The structure of N-Boc isovarenicline **SI8** was confirmed by X-ray crystallographic analysis (Figure S1). X-ray diffraction experiments of **S18** were carried out at 100(2) K on a Bruker APEX II diffractometer using Mo-Kα radiation (λ = 0.71073 A) and a CCD area detector. Intensities were integrated in SAINT[a] and absorption corrections based on equivalent reflections were applied using SADABS.[b] The structure was solved using ShelXT[c] and refined by full matrix least squares against FS2[d] in ShelXL[d,e] using Olex2.[f] All of the non-hydrogen atoms were refined anisotropically while all of the hydrogen atoms were located geometrically and refined using a riding model. The crystal structure, refinement data and references relating to this structure

determination are given below. Crystallographic data has been deposited with the Cambridge Crystallographic Data Centre as supplementary publication **CCDC 2464749**.

a. Bruker, SAINT+ v8.39.0 Integration Engine, Data Reduction Software, Bruker Analytical X-ray Instruments Inc., Madison, WI, USA, **2018**.
b. Bruker, SADABS 2018, Bruker AXS area detector scaling and absorption correction, Bruker Analytical X-ray Instruments Inc., Madison, Wisconsin, USA, **2018**.
c. Sheldrick, G. M. Acta Crystallographica a-Foundation and Advances **2015**, 71, 3–8.
d. Sheldrick, G. M. *Acta Crystallogr., Sect. A: Found. Crystallogr.* **2008**, *64*, 112–122.
e. Sheldrick, G. M. *Acta Crystallogr. C* **2015**, *71*, 3–8.
f. Dolomanov, O. V.; Bourhis, L. J.; Gildea, R. J.; Howard, J. A. K.; Puschmann, H. *J. Appl. Crystallogr.* **2009**, *42*, 339–341.

Figure S1. Crystal structure of *N*-Boc isovarenicline **S18**, with the anisotropic displacement parameters depicted at the 50% probability level and hydrogens omitted for clarity.

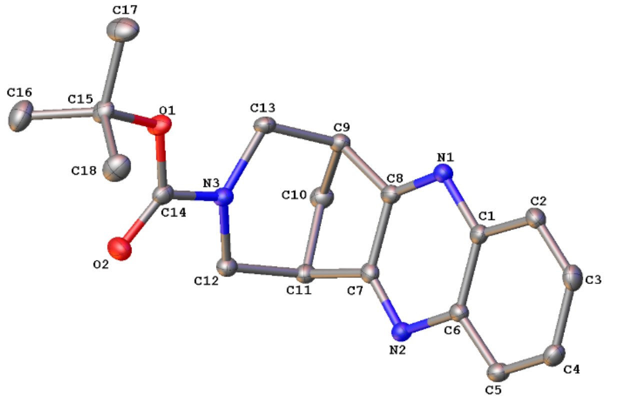

**CCDC number**	**2464749**	μ/mm^{-1}	0.086
Empirical formula	$C_{18}H_{21}N_3O_2$	F(000)	664.0
Formula weight	311.38	Crystal size/mm^3	0.502 × 0.363 × 0.126
Temperature/K	100(2)	Radiation	MoKα (λ = 0.71073)
Crystal system	Monoclinic	2Θ range for data collection/°	2.872 to 56.068
Space group	$P2_1/n$	Index ranges	$-8 \le h \le 8$, $-36 \le k \le 37$, $-11 \le l \le 8$
a/Å	6.7087(3)	Reflections collected	14534
b/Å	28.3664(11)	Independent reflections	3865 [R_{int} = 0.0505, R_{sigma} = 0.0504]
c/Å	8.4356(4)	Data/restraints/parameters	3865/0/211
α/°	90	Goodness-of-fit on F^2	1.027
β/°	93.782(3)	Final R indexes [I>=2σ (I)]	R_1 = 0.0481, wR_2 = 0.1058
γ/°	90	Final R indexes [all data]	R_1 = 0.0744, wR_2 = 0.1174
Volume/Å^3	1601.81(12)	Largest diff. peak/hole / e Å^{-3}	0.28/-0.23
Z	4	μ/mm^{-1}	0.086
ρ_{calc}g/cm^3	1.291		

Isovarenicline HCl salt 5.

To a solution of *N*-Boc isovarenicline **SI8** (180 mg, 0.58 mmol) in MeOH (5 mL) was added HCl (4M in dioxane, 1 mL). After 8 h, the solvents were removed and the solid was triturated with cold EtOAc to give **isovarenicline HCl 5** (140 mg, quantitative) as a light tan solid^{1}H NMR (600 MHz,D_2O): δ 7.98 (m, 2 H, H1), 7.80 (m, 2 H, H2), 3.62 (br d, 2 H, *J* = 12 Hz, H7a), 3.58 (m, 2 H, H6), 3.44 (d, 2 H, *J* = 12 Hz, H7b), 2.60 (m, 1 H, H9a), 2.33 (d, 1 H, *J* = 12 Hz, H9b); ^{13}C NMR (100 MHz, D_2O): δ 158.7, 141.5, 130.8, 128.1, 46.3, 38.2, 36.8. MS (ESI): calculated for $[C_{13}H_{14}N_3]^+$: 212.1188, found $[M]^+$: 212.1190.

Alternative salt preparation

Isovarenicline tartrate salt

Isovarenicline (from N-Bn isovarenicline **SI7** - see** above; 134 mg, 0.64 mmol) in EtOH:acetone (10:3, 1.5 mL) was added to a solution of (*L*)-(+)-tartaric acid (102 mg, 0.68 mmol) in EtOH:acetone (10:3, 1 mL). After 15 min, the mixture was cooled to 0°C and the solid was isolated by filtration, washed with a small quantity of cold 10:3 EtOH:acetone and air-dried to give **isovarenicline tartrate** (174 mg, 76%) as an off-white powder. Spectroscopic properties were identical to the HCl salt with the exception of the signals associated with tartrate, but full details are provided below. ^{1}H NMR (600 MHz, D_2O): δ 7.96 (m, 2 H, H1), 7.80 (m, 2 H, H2), 4.39 (s, 2 H, tartrate), 3.62 (br d, 2 H, *J* = 12 Hz, H7), 3.58 (m, 2 H, H6), 3.44 (d, 2 H, *J* = 12 Hz, H7), 2.60 (m, 1 H, H9), 2.33 (d, 1 H, *J* = 12 Hz, H9); ^{13}C NMR (100 MHz, D_2O): δ 176.3 (C=O, tartrate), 158.7, 141.5, 130.8, 128.1 , 72.8, 46.3, 38.2, 36.8.

3. N_2 Varenicline 6

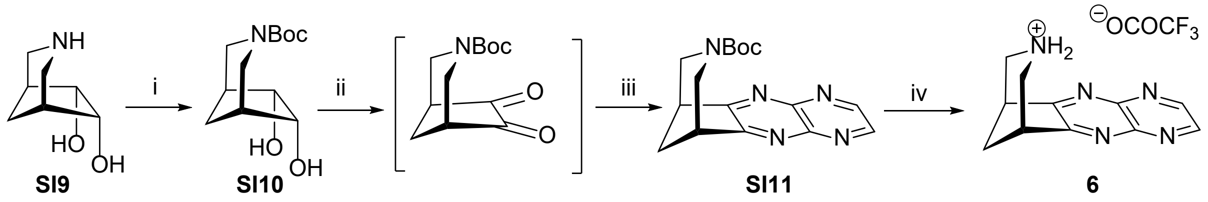

SI Scheme 3. *Reagents*: i, Boc_2O, Na_2CO_3, THF/water, rt, 16h (90%); ii, TFAA, DMSO, CH_2Cl_2, then Et_3N; iii, 2,3-diaminopyrazine, MeOH, 65 °C, 16h (38% over 2 steps); iv, 5% TFA in MeOH (69%).

***tert*-Butyl-6,7-dihydroxy-3-azabicyclo[3.2.1]octane-3-carboxylate SI10**

To a solution of commercially available diol **SI9**·(as HCl salt; also available by hydrogenolysis of **SI6**) (0.89 g, 5.00 mmol) in THF (16 mL) was added di-tert-butyl dicarbonate (1.31 g, 6.00 mmol), followed by a solution of Na_2CO_3 (1.11 g, 10.5 mmol) in water (8 mL). The mixture was stirred 16 h at rt, then diluted with EtOAc (50 mL) and brine (20 mL). The phases were separated, and the aqueous phase was extracted with EtOAc (2 x 50 mL). The combined EtOAc extracts were washed with brine (50 mL), dried (Na_2SO_4), concentrated and the residue was purified by silica gel chromatography (pentane/EtOAc 20:80 to 100% EtOAc) to give **SI10** (1.10 g, 90%) as a colorless solid. ^1H NMR (500 MHz, $CDCl_3$) δ 4.18 – 4.05 (m, 4H), 2.83 (d, *J* = 13.0 Hz, 2H), 2.26 (m, 2H), 1.58 – 1.49 (m, 2H), 1.45 (s, 9H). ^{13}C NMR (126 MHz, $CDCl_3$) δ 156.2, 80.1, 70.3, 37.7, 29.4, 29.4, 28.5. HRMS (ESI) calculated for $C_{12}H_{21}NO_4$, $[M+H]^+$: 244.1543, found: 244.1546.

***N*-Boc N_2 Varenicline SI11**

To a solution of DMSO (0.15 mL) in CH_2Cl_2 (1.2 mL) at -78 °C, was slowly added trifluoroacetic anhydride (0.21 mL, 1.5 mmol), followed by dropwise addition of a solution of diol **SI10** (0.12 g, 0.5 mmol) in CH_2Cl_2 (0.4 mL). The reaction mixture was stirred for 1 h at -78 °C, then triethylamine (0.35 mL, 2.5 mmol) was added. The resulting mixture was stirred 1 h at rt, then water (5 mL) was added and the mixture was extracted with CH_2Cl_2 (2 x 10 mL). The combined extracts were dried (Na_2SO_4) and the crude 1,2-diketone was used immediately in the next step. To a solution of crude diketone (as above) in MeOH (1.0 mL) was added 2,3-diaminopyrazine[5] (55 mg, 0.5 mmol) The resulting heterogeneous mixture was then stirred at 65 °C in a closed Schenck tube for 16 h affording a tan-colored homogeneous solution. The mixture was concentrated and purification by chromatography (BIOTAGE, Sfar Silica HC D 10 g column, CH_2Cl_2/MeOH gradient 2 to 20%) and recrystallization from diethyl ether, afforded ***N*-Boc N_2 varenicline SI11** (60 mg, 38% over 2 steps) as a pale-yellow solid. ^1H NMR (400 MHz, $CDCl_3$) δ 9.04 (s, 2H), 4.36 – 4.19 (m, 2H), 3.66 – 3.48 (m, 2H), 3.49 – 3.35 (m, 2H), 2.61 (m, 1H), 2.20 (d, *J* = 11.7 Hz, 1H), 1.23 (s, 9H). ^{13}C NMR (101 MHz, $CDCl_3$) δ 167.3, 155.5, 147.0,

146.5, 80.5, 49.5, 48.5, 40.2, 38.0, 28.2. HRMS (ESI) calculated for $C_{16}H_{19}N_5O_2$, $[M+Na]^+$: 336.1431, found: 336.1428.

N_2 Varenicline trifluoroacetate 6

N-Boc N_2 varenicline **SI11** (40 mg, 0.13 mmol) was dissolved in a solution of trifluoroacetic acid (5% v/v in dichloromethane, 4 mL) and allowed to stand for 16 h at rt. The solvent was removed and toluene was added to facilitate removal of residual TFA under vacuum. The resulting brown oil was triturated with acetone affording a crystalline yellow solid, from which the solvent was decanted, and the solids were washed again with acetone, acetone decanted and the solid was dried under vacuum to give **N_2 varenicline trifluoroacetate 6** (30 mg, 69%) as a yellow crystalline solid. . ^1H NMR (500 MHz, DMSO) δ 9.22 (s, 1H), 9.19 (s, 2H), 8.61 (s, 1H), 3.65 (dd, *J* = 4.9, 2.3 Hz, 2H), 3.62 (d, *J* = 11.0 Hz, 2H), 3.42 (d, *J* = 11.0 Hz, 2H), 2.63 (m, 1H), 2.42 (d, *J* = 11.7 Hz, 1H). ^{13}C NMR (126 MHz, DMSO) δ 164.4, 158.3 (q, *J* = 35.8 Hz), 147.7, 146.4, 115.8 (q, *J* = 292.4 Hz), 45.9, 38.3, 36.5. ^{19}F NMR (376 MHz, DMSO-*D*$_6$) δ -74.57. HRMS (ESI) calculated for $C_{11}H_{12}N_5$, $[M+H]^+$: 214.1087, found: 214.1089.

We also evaluated an alternative approach to N_2 varenicline **6** based on use of the N-benzyl intermediate by analogy to the chemistry used for **4** and **5**.

N-Benzyl N_2 varenicline SI12

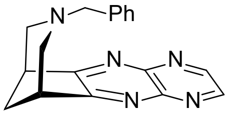

SI12

To mixture of DCC (1.77 g, 8.57 mmol) in DMSO (10 mL) a solution of benzyl-3-azabicyclo[3.2.1]octane-6,7-diol **SI6** (250 mg, 1.07 mmol) in DMSO (2 mL) was added followed by dichloroacetic acid (221 mg, 140 μL, 1.72 mmol). The reaction mixture was stirred at rt for 20h, then filtered through celite which was washed with ethyl acetate (50 mL). EtOAc was removed and to the orange residue was added 2,3-diaminopyrazine (118 mg, 1.072 mmol) and the mixture was stirred at 65 °C for 3h. Water (150 mL) was added, and aqueous phase was extracted with ethyl acetate (3 x 50mL). The combined extracts were washed with water (150 mL) and brine (150 mL), concentrated, and the orange residue was purified by silica

chromatography (eluent 1% MeOH → 2.5% MeOH in DCM) to give **N-benzyl N_2 Varenicline SI12** (130 mg, 40%) as a pale-yellow solid. ^{1}H NMR (400 MHz, $CDCl_3$): δ 8.94 (s, 2H), 7.05 – 7.00 (m, 3H), 6.71 – 6.69 (m, 2H), 3.42 (bt, *J* = 4 Hz, 2H), 3.38 (s, 2H), 3.23 – 3.19 (m, 2H), 2.68 (d, *J* = 8 Hz, 2H), 2.44 (m, 1H), 2.02 (d, *J* = 12 Hz, 1H); ^{13}C NMR (100 MHz, $CDCl_3$): δ 169.3, 146.4, 146.1., 137.1, 128.4, 128.3, 127.1, 61.8, 57.4, 41.3, 39.5; Calculated for $[C_{18}H_{18}N_5]^+$: 304.1562, found $[M+H]^+$: 304.1560.

N-Benzyl N_2 varenicline **SI12** was of very limited synthetic utility because under a variety of conditions (essentially those approaches that had worked for debenzylation of **SI2** and **SI7**), we were unable to deprotect successfully **SI12**. We only observed substrate decomposition but ^{1}H NMR analysis of the crude product indicated heteroarene reduction/fragmentation had probably occurred.

***(iii)* Data S1: ¹H and ¹³C NMR spectra of key intermediates and final products.**

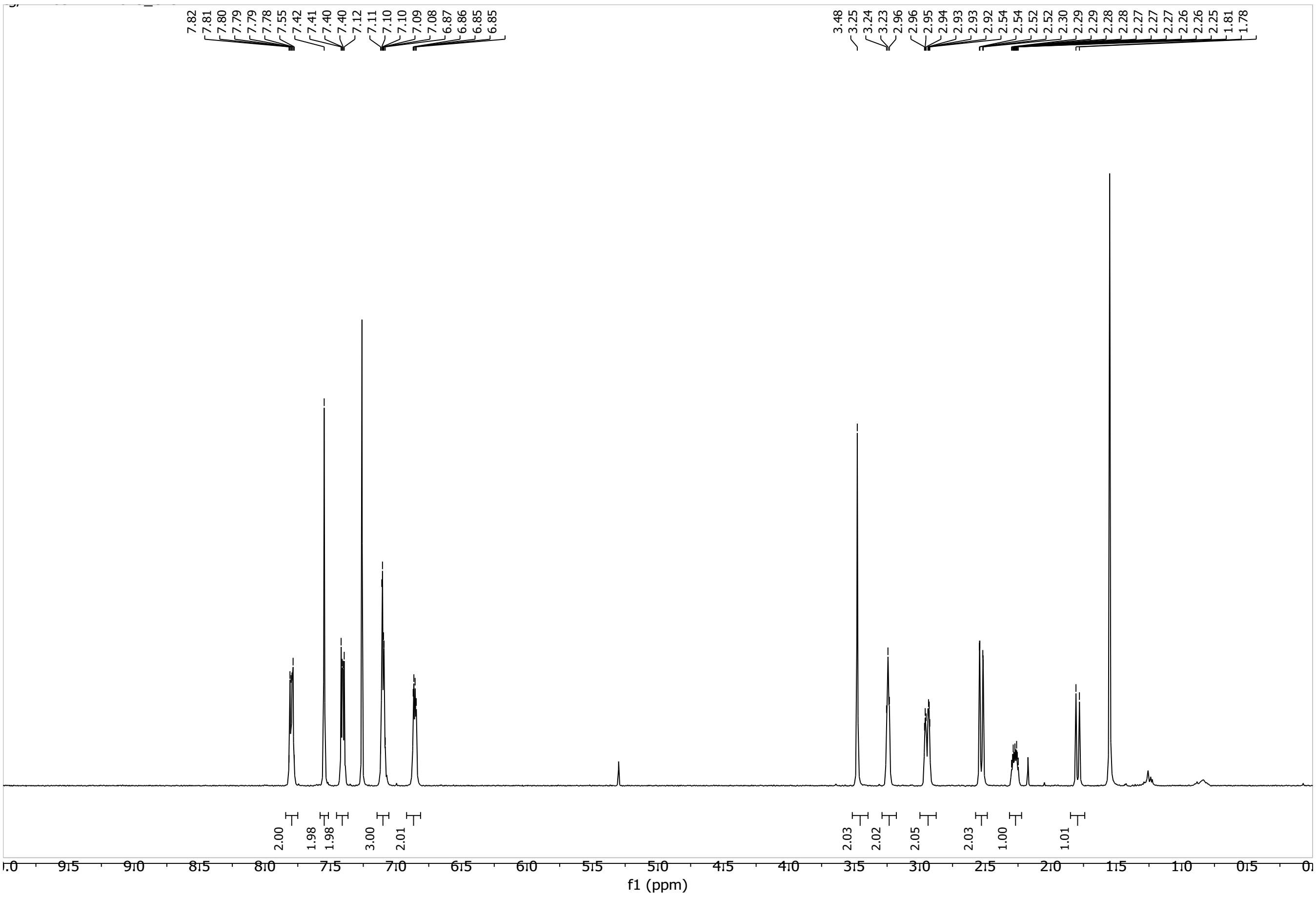

¹H NMR of *N*-Bn C_2 varenicline **SI2**

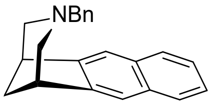

SI2

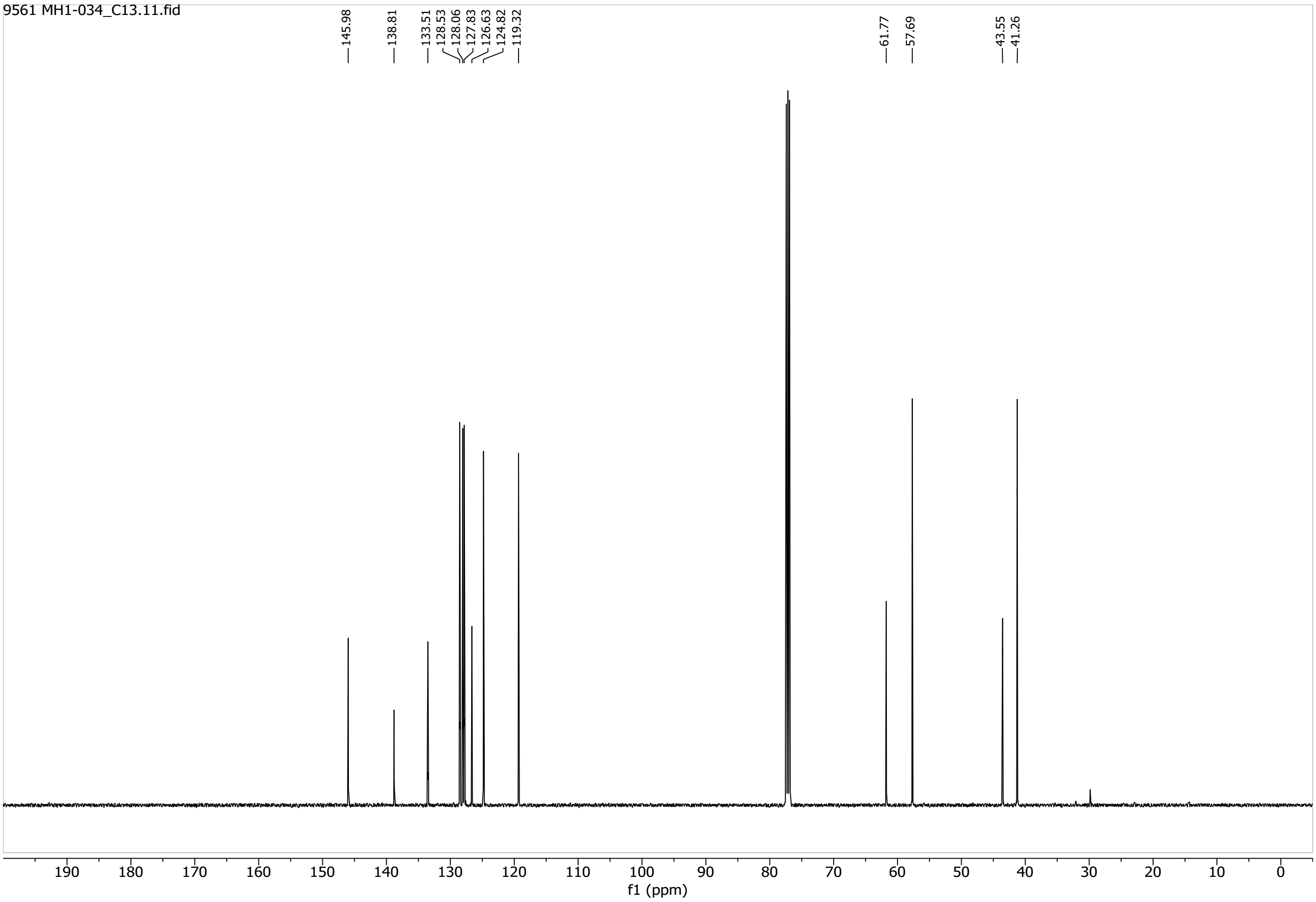

^{13}C NMR of *N*-Bn C_2 varenicline **SI2**

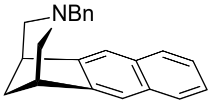

SI2

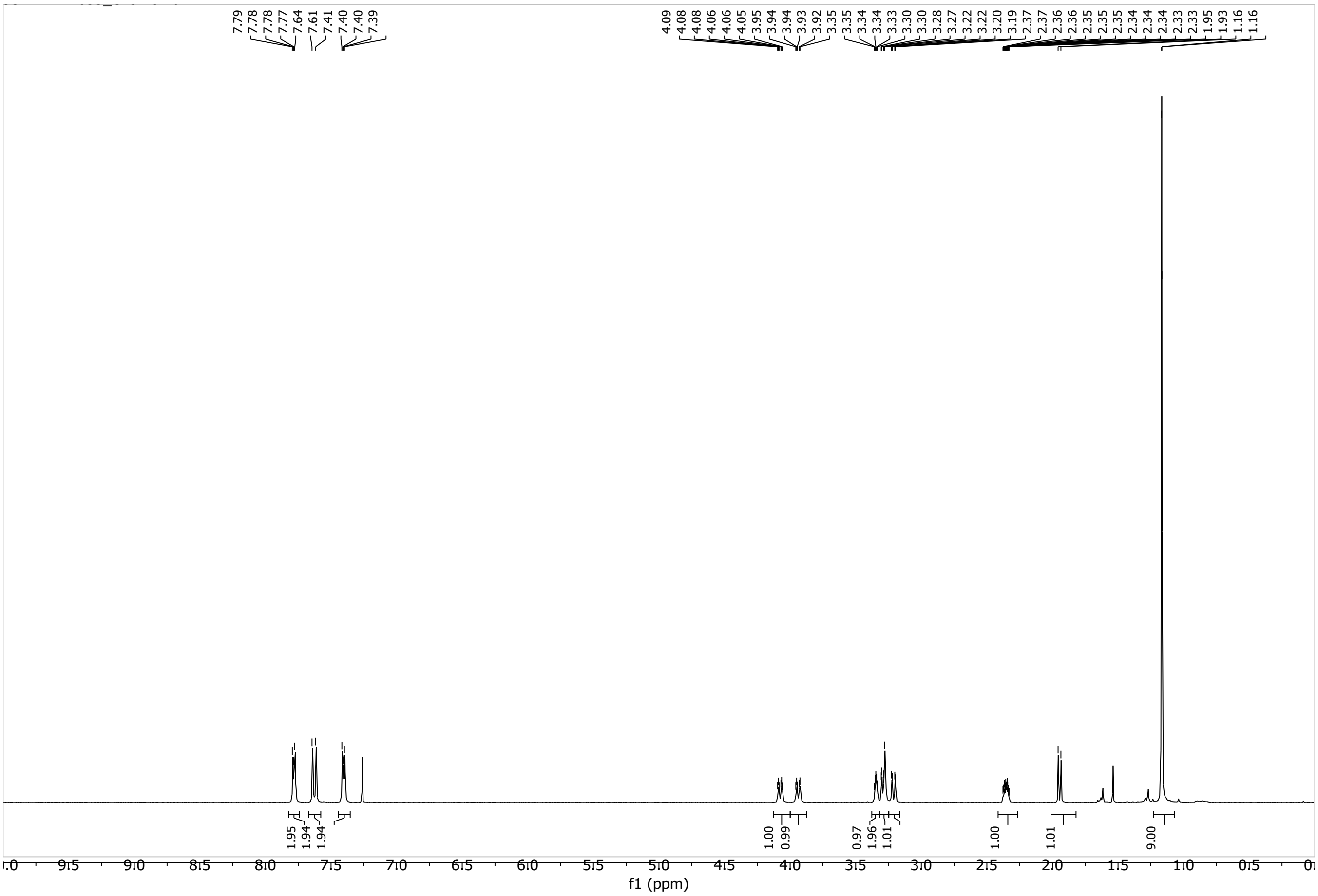

^{1}H NMR of *N*-Boc C_2 varenicline **SI3**

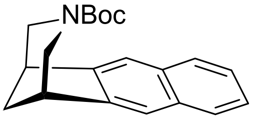

SI3

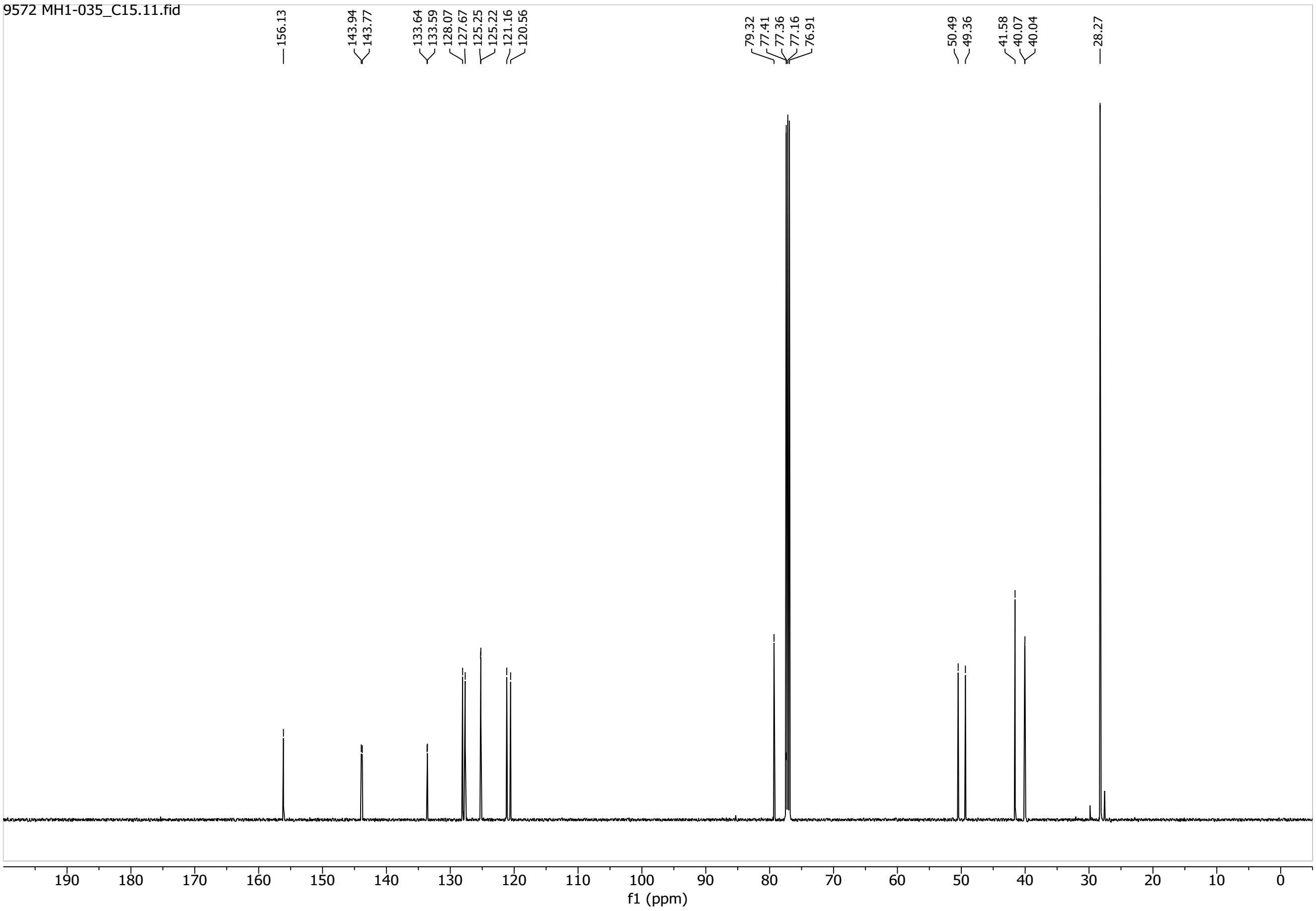

^{13}C NMR of *N*-Boc C_2 varenicline **SI3**

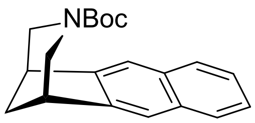

SI3

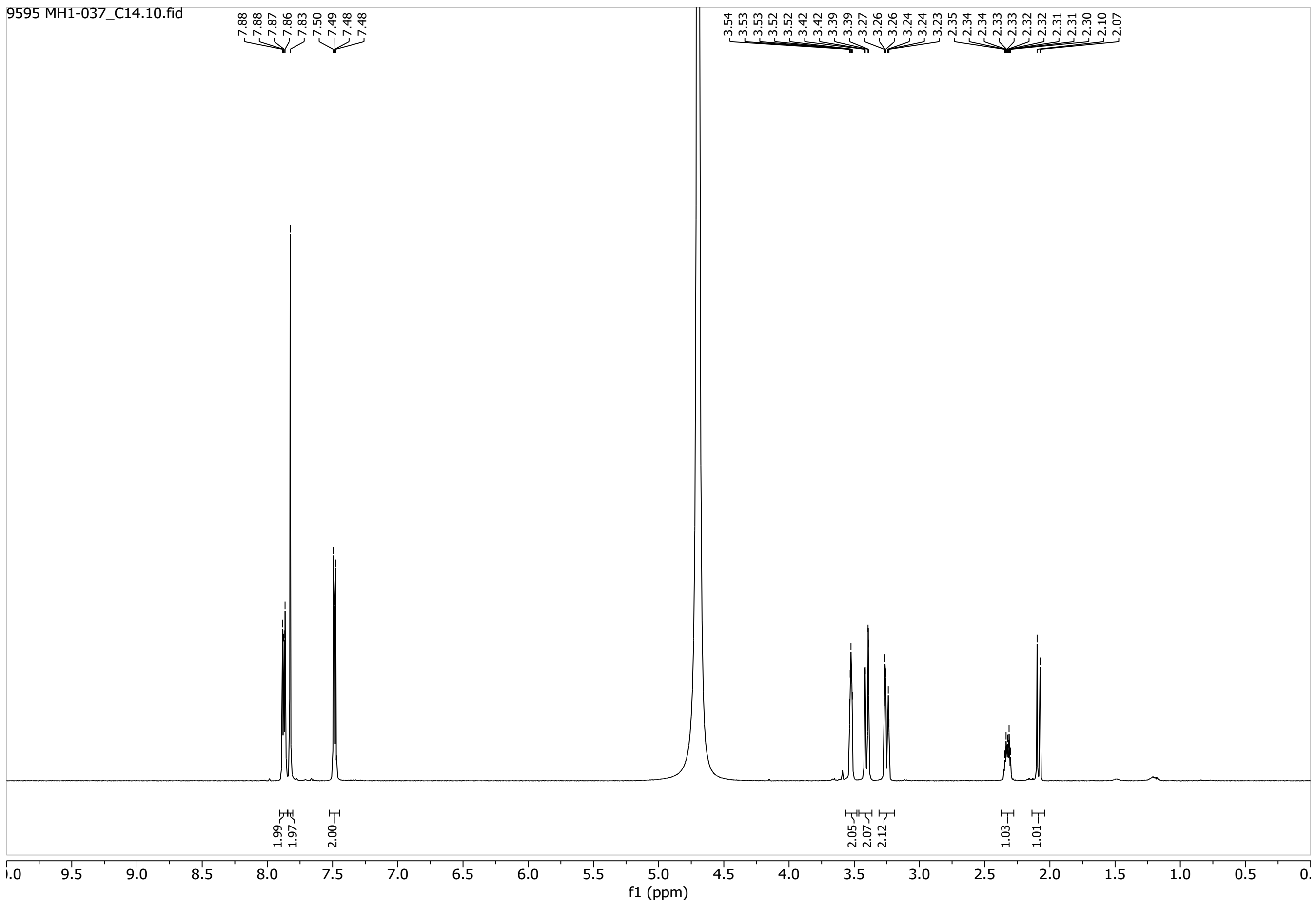

^{1}H NMR C_2 varenicline.HCl **4**

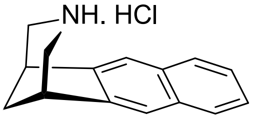

4

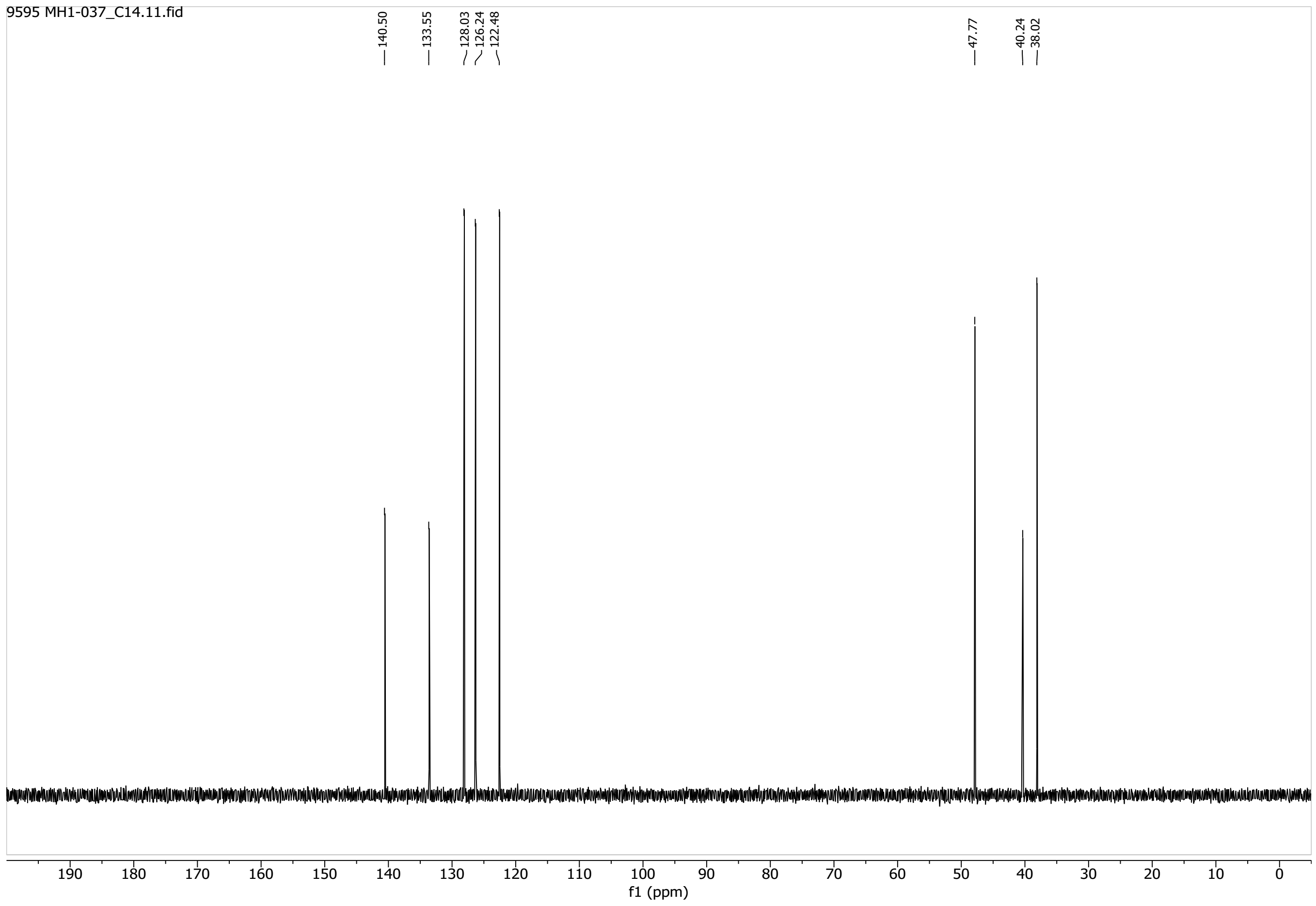

^{13}C NMR C_2 varenicline.HCl **4**

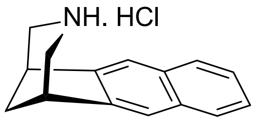

4

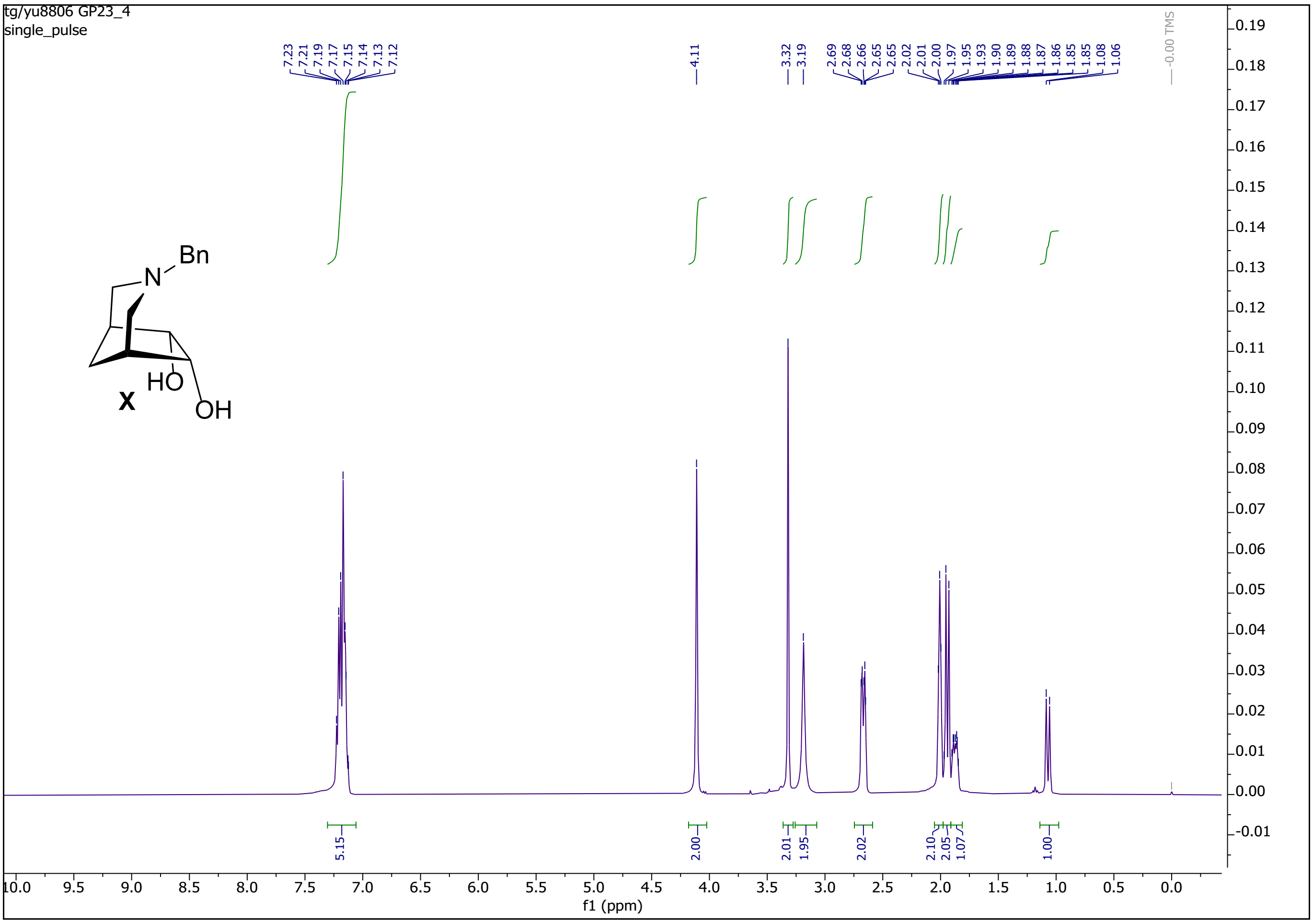

^{1}H NMR of *N*-Bn diol **SI6**

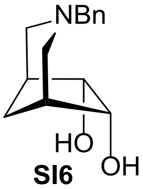

SI6

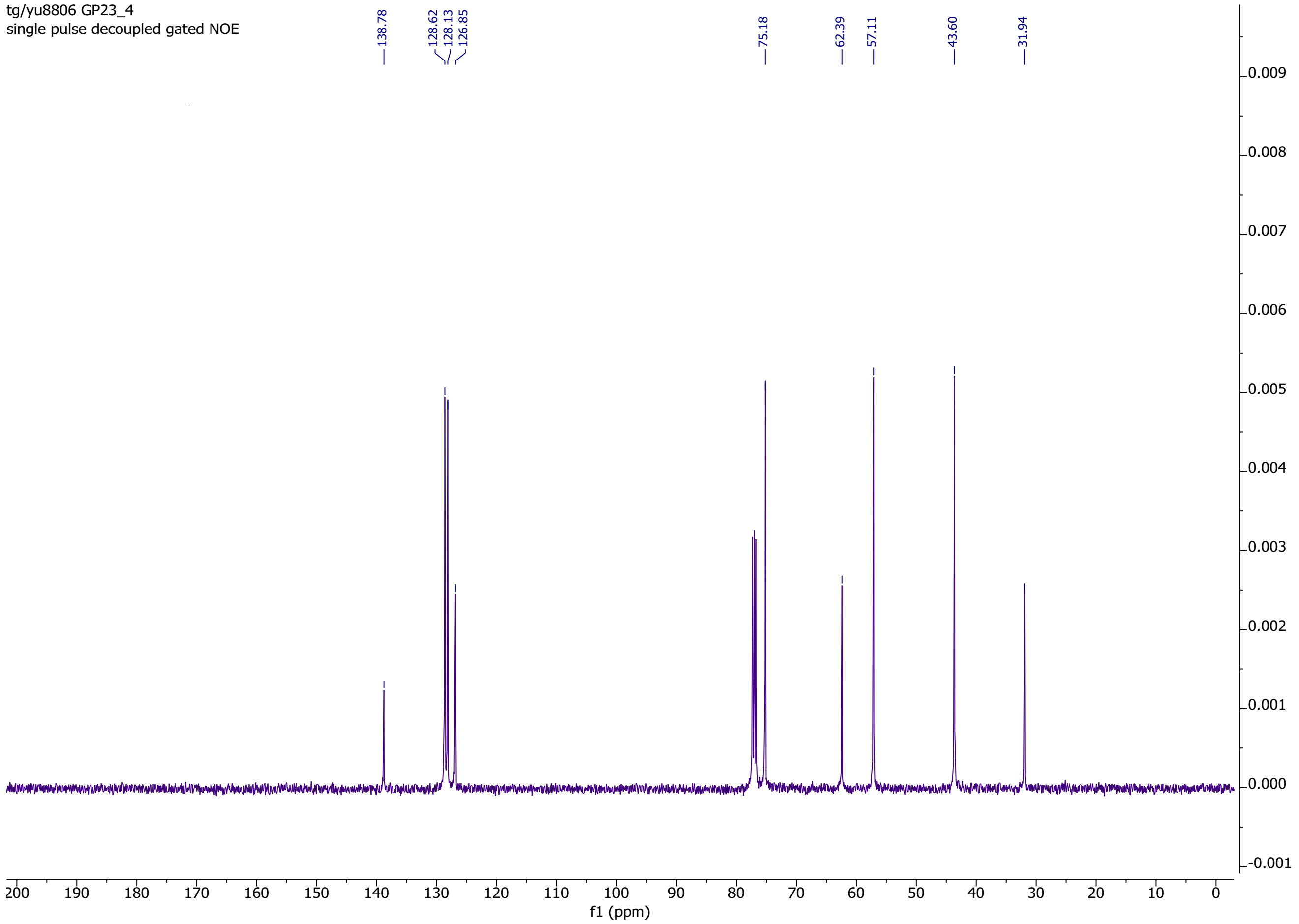

^{13}C NMR of *N*-Bn diol **SI6**

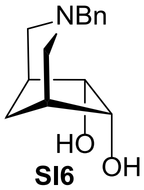

SI6

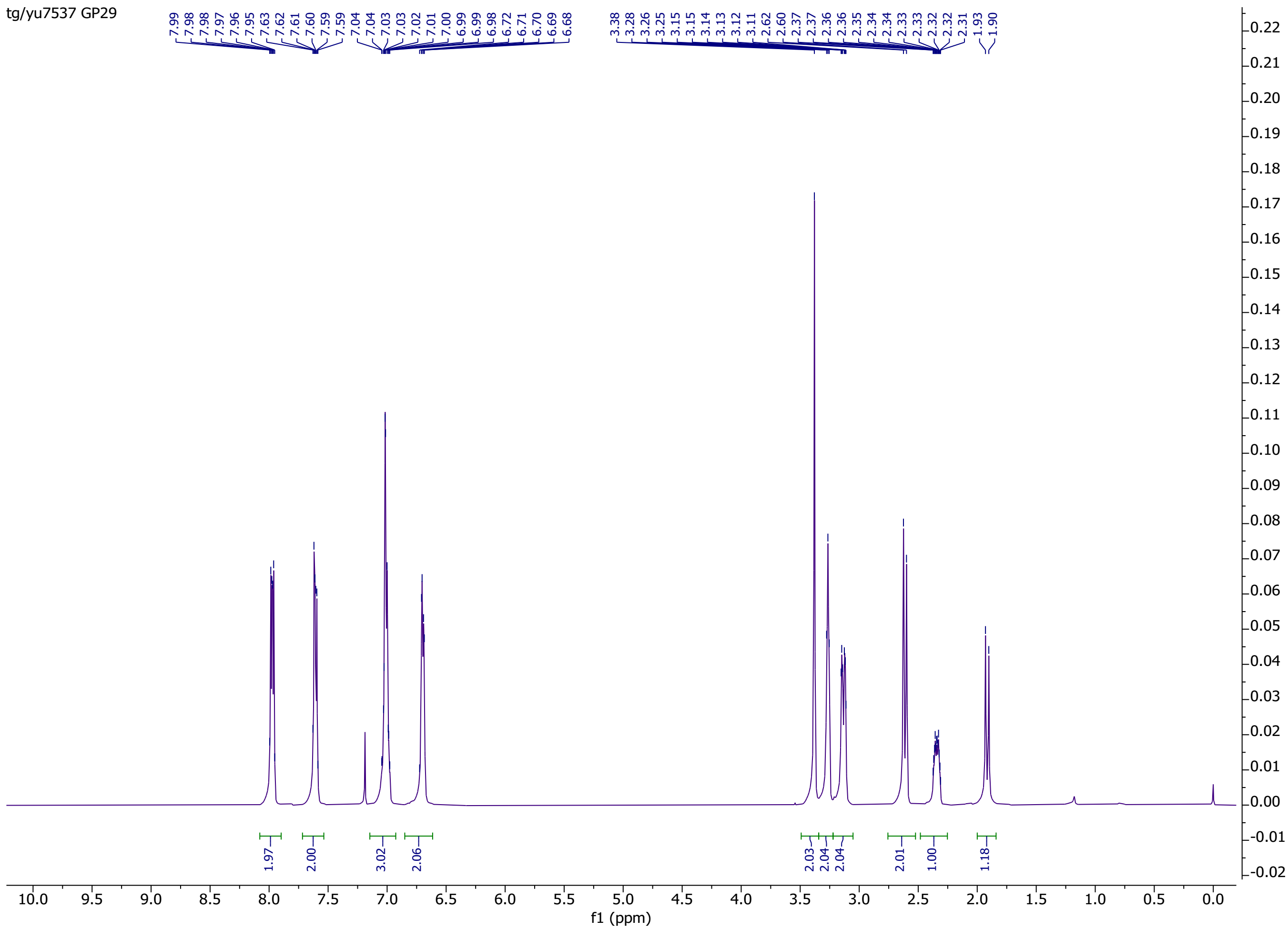

^{1}H NMR of *N*-Bn isovarenicline **SI7**

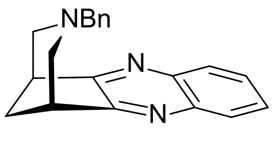

SI7

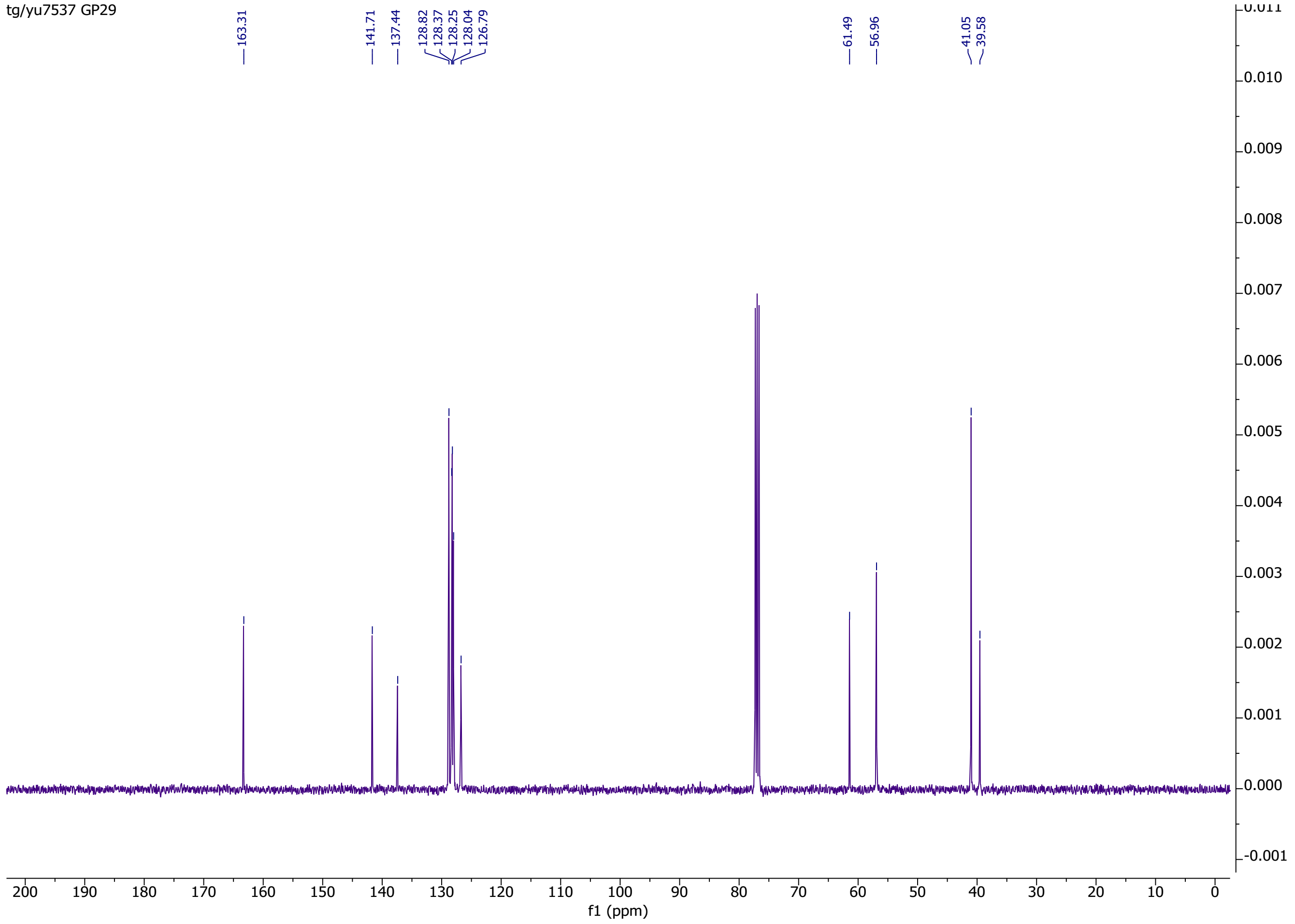

^{13}C NMR of *N*-Bn isovarenicline **SI7**

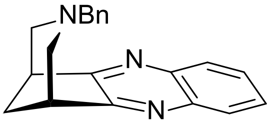

SI7

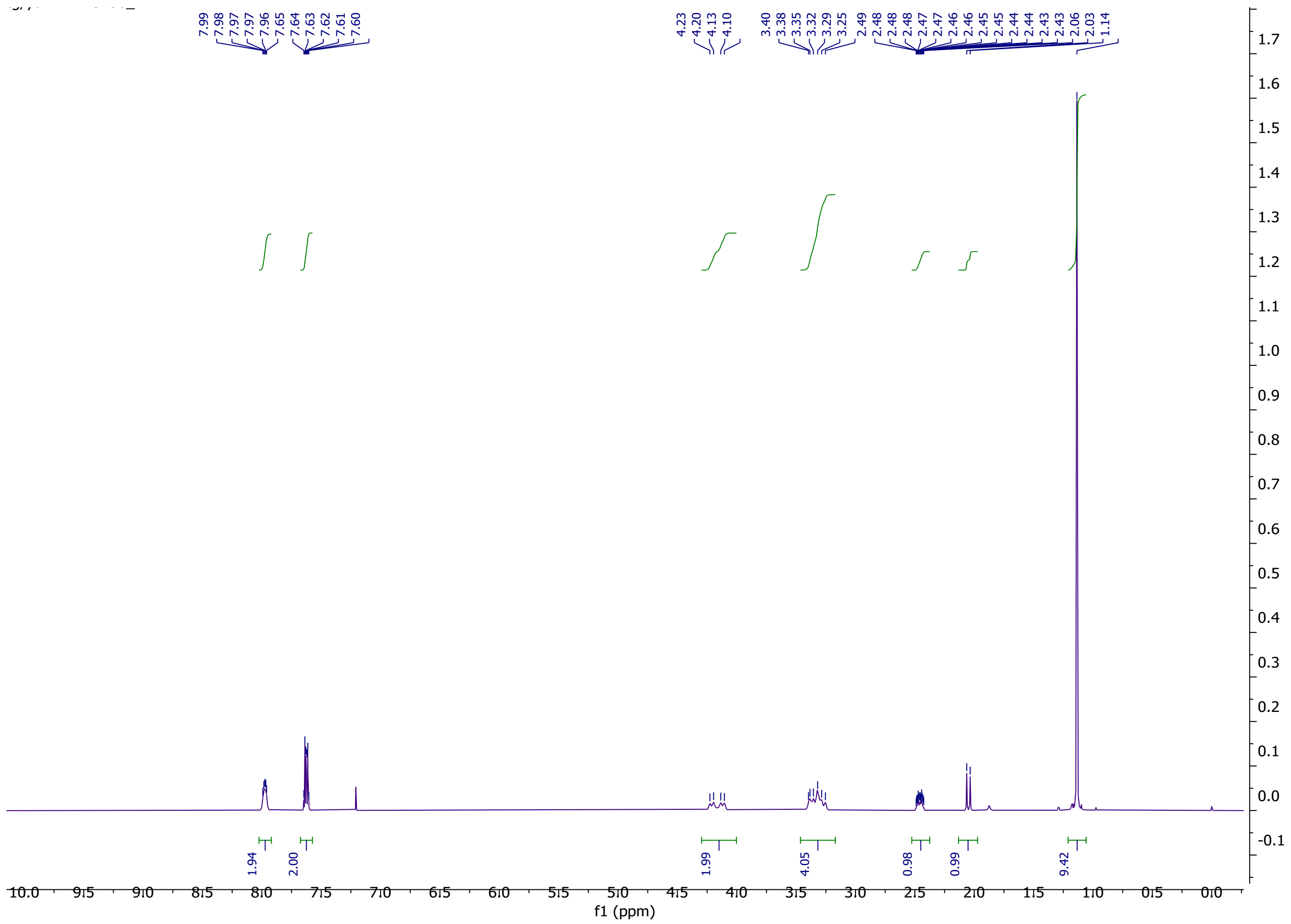

^{1}H NMR of *N*-Boc isovarenicline **SI8**

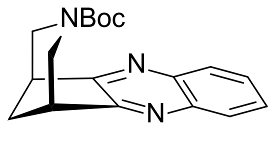

SI8

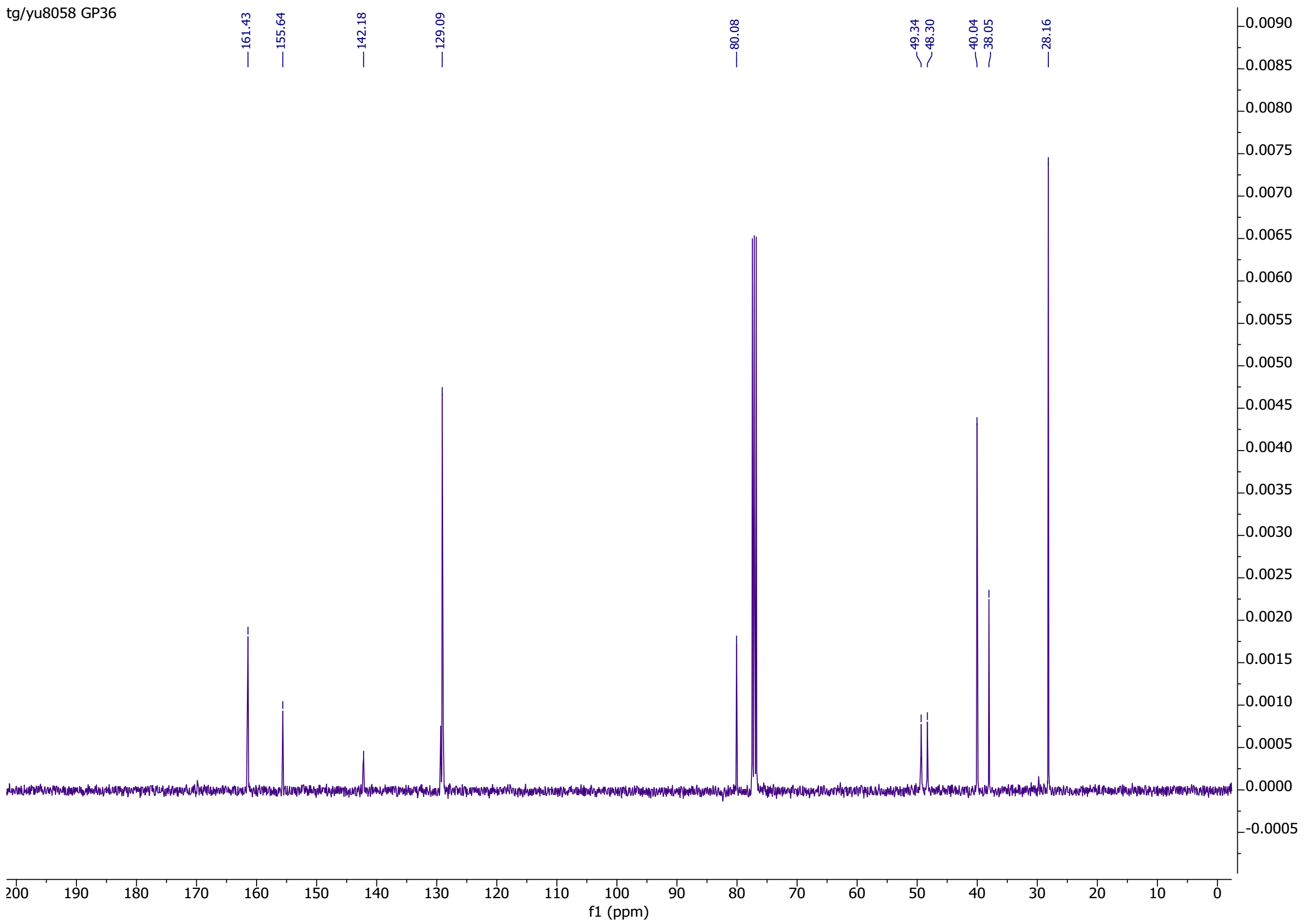

^{13}C NMR of *N*-Boc isovarenicline **SI8**

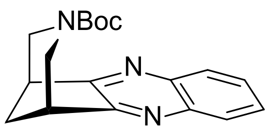

SI8

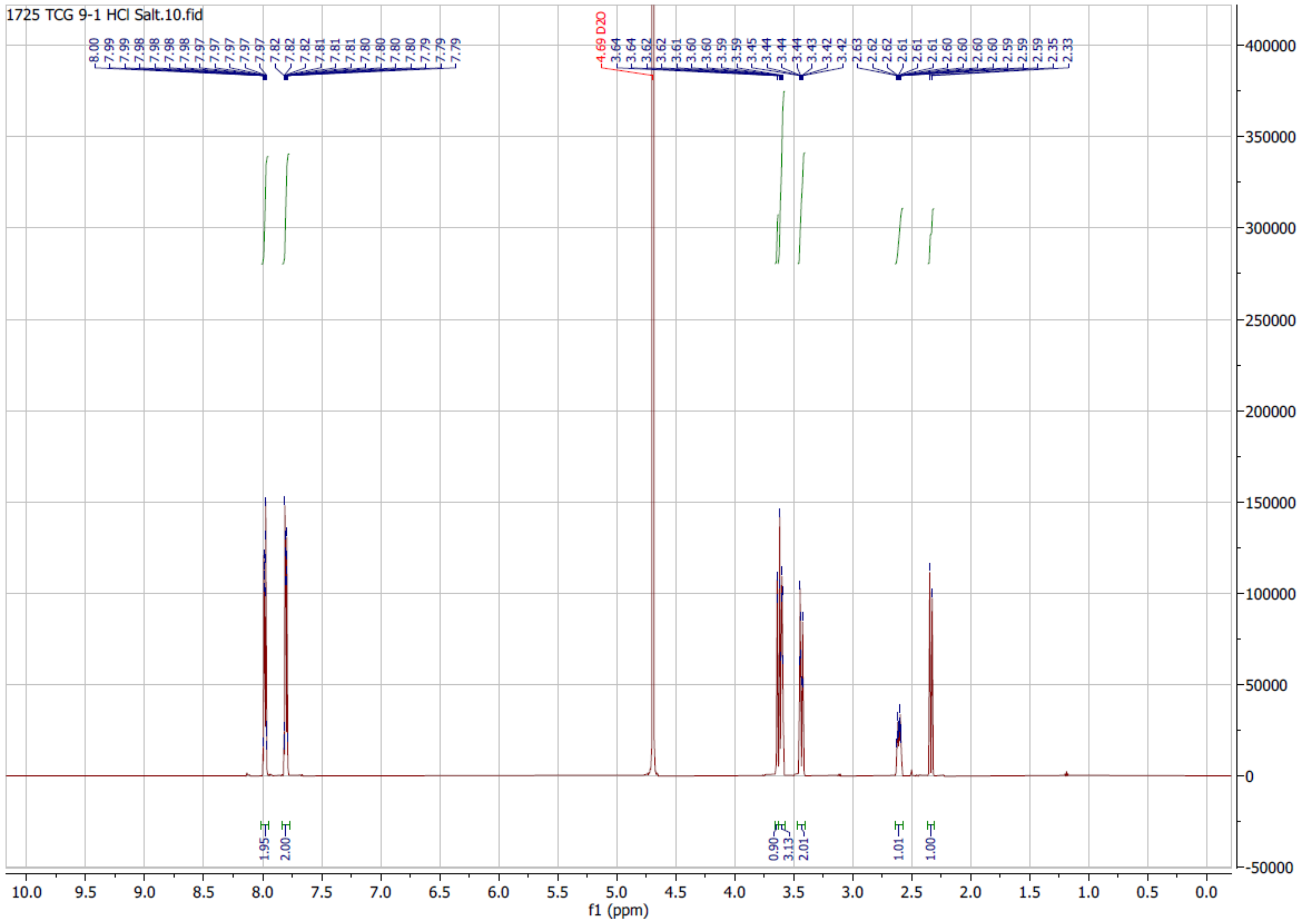
1725 TCG 9-1 HCl Salt.10.fid
8.00 7.99 7.99 7.98 7.98 7.98 7.98 7.97 7.97 7.97 7.97 7.82 7.82 7.82 7.81 7.81 7.81 7.80 7.80 7.80 7.79 7.79
4.69 D2O
3.64 3.64 3.62 3.62 3.61 3.60 3.60 3.59 3.59 3.45 3.44 3.44 3.44 3.43 3.42 3.42 2.63 2.62 2.62 2.61 2.61 2.61 2.60 2.60 2.60 2.60 2.59 2.59 2.59 2.35 2.33
1.95
2.00
0.90
3.13
2.01
1.01
1.00
f1 (ppm)
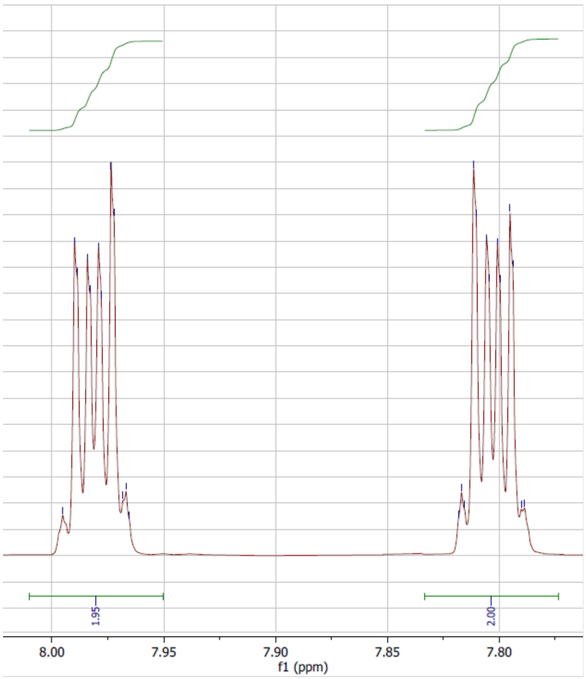
1.95
2.00
f1 (ppm)

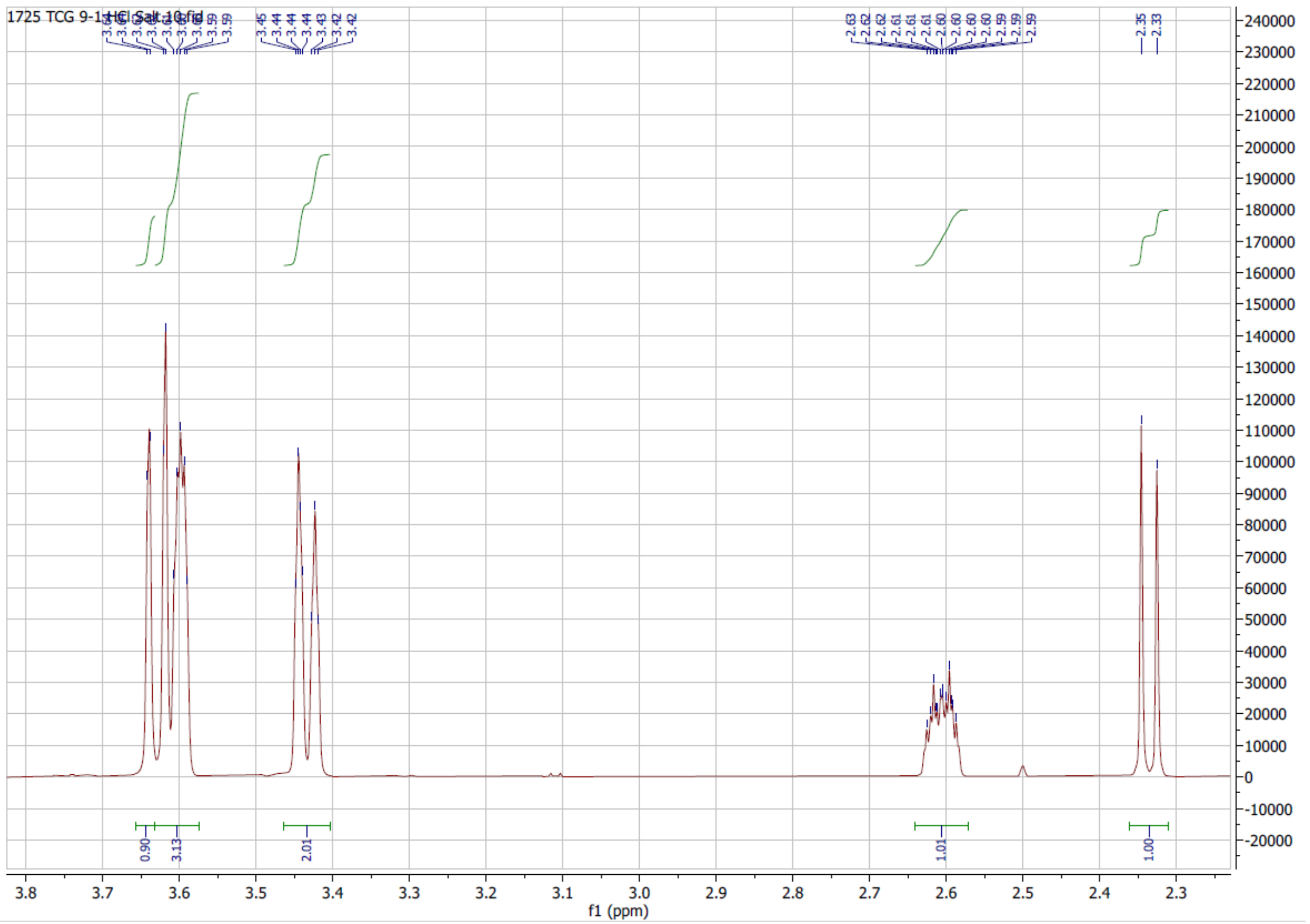

^{1}H NMR of isovarenicline HCl **5**

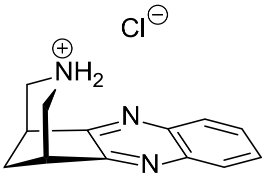

5

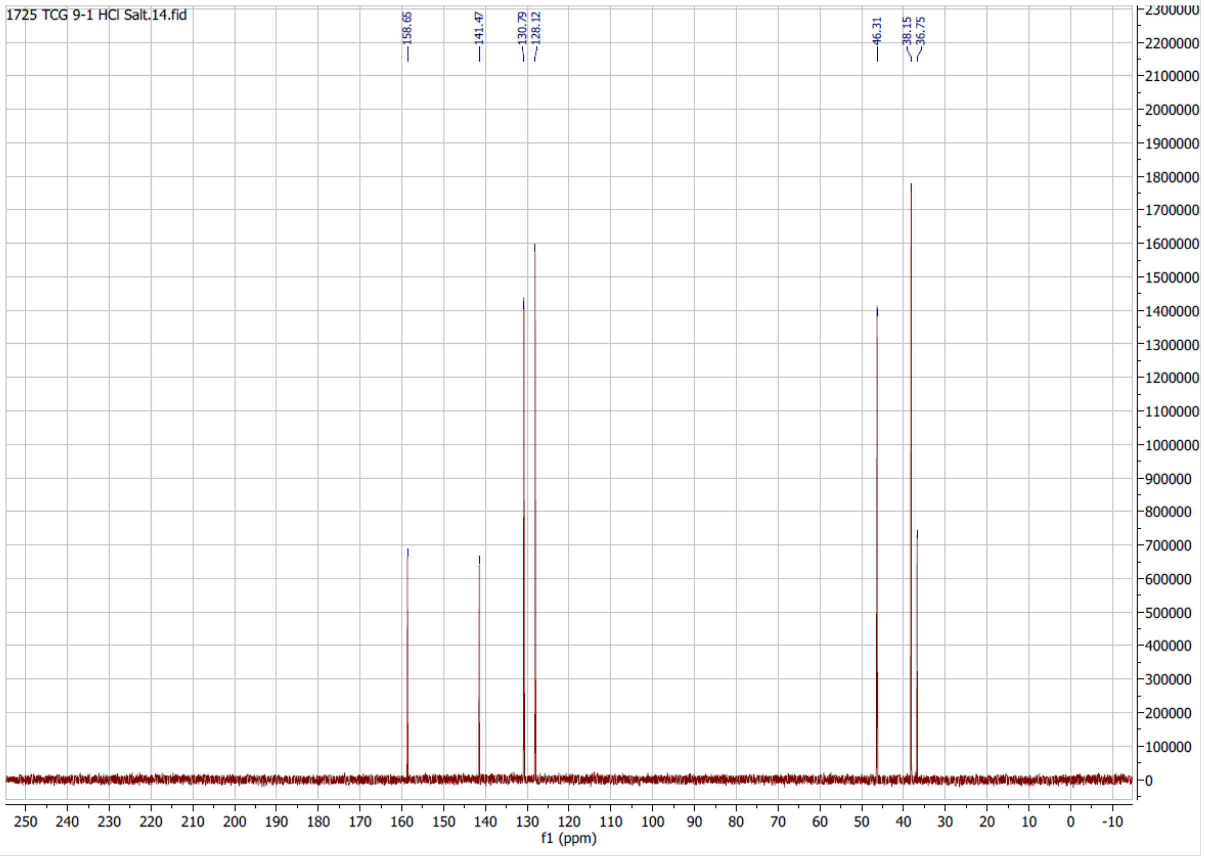

^{13}C NMR of isovarenicline HCl **5**

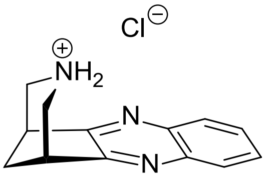

5

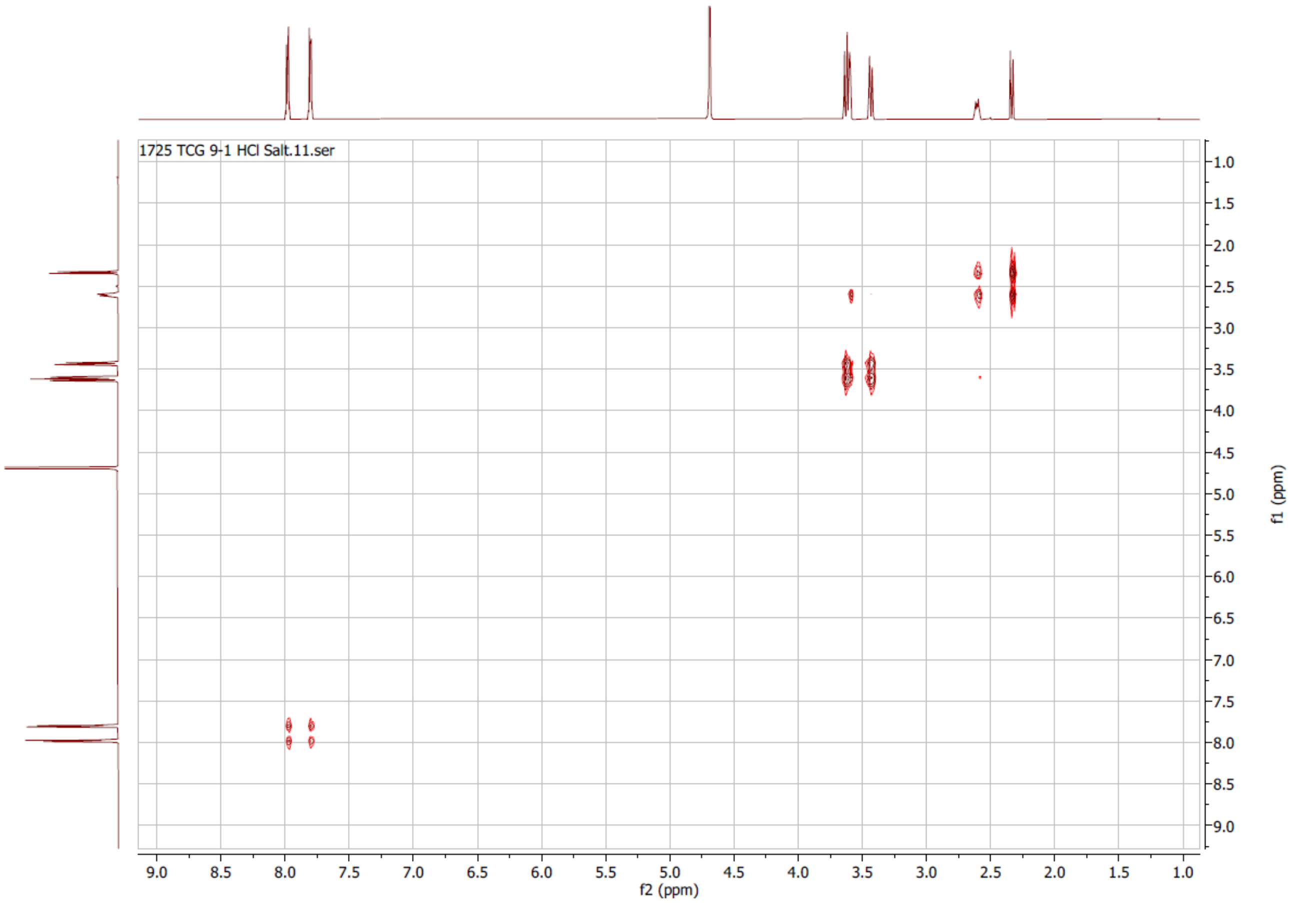
1725 TCG 9-1 HCl Salt.11.ser
f2 (ppm)
f1 (ppm)
9.0
8.5
8.0
7.5
7.0
6.5
6.0
5.5
5.0
4.5
4.0
3.5
3.0
2.5
2.0
1.5
1.0

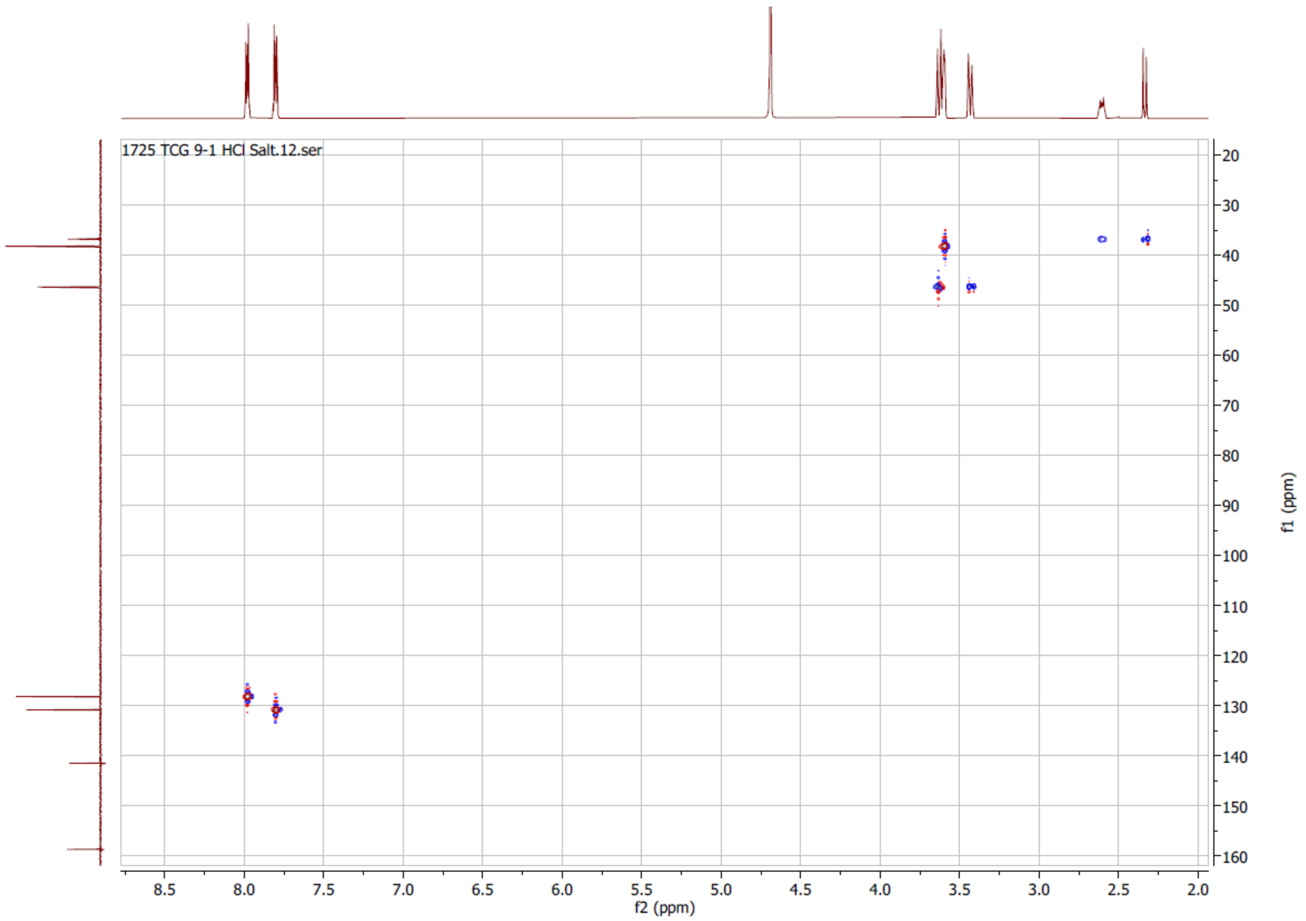
1725 TCG 9-1 HCl Salt.12.ser
f2 (ppm)
f1 (ppm)

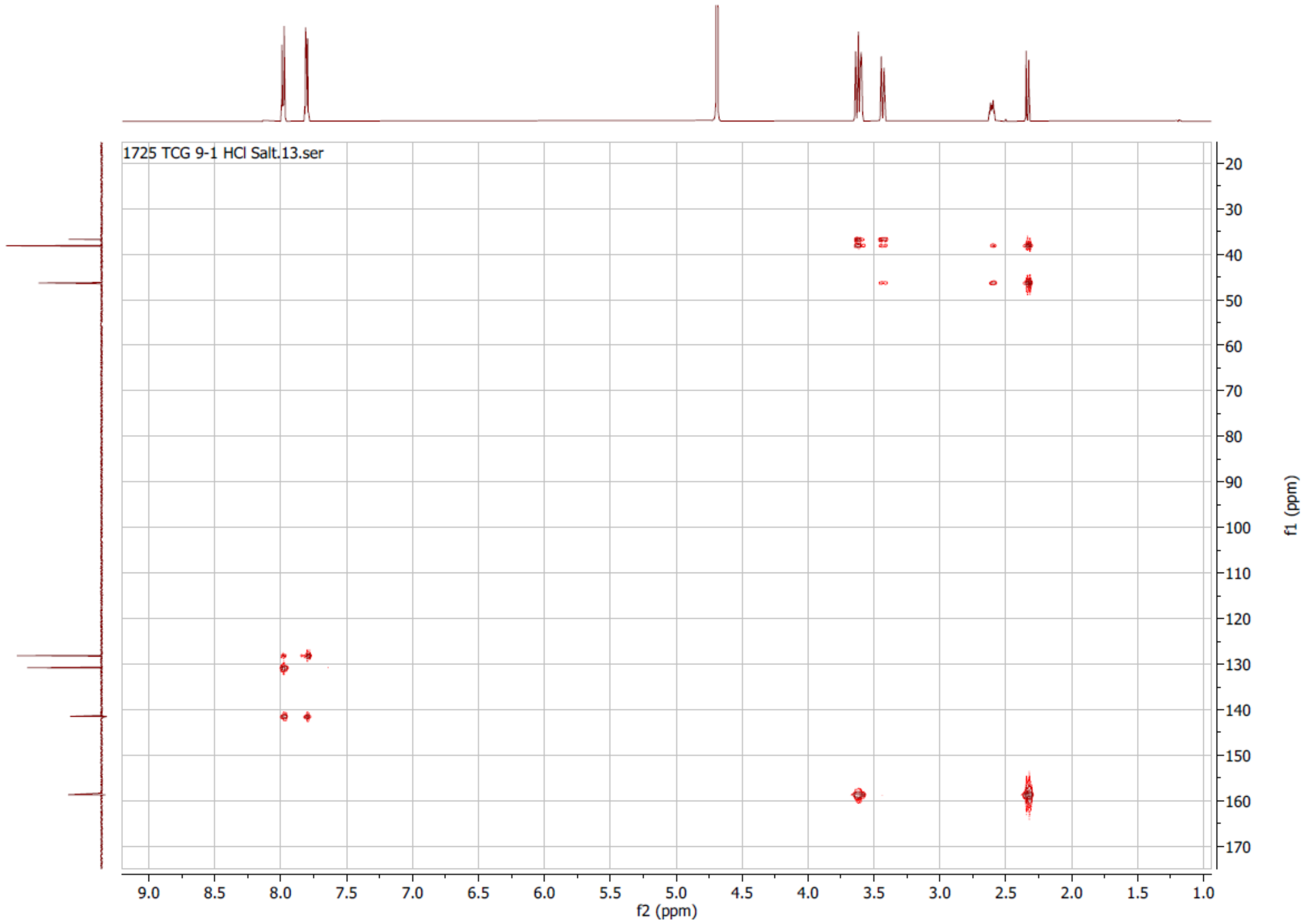

2D COSY. HSQC and HMBC spectra of isovarenicline HCl **5**

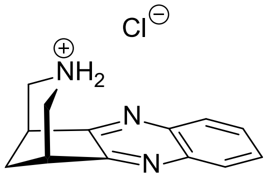

5

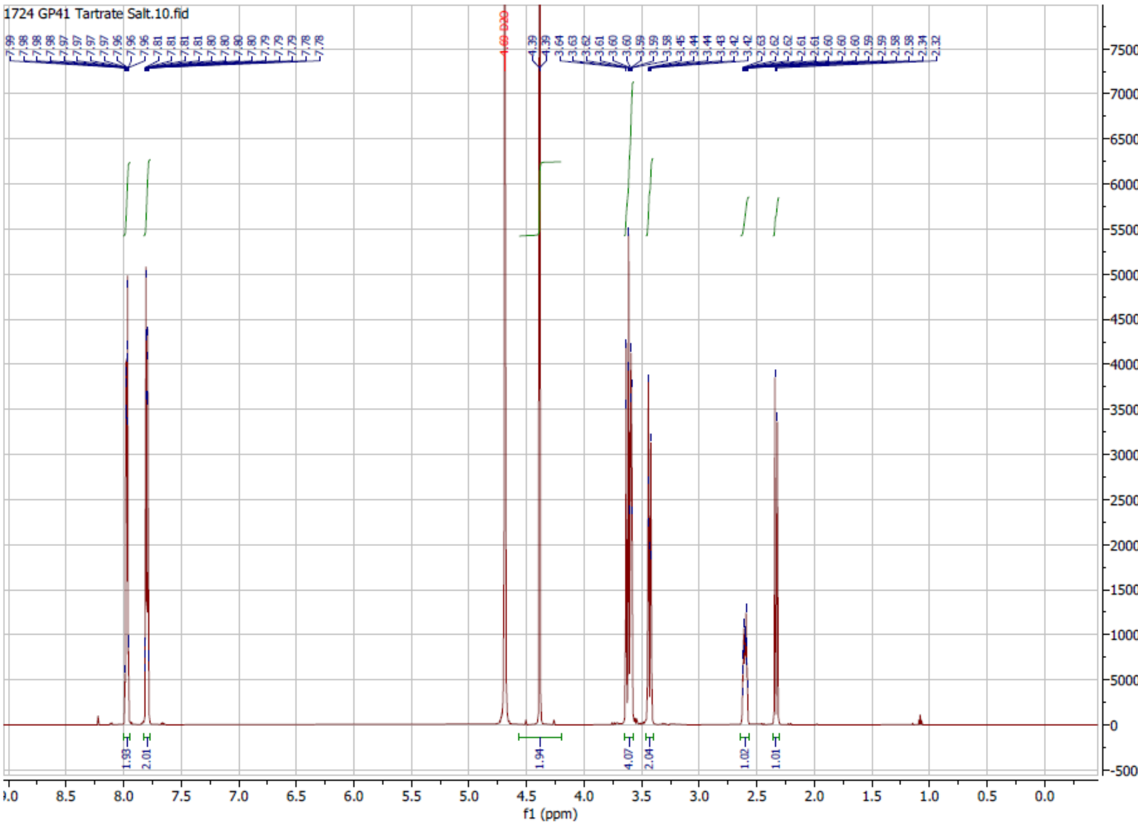

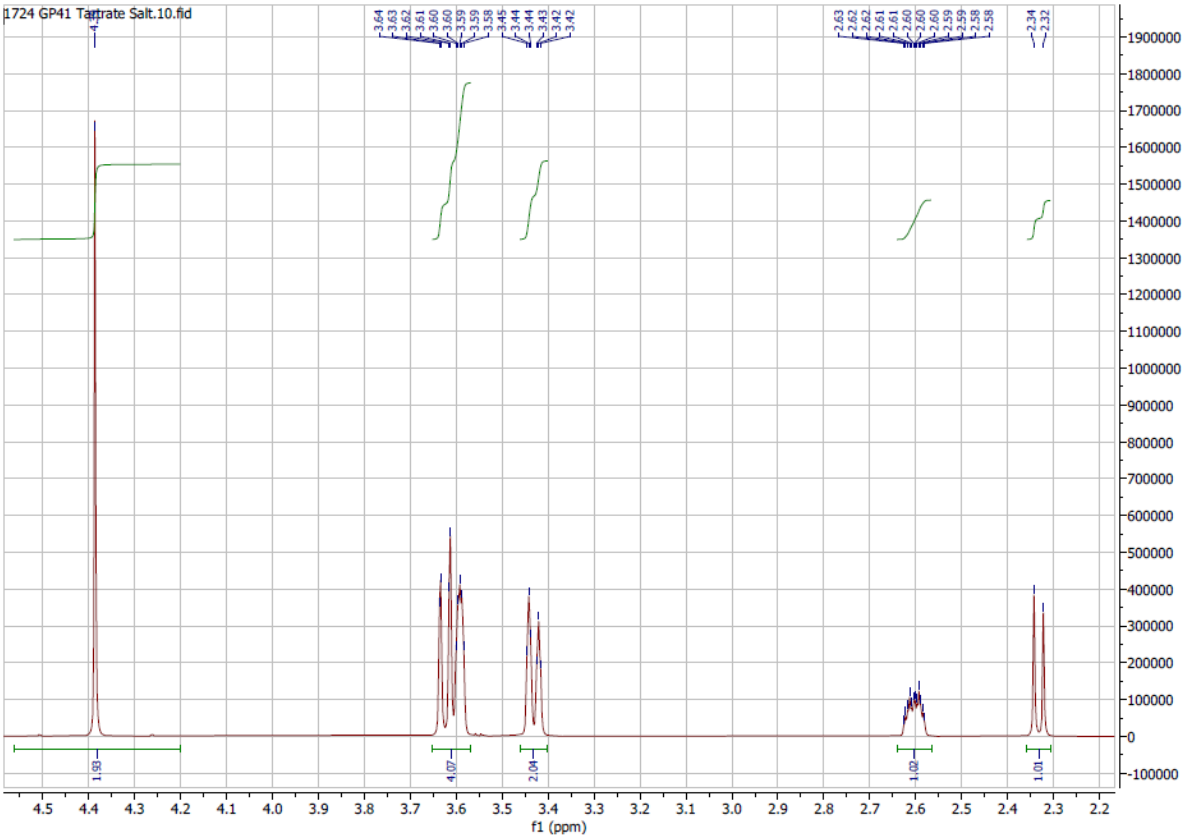

^{1}H NMR of isovarenicline tartrate

16015 df-tg-02-fullanalysis.10.fid

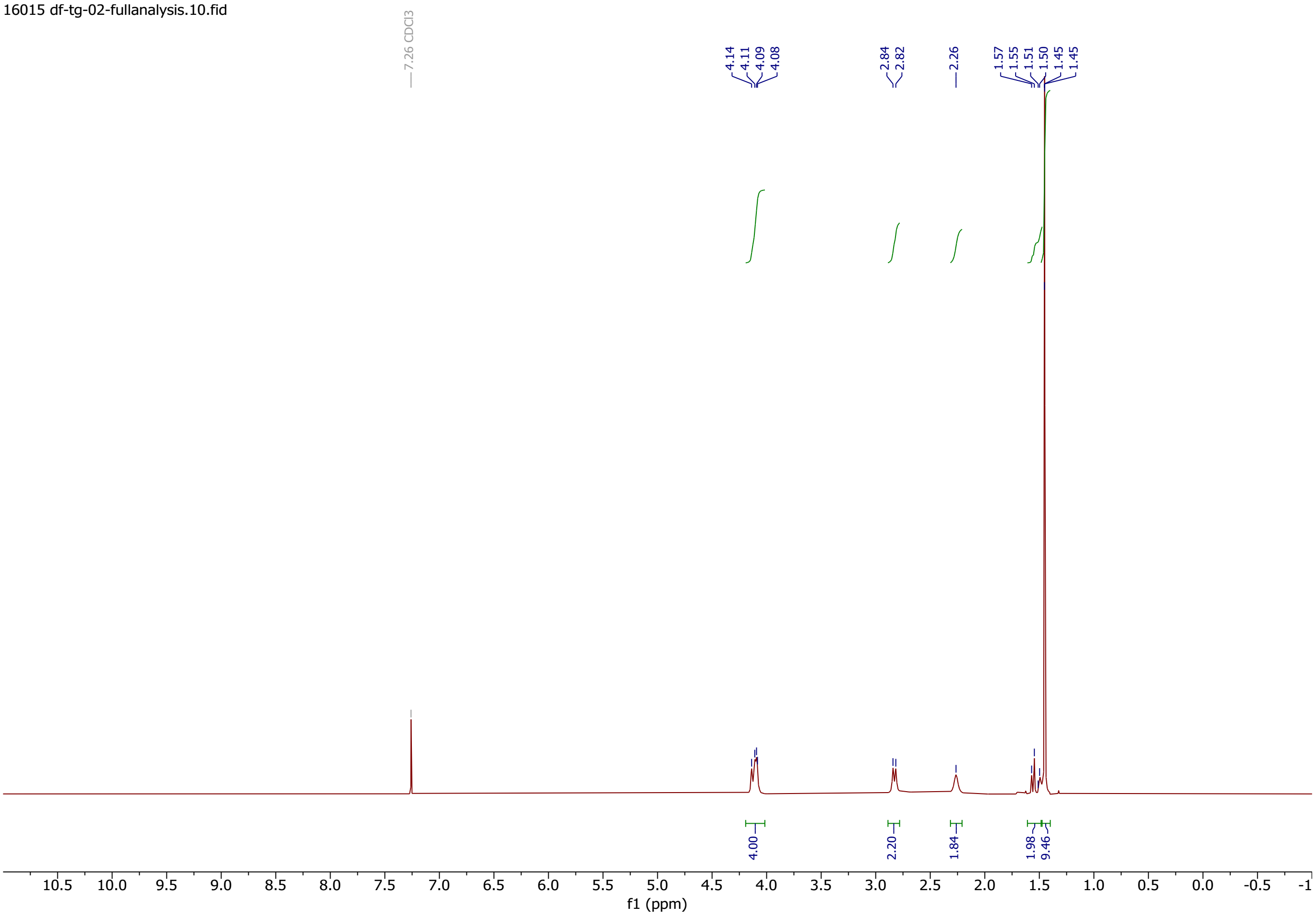

^{1}H NMR of N-Boc diol **SI10**

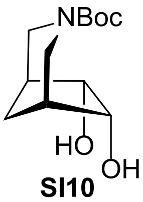

SI10

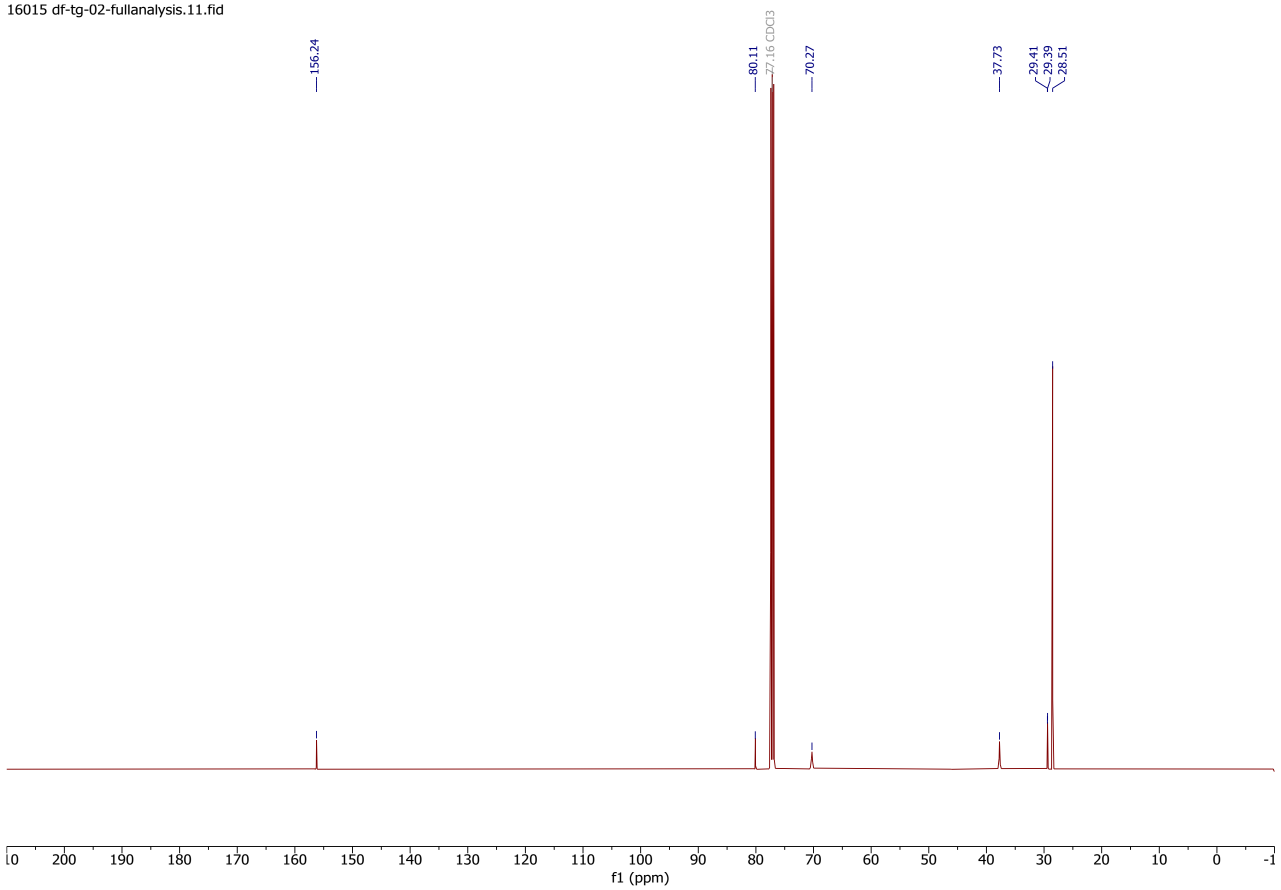

^{13}C NMR of N-Boc diol **SI10**

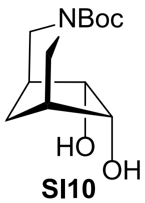

SI10

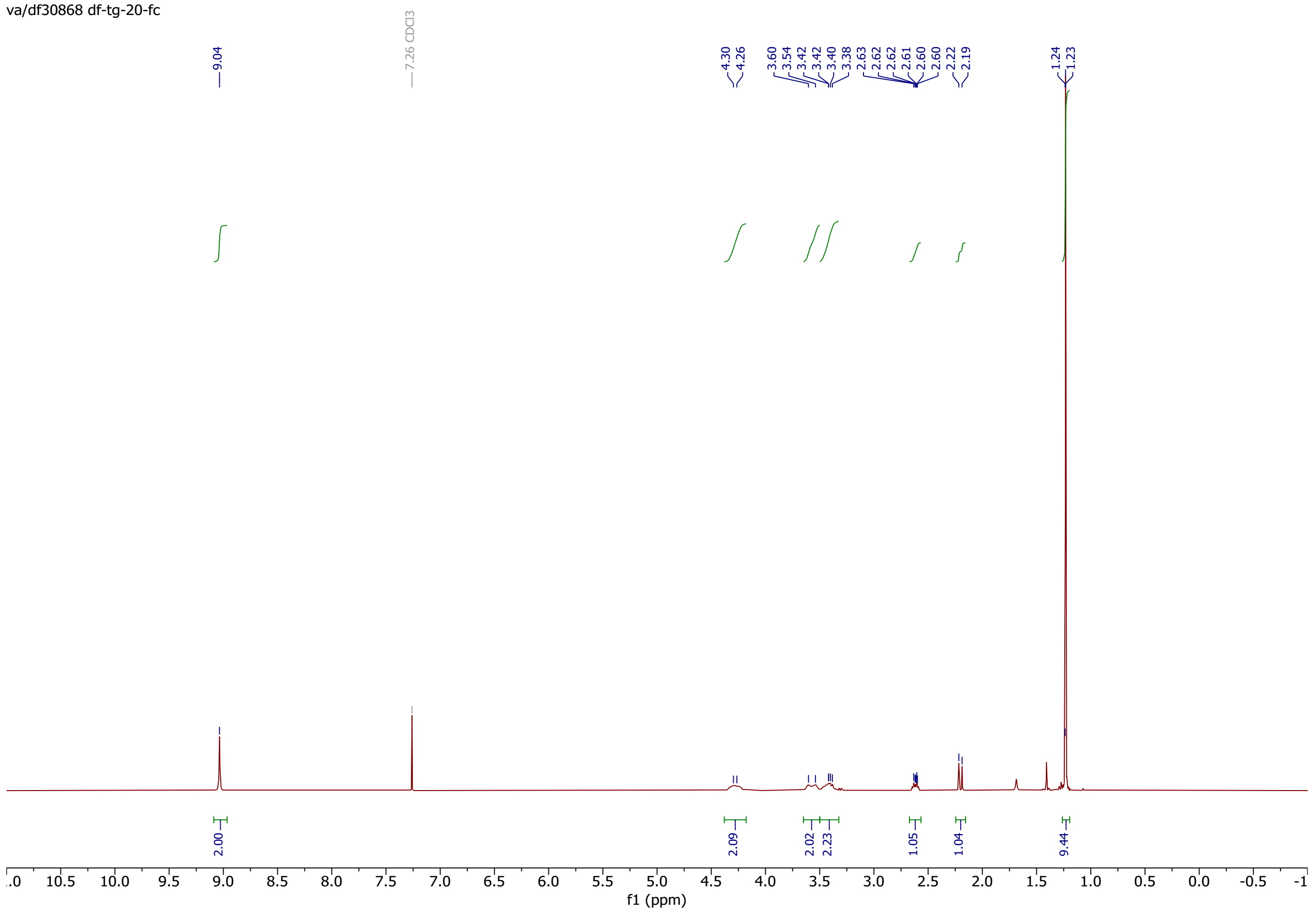

^{1}H NMR of N-Boc N_2 varenicline **SI11**

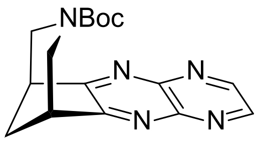

SI11

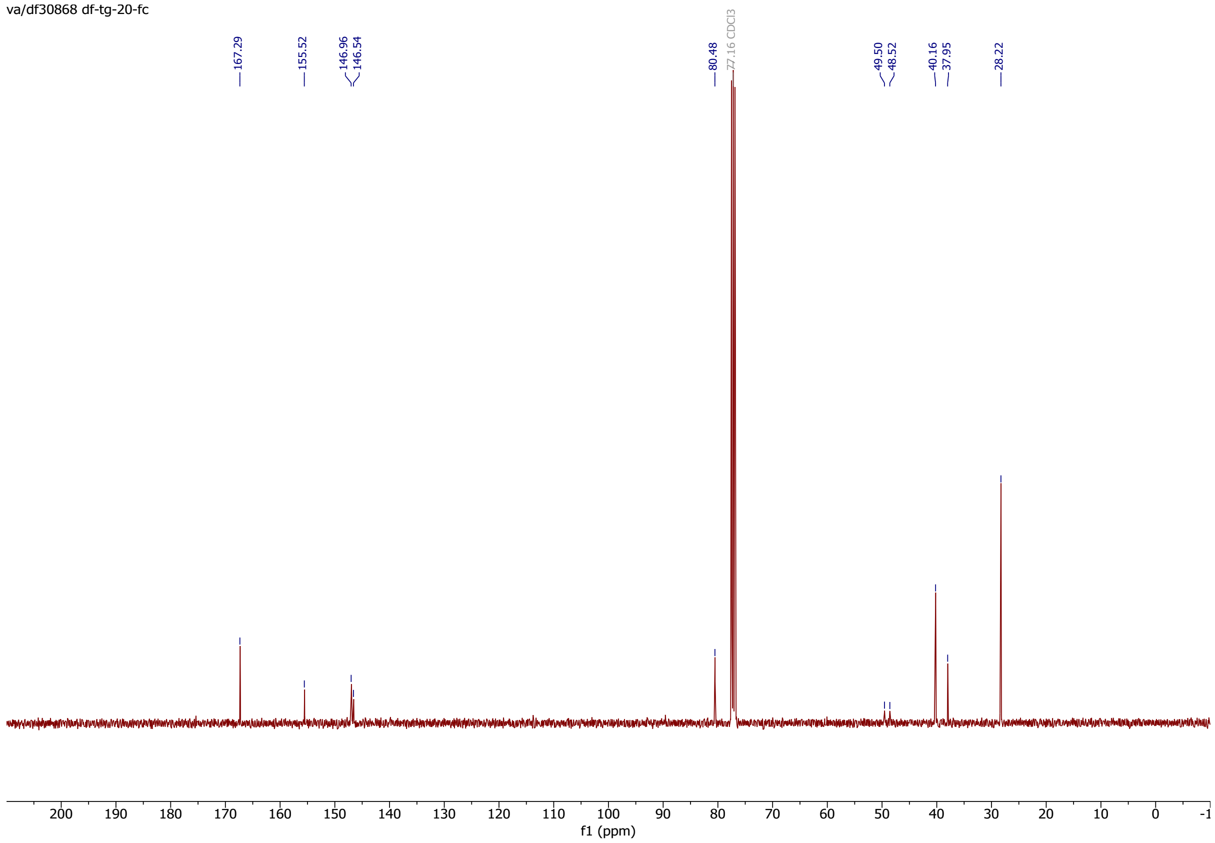

^{13}C NMR of N-Boc N_2 varenicline **SI11**

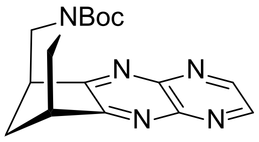

SI11

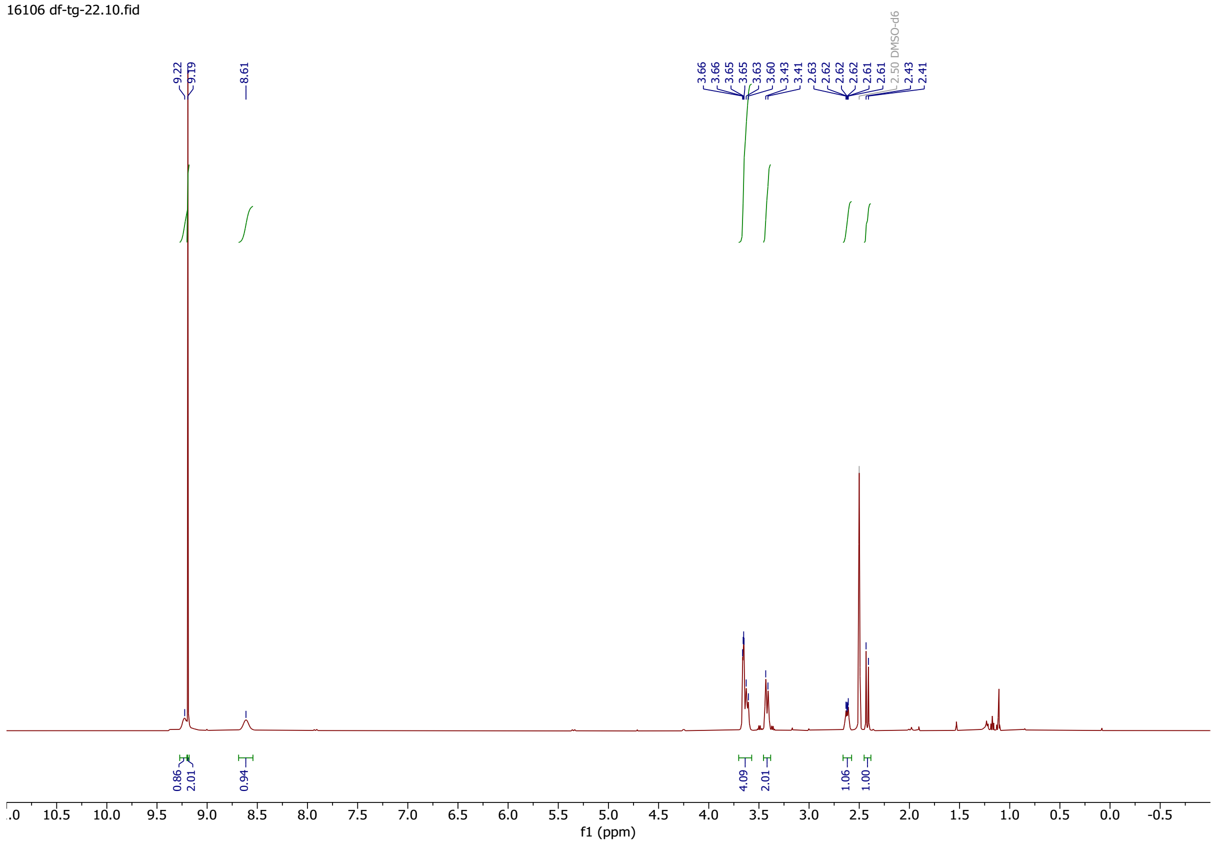

^1H NMR of N_2 varenicline TFA **6**

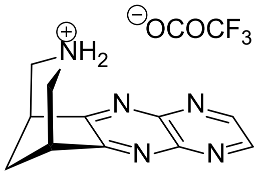

6

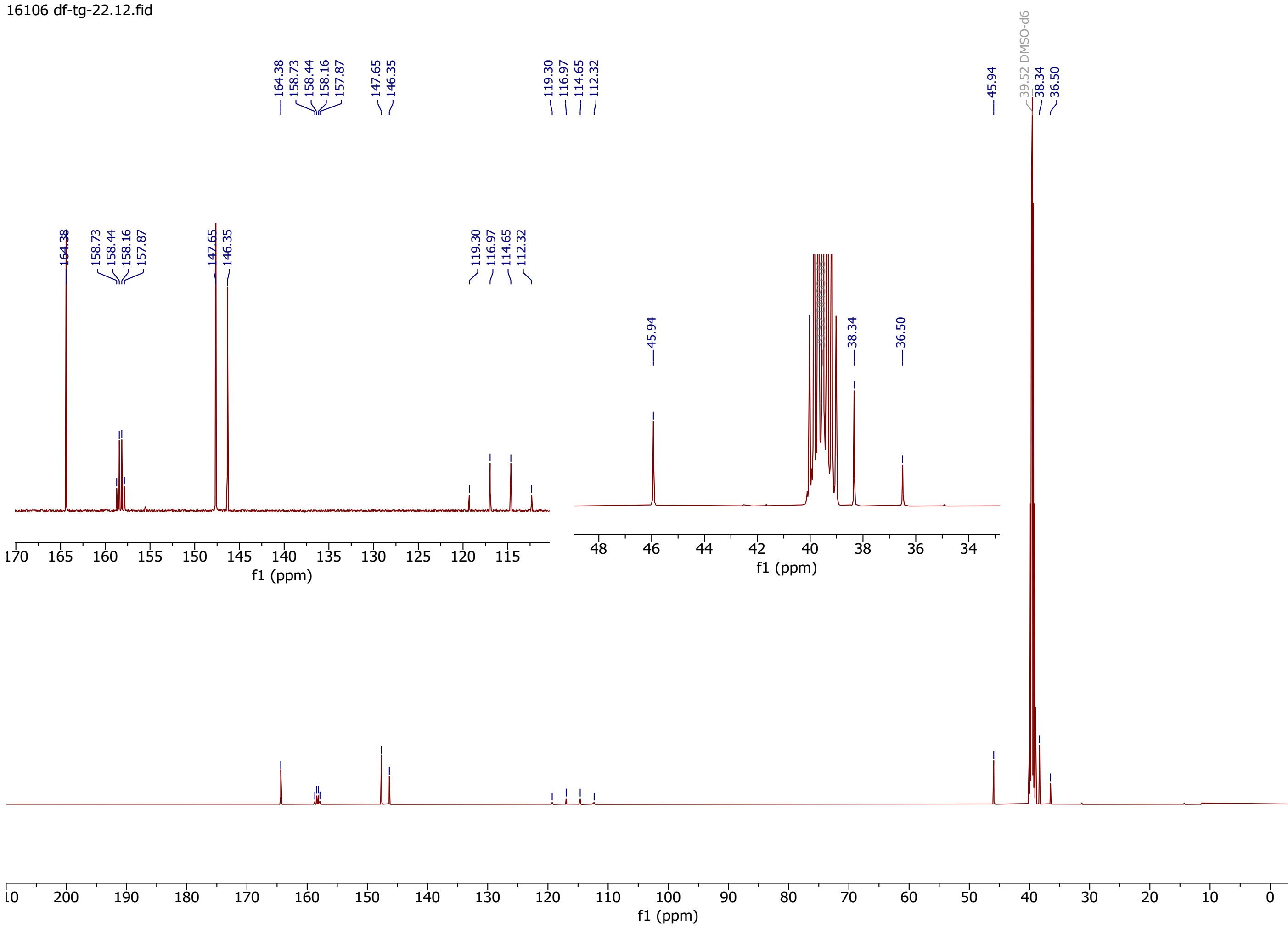

^{13}C NMR (DMSO) of N_2 varenicline TFA **6**

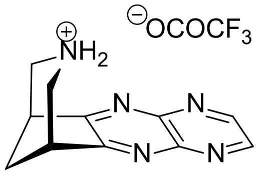

6

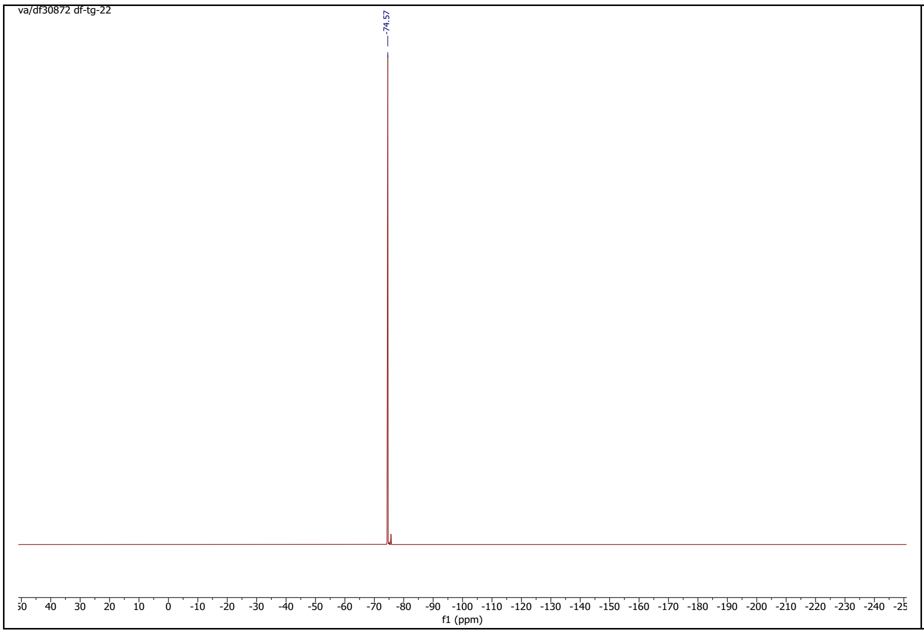

^{19}F NMR of N_2 varenicline TFA **6**

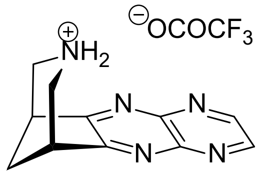

6

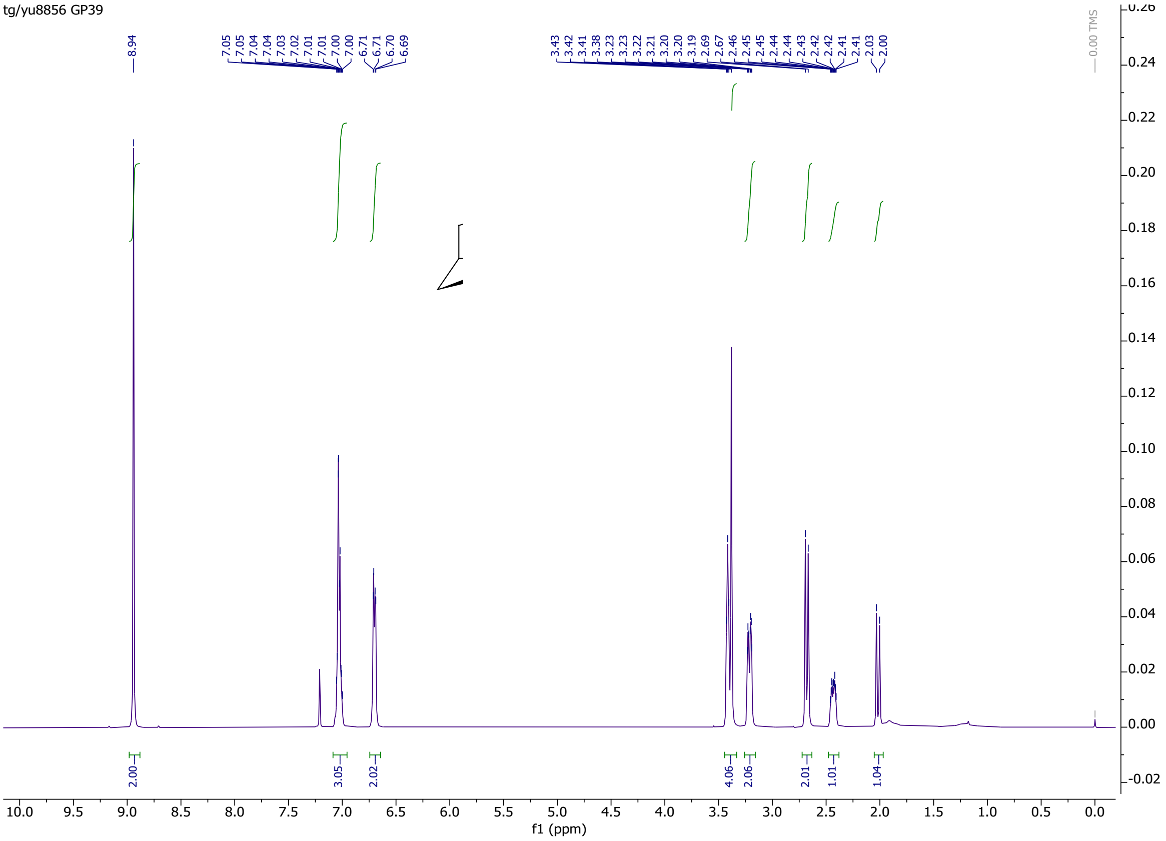

^{1}H NMR of N-Bn N_2 varenicline **SI12**

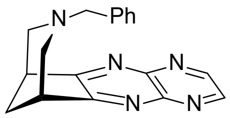

SI12

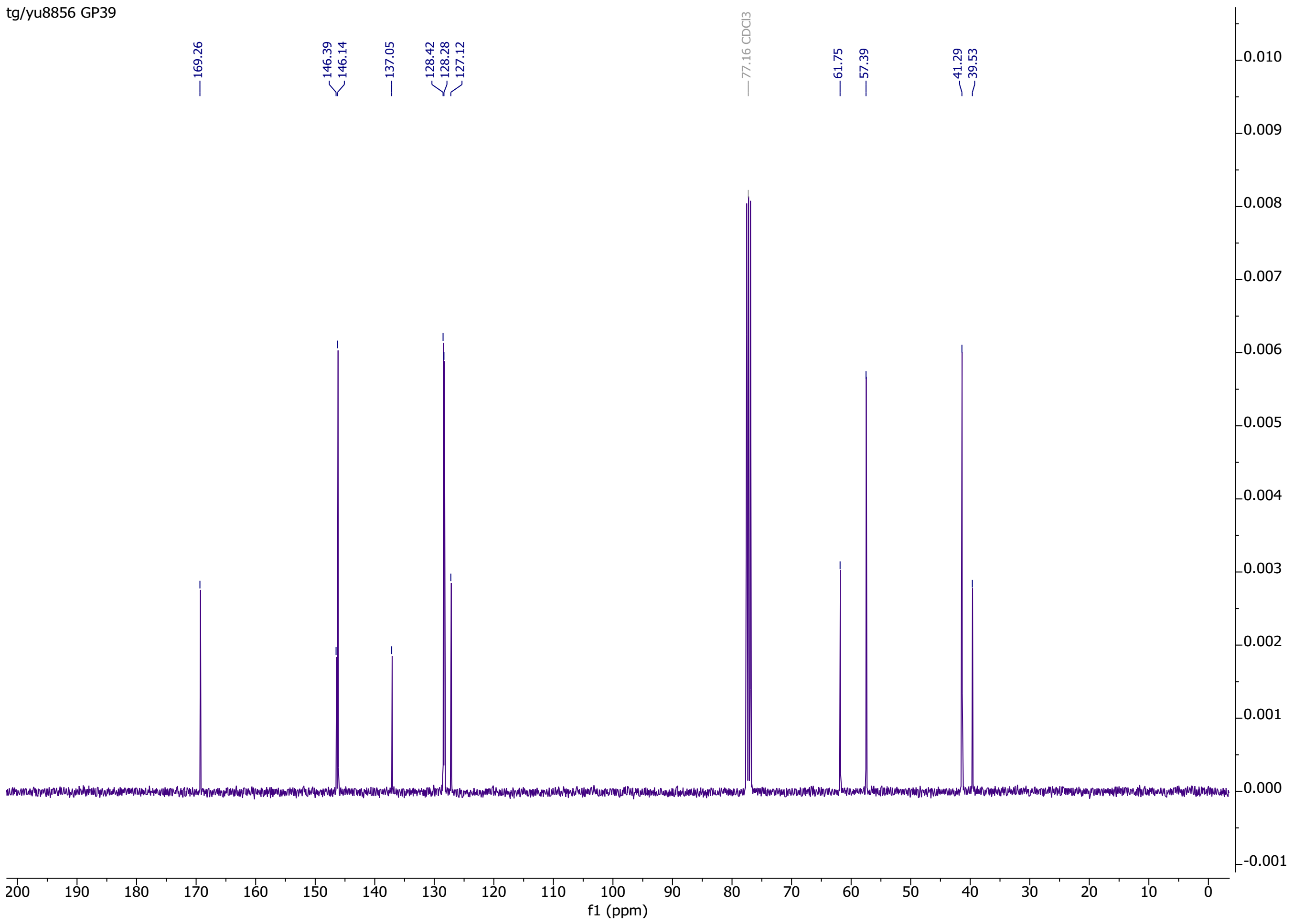

^{13}C NMR of N-Bn N_2 varenicline **SI12**

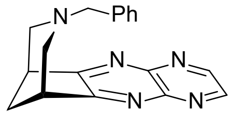

SI12

B. Computational Modelling

(i) Molecular dynamics (MD) simulations

Molecular dynamics (MD) simulations of the extracellular domain (ECD) of human wild-type (WT) $(\alpha4)_3(\beta2)_2$ and $(\alpha4)_2(\beta2)_3$ nicotinic acetylcholine receptors (nAChRs) were performed to identify the key interactions formed by different agonists within the α4-α4 and α4-β2 binding pockets. Since the agonist binding sites are exclusively located in the ECD, the transmembrane and intracellular domains were excluded from the simulations to reduce computational costs. The complexes between the ECD of the human low-sensitivity (LS) isoform of the α4β2 nAChR, namely $(\alpha4)_3(\beta2)_2$, and seven different agonists (varenicline **1**, **Var 1**; nicotine **2**, **Nct 2**; cytisine **3**, **Cyt 3**; C_2 varenicline **4**, **C_2 Var 4**; isovarenicline **5**, **Isovar 5**; N_2 varenicline **6**, **N_2 Var 6;** and, acetylcholine, **ACh**) were constructed using the cryo-EM structure of the complete $(\alpha4)_3(\beta2)_2$ receptor with nicotine bound (PDB code: 6CNK[6]). The binding modes for ACh and cytisine **3** were the same as those previously described in Minguez-Viñas *et.*[7] and Rego-Campello *et al.,*[8] respectively. The binding mode for varenicline **1** and its variants was the same as the one observed in the cryo-EM structure (PDB code: 6UR8[9]).

Complexes between the ECD of the human high-sensitivity (HS) isoform of the α4β2 nAChR, namely $(\alpha4)_2(\beta2)_3$, and varenicline **1** and the three new varenicline variants were also built based on the X-ray structure of the complete HS α4β2 receptor with nicotine bound (PDB code: 5KXI[10]).

All simulated systems contained one agonist bound to each agonist binding site: in the LS isoform, the α-α and both α-β pockets were occupied; conversely, in the HS isoform, each of the two non-consecutive α-β pockets contained one agonist molecule. The structures for the wild-type complexes (Figures S2-S5) were used as the starting inputs for wild-type simulations.

Five mutant complexes formed by the ECD of the human LS α4β2 isoform and varenicline **1**, nicotine **2**, cytisine **3** and ACh were also built and simulated to investigate the dynamic and structural effect of the mutations (Table S1). The mutations introduced were: a serine-to-valine substitution in position 133 in the complementary β2 face of the α-β binding pockets (hereafter named β2S133V), a threonine-to-valine substitution in position 183 in the principal α4 face of the α-β and α-α pockets (α4T183V), a threonine-to-valine substitution in position 139 in the complementary α4 face of the α-α pocket (α4T139V) and two double mutants, one

simultaneously co-expressing the α4T183V and β2S133V substitutions (β2S133Vα4T183V) and the other the α4T139V and β2S133V mutations (β2S133Vα4T139V). Note that the numbering here refers to Uniprot sequences P43681 and P17787 for the human α4 and β2 subunits, respectively. Starting structures for simulations of the mutants were created using the mutagenesis tool in PyMOL.[11]

In this work, a total of 32 different systems were investigated (Table S1). The simulations for ECD of the human HS α4β2 isoform with nicotine **2,** cytisine **3** and ACh were taken from our previous work.[7, 8]

All titratable residues were modelled in their standard state at a physiological pH (i.e. aspartates and glutamates were negatively charged, lysines and arginines were positively charged, and histidines were neutral), similarly to our previous work.[7, 8] All agonists (varenicline **1**, nicotine **2**, cytisine **3**, **C_2** varenicline **4,** isovarenicline **5** and ACh) were considered to be positively charged.

MD simulations were performed using Gromacs.[12] The Amber ff99SB-ILDN forcefield[13] was used to describe the protein, whereas the parameters for varenicline **1**, nicotine **2,** cytisine **3** and ACh were taken from our previous work.[7, 8] Acpype[14] was used to generate Amber-compatible GAFF parameters for **C_2** varenicline **4** and isovarenicline **5**. All systems were solvated using the TIP3P water model.[15] The simulations were performed using a 2-fs time-step for the integration of the equations of motion. Non-bonded long-range electrostatic interactions were calculated using the smooth particle mesh Ewald method,[16] with a Fourier grid spacing of 0.16 Å and a 1.2 Å cutoff for direct contributions. A 12 Å cut-off was also used for the van der Waals interactions, with long-range dispersion corrections for the energy and pressure. The LINCS algorithm[17] was used to constrain bonds in the protein and agonists, and SETTLE[18] was used to keep water molecules rigid.

Prior to the unrestrained simulations, all systems were energy minimized and initialized using the protocol described in Rego-Campello *et al.*[8] Briefly, this procedure involves a three-step energy minimization: in the first step, harmonic restraints were applied to all non-hydrogen atoms; in the second step, to Cα atoms only; and, in the third step, no restraints were used. After energy minimization, a short MD simulation step with all the non-hydrogen atoms restrained was performed, followed by a second short simulation in which position restraints

were applied to Cα atoms only. All the unrestrained simulations started from these relaxed conformations.

Unrestrained simulations were performed at a constant temperature of 310 K using the velocity-rescaling thermostat,[19] with separate couplings for the solutes (protein and ligands) and solvent and a relaxation time constant of 0.1 ps. The pressure within the simulations was maintained at 1 bar using the Parrinello-Rahman barostat[20, 21] with a coupling constant of 1 ps. Each system was simulated three times, each 300 ns, leading to a total of 27 μs of simulation time across all systems. MD input and output files (including the simulation trajectories) are publicly available *via* the University of Bristol Research Data Repository (https://data.bris.ac.uk/).

(ii) Analysis of MD simulations

The trajectories were analyzed using Gromacs tools.[12] The structural stability of the simulated systems was examined by monitoring the Cα root mean square deviation (RMSD) relative to the starting structures. All systems remained stable over the simulation time, with the average Cα RMSD profiles showing a plateau after ~20 ns (Figures S7 and S27). The receptor's secondary structure content was monitored using the DSSP software,[22] with minimal secondary structure loss after the simulation time (Figures S8 and S27).

Principal component analysis (PCA) was performed to examine the sampling and equilibration of the replicates (Figure S9), as previously described.[23, 24] All replicates were combined before the analysis so that they all shared a common subspace, and their behavior could be directly compared. Each trajectory used for PCA contained one conformation per nanosecond per replicate with the protein Cα atoms. The two principal components (PC) 1 and 2 were used to assess the sampling and equilibration of the simulations. Generally, the different replicates sampled different regions of conformational space, thus improving the overall sampling for each system and helping to mitigate sampling problems.

Cα root mean square fluctuations (RMSF) were calculated to characterize the dynamic behavior of the receptor across the various systems (Figures S10 and S28), and a Student's t-test was used to assess the significance of the differences observed between wild type and mutants (similarly to Oliveira *et al.*[23]) (Figure S10). A sample size of three was used for the t-

test, which assumed the two samples were independent and the dependent variable was normally distributed.

The RMSD of the agonists was monitored to assess the stability of their initial binding poses (Figures S11-S13 and S27). Despite changes in binding mode observed for some ligands (e.g. ACh in the α-α binding pocket), all agonists remained bound to their respective binding sites in both the wild-type and mutant systems. The only exception was the ACh molecule bound to the second α-β pocket of replicate 3 in the β2S133Vα4T139V-ACh system, which exited the pocket after about 112 ns (Figure S13G). This analysis revealed distinct dynamic behaviors between agonists: ACh exhibited high positional and conformational variability; in contrast, the bulkier ligands, probably due to additional interactions with the protein compared to ACh,[25-29] showed reduced mobility and generally maintained an orientation closer to the initial one throughout the simulation time. Differences in agonist dynamics were also observed between the α–α and α–β pockets, mainly for nicotine **2** and cytisine **3**, with these ligands showing increased mobility within the α–α pocket.

Probability density maps were generated to visualize the spatial distribution and identify preferred positions for the agonists (Figures S14-S16 and S30) for the wild-type and mutant complexes. For each agonist-receptor system, the maps were calculated by combining the entire trajectories for all replicates of that system.

Statistical correlations between the protonated nitrogen atom of varenicline **1**, nicotine **2,** cytisine **3** and ACh and all the Cα atoms in the wild-type receptor were determined to identify the regions in the protein whose motions are coupled to the ligands (Figure S22). The correlations were determined by combining all replicates' trajectories for each individual system, with each trajectory containing a total of 90001 conformations.

The distance between the ammonium centers present in the ligands (piperidines in the case of varenicline **1** and its derivatives and cytisine **3;** pyrrolidine of nicotine **2**; and quaternary ammonium nitrogen of ACh) and the side chains of TrpB (Trp182 located in loop B in the principal α4 face of the pockets), TyrA (Tyr126 in loop A in the principal α4 face of the pockets), and TrpD (Trp88 and Trp82 in loop D in the complementary α4 face of the α–α pocket and the complementary β2 face of the α–β pocket) were determined (Figures S17-S19 and S29). These

distances indicate persistent cation-π interactions between the ligands and TrpB, and occasionally with TyrA and TrpD.

(iii) Supporting figures and tables

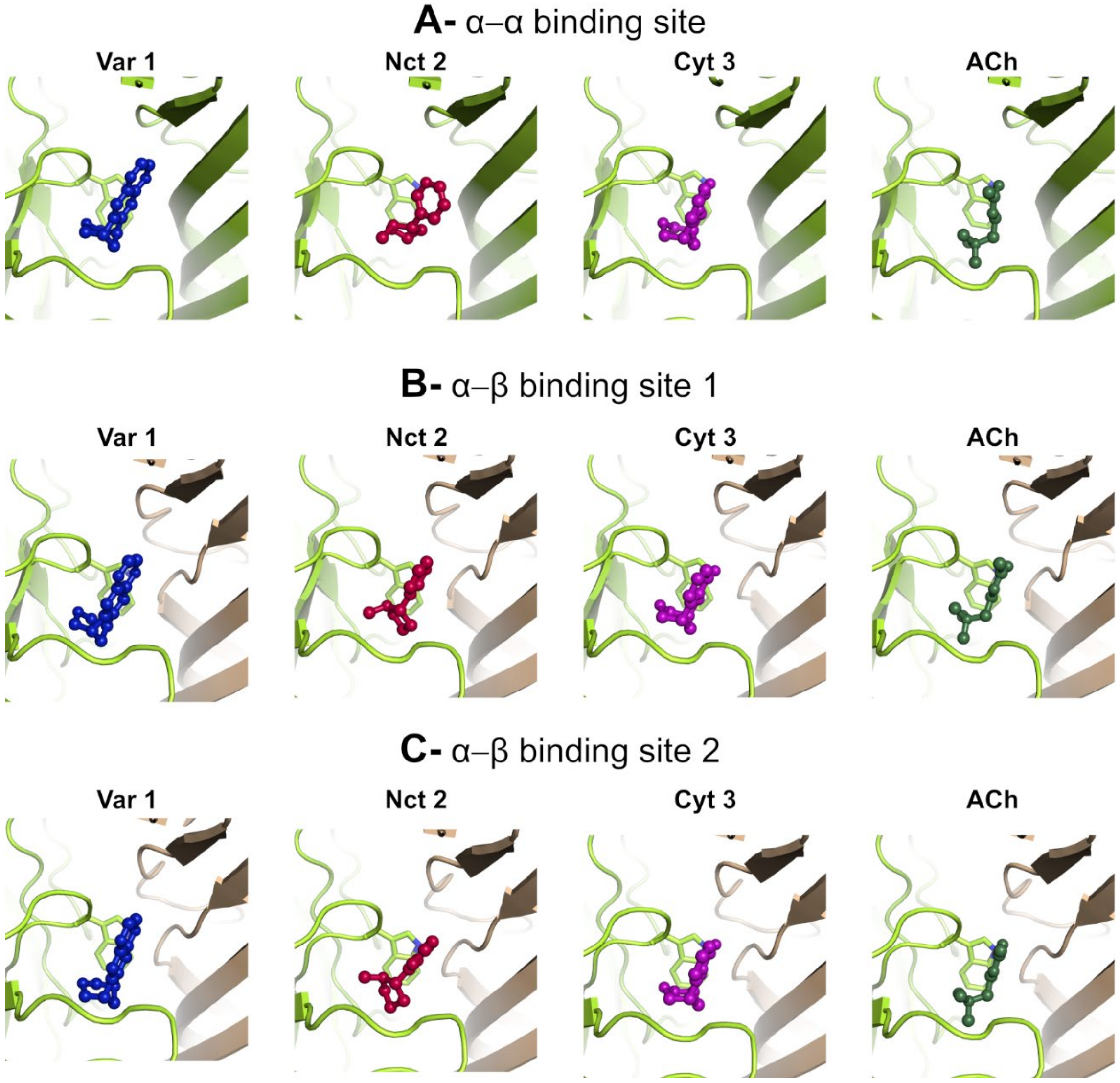

Figure S2- Agonist binding mode in the α-α and α-β pockets in the wild-type LS isoform of the α4β2 nAChR. (**A**) Binding mode of **1**, nicotine **2**, cytisine **3,** and ACh in the α-α pocket. (**B**) Binding mode of varenicline **1**, nicotine **2**, cytisine **3** and ACh in the first α-β pocket (**C**) Binding mode of varenicline **1**, nicotine **2**, cytisine **3** and ACh in the second α-β pocket. Agonists are represented as balls-and-sticks and TrpB (W182 in the principal α4 face), a tryptophan residue that provides the anchor point for the agonist, shown with sticks. The α4 and β2 subunits are colored in yellow and light brown, respectively.

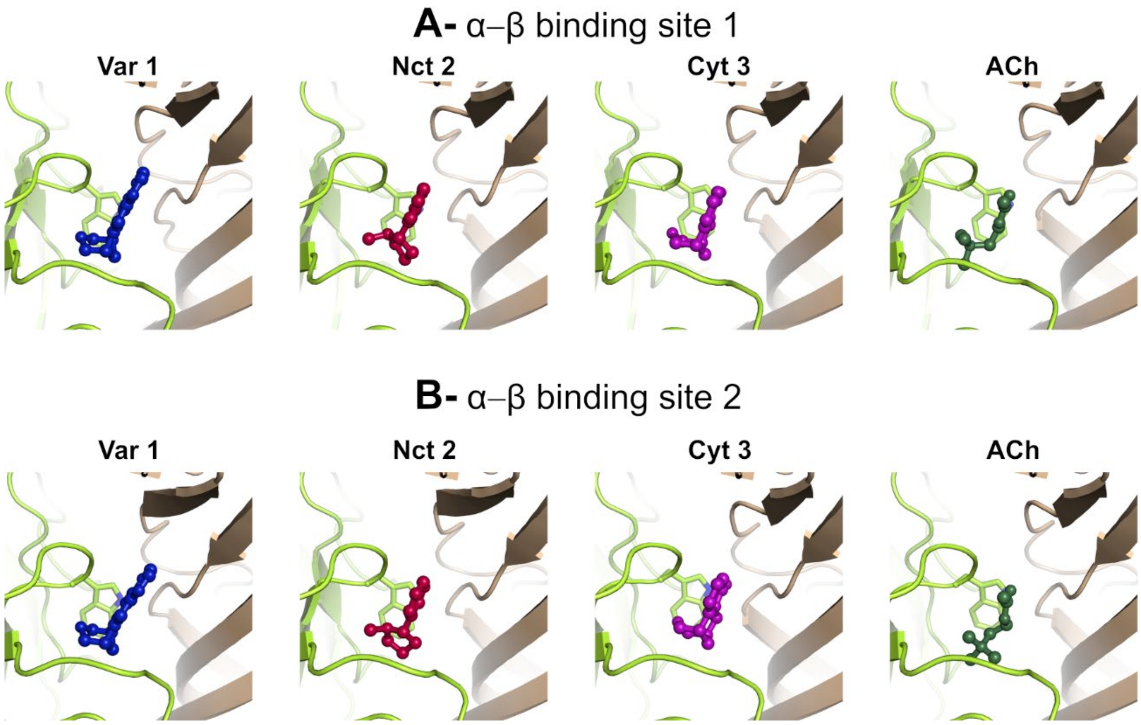

Figure S3- Agonist binding mode in the α-β pockets in the wild-type HS isoform of the α4β2 nAChR. (**A**) Binding mode of varenicline **1**, nicotine **2**, cytisine **3**, and ACh in the first α-β pocket (**B**) Binding mode of varenicline **1**, nicotine **2**, cytisine **3**, and ACh in the second α-β pocket. Agonists are represented as balls-and-sticks and TrpB (W182 in the principal α4 face), a tryptophan residue that provides the anchor point for the agonist, shown with sticks. The α4 and β2 subunits are colored in yellow and light brown, respectively.

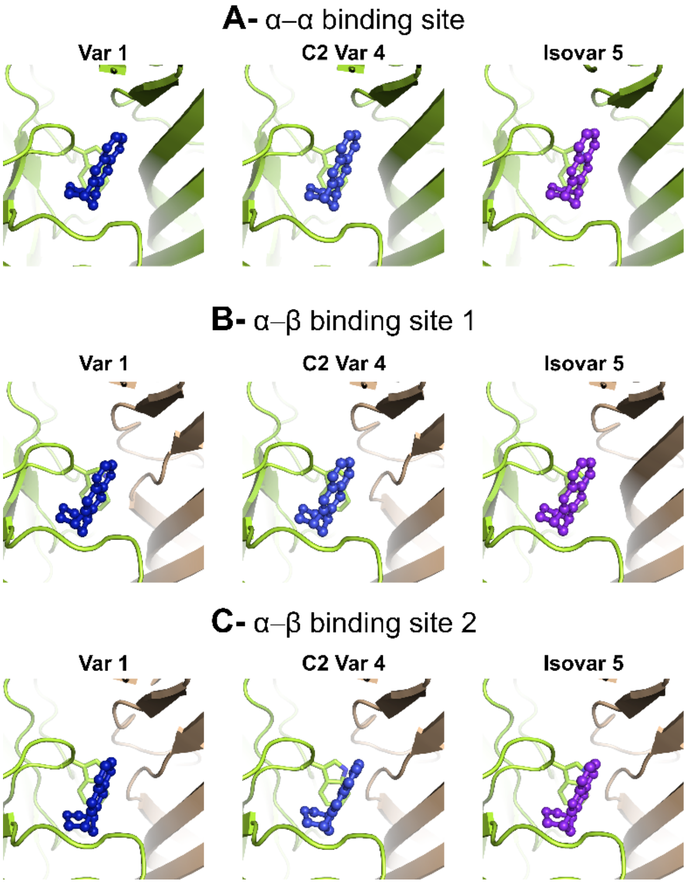

Figure S4- Binding mode of varenicline **1** and its variants in the α-α and α-β pockets of the wild-type LS form of the α4β2 nAChR. (**A**) Binding mode of varenicline **1**, C_2 varenicline **4,** and isovarenicline **5** in the α-α pocket. (**B**) Binding mode of varenicline **1**, C_2 varenicline **4**, and isovarenicline **5** in the first α-β pocket (**C**) Binding mode of varenicline **1**, C_2 varenicline **4**, and isovarenicline **5** in the second α-β pocket. Agonists are represented as balls-and-sticks and TrpB (W182 in the principal α4 face), a tryptophan residue that provides the anchor point for the agonist, shown with sticks. The α4 and β2 subunits are colored in yellow and light brown, respectively.

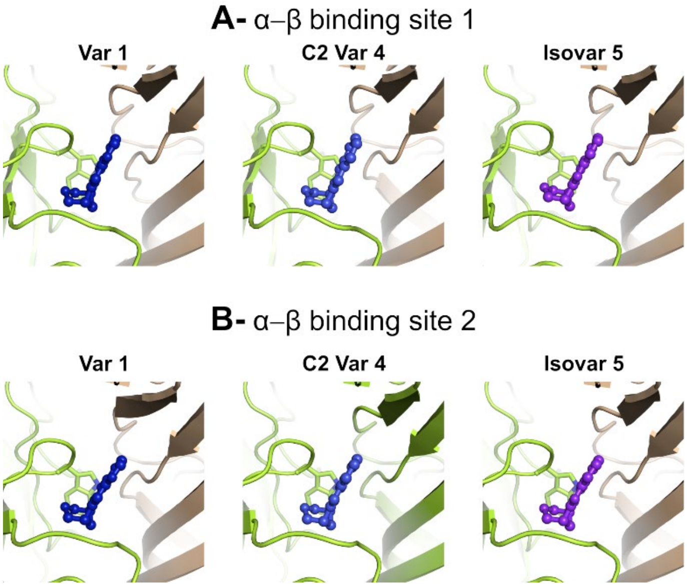

Figure S5- Binding mode of varenicline **1** and its variants in the α-β pockets of the wild-type HS isoform of the α4β2 nAChR. (**A**) Binding mode of varenicline **1**, C_2 varenicline **4**, and isovarenicline **5** in the first α-β pocket (**B**) Binding mode of varenicline **1**, C_2 varenicline **4**, and isovarenicline **5** in the second α-β pocket. Agonists are represented as balls-and-sticks and TrpB (W182 in the principal α4 face), a tryptophan residue that provides the anchor point for the agonist, shown with sticks. The α4 and β2 subunits are colored in yellow and light brown, respectively.

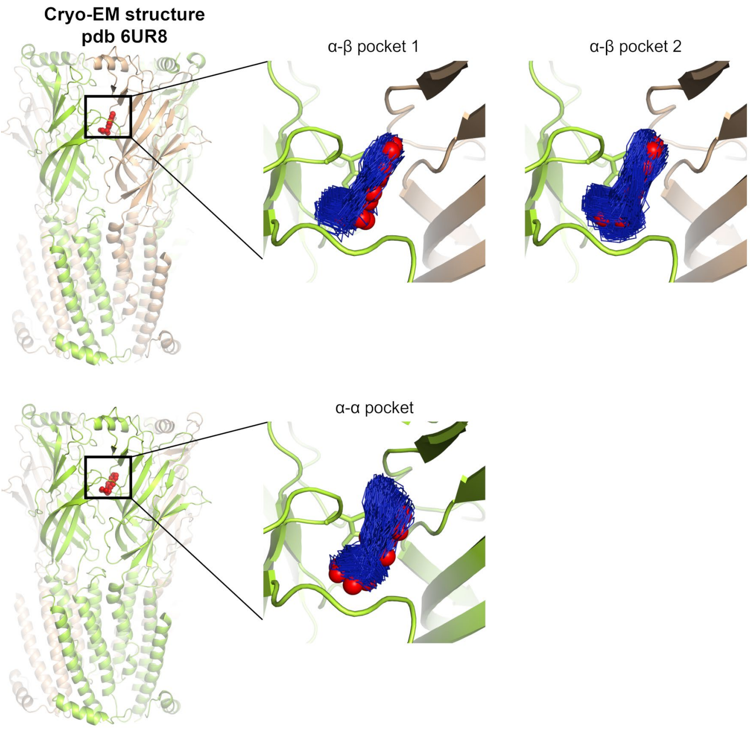

Figure S6- Comparison of varenicline **1** binding mode in the cryo-EM structure 6UR8[9] (red spheres) and during the MD simulations (blue sticks) in the α4β2 nAChR. The blue sticks represent the binding modes of varenicline **1** throughout the simulations, sampled at one frame per nanosecond per replicate. These binding modes are illustrated within both the α-β and α-α pockets of the LS isoform of the wild-type α4β2 nAChR. The anchoring residue TrpB (W182 in the principal α4 face) is shown in yellow sticks. The α4 and β2 subunits are colored in yellow and light brown, respectively.

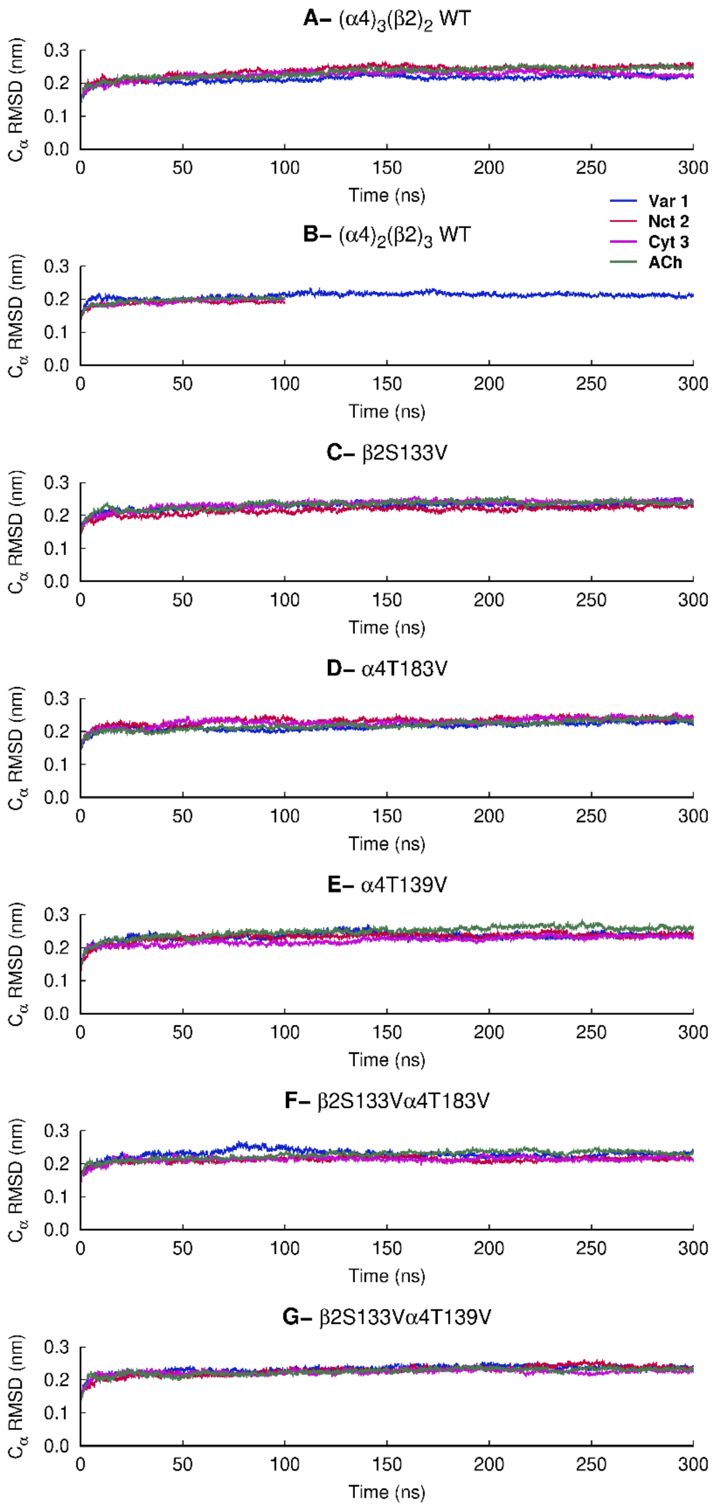

Figure S7- Temporal evolution of the average Cα RMSD for the varenicline **1**, nicotine **2**, cytisine **3** and ACh-bound systems. The Cα RMSD was calculated relative to each system's starting structure and the averages obtained over all replicates for each system. Please note that the simulations of the complexes formed by the HS isoform of the α4β2 wild-type receptor and nicotine **2**, cytisine **3** and ACh (panel B) were taken from our previous work.[7,8] Please zoom in on the image for detailed visualization.

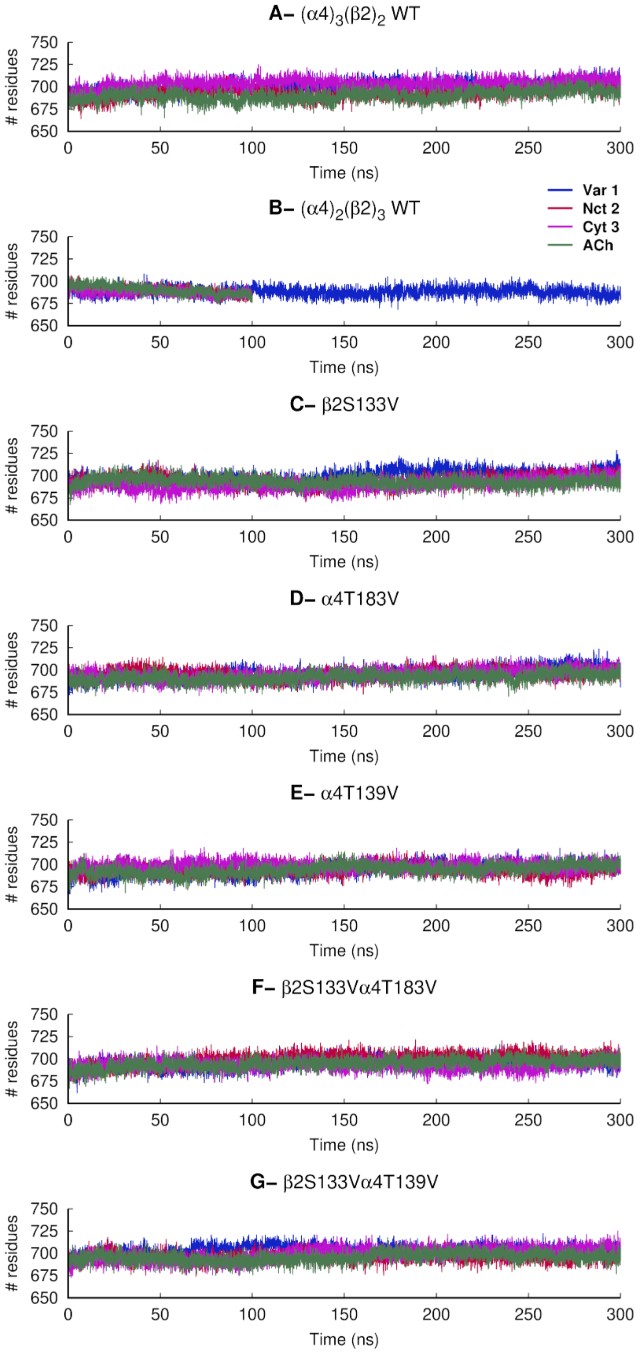

Figure S8- Temporal evolution of the number of residues involved in secondary structure features for the varenicline **1**, nicotine **2**, cytisine **3** and ACh-bound systems. The secondary structure assignment was performed with the DSSP software[22] and includes all residues assigned to α-helix, π-helix, 3_{10}- helix, 5-helix, β-sheet, β-strand and β-bridge secondary structure classes. The averages were obtained over all replicates for each system. The trajectories for the complexes formed by the HS isoform of the α4β2 wild-type receptor and nicotine **2**, cytisine **3** and ACh (in panel B) were taken from our previous work.[7,8] Please zoom in on the image for detailed visualization.

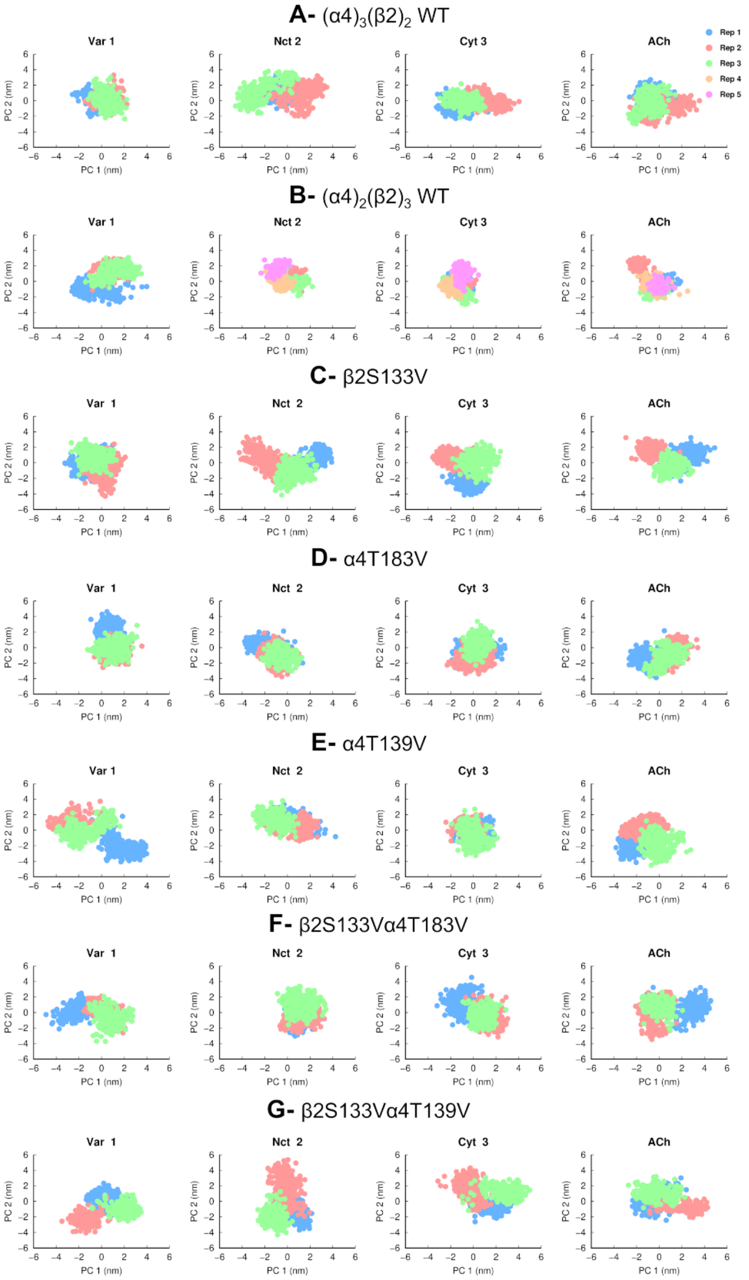

Figure S9- Principal component analysis (PCA) of the replicates for the LS (**A**) and HS (**B**) isoforms of the α4β2 wild-type and mutant (**C-G**) simulations. All replicates for each system were combined before the analysis so that they all shared a common space, and their behavior could be directly compared. Each trajectory used for PCA contained one conformation per nanosecond per replicate with the protein Cα atoms. Principal component (PC) 1 and 2 were used to assess the sampling of the conformational space. This analysis shows that, generally, the different replicates explore different regions of the space, thus improving the overall sampling for each system. Please zoom in on the image for detailed visualization.

A- β2S133V

Var 1

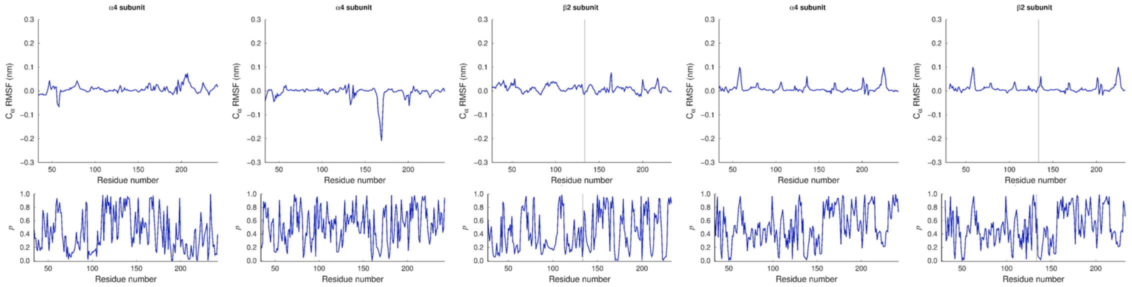

Nct 2

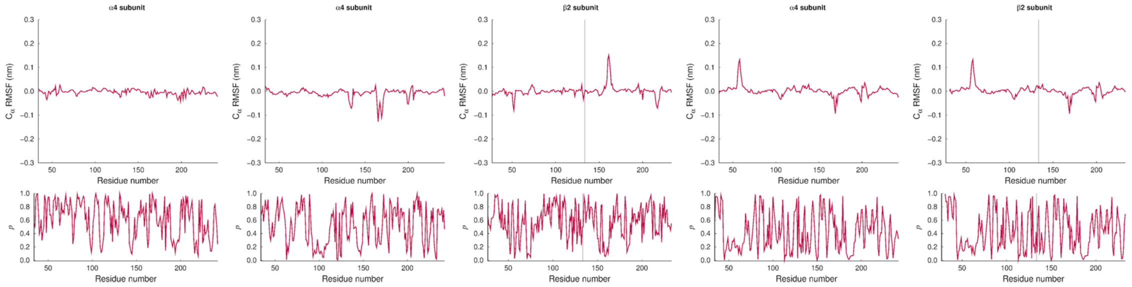

Cyt 3

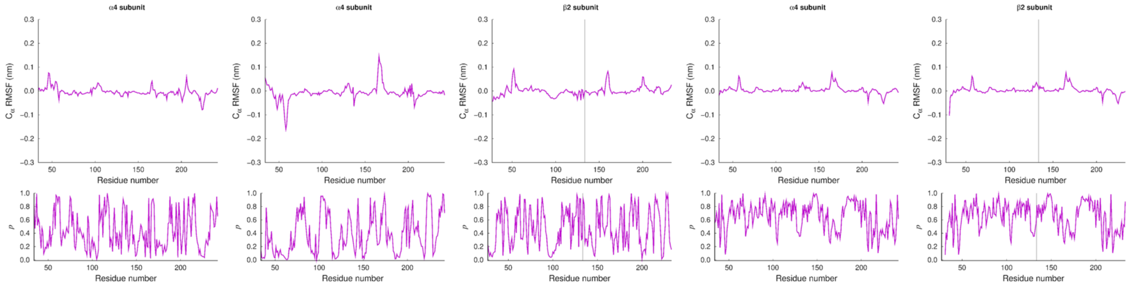

ACh

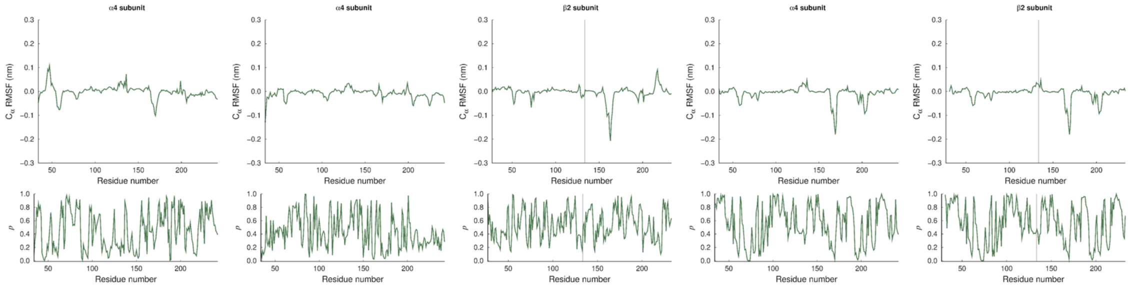

B- α4T183V

Var 1

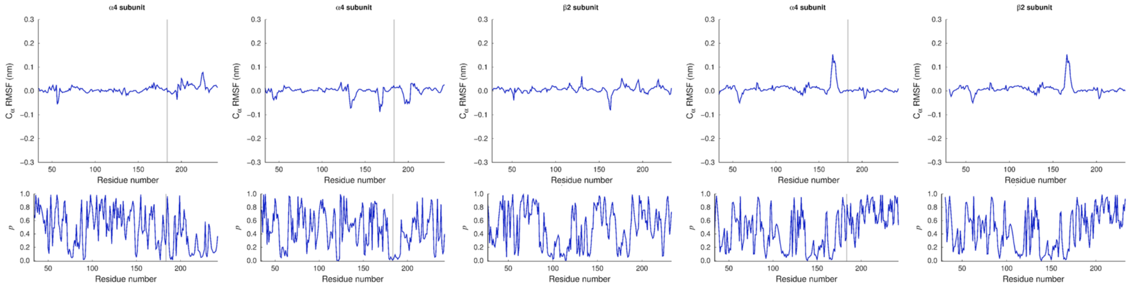

Nct 2

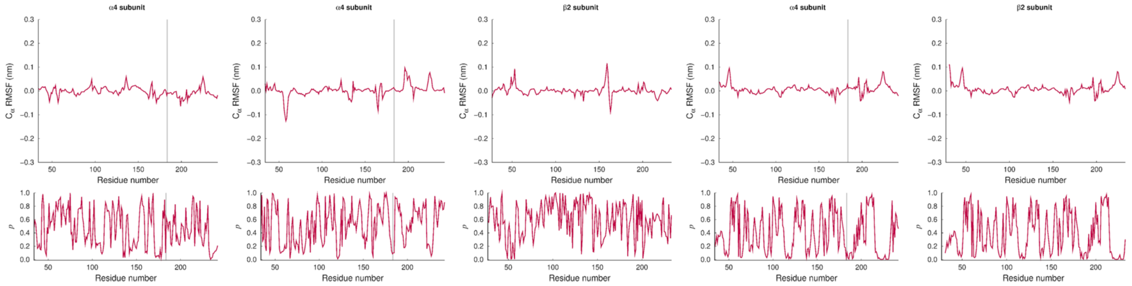

Cyt 3

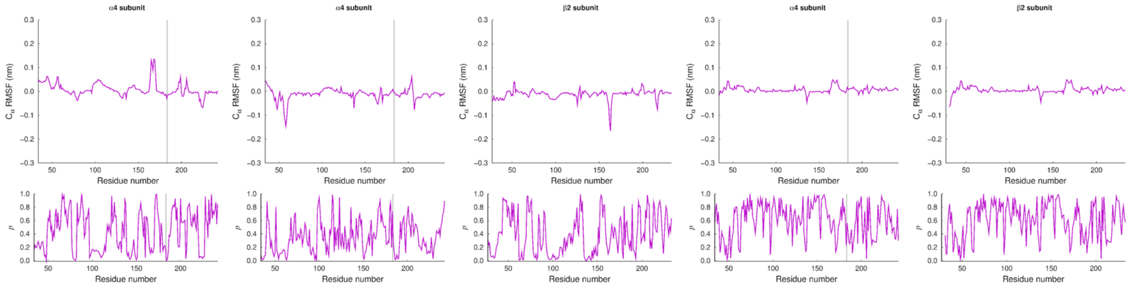

ACh

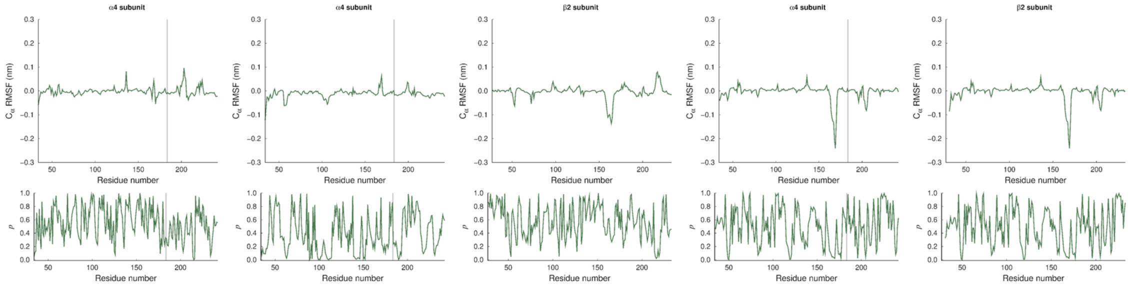

C- α4T139V

Var 1

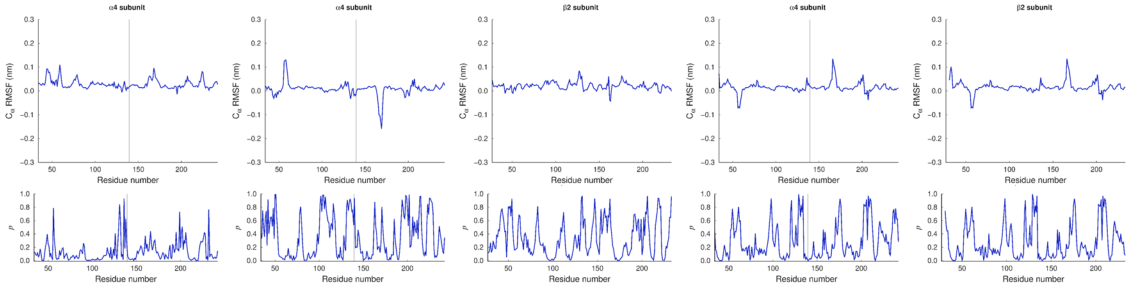

Nct 2

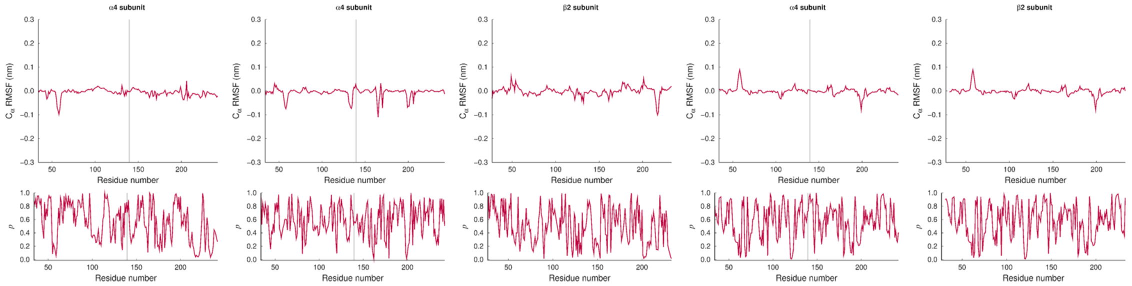

Cyt 3

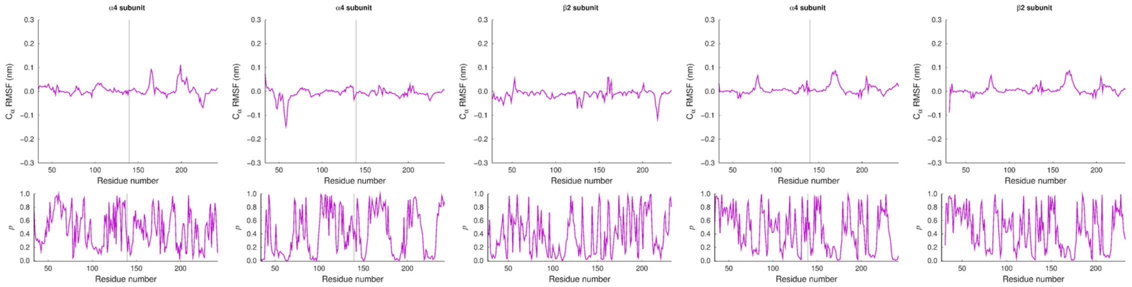

ACh

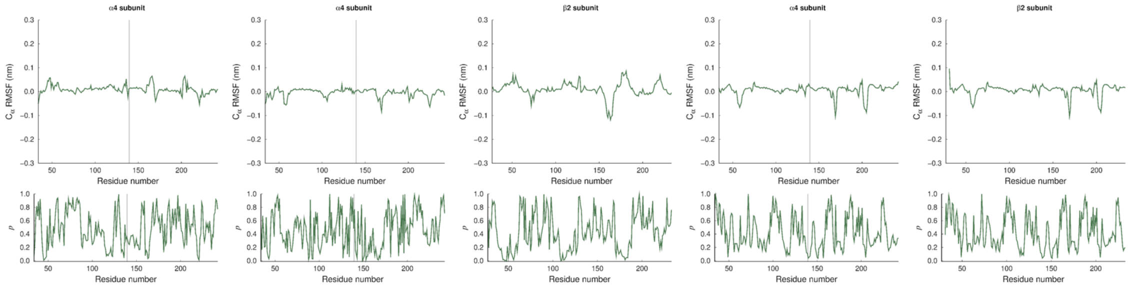

D- β2S133Vα4T183V

Var 1

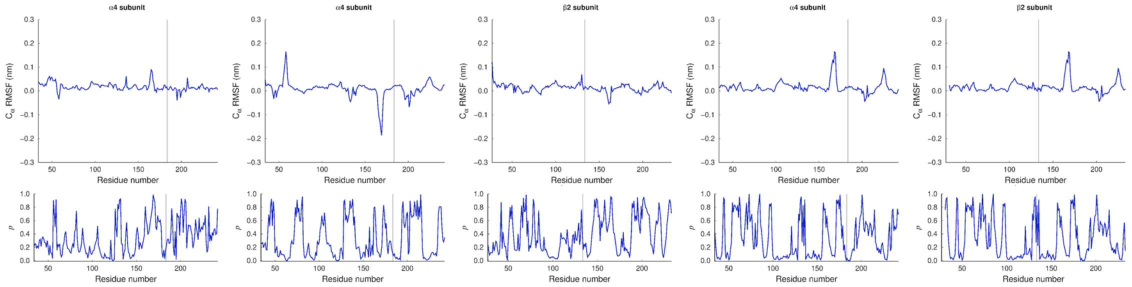

Nct 2

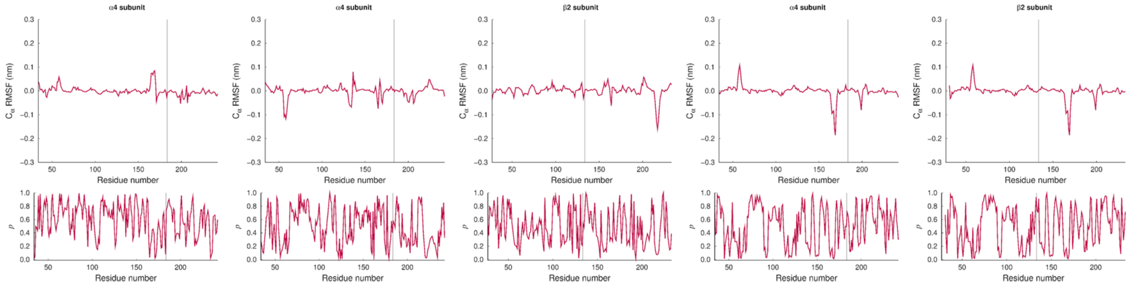

Cyt 3

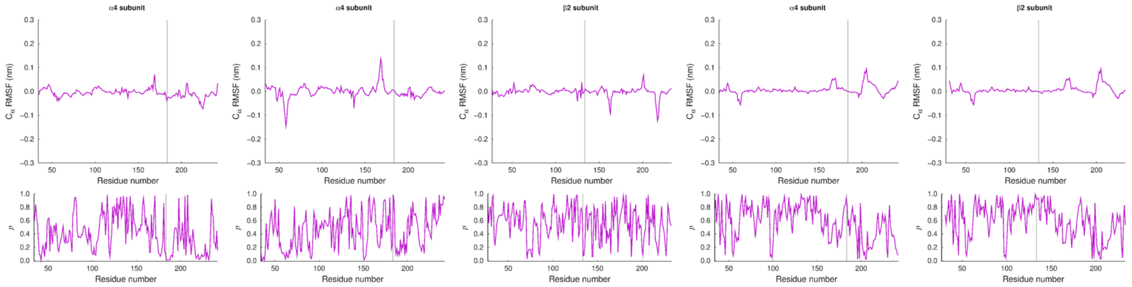

ACh

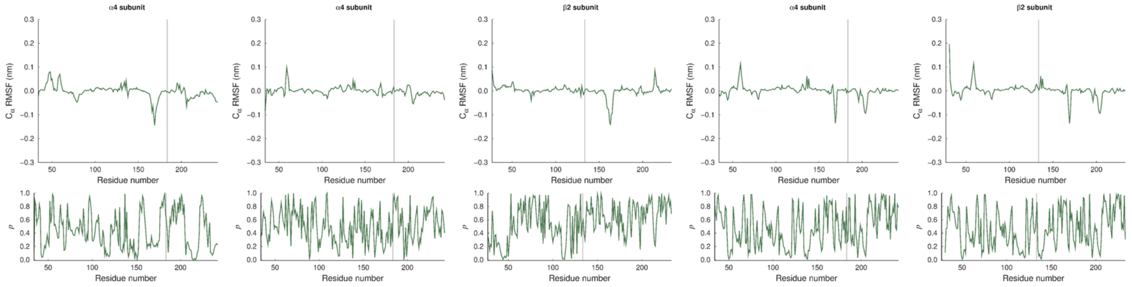

E- β2S133Vα4T139V

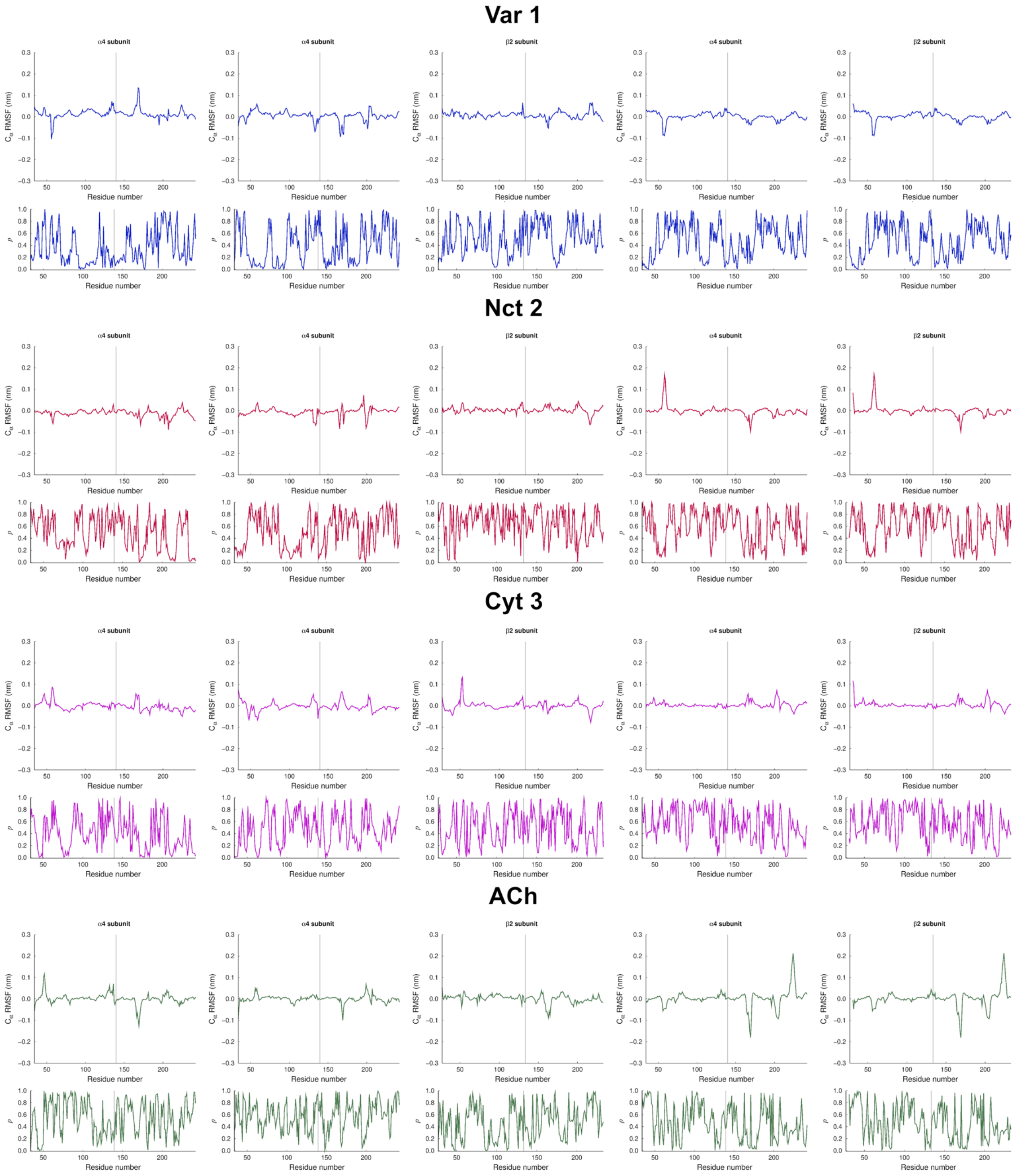

Figure S10- Average Cα RMSF difference between wild-type and mutant systems (**panels A-E**) and associated *p* values. A Student's t-test was used to compare the wild-type and mutant systems and to assess the significance of the differences. Positive values in the Cα RMSF difference plots correspond to a greater flexibility of the wild type during the simulations, whereas negative values correspond to an increased flexibility of the mutant. The vertical grey lines highlight the position of the mutations. Please zoom in on the image for detailed visualization.

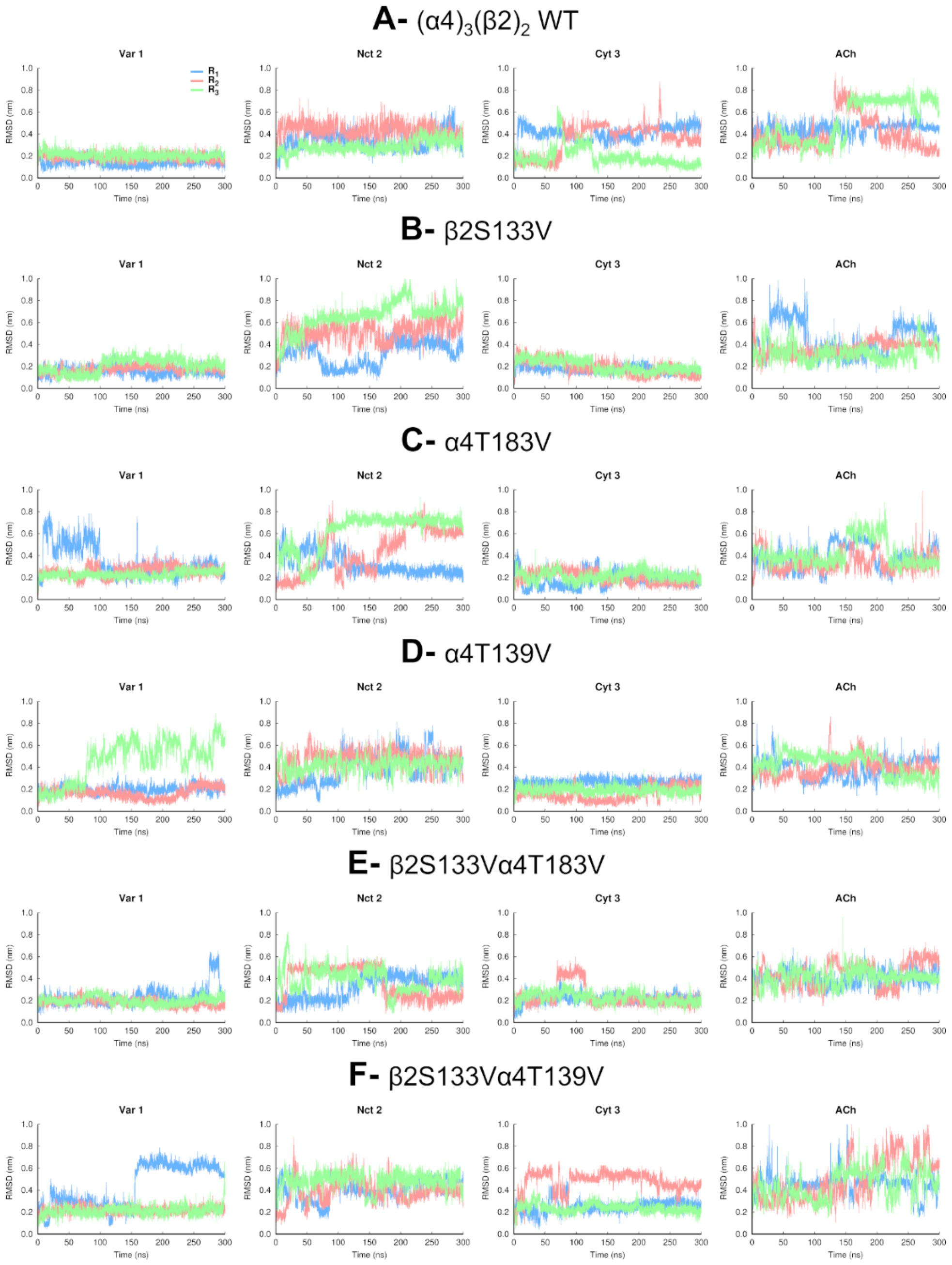

Figure S11- Temporal evolution of the RMSD for the agonists bound to the α-α binding pocket in the LS isoform of the α4β2 wild-type (**A**) and mutant (**B**-**F**) systems. The RMSD was determined with respect to the initial binding mode of the agonists at the start of the simulations. Note that the α-α binding pocket only exists in the LS isoform of the receptor. This analysis shows that despite the changes in binding mode observed for some ligands, all agonists remained stably bound to their respective binding sites. Please zoom in on the image for detailed visualization.

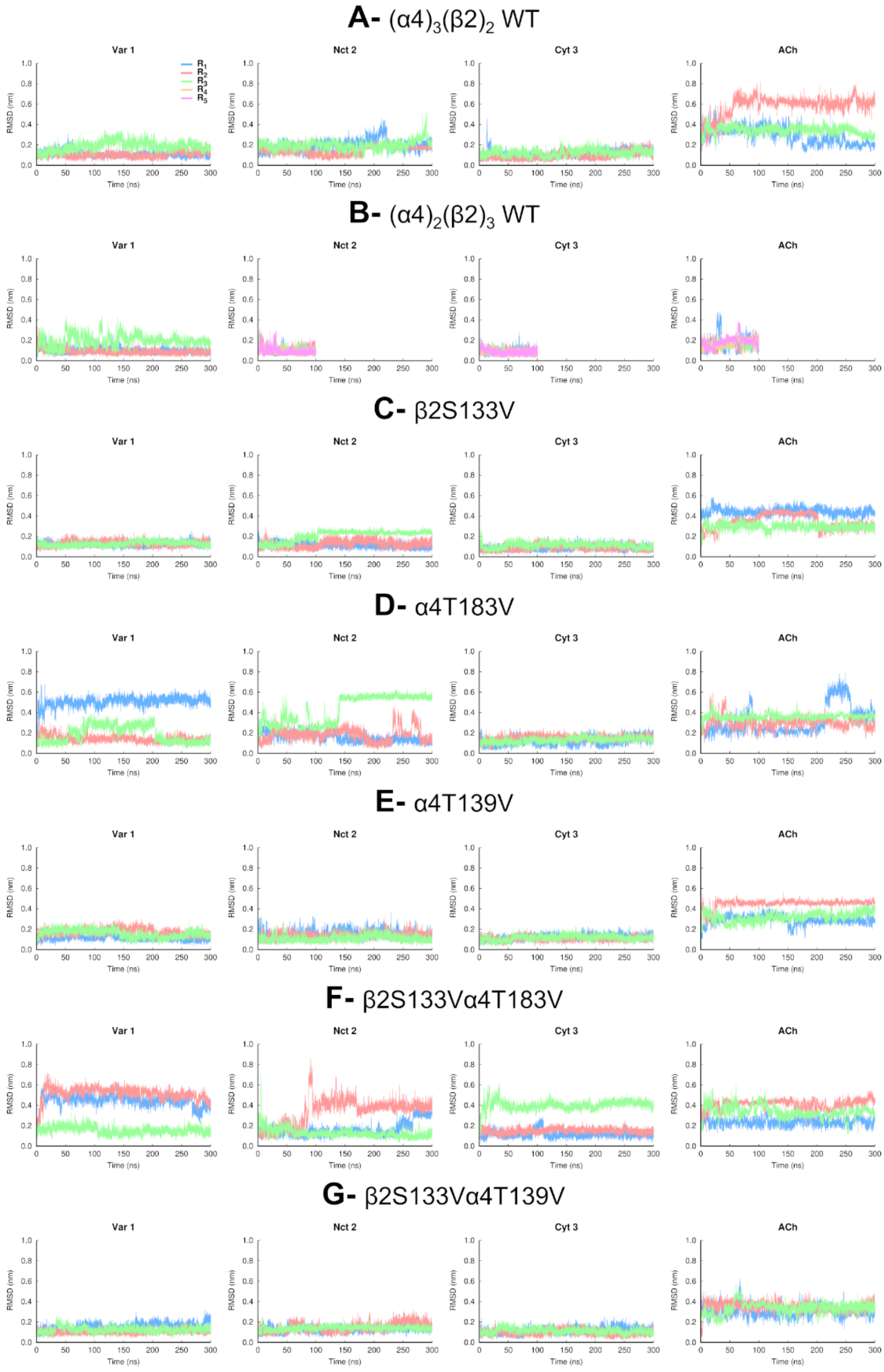

Figure S12- Temporal evolution of the RMSD for the agonists bound to the first α-β pocket in the LS (**A**) and HS (**B**) isoforms of the α4β2 wild-type and mutant (**C**-**G**) systems. The RMSD was determined with respect to the initial binding mode of the agonists at the start of the simulations. Note that despite the changes in binding mode observed for some ligands, all agonists remained stably bound to their respective binding sites. Please zoom in on the image for detailed visualization.

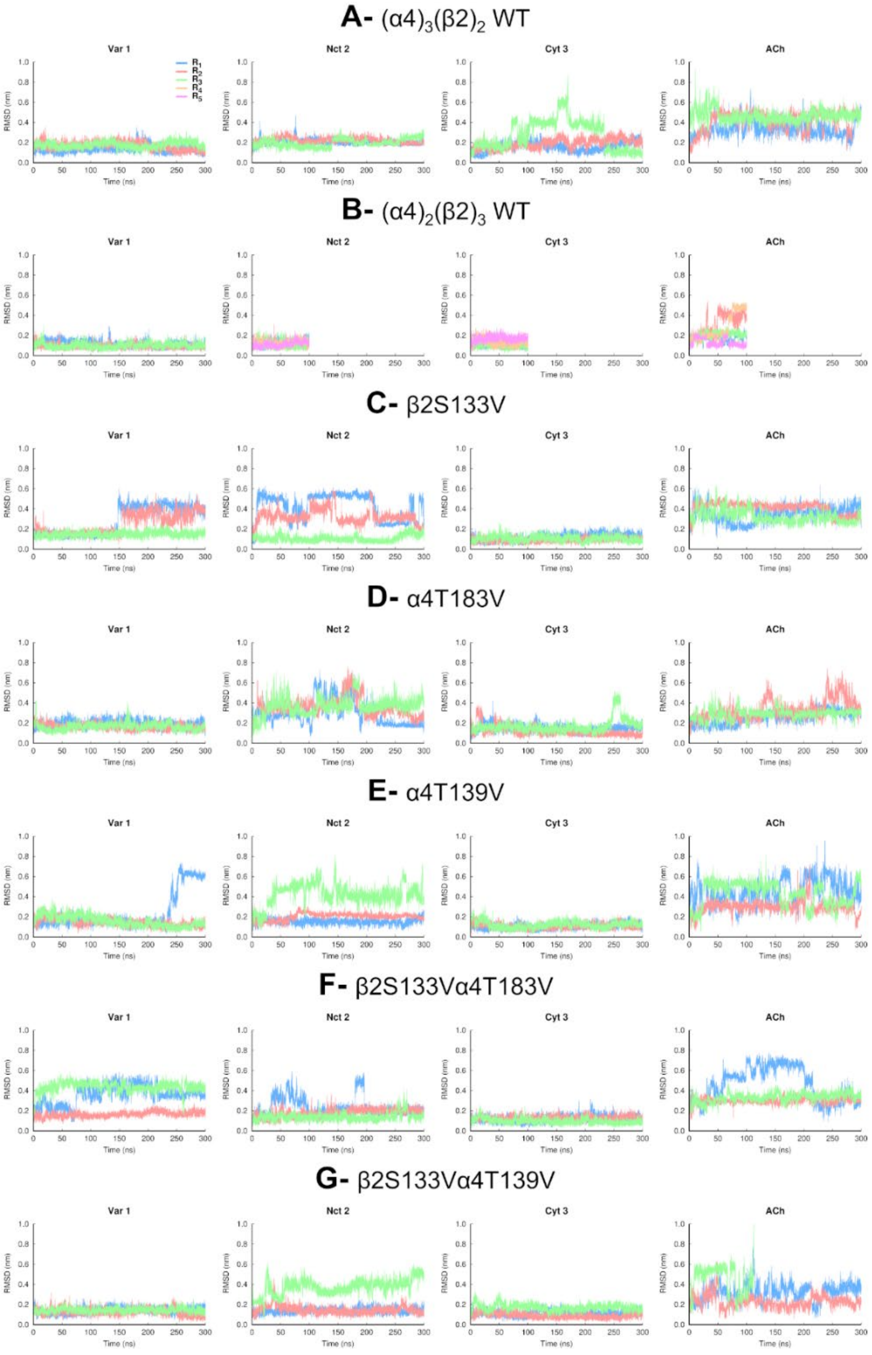

Figure S13- Temporal evolution of the RMSD for the agonists bound to the second α-β pocket in the LS (**A**) and HS (**B**) forms of the α4β2 wild-type and mutant (**C**-**G**) systems. The RMSD was determined with respect to the initial binding mode of the agonists at the start of the simulations. Note that all agonists remained stably bound to their respective binding sites, except for the ACh molecule in the second α-β pocket for replicate 3 in the β2S133Vα4T139V-ACh complex, which exited the pocket after 112 ns (as indicated by the green line in the rightmost plot of panel G). Please zoom in on the image for detailed visualization.

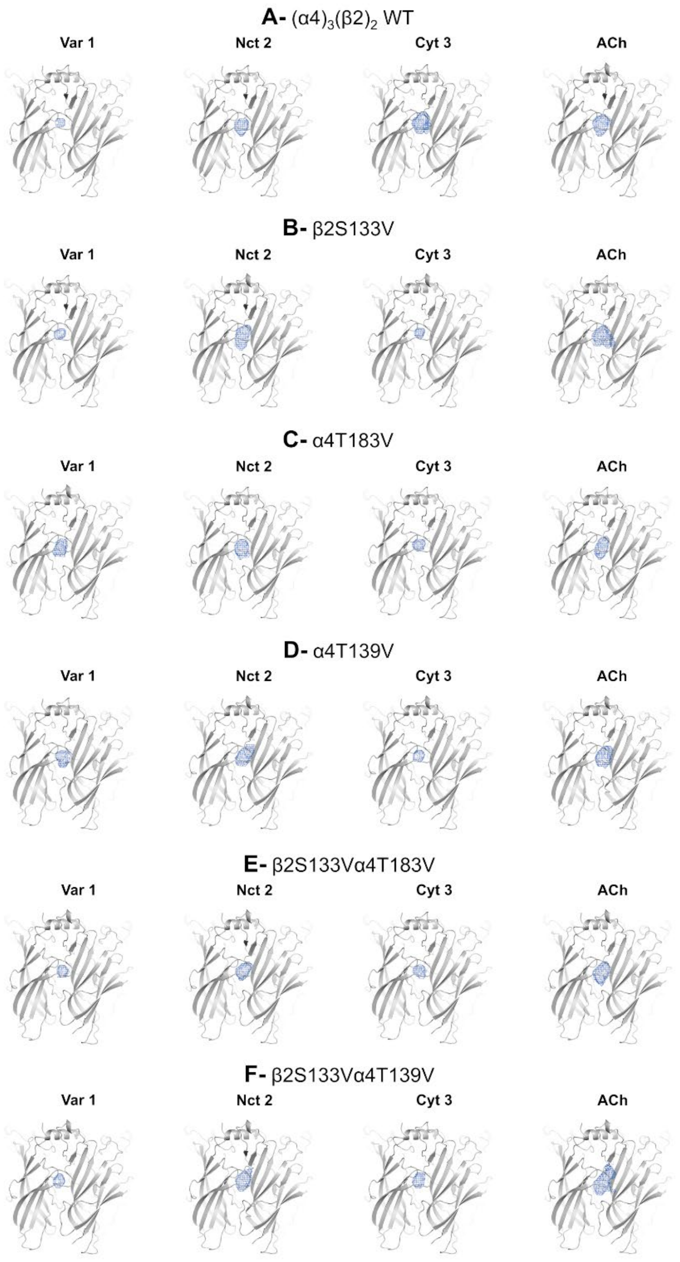

Figure S14- Probability density maps for the agonists bound to the α-α binding pocket in the wild-type (**A**) and mutant (**B-F**) LS isoform simulations. The contours at 0.00001 $Å^{-3}$ for the protonated nitrogen atoms of the agonist are depicted as a blue mesh. For all systems, the maps were calculated by combining the entire trajectories for each one of the three replicates of that system. Please zoom in on the image for detailed visualization.

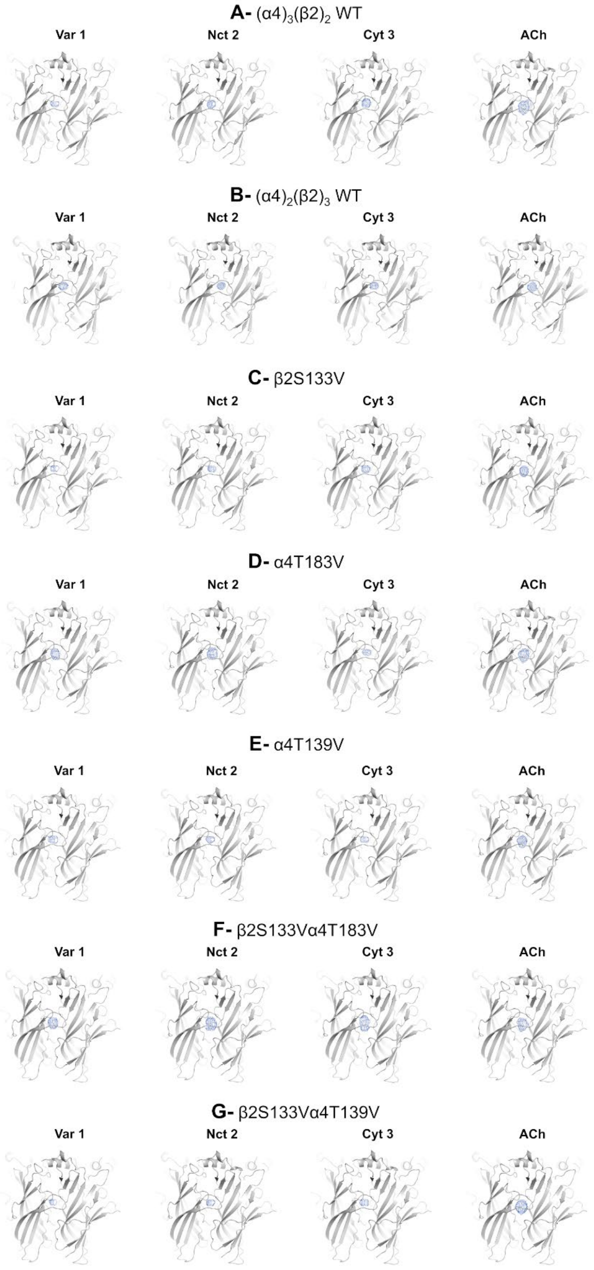

Figure S15- Probability density maps for the agonists bound to the first α-β binding pocket in the LS and HS isoforms of the α4β2 wild-type (**A-B**) and mutant (**C-G**) simulations. The contours at 0.00001 $Å^{-3}$ for the protonated nitrogen atoms of the agonist are depicted as a blue mesh. For all systems, the maps were calculated by combining the entire trajectories for each one of the three replicates of that system. Please zoom in on the image for detailed visualization.

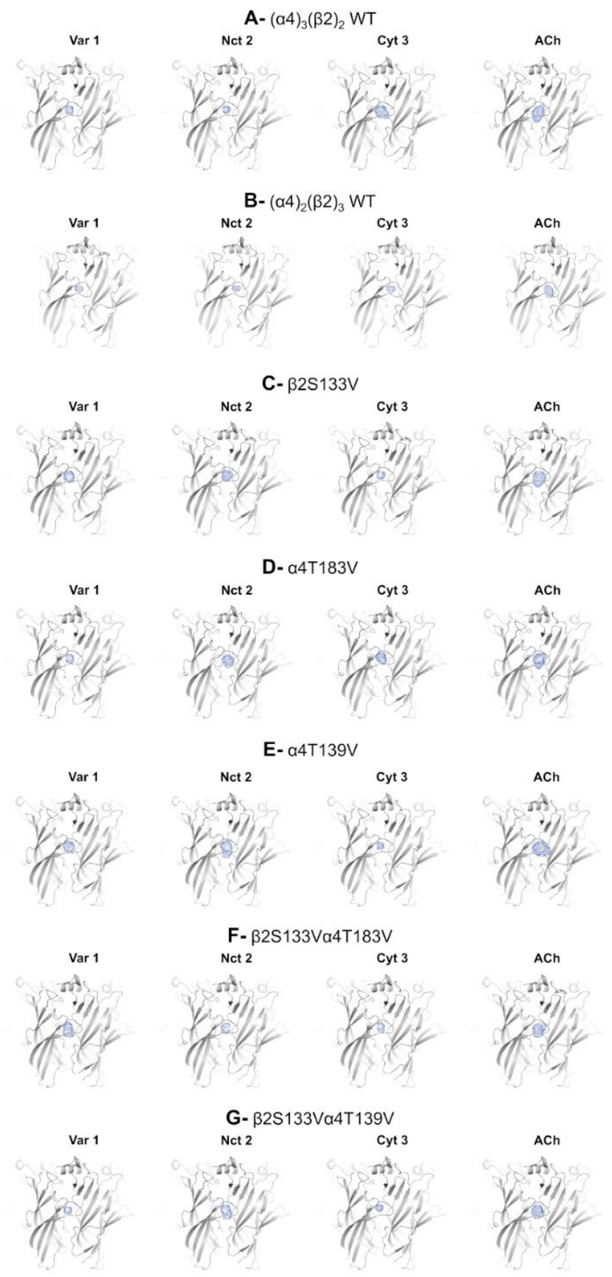

Figure S16- Probability density maps for the agonists bound to the second α-β binding pocket in the LS and HS isoforms of the α4β2 wild-type (**A-B**) and mutant (**C-G**) simulations. The contours at 0.00001 $Å^{-3}$ for the protonated nitrogen atoms of the agonist are depicted as a blue mesh. For all systems, with the exception of β2S133Vα4T139V-ACh, the maps were calculated by combining the entire trajectories for each one of the three replicates of that system. For the β2S133Vα4T139V-ACh complex, the map was obtained using the entire trajectory for replicates 1 and 2 and the first 112 ns for replicate 3 (as ACh exits the second α-β binding pocket after 112 ns of simulation). Please zoom in on the image for detailed visualization.

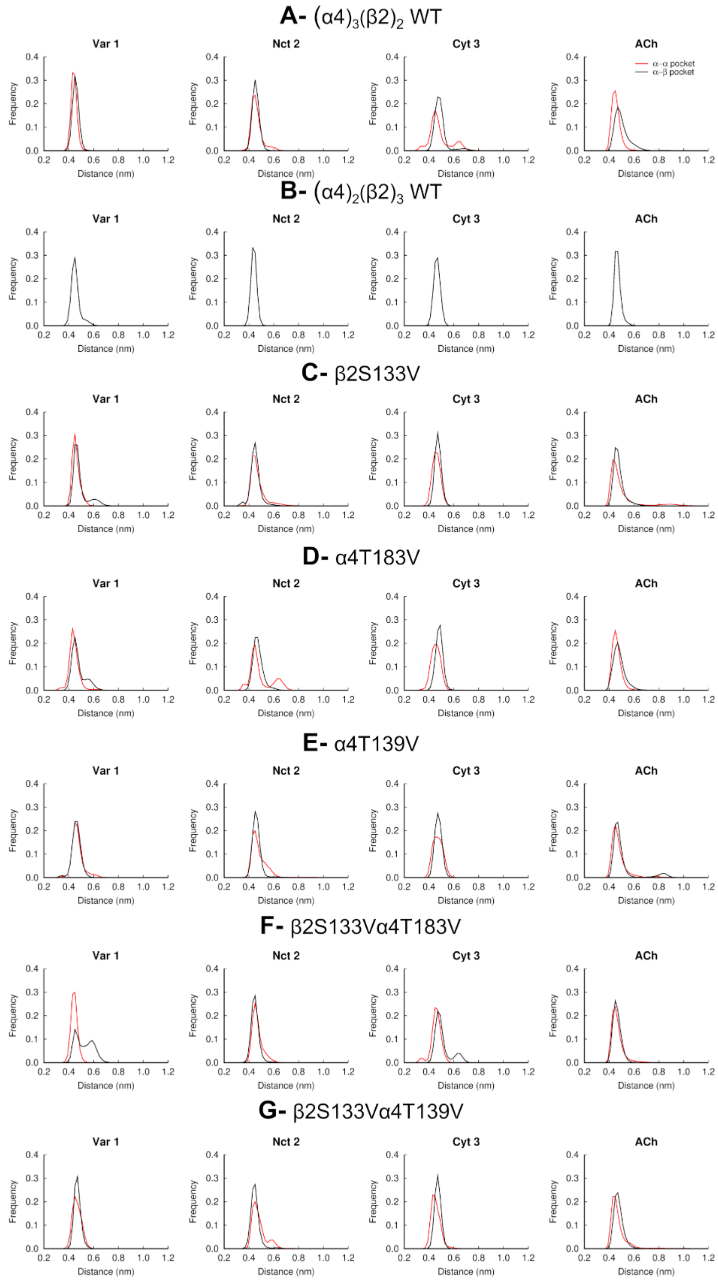

Figure S17- TrpB-agonist distance for the LS (**A**) and HS (**B**) isoforms of the α4β2 wild-type and mutant (**C-G**) systems. The distance between the side-chain of TrpB (W182 in the principal α4 subunit) and the protonated nitrogen atom of varenicline **1**, nicotine **2**, cytisine **3** and ACh for the α-α (red line) and α-β (black line) binding pockets is shown. The histogram for the α-β pocket reflects the distances over the two α-β binding pockets present in both the LS and HS isoforms of the α4β2 nAChR. Note that for the β2S133Vα4T139V–ACh complex, the histogram excludes the data for the second α–β pocket in replicate 3 from 112 ns onward, as the agonist exits the binding pocket during the simulation.

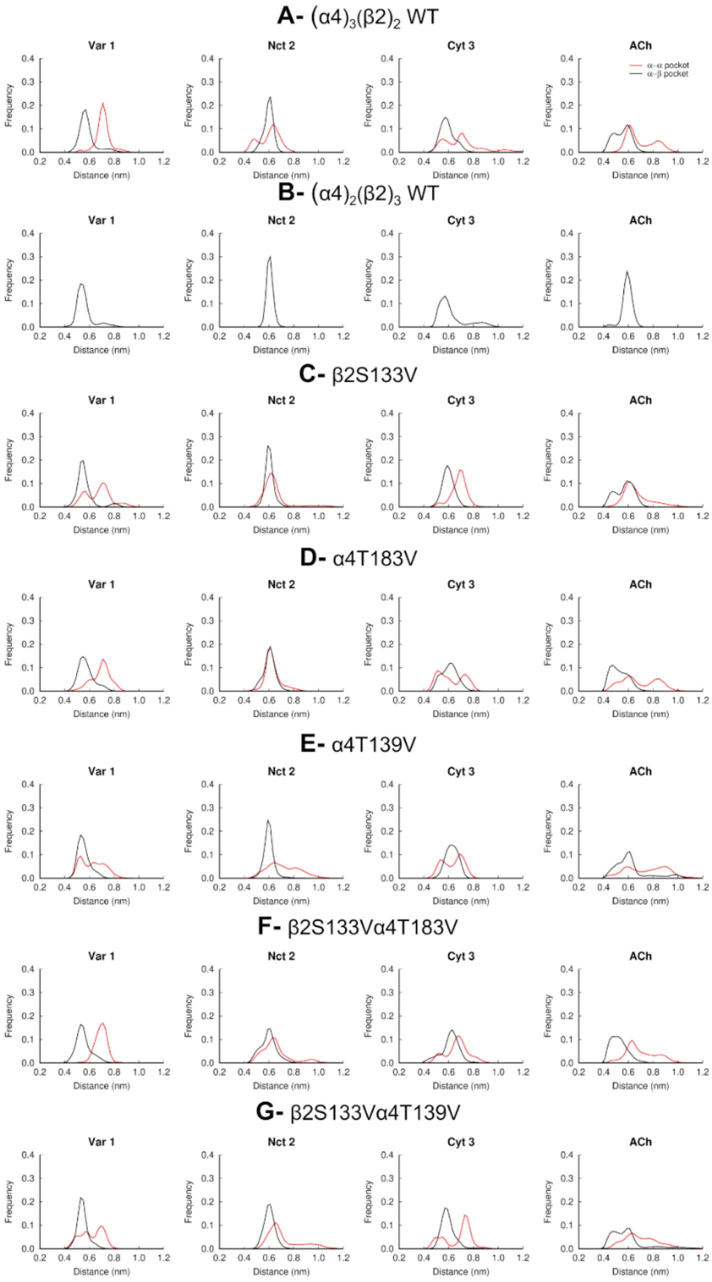

Figure S18- TyrA-agonist distance for the LS (**A**) and HS (**B**) isoforms of the α4β2 wild-type and mutant (**C-G**) systems. The distance between the side-chain of TyrA (Y126 in the principal α4 subunit) and the protonated nitrogen atom of varenicline **1**, nicotine **2**, cytisine **3** and ACh for the α-α (red line) and α-β (black line) binding pockets is shown. The histogram for the α-β pocket reflects the distances over the two α-β binding pockets present in both the LS and HS isoforms of the α4β2 nAChR. Note that for the β2S133Vα4T139V–ACh complex, the histogram excludes the data for the second α–β pocket in replicate 3 from 112 ns onward, as the agonist exits the binding pocket during the simulation.

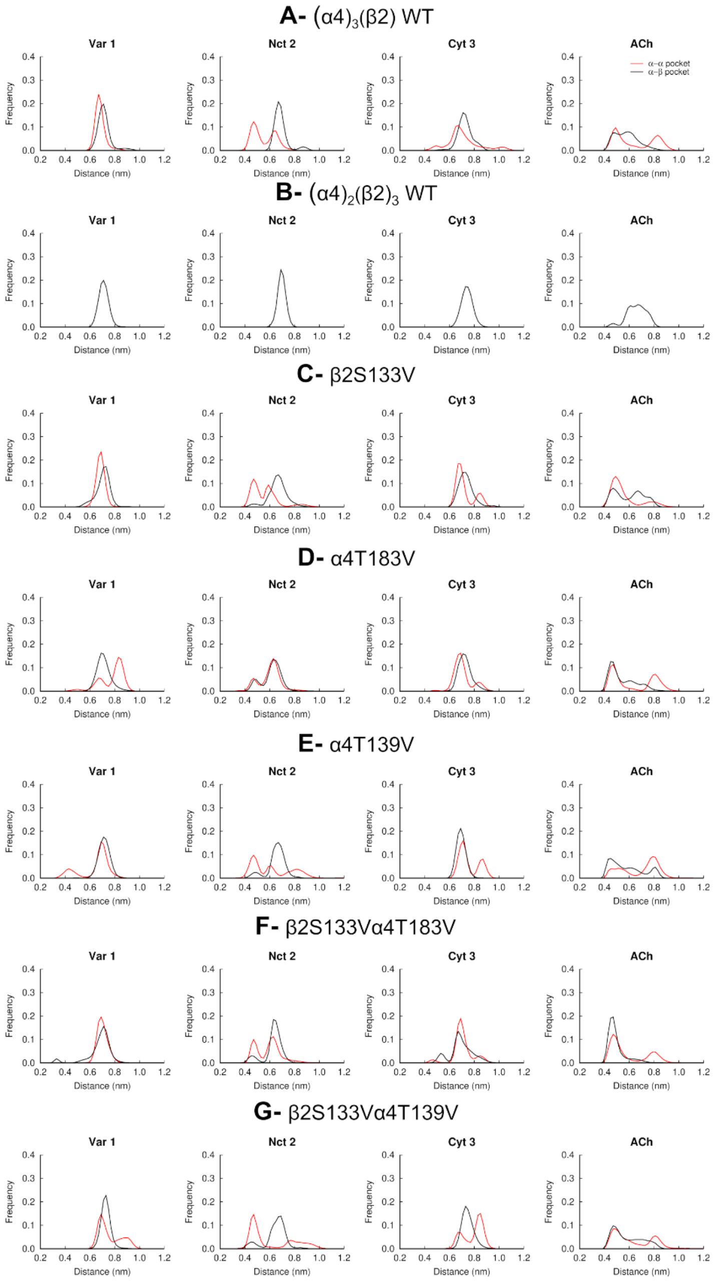

Figure S19- TrpD-agonist distance for the LS (**A**) and HS (**B**) isoforms of the α4β2 wild-type and mutant (**C-G**) systems. The distance between the side-chain of TrpD (W88 in the complementary α4 subunit of the α-α binding pocket and W82 in the complementary β2 subunit of the α-β binding pocket) and the protonated nitrogen atom of varenicline **1**, nicotine **2**, cytisine **3** and ACh for the α-α (red line) and α-β (black line) binding pockets. The histogram for the α-β pocket reflects the distances over the two α-β binding pockets present in both the LS and HS isoforms of the α4β2 nAChR. Note that for the β2S133Vα4T139V–ACh complex, the histogram excludes the data for the second α–β pocket in replicate 3 from 112 ns onward, as the agonist exits the binding pocket during the simulation.

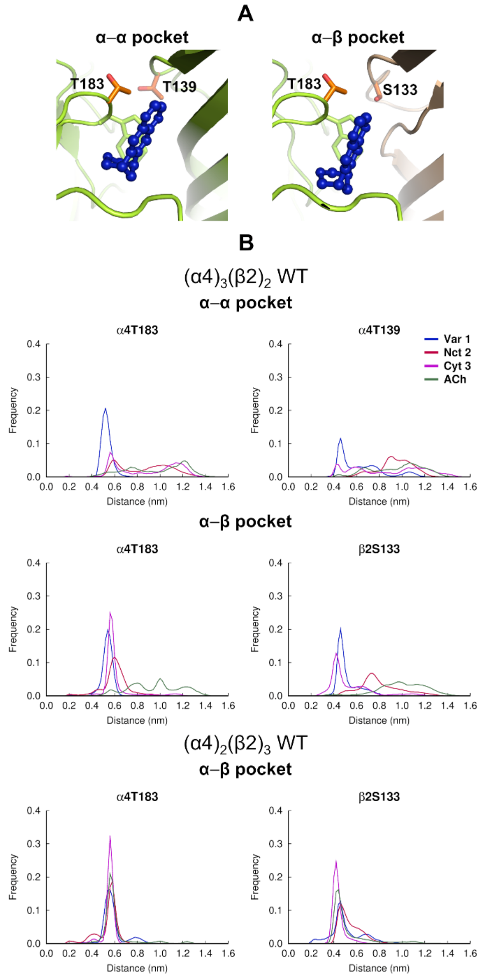

Figure S20- Agonist interactions with α4T183, α4T139 and β2S133 in the LS and HS isoforms of the α4β2 wild-type systems. (**A**) Location of α4T183 and α4T139 in the α-α pocket (left panel) and α4T183 and β2S133 in the α-β (right panel) binding site. The α4 and β2 subunits are colored in yellow and light brown, respectively. Varenicline **1** is highlighted in dark blue. The side-chains of α4T183, α4T139 and β2S133 are represented with orange sticks, whereas TrpB is shown with yellow sticks. Note that α4T183 is located in the principal α4 face of the pockets, whereas α4T139 and β2S133 are in the complementary face of the α-α and α-β pockets, respectively. (**B**) Distribution of the minimum distance between the agonist and α4T183, α4T139 and β2S133 in the α-α and α-β binding pockets of the LS and HS receptors. The reported values correspond to the minimum distances between the agonist (specifically, the closest pyrazine nitrogen in the quinoxaline moiety of varenicline **1**, the pyridine nitrogen of nicotine **2,** the pyridone carbonyl oxygen of cytisine **3** and the closest oxygen in the ester group of ACh), and the hydroxyl group of α4T183, α4T139 and β2S133 in all the MD trajectories for each complex. The histogram for the α-β pocket reflects the distances over the two α-β binding pockets present in the LS and HS isoforms of the α4β2 nAChR. These distance profiles indicate that some agonists can closely approach the H-bond donor groups in the side-chain of α4T183, α4T139, and β2S133, thus suggesting the possibility of transient interactions occurring between them.

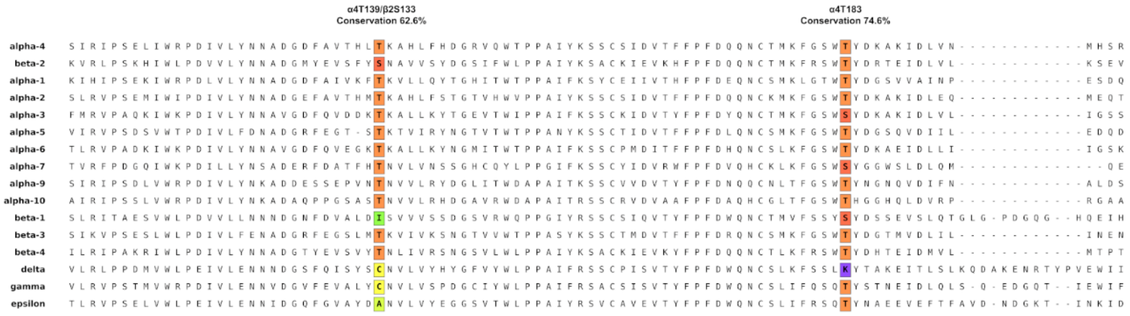

Figure S21- Sequence alignment for the human α1-α7, α9-α10, β1-β4, δ, γ, and ε nAChR subunits. The sequence alignments were performed using the Muscle server.[30] The colored boxes highlight the locations of α4T183, α4T139 and β2S133, with threonine, serine, isoleucine, cysteine, alanine, and lysine residues represented by orange, red, green, yellow, light green, and purple, respectively. Conservation percentages are expressed as 100×(1−H/H_{max}), where H is the Shannon entropy of the residue at the alignment position.[31] The sequences shown correspond to the following UniProt codes: P43681 (human α4), P17787 (human β2), P02708 (human α1), Q15822 (human α2), P32297 (human α3), P30532 (human α5), Q15825 (human α6), P36544 (human α7), Q9U6MI (human α9), Q9GZZ6 (human α10), P11230 (human β1), Q05901 (human β3), Q07001 (human δ), P07510 (human γ), and Q04844 (human ε).

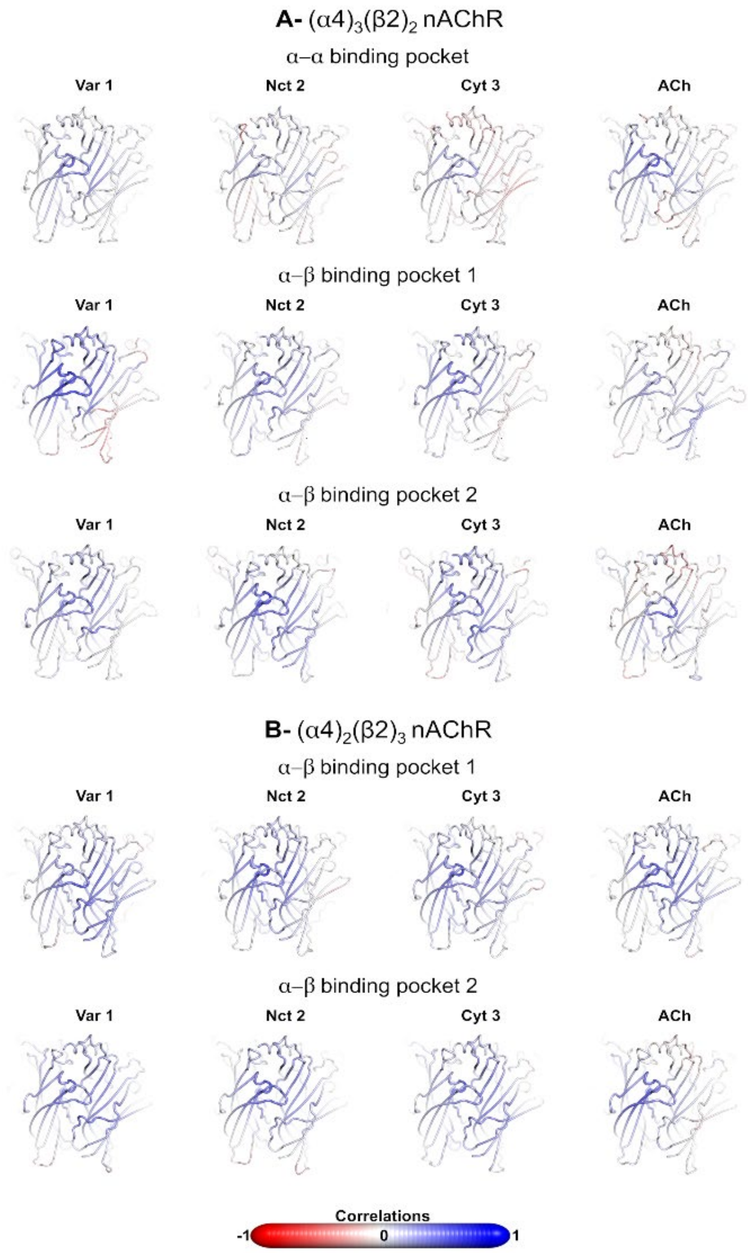

Figure S22- Statistical correlations for the different agonists when bound to ECD of the (**A**) LS and (**B**) HS isoforms of the α4β2 nAChR. Correlated motions for the agonist in the α-α and α-β binding pockets of the wild-type system. The correlations between the protonated nitrogen atom of the agonists and all the Cα atoms in the receptor are shown. Note that the atoms that systematically move in opposite directions have a correlation value of -1, whereas those systematically moving along the same direction show a correlation of 1. The atoms whose movements relative to the agonist are uncorrelated present a correlation value of 0. Please zoom in on the image for detailed visualization.

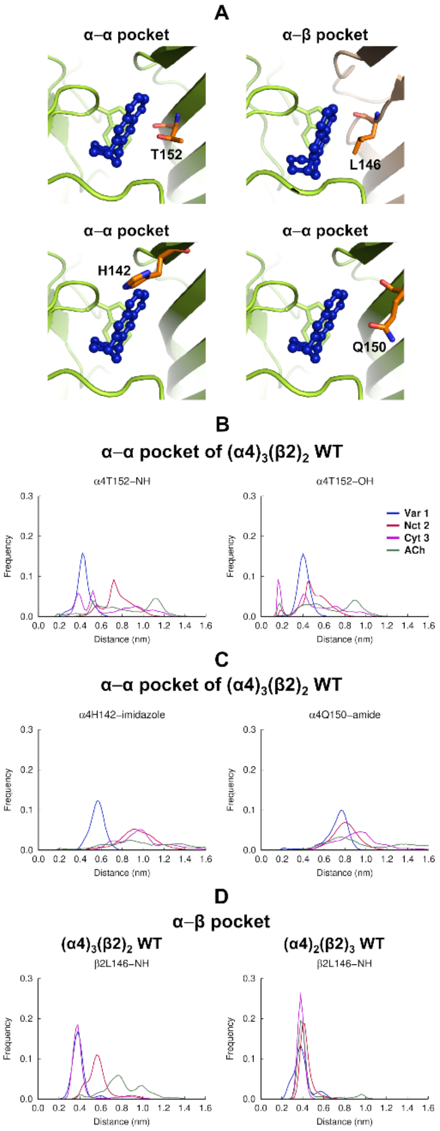

Figure S23- Interactions between the agonists and α4H142, α4Q150, α4T152, and β2L146 in the LS and HS isoforms of the α4β2 wild-type systems. (**A**) Location of α4H142, α4Q150 and α4T152 in the α-α pocket and β2L146 in the α-β pocket. The α4 and β2 subunits are colored in yellow and light brown, respectively. Varenicline **1** is highlighted in dark blue. α4H142, α4Q150, α4T152 and β2L146 are represented with orange sticks while TrpB is shown with yellow sticks. Note that α4H142, α4Q150 and α4T152 are located in the complementary face of the α-α pocket, whereas β2L146 is situated in the complementary side of the α-β pockets. (**B**) Distribution of the minimum distance between the agonist and the backbone NH and side-chain hydroxyl group of α4T152 in the α-α pocket of the LS isoform. The values reported correspond to the minimum distance between the agonist (namely, the closest pyrazine nitrogen in the quinoxaline group of varenicline **1**, the pyridine nitrogen of nicotine **2**, the pyridone carbonyl oxygen of cytisine **3**, and the closest oxygen in the ester group of ACh) and the NH

and OH group of α4T152 in all the MD trajectories for each complex. (**C**) Distribution of the minimum distance between the agonist and the H-bond donors in the side-chain of α4H142 and α4Q150 in the α-α pocket of the LS isoform. The values reported correspond to the minimum distance between the agonist (namely, the closest pyrazine nitrogen in the quinoxaline group of varenicline **1**, the pyridine nitrogen of nicotine **2**, the pyridone carbonyl oxygen of cytisine **3**, and the closest oxygen in the ester group of ACh) and the imidazole NH and amide NH2 group in the side-chain of α4H142 and α4Q150 in all the MD trajectories for each complex. (**D**) Distribution of the minimum distance between the agonist and the backbone NH of β2L146 in the α-β pockets of the LS (left panel) and HS (right panel) isoforms of the α4β2 receptor. The values reported correspond to the minimum distance between the agonist (namely, the closest pyrazine nitrogen in the quinoxaline group of varenicline **1**, the pyridine nitrogen of nicotine **2**, the pyridone carbonyl oxygen of cytisine **3**, and the closest oxygen in the ester group of ACh) and the NH group of β2L146 in all the MD trajectories for each complex. The histograms reflect the distances over the two α-β binding pockets present in the LS and HS isoforms of the α4β2 nAChR. Note that the distance profiles above indicate that some agonists can directly interact with the α4T152 hydroxyl donor in the α-α pocket.

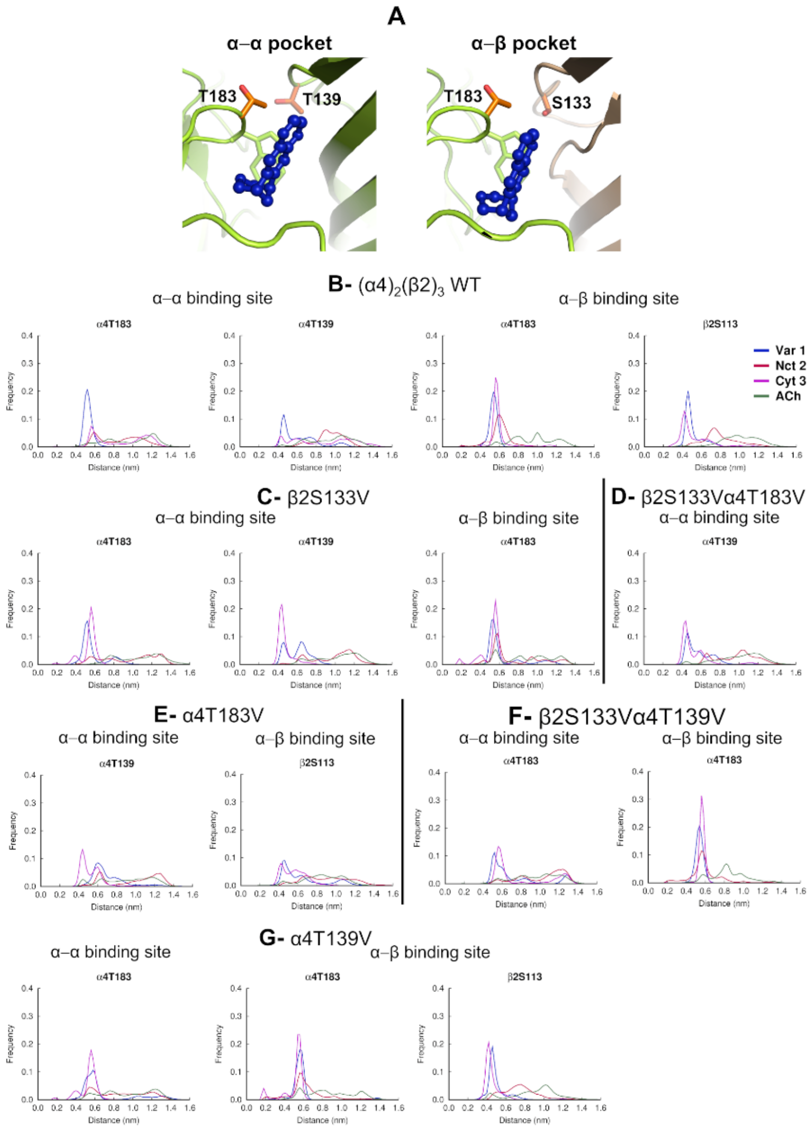

Figure S24- Agonist interactions with α4T183, α4T139 and β2S133 in the LS isoform of the wild-type and mutant systems. (**A**) Location of α4T183, α4T139 and β2S133 in the α-α (left panel) and α-β (right panel) binding pockets of the LS wild-type receptor. The α4 and β2 subunits are colored in yellow and light brown, respectively. Varenicline **1** is highlighted in dark blue. The side-chains of α4T183, α4T139 and β2S133 are represented with orange sticks, whereas TrpB is shown with yellow sticks. (**B-G**) Distribution of the minimum distance between the agonist and α4T183, α4T139 and β2S133 in the α-α and α-β binding pockets of the wild-type and β2S133V, α4T183V, α4T139V, β2S133Vα4T183V and β2S133Vα4T139V mutants. The reported values correspond to the minimum distances between the agonist (specifically, the closest pyrazine nitrogen in the quinoxaline group of varenicline **1**, the pyridine nitrogen of nicotine **2**, the pyridone carbonyl oxygen of cytisine **3**, and the closest oxygen in the ester group of ACh) and the hydroxyl group of α4T183, α4T139 and β2S133 in the MD trajectories for each complex. The histogram for the α-β pocket reflects the distances over the two α-β binding pockets present in the LS isoform of the α4β2 nAChR. Note that the substitution of α4T183, α4T139,

and β2S133 by valine (which lacks the hydroxyl group in its side-chain) prevents potential hydrogen bonding with the agonists. These distance profiles indicate that, as anticipated, the mutations altered the interaction patterns between the H-bond donors in the receptor binding sites and the agonists' acceptor groups.

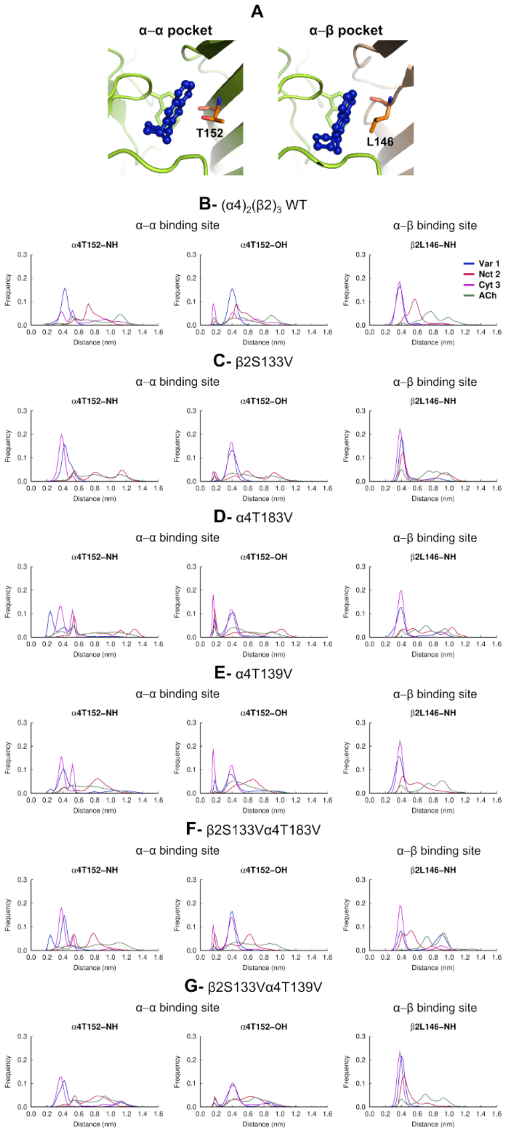

Figure S25- Agonist interactions with α4T152 and β2L146 in the LS isoform of the wild-type and mutant systems. (**A**) Location of α4T152 and β2L146 in the α-α (left panel) and α-β (right panel) binding pockets of the LS wild-type receptor. The α4 and β2 subunits are colored in yellow and light brown, respectively. Varenicline **1** is highlighted in dark blue. The side-chains of α4T152 and β2L146 are represented with orange sticks, whereas TrpB is shown with yellow sticks. (**B-G**) Distribution of the minimum distance between the agonist and α4T152 and β2L146 in the α-α and α-β binding pockets of the wild-type (**B**) and β2S133V (**C**), α4T183V (**D**), α4T139V (**E**), β2S133Vα4T183V (**F**) and β2S133Vα4T139V (**G**) mutants. The reported values correspond to the minimum distances between the agonist (specifically, the

closest pyrazine nitrogen in the quinoxaline group of varenicline **1**, the pyridine nitrogen of nicotine **2**, the pyridone carbonyl oxygen of cytisine **3**, and the closest oxygen in the ester group of ACh) and the backbone NH of α4T152 and β2L146 and the side-chain OH of α4T152 in the MD trajectories for each complex. The histogram for the α-β pocket reflects the distances over the two α-β binding pockets present in the LS isoform of the α4β2 nAChR. Overall, these distance profiles indicate that the mutations mainly altered the interaction pattern between the agonists and α4T152 within the α-α pocket.

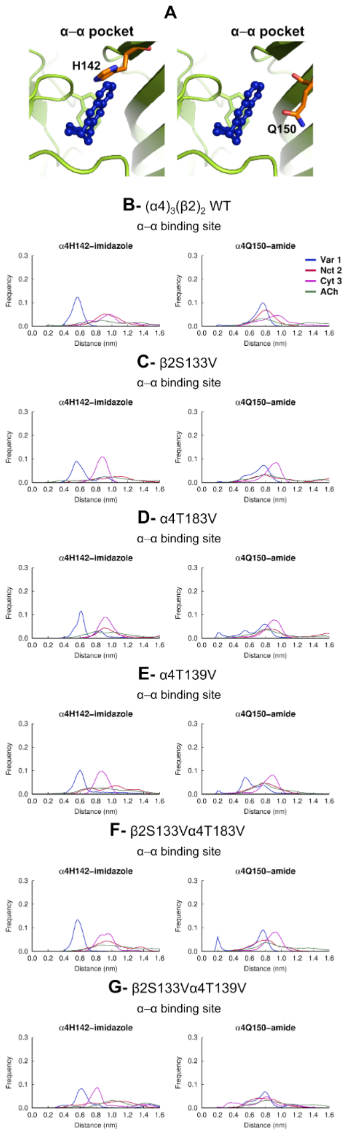

Figure S26- Agonist interactions with α4H142 and α4Q150 in the LS isoform of the wild-type and mutant systems. (**A**) Location of α4H142 (left panel) and α4Q150 (right panel) in the α-α binding pocket of the LS wild-type receptor. The α4 and β2 subunits are colored in yellow and light brown, respectively. Varenicline **1** is highlighted in dark blue. The side-chains of α4H142 and α4Q150 are represented with orange sticks, whereas TrpB is shown with yellow sticks. (**B-G**) Distribution of the minimum distance

between the agonists and α4H142 and α4Q150 in the α-α binding pocket of the wild-type (**B**) and β2S133V (**C**), α4T183V (**D**), α4T139V (**E**), β2S133Vα4T183V (**F**) and β2S133Vα4T139V (**G**) mutants. The reported values correspond to the minimum distances between the agonist (specifically, the closest pyrazine nitrogen in the quinoxaline group of varenicline **1**, the pyridine nitrogen of nicotine **2**, the pyridone carbonyl oxygen of cytisine **3**, and the closest oxygen in the ester group of ACh) and the NH in the imidazole side-chain of α4H142 and the side-chain NH2 amide of α4Q150 in the MD trajectories for each complex. Overall, these distance profiles indicate that the mutations mainly altered the interaction patterns between the agonists and α4Q150 within the α-α pocket.

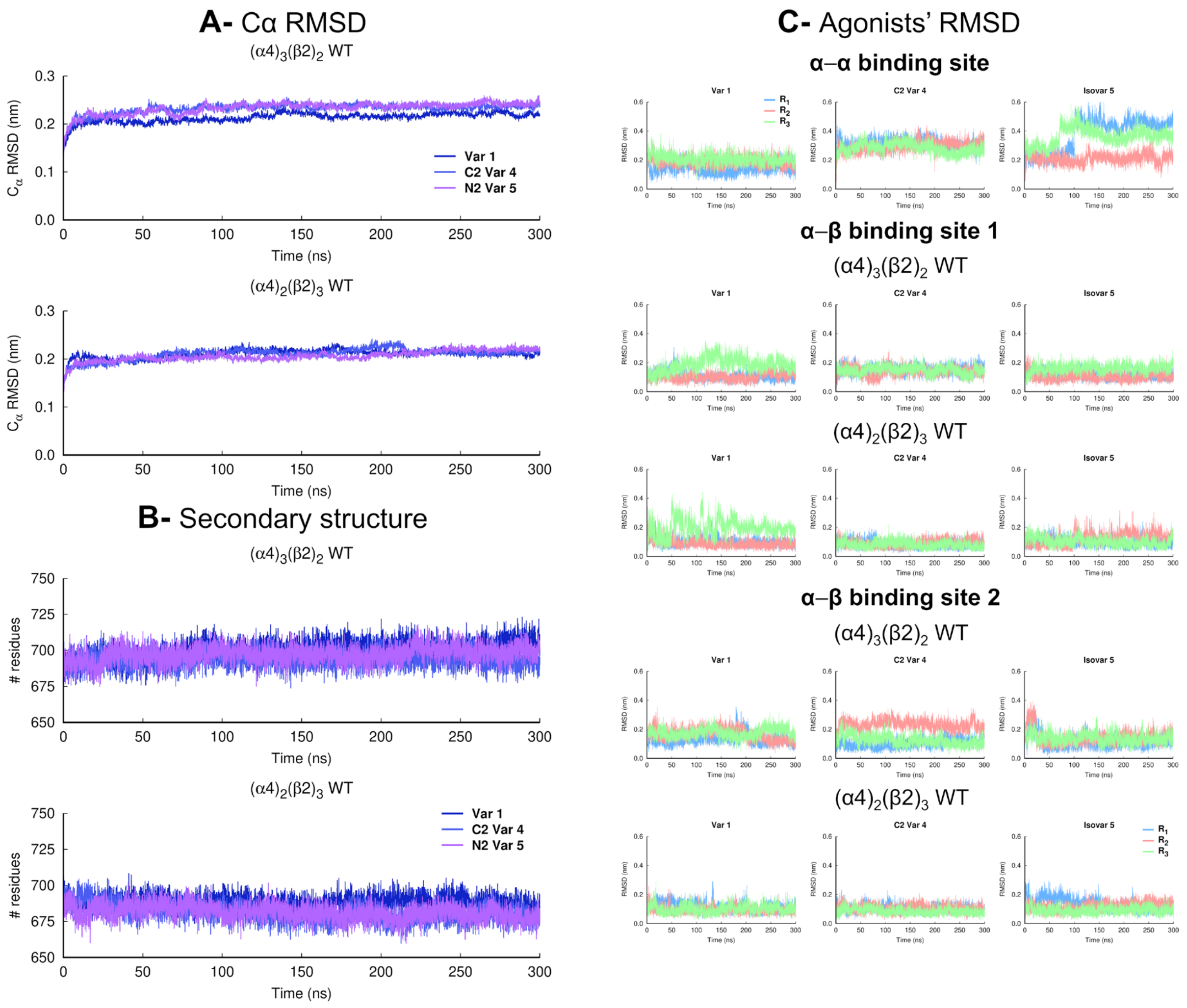

Figure S27- (**A**) Temporal evolution of the average Cα RMSD for the varenicline **1,** C_2 varenicline **4** and isovarenicline **5** bound to the LS and HS isoforms of the α4β2 wild-type systems. The Cα RMSD was calculated relative to the starting structures, and the averages were obtained over all replicates for each system. (**B**) Time evolution of number of residues involved in secondary structure motifs for the varenicline **1**, C_2 varenicline **4** and isovarenicline **5** bound systems. The secondary structure assignment was performed with the DSSP software[22] and includes all residues assigned to α-helix, π-helix, 3_{10}-helix, 5-helix, β-sheet, β-strand and β-bridge secondary structure classes. The averages were obtained over all replicates for each system. (**C**) Time evolution of the RMSD for varenicline **1,** C_2 varenicline **4** and isovarenicline **5** when bound to the α-α and α-α binding pockets in the LS and HS isoforms of the wild-type system. The RMSD was determined with respect to the initial binding mode of the agonists at the start of the simulations. Note that the α-α binding pocket only exists in the LS isoform of the receptor. Please zoom in on the image for detailed visualization.

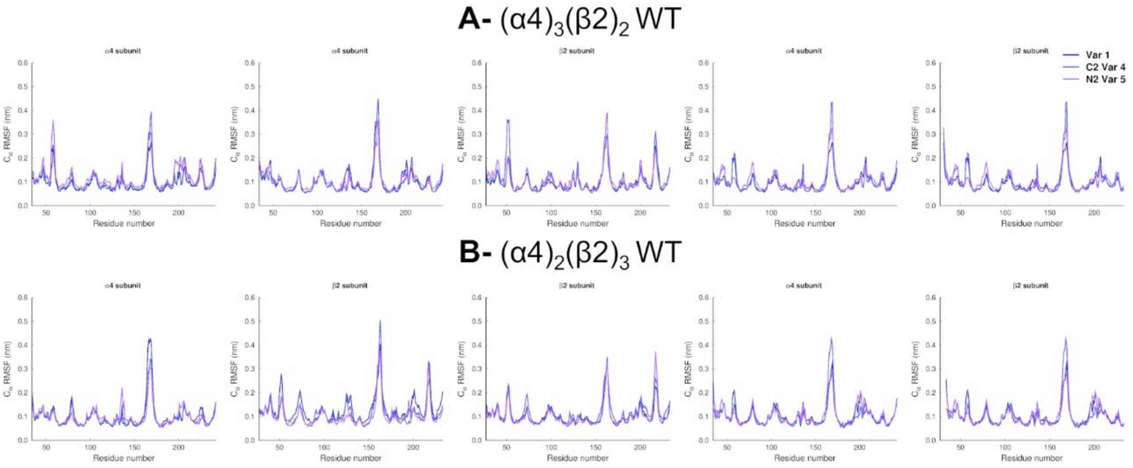

Figure S28- Average Cα RMSF for the LS (**A**) and HS (**B)** isoforms of the wild-type receptors with varenicline **1**, C_2 varenicline **4** and isovarenicline **5** bound. The Cα RMSF was calculated using the entire trajectories and averaged across all replicates for each complex. Please zoom in on the image for detailed visualization.

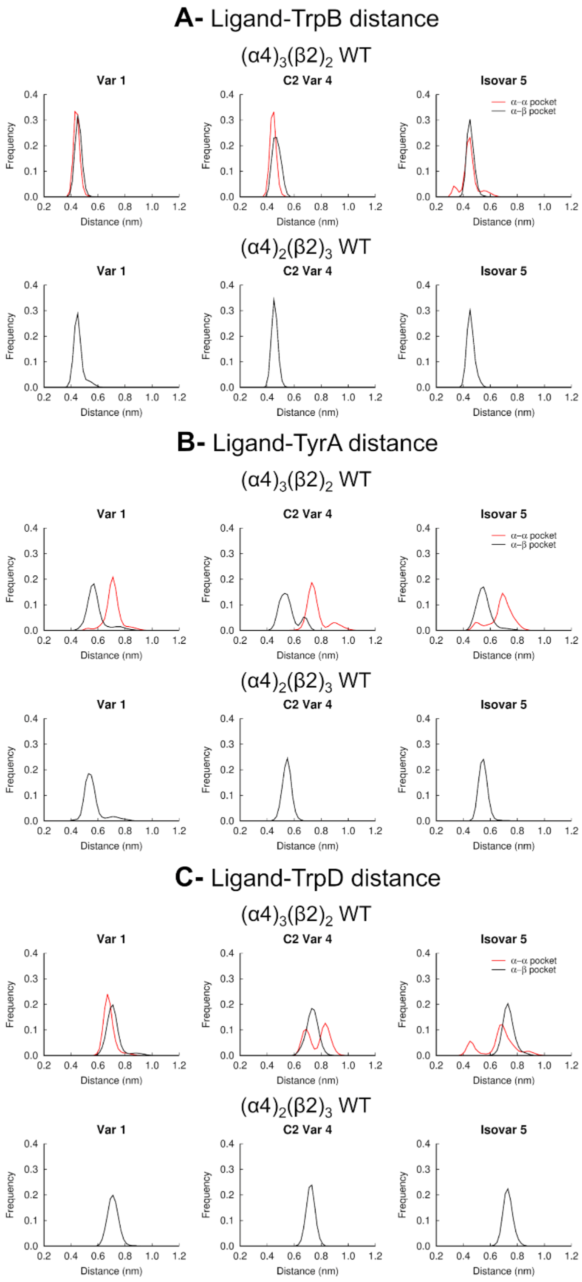

Figure S29- Distance profiles between varenicline **1**, C_2 varenicline **4** and isovarenicline **5** and TrpB **(A)**, TyrA (**B)** and TrpD (**C**) for the LS and HS isoforms of the α4β2 wild-type systems. Distance between the side chain of TrpB (W182 in the principal α4 subunit), TyrA (Y126 in the principal α4 subunit) and TrpD (W88 in the complementary α4 subunit of the α-α binding pocket and W82 in the complementary β2 subunit of the α-β binding pocket) and the protonated (piperidine) nitrogen atom of varenicline**1**, C_2 varenicline **4** and isovarenicline **5** for the α-α (red line) and α-β (black line) binding pockets is shown. The histogram for the α-β pocket reflects the distances over the two α-β binding pockets present in the LS and HS nAChRs.

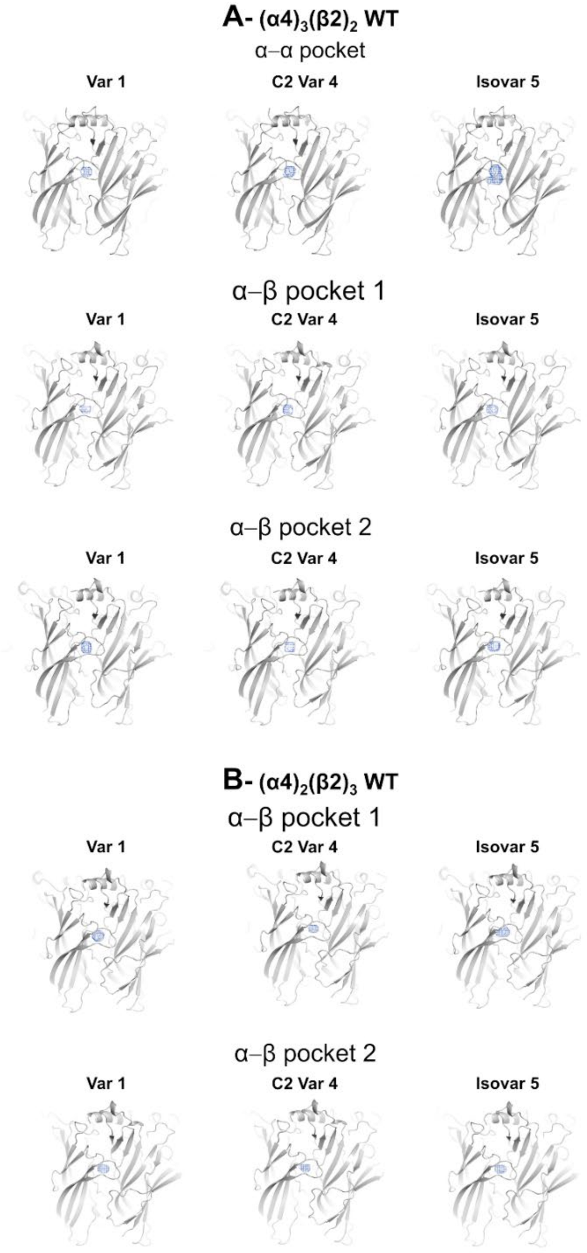

Figure S30- Probability density maps for varenicline **1**, C_2 varenicline **4** and isovarenicline **5** bound to the α-α and α-β binding pocket in the LS (**A**) and HS (**B**) isoforms of the α4β2 wild-type simulations. The contours at 0.00001 Å^{-3} for the protonated nitrogen atom of the agonists are depicted as a blue mesh. The maps were calculated by combining the entire trajectories (0-300 ns) for each one of the three replicates of that system. Please zoom in on the image for detailed visualization.

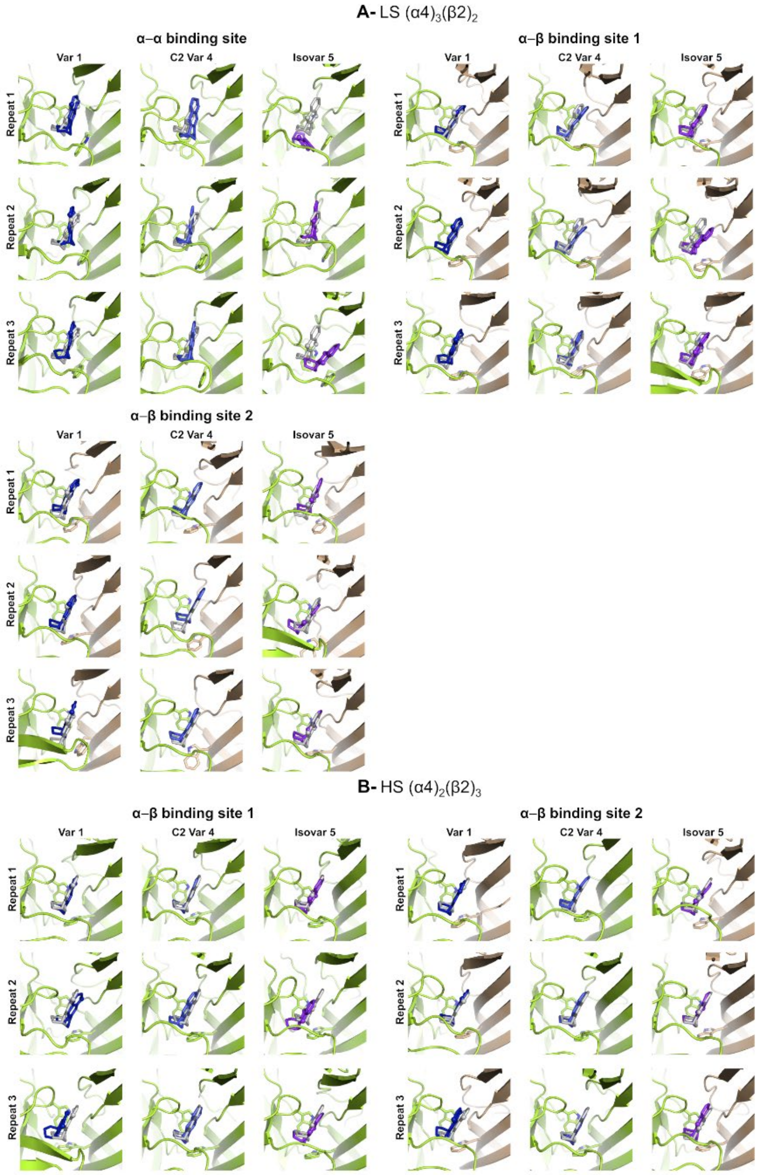

Figure S31- Binding mode of varenicline **1,** C_2 varenicline **4**, and isovarenicline **5** after 300 ns of simulation for the wild-type α4β2 complexes. (**A**) Binding mode of varenicline **1**, C_2 varenicline **4** and isovarenicline **5** in the α-α and α-β pockets of the wild-type LS nAChR. (**B**) Binding mode of varenicline **1**, C_2 varenicline **4** and isovarenicline **5** in the α-β pockets of the wild-type HS nAChR. The α4 and β2 subunits are colored in yellow and light brown, respectively. Agonists are depicted in blue, with the nitrogen atoms of the quinoxaline moiety (which serve as H-bond acceptors) highlighted by spheres. Please note that C_2 varenicline **4** contains a naphthalene residue instead of a quinoxaline unit, and thus lacks the nitrogen atoms needed to form hydrogen bonds. The grey sticks represent the starting binding mode for the agonists. TrpB and TrpD are shown with sticks. Please zoom in on the image for detailed visualization.

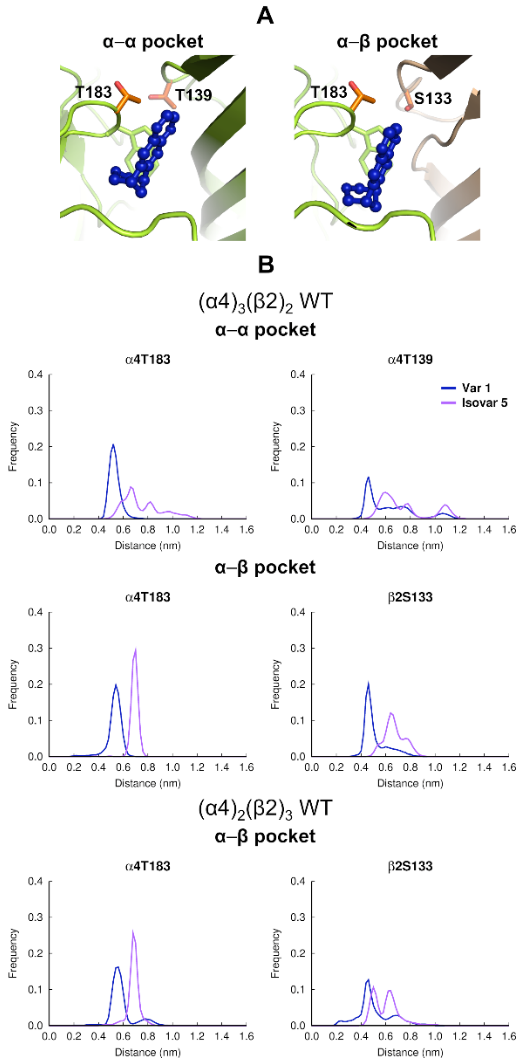

Figure S32- Varenicline **1** and isovarenicline **5** interactions with α4T183, α4T139 and β2S133 in the LS and HS isoforms of the α4β2 wild-type systems. (**A**) Location of α4T183, α4T139 and β2S133 in the α-α (left panel) and α-β (right panel) binding pockets. The α4 and β2 subunits are colored in yellow and light brown, respectively. Varenicline **1** is highlighted in dark blue. The side-chains of α4T183, α4T139 and β2S133 are represented with orange sticks, whereas TrpB is shown with yellow sticks. (**B**) Distribution of the minimum distance between the closest pyrazine nitrogen in the quinoxaline group of varenicline **1** and isovarenicline **5** and α4T183, α4T139 and β2S133 in the α-α and α-β binding pockets of the LS $(\alpha4)_3(\beta2)_2$ and HS $(\alpha4)_2(\beta2)_3$ receptor in all the MD trajectories for each complex. Note that C_2 varenicline **4**, instead of a quinoxaline unit, possesses a naphthalene group, and is therefore unable to form hydrogen bonds. The histogram for the α-β pocket reflects the distances over the two α-β binding pockets present in the LS and HS of the α4β2 nAChR. These distance profiles clearly demonstrate that, as expected, the distance between the hydrogen acceptor group in isovarenicline **5** and the α4T183/α4T139/β2S133 side-chain OH group is too large, thereby preventing any direct interaction between the two.

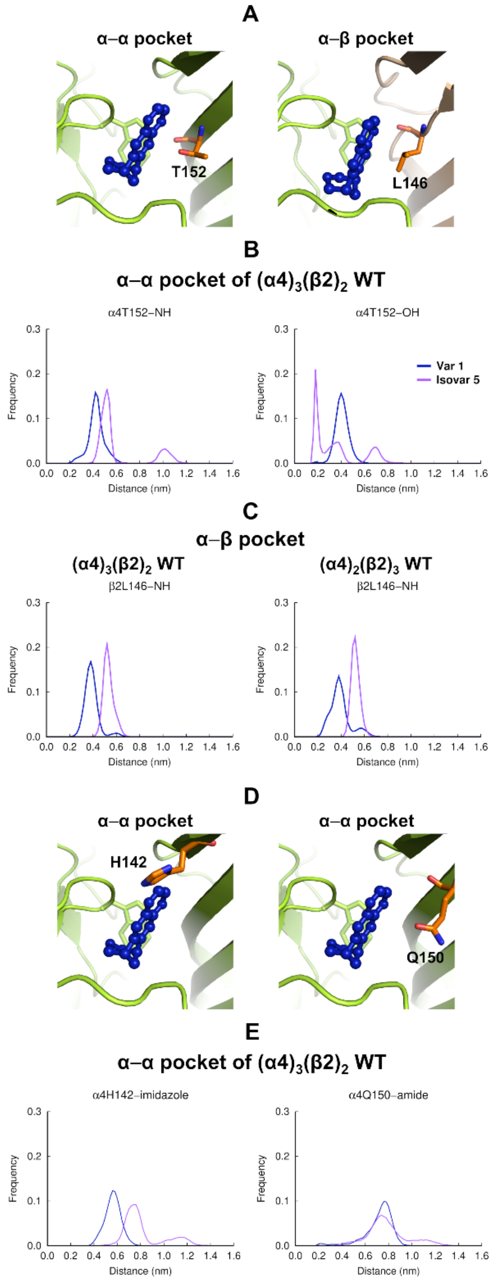

Figure S33- Varenicline **1** and isovarenicline **5** interactions with α4T152 and β2L146 in the LS and HS isoforms of the α4β2 wild-type systems. (**A**) Location of α4T152 and β2L146 in the α-α (left panel) and α-β (right panel) binding pockets of the $(\alpha4)_3(\beta2)_2$ wild-type receptor. See caption of Figure S32 for more details. (**B**) Distribution of the minimum distance between the closest pyrazine nitrogen in the quinoxaline group of varenicline **1** and isovarenicline **5** and the backbone NH and side-chain OH of α4T152 in the α-α pocket of the LS isoform of α4β2. Please note that **C_2** varenicline **4**, instead of a quinoxaline unit, possesses a naphthalene group, and is therefore unable to form hydrogen bonds. (**C**) Distribution of the minimum distance between the closest pyrazine nitrogen in the quinoxaline group of varenicline **1** and isovarenicline **5** and the backbone NH of β2L146 in the α-β pockets of the LS (left panel) and HS (right panel) forms of the receptor. The histograms reflect the distances over the two α-

β binding pockets present in the LS and HS isoforms of the α4β2 nAChR. Surprisingly, the distance profiles above show that isovarenicline **5** can make a persistent interaction with the α4T152 hydroxyl donor in the α-α pocket. (**D**) Location of α4H142 (left panel) and α4Q150 (right panel) in the α-α binding pockets of the LS wild-type receptor. See caption of Figure S32 for more details. (**E**) Distribution of the minimum distance between the closest pyrazine nitrogen in the quinoxaline group of varenicline **1** and isovarenicline **5** and the NH group in the imidazole side-chain of α4H142 (left side) and the NH2 in the amide side-chain of α4Q150 (right side) in the α-α pocket of the LS isoform. The distance profiles show that isovarenicline **5** is unable to directly H-bond to α4H142 and α4Q150.

Table S1- Summary of the simulations performed for the ECD of the human α4β2 nAChR. [a] The LS isoform corresponds to the $(\alpha4)_3(\beta2)_2$ receptor, whereas the HS to the $(\alpha4)_2(\beta2)_3$ one. [b] The simulations for the complexes between HS receptor with nicotine **2**, cytisine **3** and ACh were taken from our previous work.[7, 8]

Abbreviations used: varenicline **1** - **Var 1**; nicotine **2** - **Nct 2**; cytisine **3** - **Cyt 3**; acetylcholine - **ACh**; C_2 varenicline **4** - **C2 Var 4**; isovarenicline **5** - **Isovar 5**.

	**Isoform[a]**	**System**	**Agonist**	**Simulation length (ns)**	**Number of replicates**
1	LS	Wild type	**Var 1**	300	3
2	LS	Wild type	**Nct 2**	300	3
3	LS	Wild type	**Cyt 3**	300	3
4	LS	Wild type	**ACh**	300	3
5	LS	β2S133V	**Var 1**	300	3
6	LS	β2S133V	**Nct 2**	300	3
7	LS	β2S133V	**Cyt 3**	300	3
8	LS	β2S133V	**ACh**	300	3
9	LS	α4T183V	**Var 1**	300	3
10	LS	α4T183V	**Nct 2**	300	3
11	LS	α4T183V	**Cyt 3**	300	3
12	LS	α4T183V	**ACh**	300	3
13	LS	α4T139V	**Var 1**	300	3
14	LS	α4T139V	**Nct 2**	300	3
15	LS	α4T139V	**Cyt 3**	300	3
16	LS	α4T139V	**ACh**	300	3
17	LS	α4T183Vβ2S133V	**Var 1**	300	3
18	LS	α4T183Vβ2S133V	**Nct 2**	300	3
19	LS	α4T183Vβ2S133V	**Cyt 3**	300	3
20	LS	α4T183Vβ2S133V	**ACh**	300	3
21	LS	α4T139Vβ2S133V	**Var 1**	300	3
22	LS	α4T139Vβ2S133V	**Nct 2**	300	3
23	LS	α4T139Vβ2S133V	**Cyt 3**	300	3
24	LS	α4T139Vβ2S133V	**ACh**	300	3
25	HS	Wild type	**Var 1**	300	3
26[b]	HS	Wild type	**Nct 2**	100	5
27[b]	HS	Wild type	**Cyt 3**	100	5
28[b]	HS	Wild type	**ACh**	100	5
29	HS	Wild type	**C2 Var 4**	300	3
30	HS	Wild type	**Isovar 5**	300	3
31	HS	Wild type	**C2 Var 4**	300	3
32	HS	Wild type	**Isovar 5**	300	3

C. nAChR Ligand Binding Measurements

(i) Expression of human α4β2, α3β4 and α7 nAChR

Heterologously expressed α4β2 and α3β4 nAChR. HEK 293 cells were grown in Dulbecco's modified Eagle medium supplemented with 10% fetal bovine serum (FBS), 1% L-glutamine, 100 units/mL penicillin G and 100 µg/mL streptomycin in a humidified atmosphere containing 10% CO_2. cDNAs encoding human α3 and β4 or α4 and β2 were transfected into the HEK 293 cells at 30% confluency.

Heterologously expressed α7 nAChR. The SH-SY5Y cells were grown in RPMI medium (Lanza) supplemented with 10% fetal bovine serum (FBS), 1% of penicillin-streptomycin and 1% of L-glutamine. cDNA encoding human α7 was transfected into the SH-SY5Y cells at 30% confluency. The cells were maintained in an environment of 37°C containing 5% CO_2. The cell transfections were carried out in 100 mm Petri dishes using 30 mL of JetPEI™ (Polypus, France) (1 mg/mL, pH 7.2) and 10 µg of cDNAs. After 48 h transfection, the cells were collected, washed with PBS by centrifugation and frozen or used for binding analysis.

(ii) Radioligand binding assays

(±)-[^{3}H]Epibatidine (specific activity of 56-60 Ci/mmol) and [^{125}I]α-bungarotoxin (α-Bgtx) (specific activity of 200-213 Ci/mmol) were purchased from Perkin Elmer (Boston MA). Non-radioactive α-Bgtx, nicotine and epibatidine were purchased from Sigma-Aldrich.

[^{3}H]Epibatidine binding. Details of the binding experiments to the nicotinic subtypes have been previously reported by Tasso *et al.*[32] Saturation experiments were performed by incubating aliquots of membranes from HEK cells expressing α4β2 or α3β4 nAChR with 0.01-2.5 nM concentrations of (±)-[^{3}H]epibatidine overnight at 4°C. Nonspecific binding was determined in parallel by incubation in the presence of 100 nM unlabelled epibatidine. At the end of the incubation, the samples were filtered on GFC filters soaked in 0.5% polyethyleneimine and washed with 15 mL ice-cold phosphate buffered saline (PBS) and the filters were counted for radioactivity in a β counter. The affinity (K_d in nM) of [^{3}H]epibatidine for the α4β2 and α3β4 nAChR subtypes were 0.075, and 0.194 respectively and were derived from the average value of three independent [^{3}H]epibatidine binding saturation experiments.

[^{125}I]α-Bgtx binding. Saturation binding experiments were performed using membranes of α7-transfected SHSY5Y incubated overnight with 0.1-1.0 nM concentrations of [^{125}I] α–Bgtx at rt. Nonspecific binding was determined in parallel by incubation in the presence of 1 µM unlabelled α–Bgtx. After incubation, the samples were filtered as described above and the bound radioactivity was directly counted in a γ counter. Specific radioligand binding was defined as total binding minus the nonspecific binding determined in the presence of 1 µM unlabelled α–Bgtx. Nonspecific binding was ~20-30% of total binding. The K_d of [^{125}I] α–Bgtx for the α7 subtype was 1.2 nM and was derived from the average value of three independent [^{125}I]α–Bgtx binding saturation experiments.

(iii) Competition binding assays

The ability of varenicline variant ligands to compete for the agonist binding sites of α4β2, α3β4 or α7 nAChR was determined by inhibition of [^{3}H]epibatidine and [^{125}I] α–Bgtx binding. Membranes from cells transfected with the appropriate nAChR subtype were incubated with increasing concentrations of test compound for five minutes, followed by overnight incubation at 4 °C, with [^{3}H]epibatidine: 0.1 nM (for α4β2 nAChR) or 0.25 nM (for α3β4 nAChR), or at rt with [^{125}I] α–Bgtx: 2-3 nM (for α7 nAChR); radioligand concentrations approximate to their experimentally determined Kd values (see below). After incubation, the membranes were washed five times with ice-cold PBS. [^{3}H]epibatidine binding was determined by liquid scintillation counting in a β counter, and [^{125}I] α–Bgtx binding by means of direct counting in an γ counter.

(iv) Statistical analysis

Data from competition binding assays were evaluated by one-site competitive binding curve-fitting procedures using GraphPad Prism version 6 (GraphPad Software, Inc, CA, USA). Half maximal inhibition concentrations (IC_{50}) for varenicline variant ligands were obtained by fitting three independent competition binding experiments, each performed in duplicate for each compound on each subtype. Inhibition constants (K_i) were estimated by reference to the K_d of the radioligand, according to the Cheng-Prusoff equation.

In the saturation binding assay, the maximum specific binding (B_{max}) and the equilibrium binding constant (K_d) values were calculated using one site–specific binding with Hill slope – model.

D. nAChR Methods and Functional Studies

(i) Animals

Adult female *Xenopus laevis* were purchased from the European *Xenopus* Resource Center (Portsmouth, UK). *Xenopus laevis* toads were housed and cared for following the UK Home Office code of practice guidelines for the species. The collecting of oocytes from *Xenopus* toads was carried in a regulated room in the Biomedical Services facility in Oxford University, where the toads were housed.

(ii) Human α4β2 nAChR expression in *Xenopus* oocytes

Compounds reported were tested for effects on the function of human α4β2 nACh receptors expressed heterologously in *Xenopus* oocytes, which were isolated from adult female *Xenopus laevis* toads as previously described.[7] Human α4β2 receptors were expressed as either $(\alpha4)_3(\beta2)_2$ (low sensitivity for ACh) or $(\alpha4)_2(\beta2)_3$ (high sensitivity for ACh) receptors. Expressions in oocytes was obtained as follows. Human cDNA for α4 or β2 were subcloned into plasmid pCI from Promega and injected into the nucleus of *Xenopus* oocytes as described previously.[33] To express $(\alpha4)_3(\beta2)_2$ nACh receptors, a mixture of 10 α4 : 1 β2 cDNAs was injected into the nucleus of oocytes, whereas for $(\alpha4)_2(\beta2)_3$ receptors the cDNA ratio injected was 1 α4 : 10 β2.

(iii) Single and double mutations

Mutations were introduced in the α4 or β2 nAChR subunits using the Stratagene QuikChange Site-Directed Mutagenesis Kit (Agilent, UK). The presence of the mutation and the absence of unwanted mutations were confirmed by sequencing the entire cDNA insert (Eurofins, UK). Note that we present the numbering of the residues according to the full length of the following UniProt sequence codes for human α4 and β2 subunits, respectively: P43681 (α4 subunit) and P17787 (β2 subunit). To obtain the position in the mature form, subtract 28 from the number for α4 and 25 for β2 subunit.

(iv) Electrophysiological recordings

Electrophysiological recordings were performed 2-5 days post-injection, as previously described.[28] Current responses were obtained by two-electrode voltage-clamp recording at a holding potential of -60 mV using an Oocyte Clamp OC-725C amplifier (Warner Instruments, USA).

Concentration-response curves for agonists assayed were obtained by normalizing agonist-induced responses to the control ACh responses induced by 1 mM, a maximum effective ACh concentration at both α4β2 nAChR stoichiometries. A minimum interval of 5 minutes was allowed between agonist applications to ensure reproducible recordings. The agonist concentration-response relationship was characterized for data from each cell using non-linear regression in GraphPad (Prism 5, GraphPad, USA) by fitting the Hill equation ($Y([compound]) = Y_{max} (1 / (1 + (EC_{50}/[compound])^{\wedge}n^{Hill}))$, where Y is the response to a concentration of compound, Y_{max} is the maximal response, EC_{50} is the concentration producing half-maximal activation, and n^{Hill} is the Hill coefficient. Concentration-Response data were collected for an individual cell, and data were normalized to the response to 1 mM ACh. The fit was rejected if the estimated error in any fit parameter was greater than 60% of the fit value, and all parameter estimates for that fit were discarded. For compounds that elicited less than 10% of the maximal ACh response, the EC_{50} was not determined, and the relative efficacy was established by using the equation: maximal response to test compound/maximal ACh response. Data points represent the mean ± standard error of the mean (SEM) of 8-10 experiments carried out in at least three different batches of oocytes donors.

(v) **Statistical analysis**

For functional assays, the final data sets were assembled from a minimum of 5 independent recordings (i.e. n = 5) conducted on oocytes obtained from at least 5 different *Xenopus* donors. Data obtained from the same batch of oocytes were considered replicates. The data sets represent full concentration-response relationships obtained from individual oocytes (i.e., incomplete experiments were discarded). The data from each experiment were fitted separately and the estimated EC_{50} values were used to obtain the mean EC_{50} or IC_{50} (95% CI) reported in the manuscript or Supplementary Information. $LogEC_{50}$ values for agonist were analyzed using one-way ANOVA, followed by a post hoc Dunnett's test and/or a posthoc Bonferroni multiple comparison test to determine the level of significance between wild type

and mutant receptors. Prior to the ANOVA analysis, the data were tested for normality using the D'Agostino and Pearson normality test in PRISM and were normally distributed. Post hoc tests were run only if F achieved $P < 0.05$ and there was no significant variance in homogeneity.

(vi) Supporting figures and tables

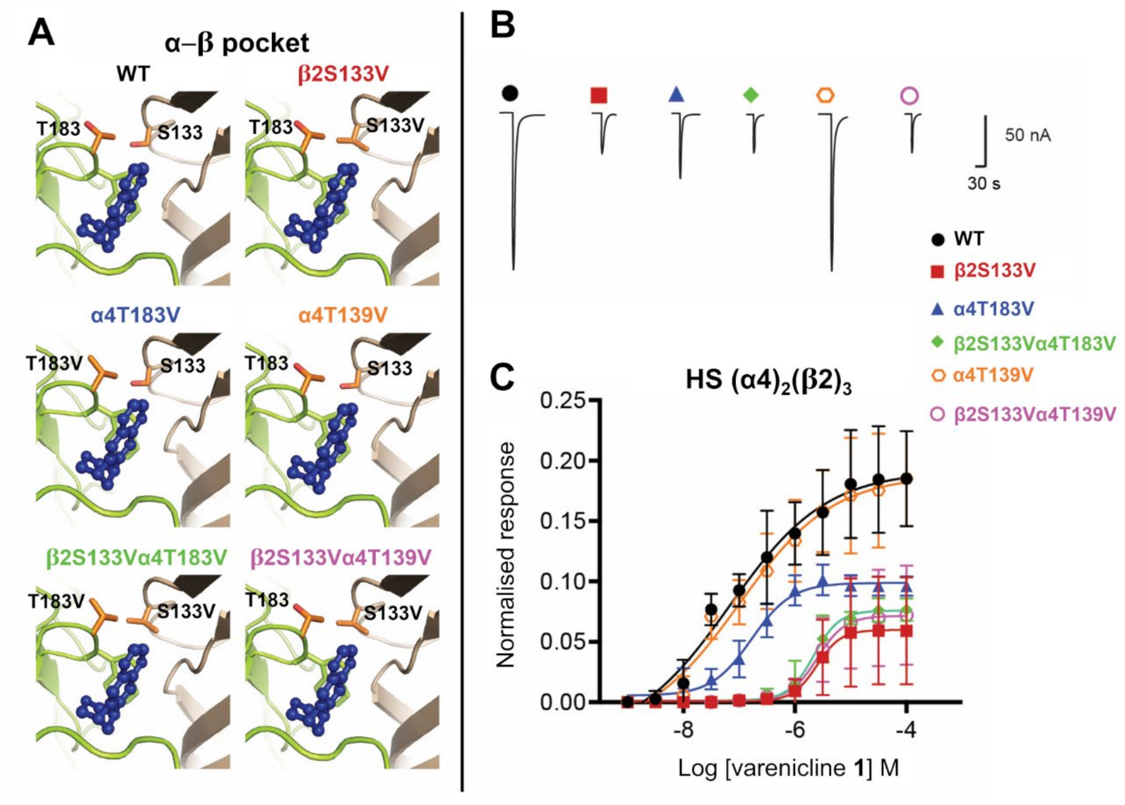

Figure S34- Effects of side-chain hydroxyl mutations on varenicline **1** agonism at the HS isoform of the α4β2 nAChR. (**A**) Location of α4T183 and β2S133 and corresponding mutations to valine in the α-β binding pockets of the wild type and mutants. The α4 and β2 subunits are colored in yellow and light brown, respectively. Varenicline **1** is highlighted in dark blue. The side-chains of α4T183 and β2S133 are represented with orange sticks, whereas TrpB is shown with yellow sticks. (**B**) Representative current traces elicited by maximal concentration of varenicline **1** (100 μM) applied to *Xenopus* oocytes expressing wild-type (WT) or mutant HS nAChR. Full concentration-responses curves are shown in panel **C**. (**C**) Concentration-response curves for varenicline **1** at wild-type (WT) and mutant HS nAChRs. Data points in the concentration-response curves represent the mean ± SEM of 8-10 experiments carried out using 6-8 different *Xenopus* donors. Current responses were measured using two-electrode voltage-clamping from *Xenopus* oocytes heterologously expressing wild type or mutant HS

isoform of the α4β2 nAChR. Peak current amplitudes for varenicline **1** were normalized to maximal ACh response (1 mM) and then fitted with the Hill equation, as described in the Materials and Methods section above. Estimated parameters EC_{50} and maximal relative efficacy (RE) are shown in Table 1 in the main text.

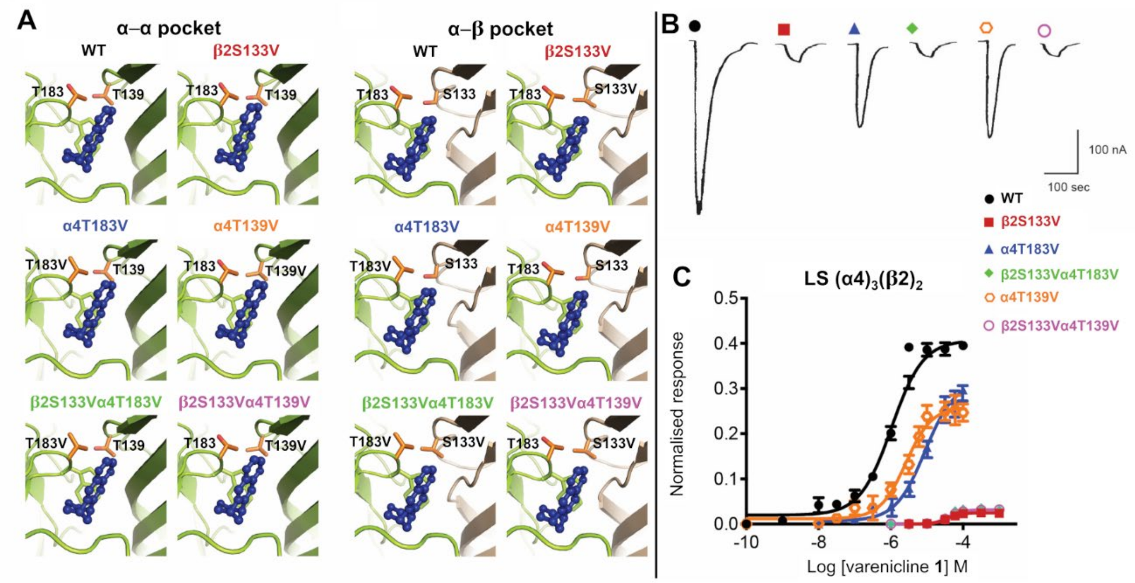

Figure S35- Effects of side-chain hydroxyl mutations on varenicline **1** agonism at the LS isoform of the α4β2 nAChR. (**A**) Location of α4T183, α4T139 and β2S133 and corresponding mutations to valine in the α-α and α-β binding pockets of the wild type and mutants. The α4 and β2 subunits are colored in yellow and light brown, respectively. Varenicline **1** is highlighted in dark blue. The side-chains of α4T183, α4T139 and β2S133 are represented with orange sticks, whereas TrpB is shown with yellow sticks. (**B**) Representative current traces elicited by maximal concentration of varenicline **1** (100 μM) applied to *Xenopus* oocytes expressing wild-type or mutant LS nAChR. Full concentration-responses curves are shown in panel **C**. (**C**) Concentration-response curves for varenicline **1** at wild-type (WT) and mutant LS isoform. Data points in the concentration-response curves represent the mean ± SEM of 8-10 experiments carried out using 6-8 different *Xenopus* donors. Current responses were measured using two-electrode voltage-clamping from *Xenopus* oocytes heterologously expressing WT or mutant LS isoform of the α4β2 nAChR. Peak current amplitudes for varenicline **1** were normalized to maximal ACh response (1 mM) and then fitted with the Hill equation, as described in the Materials and Methods section above. Estimated parameters EC_{50} and maximal relative efficacy (RE) are shown in Table 1 in the main text.

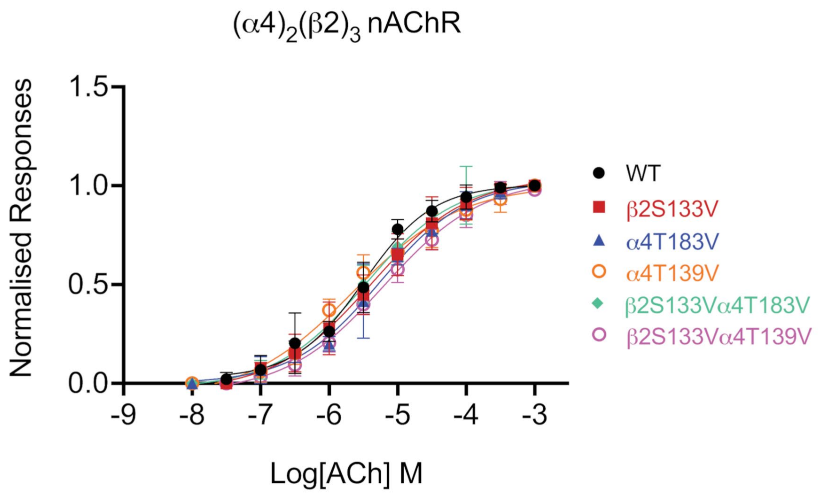

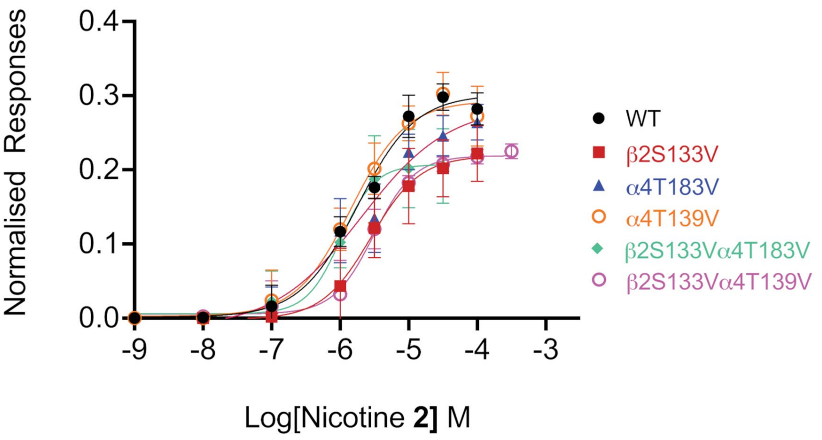

Figure S36- Effects of side-chain hydroxyl mutations on ACh or nicotine **2** agonism at the HS isoform of the α4β2 nAChR. Concentration-response curves for ACh and nicotine **2** at wild-type (WT) and mutant HS nAChRs. Data points in the concentration-response curves represent the mean ± SEM of 8-10 experiments carried out using 6-8 different *Xenopus* donors. Current responses were measured using two-electrode voltage-clamping from *Xenopus* oocytes heterologously expressing WT or mutant HS isoform of the α4β2 nAChR. Peak current amplitudes for ACh or nicotine **2** were normalized to maximal ACh response (1 mM) and then fitted with the Hill equation, as described in the Materials and Methods section above. Estimated parameters EC_{50} and maximal relative efficacy (RE) are shown in Table 1 in the main text.

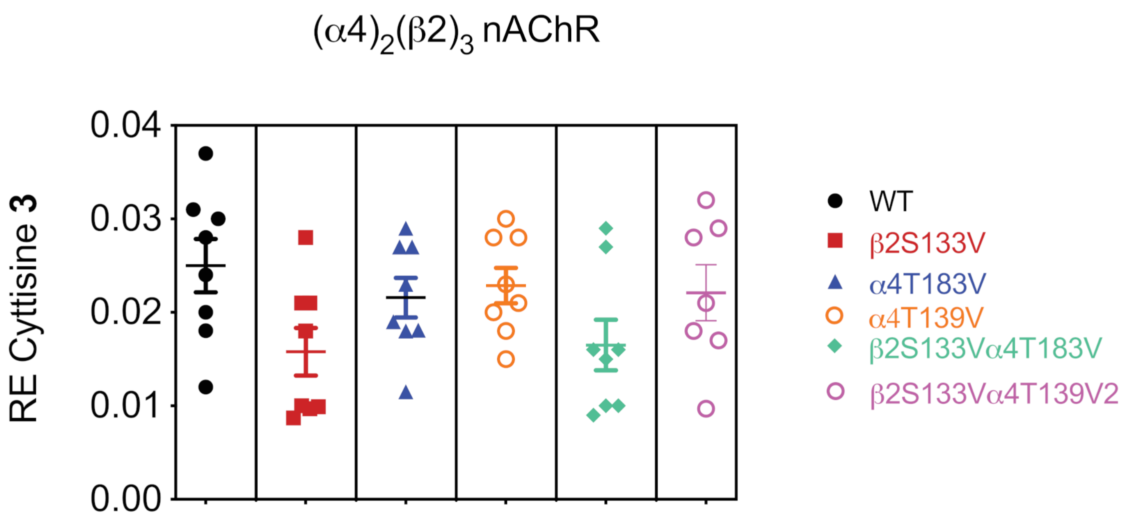

Figure S37- Relative efficacy of cytisine **3** at wild-type (WT) or mutant HS isoform of the α4β2 nAChR. Cytisine **3** displays poor agonist efficacy at $(\alpha4)_2(\beta2)_3$ nAChRs, making it difficult to generate concentration-response curves. To assess the functional effects of side-chain hydroxy mutations on cytisine agonism, we estimated the relative efficacy of cytisine using the equation $I_{max}/I_{max}ACh$. Oocytes were challenged with increasing concentrations of cytisine until the responses reached a plateau, which was considered as the maximal current response to the agonist. Data are shown as a box and whisker plot. Current responses from wild-type (WT) or mutant receptors were measured using two-electrode clamping, as described in the Materials and Methods section above. Statistical comparisons between WT and mutant receptors were performed using One Way ANOVA followed by a post hoc Dunnett's and/or Bonbeferroni multiple comparison tests. The estimated means ± SEM are shown in Table 1 in the main text.

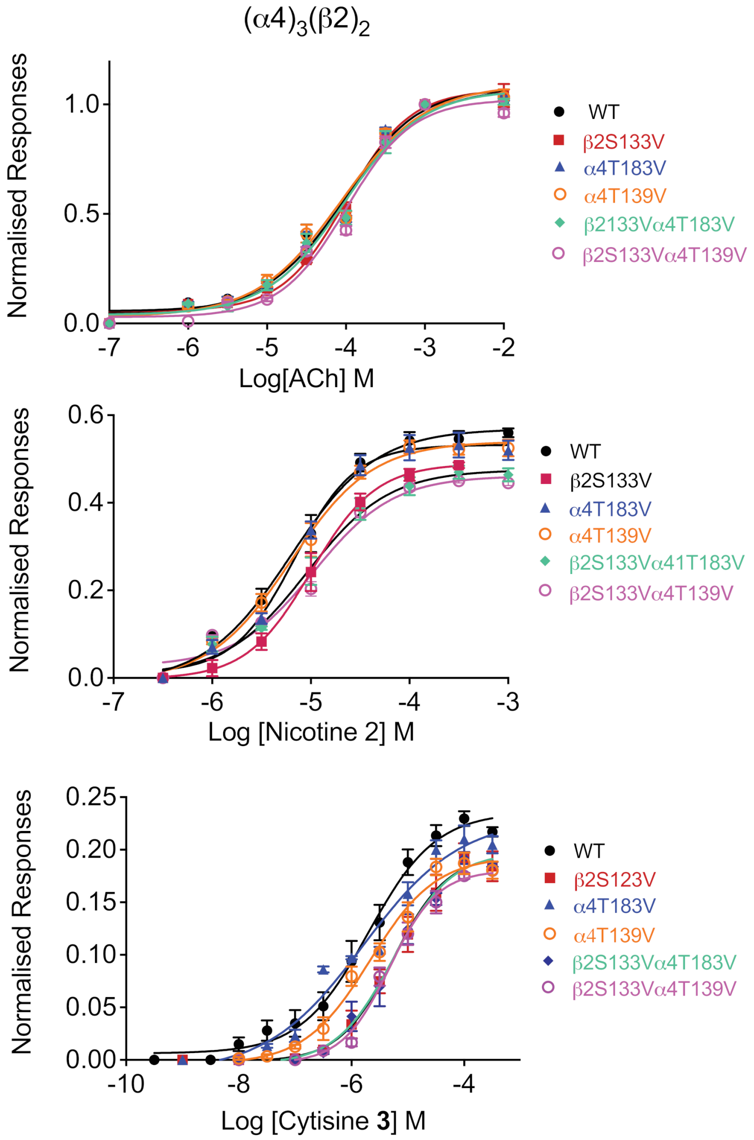

Figure S38- Effects of side-chain hydroxyl mutations on agonist sensitivity of the LS isoform of the α4β2 nAChR. Concentration-response curves for ACh, nicotine **2** and cytisine **3** were obtained at wild type (WT) or mutant $(\alpha4)_3(\beta2)_2$ nAChRs. Data points in the concentration-response curves represent the mean ± SEM of 8-10 experiments carried out using 6-8 different *Xenopus* donors. Current responses were measured using two-electrode voltage-clamping from *Xenopus* oocytes heterologously expressing WT or mutant $(\alpha4)_3(\beta2)_2$ nAChRs. Peak current amplitudes for all agonists tested ACh were normalized to maximal ACh responses (1 mM) prior fitting the data with the Hill equation, as described in the Materials and Methods section above. Estimated parameters EC_{50} and maximal relative efficacy (RE) are shown in Table 1 in the main text.

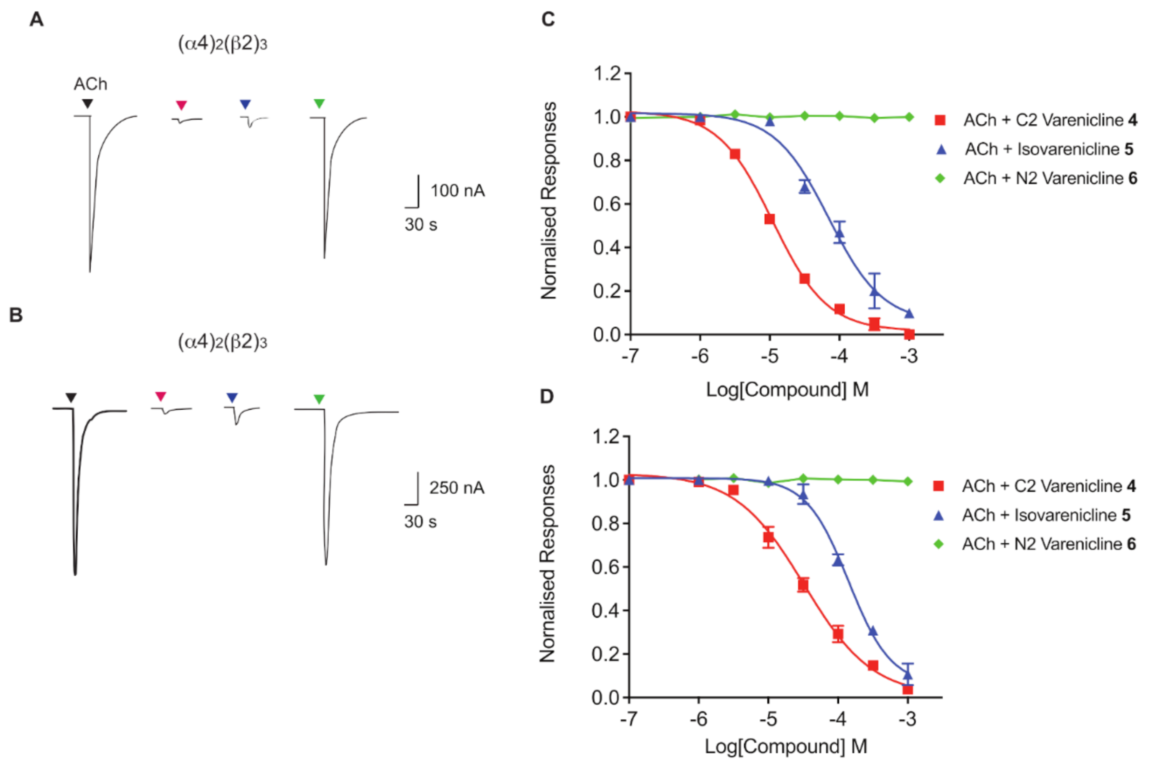

Figure S39- Inhibition of α4β2 by varenicline **1**, C_2 varenicline **4**, isovarenicline **5** and N_2 varenicline **6** on the α4β2 nAChR. C_2 varenicline **4** and isovarenicline **5** behave as partial agonist at α4β2 nAChRs and, as such, should be able to inhibit the responses to ACh. To obtain concentration-response curves data for the inhibitory effects of the varenicline ligands on the alternate forms of the α4β2 nAChR, the compounds (1 nM to 1 mM range) were co-applied with ACh EC_{80}: 30 µM for HS and 300 µM for LS isoforms of the α4β2 receptor. The peak of the current responses obtained in this manner were then normalized to the peak of the responses elicited by ACh EC_{80} alone. The normalized data were then fit by non-linear regression to the Hill equation, as described in the Materials and Methods section above. As shown in the concentration response curves for C_2 varenicline **4** and isovarenicline **5**, these ligands inhibited the responses to ACh in a concentration-dependent manner. In contrast, N_2 varenicline **6**, which had no agonist effect at α4β2 nAChR, did not inhibit the responses to ACh, indicating that this ligand does not bind the agonist sites present on the HS or LS isoform of the α4β2 nAChR.

Table S2- IC_{50} values for the inhibition of α4β2 nAChR by varenicline variants **4**-**6**. The IC_{50} values shown were estimated non-linearly from the concentration-response data shown above, as described in the part (*iv*) of this section above. NE, no effect. [a] The HS isoform corresponds to the $(\alpha4)_2(\beta2)_3$ receptor. [b] The LS isoform corresponds to the $(\alpha4)_3(\beta2)_2$ receptor.

**Ligand**	**IC_{50} at HS isoform[a]**	**IC_{50} at LS isoform[b]**
C_2 varenicline **4**	11±1.2	33±14
Isovarenicline **5**	70±12	142±21
N_2 varenicline **6**	NE	NE

E. 5-HT_3 Methods and Functional Studies

(i) Cell culture

Human embryonic kidney (HEK) 293 cells (ATCC, Teddington, UK) were maintained on 90 mm tissue culture plates at 37 °C and 7% CO_2 in a humidified atmosphere. They were cultured in DMEM:F12 (Dulbecco's Modified Eagle Medium / Nutrient Mix F12 (1:1)) with GlutaMAX™ (Thermo Fischer Scientific, Paisley, UK), containing 10% HyClone fetal calf serum (GE Healthcare, Hatfield, UK). For radioligand binding studies, cells in 90 mm dishes were transfected with pcDNA3.1 (Thermo Fischer Scientific, Paisley, UK), containing the 5-HT_3AR subunit (Uniprot id P46098) using electroporation. For functional studies, cells were transfected as above and then plated in 96-well plates. Cells were incubated 1-3 days before assay.

(ii) Radioligand binding

This was undertaken as previously described in Price *et al.*.[34] Briefly, transfected HEK293 cell membranes were incubated in 0.5 ml HEPES buffer containing the 5-HT_3 receptor antagonist [^{3}H]GR65630 (0.3 nM; Perkin Elmer, Beaconsfield, UK). Non-specific binding was determined using 1 µM quipazine (Tocris Bioscience, Bristol, UK).

(iii) FlexStation analysis

This technique uses fluorescent voltage-sensitive dyes to detect changes in the membrane potential and has been used to examine a range of ion channels including 5-HT_3 receptors.[35, 36] The methods were as previously described.[36] Briefly, blue fluorescent membrane potential dye (Molecular Devices Ltd., Wokingham, UK) was diluted in Flex buffer (10 mM HEPES, 115 mM NaCl, 1 mM KCl, 1 mM $CaCl_2$, 1 mM $MgCl_2$, 10 mM glucose, pH 7.4) and added to transfected cells grown on a 96-well plates. The cells were incubated at 37 ^{o}C for 30 min and then fluorescence was measured in a FlexStation™ (Molecular Devices Ltd.) every 2 s for 200 s. Buffer or ligand was added to each well after 20 s.

(iv) Data analysis

Concentration response and radioligand binding data were analyzed using Prism software (GraphPad, PRISM, San Diego, CA). Statistical analysis was performed using ANOVA in conjunction with a Dunnett's multiple comparisons post test, or an unpaired t-test as appropriate; $p < 0.05$ was taken as statistically significant.

(v) Supporting figures and tables

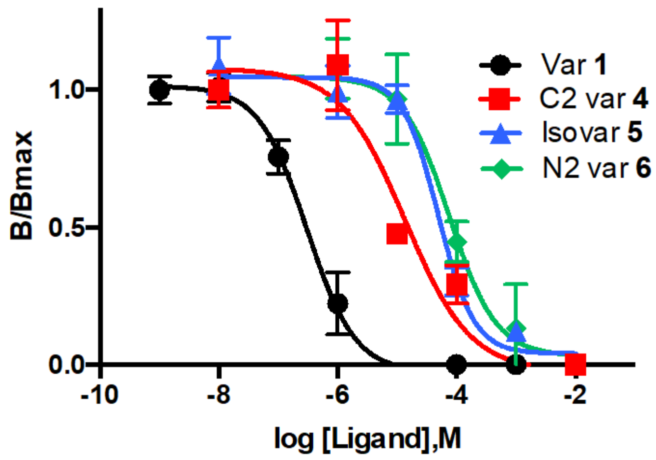

Figure S40. Inhibition of [^{3}H]GR65630 binding reveals varenicline **1** is more potent at the 5-HT$_3$A receptor than analogues **4-6** tested: IC$_{50}$ values were 0.3 μM (pIC$_{50}$= 6.517 ± 0.07), 13.6 μM (pIC$_{50}$= 4.866 ± 0.19), 49.6 μM (pIC$_{50}$ = 4.305 ± 0.08) and 75.8 μM (pIC$_{50}$= 4.12 ± 0.12) for varenicline **1**, C$_2$ varenicline **4**, isovarenicline **5**, and N$_2$ varenicline **6**, respectively (data = mean ± SEM, n=3).

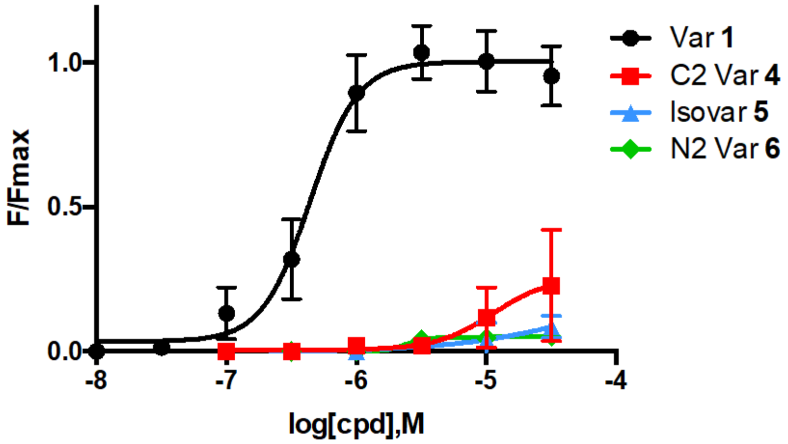

Figure S41. Functional studies using a fluorescent membrane-potential sensitive dye reveal varenicline **1** can activate 5-HT_3A receptors with an EC_{50} of 0.4 μM (pEC_{50} = 6.36 $\pm$ 0.05). No significant responses were observed upon application of C_2 varenicline **4**, isovarenicline **5**, and N_2 varenicline **6** at concentrations up to 30 μM. Fluorescent values (F) are normalized to the maximum response to varenicline **1** (Fmax), data = mean + SEM, n=4.

F. p*K*a Determination

Throughout, pKa refers to the pKa of the protonated form of the base, i.e. pKaH.

(i) Experimental description of the materials and assays

Table S3- Summary of all buffers used in spectrophotometric titrations of compounds reported here. The ionic strength of the aqueous component of each sample was maintained at I = 0.3 M with KCl. Aqueous solutions of HCl and KOH were used to access pH values outside of the range accessible by buffers.

Entry	Aqueous Solution	Total Concentration / M	%fb range	pH range
**1**	Hydrochloric acid (HCl)	0.01 – 0.3	-	0.66 – 2.08
**2**	Formic acid buffer (HCOOH/ HCOOK)	0.1	10 – 40	2.77 – 3.55
**3**	Acetic acid buffer (CH_3COOH/ CH_3COOK)	0.1	10 – 90	3.75 – 5.98
**4**	Phosphate buffer (KH_2PO_4/K_2HPO_4)	0.1	10 – 30	5.89 – 6.96
**5**	Triethanolammonium buffer ($N(CH_2CH_2OH)_3$.HCl)	0.1	10 – 90	6.96 – 9.06
**6**	Carbonate buffer ($KHCO_3$/K_2CO_3)	0.1	10 – 90	9.19 – 11.15
**7**	Triethylammonium buffer (NEt_3.HCl)	0.1	80 – 90	11.40 – 11.92
**8**	Potassium hydroxide (KOH)	0.1 – 0.3	-	12.98 – 13.39

Measurements of the pH of the buffer solutions used were performed using a Radiometer Analytical MeterLab® PHM210 Standard pH Meter with a Radiometer Analytical XC161 Combination pH electrode containing 3 M KCl solution saturated with AgCl.

UV-Vis absorbance spectra were obtained using a Varian Cary 100 Bio UV-Vis spectrophotometer with a temperature regulated cuvette holder and attached heating unit. All absorbance data were obtained at 25 °C. Spectra were obtained for each substrate for wavelengths in the range 800 nm to 200 nm at 600 nm min^{-1} (1 nm interval, 0.1 s average time, UV/Vis source change over at 350 nm) with baseline correction to account for absorbance due to the buffer present.

For single wavelength absorbance experiments, the mean absorbance at a chosen wavelength was measured over the course of 1 min, with correction of the absorbance due to the buffer

present. Margins of error reported for values of K_a and pK_a constants were taken from the standard error in the respective spectrophotometric titration.

(ii) Theoretical background

Due to the comparatively small amounts of material available (e.g. varenicline **1** and the variants described in the main text), a UV-Vis spectrophotometric titration method was employed for pK_a determination. To access the pK_a of a given compound, the change in absorbance at a chosen wavelength, λ_{obs}, is measured as the pH is changed. The observed absorbance at a given pH, A_{obs}, at λ_{obs} is determined by the concentrations of the protonated and deprotonated forms of the analyte.

Absorbance-pH data were fitted to Equation (1):

$$K_a = \frac{10^{-pH}(A_{max} - A_{obs})}{(A_{obs} - A_{min})} \quad (1)$$

Equation (2) was used when A_{obs} decreased with increasing pH at λ_{obs}.

$$A_{obs} = \frac{A_{max}.10^{-pH} + A_{min}.K_a}{10^{-pH} + K_a} \quad (2)$$

Where A_{obs} increased with increasing pH at λ_{obs}, the data were instead fitted to Equation (3).

$$A_{obs} = \frac{A_{min}.10^{-pH} + A_{max}.K_a}{10^{-pH} + K_a} \quad (3)$$

The following conditions were maintained for each titration: 25 °C, buffer ionic strength, I.S. = 0.3 M and 10 v/v% acetonitrile co-solvent in aqueous solution.

(iii) Spectrophotometric method validation: 4-dimethylaminopyridine (DMAP) and nicotine

As a control, a spectrophotometric titration of DMAP was performed under the experimental conditions described above (Figure S42) .

Figure S42- Spectrophotometric titration of DMAP

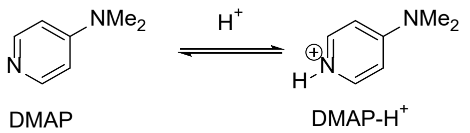

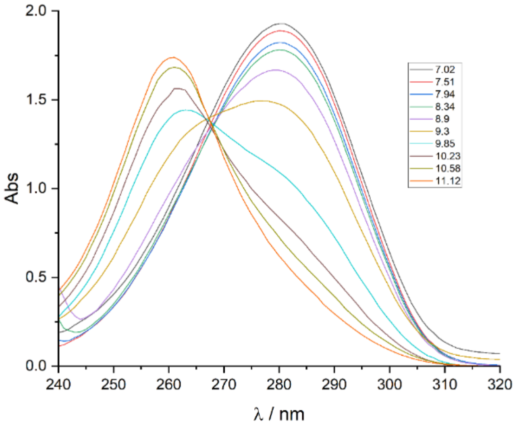

Analytical wavelengths (λ_{obs}) of 261 nm and 280 nm were chosen on either side of the isosbestic point at 268 nm. At 261 nm, DMAP is more absorbing than DMAP-H^+, therefore the data were fit to Equation (3). At 280 nm, the opposite is the case, so the data were fit to Equation (2). The titrations at both wavelengths are shown in Figure S43.

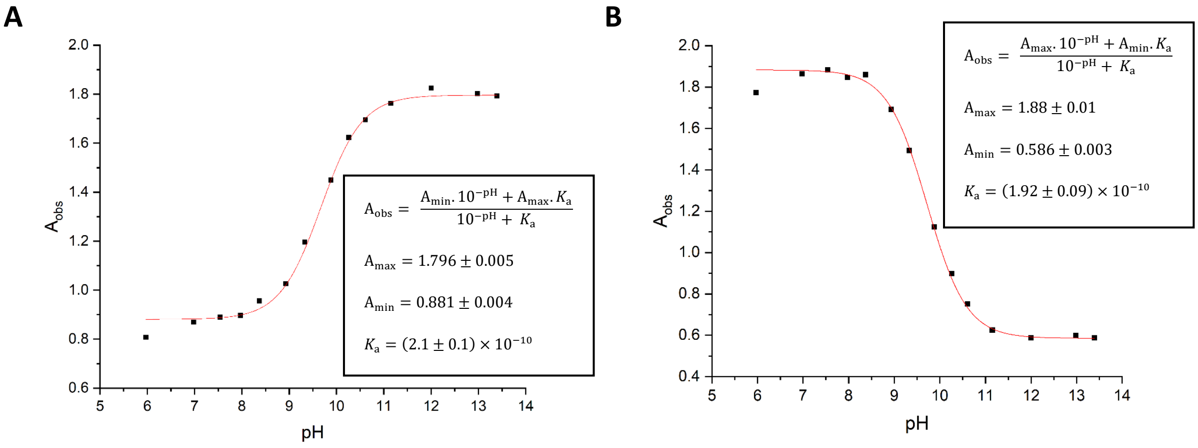

Figure S43- Spectrophotometric titration of DMAP at 261 nm (**A**) and 280 nm (**B**).

For the spectrophotometric titration of DMAP at 261 nm, the acid dissociation constant K_a of DMAP-H^+ was determined as (2.1 ± 0.1) x 10^{-10} M (pK_a = 9.67 ± 0.02) via equation (3). By analysis of data at 280 nm, K_a = (1.92 ± 0.09) x 10^{-10} M (pK_a = 9.72 ± 0.02) was obtained via Equation (2). The close similarity in pK_a values determined at both wavelengths and the independent literature value of 9.6, asserts the validity of this method.[37] The small 0.1 unit increase in pK_a of the conjugate acid of DMAP versus the literature value can be attributed to the presence of 10 vol% acetonitrile in aqueous solution, necessary for the solubility of the substrates in the present study. Increases in pK_a values of neutral and cationic acids are observed in the pure weak donor solvent MeCN versus more polar protic media.

The pK_a values (pK_{a1} pK_{a2}) of the conjugate acids of nicotine **2** were also determined under these experimental conditions to further validate the UV-Vis spectrophotometric method versus available literature values. The analytical wavelengths for the spectrophotometric titrations were 259 nm and 269 nm, respectively.

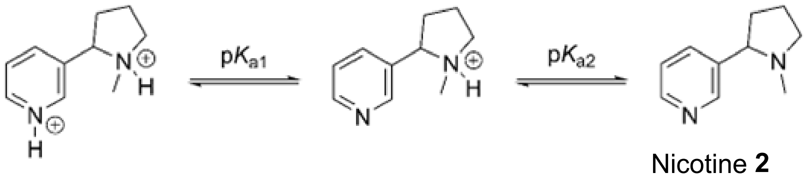

Nicotine **2**

Using the same protocol as described for DMAP, the observed absorbance change due to the deprotonation of the pyridinium moiety is larger than that for the *N*-methylpyrrolidinium component hence a greater concentration of nicotine **2** was necessary to determine K_{a2} (3.75 x 10^{-4} M) than for K_{a1} (1.67 x 10^{-4} M). Values of pK_{a1} = 3.27 $\pm$ 0.02 and pK_{a2} = 8.19 $\pm$ 0.03 were obtained by UV-Vis spectrophotometric titration under our experimental conditions. These pK_as values are closely similar to reported literature values in water which range from 3.04-3.41 for pK_{a1} and 7.94-8.02 for pK_{a2}.[38-41]

(iv) Spectrophotometric titration of varenicline 1, nicotine 2, cytisine 3 and varenicline variants 4-6

The pK_a values of varenicline **1**, nicotine **2**, cytisine **3**, and varenicline derivatives **4-6** were determined. Spectrophotometric titration curves are shown in Figures S44-S49 and the resultant pK_a values are summarized in Table S4.

Figure S44- Spectrophotometric titrations of nicotine **2**

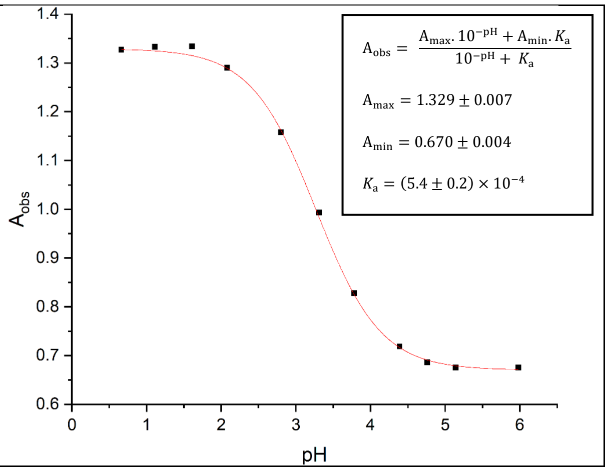

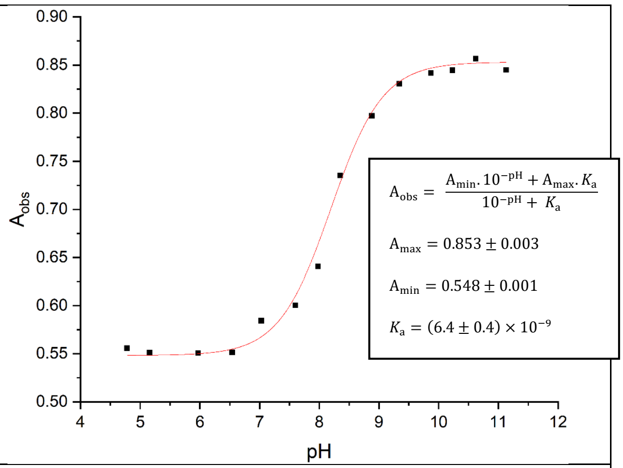

Figure S45- Spectrophotometric titration of cytisine **3**

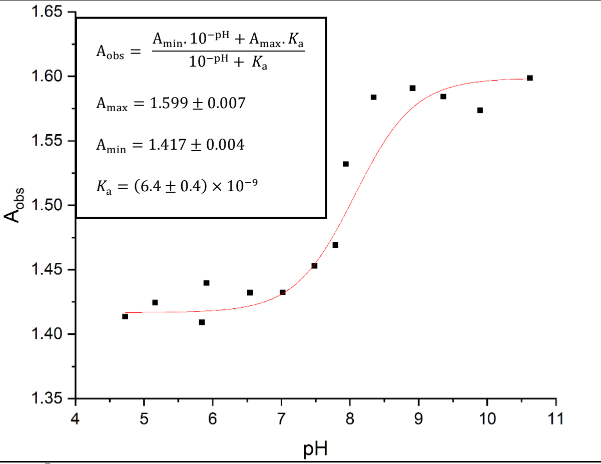

Figure S46- Spectrophotometric titration of varenicline tartrate **1**

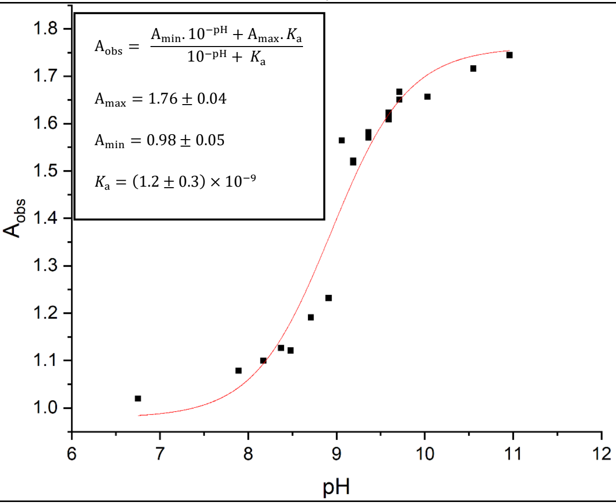

**Figure S47**- Spectrophotometric titration of C_2 varenicline hydrochloride **4**	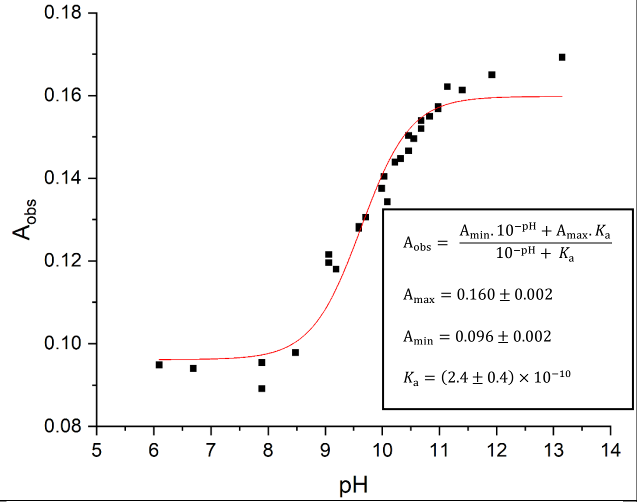
**Figure S48**- Spectrophotometric titration of isovarenicline hydrochloride **5**	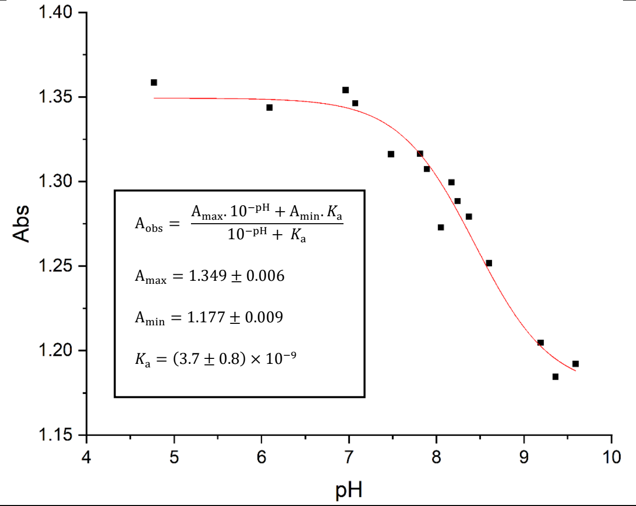
**Figure S49**- Spectrophotometric titration of N_2 varenicline trifluoroacetate **6**	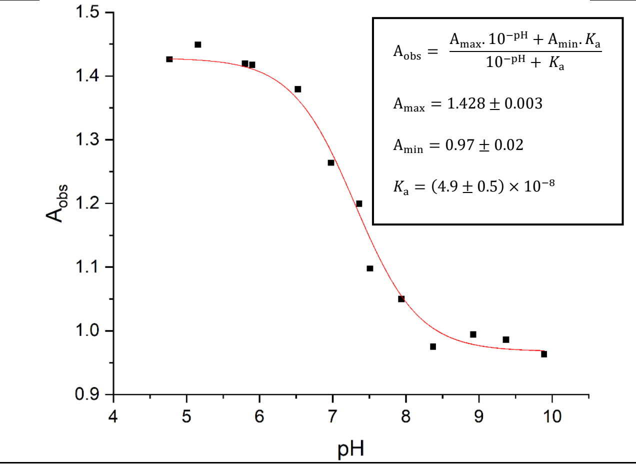

Table S4- Summary of experimentally-determined pK_a values. [a]Determined at 25 °C, buffer ionic strength, I.S. = 0.3 M and 10 v/v% acetonitrile co-solvent in aqueous solution.

**Compound**	**pK_a value[a]**	**Literature value**
4-Dimethylaminopyridine (DMAP)	9.67 ± 0.02	9.6[37]
Nicotine **2**	3.27 ± 0.02<br>8.19 ± 0.03	3.04 – 3.41[38-41]<br>7.94 – 8.02[38-41]
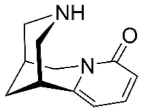<br>Cytisine **3**	8.07 ± 0.07	7.8[42]
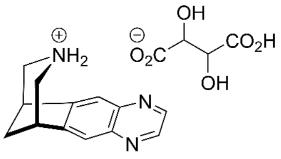<br>Varenicline tartrate **1**	8.90 ± 0.1	9.3[42]<br>9.2 ± 0.1[43]
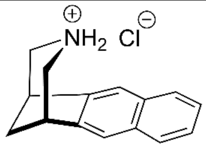<br>C_2 Varenicline hydrochloride **4**	9.63 ± 0.08	-
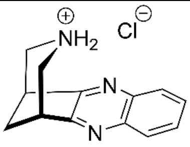<br>Isovarenicline hydrochloride **5**	8.44 ± 0.09	-
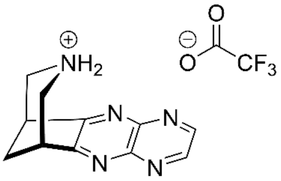<br>N_2 Varenicline trifluoroacetate **6**	7.31 ± 0.05	-

The pK_a value for varenicline tartrate **1** (8.9 ± 0.1) is ~0.3 units lower than the literature values (9.3[6] and 9.22 ± 0.13[7]). The literature value of 9.3 is provided without a quoted error or discussion of how the value was reached. The second literature value of 9.22 ± 0.13 was determined by a potentiometric titration in 100% water and at a different ionic strength of 0.15 M, which likely accounts for the observed difference of 0.29 units. The pK_a determined for cytisine **3** is 0.3 units greater than the literature value (7.8); again, this literature value is provided without a quoted error or discussion of how the value was reached.[42] The literature pK_a of the conjugate acid of piperidine is reported in the range 11.06-11.18.[38, 44-47]

G. Supplemental Methods References

(1) Paddon-Row, MN; Patney, HK. *An Efficient Synthetic Strategy for Naphthalene Annellation of Norbornenylogous Systems*. Synthesis. **1986**, *328*, 328-330.

(2) Grabowski, EY; AbuSalim, DI; Lash, TD. *Naphtho[2,3- b]carbaporphyrins*. J Org Chem. **2018**, *83*, 11825-11838.

(3) Brooks, P; Caron, S; Coe, J*; et al. Synthesis of 2,3,4,5-Tetrahydro-1,5-methano-1H-3-benzazepine via Oxidative Cleavage and Reductive Amination Strategies*. Synthesis. **2004**, *2004*, 1755-1758.

(4) Maier, L; Khirsariya, P; Hylse, O*; et al. Diastereoselective Flexible Synthesis of Carbocyclic C-Nucleosides*. J Org Chem. **2017**, *82*, 3382-3402.

(5) Muehlmann, FL; Day, AR. *Metabolite Analogs. V. Preparation of Some Substituted Pyrazines and Imidazo[b]pyrazines*. J Am Chem Soc. **1956**, *78*, 242-244.

(6) Walsh, RM, Jr.; Roh, SH; Gharpure, A*; et al. Structural principles of distinct assemblies of the human alpha4beta2 nicotinic receptor*. *Nature*. **2018**, *557*, 261-265.

(7) Minguez-Viñas, T; Nielsen, BE; Shoemark, DK*; et al. A conserved arginine with non-conserved function is a key determinant of agonist selectivity in α7 nicotinic acetylcholine receptors*. Br J Pharmacol. **2021**, *178*, 1651-1668.

(8) Campello, HR; Del Villar, SG; Honraedt, A*; et al. Unlocking nicotinic selectivity via direct C–H functionalisation of (−)-cytisine*. Chem. **2018**, *4*, 1710-1725.

(9) Mukherjee, S; Erramilli, SK; Ammirati, M*; et al. Synthetic antibodies against BRIL as universal fiducial marks for single-particle cryoEM structure determination of membrane proteins*. Nat Commun. **2020**, *11*, 1598.

(10) Morales-Perez, CL; Noviello, CM; Hibbs, RE. *X-ray structure of the human alpha 4 beta 2 nicotinic receptor*. Nature. **2016**, *538*, 411-415.

(11) DeLano, WL. *PyMOL molecular viewer: Updates and refinements*. Abstr Pap Am Chem S. **2009**, *238*,

(12) Abraham, MJ; Murtola, T; Schulz, R*; et al. GROMACS: High performance molecular simulations through multi-level parallelism from laptops to supercomputers*. SoftwareX. **2015**, *1-2*, 19-25.

(13) Lindorff-Larsen, K; Piana, S; Palmo, K*; et al. Improved side-chain torsion potentials for the Amber ff99SB protein force field*. Proteins. **2010**, *78*, 1950-1958.

(14) Sousa da Silva, AW; Vranken, WF. *ACPYPE - AnteChamber PYthon Parser interfacE*. BMC Res Notes. **2012**, *5*, 367.

(15) Jorgensen, WL; Chandrasekhar, J; Madura, JD*; et al. Comparison of simple potential functions for simulating liquid water*. J Chem Phys. **1983**, *79*, 926-935.

(16) Essmann, U; Perera, L; Berkowitz, ML. *A smooth particle mesh Ewald method*. J Chem Phys. **1995**, *103*, 8577-8593.

(17) Hess, B; Bekker, H; Berendsen, HJC*; et al. LINCS: a linear constraint solver for molecular simulations*. J Comput Chem. **1997**, *18*, 1463-1472.

(18) Miyamoto, S; Kollman, PA. *SETTLE: an analytical version of the SHAKE and RATTLE algorithms for rigid water models*. J Comput Chem. **1992**, *13*, 952-962.

(19) Bussi, G; Donadio, D; Parrinello, M. *Canonical sampling through velocity rescaling*. J Chem Phys. **2007**, *126*, 014101.

(20) Parrinello, M; Rahman, A. *Polymorphic transitions in single crystals: A new molecular dynamics method.* J Appl Phys. **1981**, ***52***, 7182–7190.

(21) Nosé, S; Klein, ML. *Constant pressure molecular dynamics for molecular systems*. Mol Phys. **1983**, *50*, 1055–1076.

(22) Kabsch, W; Sander, C. *Dictionary of protein secondary structure: pattern recognition of hydrogen-bonded and geometrical features*. Biopolymers. **1983**, *22*, 2577-2637.

(23) Oliveira, ASF; Shoemark, DK; Campello, HR*; et al. Identification of the initial steps in signal transduction in the α4β2 nicotinic receptor: insights from equilibrium and nonequilibrium simulations*. Structure. **2019**, *27*, 1171-1183.

(24) Oliveira, ASF; Edsall, C; Woods, C*; et al. A general mechanism for signal propagation in the nicotinic acetylcholine receptor family*. J Am Chem Soc. **2019**, *141*, 19953–19958.

(25) Xiu, X; Puskar, NL; Shanata, JA*; et al. Nicotine binding to brain receptors requires a strong cation-pi interaction*. Nature. **2009**, *458*, 534-537.

(26) Cashin, A; Petersson, E; Lester, H*; et al. Using physical chemistry to differentiate nicotinic from cholinergic agonists at the nicotinic acetylcholine receptor*. J Am Chem Soc. **2005**, *127*, 350-356.

(27) Marotta, CB; Rreza, I; Lester, HA*; et al. Selective ligand behaviors provide new insights into agonist activation of nicotinic acetylcholine receptors*. ACS Chem Biol. **2014**, *9*, 1153-1159.

(28) Tavares, XDS; Blum, AP; Nakamura, DT*; et al. Variations in binding among several agonists at two stoichiometries of the neuronal, alpha4beta2 nicotinic receptor*. J Am Chem Soc. **2012**, *134*, 11474-11480.

(29) Blum, AP; Lester, HA; Dougherty, DA. *Nicotinic pharmacophore: the pyridine N of nicotine and carbonyl of acetylcholine hydrogen bond across a subunit interface to a backbone NH*. Proc Natl Acad Sci U S A. **2010**, *107*, 13206-13211.

(30) Madeira, F; Madhusoodanan, N; Lee, J*; et al. The EMBL-EBI Job Dispatcher sequence analysis tools framework in 2024*. Nucleic Acids Res. **2024**, *52*, W521-W525.

(31) Valdar, WS. *Scoring residue conservation*. Proteins. **2002**, *48*, 227-241.

(32) Tasso, B; Canu Boido, C; Terranova, E*; et al. Synthesis, binding, and modeling studies of new cytisine derivatives, as ligands for neuronal nicotinic acetylcholine receptor subtypes*. J Med Chem. **2009**, *52*, 4345-4357.

(33) Moroni, M; Zwart, R; Sher, E*; et al. alpha4beta2 nicotinic receptors with high and low acetylcholine sensitivity: pharmacology, stoichiometry, and sensitivity to long-term exposure to nicotine*. Mol Pharmacol. **2006**, *70*, 755-768.

(34) Price, KL; Lummis, SC. *The role of tyrosine residues in the extracellular domain of the 5-hydroxytryptamine3 receptor*. J Biol Chem. **2004**, *279*, 23294-23301.

(35) Fitch, RW; Xiao, Y; Kellar, KJ*; et al. Membrane potential fluorescence: a rapid and highly sensitive assay for nicotinic receptor channel function*. Proc Natl Acad Sci U S A. **2003**, *100*, 4909-4914.

(36) Price, KL; Lummis, SC. *FlexStation examination of 5-HT3 receptor function using Ca2+ - and membrane potential-sensitive dyes: advantages and potential problems*. J Neurosci Meth. **2005**, *149*, 172-177.

(37) Kaljurand, I; Kutt, A; Soovali, L*; et al. Extension of the self-consistent spectrophotometric basicity scale in acetonitrile to a full span of 28 pKa units: unification of different basicity scales*. J Org Chem. **2005**, *70*, 1019-1028.

(38) Perrin, DD. *Dissociation Constants of Organic Bases in Aqueous Solution*; Butterworths, 1965.

(39) Vickery, H; Pucher, G. *The determination of 'free nicotine' in tobacco : The apparent dissociation constants of nicotine*. J Biol Chem. **1929**, *84*, 233–241.

(40) Fowler, RT. *A redetermination of the ionization constants of nicotine*. J App Chem. **1954**, *4*, 449–452.

(41) Barlow, RB; Hamilton, JT. *Effects of some isomers and analogues of nicotine on junctional transmission*. Br J Pharmacol Chemother. **1962**, *18*, 510-542.

(42) Rollema, H; Shrikhande, A; Ward, KM*; et al. Pre-clinical properties of the alpha4beta2 nicotinic acetylcholine receptor partial agonists varenicline, cytisine and dianicline translate to clinical efficacy for nicotine dependence*. Br J Pharmacol. **2010**, *160*, 334-345.

(43) Unal, G; Yeloglu, I; Anilanmert, B*; et al. pKa Constant of Varenicline*. J Chem Eng Data. **2012**, *57*, 14–17.

(44) Searles, S; Tamres, M; Block, F*; et al. Hydrogen Bonding and Basicity of Cyclic Imines*. J Am Chem Soc. **1956**, *78*, 4917–4920.

(45) Bates, R; Bower, V. *Dissociation Constant of Piperidinium Ion from 0-Degrees to 50-Degrees-C and Related Thermodynamic Quantities*. J Res Nat Bur Stand. **1956**, *57*, 153–157.

(46) Horwitz, JP; Rila, CC. *A Comparison of the Reactions of Some Amines with Nitrosoguanidine, Cyanamide and S-Methylisothiourea Hydrochlorides*. J Am Chem Soc. **1958**, *80*, 431–437.

(47) Geissman, TA; Wilson, BD; Medz, RB. *The Base Strengths of cis- and trans-1,2-Aminoalcohols*. J Am Chem Soc. **1954**, *76*, 4182–4183.
