# Supplementary material for: Understanding varenicline function via key receptor and ligand interactions
Source: Cell Rep Phys Sci. 2025 Dec 17;6(12):102992. doi: 10.1016/j.xcrp.2025.102992 (PMC12711630; doi:10.1016/j.xcrp.2025.102992)
Supplement: Document S2. Article plus supplemental information [file mmc2.pdf]

Cell Reports Physical Science

Article

Understanding varenicline function via key receptor and ligand interactions

Graphical abstract

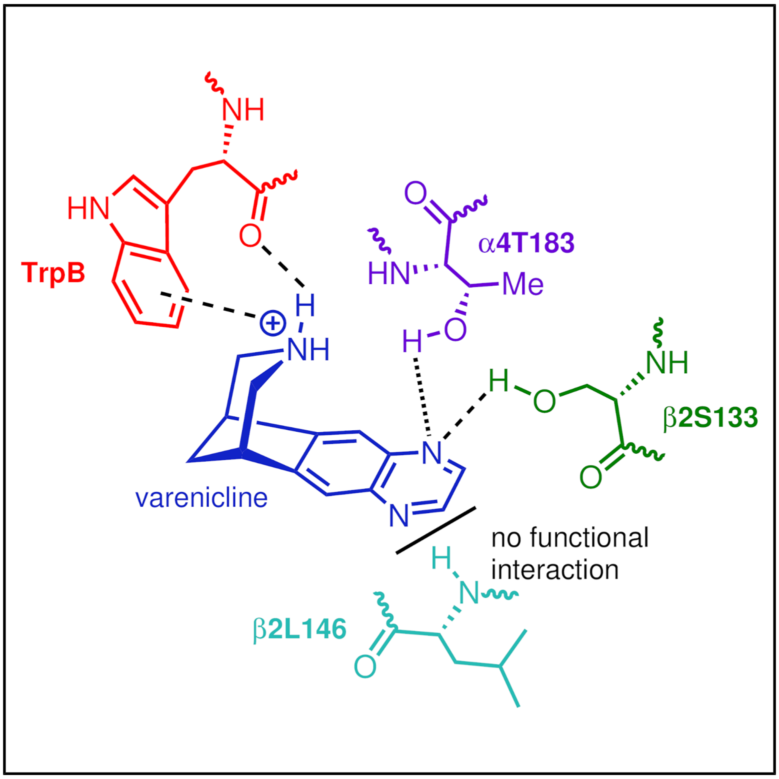

Authors

Sheenagh G. Aiken, Daniele Fiorito, Matthew Harper, ..., Isabel Bermudez, Timothy Gallagher, A. Sofia F. Oliveira

Correspondence

ibermudez@brookes.ac.uk (I.B.), t.gallagher@bristol.ac.uk (T.G.), sofia.oliveira@bristol.ac.uk (A.S.F.O.)

In brief

Aiken et al. reveal that serine and threonine hydrogen-bond donors, alongside cation-π and hydrogen-bond acceptor interactions, shape varenicline's partial agonism at α4β2 nicotinic receptors. Structural variants show the heteroaryl hydrogen-bond acceptor's position is crucial for activating α4β2 and 5-HT_3 receptors across multiple related binding contexts and functional receptor assays *in vitro*.

Highlights

- Varenicline interacts with serine and threonine donors to drive α4β2 nAChR partial agonism
- β2S133 and α4T183 are key in the α-β binding site, with β2S133 the critical determinant
- β2S133V reduces varenicline efficacy, underscoring β2S133 functional importance
- New varenicline variants pinpoint the quinoxaline moiety's role for receptor activation

Aiken et al., 2025, Cell Reports Physical Science *6*, 102992
December 17, 2025 © 2025 The Authors. Published by Elsevier Inc.
https://doi.org/10.1016/j.xcrp.2025.102992

Cell Reports
Physical Science

CellPress
OPEN ACCESS

Article

Understanding varenicline function via key receptor and ligand interactions

Sheenagh G. Aiken,[1] Daniele Fiorito,[1] Matthew Harper,[1] Grzegorz Pikus,[1] Juno Underhill,[1,6] Jacob Murray,[2] Joshua Rawlinson,[2] AnnMarie C. O'Donoghue,[2] Cecilia Gotti,[3] Sarah C.R. Lummis,[4] Teresa Minguez Viñas,[5] Franco Viscarra,[5] Isabel Bermudez,[5,*] Timothy Gallagher,[1,6,*] and A. Sofia F. Oliveira[1,6,7,*]

[1]School of Chemistry, University of Bristol, Bristol BS8 1TS, UK
[2]Department of Chemistry, Durham University, South Road, Durham DH1 3LE, UK
[3]CNR, Institute of Neuroscience, University of Milan, 20129 Milan, Italy
[4]Department of Biochemistry, University of Cambridge, Cambridge CB2 1QW, UK
[5]Department of Biological and Medical Sciences, Oxford Brookes University, Oxford OX3 0BP, UK
[6]Centre for Computational Chemistry, School of Chemistry, University of Bristol, Bristol BS8 1TS, UK
[7]Lead contact
*Correspondence: ibermudez@brookes.ac.uk (I.B.), t.gallagher@bristol.ac.uk (T.G.), sofia.oliveira@bristol.ac.uk (A.S.F.O.)
https://doi.org/10.1016/j.xcrp.2025.102992

SUMMARY

Approved by the US Food and Drug Administration in 2006, varenicline was the first nicotinic-based therapy for smoking cessation, targeting the α4β2 nicotinic acetylcholine receptor (nAChR). While inspired by cytisine, varenicline has distinct effects at both target and off-target receptors; however, despite being widely used clinically, the precise molecular interactions underpinning varenicline's mode of action remain unclear. Using a multidisciplinary approach, the interactions that set varenicline apart from related compounds such as nicotine and cytisine have been identified. In particular, the binding-site residues α4T139, α4T183, and especially β2S133 were shown to be key modulators for varenicline's function. Substituting β2S133 with valine significantly reduced efficacy, pinpointing it as a crucial determinant. Additionally, a set of novel varenicline variants showed that the positioning of the quinoxaline moiety in varenicline is essential for receptor activation. These insights reveal a unique interaction network at α4β2 that underlies varenicline's function, offering a deeper understanding of the ligand's working mechanism.

INTRODUCTION

Tobacco consumption, with the World Health Organization estimating >8 million deaths annually, is a leading cause of preventable disease and death worldwide. Of this total, 7 million deaths are attributable to direct smoking, and approximately 1.3 million are due to second-hand smoke exposure.[1,2] As a result, smoking cessation still represents a major but frustratingly challenging and increasingly ephemeral global health objective.[3–7] Further, recent declines in the prevalence of tobacco consumption have slowed, legislation to limit tobacco sales has failed to keep pace (or been reversed), and this situation has been exacerbated by a sharp increase in nicotine consumption via electronic vapes[8–11] and pouches.[12,13] Consequently, this major public health threat should be viewed as one of nicotine, as opposed to solely tobacco, addiction.[14–17]

A key part of the smoking cessation toolkit is varenicline **1** (Figure 1A), which was launched in 2006 as Chantix (Champix in Europe)[18] to support smoking cessation.[17,18–20] Available (since 2022) in generic form, varenicline **1** is estimated to have been used by >24 million smokers and represents the first nicotinic acetylcholine receptor (nAChR) therapeutic[21] approved by the US Food and Drug Administration.

As a partial agonist, varenicline **1** targets the α4β2 subtype of the nAChRs found in the central nervous system.[19,27–29] Due to its high affinity for nicotine **2**, this subtype emerged as the primary focus for nicotine addiction[14,30,31] and, consequently, the primary target receptor for smoking cessation.[14,30,31] Besides the α4β2 subtype, varenicline **1** also activates (as a full agonist) the α7 subtype,[29,32,33] although the α7 nAChR role in smoking cessation remains undetermined. Significantly, varenicline **1** also activates the 5-HT_3 serotonin receptor,[34] a structurally related member of the Cys loop superfamily.[15,35]

The genesis of varenicline **1** as a novel (and consequently patentable) smoking cessation agent lies in the known profile of cytisine (**3** [the generic drug name cytisinicline was designated by the USAN Council in 2018]) (Figure 1A).[36,37] Cytisine **3**, isolated from laburnum[38] and used in eastern Europe as a smoking cessation agent since the 1960s,[39–43] is both a high-affinity partial agonist for the α4β2 nAChR and a full agonist at the α7 subtype.[29,32,44,45] However, varenicline **1** and cytisine **3** diverge in terms of their functional profile at the α4β2 and 5-HT_3 receptors. First, varenicline **1** and cytisine **3** have substantially different profiles in α4β2 nAChR,[29,32,44,45] which presents in two receptor stoichiometries: $(\alpha4)_2(\beta2)_3$, the high-sensitivity (to activation by ACh; HS) complex, which contains two α-β binding sites; and

© 2025 The Authors. Published by Elsevier Inc.
This is an open access article under the CC BY license (http://creativecommons.org/licenses/by/4.0/).

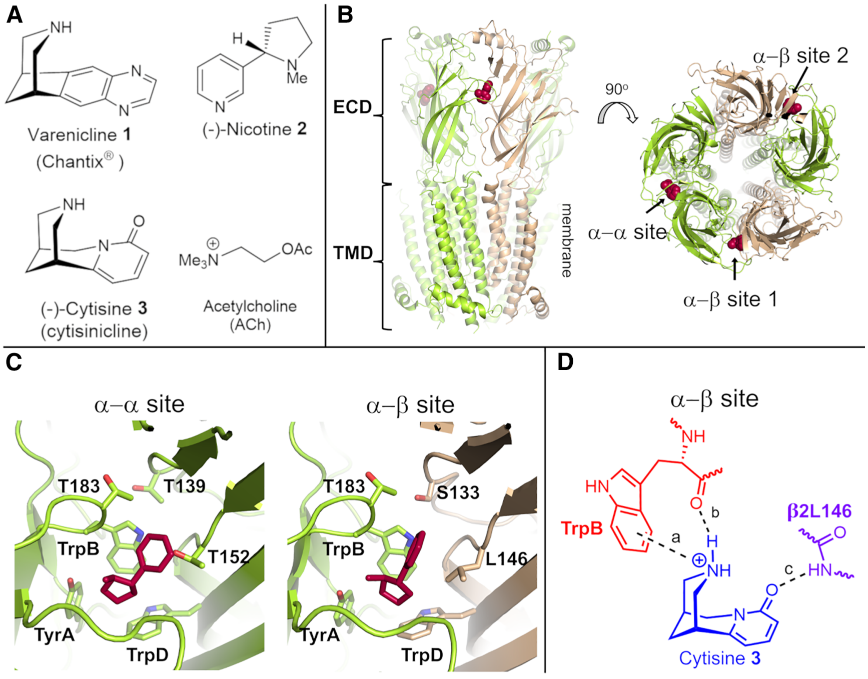

Figure 1. The α4β2 nAChR: structure, agonists, and key agonist-receptor interactions

(A) Chemical structures of varenicline **1**, nicotine **2**, cytisine **3**, and ACh.

(B) Cryo-EM structure of the human LS isoform of the α4β2 nAChR (i.e., $(\alpha4)_3(\beta2)_2$) with nicotine bound (PDB: 6CNK).[22] nAChRs are composed of three domains: an extracellular domain (ECD), a transmembrane domain (TMD), and an intracellular domain (ICD); the ICD is absent in the cryo-EM 6CNK structure.[22] The LS isoform of the α4β2 nAChR contains one α-α pocket (at the interface between the two α4 subunits) and two α-β sites (formed by an α4 and a β2 subunit).

(C) Close-up view of the α-α and α-β binding pockets in 6CNK.[22] The side chains of the several conserved residues, namely TyrA (Y126 in the principal α4 side), TrpB (W182 in the principal α4 side), and TrpD (W88 in the complementary α4 side of the α-α pocket and W82 in the complementary β2 side of the α-β pocket) as well as the residues that are the focus of the current work, notably α4T183 (principal α4 side), α4T139 (complementary α4 side of the α-α pocket), β2S133 (complementary β2 side of the α-β pocket), α4T152 (complementary α4 side of the α-α pocket), and β2L146 (complementary β2 side of the α-β pocket), are shown with sticks. The residue numbers refer to UniProt sequences P43681 and P17787 for the human α4 and β2 subunits, respectively. Here and in (B), the α4 and β2 subunits are colored yellow and light brown, respectively. Nicotine **2** is highlighted in red.

(D) Dougherty-Lester nAChR functionally important binding model for the α-β pocket illustrated for cytisine **3** showing the three key functional interactions identified: (a) cation-π and (b) backbone C=O as H-bond acceptor associated with TrpB within the α4 subunit; and (c) backbone NH as H-bond donor associated with β2L146 (in the complementary β2 subunit).[23–26]

$(\alpha4)_3(\beta2)_2$, the low-sensitivity (toward ACh; LS) complex, which exhibits two α-β sites plus an α-α pocket (Table 1; Figures 1B and 1C).[29,32] Varenicline **1** is efficacious in both α4β2 isoforms, while cytisine **3** only activates the LS stoichiometry; the efficacy at both isoforms of varenicline **1**, nicotine **2**, and cytisine **3** (relative to ACh, which is a full agonist) in the α4β2 subtype are shown in Table 1.[21] Varenicline **1**, nicotine **2**, and cytisine **3** are all partial agonists (with an efficacy relative to ACh of 0.18, 0.31, and 0.025 for the HS isoform and 0.41, 0.53, and 0.23 for the LS isoform), whereas ACh is a full agonist of both LS and HS isoforms of the α4β2 receptor (Table 1).

Second, cytisine **3** is a weak antagonist at the 5-HT_3 receptor, unlike varenicline **1**, which is an agonist at this receptor,[34,46] with this difference linked to side effects experienced with varenicline **1** therapy.[47] These differing profiles of otherwise two closely related compounds raise fundamental questions about their underlying mechanisms of action at the α4β2 nAChR. First, what are the details of the protein-ligand interactions that both mediate and differentiate the functional effects of varenicline **1** vs. cytisine **3** at the α4β2 nAChR? Second, how does varenicline **1** binding translate into efficacy, i.e., receptor activation? Clearly, this begs the question that if varenicline **1** differs from cytisine **3** in terms of how it binds to the receptor, does that difference regulate function? Further, if the specifics of the α4β2 interactions are different, does this shed light on why varenicline **1** is an agonist while cytisine **3** is an antagonist at the human 5-HT_3 receptor? All these questions direct us toward the need to enhance our understanding of the network of receptor-ligand interactions that govern the wider functional profile of varenicline **1**.

The seminal work of Dougherty and Lester,[23–26,48,49] together with structural studies of soluble acetylcholine-binding proteins (AChBP)[50–53] and recent high-resolution structural data for complete nAChRs,[54–58] including the α4β2-nicotine, acetylcholine, and varenicline complexes,[22,59–61] has defined a binding model for nicotinic agonists (Figure 1D).

The salient features of the Dougherty-Lester model consist of a cation-π and H-bond donor (except for acetylcholine) associated with the piperidinium center and a highly conserved tryptophan residue in loop B (TrpB) in the principal face of the agonist binding site,[23,24] and an H-bond acceptor component within the ligand (C=O, in the case of cytisine **3**) that interacts with a donor within the complementary face (Figure 1D).[25] In addition to TrpB, the binding pockets are formed by several conserved aromatic residues, including a tyrosine in loop A (TyrA), two tyrosines in loop C (TyrC1 and TyrC2), and a tryptophan in loop D (TrpD). These residues play a critical role in ligand recognition and binding.[62] For most agonists bound to the α4β2 nAChR, the H bond with the complementary face involves an interaction with the backbone NH of β2L146 (which corresponds to β2L119 in the work of Blum et al.[25]) in the complementary face.[25,26,49] However, while this interaction mediates ACh, nicotine **2**, cytisine **3**, sazetidine-A, carbamylcholine, and epibatidine function,[25,26,49] varenicline **1** was shown not to engage with β2L146, at least in

CellPress
OPEN ACCESS

Table 1. Potency and relative efficacy of nicotinic ligands at wild-type and targeted mutants of both HS and LS isoforms of the α4β2 nAChR

	ACh		Varenicline **1**		Nicotine **2**		Cytisine **3**	
Mutation	EC_{50} (μM)	RE	EC_{50} (μM)	RE	EC_{50} (μM)	RE	EC_{50} (μM)	RE
$(\alpha4)_2(\beta2)_3$ (HS isoform)								
WT	3.2 ± 0.7	1	0.090 ± 0.01	0.18 ± 0.02	1.77 ± 0.4	0.31 ± 0.01	ND	0.025 ± 0.004
β2S133V	4 ± 1.3	1	3.10 ± 0.4[a]	0.059 ± 0.017[a]	3.0 ± 0.5	0.23 ± 0.02[a]	ND	0.016 ± 0.002
α4T183V	3.4 ± 0.4	1	0.57 ± 0.01[a]	0.099 ± 0.004[a]	2.9 ± 0.5	0.29 ± 0.01	ND	0.021 ± 0.003
α4T139V	2.8 ± 0.4	1	0.051 ± 0.001	0.18 ± 0.02	1.5 ± 0.4	0.29 ± 0.02	ND	0.023 ± 0.001
β2S133Vα4T183V	3.3 ± 1.0	1	2.47 ± 0.6[a]	0.07 5 ± 0.0031[a]	3.2 ± 0.5	0.20 ± 0.01[a]	ND	0.018 ± 0.003
β2S133Vα4T139V	3.5 ± 0.9	1	3.58 ± 0.4[a]	0.067 ± 0.018[a]	2.8 ± 0.9	0.22 ± 0.02[a]	ND	0.022 ± 0.009
$(\alpha4)_3(\beta2)_2$ (LS isoform)								
WT	99 ± 10	1	1.15 ± 6	0.41 ± 0.02	7.5 ± 1.6	0.53 ± 0.05	2.9 ± 0.8	0.23 ± 0.08
β2S133V	105 ± 15	1	33 ± 1.4[a]	0.02 ± 0.003[a]	11.2 ± 2	0.48 ± 0.06[a]	5.1 ± 1	0.21 ± 0.01
α4T183V	95 ± 10	1	8.0 ± 0.3[a]	0.20 ± 0.02[a]	6.9 ± 2	0.52 ± 0.03	4.6 ± 1	0.22 ± 0.02
α4T139V	88 ± 9	1	8.4 ± 5.4[a]	0.20 ± 0.02	7.4 ± 1	0.54 ± 0.05	2.5 ± 0.7	0.19 ± 0.02
β2S133Vα4T183V	101 ± 4	1	33.0 ± 5[a]	0.033 ± 0.005[a]	10.2 ± 2	0.47 ± 0.01[a]	6.9 ± 1.5	0.19 ± 0.01
β2S133Vα4T139V	98 ± 10	1	41.34 ± 2[a]	0.031 ± 0.005[a]	11.4 ± 2	0.46 ± 0.02[a]	5.8 ± 1.4	0.18 ± 0.01

Relative efficacy (RE) was determined by normalizing the maximal current responses elicited by varenicline **1** to the maximal current response to ACh (I/I_{max}ACh). EC_{50} values were estimated as described in supplemental methods. Data shown represent the mean ± SEM of n = 8–10 experiments in 6–8 different batches of *Xenopus* oocytes. Statistical differences between wild-type (WT) and mutant receptors were determined by one-way ANOVA followed by a post hoc Dunnett's test and/or a post hoc Bonferroni multiple comparison test to determine the level of significance between WT and mutants. ND, not determined due to low levels of functional expression (less than 50 nA of ACh maximal currents).
[a]Statistically significant difference ($p < 0.05$) between mutant and WT receptors.

terms of linking to receptor function, in the α4β2 subtype.[49] Dougherty and co-workers used changes in EC_{50} to determine the relevance of a ligand-receptor interaction,[48] and backbone mutation of β2L146 did not affect varenicline **1** function in either of the LS and HS α4β2 isoforms.[49] Note, however, that these findings do not exclude the involvement of β2L146 in varenicline **1** recognition/binding. Moreover, other interactions within the agonist binding sites can contribute to subtype differentiation. For instance, we have shown how α7R101 and β2R106 can modulate functional outcomes across nAChR subtypes[32,63] and how agonist-induced structural and dynamics changes are transmitted from the agonist binding site (in α4β2 and α7) to the ion channel,[64,65] allowing gating to occur.

Given that Dougherty and Lester demonstrated that varenicline **1** retains the cation-π and H-bond donor components in the α4β2 nAChR (Figure 1D)[26] but lacks the β2L146 H-bond donor interaction, we have focused here on uncovering the specific residue(s) that partner with varenicline (as an H-bond acceptor) in shaping the functional profile of this ligand.

To investigate the variation of receptor-ligand network that mediates function across different nicotinic agonists, including varenicline **1**, we have integrated computational and experimental approaches to identify networks of H-bond donor interactions within the primary and complementary faces of α4β2 nAChR. In addition, we have also designed a unique set of varenicline **1** variants that probe the ligand features that mediate binding to and function of the human α4β2 nAChR. These studies offer a nuanced explanation of how and why the profile of varenicline **1** deviates from that of other nicotinic ligands, including cytisine **3**, in the α4β2 nAChR. We demonstrate that varenicline **1** engages in functionally relevant interactions with β2S133 on the complementary β2 side and (to a lesser extent) with α4T183 on the principal α4 side of the agonist pocket. Notably, the interaction with β2S133 plays a substantial role in shaping the functional profile of varenicline **1**, emerging as a distinguishing feature of its association with the receptor. Varying varenicline **1** structure suggests that the size and shape of the ligand (which were both retained in all designed variants) are significant for binding at α4β2 nAChR, but that function is associated with the precise location of the H-bond acceptor (quinoxaline) moiety within varenicline **1**. A similar picture was observed regarding the location of the quinoxaline group at the 5-HT_3 receptor, where it was also shown to be a critical determinant of varenicline's profile.

RESULTS

Identifying alternative H-bond networks in the agonist binding sites of α4β2 nAChR

Molecular dynamics (MD) simulations were performed for several α4β2 nAChR-agonist complexes to identify alternative H-bonding donor networks within the α4β2 agonist binding sites (see supplemental methods and Table S1). The complexes between the extracellular domain (ECD) of the human α4β2 LS and HS isoforms and varenicline **1**, nicotine **2**, cytisine **3**, and ACh were constructed (wild-type [WT] complexes) and optimized (Figures S2 and S3) as described in supplemental methods.

In the WT varenicline **1** models, the H-bond acceptor quinoxaline moiety does not directly engage with the backbone amide of either α4T152 (in the α-α pocket) or β2L146 (in the α-β pockets) (Figure 2A), which is consistent with Dougherty and co-workers'

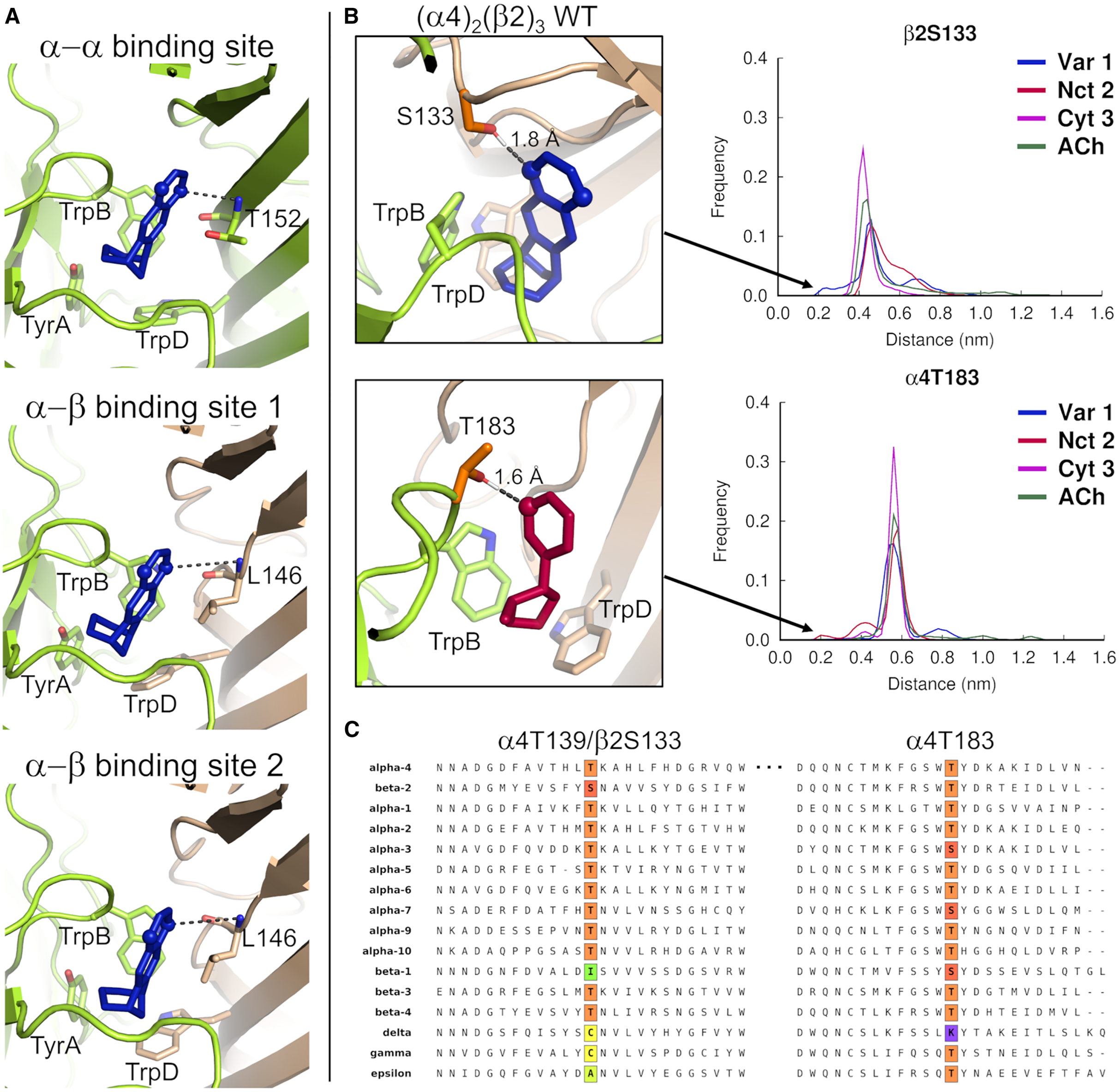

Figure 2. Varenicline interactions in the α-α and α-β binding sites in the wild-type complexes

(A) Optimized binding mode for varenicline **1** in the α-α and α-β binding sites in the LS wild-type complex. The α4 and β2 subunits are colored yellow and brown, respectively. TyrA, TrpB, TrpD, α4T152, and β2L146 are shown as sticks. Varenicline **1** is colored blue, with the nitrogen atoms of the quinoxaline moiety highlighted by spheres. Note that the distance between the nearest H-bond acceptor in the quinoxaline ring and the backbone NH group of α4T152 and β2L146 (indicated by dashed lines) exceeds 3.8 Å (distances >3.8 Å are also observed for the α-β binding sites in the HS complex), suggesting that no H bond is formed between these two groups.

(B) Distribution of the minimum distance between the agonist (specifically, the closer pyrazine nitrogen in the quinoxaline moiety of varenicline **1**, the pyridine nitrogen of nicotine **2**, the pyridone oxygen of cytisine **3**, and the carbonyl oxygen of ACh) and α4T183 and β2S133 in the α-β binding pockets of the HS wild-type complex (right panels). The left panels illustrate examples of conformations where a hydrogen bond between varenicline **1** and β2S133 and nicotine **2** and α4T183 are present (as indicated by the dashed lines). The α4 and β2 subunits are colored yellow and brown, respectively, whereas varenicline **1** and nicotine **2** are highlighted in blue and red. The side chains of TrpB, TrpD, α4T183, and β2S133 are represented by sticks. The nitrogen atoms of the quinoxaline ring of varenicline **1** and the pyridine nitrogen of nicotine **2** are highlighted by spheres.

(C) Sequence alignment for the α4T183, α4T152/β2L146, and α4T139/β2S133 regions of various human nAChR subunits. The colored boxes highlight the locations of α4T183, α4T139, and β2S133 (the residues mutated in this work), with threonine, serine, isoleucine, cysteine, alanine, and lysine residues represented by orange, red, green, yellow, light green, and purple, respectively. The white box marks the location of α4T152 and β2L146, which were not mutated here. The sequences shown correspond to UNIPROT: P43681 (human α4), UNIPROT: P17787 (human β2), UNIPROT: P02708 (human α1), UNIPROT: Q15822 (human α2), UNIPROT: P32297 (human α3), UNIPROT: P30532 (human α5), UNIPROT: Q15825 (human α6), UNIPROT: P36544 (human α7), UNIPROT: Q9U6MI (human α9), UNIPROT: Q9GZZ6 (human α10), UNIPROT: P11230 (human β1), UNIPROT: Q05901 (human β3), UNIPROT: Q07001 (human δ), UNIPROT: P07510 (human γ), and UNIPROT: Q04844 (human ε). The sequence alignments were performed using the Muscle server.[66]

observation that β2L146 does not modulate the function of varenicline **1**.[49]

MD simulations were performed for the WT HS and LS complexes to probe relevant differences in H-bond patterns within the receptor. These simulations provided a more complete picture of the dynamic behavior of the complexes and the nature of the ligand-receptor interactions (i.e., cation-π and H-bond interactions) within the α-β and α-α binding sites (Figures S6–S19). Besides the well-established interactions with TrpB, TyrA, and TrpD (Figures S17–S19), analysis of the distances between the H-bond acceptor in varenicline **1**, nicotine **2**, cytisine **3**, and ACh and H-bond donors within the binding pockets further identified three alternative residues that can transiently interact with some of the agonists (Figure S20). These involved α4T183 in the principal face of the pocket and β2S133 and α4T139 in the complementary face of the α-β and α-α pockets, respectively. As can be seen in Figures 2B and S20, the H-bond acceptor groups of the agonists can closely approach the side-chain H-bond donors of α4T183, β2S133, and α4T139, suggesting that a transient (either direct or mediated by a water molecule) interaction between these groups is feasible.

Within the α-β binding sites, the residues identified as potential H-bond partners for the agonists were the hydroxyl groups of α4T183 and β2S133. While α4T183 is positioned on the side of the pocket immediately after the key TrpB residue, β2S133 lines the back of the orthosteric site (Figure 1C). In our simulations, only one of the quinoxaline nitrogen atoms of (meso) varenicline **1** was able to closely contact β2S133, while the pyridine nitrogen of nicotine **2** was able to interact with the α4T183 (Figures 2B and S20). In the LS isoform, which contains an additional agonist site on the α-α subunit interface,[22,67] the new interaction is associated with α4T139, which is located in the complementary face in a position analogous to that of β2S133 on the α-β interface (Figure S20). Note that the interactions involving α4T139/β2S133 and α4T183 do not involve backbone NH donors (as in the case of β2L146 in Figure 1D) but rather the hydroxyl-containing side chain and are potentially synergistic in terms of the network offered.

Sequence alignment of nAChR subunits indicates that the presence of an H-bond donor group at positions equivalent to α4T139/β2S133 and α4T183 is highly conserved across human receptors (Figure 2C; for further details, see Figure S21). All human neuronal subunits possess a residue with a hydroxyl-containing side chain at the α4T139/β2S133 position, with the α2-α7, α9-α10, and β3-β4 subunits featuring a threonine and the β2 subunit a serine. In contrast to the human neuronal subunits, the muscle subunits (i.e., α1, β1, γ, δ, and ε) exhibit greater diversity in the residue at position α4T139/β2S133, with less scope to participate in H bonding.[68,69] At the α4T183 position, all human neuronal and muscle subunits have either a threonine or a serine, except for a lysine in the δ subunit. The highly conserved nature of residues at the α4T139/β2S133 and α4T183 positions in neuronal subunits suggests that the side-chain hydroxyl group, acting as an H-bond donor, may play a key role in defining the action of certain ligands.

In the WT simulations, varenicline **1** exhibits strong positively concerted motions with α4T139, α4T183, and β2S133 in both α-α and α-β sites, whereas the correlation profiles for nicotine **2**, cytisine **3**, and ACh vary between pockets, generally showing weaker correlations with the α-α pocket (Figure S22). Previous work comparing the α-α and α-β sites has shown that their differences stem primarily from the chemical characteristics of three residues located on the complementary face of the sites, namely the hydrophobic β2V136, β2F144, and β2L146 in the α-β pocket and the polar α4H142, α4Q150, and α4T152 in the α-α site.[49,67,70,71] These substitutions alter the pocket properties, increasing its hydrophilicity and influencing agonist affinity and functional profile.

The distance between the H-bond acceptor in varenicline **1**, nicotine **2**, cytisine **3**, and ACh, and the backbone NH donor of β2L146 (in the α-β pockets) and α4T152 (at the equivalent position within the α-α pocket) and the α4T152 hydroxyl donor in the residue's side chain, was also determined (Figure S23). These profiles clearly demonstrate that cytisine **3** (and, to a lesser extent, nicotine **2** and ACh) can directly interact with the α4T152 side chain in the α-α site. A role for residues α4H142 and α4Q150, both located on the complementary side of the α-α pocket, was also evaluated, but no interactions between these residues and the ligands were observed (Figure S23). The interaction observed with the side chain of α4T152, specific to the α-α pocket, highlights the distinct nature of this pocket compared to the α-β site and the unique interactions this region can form with agonists. The presence of an α-α pocket in the α4β2 nAChR lowers the overall receptor sensitivity to agonists like varenicline **1**, nicotine **2**, and cytisine **3** (Table 1) but increases their relative efficacy compared to the HS isoform.[72,73] Also, the α-α pocket is a known binding site for allosteric modulators, such as NS9283, that increase agonist efficacy.[74–76]

Receptor mutations to explore new H-bond interactions within the α4β2 binding sites

As MD simulations suggested that β2S133, α4T183, and α4T139 can sustain hydrogen-bonding interactions with certain agonists (Figures 2 and S20), we posited that removal of the hydroxyl donor(s) from these residues would affect agonist binding (particularly for varenicline **1**) to the α4β2 nAChR, resulting in a decrease in their functional potency (half-maximal effective concentration [EC_{50}]) and/or relative efficacy (RE). To test this hypothesis, we employed a valine substitution approach to create the corresponding β2S133V, α4T183V, and α4T139V mutants.

Using oocytes expressing heterologously HS or LS receptor mutants, concentration-response curves were obtained for varenicline **1**, nicotine **2**, cytisine **3**, and ACh. These are shown in Figures 3 and S34–S38, with data summarized in Table 1. The β2S133V significantly reduced the EC_{50} and RE of varenicline **1** at the α4β2 nAChR, with approximately 30-fold decrease in potency at both receptor isoforms. Changes in RE were more pronounced at the LS stoichiometry (with 95% of efficacy loss in LS relative to WT compared to a 67% reduction in the HS), although the difference between isoforms could be due to the challenges of measuring the very low efficacy of varenicline **1** in the β2S133V mutant. The α4T183V mutation also affected the EC_{50} and RE of varenicline **1** at both isoforms. Nonetheless, the effect was less marked than with the β2S133V mutation, with a potency and RE decrease of only 2-fold. Note that the β2S133V mutation is

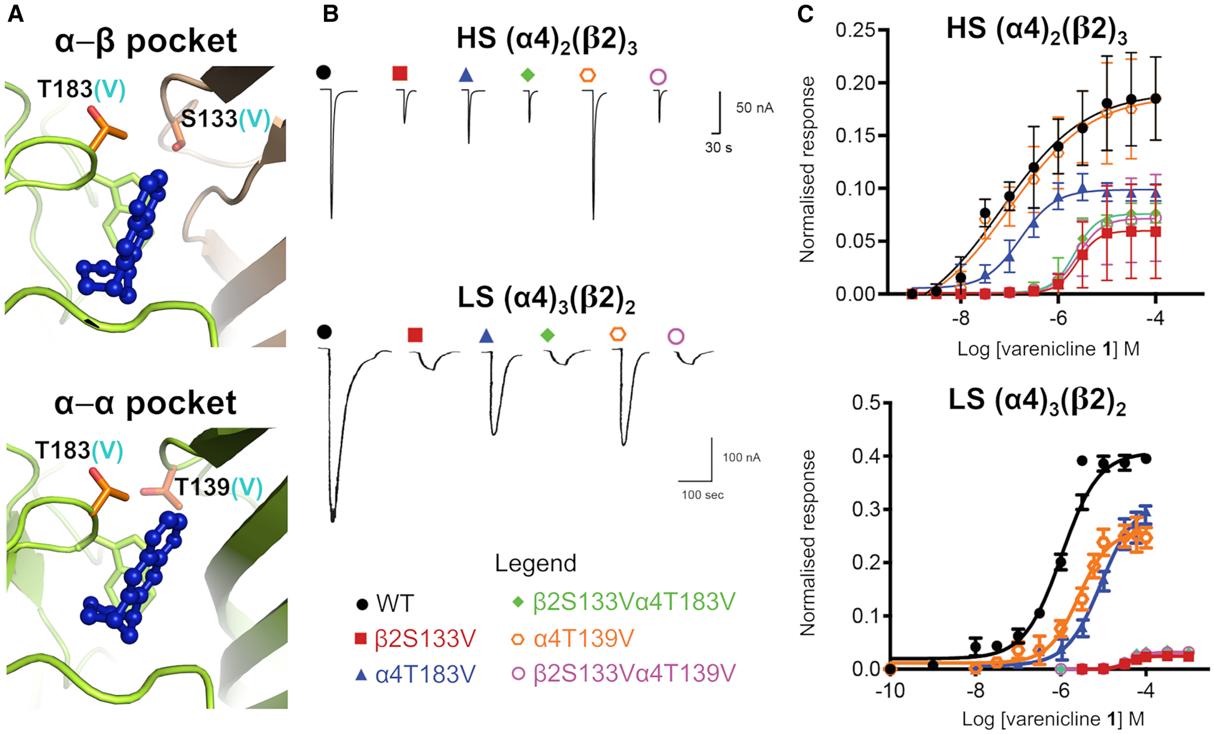

Figure 3. Functional effect of threonine-to-valine mutation with subsequent side-chain hydroxyl removal on varenicline 1 agonism at α4β2 nAChR

(A) Location of α4T183, α4T139, and β2S133 in the α-β (top panel) and α-α (bottom panel) binding pockets of the wild-type α4β2 receptor. Residues α4T183, α4T139, and β2S133 were replaced with valine, as indicated by the cyan-colored "(V)" in the residue labels, resulting in the three single mutants β2S133V, α4T183V, and α4T139V, and the two double mutants β2S133Vα4T183V and β2S133Vα4T139V. The α4 and β2 subunits are colored yellow and light brown, respectively. Varenicline **1** is highlighted in dark blue. The side chains of α4T183, α4T139, and β2S133 are represented by orange sticks, whereas TrpB is shown as yellow sticks.

(B) Representative current traces of the current responses elicited by varenicline **2** in oocytes expressing heterologously wild-type or mutant HS or LS α4β2 receptors. The current responses were concentration dependent. Full concentration-response curves are shown in (C).

(C) Varenicline **1** concentration-response curves for wild-type HS and LS isoforms (data shown in black) and corresponding mutants β2S133V (data shown in red), α4T183V (data shown in blue), β2S133Vα4T183V (data shown in green), α4T139V (data shown in orange), and β2S133V α4T139V (data shown in magenta). Data points in the concentration-response curves represent the mean ± SEM of 8–10 experiments using 6–8 different *Xenopus* donors. Current responses were measured using two-electrode voltage clamping from *Xenopus* oocytes heterologously expressing wild-type or mutant HS/LS α4β2 nAChRs. Peak current amplitudes for all agonists were normalized to the maximal ACh response (1 mM), as described in supplemental methods. Estimated potency (EC_{50}) and maximal relative efficacy (RE) parameters are shown in Table 1.

only present in the α-β binding pockets, whereas α4T183V affects both the α-β and α-α sites.

Within the α-α binding site, where α4T139 occupies a position homologous to that of β2S133 in the α-β agonist site, α4T139V reduced functional potency (by approximately 7-fold) and RE (by 50%) of varenicline **1** at the LS receptor. However, this mutation has no effect on agonist binding at the HS stoichiometry, i.e., α4T139 plays no role in the function of the orthosteric α-β site.

When β2S133V was co-expressed with α4T183V or α4T39V, the changes in potency and RE associated with varenicline **1** were generally no different from those of the single mutant β2S133, underscoring the essential functional role of the β2S133-varenicline interaction in α4β2 receptors.

As shown in Table 1 and Figures S36–S38, none of β2S133V, α4T183V, or α4T139V mutations had a significant impact on the agonist profiles of nicotine **2**, cytisine **3**, and ACh at either the HS or LS isoforms. This is consistent with our MD simulations showing that varenicline **1** is the only agonist that can approach closely the hydroxyl group of β2S133 and make a direct H bond with this residue (Figures 2B and S20).

To understand the structural and dynamic effect of the serine/threonine-to-valine mutations described above, MD simulations for the LS isoform incorporating the β2S133V, α4T183V, α4T139V, β2S133Vα4T183V, and β2S133Vα4T139V mutations with varenicline **1**, nicotine **2**, cytisine **3**, and ACh were carried out, with all complexes remaining stable throughout the simulation (Figures S7 and S8). To probe whether the mutations affect the dynamics of the receptor, C_α atom fluctuations were determined for both WT and mutant simulations, with all systems showing generally similar profiles, indicating comparable dynamics (Figure S10). Despite this, some regions (e.g., the Cys loop of the β2S133V complexes) showed discernible fluctuation differences compared to WT; however, these differences were not statistically significant (Figure S10).

The impact of the β2S133V, α4T183V, and α4T139V mutations on a range of other relevant interactions associated with ligand-receptor interactions was also assessed (Figures S17–S19 and S24–S26). In our mutant complexes, all agonists remained in their respective binding sites (Figures S11–S16), forming interactions with TrpB, TyrA, and TrpD, with varying frequencies depending on the mutant (Figures S17–S19). The only exception was the ACh molecule in the second α-β pocket of one β2S133Vα4T139V-ACh replicate, which exited the pocket after approximately 112 ns (Figure S13G).

An analysis of the distance between the H-bond acceptor groups within the agonists and H-bond donors present within the mutants' binding pockets was performed to evaluate how the mutations alter the pattern of interactions with the receptor. As anticipated, the serine/threonine-to-valine mutations redefined the agonist H-bond network within the binding pockets to varying extents (Figures S24–S26). For instance, in the β2S133V mutant, changes in H-bonding profiles were observed in the α-β pockets, with an increase in the frequency of interaction between α4T183 and cytisine **3** (Figure S24). In the α4T183V and α4T139V mutants, a significant increase in the interactions between the ligands and the backbone NH and side-chain OH group of α4T152 in the α-α pocket was observed, with this increase being especially pronounced for varenicline **1** and cytisine **3** (Figure S25). Furthermore, mutations involving α4T183 and α4T139 within the α-α pocket generally resulted in enhanced interactions between varenicline **1** and the side chain of α4Q150 but not α4H142 (Figure S26).

Exploring varenicline's structural features that contribute to binding and function in α4β2 nAChR: The role of the heteroaryl moiety

While the ammonium center of varenicline **1** enables the well-characterized cation-π and H-bond donor contacts with the receptor,[26] potential interactions (e.g., as an H-bond acceptor) involving the heteroaryl (quinoxaline) moiety of this ligand have also been identified from structural studies.[46,50,51,60] However, given the weakly basic nature of a quinoxaline (pK_a = 0.6 vs. the pyridyl moiety of nicotine **2**; pK_a = 3.1 [pK_a here refers to the pK_a of the protonated form of the base, i.e., pK_aH, and is used as defined in supplemental methods]), the functional contribution of any interaction involving this group remains unclear. To gain a more complete picture of the role of the quinoxaline moiety of varenicline **1**, we have explored the interactions involving the (hetero)aryl-based group in both receptor recognition (binding) and function (gating). Others, in particular the Pfizer group, have reported heteroaryl variants of varenicline.[28,37,77–79] However, these generally differ significantly in geometry/size and may incorporate aryl/heteroaryl cores or peripheral substituents capable of enabling additional/different ligand-receptor interactions, which complicates an assessment of the functional role played by the quinoxaline.

Here, we aimed to focus only on the heteroaryl region of varenicline **1** with two considerations guiding ligand design: (1) retain overall ligand size and shape, as well as the crucial cation-π/H-bond donor components (Figure 1D) within the protonated piperidine unit; and (2) have the ability to include or exclude, or vary, the location of an H-bond acceptor moiety within an otherwise conserved ligand scaffold. These considerations led to the design of three new ligands C_2 varenicline **4**, isovarenicline **5**, and N_2 varenicline **6**, as shown in Figure 4A. Importantly, all three ligands retained essentially the same geometry and volume as the parent compound, varenicline **1**, and did not present any additional or significant interactions associated with the structural periphery.

The synthetic chemistry involved is outlined in Scheme 1, and full details and compound characterization are available in supplemental methods. Each new ligand was isolated as an ammonium salt, which was then used for affinity binding and functional pharmacology studies (Tables 2 and 3).

For each variant **4–6**, we have assessed (1) ligand affinity constants (K_i) to the human WT α4β2 nAChR (as well as to the human α3β4 and α7 nAChR subtypes); (2) functional (full/partial agonist) profiles at the LS and HS α4β2 isoforms, and compared these to varenicline **1**, nicotine **2**, and cytisine **3**; (3) the effect of the aryl/heteroaryl moieties on the pK_a of the piperidine amine; and (4) using MD, the optimized binding modes of variants **4** and **5** and their dynamics in the LS and HS α4β2 isoforms relative to varenicline **1**.

(1) Given a requirement for initial recognition, ligand affinity constants for ligands **4** and **5** (together with varenicline **1**) to various nAChRs, including the human α4β2, are shown in Table 2. Comparative K_i data for human α3β4 and human α7 receptors have also been included, as varenicline also interacts with these subtypes. C_2 varenicline **4**, while weaker than the parent compound, has a K_i value within the nanomolar (nM) range and is most potent at the α4β2 subtype; further support for binding of **4** to the α4β2 nAChR comes from the inhibition of ACh by **4** (see Figure S39 and Table S2). A marked decrease in K_i was observed for isovarenicline **5**, and the relative differences observed at α4β2 for **4** and **5** are replicated at the human α3β4 and α7 subtypes. Pfizer assessed the benzo variant (2,3,4,5-tetrahydro-1H-1,5-methanobenzo[*d*]azepine) of naphthyl-based C_2 varenicline **4**, which had similar affinity at α4β2 (20 nM vs. 14.3 nM for **4**), although **4** showed higher affinity at α3β4 and α7 nAChR subtypes.[28] Binding affinity data for N_2 varenicline **6** could not be directly determined (see Table 2); however, this ligand's inability to bind was characterized indirectly.[32] Using α4β2 nAChRs expressed heterologously in *Xenopus* oocytes, N_2 varenicline **6** displayed very poor efficacy at both HS and LS α4β2 nAChRs (Table 3 and Figure 4B). Further, N_2 varenicline **6** does not affect ACh current response in oocytes heterologously expressing α4β2 nAChRs (Figure S39). Taken together, these data suggest that N_2 varenicline **6** does not bind appreciably to α4β2 nAChR.

(2) To determine the functional profile of the new varenicline variants, we have evaluated **1** and **4–6** against both the LS and HS stoichiometries of α4β2, with their concentration-response curves shown in Figure 4B and their EC_{50} and RE values in Table 3. These data show a similar trend to that associated with K_i, with similar patterns within the two α4β2 isoforms: C_2 varenicline **4** shows a (very) weak agonist profile, which reduces significantly further in the

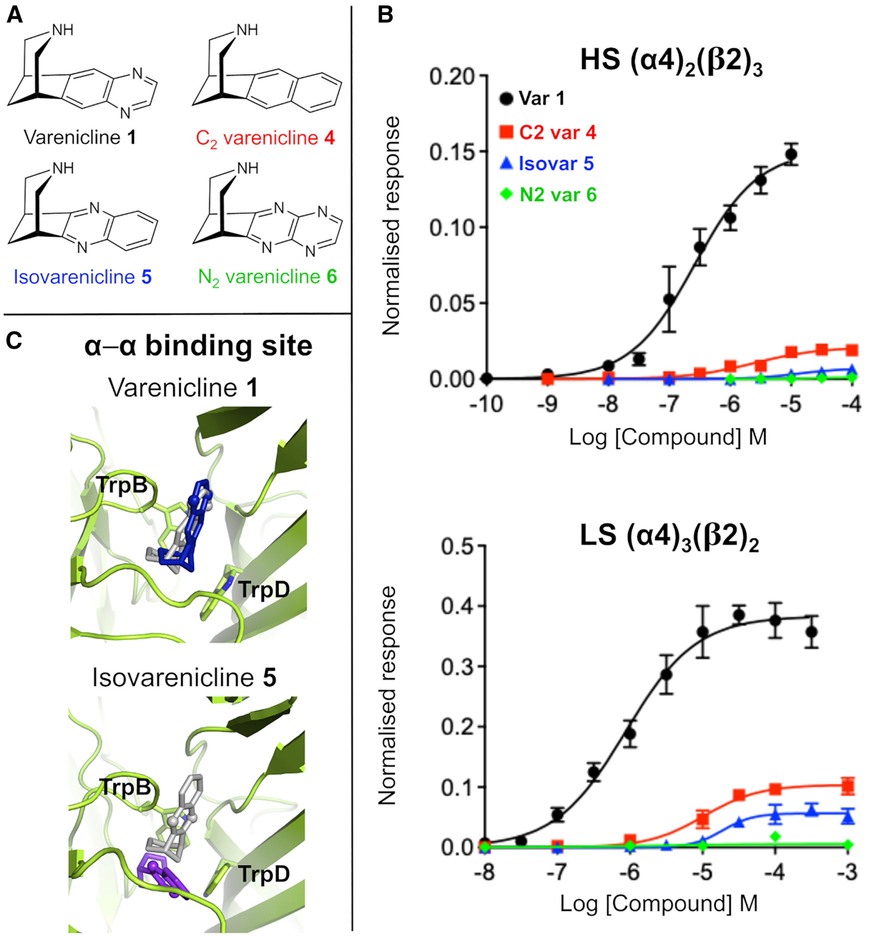

Figure 4. Functional profiles of varenicline 1 and variants 4–6

(A) Chemical structure of varenicline **1** and variants C_2 varenicline **4**, isovarenicline **5**, and N_2 varenicline **6**.

(B) Concentration-response curves for varenicline **1**, C_2 varenicline **4**, isovarenicline **5**, and N_2 varenicline **6** for the HS and LS α4β2 isoforms. Concentration-response curves for varenicline **1** and its variants were obtained as described in Figure 3 and supplemental methods. Estimated parameters EC_{50} and maximal relative efficacy (RE) are shown in Table 3.

(C) The different binding modes adopted by varenicline **1** and isovarenicline **5** in the α-α pocket after 300 ns of simulation. The gray sticks represent the starting binding mode (after energy minimization) for the agonists (see supplemental methods for a detailed description of how complexes were constructed). The final binding poses for varenicline **1** and isovarenicline **5** are colored blue and purple, respectively, with the nitrogen atoms of the quinoxaline moiety highlighted by spheres. TrpB and TrpD are shown as sticks.

case of isovarenicline **5**, and N_2 varenicline **6** is essentially inactive.

(3) Given the trends that emerged in Figure 4B and, in particular, the obvious discontinuity between the profiles of **1** and **4** vs. **5**, we have determined the pK_a values of the basic piperidinyl amine center. This was done using a spectrophotometric titration method (see supplemental methods and Figures S42–S49), with results shown in Table 4. As anticipated, the basicity of the ligands decreased in the following order: C_2 varenicline **4**, varenicline **1**, isovarenicline **5**, and N_2 varenicline **6**, correlating with an increasingly electron-deficient heteroarene. Amine protonation is a prerequisite for enabling both the cation-π and H-bond donor interactions (Figure 1D) with the conserved aromatic residues within the receptor's binding sites.[26] However, the range of pK_a values observed indicates that amine protonation at physiological pH should not be an issue for C_2 varenicline **4**, varenicline **1**, and isovarenicline **5** but may be relevant to N_2 varenicline **6** (see detailed discussion below).

(4) To assess the stability of the ligand-receptor complexes, models for the complexes between ECD of the WT HS and LS α4β2 isoforms and C_2 varenicline **4** and isovarenicline **5** were constructed and energy minimized. Given the extremely low level of activity and potency observed for ligand **6**, this system was not modeled. For each complex, one ligand molecule was placed in the α-α and α-β binding sites in poses corresponding to those observed in the cryo-electron microscopy (cryo-EM) structure of the human α4β2 nAChR-varenicline complex (PDB: 6UR8[60]). Energy minimized structures of the protonated variant **4** and **5** complexes show that both ligands were optimally located within the receptor's binding sites in a similar orientation as varenicline **1** (Figures S4 and S5).

Equilibrium MD simulations were performed to assess the dynamics of ligands **4** and **5** when bound to the WT HS and LS α4β2 nAChR (Figures S27 and S28). Like varenicline **1**, variants **4** and **5** remained bound to both the α-β and α-α pockets, forming a stable cation-π interaction with TrpB and sporadic interactions with TyrA and TrpD (Figures S27 and S29). Varenicline **1** and variant **4** exhibited limited mobility within the binding sites, consistently maintaining similar orientations throughout the simulation (Figures S30 and S31). However, variant **5** exhibited (1) notable differences in dynamics compared to varenicline **1** (Figures S27 and S30) and (2) varying levels of mobility between the α-β and α-α pockets, with the ligand displaying high mobility in the latter (Figure S30) and adopting binding modes that were different from the initial one (Figures 4D and S31).

Role of varenicline heteroarene for agonist profile at 5-HT_3 receptor

The agonist activity of varenicline **1** vs. the antagonist profile of cytisine **3** at 5-HT_3 receptors is a key characteristic differentiating these ligands.[34] Although a full analysis of varenicline's

CellPress
OPEN ACCESS

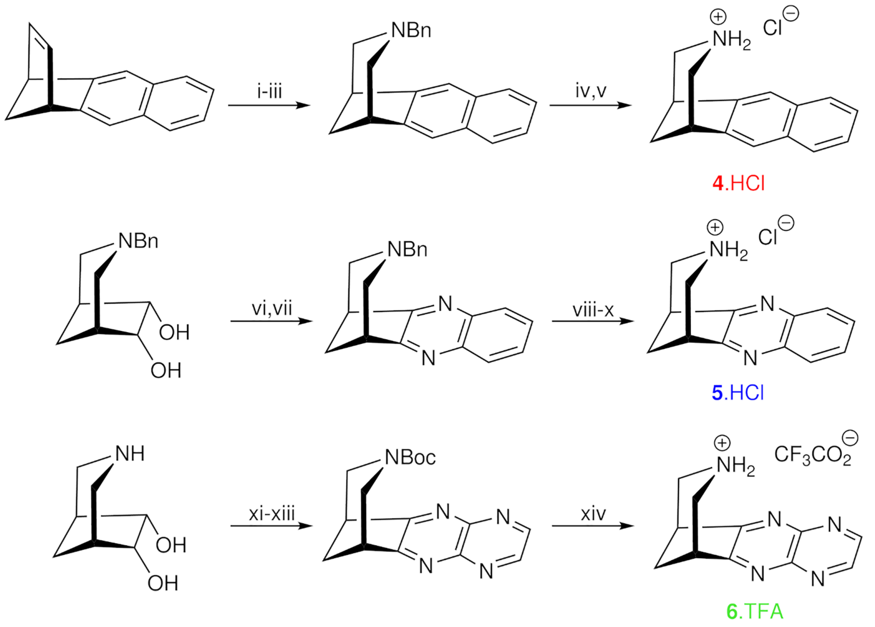

Scheme 1. Reagents

C_2 varenicline **4**: i, NMNO, OsO_4 (cat), acetone, water (98%); ii, $NaIO_4$, THF/water; iii, $NaBH(OAc)_3$, $BnNH_2$ (65% over 2 steps); iv, H_2, $Pd(OH)_2$ (20 wt % on C), Boc_2O, MeOH/EtOAc (74%); v, HCl in MeOH (quantitative). Isovarenicline **5**: vi, DCC, DMSO, Cl_2CHCO_2H, room temperature (rt), 20 h; vii, 1,2-phenylenediamine, rt, 18 h (70% over 2 steps); viii, chloroethyl chloroformate, $ClCH_2CH_2Cl$, 80°C, 18 h; ix, (A) MeOH, reflux 2 h (B) Boc_2O, rt, 18 h (86% over 2 steps); x, HCl in MeOH, quantitative. N_2 varenicline **6**: xi, Boc_2O, Na_2CO_3, THF/water, rt, 16 h (90%); xii, TFAA, DMSO, CH_2Cl_2, then Et_3N; xiii, 2,3-diaminopyrazine, MeOH, 65°C, 16 h (38% over 2 steps); xiv, 5% TFA in MeOH (69%).

interactions at 5-HT_3 receptors falls outside the scope of this study, we used variants **4–6** to assess the structural features of varenicline **1** that link to this profile.

The potency of variants **4–6** at 5-HT_3A was assessed using the receptor antagonist [^{3}H]GR65630. Compared to varenicline **1**, C_2 varenicline **4** showed a marked decrease in potency, and a similar trend was observed for both isovarenicline **5** and N_2 varenicline **6**, with half-maximal inhibitory concentration (IC_{50}) values (against [^{3}H]GR65630) increased by 44-, 165-, and 250-fold, respectively (Figure S40). Furthermore, none of the variants **4–6** elicited a significant functional response at concentrations up to 30 μM in cells expressing 5-HT_3A receptors (Figure S41).

DISCUSSION

An attractive pharmacological profile and strong track record as a smoking cessation agent[41] made cytisine **3** an attractive lead for the development of a proprietary therapeutic, which in 2006 led to the launch of varenicline **1** (Chantix).[17,18–20] However, accumulating evidence now suggests that these two ligands are perhaps not as closely related as initially thought, particularly in terms of their functional mechanisms.[29,32,34,46] While structurally similar, this relationship may be superficial, and pharmacologically varenicline **1** and cytisine **3** should be considered as "cousins" rather than "siblings." Auerbach and co-workers have recently shown that varenicline **1** stands out as the first ligand in the "low-efficiency" category (η = 33%), whereas typical nAChR ligands, such as ACh, nicotine **2**, and cytisine **3** (as well as anatoxin-a, epibatidine, and epiboxidine) usually show higher efficiencies (η = 41%–51%).[83] Efficiency (η), which is a measure of the proportion of binding energy converted to gating, likely reflects the relationship between ligand structure and the specifics of the receptor-ligand interactions that drive binding and receptor activation.[84,85]

Structurally, varenicline **1** and cytisine **3** both feature a rigid bicyclic core incorporating a piperidyl unit; however, the adjacent heteroaryl moieties (quinoxaline and 2-pyridone, respectively) are quite different. Functionally, these two molecules are also distinct; for instance, although both ligands have similar full agonist profiles at the α7 nAChR,[29,32] their profiles differ at the α4β2 subtype, mainly at the HS isoform.[29,32] While varenicline **1** acts as a weak partial agonist of the HS isoform, achieving approximately 13%–18% of the maximum efficacy relative to ACh, cytisine **3** is essentially inactive (Table 1).[29,32] Another differentiating feature is that varenicline **1** is a potent agonist at the human 5-HT_3 serotonin receptor, with an efficacy of 80% relative to 5-HT (serotonin), whereas cytisine **3** is an antagonist. While the pharmacological requirements for an effective smoking cessation drug (e.g., roles of the HS vs. LS isoforms; profile at α7) are still to be fully elucidated,[86,87] off-target interactions (and associated side effects), such as binding to the 5-HT_3 receptor, are a factor in reducing end-user compliance.[88,89] Therefore, understanding the structure and dynamic basis underpinning functional differences between related ligands, such as varenicline **1** and cytisine **3**, and various receptors is paramount. The work of Dougherty, Lester, and co-workers has demonstrated that, at the molecular level, varenicline **1** does not utilize the same critical set of three receptor-ligand interactions as nicotine **2** or the related compound cytisine **3** to enable receptor activation.[25,26,49] Based on an insightful and selective modification of the protein scaffold, Dougherty showed that, unlike nicotine **2** and cytisine **3**, varenicline **1** does not make a functional interaction with β2Leu146 (via the backbone NH).[49] This important finding prompted our studies and led us to seek and identify alternative interaction(s) to that "missing" third component associated with varenicline **1** function.

Our efforts have aimed to provide a comprehensive description of the receptor-ligand interaction patterns involving varenicline **1** in order to enhance our understanding of the mode of action of this ligand and ultimately to shed light on the differences associated with varenicline **1** vs. cytisine **3**, such as, e.g., profiles at HS vs. LS 4β2 isoforms and at the 5-HT_3 receptor. To this end, we have employed a multidisciplinary strategy based on known

Table 2. Binding affinity constants K_i for varenicline 1 and ligands 4 and 5

	K_i				
Ligand	α4β2 (nM)	α3β4 (nM)	α3β4/α4β2	α7 (nM)	α7/α4β2
Varenicline **1**	0.46 ± 0.12	171 ± 41	372	75 ± 22.5	163
C_2 varenicline **4**	14.3 ± 1.6	267 ± 73	18.7	1,325 ± 595	93
Isovarenicline **5**	5,460 ± 1,280 (5.46 μM)	67,700 ± 37,600 (67.7 μM)	12	126,800 ± 86,000 (126.8 μM)	23

The K_i values for the human α4β2, α3β4, and α7 nAChRs (as well as ratios relative to α4β2) are shown. The K_i value for N_2 varenicline **6** was not determined, due to disruption (and subsequent closure) of the Milan lab owing to the COVID-19 pandemic.

and novel ligands, integrating biomolecular modeling and simulation; chemical synthesis and physiochemical characterization; receptor binding; and comprehensive functional studies.

Using the experimental structural data available, we constructed models for the complexes between the ECDs of the LS and HS isoforms of the human α4β2 nAChR and varenicline **1**, nicotine **2**, cytisine **3**, and ACh (Figures 2A, S2, and S3). This was followed by extensive MD simulations to uncover potential new functional interactions involving the agonists, particularly varenicline **1**, within the α-β and α-α binding pockets of the α4β2 nAChR. The simulations revealed two new potential interactions associated with the H-bond hydroxyl donor in the side chain of α4T183 and β2S133 within the principal and complementary faces of the α-β binding site (Figure 2B). In the simulations, these residues can form transient H bonds with some ligands, including varenicline **1** and nicotine **2**. Note that, unlike α4T183 (which is present on the principal side of both binding sites), the β2S133 on the complementary face of the α-β site is replaced by α4T139 in the α-α pocket (Figure 1C). These three residues all had the potential to interact with the quinoxaline moiety of varenicline **1** and, thereby, provide a (necessary) third H-bond (receptor donor/ligand acceptor) interaction (in addition to the cation-π and H bond associated with the ammonium center of the ligand) characterized in the Dougherty-Lester model (Figure 1D). The interactions with α4T183, β2S133, and/or α4T139 also require participation of the ligand (as the H-bond acceptor), and, using synthetic chemistry, we have explored further those structural features of varenicline **1** that mediate its binding and function.

Targeted mutagenesis, together with electrophysiological assays, allowed us to assess the relative importance of each of these hydroxyl-containing residues across ACh and varenicline **1** as well as nicotine **2** and cytisine **3**. For this, the polar residues α4T183, β2S133, and α4T139 were mutated to valine, thereby eliminating their side-chain hydrogen-bonding potential. Note that by removing the hydroxyl group (an H-bond donor) from their side chains, the serine/threonine-to-valine mutations prevent α4T183, β2S133, and α4T139 from participating in H-bond interactions with the ligands and/or water molecules. However, the backbone amide NH in these residues, which also serves as an H-bond donor, may still engage in hydrogen bonding. The profiles of varenicline **1**, nicotine **2**, cytisine **3**, and ACh were then determined in the mutant receptors (Figures 3 and S34–S38; Table 1). While the α4T139V and β2S133V mutations, both situated on the α4/β2 complementary face, affect the α-α and the α-β sites individually, α4T183V impacts all binding sites simultaneously due to its position on the principal side of the pocket (Figure 1C).

These studies (Figure 3 and Table 1) demonstrated that, overall, none of the mutations introduced (whether individually or in clusters) affect the ACh profile at either the HS or LS isoforms of the α4β2 receptor. This confirms that α4T183, β2S133, and α4T139 do not play a role in ACh function and further supports Dougherty's observation that β2L146 (or α4T152 in the α-α pocket) likely accounts for the receptor H-bond donor interaction with ACh.[25,26]

In contrast to the scenario described above for ACh, varenicline **1** exhibited a significantly different response at the β2S133V mutant, displaying a marked reduction in relative efficacy and showing a 3-fold and 21-fold decrease at the HS and LS β2S133V mutant, respectively, along with concomitant shifts toward higher EC_{50} values (Figure 3 and Table 1). Note that at the HS WT receptor, varenicline **1** is already a relatively weak partial agonist, with an efficacy of only 18% relative to ACh; however, upon introducing the serine-to-valine (β2S133V) mutation in the complementary face of the α-β sites, varenicline **1** shows negligible efficacy (Table 1).

Additionally, the impact of the α4T183V and α4T139V mutations on the functional profile of varenicline **1** was generally less pronounced than that observed for the β2S133V mutation. At the α4T183V receptor, varenicline **1** had a modest loss of efficacy and potency, with about a 2-fold and 6-fold decrease, respectively, at the HS and LS isoforms (Table 1). For the HS form of the α4T139V nAChR, as expected, no effect on the functional profile of varenicline **1** (or any other ligands for that matter) was observed, as this isoform lacks an α-α site; in contrast, the α4T139V mutation had a moderate effect on the efficacy and potency of varenicline **1** in LS isoform, with a level of reduction similar to that observed for the LS α4T183V mutant (Table 1). At the two double mutants, β2S133Vα4T183V and β2S133Vα4T139V, varenicline **1** exhibited again a dramatic reduction in its functional profile (both efficacy and potency), with the magnitude of the changes generally on the same order as those observed in the single-point β2S133V mutant (Table 1).

Based on all the data presented above, we conclude that β2S133 (rather than α4T183 or α4T139), via the H bond formed by its side-chain hydroxyl group (as opposed to the backbone NH, which is still present in the mutants), is the dominant and (up until now unidentified) "third component" required to mediate the function of varenicline **1**. Regarding the roles of β2S133, α4T183, and α4T139 in the function of nicotine **2** and cytisine **3**, the situation is less clear than for varenicline **1**. The effects of the β2S133V, α4T183V, and α4T139V mutations, when present, are markedly more muted compared to those observed for varenicline **1** (Table 1). However, for both nicotine **2** and cytisine **3**, the most impactful interaction still appears to be associated with

CellPress
OPEN ACCESS

Table 3. Agonist sensitivity of HS and LS α4β2 isoforms to varenicline 1, C_2 varenicline 4, isovarenicline 5, and N_2 varenicline 6

	HS $(\alpha4)_2(\beta2)_3$		LS $(\alpha4)_3(\beta2)_2$	
Ligand	EC_{50} (μM)	RE	EC_{50} (μM)	RE
Varenicline **1**	0.286 ± 0.1	0.14 ± 0.012	1.01 ± 0.4	0.38 ± 0.025
C_2 varenicline **4**	2.64 ± 0.8[a]	0.022 ± 0.0033[a]	11.50 ± 1.74[a]	0.102 ± 0.012[a]
Isovarenicline **5**	13.27 ± 2.5[a]	0.0071 ± 0.00086[a]	26 ± 8.5[a]	0.054 ± 0.013[a]
N_2 varenicline **6**	ND	0.0010 ± 0.0001[a]	ND	0.0032 ± 0.0023[a]

Potency (EC_{50}) and relative efficacy (RE) were determined as described in Table 1 and supplemental methods. Statistical differences between varenicline **1** and ligands **4**, **5** and **6** were determined as described in Table 1. ND, not determined due to low levels of functional expression (less than 50 nA of ACh maximal currents). These data are also presented in a graphical format in Figure 4B.
[a]Statistically significant difference ($p < 0.05$) between varenicline **1** and ligands **4**, **5**, or **6**.

β2S133, although the changes in the ligand functional profiles in the β2S133V-containing mutants were modest (Table 1). We interpret these results as an ability of nicotine **2** and cytisine **3** to access β2S133 in addition to (but not to the exclusion of) β2L146, which nevertheless remains the optimal partner for mediating the functional profiles of these two ligands. A potential role for β2S133 in mediating the binding of a series of 2-(2-pyrrolidinyl)-1,4-benzodioxane ligands was suggested previously[90]; however, in this case, the role of β2S133 was limited to ligand recognition, with the corresponding β2S133A mutation shown to have no effect on function.

As stated above, the α4T139V mutation, which is the residue associated with the complementary face of the α-α binding site, unsurprisingly had no effect on the functional profiles of the ligands studied at HS receptor isoform and, at best, showed only a minimal impact at the α-α-containing LS stoichiometry, depending on the ligand used (Table 1). This is consistent with the findings of, for example, Balle and co-workers, who have previously shown that α4T139A has only a modest impact on the maximal efficacy of NS9283.[74]

If we consider that, as with the α-β site, a third anchoring connection is required to potentiate activity via the α-α site, then the residue(s) responsible for mediating this interaction remain unclear. The α-α and α-β binding sites are structurally different on the complementary face, as the former is formed by an α4 subunit and the latter by a β2 subunit, with these differences resulting in distinct pharmacological profiles for each site.[22,67] Examples of these differences include the substitution of β2S133, β2V136, β2F144, and β2L146 located in loop E of the α-β site by α4T139, α4H142, α4Q150, and α4T152 in the α-α site, respectively.[49,67,70,71] The MD simulations performed in this study indicate that the hydroxyl moiety of α4T152 in the α-α site can directly interact with the H-bond acceptor groups of certain agonists (Figure S25), therefore suggesting that this residue may potentially serve as the third interaction in this binding site. Previous experimental studies have demonstrated that the α4T152A and α4T152V mutations can decrease the activity of NS9283 several-fold,[74] supporting the hypothesis that α4T152 can indeed sustain interactions with some ligands. Additionally, given that α-α site residues, such as α4W88 and α4H142, have been identified as key for the gating efficiency of the LS form of the α4β2 receptor,[52,74] it would be prudent to say that the precise role of α4T152 in modulating the functional profile of different agonists requires further investigation.

The structure of varenicline **1** and its comparison to cytisine **3** also merit comment. As explained above, the bicyclic piperidine moiety is common to both varenicline **1** and cytisine **3**, but their adjacent heteroaryl units, quinoxaline and 2-pyridone, respectively, differ in terms of the spatial relationships associated with these H-bond acceptor elements. However, given the relatively non-basic nature of the quinoxaline moiety, to comprehend how varenicline **1** exerts its action, two fundamental questions must be addressed: (1) is the quinoxaline unit, as present in varenicline **1**, required for both binding and function, or do those characteristics rely primarily on rigidity and overall shape?; and (2) if the quinoxaline group is essential for function, what evidence is available to support the role of the quinoxaline as an H-bond acceptor within the context of nAChRs?

To answer these questions, we have designed a targeted set of novel varenicline variants, namely C_2 varenicline **4**, isovarenicline **5**, and N_2 varenicline **6**. These new ligands, which all incorporate a basic piperidine center to retain the critical cation-π and H-bond donor characteristics, maintain the same size, shape, and molecular volume as the parent compound, varenicline **1** (Figure 4A). This enabled us to avoid added complications associated with additional peripheral substituents to probe receptor-ligand recognition and function and the role played by the aryl moiety in these two connected processes. C_2 varenicline **4**, which differs from varenicline **1** in containing a naphthalene (lacking an H-bond acceptor) instead of a quinoxaline group (Figure 4A), displays a K_i of 14.3 nM, corresponding to an approximately 30-fold decrease in affinity compared to the parent compound (Table 2). In terms of function, variant **4** is, however, a very weak partial agonist showing a 10-fold increase in EC_{50} at both HS and LS isoforms of α4β2, together with reduced efficacy, with a 6- and 4-fold reduction at HS and LS, respectively (Table 3). We conclude from these results that while the quinoxaline moiety (as present in varenicline) enhances binding, it is not a prerequisite, with size, rigidity, and shape (and the interactions associated with the ammonium center) being primary determinants for recognition. Further, the MD simulations performed suggest that when bound to the α4β2 nAChR, variant **4** exhibits comparable dynamics and adopts similar binding modes to those of varenicline **1** (Figures S30 and S31), with the biggest differences between the two ligands arising from the lack of the H bonds associated with the naphthalene group. However, the presence of a quinoxaline unit within varenicline **1** is an essential requirement for function (Table 3), therefore

Table 4. Experimentally determined pK_a values for the piperidine amine center of varenicline 1, C_2 varenicline 4, isovarenicline 5, and N_2 varenicline 6

Ligand (salt used)	pK_a	Literature values
Varenicline **1** (tartrate$^-$)	8.90 ± 0.1	9.2 (Pfizer[80]), 9.3 (Rollema et al.[81]), 9.22 ± 0.13 (Unal et al.[82])
C_2 varenicline **4** (Cl^-)	9.63 ± 0.08	–
Isovarenicline **5** (Cl^-)	8.44 ± 0.09	–
N_2 varenicline **6** ($CF_3CO_2^-$)	7.31 ± 0.05	–

The specific salt involved is indicated (based on the final deprotection method used in Scheme 1), and commercially available varenicline tartrate was used.

showing that this structural component contributes to additional interaction(s) unavailable to C_2 varenicline **4**. In this regard, it is noteworthy that C_2 varenicline **4** exhibits an identical pattern of functional responses at both the HS WT and mutant β2S133V/α4T183V receptors (Tables 1 and 2). Both C_2 varenicline **4** and these receptor variants preserve the capacity to form cation-π and hydrogen-bond interactions but lack the distinctive third interaction characteristic of varenicline **1**. This observation supports the conclusion that the quinoxaline moiety in varenicline mediates an additional, functionally critical interaction that is absent in variant **4**. For the LS isoform, where the α-α binding site present is not subject to mutation, EC_{50} values are, nevertheless, still very similar.

In the case of isovarenicline **5**, where the orientation of the quinoxaline moiety has been reversed (Figure 4A), a step change in both binding and function at α4β2 was observed (Tables 2 and 3). Variant **5** binds very weakly to α4β2 (high μM, which was also observed at both the α3β4 and α7 subtypes) and also shows markedly reduced efficacy, with a 20-fold and 7-fold decrease at HS and LS isoforms, respectively. MD simulations associated with variant **5** align with these findings, revealing the ligand to be less stable when bound to α4β2, adopting configurations markedly different from varenicline **1** (Figures 4C, S30, and S31) and, importantly, forming alternative interaction networks within the binding pockets (Figures S32 and S33). A clear distinction between the interaction networks for varenicline **1** vs. isovarenicline **5** is the loss of H bonding to the side chain of β2S133 and the emergence of a frequent and stable interaction with the side chain of α4T152 in the α-α pocket (Figures S32 and S33). As discussed, the interaction between varenicline **1** and β2S133 is essential for the ligand functional activity (Table 1), and its absence for variant **5** likely contributes to its diminished efficacy. Taken together, the experimental and computational findings allow us to answer the first of the two questions posed above, namely, whether the quinoxaline in varenicline **1** is necessary for binding and function. Our results indicate that both the incorporation and the specific orientation of the quinoxaline unit within varenicline **1** is essential for function.

The results reported here for C_2 varenicline **4** and isovarenicline **5** also shed light on the second question raised above regarding the role of the quinoxaline as an H-bond acceptor. Our findings demonstrate that H bonding, either direct or water mediated, between the quinoxaline group and the protein is crucial for varenicline function. Further compelling experimental evidence that this key third varenicline **1** interaction is based on quinoxaline acting as an H-bond acceptor comes from crystallographic data for the *Capitella teleta* AChBP-varenicline **1** complex (PDB:4AFG),[51] a complex analogous with that of the serotonin binding protein from *Aplysia californica* (PDB: 5AIN)[46] as well as the structure of varenicline **1** bound to the iCytSnFR cytisine sensor precursor binding protein (PDB: 7S7X).[91] Although they are not functionally significant, in all of these cases, water (and also tyrosine)-varenicline **1** interactions involving the quinoxaline N atoms of varenicline are evident. Furthermore, our findings underscore the importance of the precise spatial positioning of the quinoxaline unit in varenicline **1**, as its interactions with the protein must occur within a specific, well-defined region of the binding site. Altering the orientation of the quinoxaline group (as in isovarenicline **5**) substantially disrupts this interaction network and, subsequently, receptor function.

N_2 varenicline **6** incorporates a 1,4,6,8-tetraazanaphthalene moiety, which we anticipated to be much less available as an H-bond acceptor than the quinoxaline of varenicline **1** (Figure 4A).[92] Variant **6** showed no evidence of binding (Figure S39), nor was activity detected at either HS or LS isoforms (Table 3). A likely explanation for this lack of binding is, we suggest, related to the pK_a of the piperidine group in N_2 varenicline **6** (Table 4). Given that piperidine protonation in varenicline **1** is necessary for the cation-π and H-bond donor/acceptor interactions with TrpB in the principal side of the α-β pockets,[26] the affinity constant (K_i) and pK_a values for varenicline **1** and variants **4–6** were determined (Tables 2 and 4, respectively). A significant decrease in K_i was observed between C_2 varenicline **4** (pK_a = 9.63) and isovarenicline **5** (pK_a = 8.44), despite no meaningful differences in the protonation state of their ammonium centers at physiological pH; both ligands remain ≥90% protonated at pH 7.5. Moreover, isovarenicline **5** is more basic than either nicotine **2** (pyrrolidine pK_a = 7.80)[93] or cytisine **3** (pK_a = 8.20),[94] further supporting that protonation differences are not linked to reduced binding affinity. Rather, the functional profile of variant **4** is attributed to its inability to act as an H-bond acceptor. In contrast, the decrease in functional profile behavior of isovarenicline **5** is associated with distinct interaction patterns that it forms within the binding sites compared to varenicline **1**. These encompass the formation of contacts with α4T152 and the loss of those involving β2S133 (Figures S32 and S33). This finding highlights the importance of the precise H-bond acceptor position within varenicline **1**, where an interaction with β2L146 is precluded by distance, yet the ligand can compensate by engaging in an alternative functional mechanism via β2S133.

Finally, the very poor H-bond acceptor characteristics of the 1,4,5,8-tetraazanaphthyl group in N_2 varenicline **6** would suggest a functional profile resembling that of naphthyl-based C_2 varenicline **4**. However, this is not the case, with the inactive profile of variant **6** likely associated with the low pK_a of the piperidine amine (pK_a = 7.31), indicating that protonation may be impaired.[95]

Given that the viability of varenicline **1** to participate as an H-bond acceptor within similar receptor binding environments

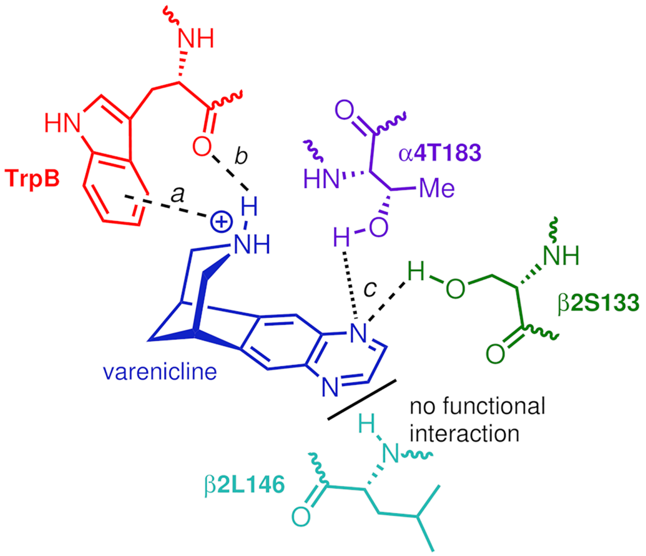

Figure 5. Expanded version of the Dougherty-Lester nAChR binding model for varenicline 1 in the α-β pocket of α4β2 nAChR, highlighting the three key functional interactions

(a) cation-π and (b) backbone C=O as H-bond acceptor associated with TrpB within the α4 subunit,[23–25] and (c) the newly identified H-bond donor-acceptor interaction between the quinoxaline moiety of varenicline **1** and the side-chain OH group of β2S133 (in the complementary subunit). The less prominent interaction involving the OH of α4T183 is also included in the scheme, as it can occur in the absence of a functional connection involving the backbone NH of β2L146. Note that although the new ligand-protein contacts involving β2S133 and α4T183 are depicted as being direct, the possibility of a bridging water molecule mediating these interactions cannot be excluded.

is now firmly established, and based on the data for the mutants above (Table 1), we propose that the key interactions of the quinoxaline moiety of varenicline **1** involve β2S133 in the complementary side and (to a lesser degree) α4T183 in the principal side of the α-β binding sites within the α4β2 nAChR. Furthermore, building on previous findings by Dougherty and colleagues showing that varenicline **1** does not rely on β2L146 to enable function,[49] we posit that the binding model of varenicline **1** to the α-β sites of the α4β2 subtype, which retains both the cation-π and H-bond donor components derived from the protonated secondary amine center in the ligand, can now be expanded to include an H-bond donor-acceptor interaction (as the "missing" third component) and that this previously unrecognized interaction involves the quinoxaline heterocycle with chiefly β2S133, as illustrated in Figure 5.

We have also probed the ligand requirements of the 5-HT_3 receptor: compared to varenicline **1**, ligands **4–6** exhibit far weaker interactions and fail to activate 5-HT_3 (Figures S40 and S41). From these findings, we conclude that the precise location of the quinoxaline moiety within the varenicline scaffold is again a critical determinant of varenicline's profile at 5-HT_3 receptors.

In conclusion, we have characterized the "missing interaction" associated to date with modulation of the partial agonist profile of varenicline **1** at the α4β2 receptor, the primary target in nicotine addiction. We have demonstrated that the quinoxaline moiety within varenicline **1** engages in functionally relevant interactions with α4β2 nAChR. In its α-β binding sites, these interactions are highly specific and well defined, involving the hydroxyl side chains of primarily β2S133 and (to a lesser degree) α4T183. Given that H-bond donor groups at positions equivalent to β2S133 and α4T183 are highly conserved across human neuronal nAChR subunits, it is plausible to suggest that these newly characterized interactions contribute to the functional profile of varenicline **1** (and potentially other agonists) across a range of neuronal subtypes, such as α7 and α3β4. This, in our opinion, warrants further investigation, as uncovering the functional interactions of each agonist will strengthen the foundation for rational drug design targeting these proteins. The ability of varenicline **1** to exploit alternative and functionally relevant binding patterns within neuronal receptors, distinct from that of its progenitor cytisine **3**, may directly account for the divergence in functional profiles observed for these two ligands. This is particularly relevant for the human 5-HT_3 receptor, where cytisine **3** is 2,000-fold less potent than varenicline **1**.[96]

METHODS

Full details of synthetic chemistry, computational chemistry, nAChR ligand binding, nAChR functional studies, 5-HT_3 functional studies, and pK_a determinations are provided in supplemental methods.

RESOURCE AVAILABILITY

Lead contact

Requests for further information and resources should be directed to and will be fulfilled by the lead contact, A. Sofia F. Oliveira (sofia.oliveira@bristol.ac.uk).

Materials availability

Subject to availability, ligands **4**, **5**, and **6** generated in this study are available upon request from Timothy Gallagher (t.gallagher@bristol.ac.uk).

Data and code availability

All MD data (including input and trajectory files) are publicly available via the University of Bristol Research Data Repository (https://data.bris.ac.uk/).

ACKNOWLEDGMENTS

A.S.F.O. was supported at the University of Bristol by a BBSRC Discovery Fellowship (BB/X009831/1). F.V. was supported by ANID Becas Chile 72210124. We thank Achieve Life Sciences for a gift of (−)-cytisine and EPSRC (EP/N024117/1) for financial support. T.G. thanks Dr. Jack Rogers for support and Professor Varinder Aggarwal for access to laboratory facilities at the University of Bristol. We thank EPSRC for providing ARCHER2 time via HECBioSim (hecbiosim.ac.uk). Data analysis was conducted using the facilities of the Advanced Computing Research Center at the University of Bristol (https://www.bris.ac.uk/acrc/).

AUTHOR CONTRIBUTIONS

A.S.F.O. and T.C.G. conceptualized the project. S.G.A., D.F., M.H., G.P., and T.G. performed the synthetic chemistry. J.M., J.R., and A.C.O.'D. carried out the pKa determinations. A.S.F.O. conducted the molecular dynamics simulations, and both A.S.F.O. and J.U. performed the analysis. C.G. performed the nACh receptor binding experiments, and I.B., F.V., and T.M.V. carried out the nACh receptor functional assays and functional data analysis. S.C.R.L.

performed the 5-HT_3 receptor functional assays and functional data analysis. All authors contributed to the preparation of the manuscript.

DECLARATION OF INTEREST

The authors declare no competing interests.

SUPPLEMENTAL INFORMATION

Supplemental information can be found online at https://doi.org/10.1016/j.xcrp.2025.102992.

Received: August 12, 2025
Revised: October 26, 2025
Accepted: November 11, 2025
Published: December 9, 2025

REFERENCES

1. World Health Organization. WHO report on the global tobacco epidemic, 2023; 2023. https://iris.who.int/bitstream/handle/10665/372043/9789240077164-eng.pdf?sequence=1.
2. GBD 2019 Tobacco Collaborators (2021). Spatial, temporal, and demographic patterns in prevalence of smoking tobacco use and attributable disease burden in 204 countries and territories, 1990-2019: a systematic analysis from the Global Burden of Disease Study 2019. Lancet *397*, 2337–2360.
3. Taylor, A.L., and Bettcher, D.W. (2000). WHO Framework Convention on Tobacco Control: a global "good" for public health. Bull. World Health Organ. *78*, 920–929.
4. Mathers, C.D., and Loncar, D. (2006). Projections of global mortality and burden of disease from 2002 to 2030. PLoS Med. *3*, e442.
5. Beard, E., Shahab, L., Cummings, D.M., Michie, S., and West, R. (2016). New Pharmacological Agents to Aid Smoking Cessation and Tobacco Harm Reduction: What Has Been Investigated, and What Is in the Pipeline? CNS Drugs *30*, 951–983.
6. Lang, A.E., and Berlin, I. (2023). Unavailability of varenicline: a global tragedy for the fight against the tobacco epidemic. Lancet Respir. Med. *11*, 518–519.
7. Rey Brandariz, J., Rumgay, H., Ayo-Yusuf, O., Edwards, R., Islami, F., Liu, S., Pérez-Ríos, M., Rodrigues Pinto Corrêa, P.C., Ruano-Ravina, A., and Soerjomataram, I. (2024). Estimated impact of a tobacco-elimination strategy on lung-cancer mortality in 185 countries: a population-based birth-cohort simulation study. Lancet Public Health *9*, e745–e754.
8. Berry, K.M., Fetterman, J.L., Benjamin, E.J., Bhatnagar, A., Barrington-Trimis, J.L., Leventhal, A.M., and Stokes, A. (2019). Association of Electronic Cigarette Use With Subsequent Initiation of Tobacco Cigarettes in US Youths. JAMA Netw. Open *2*, e187794.
9. Hajek, P., Phillips-Waller, A., Przulj, D., Pesola, F., Myers Smith, K., Bisal, N., Li, J., Parrott, S., Sasieni, P., Dawkins, L., et al. (2019). A Randomized Trial of E-Cigarettes versus Nicotine-Replacement Therapy. N. Engl. J. Med. *380*, 629–637.
10. Action on Smoking and Health. Use of e-cigarettes among adults in Great Britain. 2024. https://ash.org.uk/resources/view/use-of-e-cigarettes-among-adults-in-great-britain.
11. Jackson, S.E., Shahab, L., Tattan-Birch, H., and Brown, J. (2024). Vaping among adults in England who have never regularly smoked: a population-based study, 2016-24. Lancet Public Health *9*, e755–e765.
12. Robichaud, M.O., Seidenberg, A.B., and Byron, M.J. (2020). Tobacco companies introduce 'tobacco-free' nicotine pouches. Tob. Control *29*, e145–e146.
13. M Jackson, J., Weke, A., and Holliday, R. (2023). Nicotine pouches: a review for the dental team. Br. Dent. J. *235*, 643–646.
14. Benowitz, N.L. (2009). Pharmacology of nicotine: addiction, smoking-induced disease, and therapeutics. Annu. Rev. Pharmacol. Toxicol. *49*, 57–71.
15. Dani, J.A. (2015). Neuronal Nicotinic Acetylcholine Receptor Structure and Function and Response to Nicotine. Int. Rev. Neurobiol. *124*, 3–19.
16. Wittenberg, R.E., Wolfman, S.L., De Biasi, M., and Dani, J.A. (2020). Nicotinic acetylcholine receptors and nicotine addiction: A brief introduction. Neuropharmacol *177*, 108256.
17. Cahill, K., Lindson-Hawley, N., Thomas, K.H., Fanshawe, T.R., and Lancaster, T. (2016). Nicotine receptor partial agonists for smoking cessation. Cochrane Database Syst. Rev. *18*, CD006103.
18. Jordan, C.J., and Xi, Z.X. (2018). Discovery and development of varenicline for smoking cessation. Expert Opin. Drug Discov. *13*, 671–683.
19. Coe, J.W., Brooks, P.R., Vetelino, M.G., Wirtz, M.C., Arnold, E.P., Huang, J., Sands, S.B., Davis, T.I., Lebel, L.A., Fox, C.B., et al. (2005). Varenicline: An alpha 4 beta 2 nicotinic receptor partial agonist for smoking cessation. J. Med. Chem. *48*, 3474–3477.
20. Livingstone-Banks, J., Fanshawe, T.R., Thomas, K.H., Theodoulou, A., Hajizadeh, A., Hartman, L., and Lindson, N. (2023). Nicotine receptor partial agonists for smoking cessation. Cochrane Database Syst. Rev. *2023*, CD006103.
21. Hurst, R., Rollema, H., and Bertrand, D. (2013). Nicotinic acetylcholine receptors: from basic science to therapeutics. Pharmacol. Ther. *137*, 22–54.
22. Walsh, R.M., Jr., Roh, S.H., Gharpure, A., Morales-Perez, C.L., Teng, J., and Hibbs, R.E. (2018). Structural principles of distinct assemblies of the human alpha4beta2 nicotinic receptor. Nature *557*, 261–265.
23. Cashin, A.L., Petersson, E.J., Lester, H.A., and Dougherty, D.A. (2005). Using physical chemistry to differentiate nicotinic from cholinergic agonists at the nicotinic acetylcholine receptor. J. Am. Chem. Soc. *127*, 350–356.
24. Xiu, X., Puskar, N.L., Shanata, J.A.P., Lester, H.A., and Dougherty, D.A. (2009). Nicotine binding to brain receptors requires a strong cation-pi interaction. Nature *458*, 534–537.
25. Blum, A.P., Lester, H.A., and Dougherty, D.A. (2010). Nicotinic pharmacophore: the pyridine N of nicotine and carbonyl of acetylcholine hydrogen bond across a subunit interface to a backbone NH. Proc. Natl. Acad. Sci. USA *107*, 13206–13211.
26. Tavares, X.D.S., Blum, A.P., Nakamura, D.T., Puskar, N.L., Shanata, J.A.P., Lester, H.A., and Dougherty, D.A. (2012). Variations in binding among several agonists at two stoichiometries of the neuronal, alpha4-beta2 nicotinic receptor. J. Am. Chem. Soc. *134*, 11474–11480.
27. Hays, J.T., and Ebbert, J.O. (2008). Varenicline for tobacco dependence. N. Engl. J. Med. *359*, 2018–2024.
28. Coe, J.W., Brooks, P.R., Wirtz, M.C., Bashore, C.G., Bianco, K.E., Vetelino, M.G., Arnold, E.P., Lebel, L.A., Fox, C.B., Tingley, F.D., III., et al. (2005). 3,5-bicyclic aryl piperidines: A novel class of alpha 4 beta 2 neuronal nicotinic receptor partial agonists for smoking cessation. Bioorg. Med. Chem. Lett. *15*, 4889–4897.
29. Mihalak, K.B., Carroll, F.I., and Luetje, C.W. (2006). Varenicline is a partial agonist at alpha 4 beta 2 and a full agonist at alpha 7 neuronal nicotinic receptors. Mol. Pharmacol. *70*, 801–805.
30. Tapper, A.R., McKinney, S.L., Nashmi, R., Schwarz, J., Deshpande, P., Labarca, C., Whiteaker, P., Marks, M.J., Collins, A.C., and Lester, H.A. (2004). Nicotine activation of alpha4* receptors: sufficient for reward, tolerance, and sensitization. Science *306*, 1029–1032.
31. Maskos, U., Molles, B.E., Pons, S., Besson, M., Guiard, B.P., Guilloux, J.P., Evrard, A., Cazala, P., Cormier, A., Mameli-Engvall, M., et al. (2005). Nicotine reinforcement and cognition restored by targeted expression of nicotinic receptors. Nature *436*, 103–107.
32. Campello, H.R., Del Villar, S.G., Honraedt, A., Minguez, T., Oliveira, A.S.F., Ranaghan, K.E., Shoemark, D.K., Bermudez, I., Gotti, C., Sessions, R.B., and Mulholland, A.J. (2018). Unlocking nicotinic selectivity via direct C−H functionalisation of (−)-cytisine. Chem *4*, 1710–1725.

33. Peng, C., Stokes, C., Mineur, Y.S., Picciotto, M.R., Tian, C., Eibl, C., Tomassoli, I., Guendisch, D., and Papke, R.L. (2013). Differential modulation of brain nicotinic acetylcholine receptor function by cytisine, varenicline, and two novel bispidine compounds: emergent properties of a hybrid molecule. J. Pharmacol. Exp. Ther. *347*, 424–437.
34. Lummis, S.C.R., Thompson, A.J., Bencherif, M., and Lester, H. (2011). Varenicline Is a Potent Agonist of the Human 5-Hydroxytryptamine(3) Receptor. J. Pharmacol. Exp. Ther. *339*, 125–131.
35. Nys, M., Kesters, D., and Ulens, C. (2013). Structural insights into Cys-loop receptor function and ligand recognition. Biochem. Pharmacol. *86*, 1042–1053.
36. Rouden, J., Lasne, M.C., Blanchet, J., and Baudoux, J. (2014). (-)-Cytisine and derivatives: synthesis, reactivity, and applications. Chem. Rev. *114*, 712–778.
37. Coe, J.W., Vetelino, M.G., Bashore, C.G., Wirtz, M.C., Brooks, P.R., Arnold, E.P., Lebel, L.A., Fox, C.B., Sands, S.B., Davis, T.I., et al. (2005). In pursuit of alpha 4 beta 2 nicotinic receptor partial agonists for smoking cessation: Carbon analogs of (-)-cytisine. Bioorg. Med. Chem. Lett. *15*, 2974–2979.
38. Dale, H., and Laidlaw, P. (1912). The physiological action of cytisine, the active alkaloid of laburnum (Cytisus laburum). J. Pharmacol. Exp. Ther. *3*, 205–221.
39. Etter, J.F., Lukas, R.J., Benowitz, N.L., West, R., and Dresler, C.M. (2008). Cytisine for smoking cessation: a research agenda. Drug Alcohol Depend. *92*, 3–8.
40. Etter, J.F. (2006). Cytisine for smoking cessation: a literature review and a meta-analysis. Arch. Intern. Med. *166*, 1553–1559.
41. Zatonski, W., Cedzynska, M., Tutka, P., and West, R. (2006). An uncontrolled trial of cytisine (Tabex) for smoking cessation. Tob. Control *15*, 481–484.
42. Walker, N., Howe, C., Glover, M., McRobbie, H., Barnes, J., Nosa, V., Parag, V., Bassett, B., and Bullen, C. (2014). Cytisine versus nicotine for smoking cessation. N. Engl. J. Med. *371*, 2353–2362.
43. Ofori, S., Lu, C., Olasupo, O.O., Dennis, B.B., Fairbairn, N., Devereaux, P., and Mbuagbaw, L. (2023). Cytisine for smoking cessation: A systematic review and meta-analysis. Drug Alcohol Depend. *251*, 110936.
44. Papke, R.L., and Heinemann, S.F. (1994). Partial Agonist Properties of Cytisine on Neuronal Nicotinic Receptors Containing the b2 Subunit. Mol. Pharmacol. *45*, 142–149.
45. Gotti, C., and Clementi, F. (2021). Cytisine and cytisine derivatives. More than smoking cessation aids. Pharmacol. Res. *170*, 105700.
46. Price, K.L., Lillestol, R.K., Ulens, C., and Lummis, S.C. (2015). Varenicline Interactions at the 5-HT3 Receptor Ligand Binding Site are Revealed by 5-HTBP. ACS Chem. Neurosci. *6*, 1151–1157.
47. Clarke, A; Price, KL; Lummis, S. Cytisine lower potency at 5-HT3 receptors may explain its lower incidence of nausea and vomiting than varenicline. In European 20th Annual Conference for Research on Nicotine and Tobacco, Virtual Meeting; 2020.
48. Van Arnam, E.B., and Dougherty, D.A. (2014). Functional probes of drug-receptor interactions implicated by structural studies: Cys-loop receptors provide a fertile testing ground. J. Med. Chem. *57*, 6289–6300.
49. Marotta, C.B., Rreza, I., Lester, H.A., and Dougherty, D.A. (2014). Selective ligand behaviors provide new insights into agonist activation of nicotinic acetylcholine receptors. ACS Chem. Biol. *9*, 1153–1159.
50. Rucktooa, P., Haseler, C.A., van Elk, R., Smit, A.B., Gallagher, T., and Sixma, T.K. (2012). Structural characterization of binding mode of smoking cessation drugs to nicotinic acetylcholine receptors through study of ligand complexes with acetylcholine-binding protein. J. Biol. Chem. *287*, 23283–23293.
51. Billen, B., Spurny, R., Brams, M., van Elk, R., Valera-Kummer, S., Yakel, J.L., Voets, T., Bertrand, D., Smit, A.B., and Ulens, C. (2012). Molecular actions of smoking cessation drugs at alpha4beta2 nicotinic receptors defined in crystal structures of a homologous binding protein. Proc. Natl. Acad. Sci. USA *109*, 9173–9178.
52. Olsen, J.A., Balle, T., Gajhede, M., Ahring, P.K., and Kastrup, J.S. (2014). Molecular recognition of the neurotransmitter acetylcholine by an acetylcholine binding protein reveals determinants of binding to nicotinic acetylcholine receptors. PLoS One *9*, e91232.
53. Zhang, H.K., Eaton, J.B., Yu, L.F., Nys, M., Mazzolari, A., van Elk, R., Smit, A.B., Alexandrov, V., Hanania, T., Sabath, E., et al. (2012). Insights into the structural determinants required for high-affinity binding of chiral cyclopropane-containing ligands to alpha4beta2-nicotinic acetylcholine receptors: an integrated approach to behaviorally active nicotinic ligands. J. Med. Chem. *55*, 8028–8037.
54. Noviello, C.M., Gharpure, A., Mukhtasimova, N., Cabuco, R., Baxter, L., Borek, D., Sine, S.M., and Hibbs, R.E. (2021). Structure and gating mechanism of the alpha7 nicotinic acetylcholine receptor. Cell *184*, 2121–2134.e13.
55. Zhao, Y., Liu, S., Zhou, Y., Zhang, M., Chen, H., Eric Xu, H., Sun, D., Liu, L., and Tian, C. (2021). Structural basis of human alpha7 nicotinic acetylcholine receptor activation. Cell Res. *31*, 713–716.
56. Li, H., Teng, J., and Hibbs, R.E. (2024). Structural switch in acetylcholine receptors in developing muscle. Nature *632*, 1174–1180.
57. Su, J., Yu, Z., Yin, Z., Zhang, Z., Zhao, J., Meng, Y., Li, R., Gao, Y., Zhang, H., Yu, R., and Zhao, Y. (2025). Molecular insights into the alpha6beta4 nicotinic acetylcholine receptor function and ligand recognition. Nat. Commun. *16*, 3153.
58. Li, A., Pike, A.C.W., Webster, R., Maxwell, S., Liu, W.W., Chi, G., Palace, J., Beeson, D., Sauer, D.B., and Dong, Y.Y. (2025). Structures of the human adult muscle-type nicotinic receptor in resting and desensitized states. Cell Rep. *44*, 115581.
59. Morales-Perez, C.L., Noviello, C.M., and Hibbs, R.E. (2016). X-ray structure of the human alpha 4 beta 2 nicotinic receptor. Nature *538*, 411–415.
60. Mukherjee, S., Erramilli, S.K., Ammirati, M., Alvarez, F.J.D., Fennell, K.F., Purdy, M.D., Skrobek, B.M., Radziwon, K., Coukos, J., Kang, Y., et al. (2020). Synthetic antibodies against BRIL as universal fiducial marks for single-particle cryoEM structure determination of membrane proteins. Nat. Commun. *11*, 1598.
61. Mazzaferro, S., Kang, G., Natarajan, K., Hibbs, R.E., and Sine, S.M. (2024). Structural bases for stoichiometry-selective calcium potentiation of a neuronal nicotinic receptor. Br. J. Pharmacol. *181*, 1973–1992.
62. Nemecz, Á., Prevost, M.S., Menny, A., and Corringer, P.J. (2016). Emerging molecular mechanisms of signal transduction in pentameric ligand-gated ion channels. Neuron *90*, 452–470.
63. Minguez-Viñas, T., Nielsen, B.E., Shoemark, D.K., Gotti, C., Sessions, R.B., Mulholland, A.J., Bouzat, C., Wonnacott, S., Gallagher, T., Bermudez, I., and Oliveira, A.S. (2021). A conserved arginine with non-conserved function is a key determinant of agonist selectivity in $\alpha7$ nicotinic acetylcholine receptors. Br. J. Pharmacol. *178*, 1651–1668.
64. Oliveira, A.S.F., Shoemark, D.K., Campello, H.R., Wonnacott, S., Gallagher, T., Sessions, R.B., and Mulholland, A.J. (2019). Identification of the initial steps in signal transduction in the $\alpha4\beta2$ nicotinic receptor: insights from equilibrium and nonequilibrium simulations. Structure *27*, 1171–1183.e3.
65. Oliveira, A.S.F., Edsall, C.J., Woods, C.J., Bates, P., Nunez, G.V., Wonnacott, S., Bermudez, I., Ciccotti, G., Gallagher, T., Sessions, R.B., and Mulholland, A.J. (2019). A general mechanism for signal propagation in the nicotinic acetylcholine receptor family. J. Am. Chem. Soc. *141*, 19953–19958.
66. Madeira, F., Madhusoodanan, N., Lee, J., Eusebi, A., Niewielska, A., Tivey, A.R.N., Lopez, R., and Butcher, S. (2024). The EMBL-EBI Job Dispatcher sequence analysis tools framework in 2024. Nucleic Acids Res. *52*, W521–W525.
67. Mazzaferro, S., Benallegue, N., Carbone, A., Gasparri, F., Vijayan, R., Biggin, P.C., Moroni, M., and Bermudez, I. (2011). Additional acetylcholine

(ACh) binding site at alpha4/alpha4 interface of (alpha4beta2)2alpha4 nicotinic receptor influences agonist sensitivity. J. Biol. Chem. *286*, 31043–31054.

68. Zhou, P., Tian, F., Lv, F., and Shang, Z. (2009). Geometric characteristics of hydrogen bonds involving sulfur atoms in proteins. Proteins *76*, 151–163.
69. Gregoret, L.M., Rader, S.D., Fletterick, R.J., and Cohen, F.E. (1991). Hydrogen bonds involving sulfur atoms in proteins. Proteins *9*, 99–107.
70. Harpsoe, K., Ahring, P.K., Christensen, J.K., Jensen, M.L., Peters, D., and Balle, T. (2011). Unraveling the high- and low-sensitivity agonist responses of nicotinic acetylcholine receptors. J. Neurosci. *31*, 10759–10766.
71. Eaton, J.B., Lucero, L.M., Stratton, H., Chang, Y., Cooper, J.F., Lindstrom, J.M., Lukas, R.J., and Whiteaker, P. (2014). The unique alpha4+/-alpha4 agonist binding site in (alpha4)3(beta2)2 subtype nicotinic acetylcholine receptors permits differential agonist desensitization pharmacology versus the (alpha4)2(beta2)3 subtype. J. Pharmacol. Exp. Ther. *348*, 46–58.
72. Moroni, M., Zwart, R., Sher, E., Cassels, B.K., and Bermudez, I. (2006). alpha4beta2 nicotinic receptors with high and low acetylcholine sensitivity: pharmacology, stoichiometry, and sensitivity to long-term exposure to nicotine. Mol. Pharmacol. *70*, 755–768.
73. Carbone, A.L., Moroni, M., Groot-Kormelink, P.J., and Bermudez, I. (2009). Pentameric concatenated (alpha4)(2)(beta2)(3) and (alpha4)(3)(-beta2)(2) nicotinic acetylcholine receptors: subunit arrangement determines functional expression. Br. J. Pharmacol. *156*, 970–981.
74. Olsen, J.A., Ahring, P.K., Kastrup, J.S., Gajhede, M., and Balle, T. (2014). Structural and functional studies of the modulator NS9283 reveal agonist-like mechanism of action at $\alpha 4\beta 2$ nicotinic acetylcholine receptors. J. Biol. Chem. *289*, 24911–24921.
75. Appiani, R., Viscarra, F., Biggin, P.C., Bermudez, I., Giraudo, A., Pallavicini, M., and Bolchi, C. (2024). Selective Potentiation of the (alpha4)(3)(-beta2)(2) Nicotinic Acetylcholine Receptor Response by NS9283 Analogues. ACS Chem. Neurosci. *15*, 1501–1514.
76. Wang, Z.J., Deba, F., Mohamed, T.S., Chiara, D.C., Ramos, K., and Hamouda, A.K. (2017). Unraveling amino acid residues critical for allosteric potentiation of (alpha4)3(beta2)2-type nicotinic acetylcholine receptor responses. J. Biol. Chem. *292*, 9988–10001.
77. Ji, Y., Brueckl, T., Baxter, R.D., Fujiwara, Y., Seiple, I.B., Su, S., Blackmond, D.G., and Baran, P.S. (2011). Innate C-H trifluoromethylation of heterocycles. Proc. Natl. Acad. Sci. USA *108*, 14411–14415.
78. Cabrera, P.J., Lee, M., and Sanford, M.S. (2018). Second-Generation Palladium Catalyst System for Transannular C-H Functionalization of Azabicycloalkanes. J. Am. Chem. Soc. *140*, 5599–5606.
79. Magnus, C.J., Lee, P.H., Bonaventura, J., Zemla, R., Gomez, J.L., Ramirez, M.H., Hu, X., Galvan, A., Basu, J., Michaelides, M., and Sternson, S.M. (2019). Ultrapotent chemogenetics for research and potential clinical applications. Science *364*, eaav5282.
80. Pfizer Australia (2009). Product Information, Champix (Varenicline as Tartrate). Version: Pfpchamt10709 (Pfizer), pp. 1–16.
81. Rollema, H., Shrikhande, A., Ward, K.M., Tingley III, F., Coe, J., O'Neill, B., Tseng, E., Wang, E., Mather, R., Hurst, R., et al. (2010). Pre-clinical properties of the alpha4beta2 nicotinic acetylcholine receptor partial agonists varenicline, cytisine and dianicline translate to clinical efficacy for nicotine dependence. Br. J. Pharmacol. *160*, 334–345.
82. Unal, G., Yeloglu, I., Anilanmert, B., and Narin, I. (2012). pKa Constant of Varenicline. J. Chem. Eng. Data *57*, 14–17.
83. Indurthi, D.C., and Auerbach, A. (2021). Agonist efficiency from concentration-response curves: Structural implications and applications. Biophys. J. *120*, 1800–1813.
84. Indurthi, D.C., and Auerbach, A. (2023). Agonist efficiency links binding and gating in a nicotinic receptor. eLife *12*, e86496.
85. Auerbach, A. (2024). Dynamics of receptor activation by agonists. Biophys. J. *123*, 1915–1923.
86. Picciotto, M.R., and Kenny, P.J. (2021). Mechanisms of Nicotine Addiction. Cold Spring Harb. Perspect. Med. *11*, a039610.
87. Xiao, C., Zhou, C.Y., Jiang, J.H., and Yin, C. (2020). Neural circuits and nicotinic acetylcholine receptors mediate the cholinergic regulation of midbrain dopaminergic neurons and nicotine dependence. Acta Pharmacol. Sin. *41*, 1–9.
88. Peng, A.R., Swardfager, W., Benowitz, N.L., Ahluwalia, J.S., Lerman, C., Nollen, N.L., and Tyndale, R.F. (2020). Impact of early nausea on varenicline adherence and smoking cessation. Addiction *115*, 134–144.
89. Drovandi, A.D., Chen, C.C., and Glass, B.D. (2016). Adverse Effects Cause Varenicline Discontinuation: A Meta-Analysis. Curr. Drug Saf. *11*, 78–85.
90. Bavo, F., Pallavicini, M., Gotti, C., Appiani, R., Moretti, M., Colombo, S.F., Pucci, S., Viani, P., Budriesi, R., Renzi, M., et al. (2020). Modifications at C(5) of 2-(2-Pyrrolidinyl)-Substituted 1,4-Benzodioxane Elicit Potent alpha4beta2 Nicotinic Acetylcholine Receptor Partial Agonism with High Selectivity over the alpha3beta4 Subtype. J. Med. Chem. *63*, 15668–15692.
91. Fan, C. (2020). Structure, Function, and Application of Bacterial ABC Transporters. PhD Thesis (California Institute of Technology).
92. Armarego, W.L.F. (1963). Covalent hydration in 1,4,5,8-tetra-azanaphthalenes. J. Chem. Soc., 4304–4312.
93. Perrin, D.D. (1965). Dissociation Constants of Organic Bases in Aqueous Solution (Butterworths).
94. Barlow, R.B., and McLeod, L.J. (1969). Some studies on cytisine and its methylated derivatives. Br. J. Pharmacol. *35*, 161–174.
95. Petersson, E.J., Choi, A., Dahan, D.S., Lester, H.A., and Dougherty, D.A. (2002). A perturbed pK(a) at the binding site of the nicotinic acetylcholine receptor: implications for nicotine binding. J. Am. Chem. Soc. *124*, 12662–12663.
96. Price, K.L., Rego-Campello, H., Gallagher, T., and Lummis, S.C.R. (2023). Cytisine, unlike varenicline, is a 5-HT3 receptor antagonist. Curr. Top. Pharmacol. *27*, 83–89.

Cell Reports Physical Science, Volume *6*

Supplemental information

Understanding varenicline function

via key receptor and ligand interactions

Sheenagh G. Aiken, Daniele Fiorito, Matthew Harper, Grzegorz Pikus, Juno Underhill, Jacob Murray, Joshua Rawlinson, AnnMarie C. O'Donoghue, Cecilia Gotti, Sarah C.R. Lummis, Teresa Minguez Viñas, Franco Viscarra, Isabel Bermudez, Timothy Gallagher, and A. Sofia F. Oliveira

Supplemental Methods

A. Synthetic Chemistry — **SI 2 – SI 39**

- *(i)* General information
- *(ii)* Synthetic procedures and characterization data; x-ray crystallographic details of N-Boc isovarenicline **S18**
- *(iii)* Data S1: ^{1}H and ^{13}C NMR spectra of key intermediates and final products

B. Computational Modelling — **SI 40 – SI 83**

- *(i)* Molecular dynamics (MD) simulations
- *(ii)* Analysis of MD simulations
- *(iii)* Supporting figures and tables

C. nAChR Ligand Binding Measurements — **SI 84 – SI 86**

- *(i)* Expression of human α4β2, α3β4 and α7 nAChR
- *(ii)* Radioligand binding assays
- *(iii)* Competition binding assays
- *(iv)* Statistical analysis

D. nAChR Methods and Functional Studies — **SI 87 – SI 96**

- *(i)* Animals
- *(ii)* Human α4β2 nAChR expression in *Xenopus* oocytes
- *(iii)* Single and double mutations
- *(iv)* Electrophysiological recordings
- *(v)* Statistical analysis
- *(vi)* Supporting figures and tables

E. 5-HT$_3$ Methods and Functional Studies — **SI 97 – SI 99**

- *(i)* Cell culture
- *(ii)* Radioligand binding
- *(iii)* FlexStation analysis
- *(iv)* Data analysis
- *(v)* Supporting figures

F. pK_a Determinations — **SI 100 – SI 106**

- *(i)* Experimental description of the materials and assays
- *(ii)* Theoretical background
- *(iii)* Spectrophotometric method validation: 4-dimethylaminopyridine (DMAP) and nicotine **2**
- *(iv)* Spectrophotometric titration of varenicline **1**, nicotine **2**, cytisine **3** and varenicline variants **4**-**6**

G. Supplemental Methods References — **SI 107 – SI 109**

A. Synthetic Chemistry

(i) General information

Reactions requiring inert conditions were conducted under an N_2 atmosphere using standard Schlenk-line techniques. Anhydrous solvents were obtained from an Anhydrous Engineering alumina column drying system or from distillation following standard procedures. All other reagents were purchased from commercial suppliers and used as received. Thin layer chromatography was performed using aluminum backed 60 F254 silica plates. Visualization was achieved by UV fluorescence or a basic $KMnO_4$ solution and heat.

Infrared spectra were recorded using a Perkin Elmer Spectrum Two FT-IR spectrometer.

NMR spectra were recorded on Bruker Advance III HD 500 Cryo, Varian 400-MR, Jeol ECS 400 or JEOL ECZ 400 spectrometers. Chemical shifts (δ) are quoted in parts per million (ppm) and are referenced to the residual solvent peak, coupling constants (*J*) are given in Hz. Multiplicities are abbreviated as: br (broad), s (singlet), d (doublet), t (triplet), q (quartet), m (multiplet) or combinations thereof. Assignments (when indicated) were made with the aid of COSY, HSQC, HMBC experiments.

Mass spectrometry was performed by the University of Bristol mass spectrometry service by either (EI^+) using a VG Micromass Autospec spectrometer or by electrospray ionization (ESI+) using a Bruker Daltonics MicrOTOF II spectrometer.

Numbering system (shown here for varenicline **SI0**) as used for $^1H/^{13}C$ NMR structural assignments of varenicline variants.

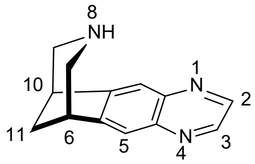

SI0 Varenicline

Structures are numbered based on the Schemes shown below except where a structure (varenicline variants) appears in the published manuscript, and the number used here corresponds to that used in the main paper.

(ii) Synthetic procedures and characterization data

1. C_2 Varenicline 4

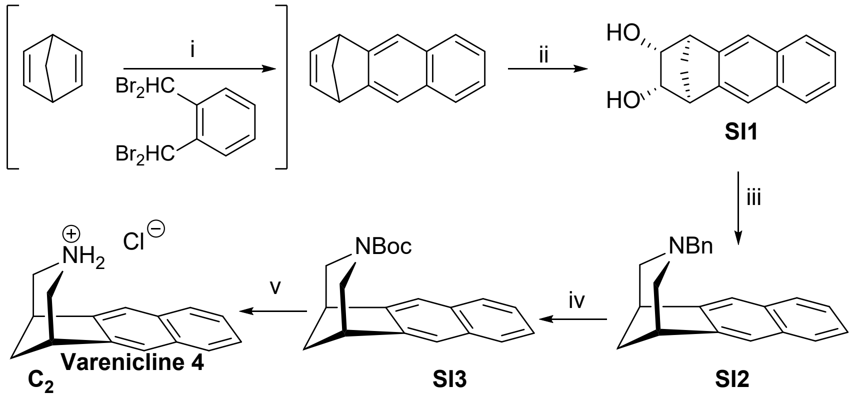

SI Scheme 1: *Reagents*: i, KI, xylene, 65 °C, 18h[1] (35%) [not included in main manuscript]; ii, NMNO, OsO_4 (cat), acetone, water (98%); iii, (a) $NaIO_4$, THF/water[2, 3] then (b) $NaBH(OAc)_3$, $BnNH_2$ (65% over 2 steps); iv, H_2, $Pd(OH)_2$ (20% wt on C), Boc_2O, MeOH/EtOAc (74%); v, HCl in MeOH (quantitative).

N-Bn C_2 Varenicline SI2

exo-1,4-Methano-1,2,3,4-tetrahydro-2,3-dihydroxyanthracenedihydroxyanthracene **SI1** (222 mg, 0.98 mmol) was dissolved in THF:H_2O (2.5:1, 20 mL) then $NaIO_4$ (230 mg, 1.08 mmol) was added in a single portion. The mixture was stirred at rt for 30 minutes then H_2O (40 mL) was added. The aqueous phase was extracted with CH_2Cl_2 (4 × 10 mL), the extracts were dried ($MgSO_4$) and concentrated to give the crude dialdehyde as a yellow oily paste. The dialdehyde was dissolved in anhydrous CH_2Cl_2 (20 mL) under an N_2 atmosphere then cooled to 0 °C. Sodium triacetoxyborohydride (830 mg, 3.92 mmol) was added followed by benzylamine (118 µL, 1.08 mmol) dropwise. The mixture was allowed to slowly warm to rt overnight, quenched with saturated aq. Na_2CO_3 (15 mL) and H_2O (20 mL). The product was extracted with CH_2Cl_2 (3 × 20 mL), the combined extracts were washed with brine (30 mL), dried ($MgSO_4$) and concentrated. Purification by silica chromatography (Biotage; 5% to 15% EtOAc in hexane) gave the ***N*-Bn C_2 varenicline SI2** (190 mg, 65%) as a colorless oil. ^1H NMR (400 MHz, $CDCl_3$) δ 7.83 – 7.76 (m, 2 H, C10/13-H), 7.55 (s, 2 H, 5/12-H), 7.44 – 7.37 (m, 2 H, 2/3-H), 7.14 – 7.06 (m, 3 H, Ph), 6.90 – 6.81 (m, 2 H, Ph), 3.48 (s, 2 H, PhCH2), 3.24 (t, *J* 4.4 Hz, 2 H, 6/10-H), 2.98 – 2.89 (m, 2 H, 7/9-H), 2.53 (d, *J* = 10.3 Hz, 2 H, 7/9-H), 2.27 (m, 1 H, 11-H), 1.79 (d, 1 H, *J* 10.5

Hz, 11-H); ^{13}C NMR (126 MHz, $CDCl_3$) δ 146.0 (C5a), 138.8 (Ph), 133.5 (C4a), 128.5 (Ph), 128.1 (Ph), 127.8 (C1), 126.6 (Ph), 124.8 (C2), 119.3 (C5), 61.8 ($PhCH_2$), 57.7 (C7), 43.6 (C11), 41.3 (C6); HRMS (ESI): calculated for $C_{22}H_{22}N$ $[M+H]^+$: 300.1747, found: 300.1733.

N-Boc C_2 Varenicline SI3

To a solution of *N*-Bn C_2 varenicline **SI2** (176 mg, 0.588 mmol) in MeOH:EtOAc (1:1, 12 mL) was added Boc_2O (0.270 mL, 1.17 mmol) and $Pd(OH)_2$ (20 wt% on carbon, 83 mg). The mixture was stirred rapidly under an atmosphere of hydrogen at room temperature for 24 h, after which the mixture was filtered through Celite. The solids were washed with CH_2Cl_2 (50 mL), the filtrate was concentrated and purification by silica chromatography (Biotage; 2% to 30% EtOAc in hexane) gave ***N*-Boc C_2 varenicline SI3** (135 mg, 74%) as a colorless solid. FTIR v_{max} / cm^{-1} (neat): 1691; ^{1}H NMR (400 MHz, $CDCl_3$; broadening and splitting of some signals due to amide resonance was observed) δ 7.80 – 7.75 (m, 2 H, 1/4-H), 7.64 (s, 1 H, 5/12-H), 7.61 (s, 1 H, 5/12-H), 7.43 – 7.37 (m, 2 H, 2/3-H), 4.07 (d, *J* 12.5 Hz, 1 H, 7/9-H), 3.94 (d, *J* 12.5 Hz, 1 H, 11-H), 3.36 – 3.25 (m, 3 H, 2 × 6/10-H, 7/9-H), 3.21 (d, *J* = 12.4 Hz, 1 H, 7/9-H), 2.35 (m, 1 H, 11-H), 1.94 (d, *J* 10.8 Hz, 1 H, C11-H), 1.16 (s, 9 H); ^{13}C NMR (126 MHz, $CDCl_3$) δ 156.1 (C=O), 143.9/143.8 (C5a, rotamers), 133.64/133.59 (C4a, rotamers), 128.1/127.7 (C1, rotamers), 125.3/125.2 (C2, rotamers), 121.2/120.6 (C5, rotamers), 79.3 ($CCMe_3$), 50.5/49.4 (C7, rotamers), 41.6 (C11), 40.1/40.0 (C6, rotamers), 28.3 ($CCMe_3$); HRMS (ESI): calculated for $C_{20}H_{24}NO_2$ $[M+H]^+$: 310.1802, found: 310.1814.

C_2 Varenicline hydrochloride salt 4

N-Boc C_2 varenicline **SI3** (24 mg, 0.078 mmol) was dissolved in HCl (4 mL, 0.5 M in MeOH) and allowed to stand for 18 h at rt. The mixture was concentrated to afford the title compound **C_2 varenicline hydrochloride 4** (19 mg, quantitative) as an off-white solid. ^{1}H NMR (400 MHz, D_2O), δ 8.02 – 7.96 (m, 2H, 1-H), 7.94 (s, 2H, 5-H), 7.63 – 7.57 (m, 2H, 2-H), 3.67 – 3.60 (m, 2H, 7-H), 3.52 (d, *J* = 12.3 Hz, 2H, 6/10-H), 3.37 (d, *J* = 12.3 Hz, 2H, 6/10-H), 2.44 (m, 1H, 11-H), 2.20 (d, *J* = 11.6 Hz, 1H, 11-H); ^{13}C NMR (126 MHz, D_2O) δ 140.5 (C4), 133.6 (C6), 128.0 (C7), 126.2 (C8), 122.5 (C5), 47.8 (C1), 40.2 (C3), 38.0 (C2); HRMS (ESI+): Calculated for $C_{15}H_{16}N$ $[M+H]^+$: 210.1283, found: 210.1268.

2. Isovarenicline 5

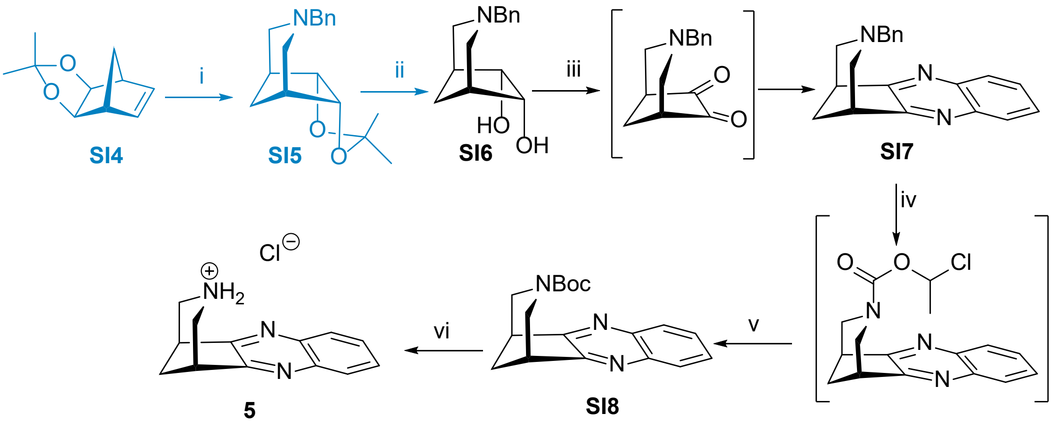

SI Scheme 2: *Reagents*: i, (a) O_3, DCM, -78 °C then Me_2S (b) $NaBH(OAc)_3$, $BnNH_2$, DCM, rt, 35% overall; ii, HCl, THF, 81%; iii, (a) DCC, DMSO, Cl_2CHCO_2H, rt, 20h; iv, (b) 1,2-phenylenediamine, rt, 18h, 70% over 2 steps; vi, chloroethyl chloroformate, $ClCH_2CHCl$ 80 °C, 18h; v, (a) MeOH, reflux 2h (b) Boc_2O, 86% over 2 steps; vi, HCl in MeOH, quantitative OR (+)-tartaric acid, EtOH/acetone 76%

Note that diol **SI6** was available commercially but synthetic details for conversion of S14 to S16 are provided here given the specialized nature of the commercial supply used.

N-Benzyl 3-azabicyclo[3.2.1]octane-6,7-diol acetonide S15

To a solution of acetonide **SI4**[4] (1.0 g, 6.0 mmol) in DCM (12 mL), ozone was bubbling at -78 °C until the blue colour appeared/persisted. The excess of ozone was removed by a stream of nitrogen at -78°C, and after that, dimethylsulfide (0.95 mL, 12.4 mmol) was added. The reaction mixture warm to rt and stirred for 16h. The solvent was removed to give a transparent oil, which was dissolved in dry DCM (80 mL) and sodium triacetoxyborohydride (5.10 g, 24.18 mmol) was added. The mixture was cooled to 0 °C and a solution of benzylamine (0.71 g, 0.73 mL) in dry DCM (20 mL) was added dropwise over 30 minutes. The mixture was warmed to rt, stirred for 18h, then washed with water (30 mL) and brine (30 mL) and dried ($MgSO_4$). The solvent was removed and the crude product was purified by silica gel chromatography (ethyl acetate:hexane) to give **acetonide SI5** (575 mg, 35%) as a colorless oil, which was used without additional purification. ^1H NMR (400 MHz, $CDCl_3$): δ 7.25-7.13 (m, 5H), 4.44 (d, *J*=1.6 Hz, 2H), 3.33 (s, 2H), 2.67-2.63 (m, 2H), 2.08 (t, *J* 4 Hz, 2H), 1.99 (d, *J* 12 Hz, 2H), 1.87 (m, 1H), 1.36 (s,

3H), 1.28 (s, 3H), 1.09 (d, J = 12 Hz, 1H); ^{13}C NMR (100 MHz, $CDCl_3$): δ 138.6, 128.6, 128.2, 126.9, 108.5, 83.4, 62.5, 56.1, 40.3, 31.6, 25.9, 23.8; R_f: 0.70 (30% ethyl acetate in hexane).

N-Benzyl 3-azabicyclo[3.2.1]octane-6,7-diol SI6

Acetonide **SI5** (663 mg, 2.43 mmol) was dissolved in THF (12 mL) and 4M HCl (12 mL) was added. The mixture was stirred at 80°C for 72h, after which time 10% aq. $NaHCO_3$(30 mL) was added and pH was adjusted to pH 10 by addition of aqueous Na_2CO_3 dropwise. The aqueous solution was extracted with ethyl acetate (3 x 30 mL), and the extracts were dried (Na_2SO_4) and concentrated. Purification of the residue by silica gel chromatography (hexane:ethyl acetate) gave **diol SI6** (459 mg, 81%) as a colorless oil that solidified, and could be further purified by recrystallization from ethyl acetate. ^{1}H NMR (400 MHz, $CDCl_3$): δ 7.23-7.12 (m, 5H), 4.11 (s, 2H), 3.32 (s, 2H), 3.19 (bs, 2H), 2.69-2.65 (m, 2H), 2.02-2.00 (m, 2H). 1.94 (d, J = 8Hz, 2H), 1.90-1.52 (m, 1H), 1.07 (d, J = 8 Hz, 1H); ^{13}C NMR (100 MHz, $CDCl_3$): δ 138.8, 128.6, 128.1, 126.9, 75.2, 62.4, 57.1, 43.6, 32.0; R_f: 0.41 (50% ethyl acetate in hexane); MS (ESI): calculated for $[C_{14}H_{20}NO_2]^+$: 234.1494, found $[M+H]^+$: 234.1490.

***N*-Benzyl isovarenicline SI7** (via Pfitzner–Moffatt oxidation).

A solution of N-benzyl 3-azabicyclo[3.2.1]octane-6,7-diol **SI6**[4] (250 mg, 1.07 mmol) in DMSO (2 mL) was added to a mixture of DCC (1.77 g, 8.57 mmol) in DMSO (10 mL) followed by dichloroacetic acid (221 mg, 140 μL, 1.72 mmol). The reaction mixture was stirred at rt for 20h, filtered through celite and the solids were washed with EtOAc (50 mL). EtOAc was removed under reduced pressure and to the resulting orange solution was added 1,2-phenylenediamine (116 mg, 1.07 mmol). The mixture was stirred at rt for 18h, water (150 mL) was then added and the product was extracted with EtOAc (3 x 50mL). The extracts were washed with water (150 mL) and brine (150 mL), dried (Na_2SO_4) and after removal of solvents, the residue was purified by chromatography (hexane: EtOAc 9:1 → 7:3) to give **N-benzyl isovarenicline SI7** (224 mg, 70%) as a pale orange solid. ^{1}H NMR (400 MHz, $CDCl_3$): δ 7.99-7.95 (m, 2 H), 7.63-7.59 (m, 2 H), 7.04-6.89 (m, 3 H). 6.72-6.68 (m, 2 H), 3.38 (s, 2 H), 3.26 (t, 2 H, = 8 Hz), 3.15- 3.11 (m, 2 H), 2.61 (d, 2 H, = 8 Hz), 2.34 (m, 1 H), 1.92 (d, 1 H, = 12 Hz); ^{13}C NMR

(100 MHz, $CDCl_3$): δ 163.3, 141.7, 137.4, 128.8, 128.4, 128.3, 128.0, 126.8, 61.5, 57.0, 41.1, 39.6; MS (ESI): calculated for $[C_{20}H_{20}N_3]^+$: 302.1652, found $[M+H]^+$: 302.1650.

[Using **SI6** and analogous Swern oxidation conditions (DMSO/TFAA/DCM at -78 °C), we isolated **SI7** in 44% yield.]

***N*-Boc isovarenicline SI8**

Chloroethyl chloroformate (251 μL, 2.32 mmol) was added to a solution of N-benzyl isovarenicline **SI7** (100 mg, 0.332 mmol) in 1,2-dichloroethane (7 mL) and the mixture was stirred at 80°C for 20 h. After cooling to rt and removal of solvent, the residue was dissolved in MeOH (7 mL) and heated under reflux for 2h.** After cooling, di-*tert*-butyl dicarbonate (87 mg, 0.4 mmol) was added and the mixture was stirred at rt for 18h. After concentration, purification by chromatography (hexane:EtOAc 7:3 → 5:5) gave **N-Boc isovarenicline SI8** (89 mg, 86%) as a colorless solid. ^{1}H NMR (400 MHz, $CDCl_3$; broadening due to amide resonance was observed): δ 7.99-7.96 (m, 2 H), 7.65-7.60 (m, 2 H), 4.23-4.10 (m 2 H), 3.40-3.25 (m, 4 H), 2.49-2.43 (m, 1 H), 2.05 (d, = 12 Hz, 1 H), 1.14 (s, 9 H); ^{13}C NMR (100 MHz, $CDCl_3$): δ 161.4, 155.6, 142.2, 129.1, 80.1, 49.3, 48.3, 40.0, 38.1, 28.2; MS (ESI): calculated for $[C_{18}H_{21}N_3O_2Na]^+$: 334.1527, found $[M+Na]^+$: 334.1526.

** At this point, the solvent can be evaporated, and the residue was filtered through a plug of silica (EtOAc as eluent) to provide isovarenicline **5** (as the free base) that was judged (by TLC and ^{1}H NMR) to be sufficiently pure to use directly to prepare the tartrate salt (see below). The advantage of the N-Boc intermediate **SI8** is its ease of purification.

The structure of N-Boc isovarenicline **SI8** was confirmed by X-ray crystallographic analysis (Figure S1). X-ray diffraction experiments of **S18** were carried out at 100(2) K on a Bruker APEX II diffractometer using Mo-Kα radiation (λ = 0.71073 A) and a CCD area detector. Intensities were integrated in SAINT[a] and absorption corrections based on equivalent reflections were applied using SADABS.[b] The structure was solved using ShelXT[c] and refined by full matrix least squares against FS2[d] in ShelXL[d,e] using Olex2.[f] All of the non-hydrogen atoms were refined anisotropically while all of the hydrogen atoms were located geometrically and refined using a riding model. The crystal structure, refinement data and references relating to this structure

determination are given below. Crystallographic data has been deposited with the Cambridge Crystallographic Data Centre as supplementary publication **CCDC 2464749**.

a. Bruker, SAINT+ v8.39.0 Integration Engine, Data Reduction Software, Bruker Analytical X-ray Instruments Inc., Madison, WI, USA, **2018**.
b. Bruker, SADABS 2018, Bruker AXS area detector scaling and absorption correction, Bruker Analytical X-ray Instruments Inc., Madison, Wisconsin, USA, **2018**.
c. Sheldrick, G. M. Acta Crystallographica a-Foundation and Advances **2015**, 71, 3–8.
d. Sheldrick, G. M. *Acta Crystallogr., Sect. A: Found. Crystallogr.* **2008**, *64*, 112–122.
e. Sheldrick, G. M. *Acta Crystallogr. C* **2015**, *71*, 3–8.
f. Dolomanov, O. V.; Bourhis, L. J.; Gildea, R. J.; Howard, J. A. K.; Puschmann, H. *J. Appl. Crystallogr.* **2009**, *42*, 339–341.

Figure S1. Crystal structure of *N*-Boc isovarenicline **S18**, with the anisotropic displacement parameters depicted at the 50% probability level and hydrogens omitted for clarity.

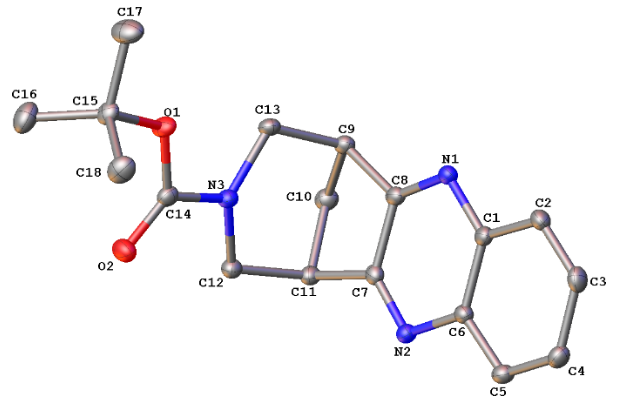

**CCDC number**	**2464749**	μ/mm^{-1}	0.086
Empirical formula	$C_{18}H_{21}N_3O_2$	F(000)	664.0
Formula weight	311.38	Crystal size/mm^3	0.502 × 0.363 × 0.126
Temperature/K	100(2)	Radiation	MoKα (λ = 0.71073)
Crystal system	Monoclinic	2Θ range for data collection/°	2.872 to 56.068
Space group	$P2_1/n$	Index ranges	-8 ≤ h ≤ 8, -36 ≤ k ≤ 37, -11 ≤ l ≤ 8
a/Å	6.7087(3)	Reflections collected	14534
b/Å	28.3664(11)	Independent reflections	3865 [R_{int} = 0.0505, R_{sigma} = 0.0504]
c/Å	8.4356(4)	Data/restraints/parameters	3865/0/211
α/°	90	Goodness-of-fit on F^2	1.027
β/°	93.782(3)	Final R indexes [I>=2σ (I)]	R_1 = 0.0481, wR_2 = 0.1058
γ/°	90	Final R indexes [all data]	R_1 = 0.0744, wR_2 = 0.1174
Volume/Å^3	1601.81(12)	Largest diff. peak/hole / e Å^{-3}	0.28/-0.23
Z	4	μ/mm^{-1}	0.086
ρ_{calc}g/cm^3	1.291		

Isovarenicline HCl salt 5.

To a solution of *N*-Boc isovarenicline **SI8** (180 mg, 0.58 mmol) in MeOH (5 mL) was added HCl (4M in dioxane, 1 mL). After 8 h, the solvents were removed and the solid was triturated with cold EtOAc to give **isovarenicline HCl 5** (140 mg, quantitative) as a light tan solid^{1}H NMR (600 MHz,D_2O): δ 7.98 (m, 2 H, H1), 7.80 (m, 2 H, H2), 3.62 (br d, 2 H, *J* = 12 Hz, H7a), 3.58 (m, 2 H, H6), 3.44 (d, 2 H, *J* = 12 Hz, H7b), 2.60 (m, 1 H, H9a), 2.33 (d, 1 H, *J* = 12 Hz, H9b); ^{13}C NMR (100 MHz, D_2O): δ 158.7, 141.5, 130.8, 128.1, 46.3, 38.2, 36.8. MS (ESI): calculated for $[C_{13}H_{14}N_3]^+$: 212.1188, found $[M]^+$: 212.1190.

Alternative salt preparation

Isovarenicline <u>tartrate</u> salt

Isovarenicline (from N-Bn isovarenicline **SI7** - see** above; 134 mg, 0.64 mmol) in EtOH:acetone (10:3, 1.5 mL) was added to a solution of (*L*)-(+)-tartaric acid (102 mg, 0.68 mmol) in EtOH:acetone (10:3, 1 mL). After 15 min, the mixture was cooled to 0°C and the solid was isolated by filtration, washed with a small quantity of cold 10:3 EtOH:acetone and air-dried to give **isovarenicline tartrate** (174 mg, 76%) as an off-white powder. Spectroscopic properties were identical to the HCl salt with the exception of the signals associated with tartrate, but full details are provided below. ^{1}H NMR (600 MHz, D_2O): δ 7.96 (m, 2 H, H1), 7.80 (m, 2 H, H2), 4.39 (s, 2 H, tartrate), 3.62 (br d, 2 H, *J* = 12 Hz, H7), 3.58 (m, 2 H, H6), 3.44 (d, 2 H, *J* = 12 Hz, H7), 2.60 (m, 1 H, H9), 2.33 (d, 1 H, *J* = 12 Hz, H9); ^{13}C NMR (100 MHz, D_2O): δ 176.3 (C=O, tartrate), 158.7, 141.5, 130.8, 128.1 , 72.8, 46.3, 38.2, 36.8.

3. N_2 Varenicline 6

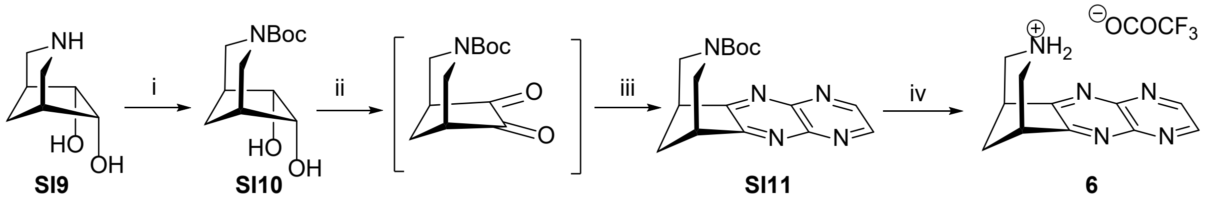

SI9 SI10 SI11 6

SI Scheme 3. *Reagents*: i, Boc_2O, Na_2CO_3, THF/water, rt, 16h (90%); ii, TFAA, DMSO, CH_2Cl_2, then Et_3N; iii, 2,3-diaminopyrazine, MeOH, 65 °C, 16h (38% over 2 steps); iv, 5% TFA in MeOH (69%).

***tert*-Butyl-6,7-dihydroxy-3-azabicyclo[3.2.1]octane-3-carboxylate SI10**

To a solution of commercially available diol **SI9**·(as HCl salt; also available by hydrogenolysis of **SI6**) (0.89 g, 5.00 mmol) in THF (16 mL) was added di-tert-butyl dicarbonate (1.31 g, 6.00 mmol), followed by a solution of Na_2CO_3 (1.11 g, 10.5 mmol) in water (8 mL). The mixture was stirred 16 h at rt, then diluted with EtOAc (50 mL) and brine (20 mL). The phases were separated, and the aqueous phase was extracted with EtOAc (2 x 50 mL). The combined EtOAc extracts were washed with brine (50 mL), dried (Na_2SO_4), concentrated and the residue was purified by silica gel chromatography (pentane/EtOAc 20:80 to 100% EtOAc) to give **SI10** (1.10 g, 90%) as a colorless solid. ^1H NMR (500 MHz, $CDCl_3$) δ 4.18 – 4.05 (m, 4H), 2.83 (d, *J* = 13.0 Hz, 2H), 2.26 (m, 2H), 1.58 – 1.49 (m, 2H), 1.45 (s, 9H). ^{13}C NMR (126 MHz, $CDCl_3$) δ 156.2, 80.1, 70.3, 37.7, 29.4, 29.4, 28.5. HRMS (ESI) calculated for $C_{12}H_{21}NO_4$, $[M+H]^+$: 244.1543, found: 244.1546.

***N*-Boc N_2 Varenicline SI11**

To a solution of DMSO (0.15 mL) in CH_2Cl_2 (1.2 mL) at -78 °C, was slowly added trifluoroacetic anhydride (0.21 mL, 1.5 mmol), followed by dropwise addition of a solution of diol **SI10** (0.12 g, 0.5 mmol) in CH_2Cl_2 (0.4 mL). The reaction mixture was stirred for 1 h at -78 °C, then triethylamine (0.35 mL, 2.5 mmol) was added. The resulting mixture was stirred 1 h at rt, then water (5 mL) was added and the mixture was extracted with CH_2Cl_2 (2 x 10 mL). The combined extracts were dried (Na_2SO_4) and the crude 1,2-diketone was used immediately in the next step. To a solution of crude diketone (as above) in MeOH (1.0 mL) was added 2,3-diaminopyrazine[5] (55 mg, 0.5 mmol) The resulting heterogeneous mixture was then stirred at 65 °C in a closed Schenck tube for 16 h affording a tan-colored homogeneous solution. The mixture was concentrated and purification by chromatography (BIOTAGE, Sfar Silica HC D 10 g column, CH_2Cl_2/MeOH gradient 2 to 20%) and recrystallization from diethyl ether, afforded ***N*-Boc N_2 varenicline SI11** (60 mg, 38% over 2 steps) as a pale-yellow solid. ^1H NMR (400 MHz, $CDCl_3$) δ 9.04 (s, 2H), 4.36 – 4.19 (m, 2H), 3.66 – 3.48 (m, 2H), 3.49 – 3.35 (m, 2H), 2.61 (m, 1H), 2.20 (d, *J* = 11.7 Hz, 1H), 1.23 (s, 9H). ^{13}C NMR (101 MHz, $CDCl_3$) δ 167.3, 155.5, 147.0,

146.5, 80.5, 49.5, 48.5, 40.2, 38.0, 28.2. HRMS (ESI) calculated for $C_{16}H_{19}N_5O_2$, $[M+Na]^+$: 336.1431, found: 336.1428.

N_2 Varenicline trifluoroacetate 6

N-Boc N_2 varenicline **SI11** (40 mg, 0.13 mmol) was dissolved in a solution of trifluoroacetic acid (5% v/v in dichloromethane, 4 mL) and allowed to stand for 16 h at rt. The solvent was removed and toluene was added to facilitate removal of residual TFA under vacuum. The resulting brown oil was triturated with acetone affording a crystalline yellow solid, from which the solvent was decanted, and the solids were washed again with acetone, acetone decanted and the solid was dried under vacuum to give **N_2 varenicline trifluoroacetate 6** (30 mg, 69%) as a yellow crystalline solid. . ^{1}H NMR (500 MHz, DMSO) δ 9.22 (s, 1H), 9.19 (s, 2H), 8.61 (s, 1H), 3.65 (dd, *J* = 4.9, 2.3 Hz, 2H), 3.62 (d, *J* = 11.0 Hz, 2H), 3.42 (d, *J* = 11.0 Hz, 2H), 2.63 (m, 1H), 2.42 (d, *J* = 11.7 Hz, 1H). ^{13}C NMR (126 MHz, DMSO) δ 164.4, 158.3 (q, *J* = 35.8 Hz), 147.7, 146.4, 115.8 (q, *J* = 292.4 Hz), 45.9, 38.3, 36.5. ^{19}F NMR (376 MHz, DMSO-*D*$_6$) δ -74.57. HRMS (ESI) calculated for $C_{11}H_{12}N_5$, $[M+H]^+$: 214.1087, found: 214.1089.

We also evaluated an alternative approach to N_2 varenicline **6** based on use of the N-benzyl intermediate by analogy to the chemistry used for **4** and **5**.

N-Benzyl N_2 varenicline SI12

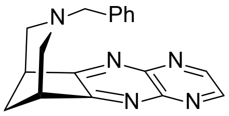

SI12

To mixture of DCC (1.77 g, 8.57 mmol) in DMSO (10 mL) a solution of benzyl-3-azabicyclo[3.2.1]octane-6,7-diol **SI6** (250 mg, 1.07 mmol) in DMSO (2 mL) was added followed by dichloroacetic acid (221 mg, 140 μL, 1.72 mmol). The reaction mixture was stirred at rt for 20h, then filtered through celite which was washed with ethyl acetate (50 mL). EtOAc was removed and to the orange residue was added 2,3-diaminopyrazine (118 mg, 1.072 mmol) and the mixture was stirred at 65 °C for 3h. Water (150 mL) was added, and aqueous phase was extracted with ethyl acetate (3 x 50mL). The combined extracts were washed with water (150 mL) and brine (150 mL), concentrated, and the orange residue was purified by silica

chromatography (eluent 1% MeOH → 2.5% MeOH in DCM) to give **N-benzyl N_2 Varenicline SI12** (130 mg, 40%) as a pale-yellow solid. ^{1}H NMR (400 MHz, $CDCl_3$): δ 8.94 (s, 2H), 7.05 – 7.00 (m, 3H), 6.71 – 6.69 (m, 2H), 3.42 (bt, J = 4 Hz, 2H), 3.38 (s, 2H), 3.23 – 3.19 (m, 2H), 2.68 (d, J = 8 Hz, 2H), 2.44 (m, 1H), 2.02 (d, J = 12 Hz, 1H); ^{13}C NMR (100 MHz, $CDCl_3$): δ 169.3, 146.4, 146.1., 137.1, 128.4, 128.3, 127.1, 61.8, 57.4, 41.3, 39.5; Calculated for $[C_{18}H_{18}N_5]^+$: 304.1562, found $[M+H]^+$: 304.1560.

N-Benzyl N_2 varenicline **SI12** was of very limited synthetic utility because under a variety of conditions (essentially those approaches that had worked for debenzylation of **SI2** and **SI7**), we were unable to deprotect successfully **SI12**. We only observed substrate decomposition but ^{1}H NMR analysis of the crude product indicated heteroarene reduction/fragmentation had probably occurred.

(iii) Data S1: ^{1}H and ^{13}C NMR spectra of key intermediates and final products.

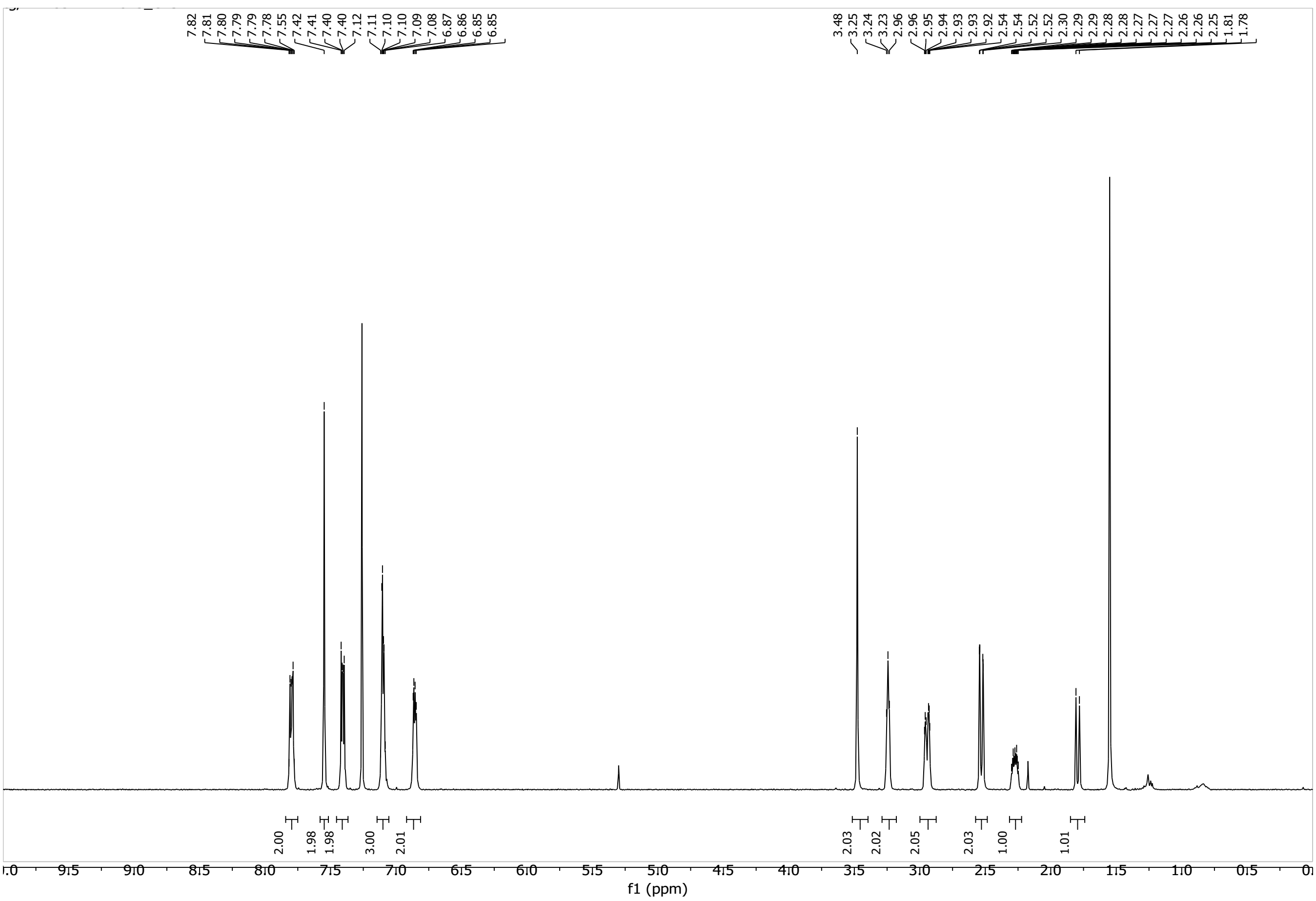

^{1}H NMR of *N*-Bn C_2 varenicline **SI2**

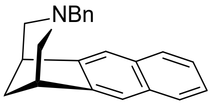

SI2

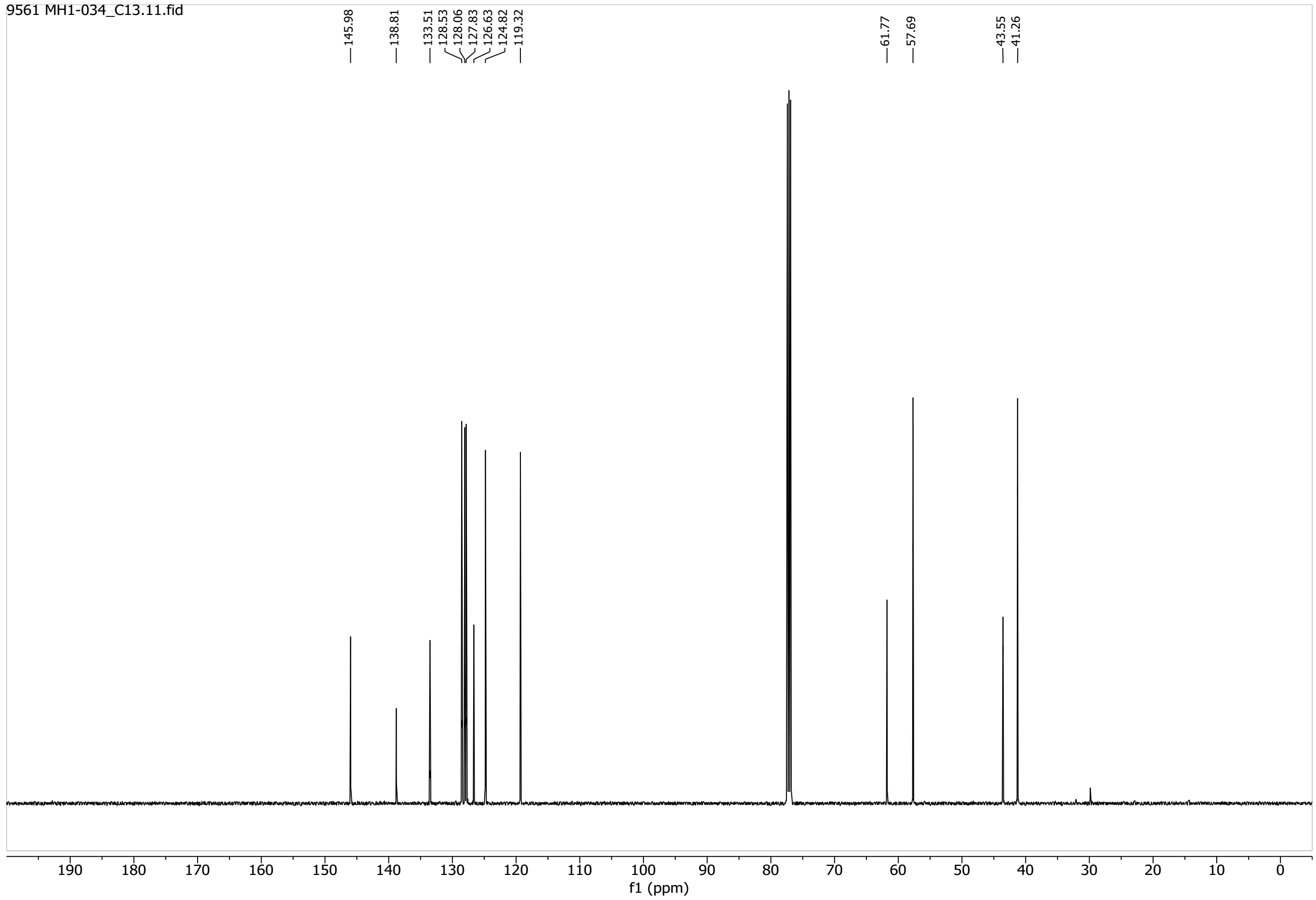

^{13}C NMR of *N*-Bn C_2 varenicline **SI2**

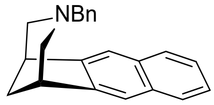

SI2

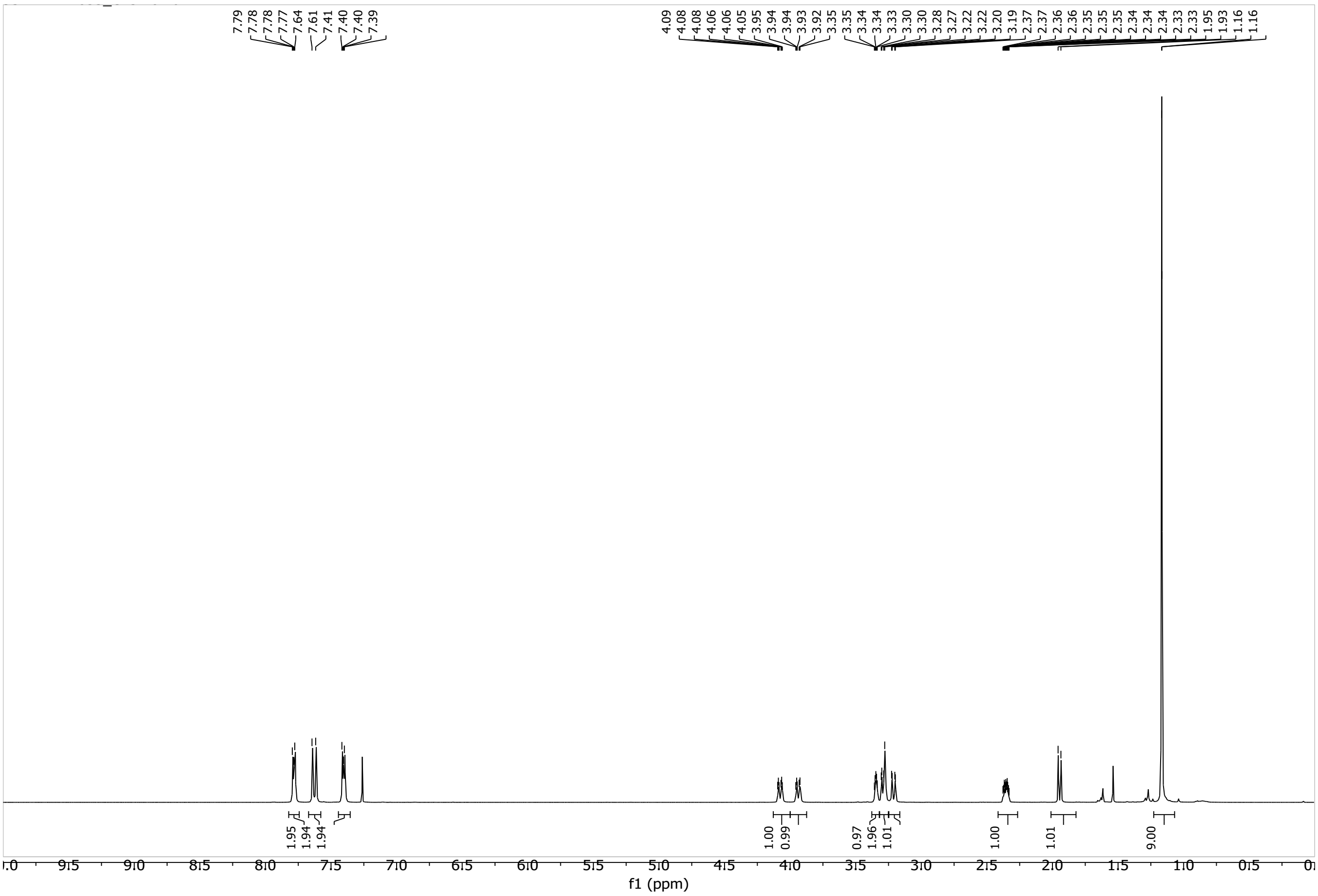

^{1}H NMR of *N*-Boc C_2 varenicline **SI3**

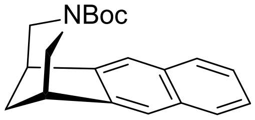

SI3

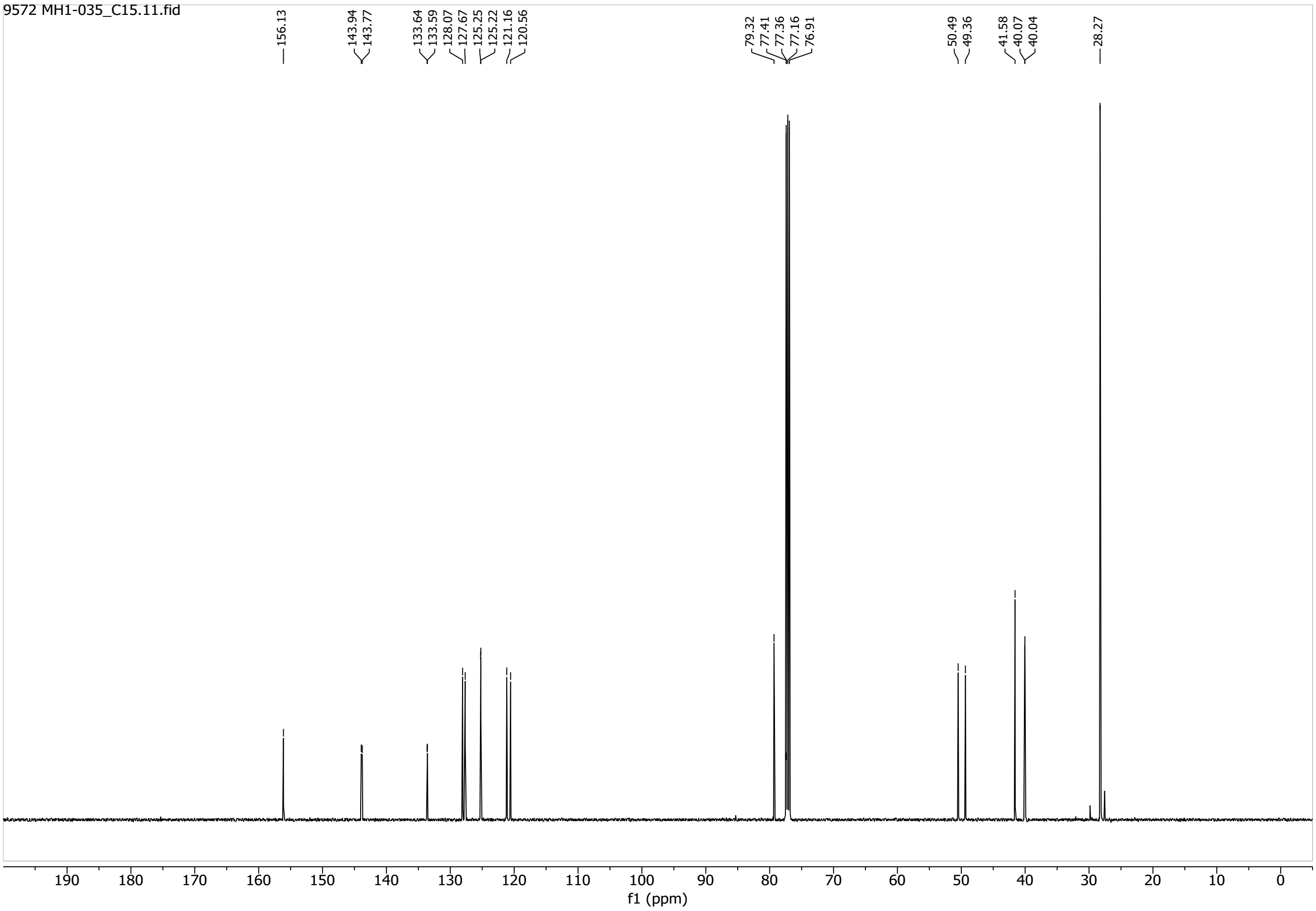

^{13}C NMR of *N*-Boc C_2 varenicline **SI3**

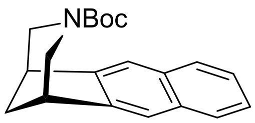

SI3

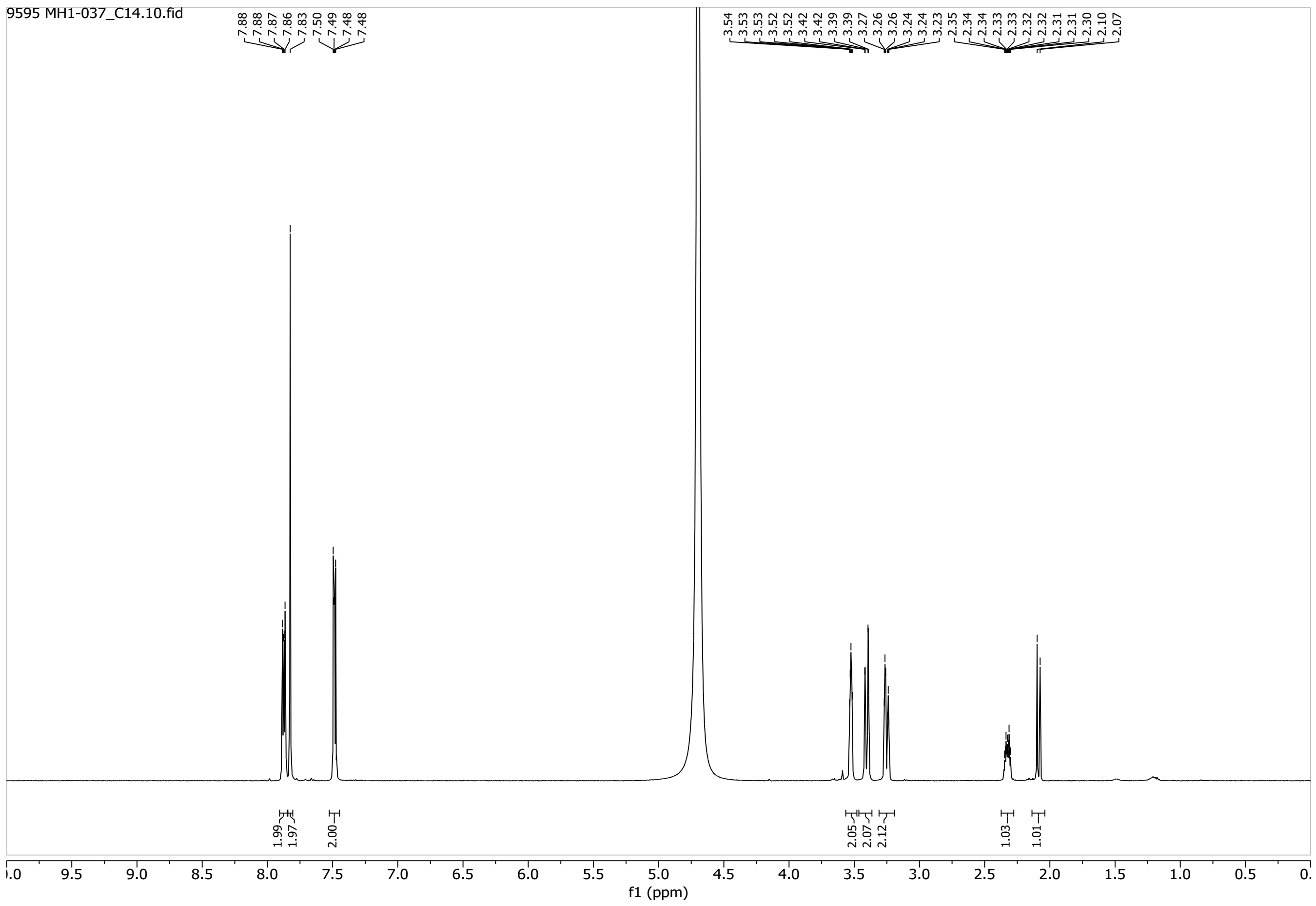

^{1}H NMR C_2 varenicline.HCl **4**

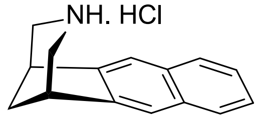

4

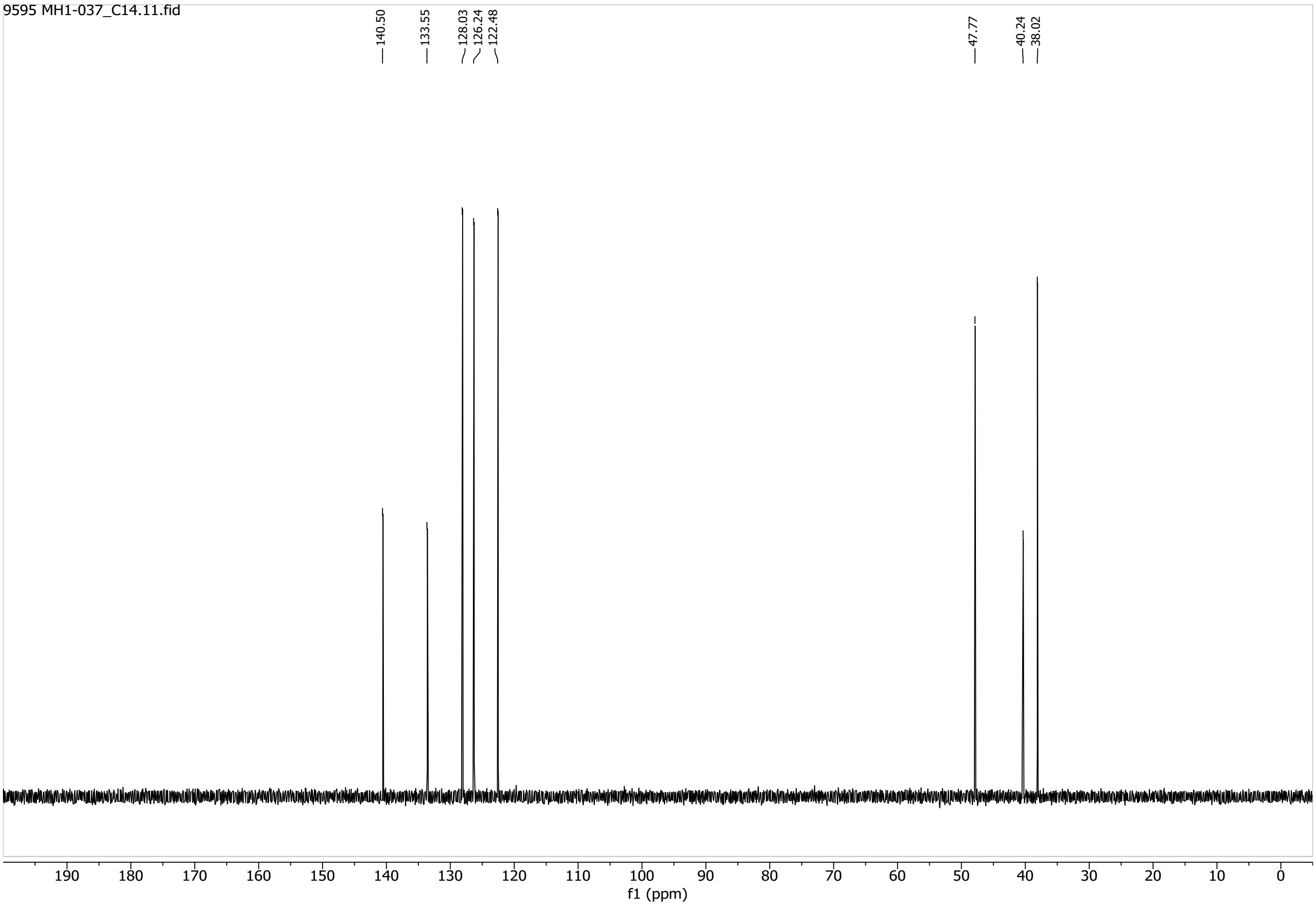

^{13}C NMR C_2 varenicline.HCl **4**

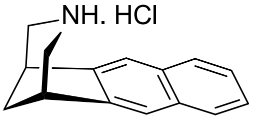

4

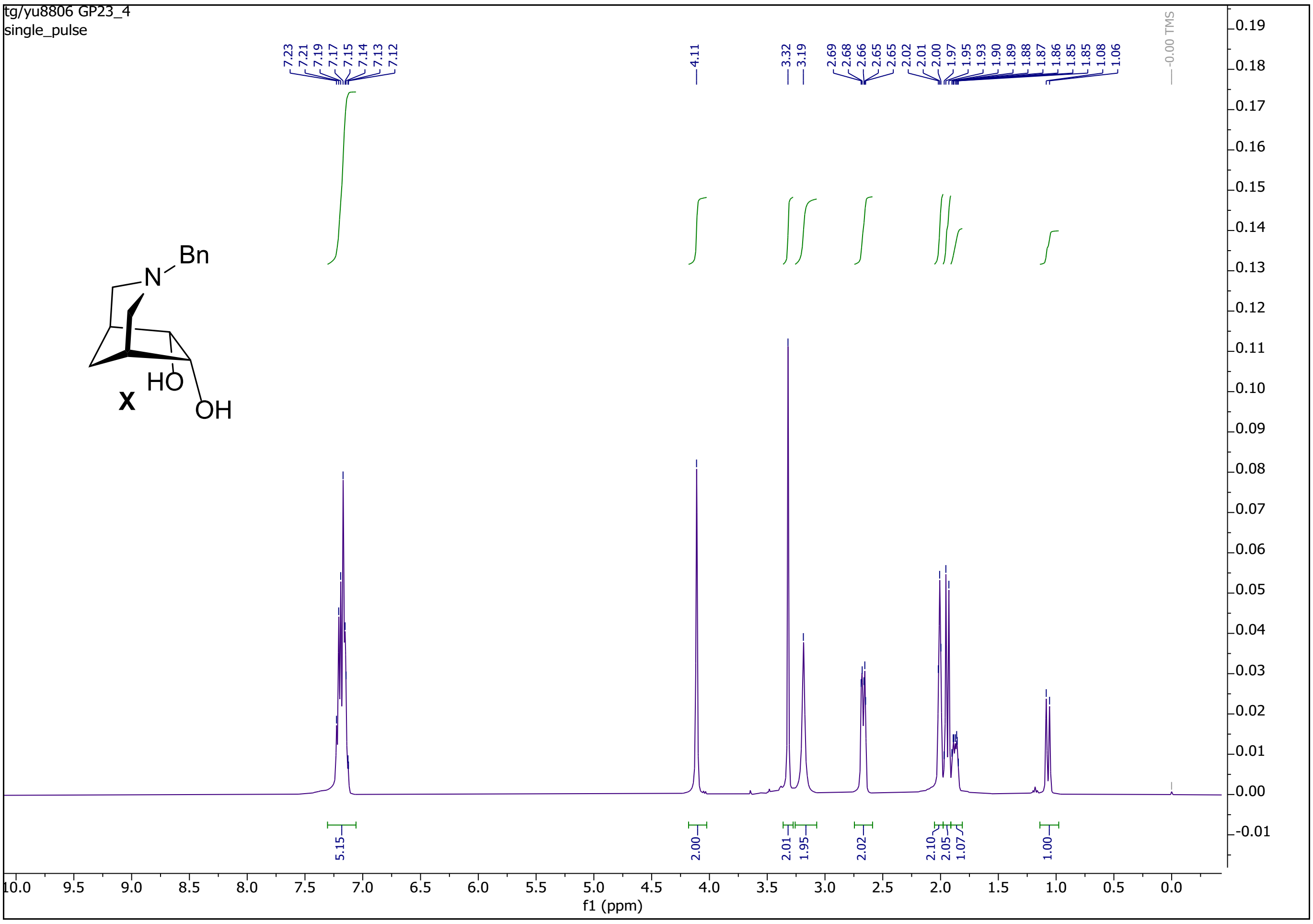

^{1}H NMR of *N*-Bn diol **SI6**

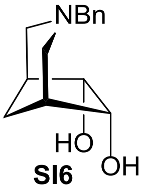

SI6

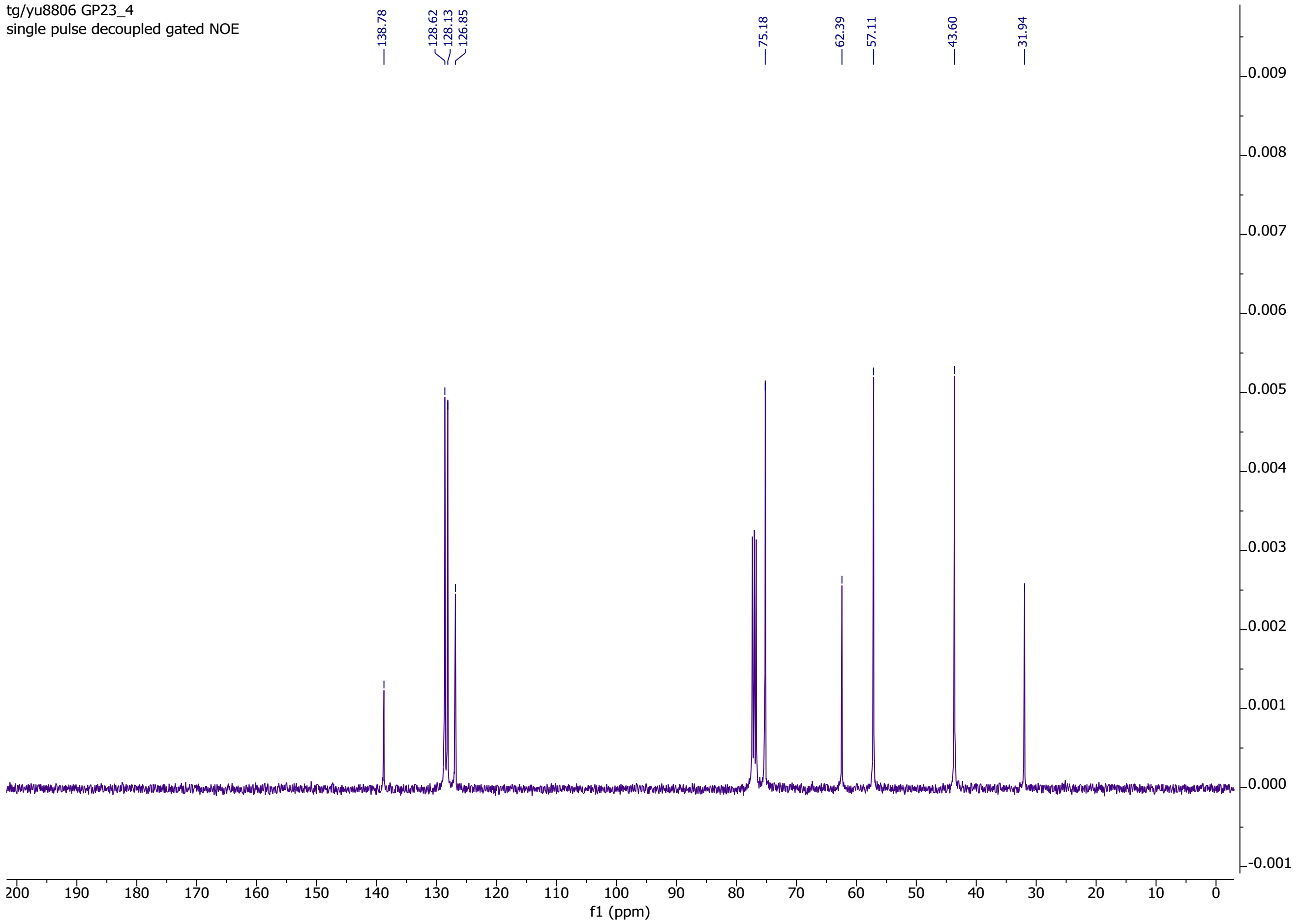

^{13}C NMR of *N*-Bn diol **SI6**

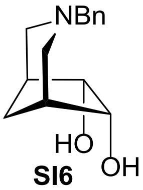

SI6

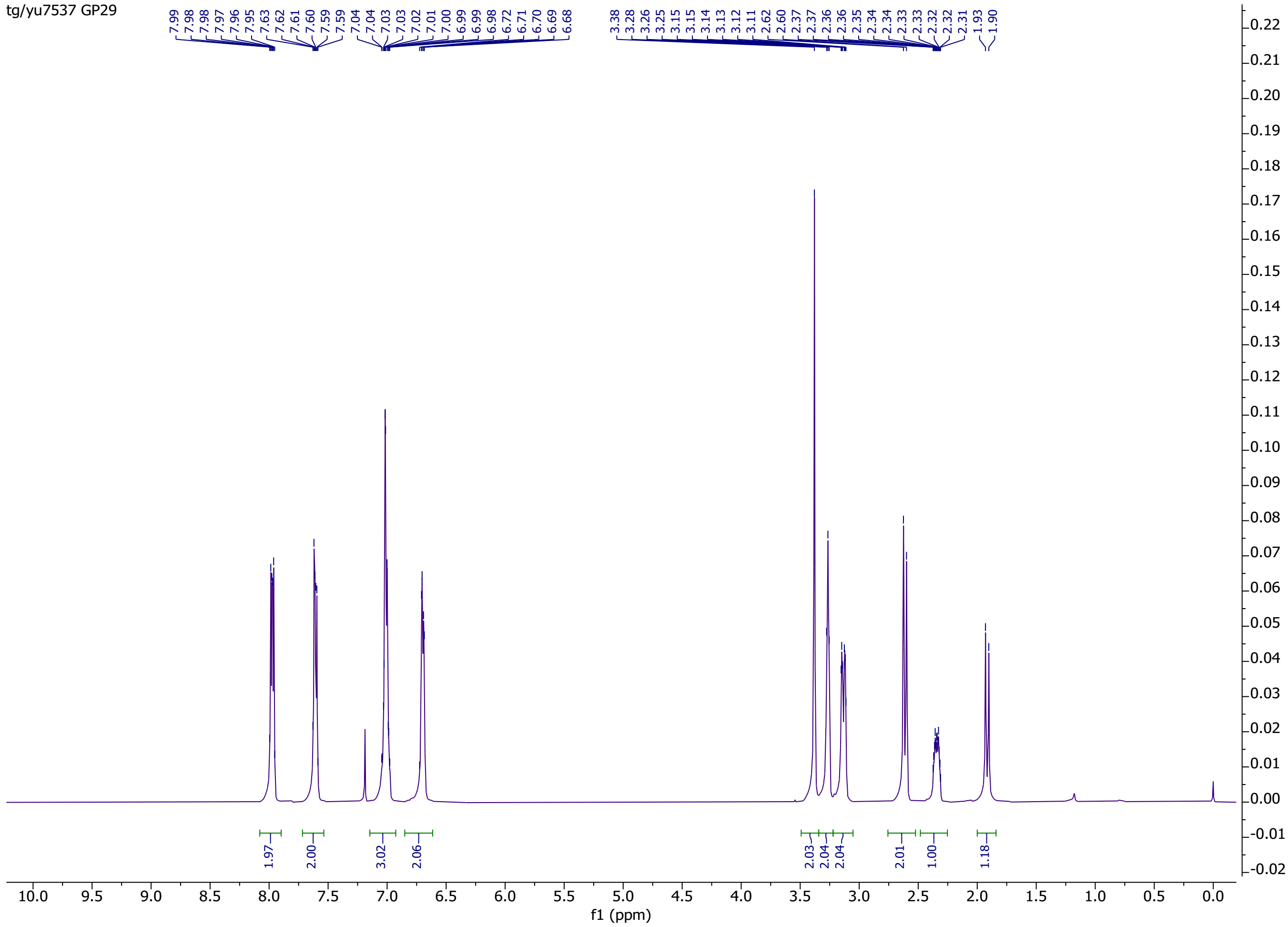

^{1}H NMR of *N*-Bn isovarenicline **SI7**

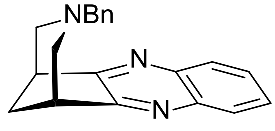

SI7

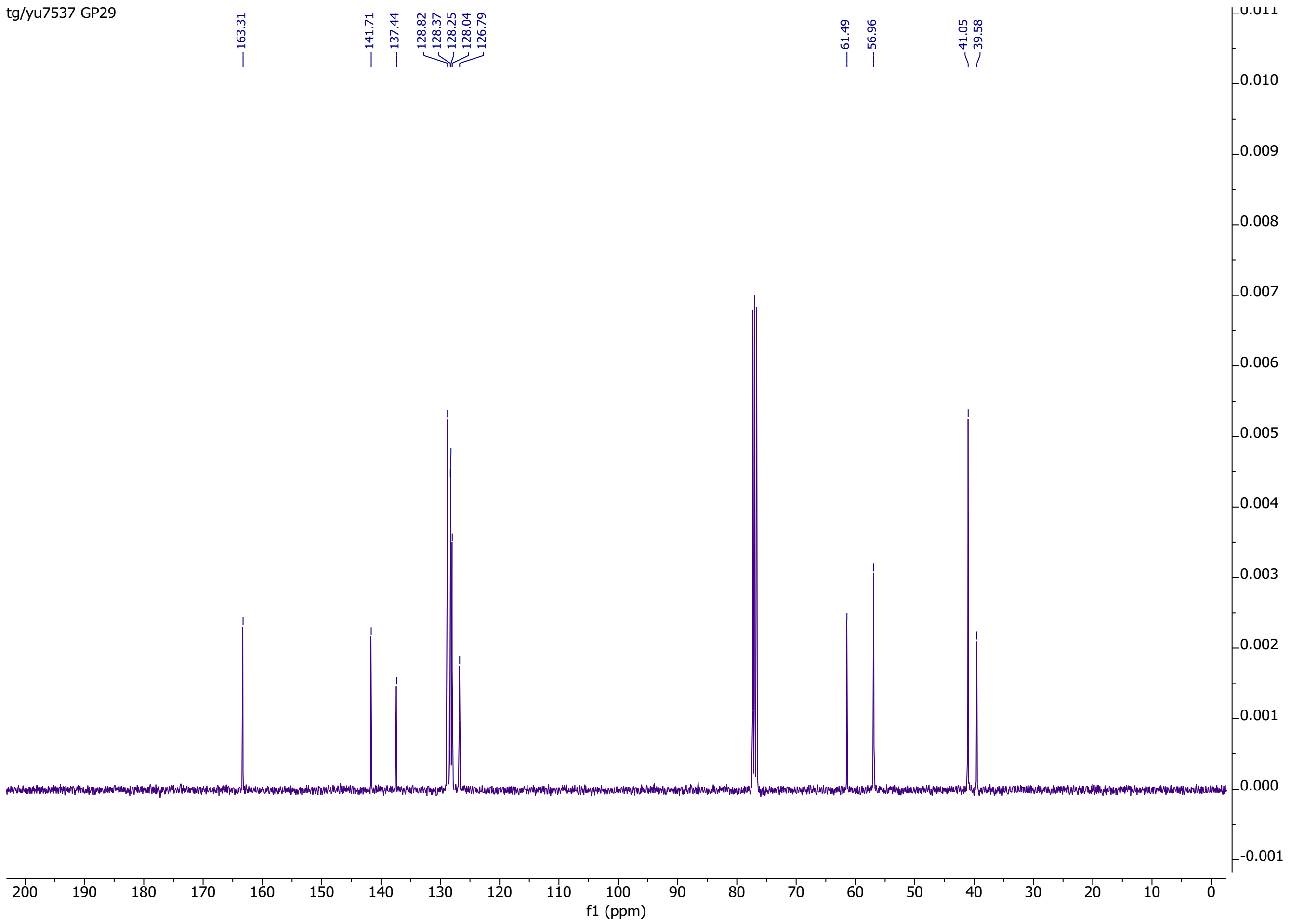

^{13}C NMR of *N*-Bn isovarenicline **SI7**

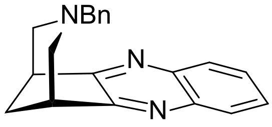

SI7

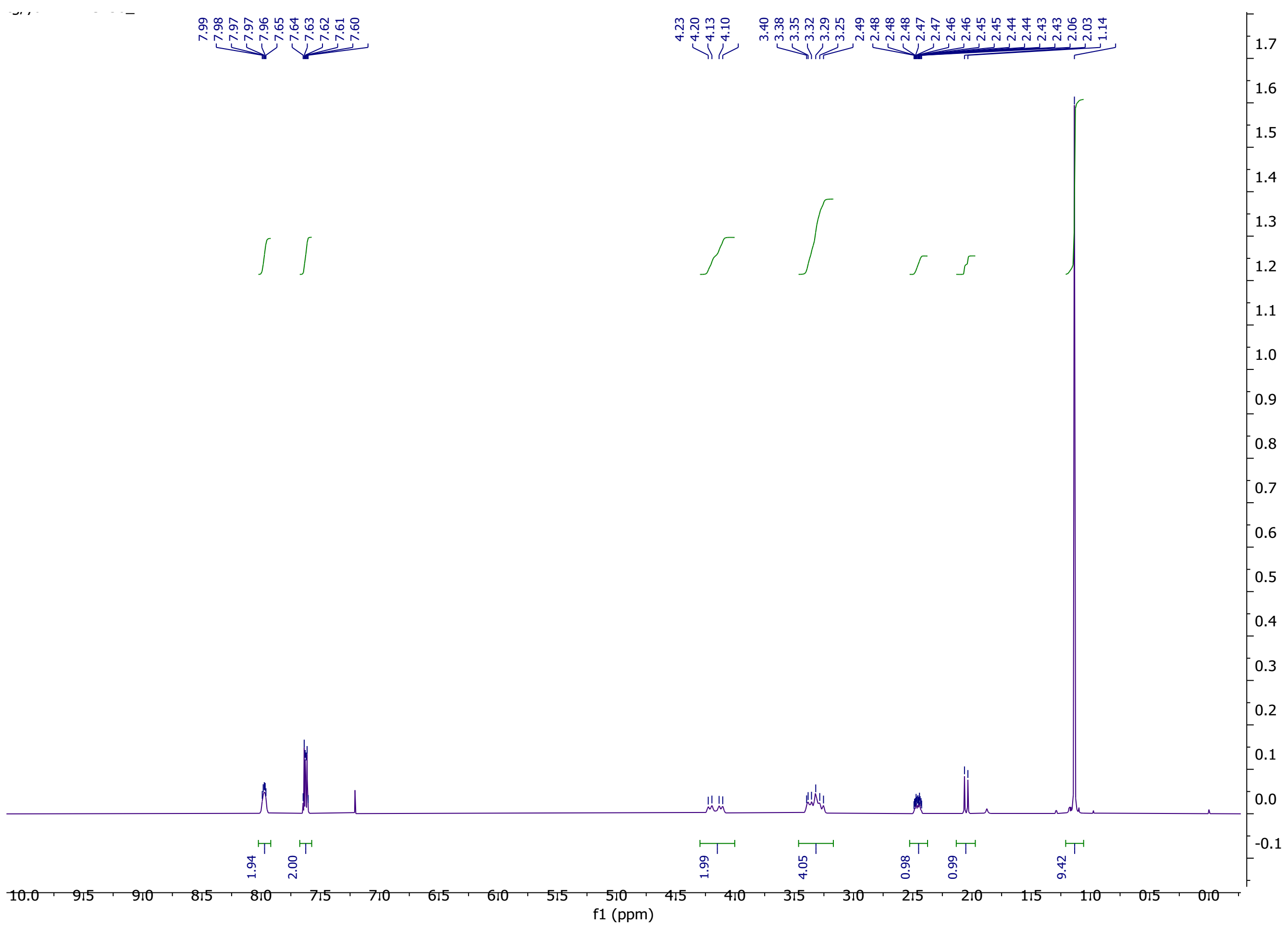

^{1}H NMR of *N*-Boc isovarenicline **SI8**

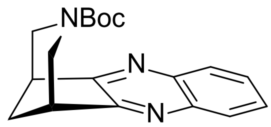

SI8

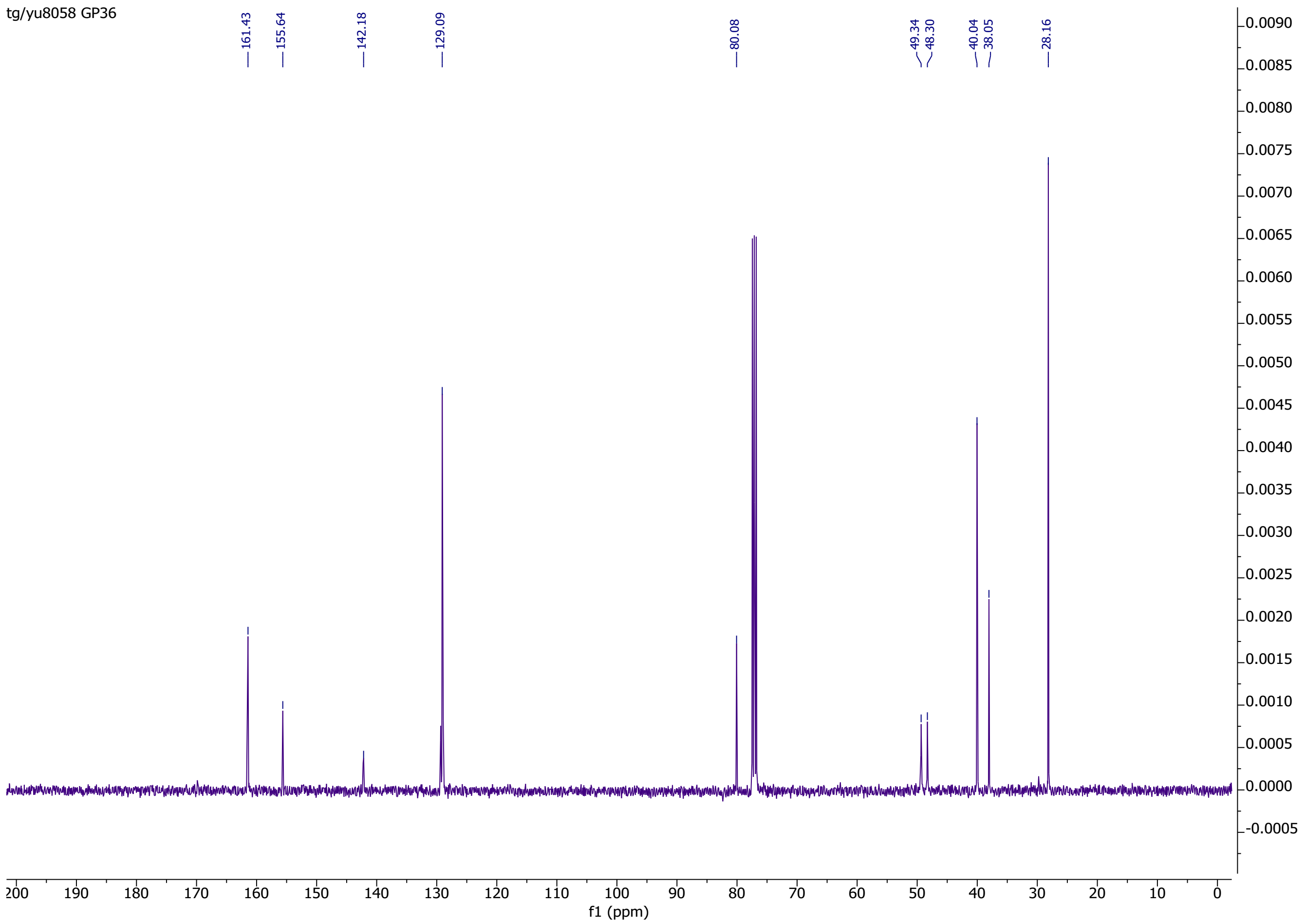

^{13}C NMR of *N*-Boc isovarenicline **SI8**

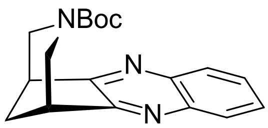

SI8

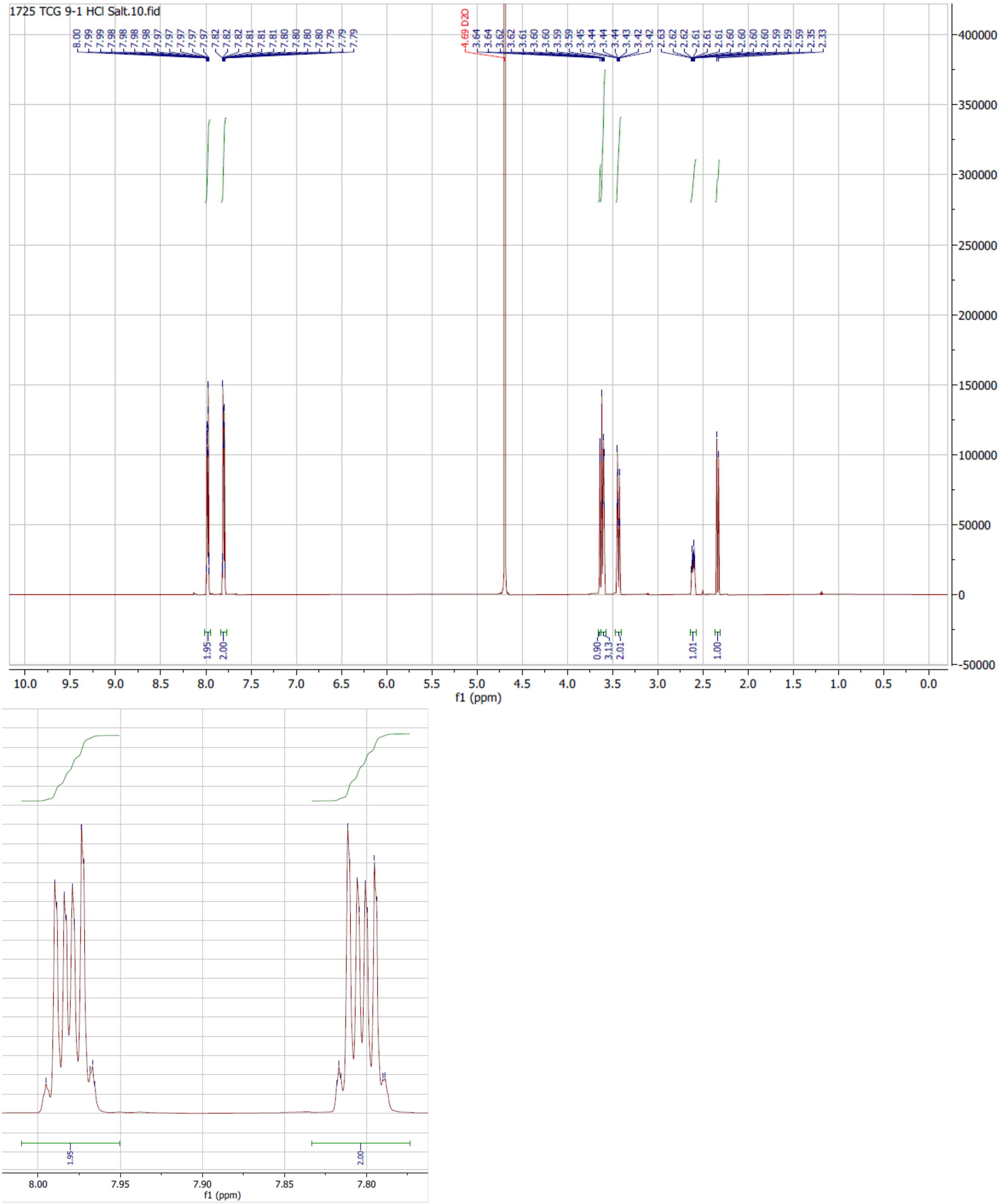

1725 TCG 9-1 HCl Salt.10.fid
4.69 D2O
f1 (ppm)

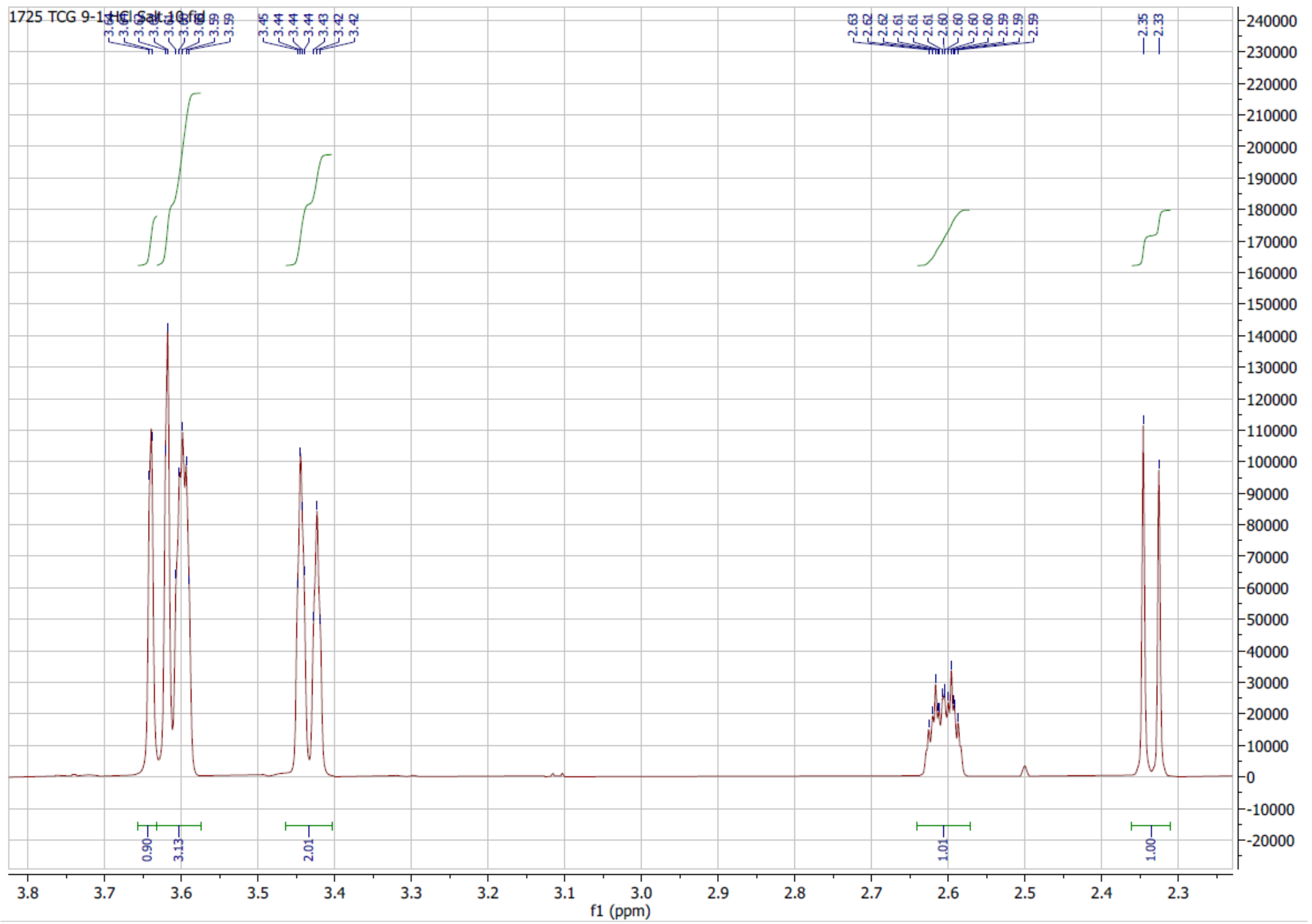

^{1}H NMR of isovarenicline HCl **5**

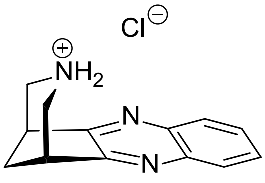

5

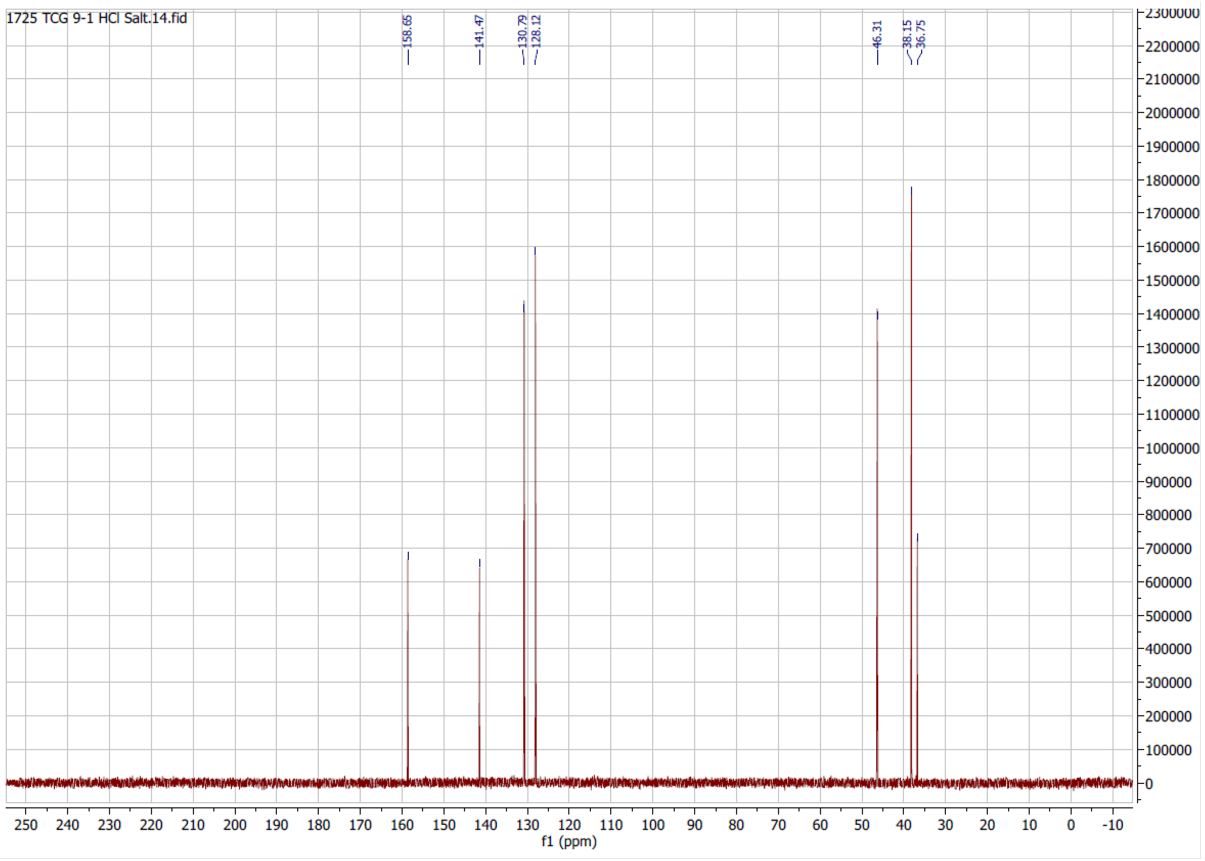

^{13}C NMR of isovarenicline HCl **5**

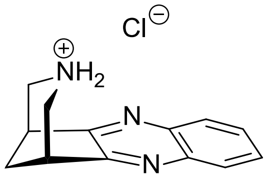

5

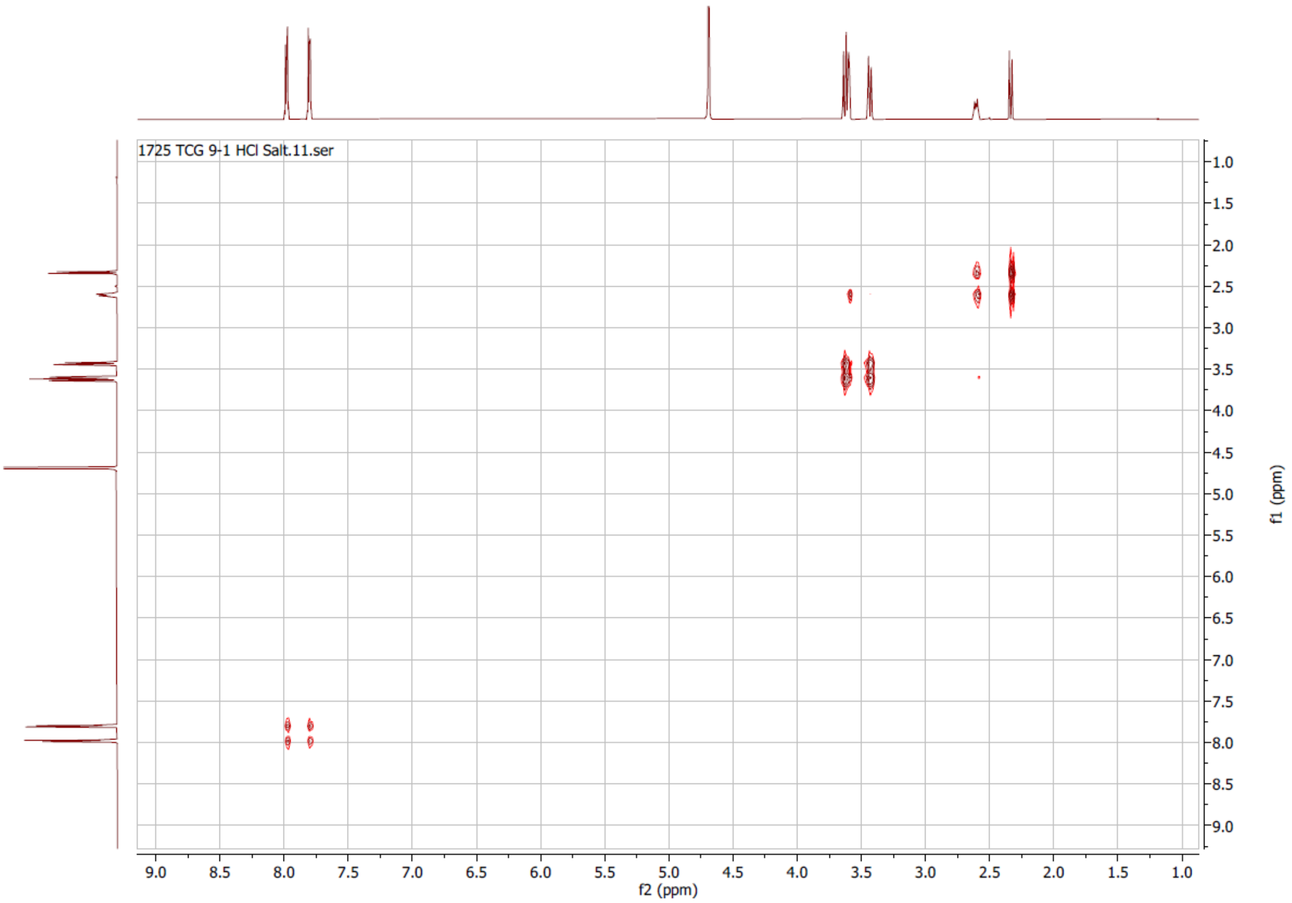
1725 TCG 9-1 HCl Salt.11.ser
f2 (ppm)
f1 (ppm)

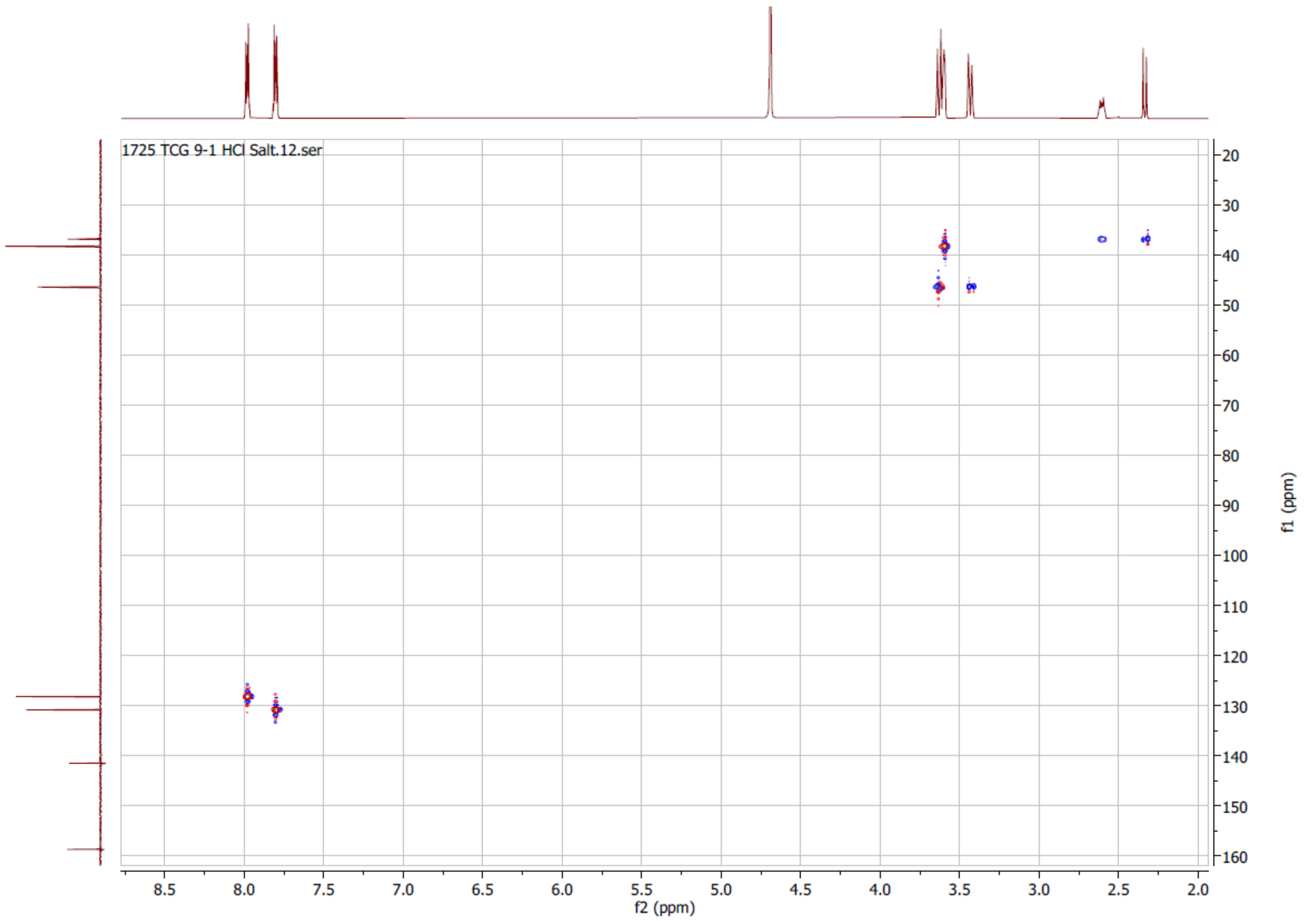
1725 TCG 9-1 HCl Salt.12.ser
8.5 8.0 7.5 7.0 6.5 6.0 5.5 5.0 4.5 4.0 3.5 3.0 2.5 2.0
f2 (ppm)
20 30 40 50 60 70 80 90 100 110 120 130 140 150 160
f1 (ppm)

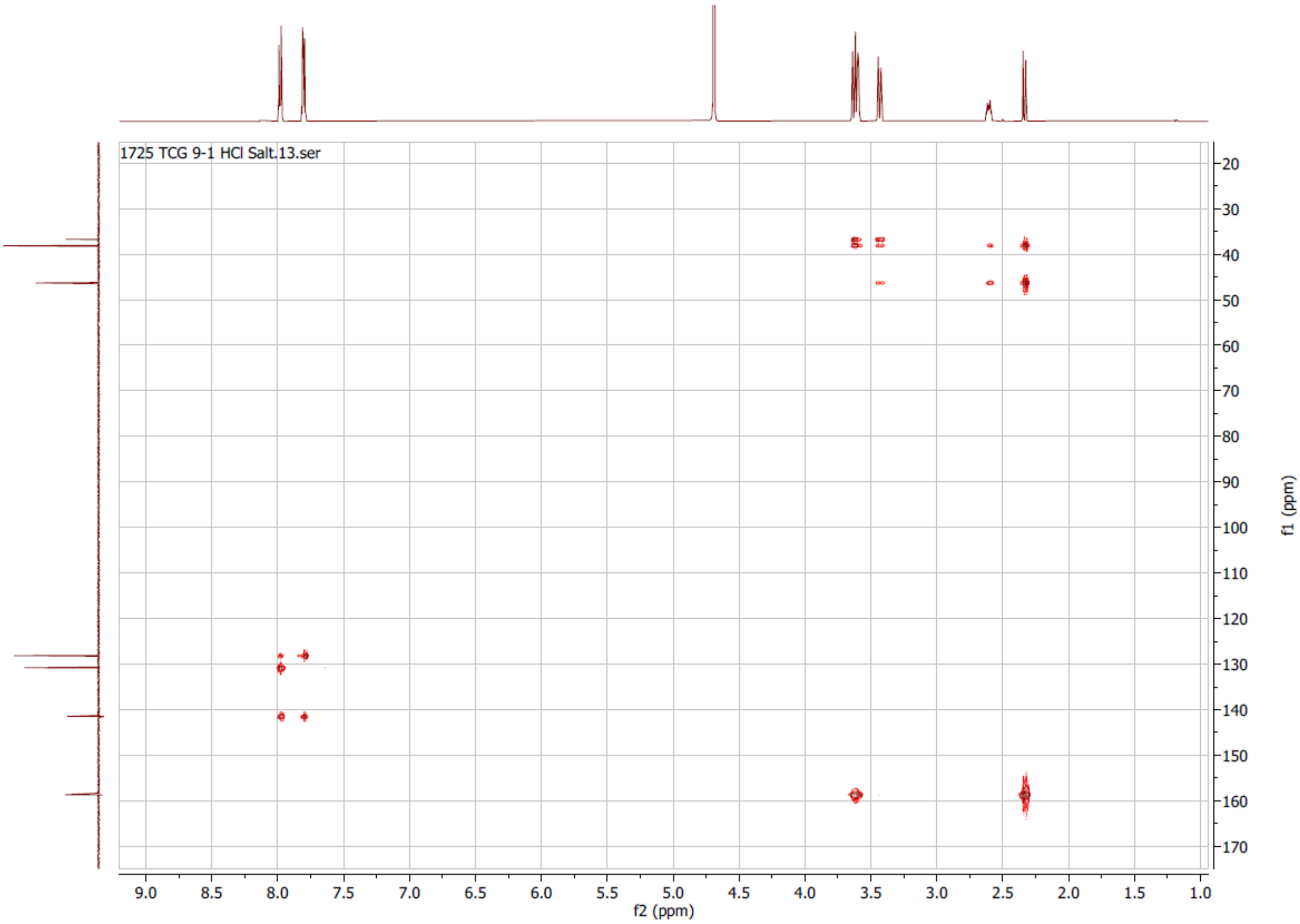

2D COSY. HSQC and HMBC spectra of isovarenicline HCl **5**

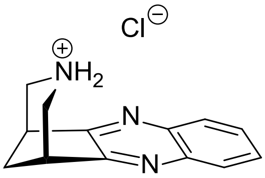

5

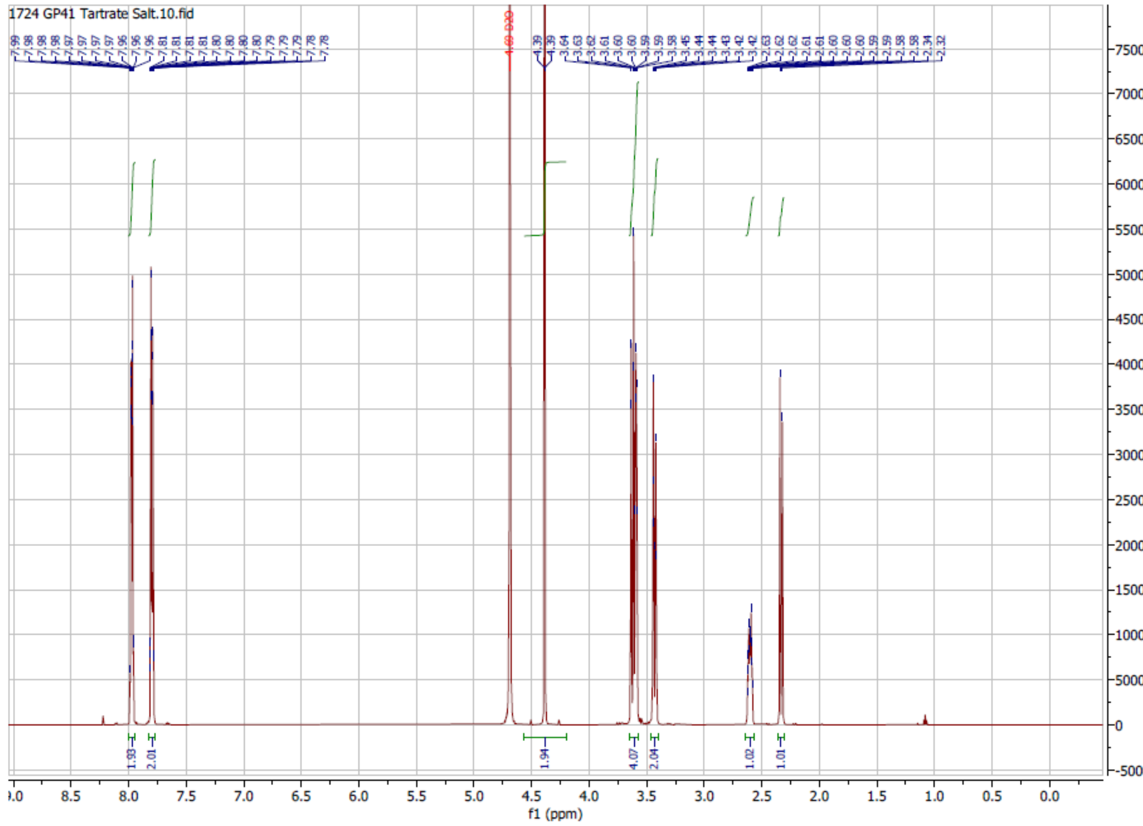

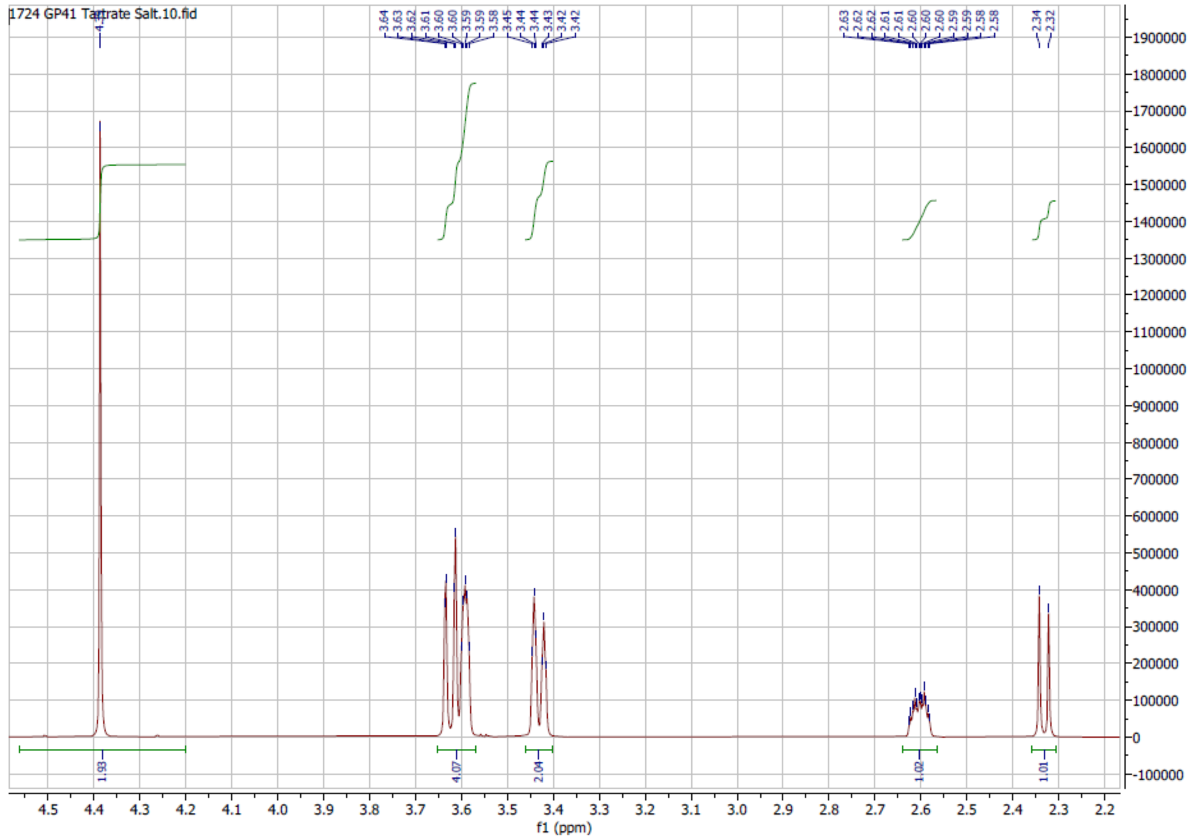

^{1}H NMR of isovarenicline tartrate

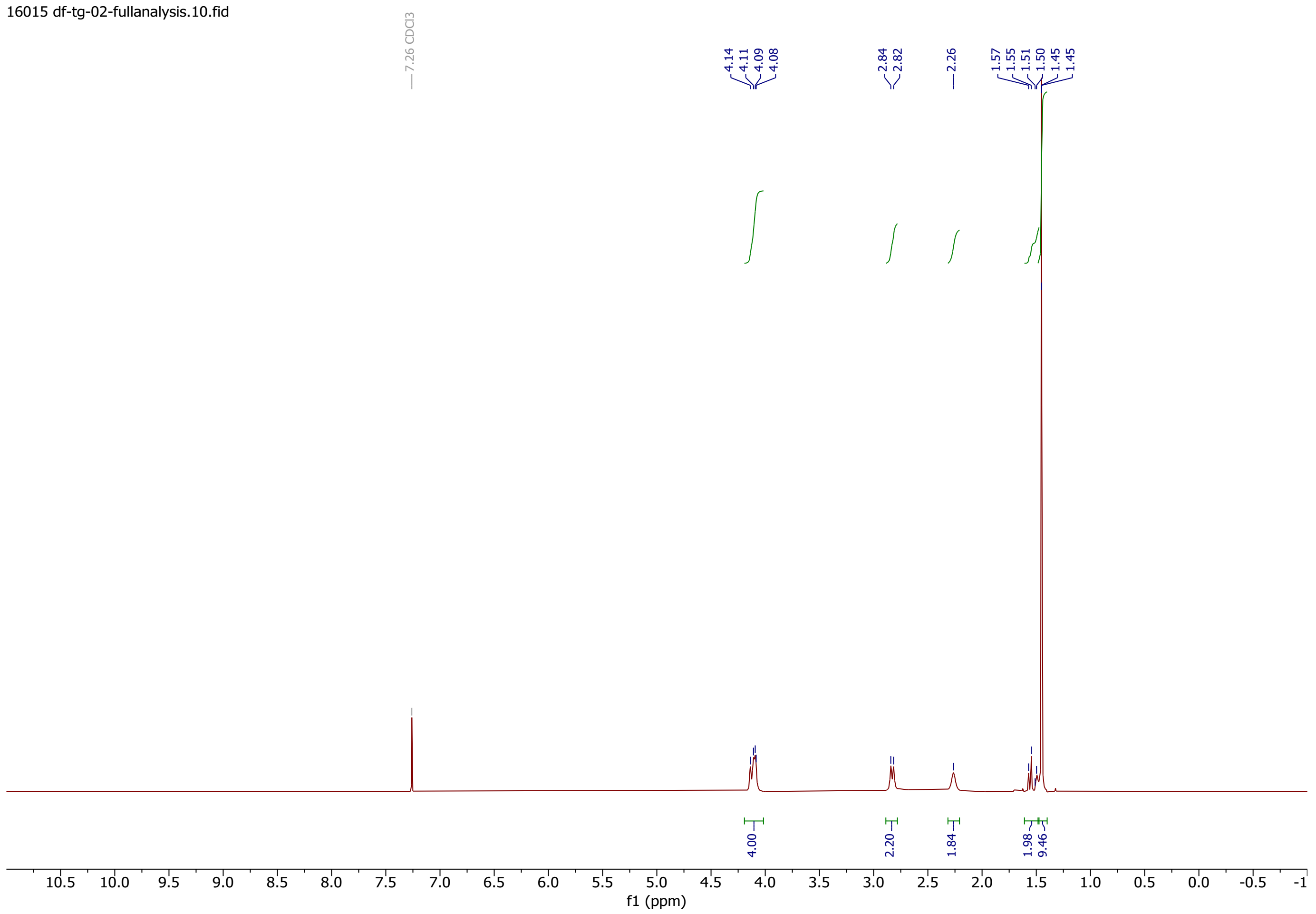

^{1}H NMR of N-Boc diol **SI10**

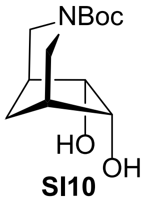

SI10

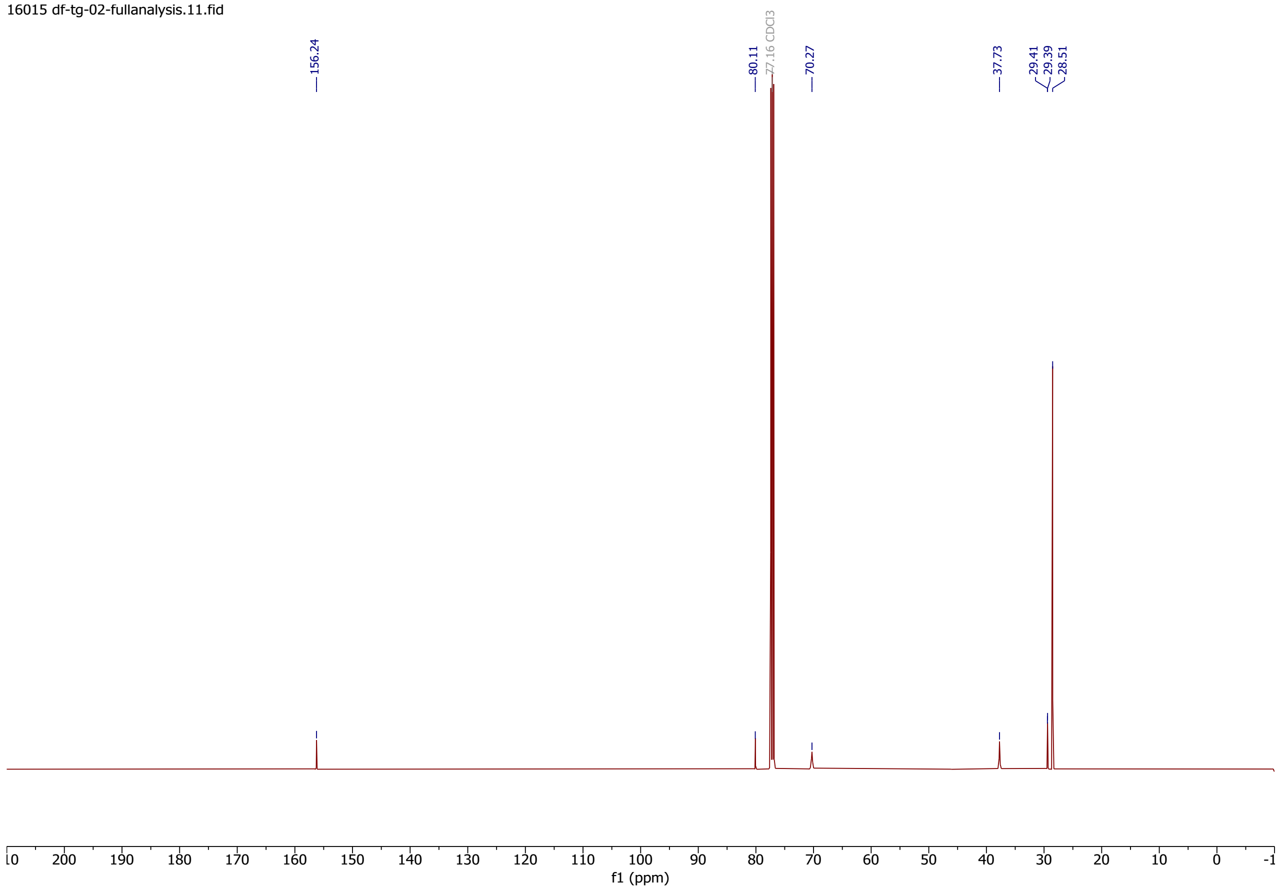

^{13}C NMR of N-Boc diol **SI10**

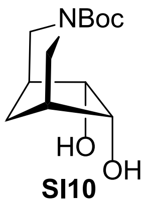

SI10

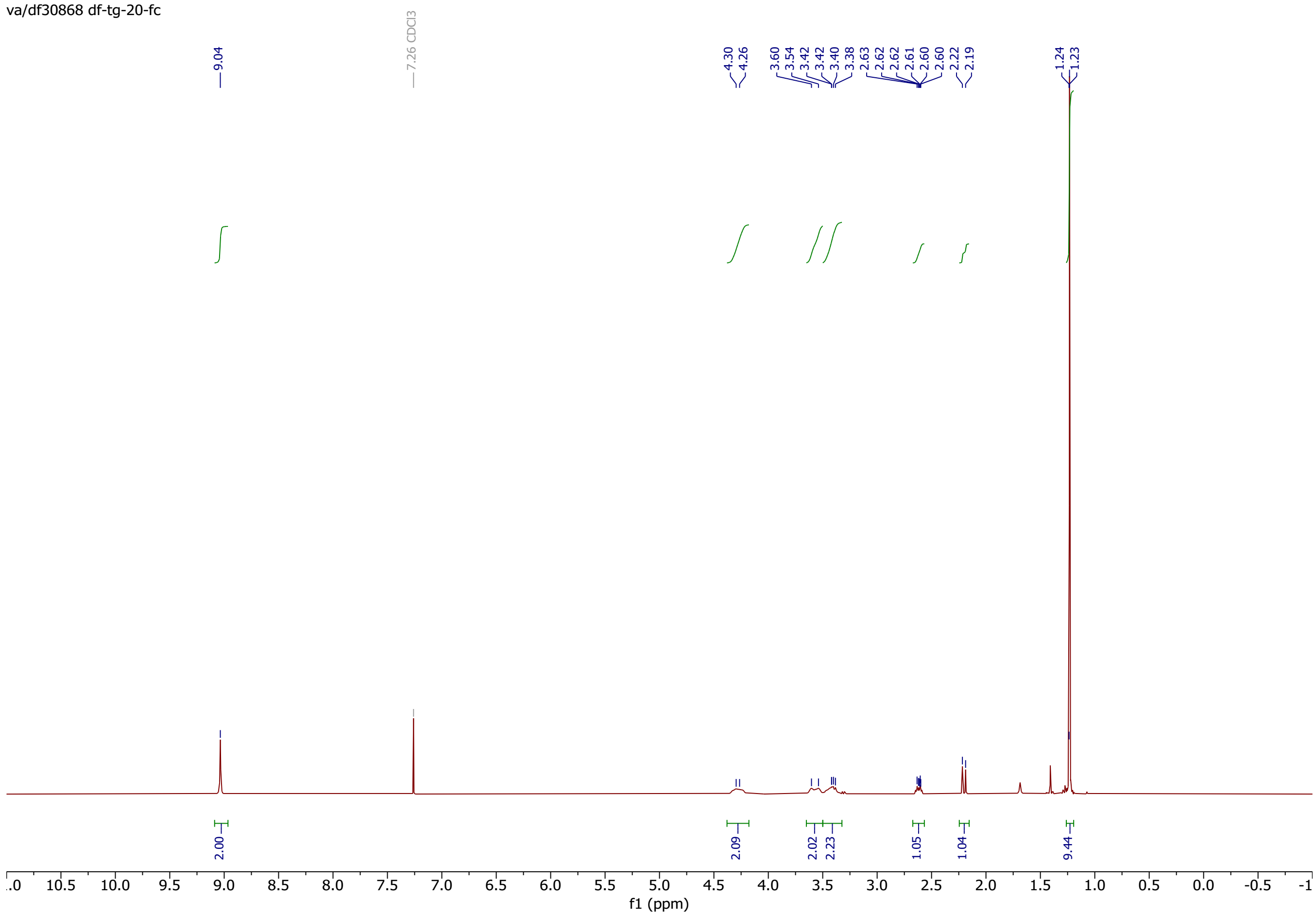

^{1}H NMR of N-Boc N_2 varenicline **SI11**

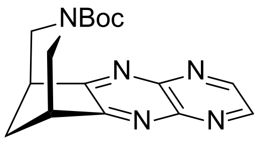

SI11

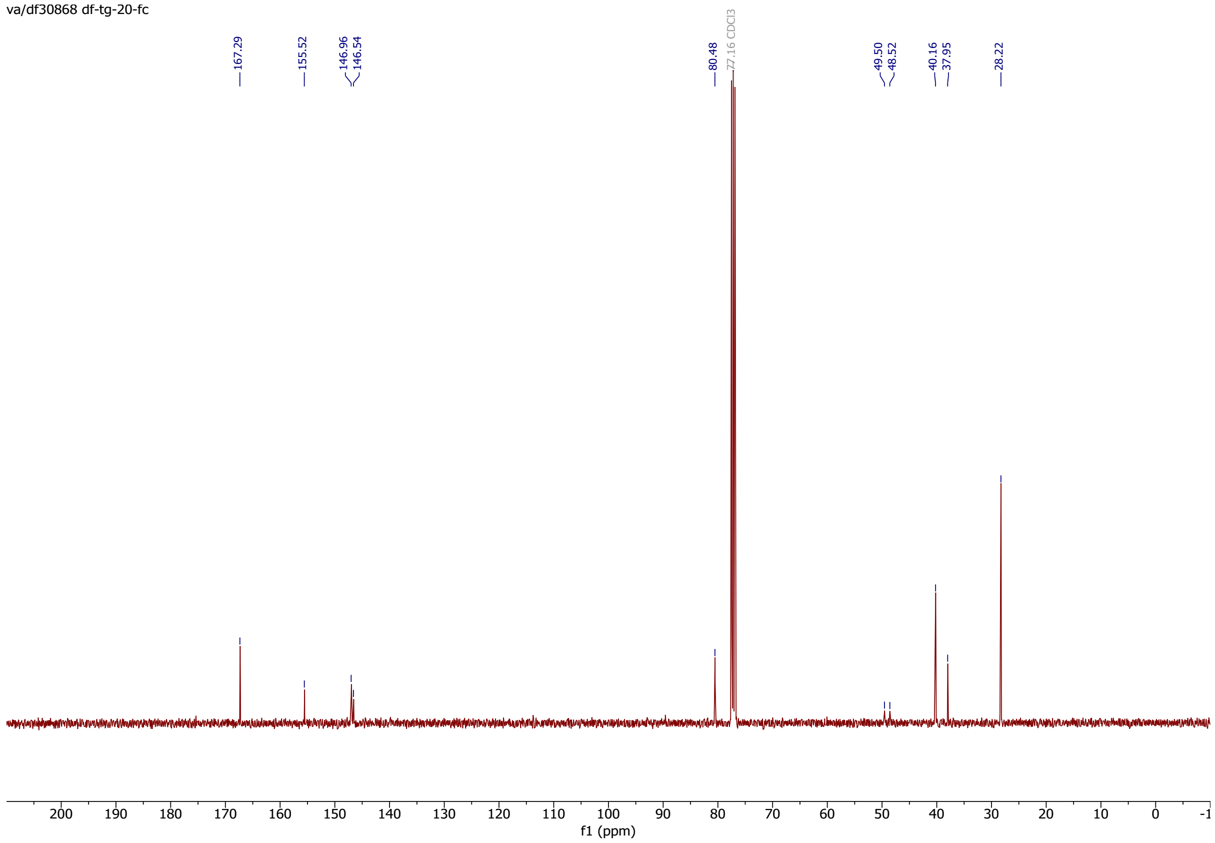

^{13}C NMR of N-Boc N_2 varenicline **SI11**

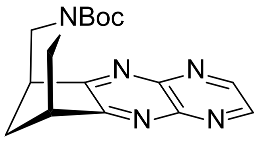

SI11

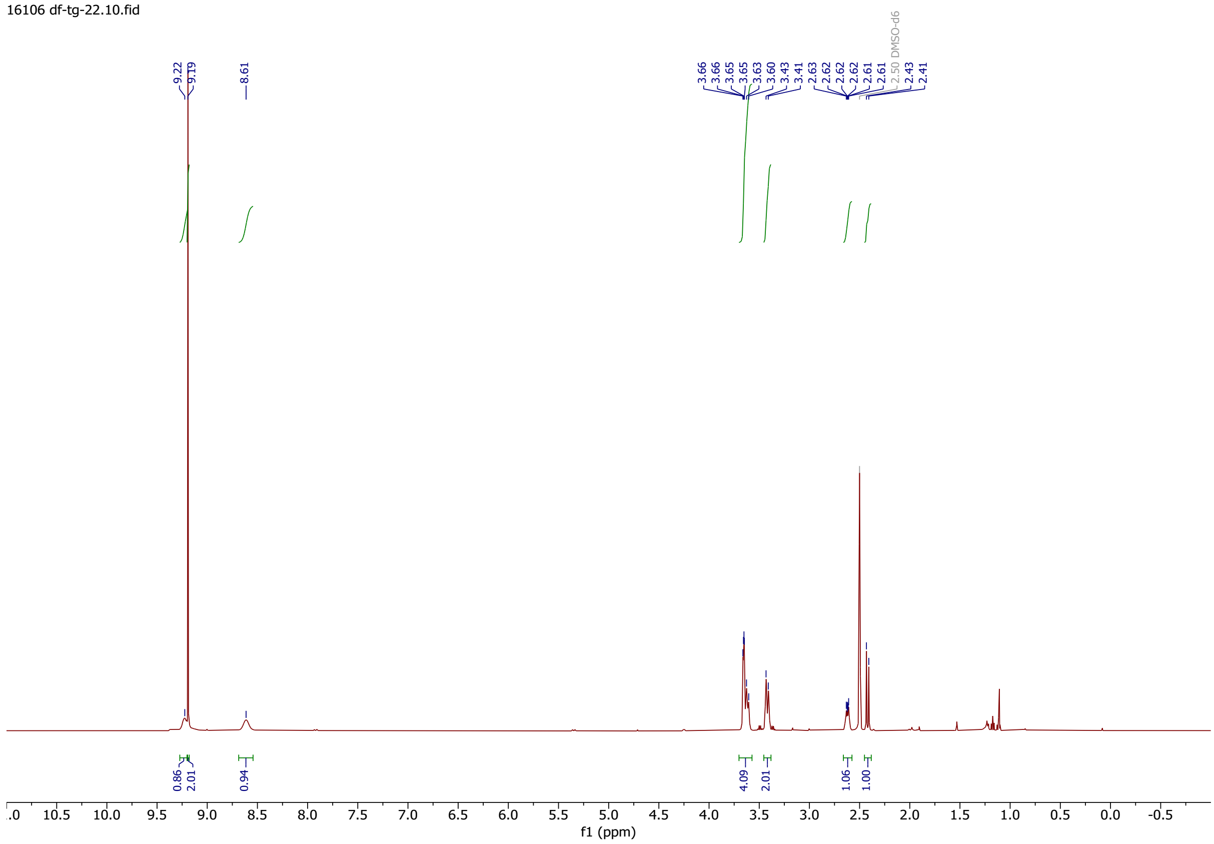

^{1}H NMR of N_2 varenicline TFA **6**

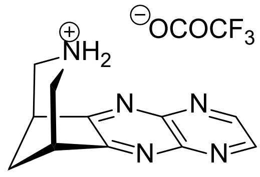

6

16106 df-tg-22.12.fid

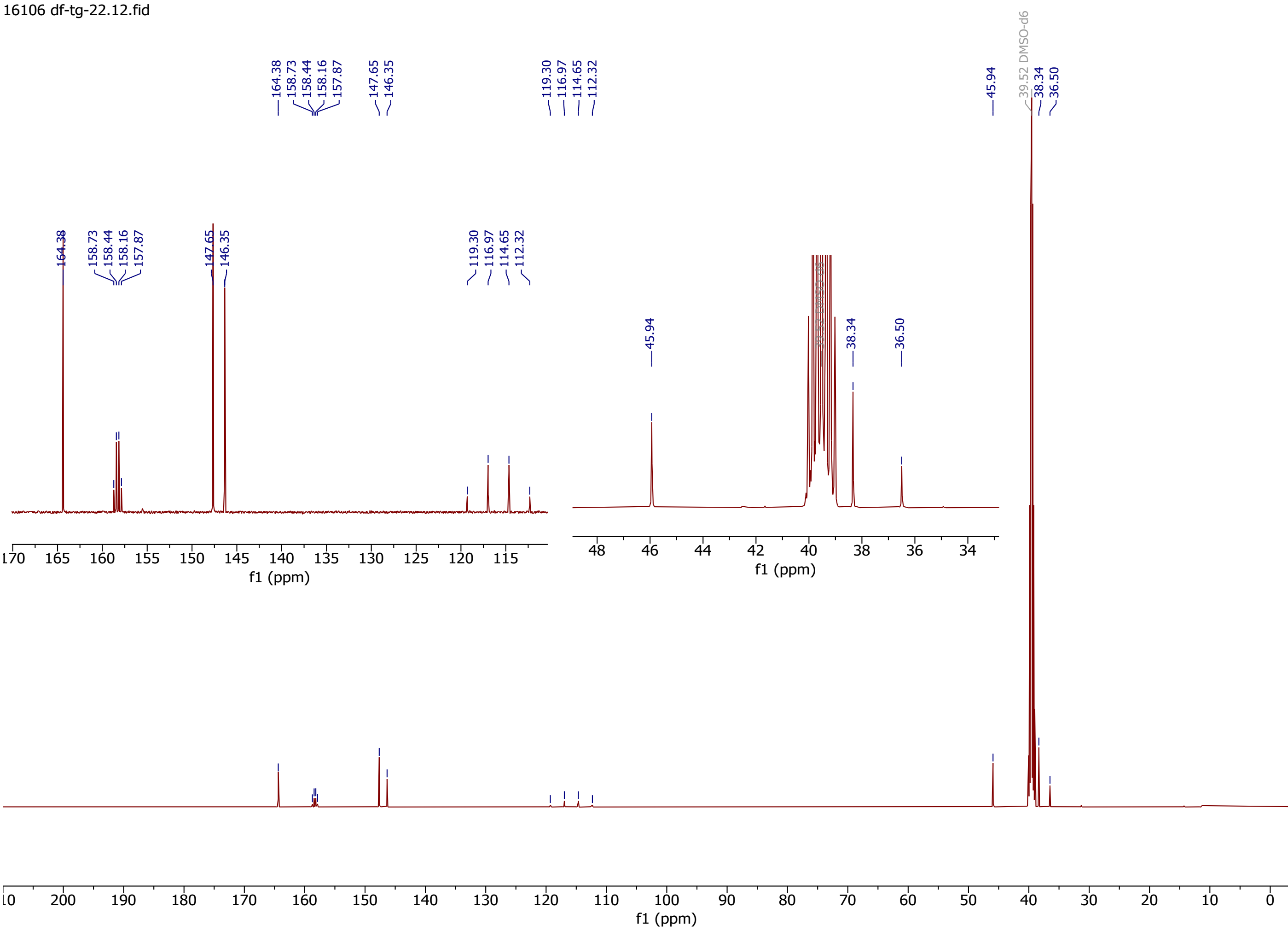

^{13}C NMR (DMSO) of N_2 varenicline TFA **6**

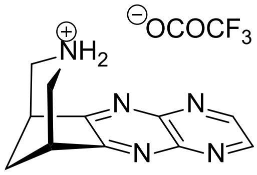

6

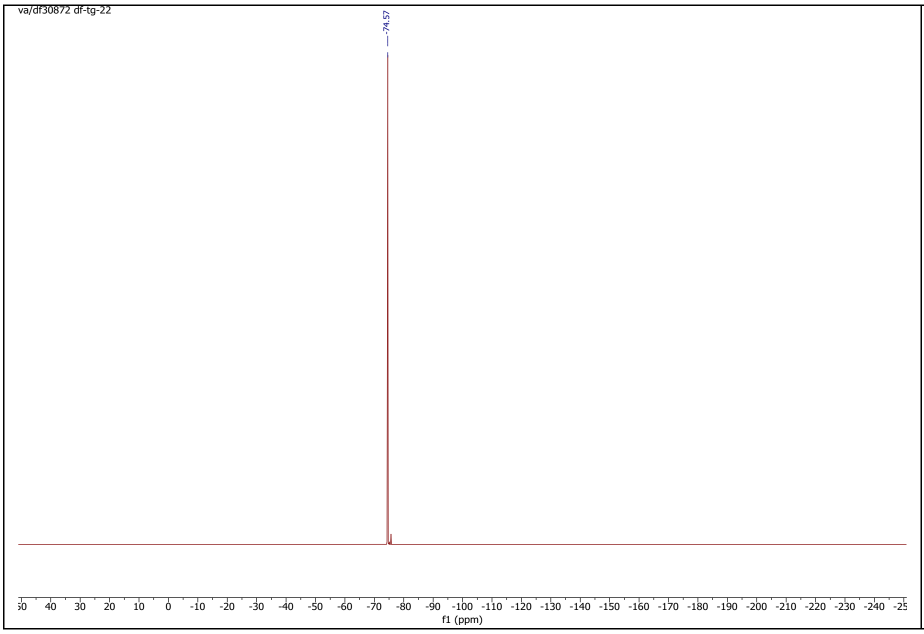

^{19}F NMR of N_2 varenicline TFA **6**

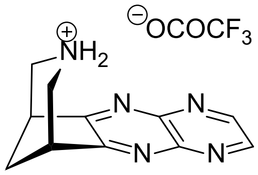

6

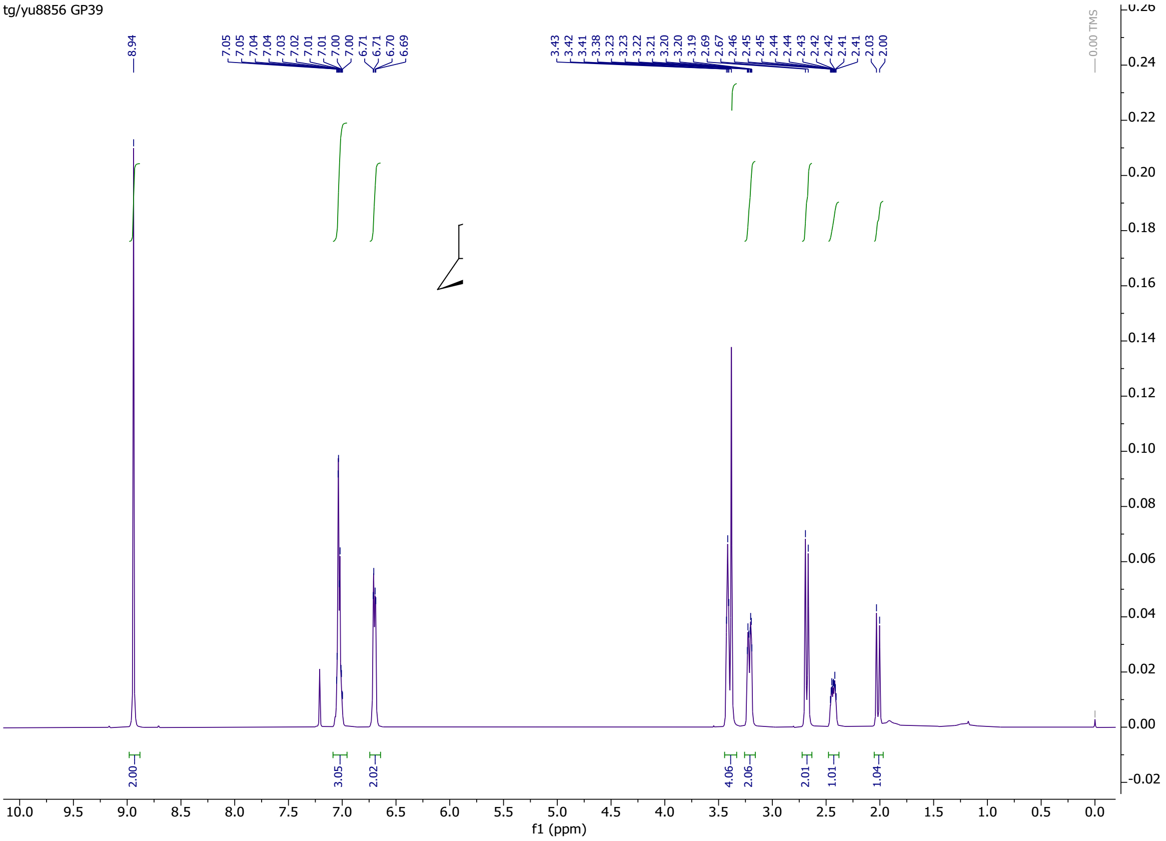

^{1}H NMR of N-Bn N_2 varenicline **SI12**

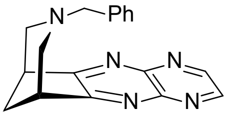

SI12

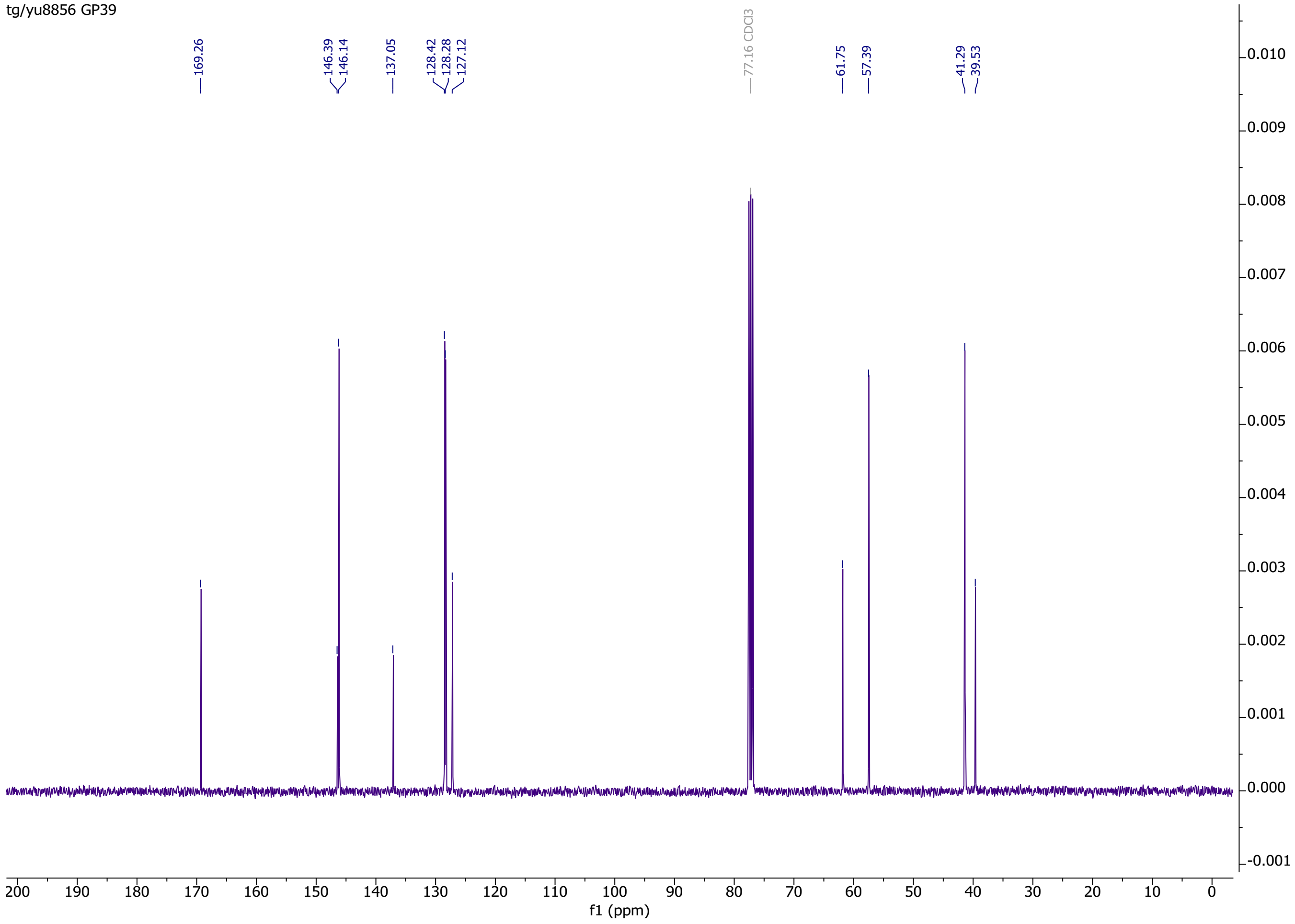

^{13}C NMR of N-Bn N_2 varenicline **SI12**

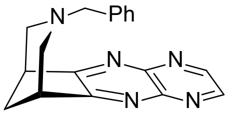

SI12

B. Computational Modelling

(i) Molecular dynamics (MD) simulations

Molecular dynamics (MD) simulations of the extracellular domain (ECD) of human wild-type (WT) $(\alpha4)_3(\beta2)_2$ and $(\alpha4)_2(\beta2)_3$ nicotinic acetylcholine receptors (nAChRs) were performed to identify the key interactions formed by different agonists within the α4-α4 and α4-β2 binding pockets. Since the agonist binding sites are exclusively located in the ECD, the transmembrane and intracellular domains were excluded from the simulations to reduce computational costs. The complexes between the ECD of the human low-sensitivity (LS) isoform of the α4β2 nAChR, namely $(\alpha4)_3(\beta2)_2$, and seven different agonists (varenicline **1**, **Var 1**; nicotine **2**, **Nct 2**; cytisine **3**, **Cyt 3**; C_2 varenicline **4**, **C_2 Var 4**; isovarenicline **5**, **Isovar 5**; N_2 varenicline **6**, **N_2 Var 6;** and, acetylcholine, **ACh**) were constructed using the cryo-EM structure of the complete $(\alpha4)_3(\beta2)_2$ receptor with nicotine bound (PDB code: 6CNK[6]). The binding modes for ACh and cytisine **3** were the same as those previously described in Minguez-Viñas *et.*[7] and Rego-Campello *et al.,*[8] respectively. The binding mode for varenicline **1** and its variants was the same as the one observed in the cryo-EM structure (PDB code: 6UR8[9]).

Complexes between the ECD of the human high-sensitivity (HS) isoform of the α4β2 nAChR, namely $(\alpha4)_2(\beta2)_3$, and varenicline **1** and the three new varenicline variants were also built based on the X-ray structure of the complete HS α4β2 receptor with nicotine bound (PDB code: 5KXI[10]).

All simulated systems contained one agonist bound to each agonist binding site: in the LS isoform, the α-α and both α-β pockets were occupied; conversely, in the HS isoform, each of the two non-consecutive α-β pockets contained one agonist molecule. The structures for the wild-type complexes (Figures S2-S5) were used as the starting inputs for wild-type simulations.

Five mutant complexes formed by the ECD of the human LS α4β2 isoform and varenicline **1**, nicotine **2**, cytisine **3** and ACh were also built and simulated to investigate the dynamic and structural effect of the mutations (Table S1). The mutations introduced were: a serine-to-valine substitution in position 133 in the complementary β2 face of the α-β binding pockets (hereafter named β2S133V), a threonine-to-valine substitution in position 183 in the principal α4 face of the α-β and α-α pockets (α4T183V), a threonine-to-valine substitution in position 139 in the complementary α4 face of the α-α pocket (α4T139V) and two double mutants, one

simultaneously co-expressing the α4T183V and β2S133V substitutions (β2S133Vα4T183V) and the other the α4T139V and β2S133V mutations (β2S133Vα4T139V). Note that the numbering here refers to Uniprot sequences P43681 and P17787 for the human α4 and β2 subunits, respectively. Starting structures for simulations of the mutants were created using the mutagenesis tool in PyMOL.[11]

In this work, a total of 32 different systems were investigated (Table S1). The simulations for ECD of the human HS α4β2 isoform with nicotine **2,** cytisine **3** and ACh were taken from our previous work.[7, 8]

All titratable residues were modelled in their standard state at a physiological pH (i.e. aspartates and glutamates were negatively charged, lysines and arginines were positively charged, and histidines were neutral), similarly to our previous work.[7, 8] All agonists (varenicline **1**, nicotine **2**, cytisine **3**, **C_2** varenicline **4,** isovarenicline **5** and ACh) were considered to be positively charged.

MD simulations were performed using Gromacs.[12] The Amber ff99SB-ILDN forcefield[13] was used to describe the protein, whereas the parameters for varenicline **1**, nicotine **2,** cytisine **3** and ACh were taken from our previous work.[7, 8] Acpype[14] was used to generate Amber-compatible GAFF parameters for **C_2** varenicline **4** and isovarenicline **5**. All systems were solvated using the TIP3P water model.[15] The simulations were performed using a 2-fs time-step for the integration of the equations of motion. Non-bonded long-range electrostatic interactions were calculated using the smooth particle mesh Ewald method,[16] with a Fourier grid spacing of 0.16 Å and a 1.2 Å cutoff for direct contributions. A 12 Å cut-off was also used for the van der Waals interactions, with long-range dispersion corrections for the energy and pressure. The LINCS algorithm[17] was used to constrain bonds in the protein and agonists, and SETTLE[18] was used to keep water molecules rigid.

Prior to the unrestrained simulations, all systems were energy minimized and initialized using the protocol described in Rego-Campello *et al.*[8] Briefly, this procedure involves a three-step energy minimization: in the first step, harmonic restraints were applied to all non-hydrogen atoms; in the second step, to Cα atoms only; and, in the third step, no restraints were used. After energy minimization, a short MD simulation step with all the non-hydrogen atoms restrained was performed, followed by a second short simulation in which position restraints

were applied to Cα atoms only. All the unrestrained simulations started from these relaxed conformations.

Unrestrained simulations were performed at a constant temperature of 310 K using the velocity-rescaling thermostat,[19] with separate couplings for the solutes (protein and ligands) and solvent and a relaxation time constant of 0.1 ps. The pressure within the simulations was maintained at 1 bar using the Parrinello-Rahman barostat[20, 21] with a coupling constant of 1 ps. Each system was simulated three times, each 300 ns, leading to a total of 27 μs of simulation time across all systems. MD input and output files (including the simulation trajectories) are publicly available *via* the University of Bristol Research Data Repository (https://data.bris.ac.uk/).

(ii) Analysis of MD simulations

The trajectories were analyzed using Gromacs tools.[12] The structural stability of the simulated systems was examined by monitoring the Cα root mean square deviation (RMSD) relative to the starting structures. All systems remained stable over the simulation time, with the average Cα RMSD profiles showing a plateau after ~20 ns (Figures S7 and S27). The receptor's secondary structure content was monitored using the DSSP software,[22] with minimal secondary structure loss after the simulation time (Figures S8 and S27).

Principal component analysis (PCA) was performed to examine the sampling and equilibration of the replicates (Figure S9), as previously described.[23, 24] All replicates were combined before the analysis so that they all shared a common subspace, and their behavior could be directly compared. Each trajectory used for PCA contained one conformation per nanosecond per replicate with the protein Cα atoms. The two principal components (PC) 1 and 2 were used to assess the sampling and equilibration of the simulations. Generally, the different replicates sampled different regions of conformational space, thus improving the overall sampling for each system and helping to mitigate sampling problems.

Cα root mean square fluctuations (RMSF) were calculated to characterize the dynamic behavior of the receptor across the various systems (Figures S10 and S28), and a Student's t-test was used to assess the significance of the differences observed between wild type and mutants (similarly to Oliveira *et al.*[23]) (Figure S10). A sample size of three was used for the t-

test, which assumed the two samples were independent and the dependent variable was normally distributed.

The RMSD of the agonists was monitored to assess the stability of their initial binding poses (Figures S11-S13 and S27). Despite changes in binding mode observed for some ligands (e.g. ACh in the α-α binding pocket), all agonists remained bound to their respective binding sites in both the wild-type and mutant systems. The only exception was the ACh molecule bound to the second α-β pocket of replicate 3 in the β2S133Vα4T139V-ACh system, which exited the pocket after about 112 ns (Figure S13G). This analysis revealed distinct dynamic behaviors between agonists: ACh exhibited high positional and conformational variability; in contrast, the bulkier ligands, probably due to additional interactions with the protein compared to ACh,[25-29] showed reduced mobility and generally maintained an orientation closer to the initial one throughout the simulation time. Differences in agonist dynamics were also observed between the α–α and α–β pockets, mainly for nicotine **2** and cytisine **3**, with these ligands showing increased mobility within the α–α pocket.

Probability density maps were generated to visualize the spatial distribution and identify preferred positions for the agonists (Figures S14-S16 and S30) for the wild-type and mutant complexes. For each agonist-receptor system, the maps were calculated by combining the entire trajectories for all replicates of that system.

Statistical correlations between the protonated nitrogen atom of varenicline **1**, nicotine **2,** cytisine **3** and ACh and all the Cα atoms in the wild-type receptor were determined to identify the regions in the protein whose motions are coupled to the ligands (Figure S22). The correlations were determined by combining all replicates' trajectories for each individual system, with each trajectory containing a total of 90001 conformations.

The distance between the ammonium centers present in the ligands (piperidines in the case of varenicline **1** and its derivatives and cytisine **3;** pyrrolidine of nicotine **2**; and quaternary ammonium nitrogen of ACh) and the side chains of TrpB (Trp182 located in loop B in the principal α4 face of the pockets), TyrA (Tyr126 in loop A in the principal α4 face of the pockets), and TrpD (Trp88 and Trp82 in loop D in the complementary α4 face of the α–α pocket and the complementary β2 face of the α–β pocket) were determined (Figures S17-S19 and S29). These

distances indicate persistent cation-π interactions between the ligands and TrpB, and occasionally with TyrA and TrpD.

(iii) Supporting figures and tables

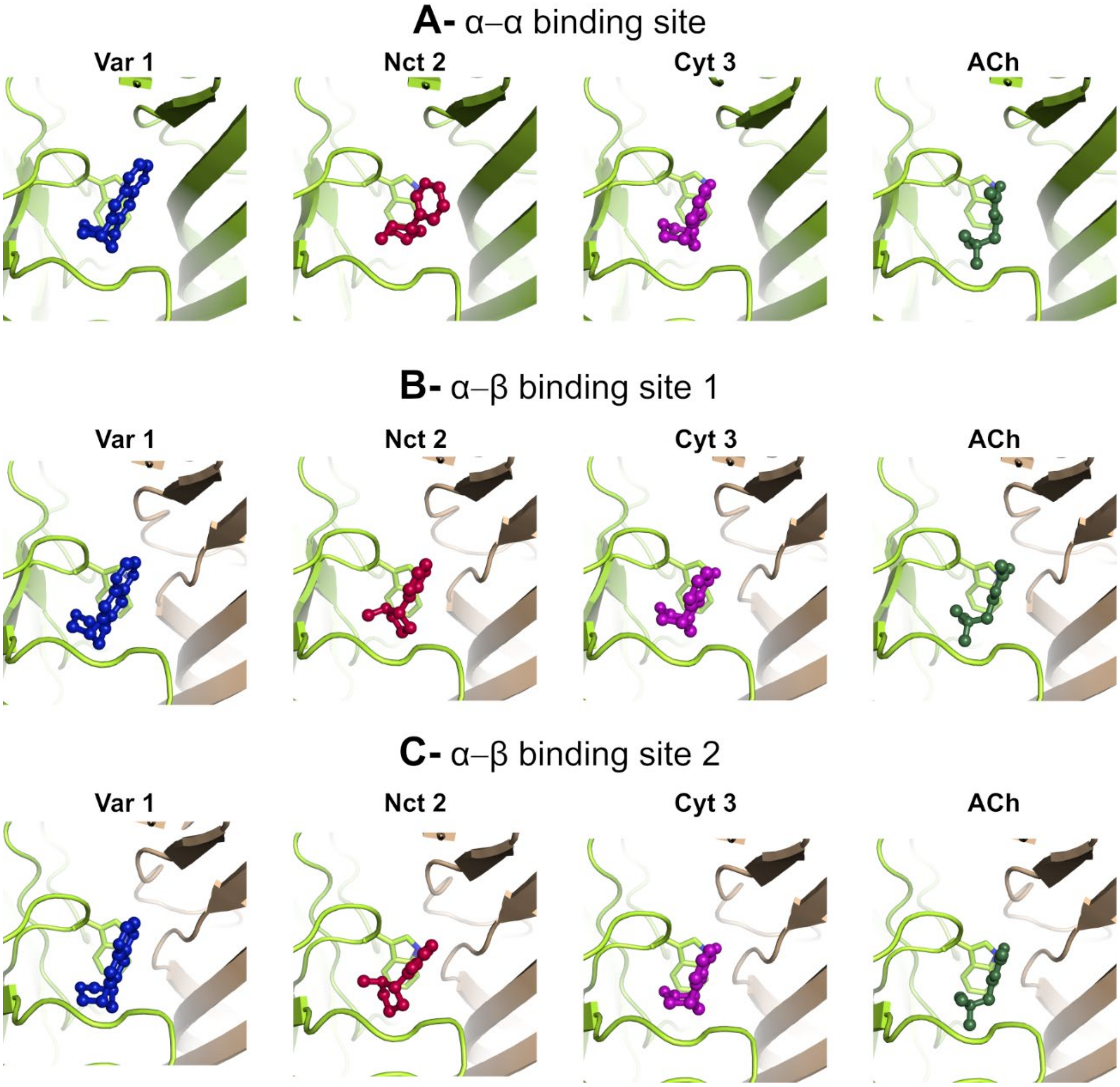

Figure S2- Agonist binding mode in the α-α and α-β pockets in the wild-type LS isoform of the α4β2 nAChR. (**A**) Binding mode of **1**, nicotine **2**, cytisine **3,** and ACh in the α-α pocket. (**B**) Binding mode of varenicline **1**, nicotine **2**, cytisine **3** and ACh in the first α-β pocket (**C**) Binding mode of varenicline **1**, nicotine **2**, cytisine **3** and ACh in the second α-β pocket. Agonists are represented as balls-and-sticks and TrpB (W182 in the principal α4 face), a tryptophan residue that provides the anchor point for the agonist, shown with sticks. The α4 and β2 subunits are colored in yellow and light brown, respectively.

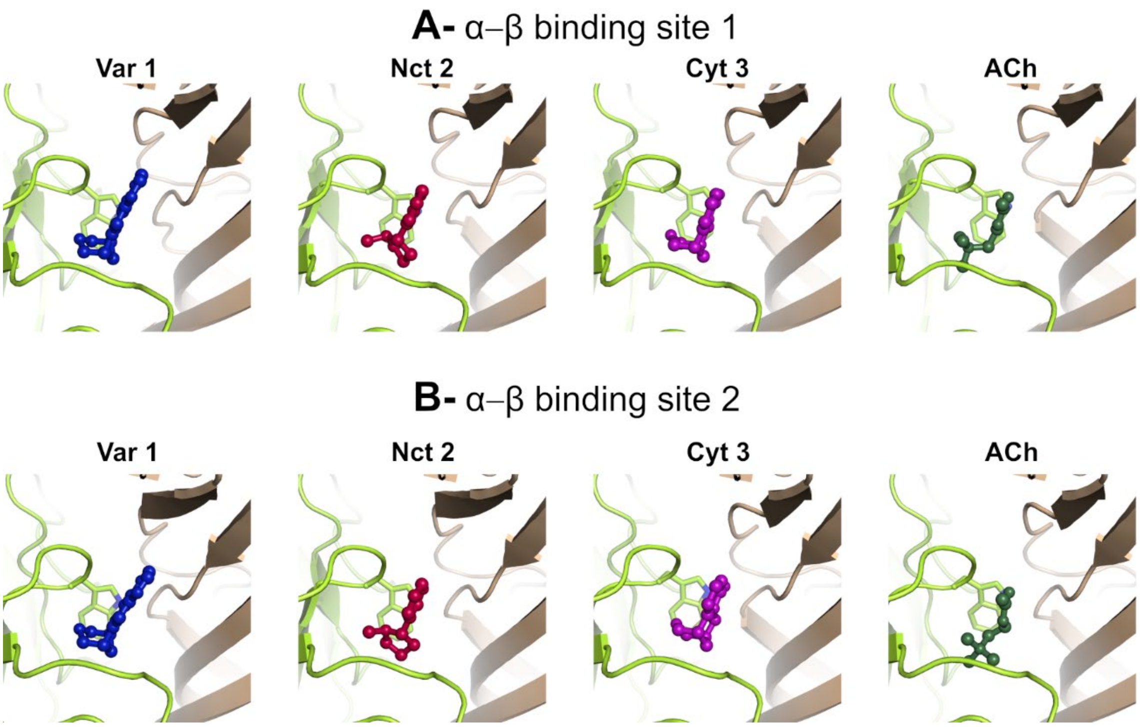

Figure S3- Agonist binding mode in the α-β pockets in the wild-type HS isoform of the α4β2 nAChR. (**A**) Binding mode of varenicline **1**, nicotine **2**, cytisine **3**, and ACh in the first α-β pocket (**B**) Binding mode of varenicline **1**, nicotine **2**, cytisine **3**, and ACh in the second α-β pocket. Agonists are represented as balls-and-sticks and TrpB (W182 in the principal α4 face), a tryptophan residue that provides the anchor point for the agonist, shown with sticks. The α4 and β2 subunits are colored in yellow and light brown, respectively.

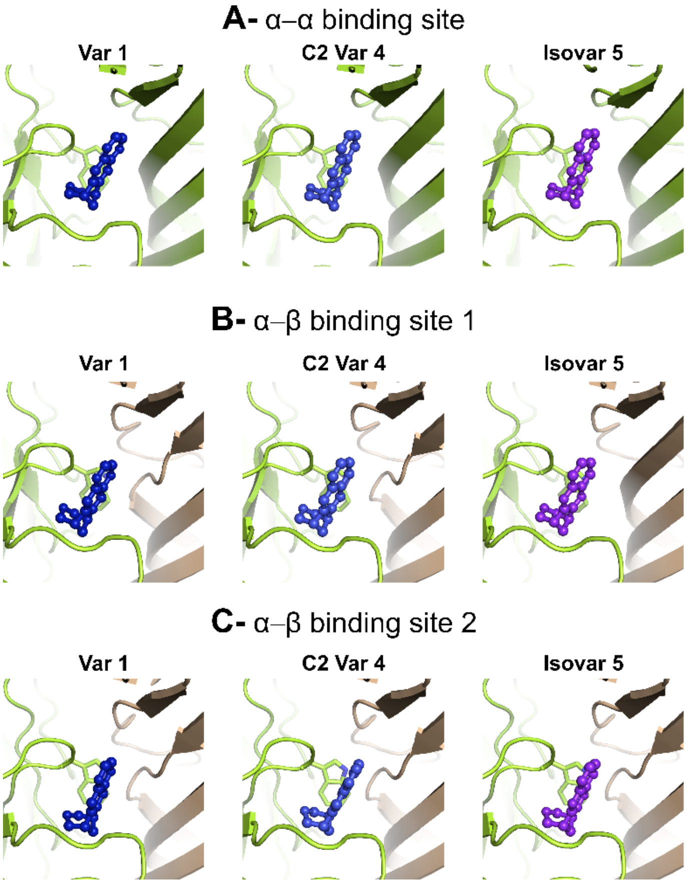

Figure S4- Binding mode of varenicline **1** and its variants in the α-α and α-β pockets of the wild-type LS form of the α4β2 nAChR. (**A**) Binding mode of varenicline **1**, C_2 varenicline **4,** and isovarenicline **5** in the α-α pocket. (**B**) Binding mode of varenicline **1**, C_2 varenicline **4**, and isovarenicline **5** in the first α-β pocket (**C**) Binding mode of varenicline **1**, C_2 varenicline **4**, and isovarenicline **5** in the second α-β pocket. Agonists are represented as balls-and-sticks and TrpB (W182 in the principal α4 face), a tryptophan residue that provides the anchor point for the agonist, shown with sticks. The α4 and β2 subunits are colored in yellow and light brown, respectively.

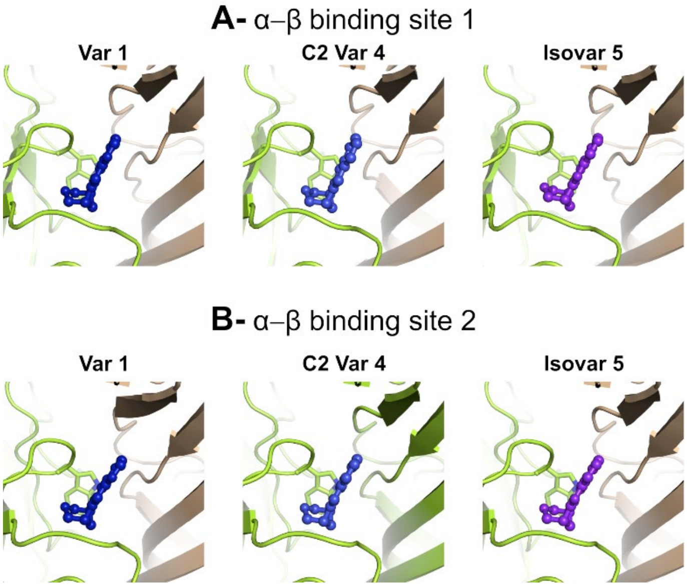

Figure S5- Binding mode of varenicline **1** and its variants in the α-β pockets of the wild-type HS isoform of the α4β2 nAChR. (**A**) Binding mode of varenicline **1**, C_2 varenicline **4**, and isovarenicline **5** in the first α-β pocket (**B**) Binding mode of varenicline **1**, C_2 varenicline **4**, and isovarenicline **5** in the second α-β pocket. Agonists are represented as balls-and-sticks and TrpB (W182 in the principal α4 face), a tryptophan residue that provides the anchor point for the agonist, shown with sticks. The α4 and β2 subunits are colored in yellow and light brown, respectively.

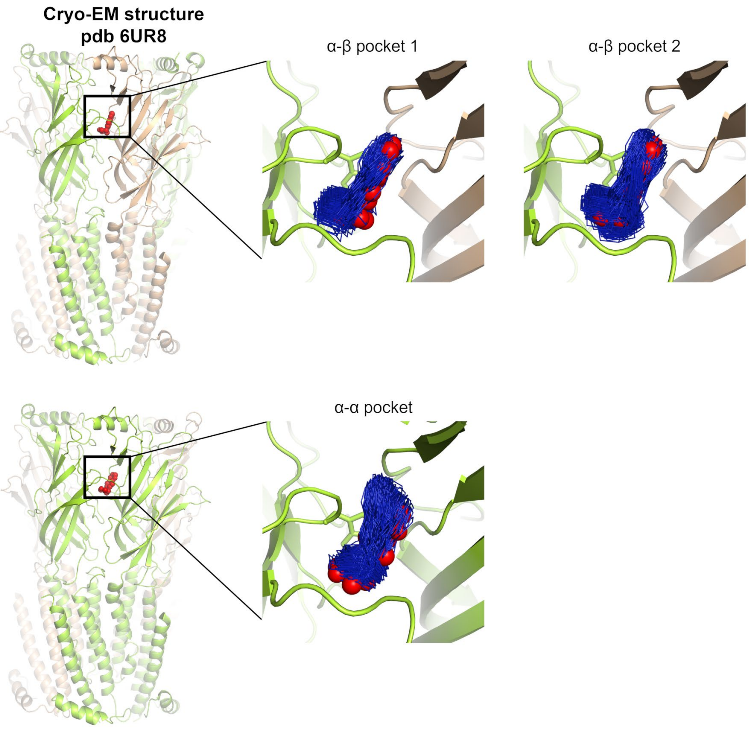

Figure S6- Comparison of varenicline **1** binding mode in the cryo-EM structure 6UR8[9] (red spheres) and during the MD simulations (blue sticks) in the α4β2 nAChR. The blue sticks represent the binding modes of varenicline **1** throughout the simulations, sampled at one frame per nanosecond per replicate. These binding modes are illustrated within both the α-β and α-α pockets of the LS isoform of the wild-type α4β2 nAChR. The anchoring residue TrpB (W182 in the principal α4 face) is shown in yellow sticks. The α4 and β2 subunits are colored in yellow and light brown, respectively.

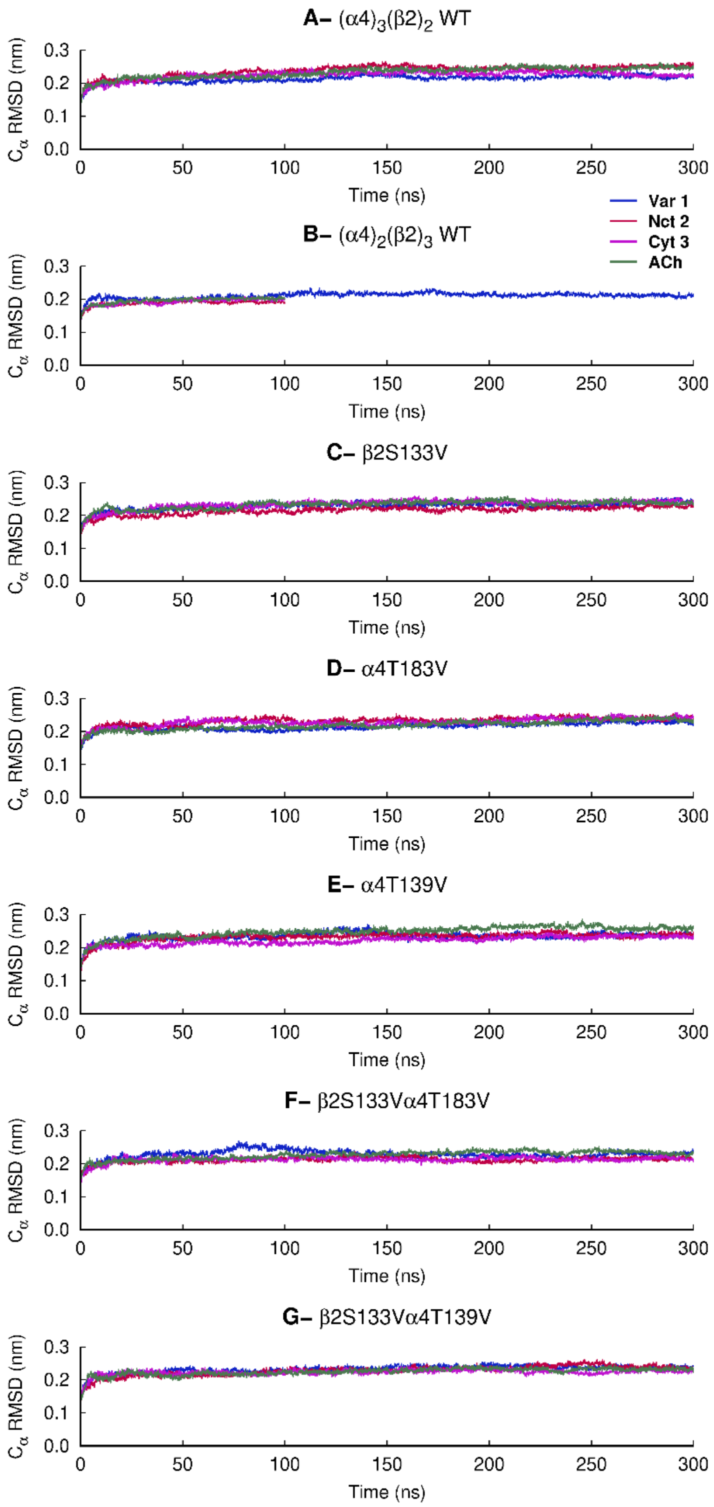

Figure S7- Temporal evolution of the average Cα RMSD for the varenicline **1**, nicotine **2**, cytisine **3** and ACh-bound systems. The Cα RMSD was calculated relative to each system's starting structure and the averages obtained over all replicates for each system. Please note that the simulations of the complexes formed by the HS isoform of the α4β2 wild-type receptor and nicotine **2**, cytisine **3** and ACh (panel B) were taken from our previous work.[7,8] Please zoom in on the image for detailed visualization.

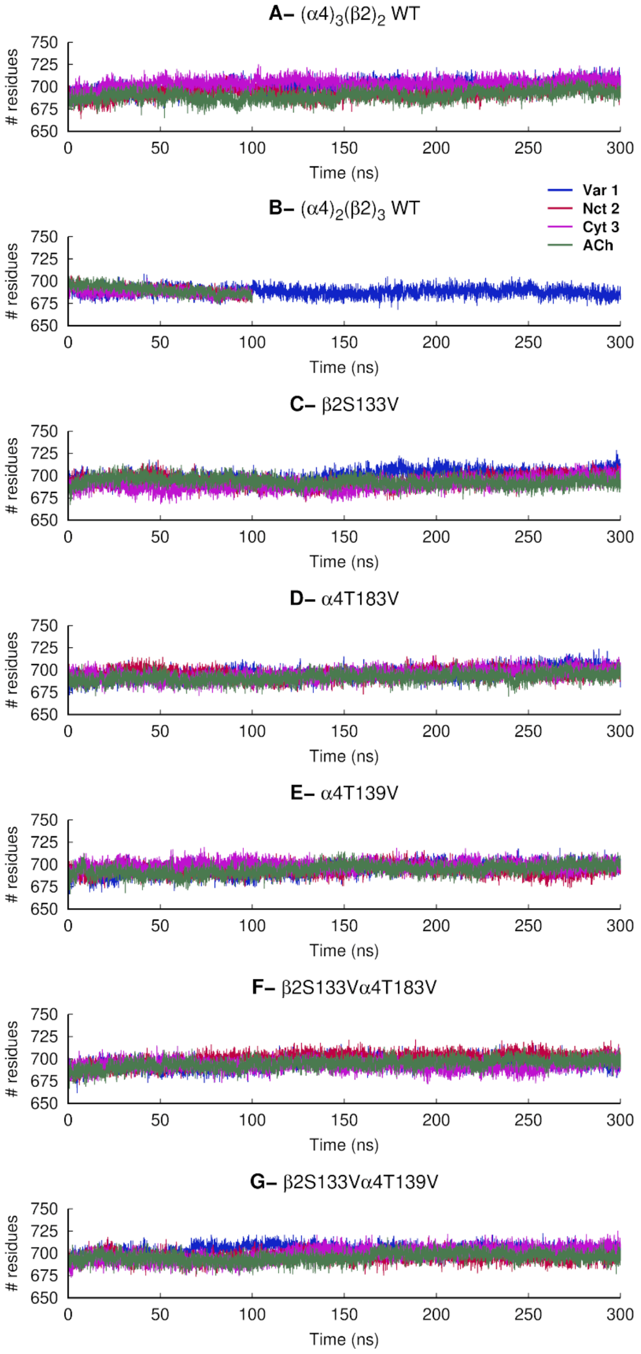

Figure S8- Temporal evolution of the number of residues involved in secondary structure features for the varenicline **1**, nicotine **2**, cytisine **3** and ACh-bound systems. The secondary structure assignment was performed with the DSSP software[22] and includes all residues assigned to α-helix, π-helix, 3_{10}- helix, 5-helix, β-sheet, β-strand and β-bridge secondary structure classes. The averages were obtained over all replicates for each system. The trajectories for the complexes formed by the HS isoform of the α4β2 wild-type receptor and nicotine **2**, cytisine **3** and ACh (in panel B) were taken from our previous work.[7, 8] Please zoom in on the image for detailed visualization.

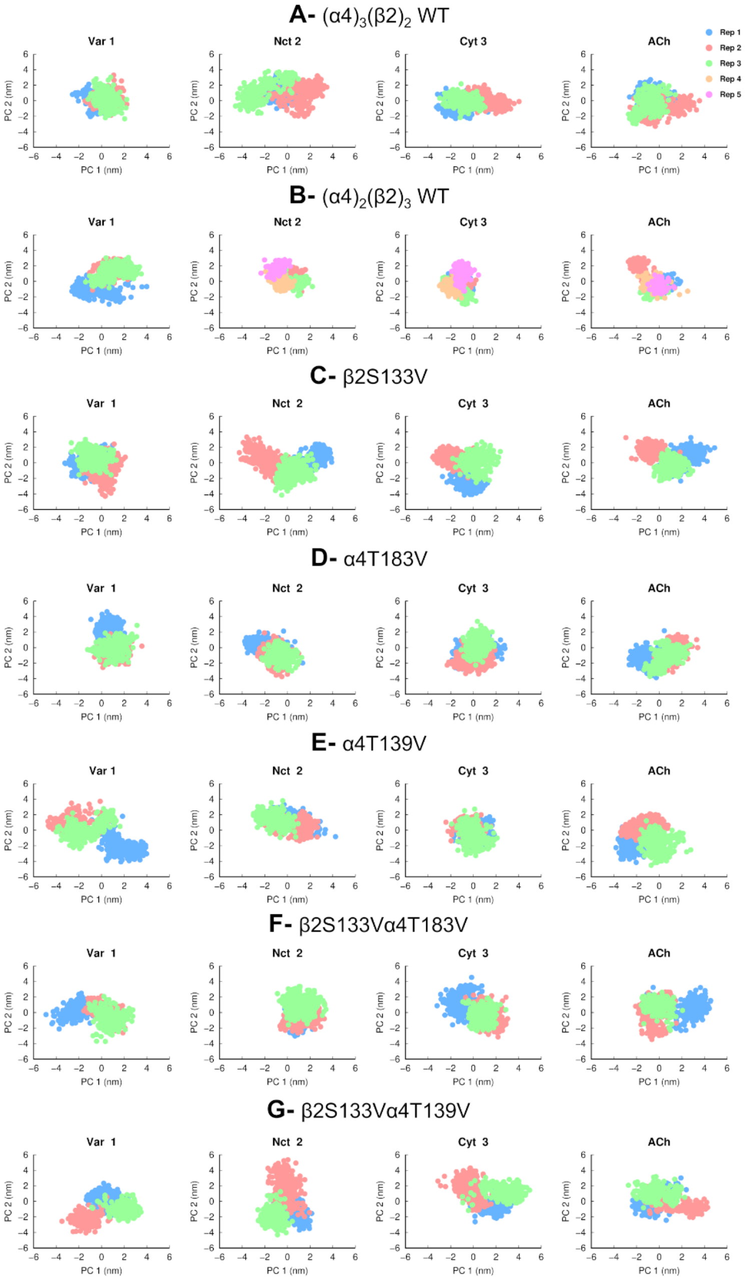

Figure S9- Principal component analysis (PCA) of the replicates for the LS (**A**) and HS (**B**) isoforms of the α4β2 wild-type and mutant (**C-G**) simulations. All replicates for each system were combined before the analysis so that they all shared a common space, and their behavior could be directly compared. Each trajectory used for PCA contained one conformation per nanosecond per replicate with the protein Cα atoms. Principal component (PC) 1 and 2 were used to assess the sampling of the conformational space. This analysis shows that, generally, the different replicates explore different regions of the space, thus improving the overall sampling for each system. Please zoom in on the image for detailed visualization.

A- β2S133V

Var 1

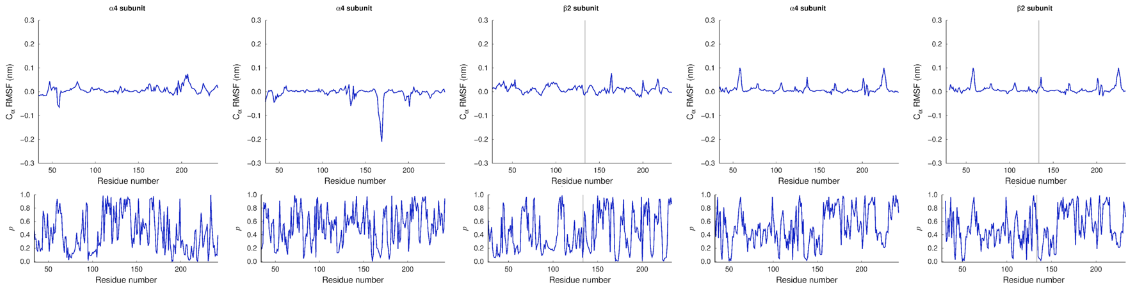

Nct 2

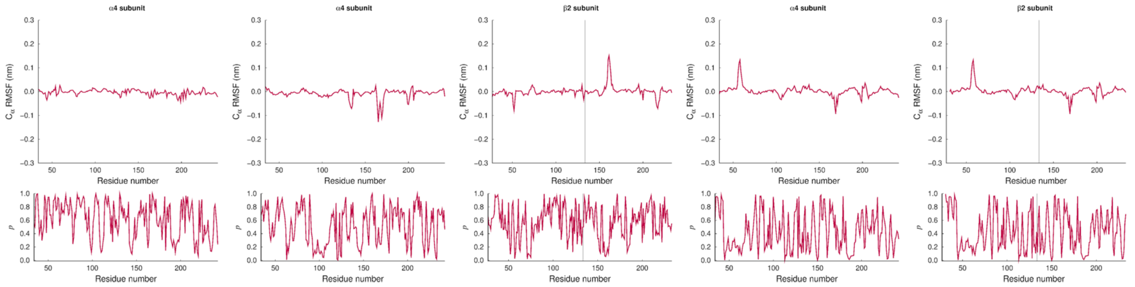

Cyt 3

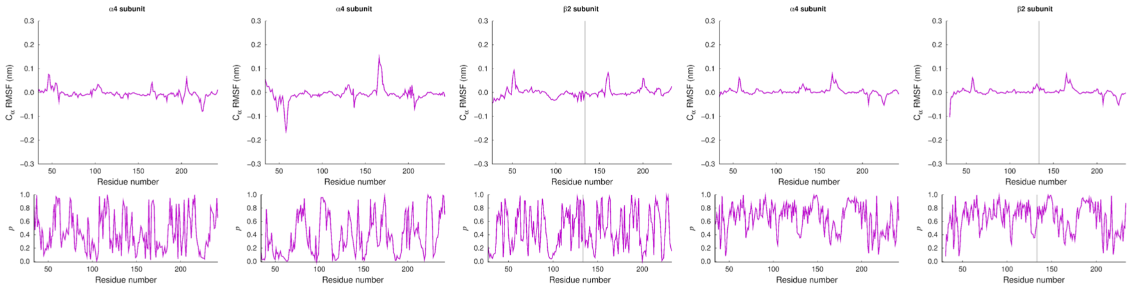

ACh

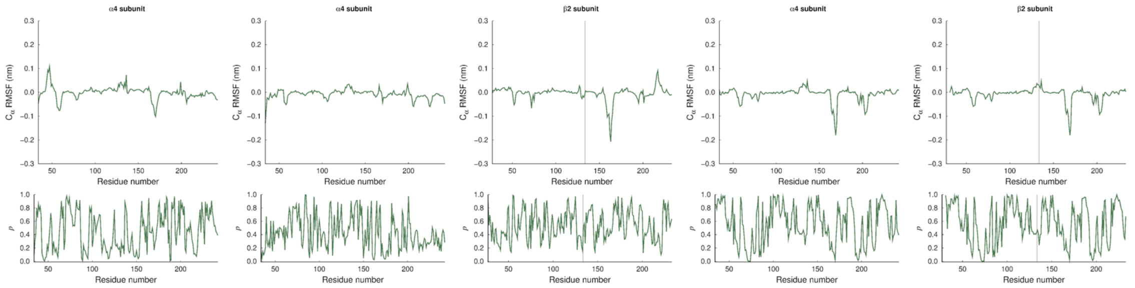

B- α4T183V

Var 1

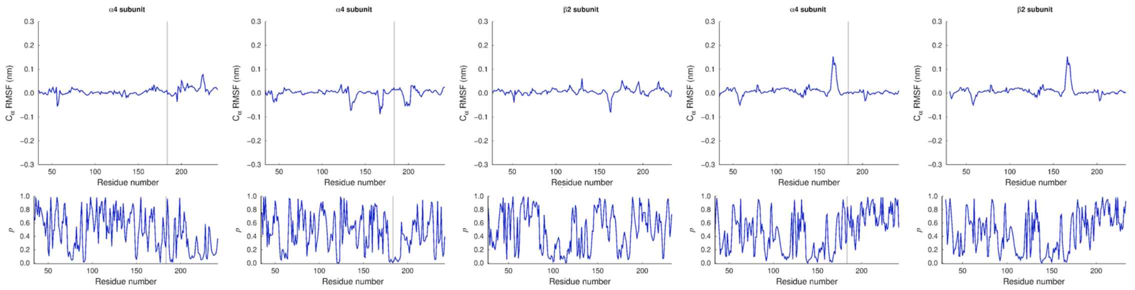

Nct 2

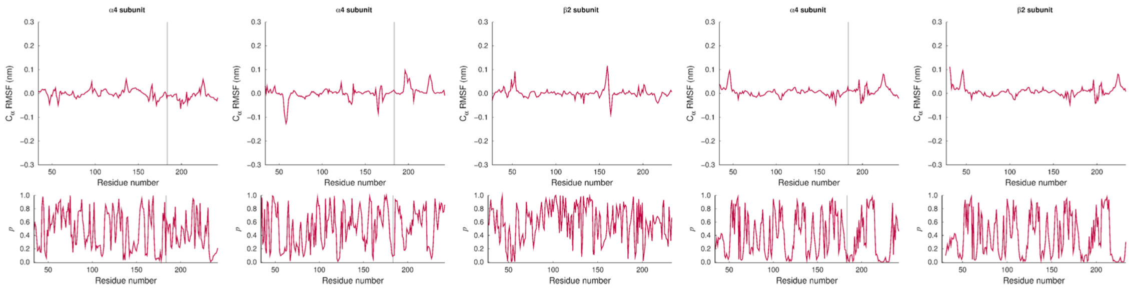

Cyt 3

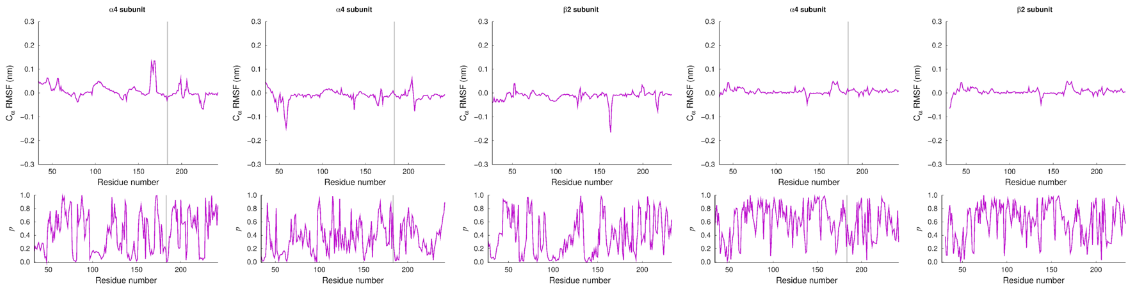

ACh

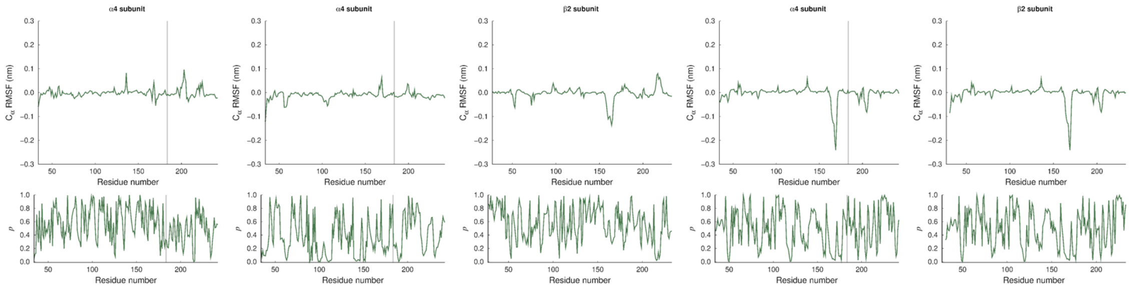

C- α4T139V

Var 1

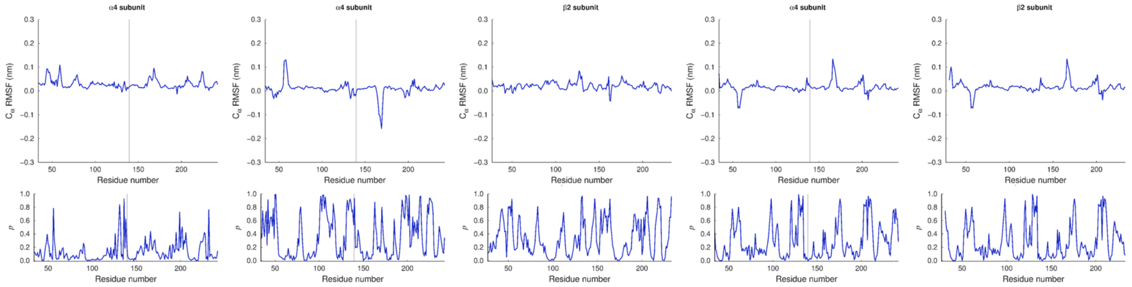

Nct 2

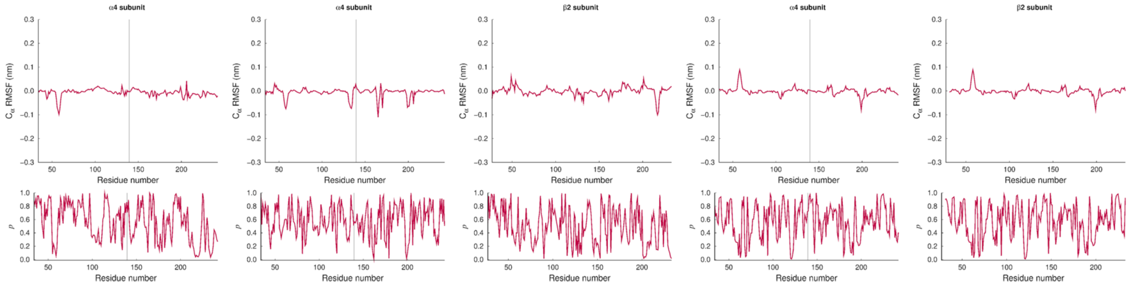

Cyt 3

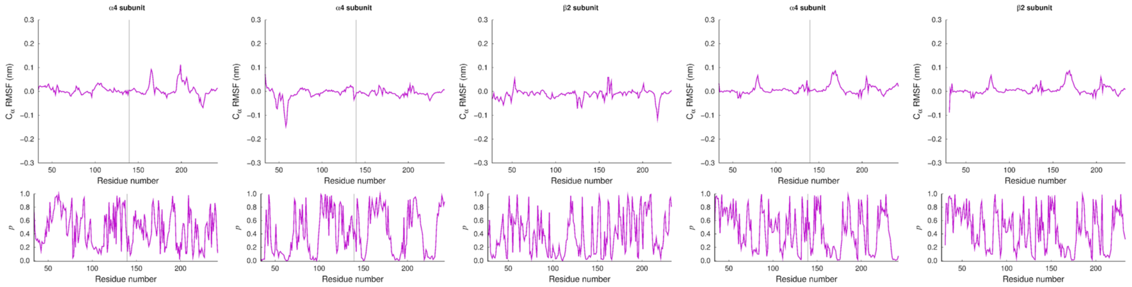

ACh

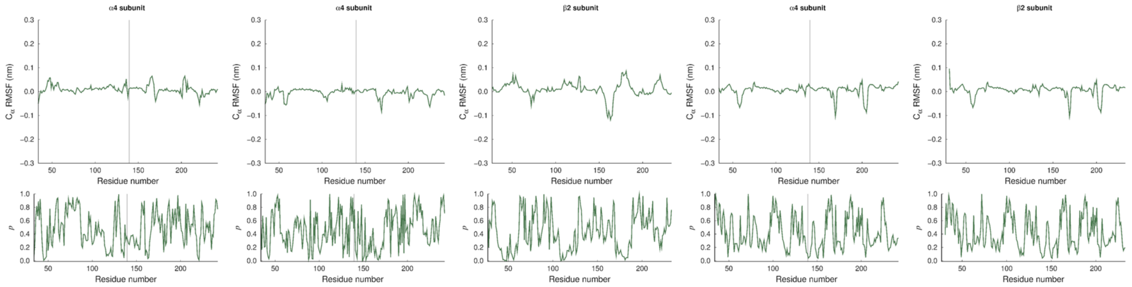

D- β2S133Vα4T183V

Var 1

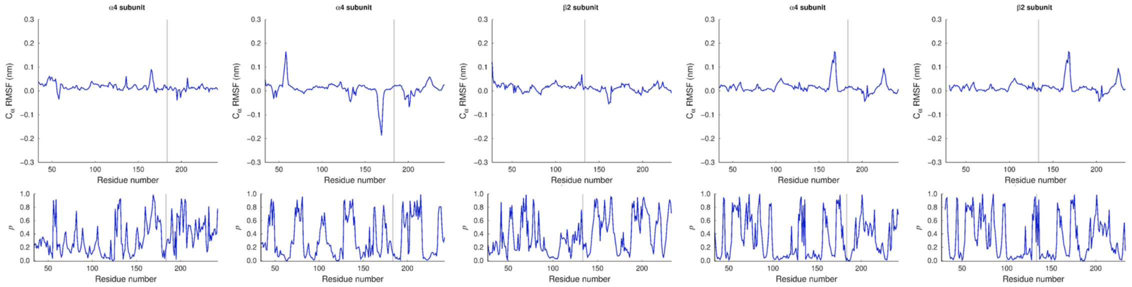

Nct 2

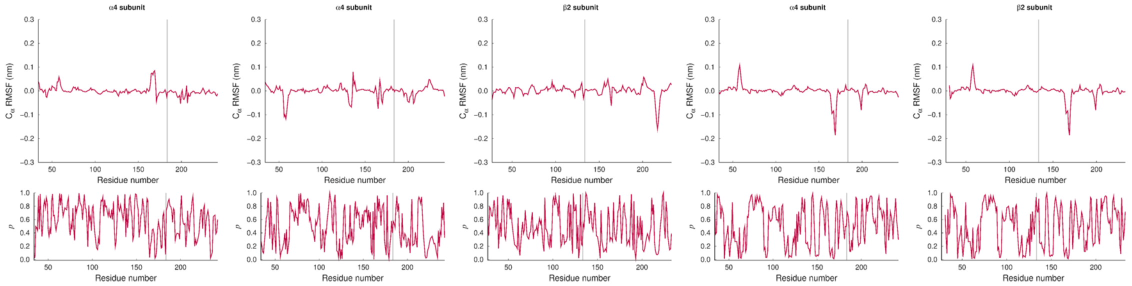

Cyt 3

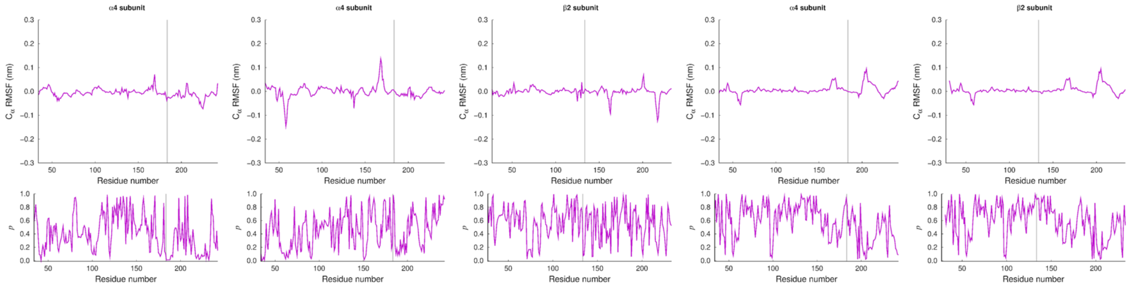

ACh

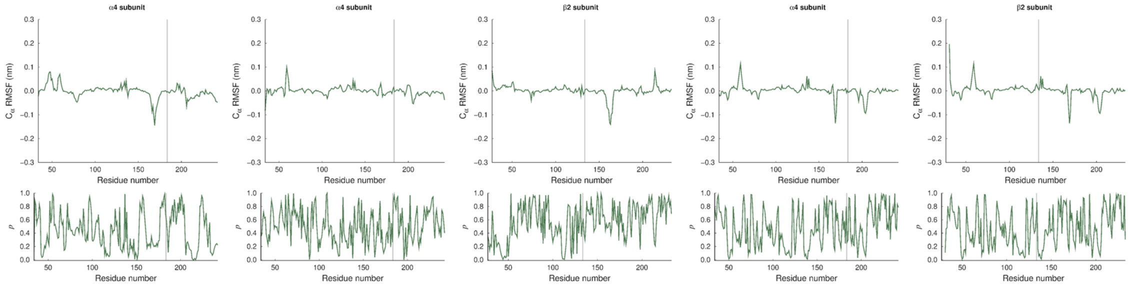

E- β2S133Vα4T139V

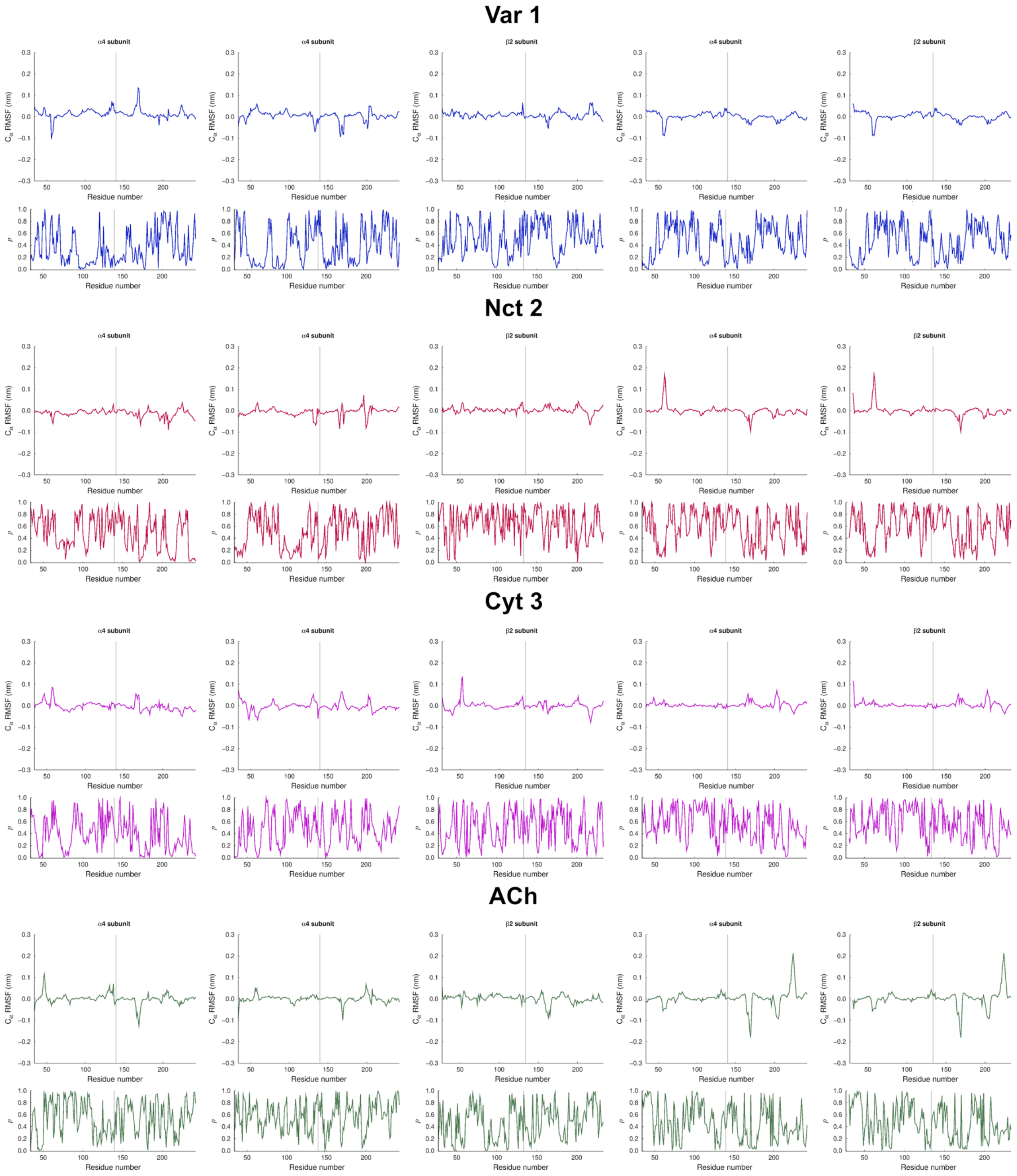

Figure S10- Average Cα RMSF difference between wild-type and mutant systems (**panels A-E**) and associated *p* values. A Student's t-test was used to compare the wild-type and mutant systems and to assess the significance of the differences. Positive values in the Cα RMSF difference plots correspond to a greater flexibility of the wild type during the simulations, whereas negative values correspond to an increased flexibility of the mutant. The vertical grey lines highlight the position of the mutations. Please zoom in on the image for detailed visualization.

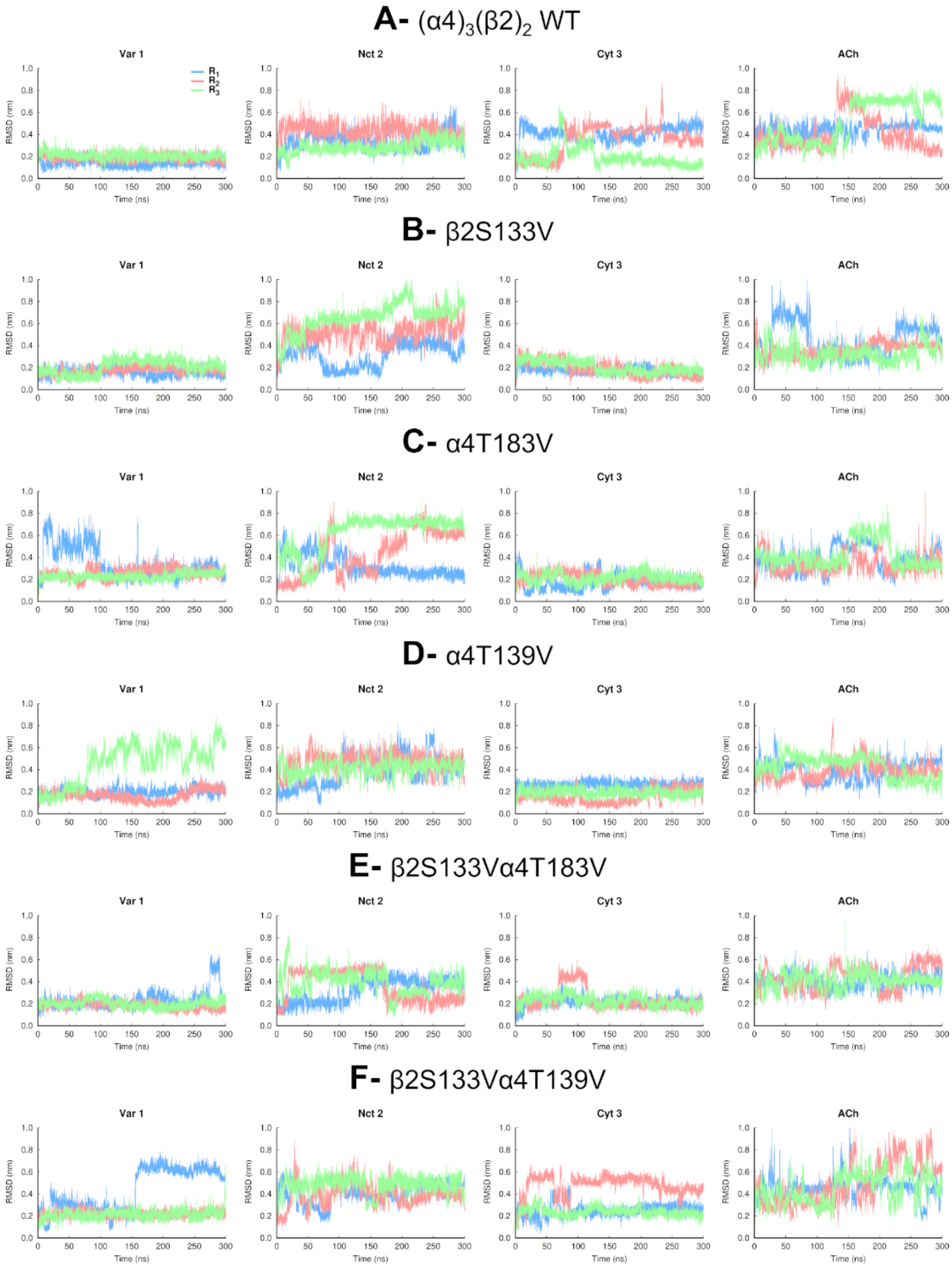

Figure S11- Temporal evolution of the RMSD for the agonists bound to the α-α binding pocket in the LS isoform of the α4β2 wild-type (**A**) and mutant (**B**-**F**) systems. The RMSD was determined with respect to the initial binding mode of the agonists at the start of the simulations. Note that the α-α binding pocket only exists in the LS isoform of the receptor. This analysis shows that despite the changes in binding mode observed for some ligands, all agonists remained stably bound to their respective binding sites. Please zoom in on the image for detailed visualization.

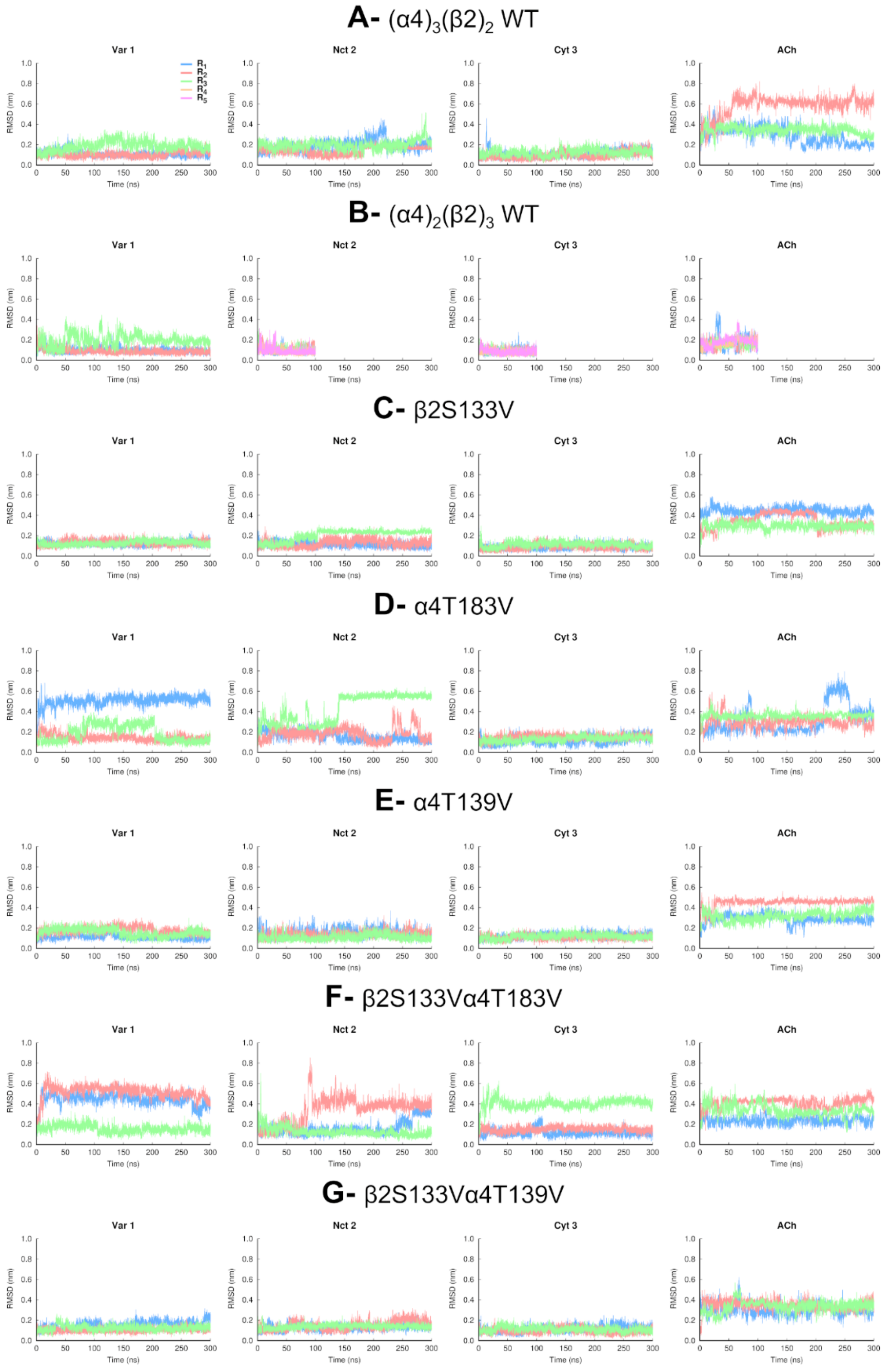

Figure S12- Temporal evolution of the RMSD for the agonists bound to the first α-β pocket in the LS (**A**) and HS (**B**) isoforms of the α4β2 wild-type and mutant (**C**-**G**) systems. The RMSD was determined with respect to the initial binding mode of the agonists at the start of the simulations. Note that despite the changes in binding mode observed for some ligands, all agonists remained stably bound to their respective binding sites. Please zoom in on the image for detailed visualization.

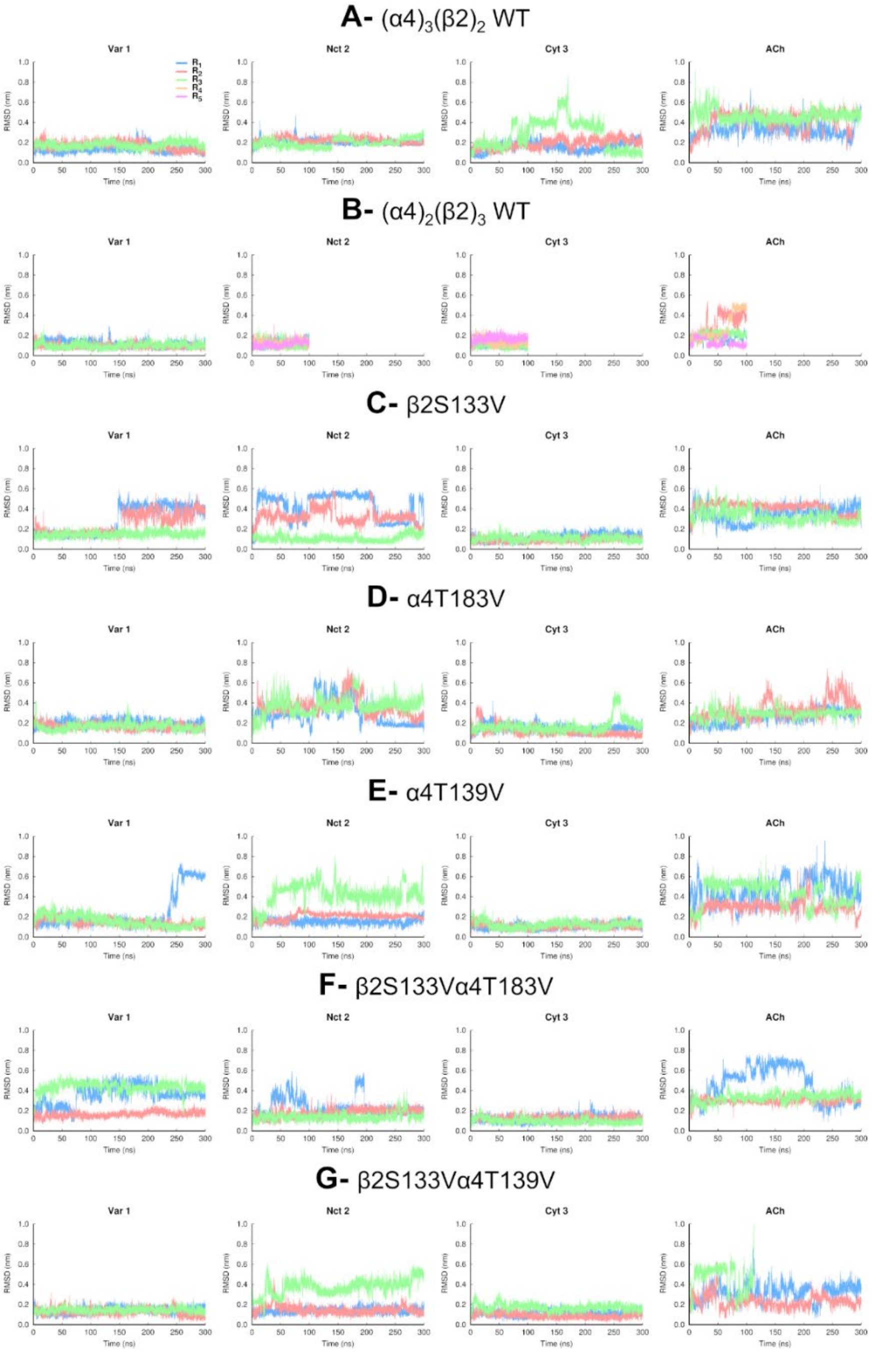

Figure S13- Temporal evolution of the RMSD for the agonists bound to the second α-β pocket in the LS (**A**) and HS (**B**) forms of the α4β2 wild-type and mutant (**C**-**G**) systems. The RMSD was determined with respect to the initial binding mode of the agonists at the start of the simulations. Note that all agonists remained stably bound to their respective binding sites, except for the ACh molecule in the second α-β pocket for replicate 3 in the β2S133Vα4T139V-ACh complex, which exited the pocket after 112 ns (as indicated by the green line in the rightmost plot of panel G). Please zoom in on the image for detailed visualization.

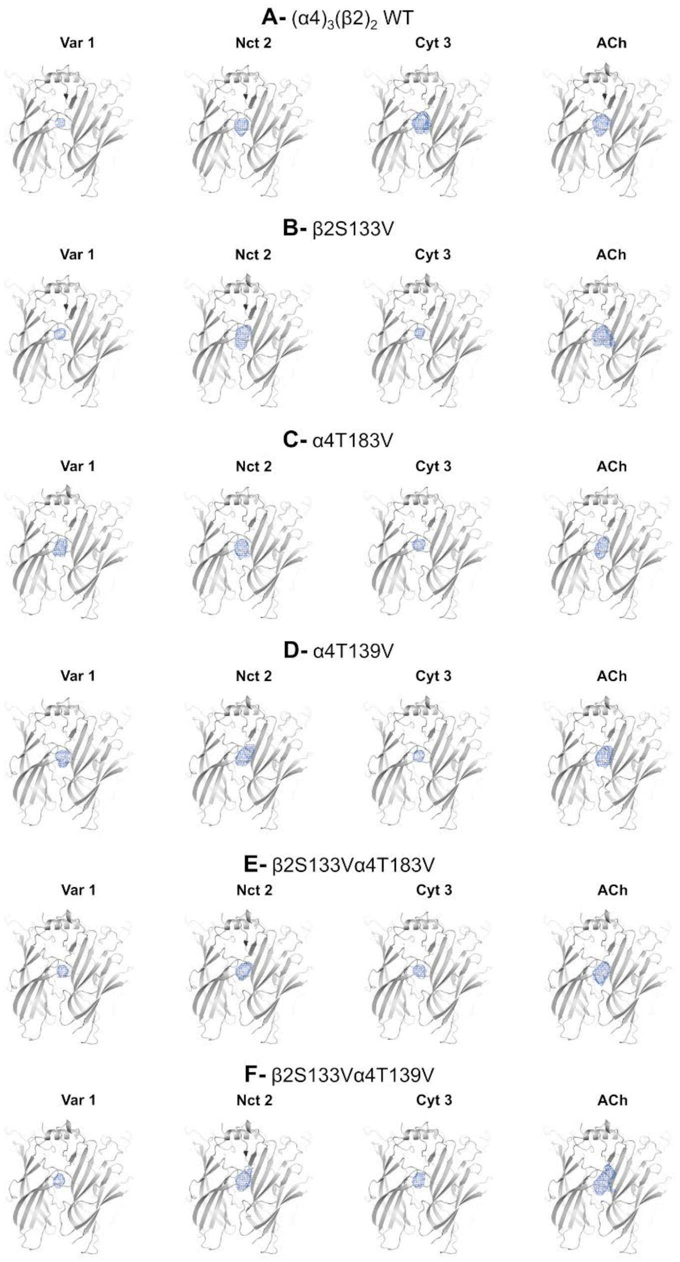

Figure S14- Probability density maps for the agonists bound to the α-α binding pocket in the wild-type (**A**) and mutant (**B-F**) LS isoform simulations. The contours at 0.00001 Å^{-3} for the protonated nitrogen atoms of the agonist are depicted as a blue mesh. For all systems, the maps were calculated by combining the entire trajectories for each one of the three replicates of that system. Please zoom in on the image for detailed visualization.

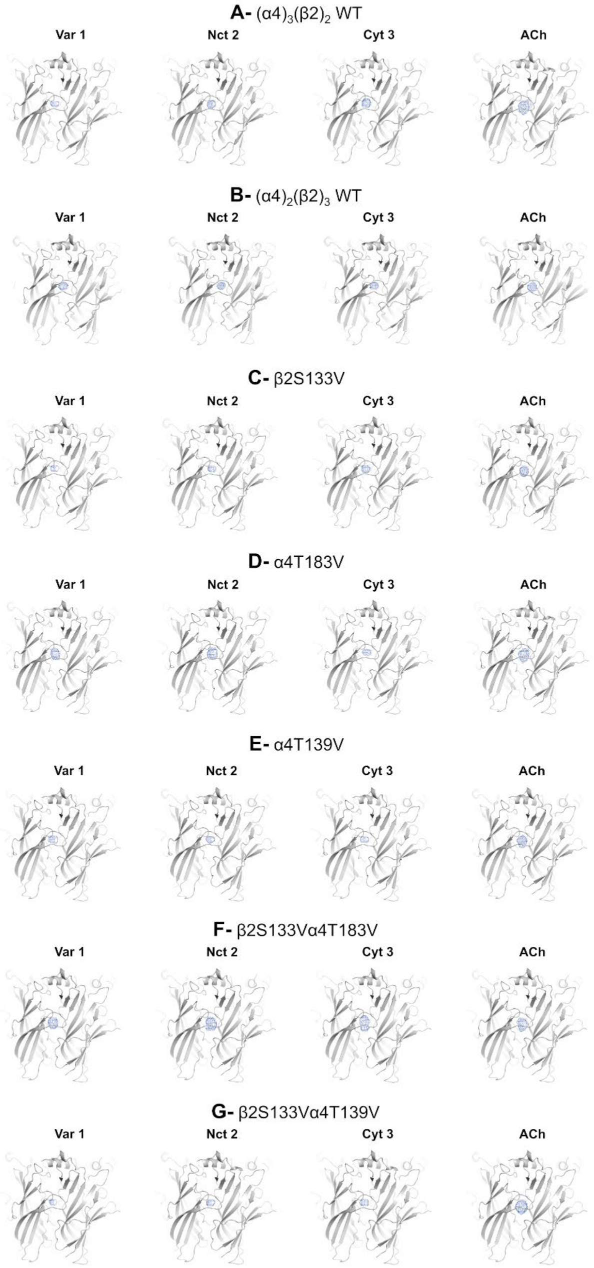

Figure S15- Probability density maps for the agonists bound to the first α-β binding pocket in the LS and HS isoforms of the α4β2 wild-type (**A-B**) and mutant (**C-G**) simulations. The contours at 0.00001 $Å^{-3}$ for the protonated nitrogen atoms of the agonist are depicted as a blue mesh. For all systems, the maps were calculated by combining the entire trajectories for each one of the three replicates of that system. Please zoom in on the image for detailed visualization.

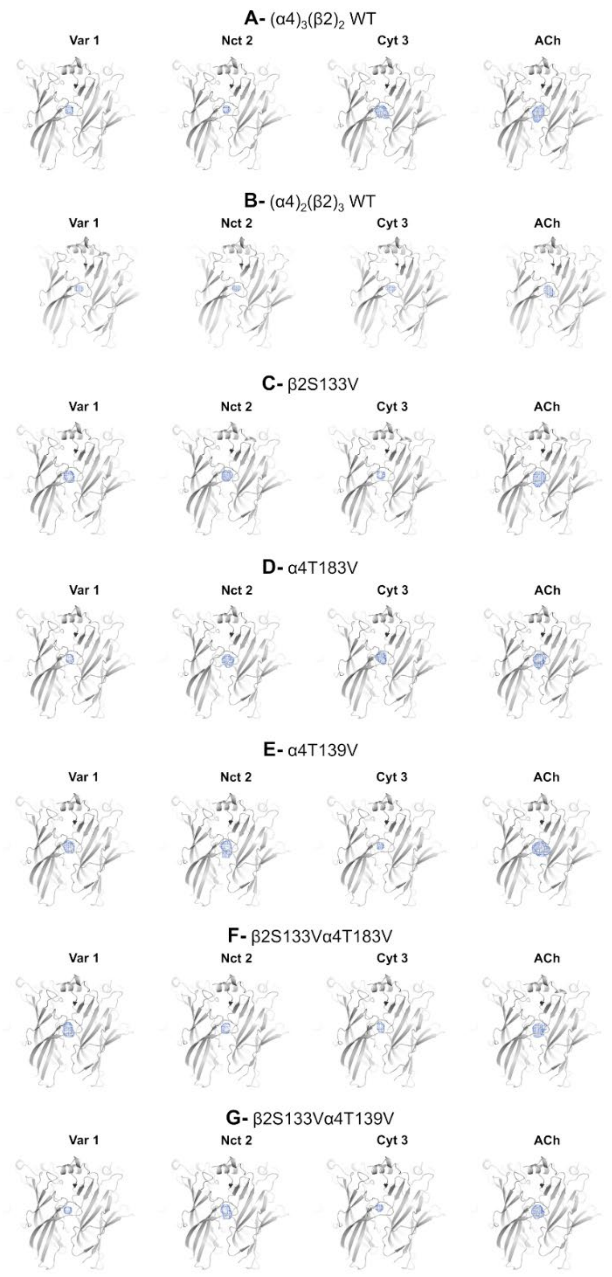

Figure S16- Probability density maps for the agonists bound to the second α-β binding pocket in the LS and HS isoforms of the α4β2 wild-type (**A-B**) and mutant (**C-G**) simulations. The contours at 0.00001 $Å^{-3}$ for the protonated nitrogen atoms of the agonist are depicted as a blue mesh. For all systems, with the exception of β2S133Vα4T139V-ACh, the maps were calculated by combining the entire trajectories for each one of the three replicates of that system. For the β2S133Vα4T139V-ACh complex, the map was obtained using the entire trajectory for replicates 1 and 2 and the first 112 ns for replicate 3 (as ACh exits the second α-β binding pocket after 112 ns of simulation). Please zoom in on the image for detailed visualization.

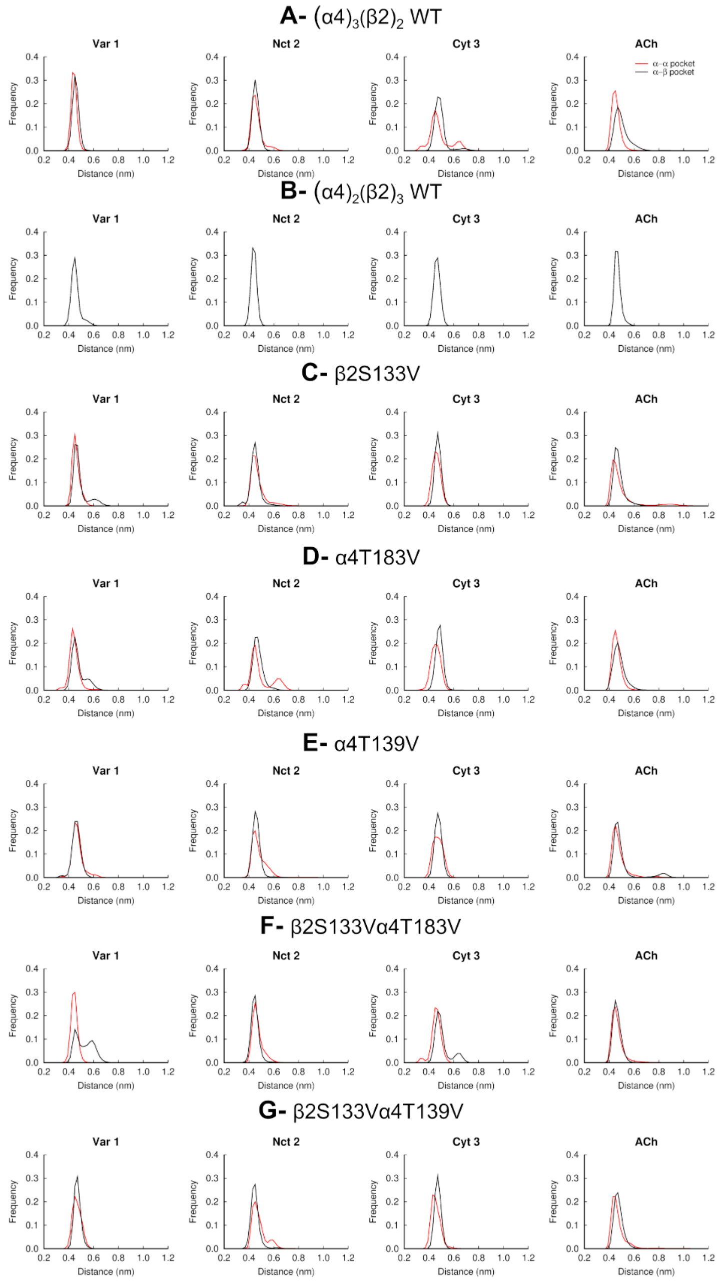

Figure S17- TrpB-agonist distance for the LS (**A**) and HS (**B**) isoforms of the α4β2 wild-type and mutant (**C-G**) systems. The distance between the side-chain of TrpB (W182 in the principal α4 subunit) and the protonated nitrogen atom of varenicline **1**, nicotine **2**, cytisine **3** and ACh for the α-α (red line) and α-β (black line) binding pockets is shown. The histogram for the α-β pocket reflects the distances over the two α-β binding pockets present in both the LS and HS isoforms of the α4β2 nAChR. Note that for the β2S133Vα4T139V–ACh complex, the histogram excludes the data for the second α–β pocket in replicate 3 from 112 ns onward, as the agonist exits the binding pocket during the simulation.

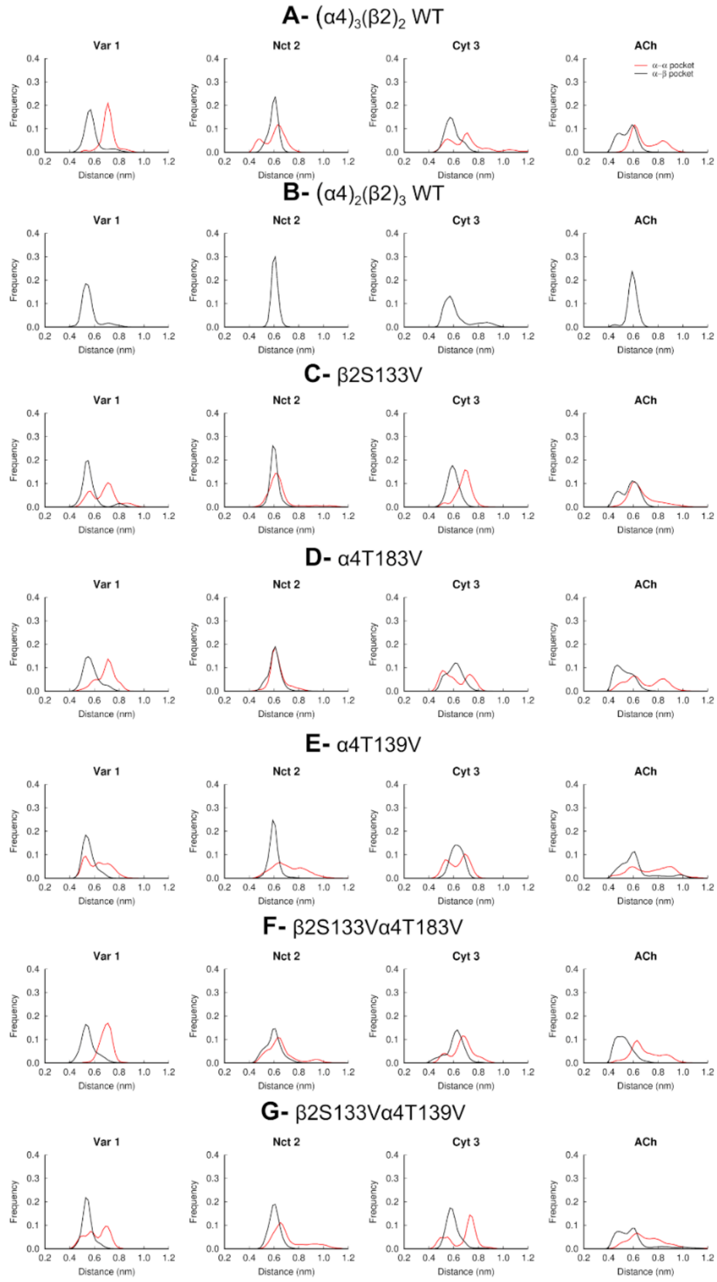

Figure S18- TyrA-agonist distance for the LS (**A**) and HS (**B**) isoforms of the α4β2 wild-type and mutant (**C-G**) systems. The distance between the side-chain of TyrA (Y126 in the principal α4 subunit) and the protonated nitrogen atom of varenicline **1**, nicotine **2**, cytisine **3** and ACh for the α-α (red line) and α-β (black line) binding pockets is shown. The histogram for the α-β pocket reflects the distances over the two α-β binding pockets present in both the LS and HS isoforms of the α4β2 nAChR. Note that for the β2S133Vα4T139V–ACh complex, the histogram excludes the data for the second α–β pocket in replicate 3 from 112 ns onward, as the agonist exits the binding pocket during the simulation.

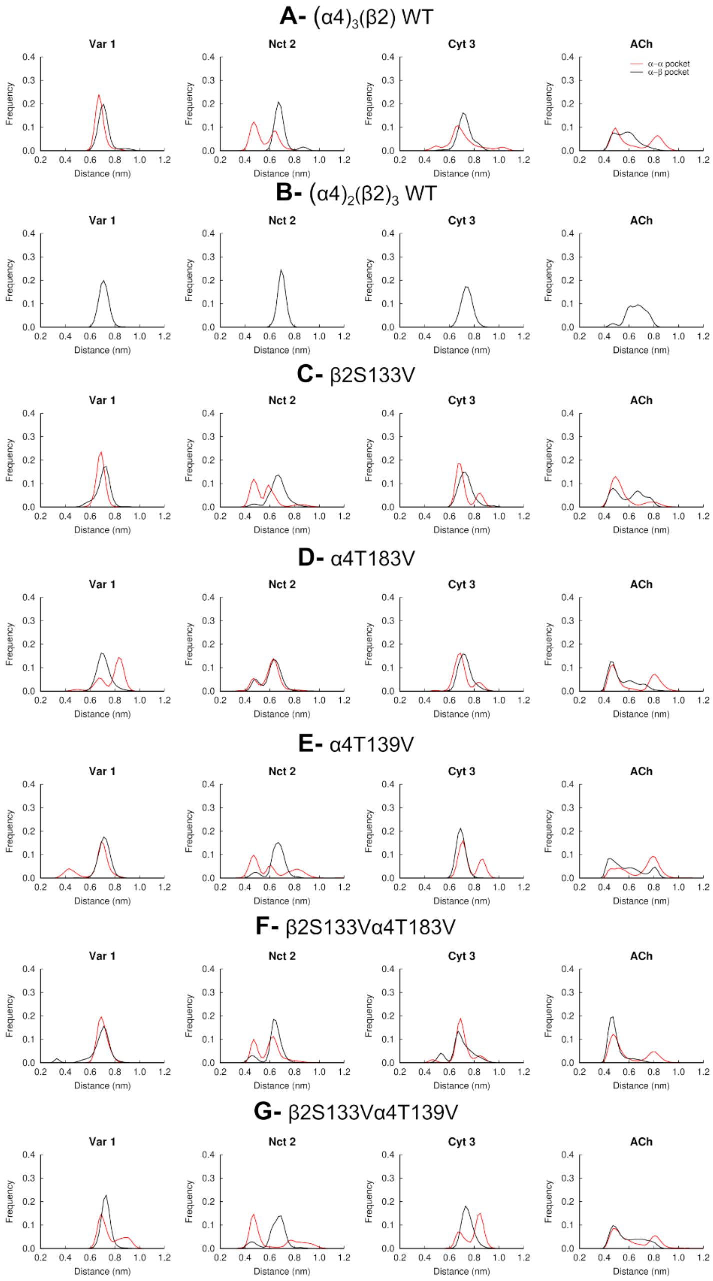

Figure S19- TrpD-agonist distance for the LS (**A**) and HS (**B**) isoforms of the α4β2 wild-type and mutant (**C-G**) systems. The distance between the side-chain of TrpD (W88 in the complementary α4 subunit of the α-α binding pocket and W82 in the complementary β2 subunit of the α-β binding pocket) and the protonated nitrogen atom of varenicline **1**, nicotine **2**, cytisine **3** and ACh for the α-α (red line) and α-β (black line) binding pockets. The histogram for the α-β pocket reflects the distances over the two α-β binding pockets present in both the LS and HS isoforms of the α4β2 nAChR. Note that for the β2S133Vα4T139V–ACh complex, the histogram excludes the data for the second α–β pocket in replicate 3 from 112 ns onward, as the agonist exits the binding pocket during the simulation.

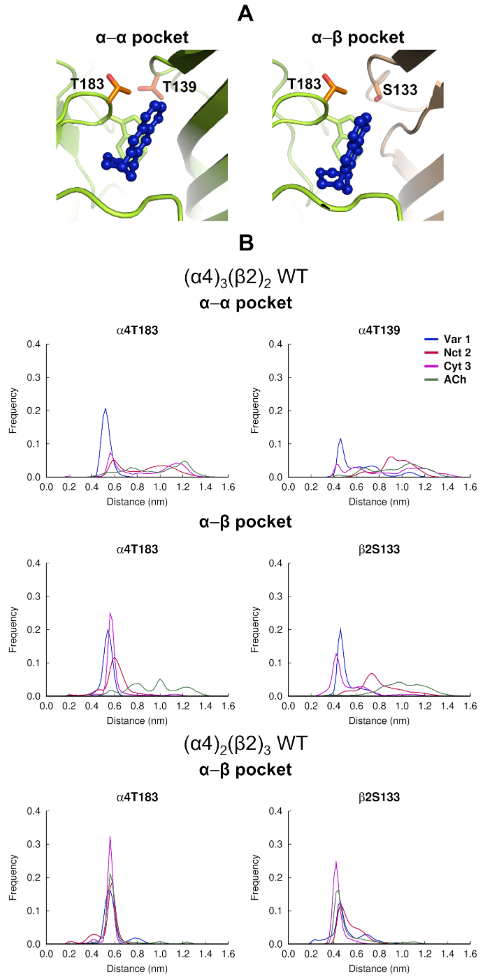

Figure S20- Agonist interactions with α4T183, α4T139 and β2S133 in the LS and HS isoforms of the α4β2 wild-type systems. (**A**) Location of α4T183 and α4T139 in the α-α pocket (left panel) and α4T183 and β2S133 in the α-β (right panel) binding site. The α4 and β2 subunits are colored in yellow and light brown, respectively. Varenicline **1** is highlighted in dark blue. The side-chains of α4T183, α4T139 and β2S133 are represented with orange sticks, whereas TrpB is shown with yellow sticks. Note that α4T183 is located in the principal α4 face of the pockets, whereas α4T139 and β2S133 are in the complementary face of the α-α and α-β pockets, respectively. (**B**) Distribution of the minimum distance between the agonist and α4T183, α4T139 and β2S133 in the α-α and α-β binding pockets of the LS and HS receptors. The reported values correspond to the minimum distances between the agonist (specifically, the closest pyrazine nitrogen in the quinoxaline moiety of varenicline **1**, the pyridine nitrogen of nicotine **2,** the pyridone carbonyl oxygen of cytisine **3** and the closest oxygen in the ester group of ACh), and the hydroxyl group of α4T183, α4T139 and β2S133 in all the MD trajectories for each complex. The histogram for the α-β pocket reflects the distances over the two α-β binding pockets present in the LS and HS isoforms of the α4β2 nAChR. These distance profiles indicate that some agonists can closely approach the H-bond donor groups in the side-chain of α4T183, α4T139, and β2S133, thus suggesting the possibility of transient interactions occurring between them.

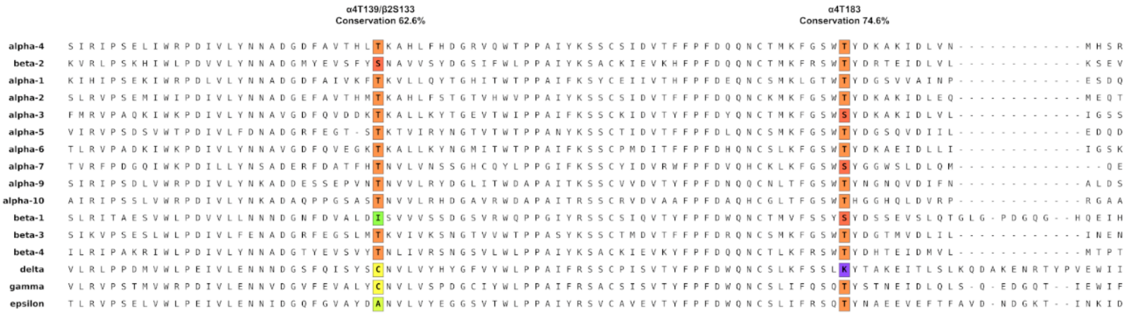

Figure S21- Sequence alignment for the human α1-α7, α9-α10, β1-β4, δ, γ, and ε nAChR subunits. The sequence alignments were performed using the Muscle server.[30] The colored boxes highlight the locations of α4T183, α4T139 and β2S133, with threonine, serine, isoleucine, cysteine, alanine, and lysine residues represented by orange, red, green, yellow, light green, and purple, respectively. Conservation percentages are expressed as $100\times(1-H/H_{max})$, where H is the Shannon entropy of the residue at the alignment position.[31] The sequences shown correspond to the following UniProt codes: P43681 (human α4), P17787 (human β2), P02708 (human α1), Q15822 (human α2), P32297 (human α3), P30532 (human α5), Q15825 (human α6), P36544 (human α7), Q9U6MI (human α9), Q9GZZ6 (human α10), P11230 (human β1), Q05901 (human β3), Q07001 (human δ), P07510 (human γ), and Q04844 (human ε).

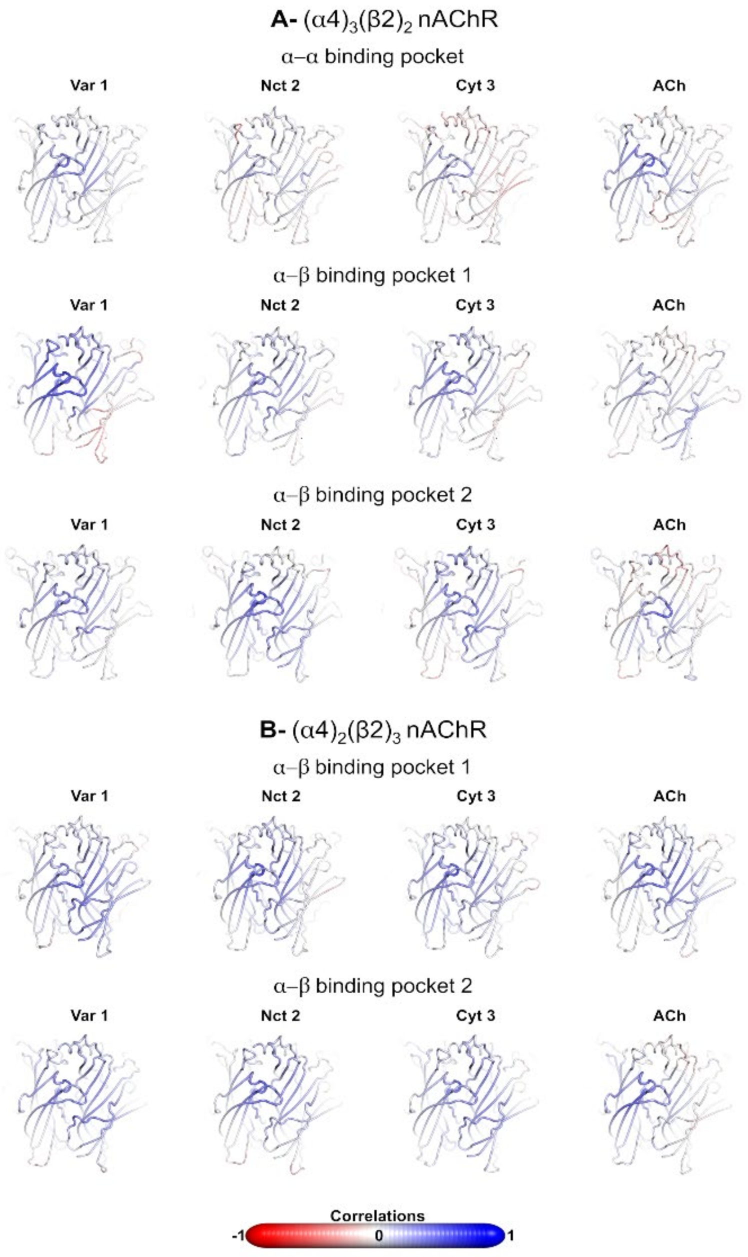

Figure S22- Statistical correlations for the different agonists when bound to ECD of the (**A**) LS and (**B**) HS isoforms of the α4β2 nAChR. Correlated motions for the agonist in the α-α and α-β binding pockets of the wild-type system. The correlations between the protonated nitrogen atom of the agonists and all the Cα atoms in the receptor are shown. Note that the atoms that systematically move in opposite directions have a correlation value of -1, whereas those systematically moving along the same direction show a correlation of 1. The atoms whose movements relative to the agonist are uncorrelated present a correlation value of 0. Please zoom in on the image for detailed visualization.

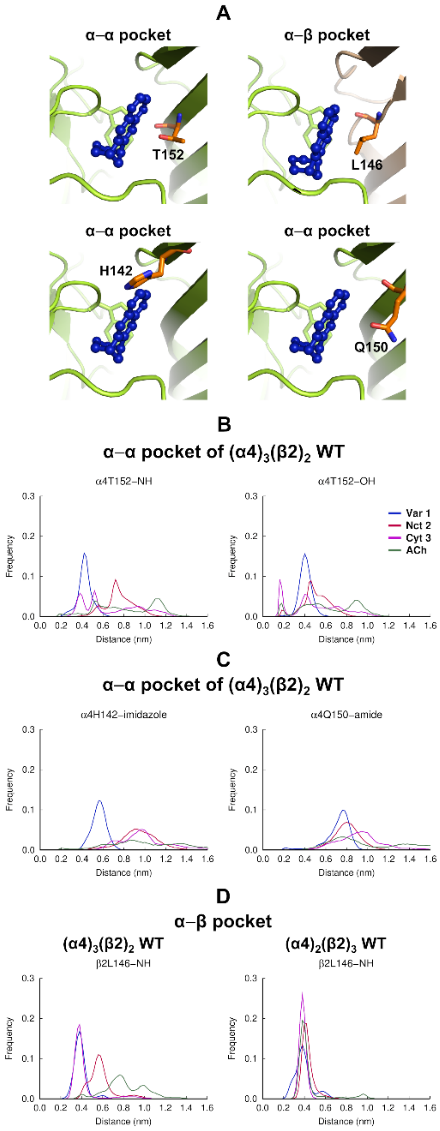

Figure S23- Interactions between the agonists and α4H142, α4Q150, α4T152, and β2L146 in the LS and HS isoforms of the α4β2 wild-type systems. (**A**) Location of α4H142, α4Q150 and α4T152 in the α-α pocket and β2L146 in the α-β pocket. The α4 and β2 subunits are colored in yellow and light brown, respectively. Varenicline **1** is highlighted in dark blue. α4H142, α4Q150, α4T152 and β2L146 are represented with orange sticks while TrpB is shown with yellow sticks. Note that α4H142, α4Q150 and α4T152 are located in the complementary face of the α-α pocket, whereas β2L146 is situated in the complementary side of the α-β pockets. (**B**) Distribution of the minimum distance between the agonist and the backbone NH and side-chain hydroxyl group of α4T152 in the α-α pocket of the LS isoform. The values reported correspond to the minimum distance between the agonist (namely, the closest pyrazine nitrogen in the quinoxaline group of varenicline **1**, the pyridine nitrogen of nicotine **2**, the pyridone carbonyl oxygen of cytisine **3**, and the closest oxygen in the ester group of ACh) and the NH

and OH group of α4T152 in all the MD trajectories for each complex. (**C**) Distribution of the minimum distance between the agonist and the H-bond donors in the side-chain of α4H142 and α4Q150 in the α-α pocket of the LS isoform. The values reported correspond to the minimum distance between the agonist (namely, the closest pyrazine nitrogen in the quinoxaline group of varenicline **1**, the pyridine nitrogen of nicotine **2**, the pyridone carbonyl oxygen of cytisine **3**, and the closest oxygen in the ester group of ACh) and the imidazole NH and amide NH2 group in the side-chain of α4H142 and α4Q150 in all the MD trajectories for each complex. (**D**) Distribution of the minimum distance between the agonist and the backbone NH of β2L146 in the α-β pockets of the LS (left panel) and HS (right panel) isoforms of the α4β2 receptor. The values reported correspond to the minimum distance between the agonist (namely, the closest pyrazine nitrogen in the quinoxaline group of varenicline **1**, the pyridine nitrogen of nicotine **2**, the pyridone carbonyl oxygen of cytisine **3**, and the closest oxygen in the ester group of ACh) and the NH group of β2L146 in all the MD trajectories for each complex. The histograms reflect the distances over the two α-β binding pockets present in the LS and HS isoforms of the α4β2 nAChR. Note that the distance profiles above indicate that some agonists can directly interact with the α4T152 hydroxyl donor in the α-α pocket.

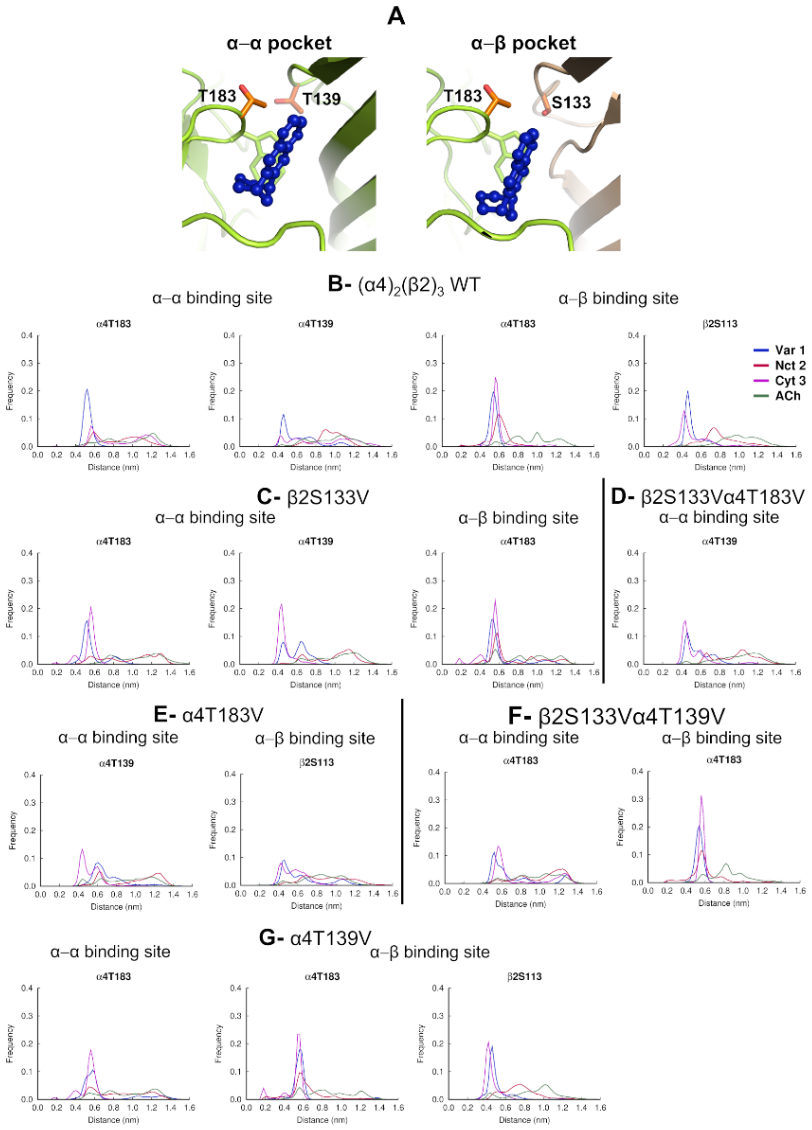

Figure S24- Agonist interactions with α4T183, α4T139 and β2S133 in the LS isoform of the wild-type and mutant systems. (**A**) Location of α4T183, α4T139 and β2S133 in the α-α (left panel) and α-β (right panel) binding pockets of the LS wild-type receptor. The α4 and β2 subunits are colored in yellow and light brown, respectively. Varenicline **1** is highlighted in dark blue. The side-chains of α4T183, α4T139 and β2S133 are represented with orange sticks, whereas TrpB is shown with yellow sticks. (**B-G**) Distribution of the minimum distance between the agonist and α4T183, α4T139 and β2S133 in the α-α and α-β binding pockets of the wild-type and β2S133V, α4T183V, α4T139V, β2S133Vα4T183V and β2S133Vα4T139V mutants. The reported values correspond to the minimum distances between the agonist (specifically, the closest pyrazine nitrogen in the quinoxaline group of varenicline **1**, the pyridine nitrogen of nicotine **2**, the pyridone carbonyl oxygen of cytisine **3**, and the closest oxygen in the ester group of ACh) and the hydroxyl group of α4T183, α4T139 and β2S133 in the MD trajectories for each complex. The histogram for the α-β pocket reflects the distances over the two α-β binding pockets present in the LS isoform of the α4β2 nAChR. Note that the substitution of α4T183, α4T139,

and β2S133 by valine (which lacks the hydroxyl group in its side-chain) prevents potential hydrogen bonding with the agonists. These distance profiles indicate that, as anticipated, the mutations altered the interaction patterns between the H-bond donors in the receptor binding sites and the agonists' acceptor groups.

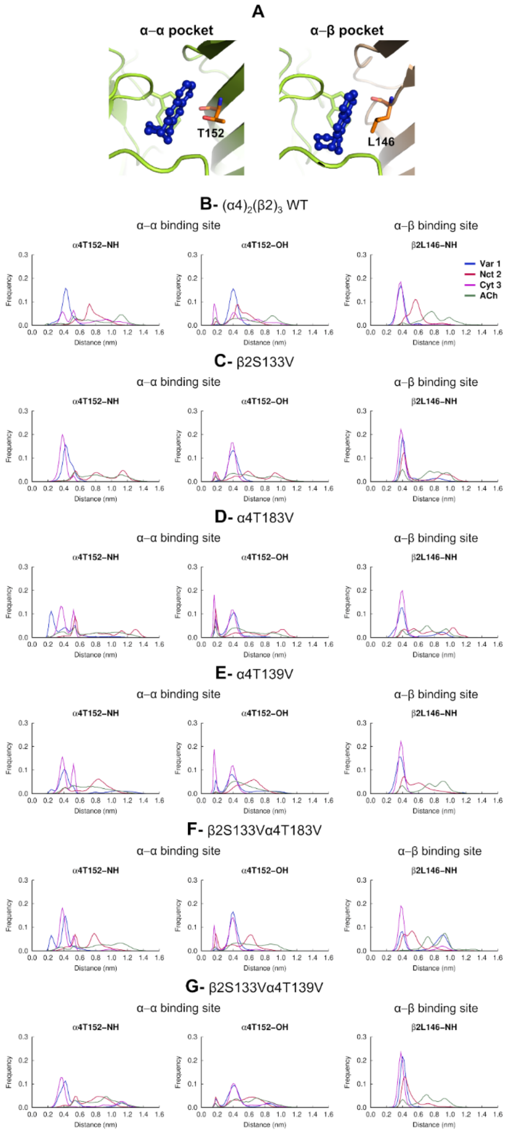

Figure S25- Agonist interactions with α4T152 and β2L146 in the LS isoform of the wild-type and mutant systems. (**A**) Location of α4T152 and β2L146 in the α-α (left panel) and α-β (right panel) binding pockets of the LS wild-type receptor. The α4 and β2 subunits are colored in yellow and light brown, respectively. Varenicline **1** is highlighted in dark blue. The side-chains of α4T152 and β2L146 are represented with orange sticks, whereas TrpB is shown with yellow sticks. (**B-G**) Distribution of the minimum distance between the agonist and α4T152 and β2L146 in the α-α and α-β binding pockets of the wild-type (**B**) and β2S133V (**C**), α4T183V (**D**), α4T139V (**E**), β2S133Vα4T183V (**F**) and β2S133Vα4T139V (**G**) mutants. The reported values correspond to the minimum distances between the agonist (specifically, the

closest pyrazine nitrogen in the quinoxaline group of varenicline **1**, the pyridine nitrogen of nicotine **2**, the pyridone carbonyl oxygen of cytisine **3**, and the closest oxygen in the ester group of ACh) and the backbone NH of α4T152 and β2L146 and the side-chain OH of α4T152 in the MD trajectories for each complex. The histogram for the α-β pocket reflects the distances over the two α-β binding pockets present in the LS isoform of the α4β2 nAChR. Overall, these distance profiles indicate that the mutations mainly altered the interaction pattern between the agonists and α4T152 within the α-α pocket.

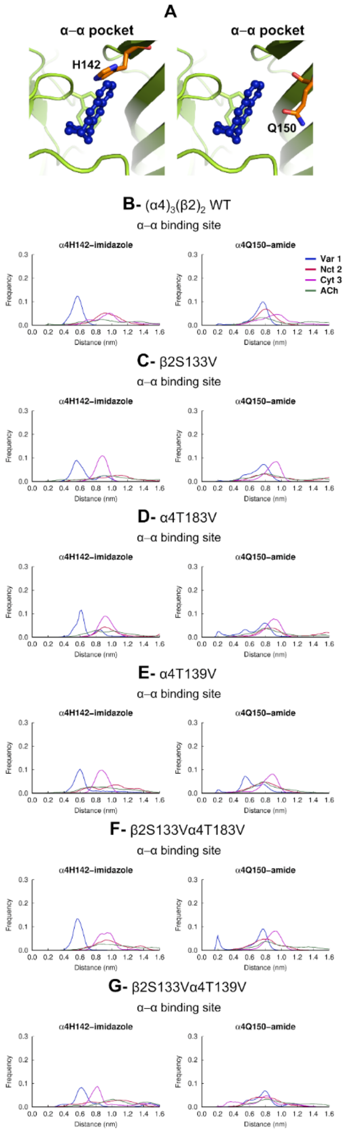

Figure S26- Agonist interactions with α4H142 and α4Q150 in the LS isoform of the wild-type and mutant systems. (**A**) Location of α4H142 (left panel) and α4Q150 (right panel) in the α-α binding pocket of the LS wild-type receptor. The α4 and β2 subunits are colored in yellow and light brown, respectively. Varenicline **1** is highlighted in dark blue. The side-chains of α4H142 and α4Q150 are represented with orange sticks, whereas TrpB is shown with yellow sticks. (**B-G**) Distribution of the minimum distance

between the agonists and α4H142 and α4Q150 in the α-α binding pocket of the wild-type (**B**) and β2S133V (**C**), α4T183V (**D**), α4T139V (**E**), β2S133Vα4T183V (**F**) and β2S133Vα4T139V (**G**) mutants. The reported values correspond to the minimum distances between the agonist (specifically, the closest pyrazine nitrogen in the quinoxaline group of varenicline **1**, the pyridine nitrogen of nicotine **2**, the pyridone carbonyl oxygen of cytisine **3**, and the closest oxygen in the ester group of ACh) and the NH in the imidazole side-chain of α4H142 and the side-chain NH2 amide of α4Q150 in the MD trajectories for each complex. Overall, these distance profiles indicate that the mutations mainly altered the interaction patterns between the agonists and α4Q150 within the α-α pocket.

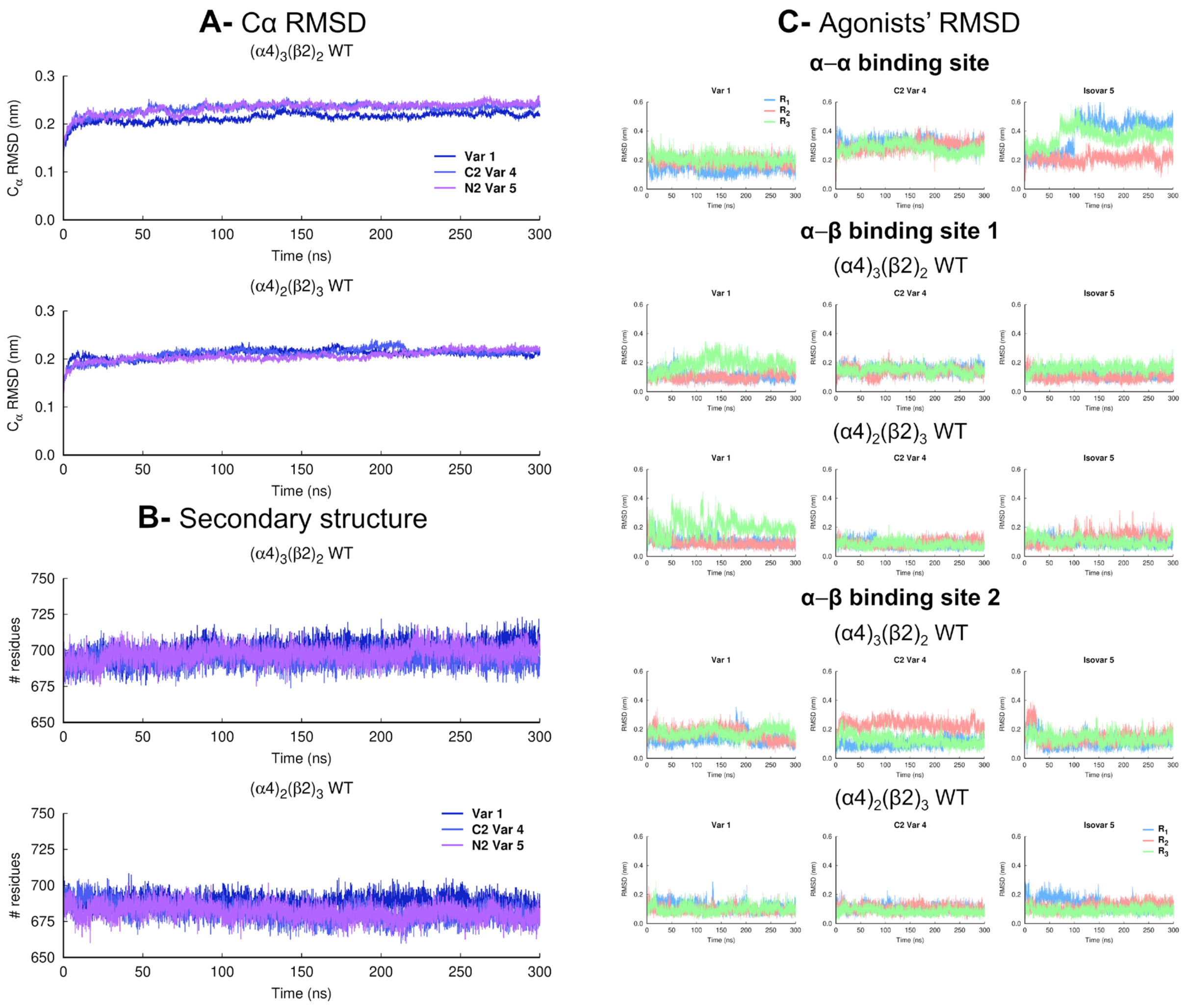

Figure S27- (**A**) Temporal evolution of the average Cα RMSD for the varenicline **1,** C_2 varenicline **4** and isovarenicline **5** bound to the LS and HS isoforms of the α4β2 wild-type systems. The Cα RMSD was calculated relative to the starting structures, and the averages were obtained over all replicates for each system. (**B**) Time evolution of number of residues involved in secondary structure motifs for the varenicline **1**, C_2 varenicline **4** and isovarenicline **5** bound systems. The secondary structure assignment was performed with the DSSP software[22] and includes all residues assigned to α-helix, π-helix, 3_{10}-helix, 5-helix, β-sheet, β-strand and β-bridge secondary structure classes. The averages were obtained over all replicates for each system. (**C**) Time evolution of the RMSD for varenicline **1,** C_2 varenicline **4** and isovarenicline **5** when bound to the α-α and α-α binding pockets in the LS and HS isoforms of the wild-type system. The RMSD was determined with respect to the initial binding mode of the agonists at the start of the simulations. Note that the α-α binding pocket only exists in the LS isoform of the receptor. Please zoom in on the image for detailed visualization.

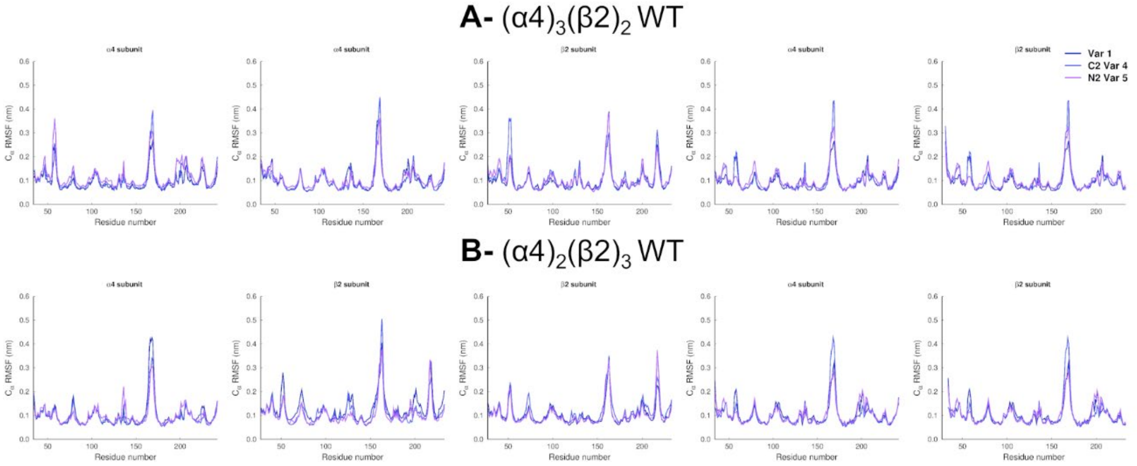

Figure S28- Average Cα RMSF for the LS (**A**) and HS (**B)** isoforms of the wild-type receptors with varenicline **1**, C_2 varenicline **4** and isovarenicline **5** bound. The Cα RMSF was calculated using the entire trajectories and averaged across all replicates for each complex. Please zoom in on the image for detailed visualization.

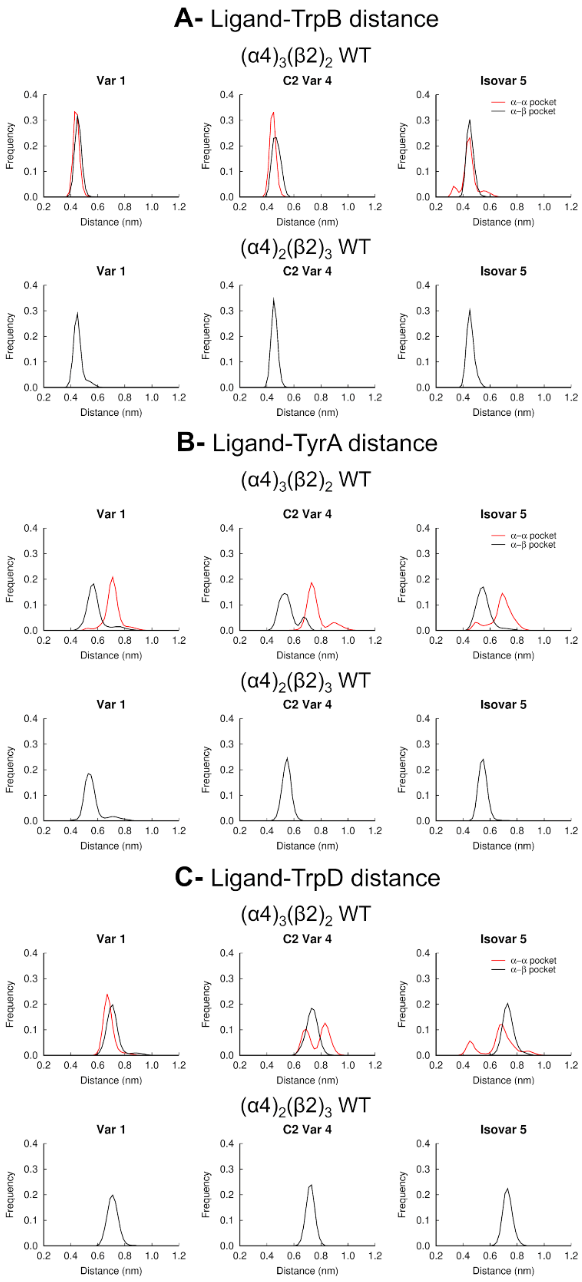

Figure S29- Distance profiles between varenicline **1**, C_2 varenicline **4** and isovarenicline **5** and TrpB **(A)**, TyrA (**B)** and TrpD (**C**) for the LS and HS isoforms of the α4β2 wild-type systems. Distance between the side chain of TrpB (W182 in the principal α4 subunit), TyrA (Y126 in the principal α4 subunit) and TrpD (W88 in the complementary α4 subunit of the α-α binding pocket and W82 in the complementary β2 subunit of the α-β binding pocket) and the protonated (piperidine) nitrogen atom of varenicline**1**, C_2 varenicline **4** and isovarenicline **5** for the α-α (red line) and α-β (black line) binding pockets is shown. The histogram for the α-β pocket reflects the distances over the two α-β binding pockets present in the LS and HS nAChRs.

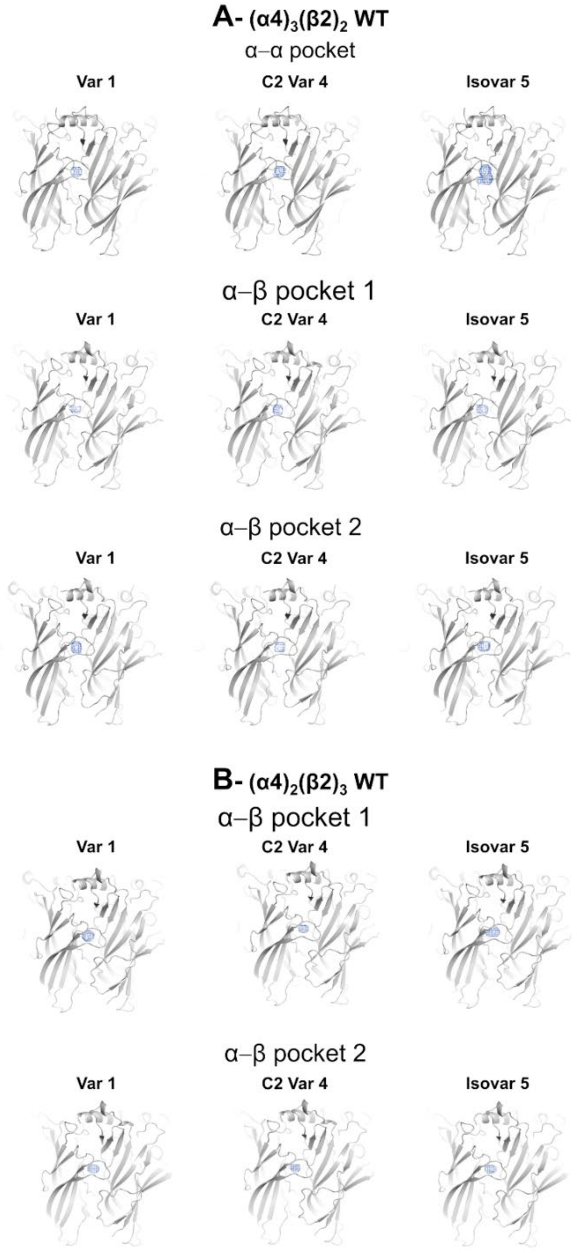

Figure S30- Probability density maps for varenicline **1**, C_2 varenicline **4** and isovarenicline **5** bound to the α-α and α-β binding pocket in the LS (**A**) and HS (**B**) isoforms of the α4β2 wild-type simulations. The contours at 0.00001 $Å^{-3}$ for the protonated nitrogen atom of the agonists are depicted as a blue mesh. The maps were calculated by combining the entire trajectories (0-300 ns) for each one of the three replicates of that system. Please zoom in on the image for detailed visualization.

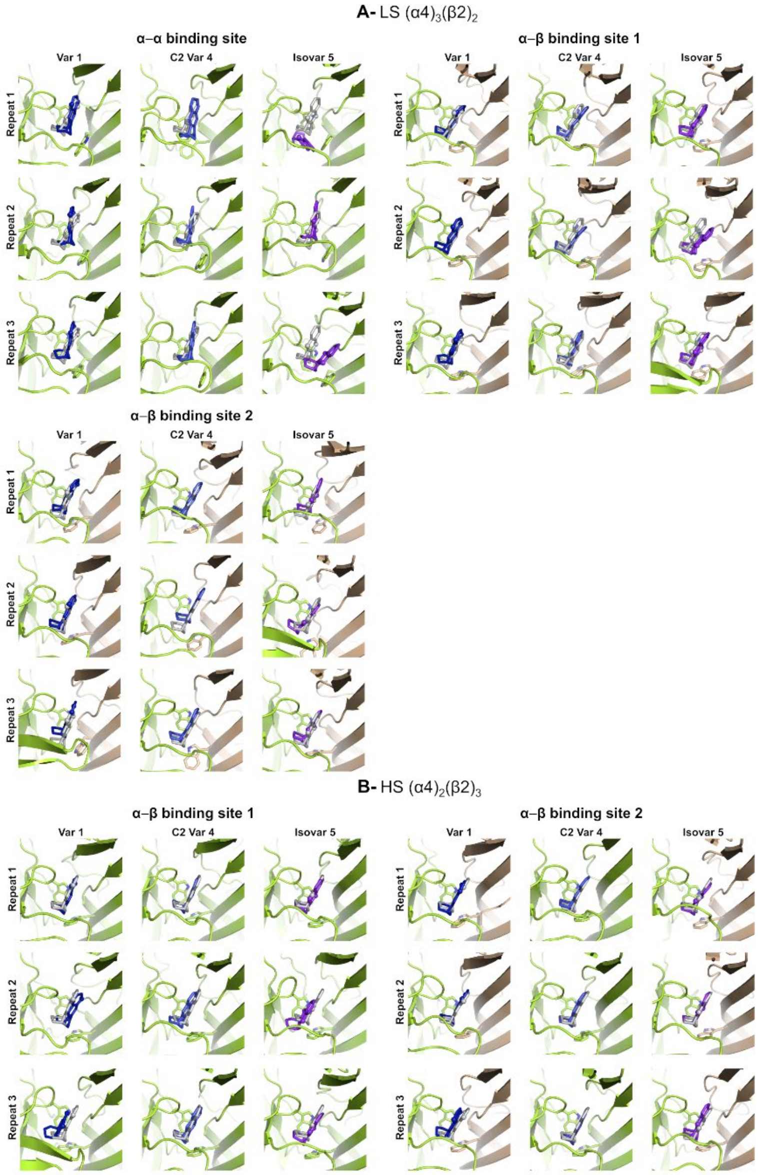

Figure S31- Binding mode of varenicline **1,** C_2 varenicline **4**, and isovarenicline **5** after 300 ns of simulation for the wild-type α4β2 complexes. (**A**) Binding mode of varenicline **1**, C_2 varenicline **4** and isovarenicline **5** in the α-α and α-β pockets of the wild-type LS nAChR. (**B**) Binding mode of varenicline **1**, C_2 varenicline **4** and isovarenicline **5** in the α-β pockets of the wild-type HS nAChR. The α4 and β2 subunits are colored in yellow and light brown, respectively. Agonists are depicted in blue, with the nitrogen atoms of the quinoxaline moiety (which serve as H-bond acceptors) highlighted by spheres. Please note that C_2 varenicline **4** contains a naphthalene residue instead of a quinoxaline unit, and thus lacks the nitrogen atoms needed to form hydrogen bonds. The grey sticks represent the starting binding mode for the agonists. TrpB and TrpD are shown with sticks. Please zoom in on the image for detailed visualization.

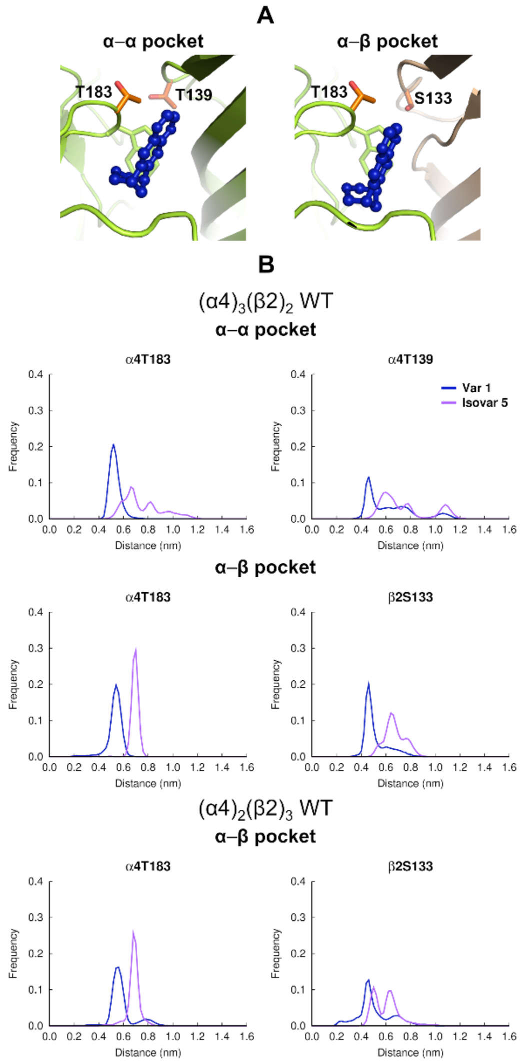

Figure S32- Varenicline **1** and isovarenicline **5** interactions with α4T183, α4T139 and β2S133 in the LS and HS isoforms of the α4β2 wild-type systems. (**A**) Location of α4T183, α4T139 and β2S133 in the α-α (left panel) and α-β (right panel) binding pockets. The α4 and β2 subunits are colored in yellow and light brown, respectively. Varenicline **1** is highlighted in dark blue. The side-chains of α4T183, α4T139 and β2S133 are represented with orange sticks, whereas TrpB is shown with yellow sticks. (**B**) Distribution of the minimum distance between the closest pyrazine nitrogen in the quinoxaline group of varenicline **1** and isovarenicline **5** and α4T183, α4T139 and β2S133 in the α-α and α-β binding pockets of the LS $(\alpha4)_3(\beta2)_2$ and HS $(\alpha4)_2(\beta2)_3$ receptor in all the MD trajectories for each complex. Note that C_2 varenicline **4**, instead of a quinoxaline unit, possesses a naphthalene group, and is therefore unable to form hydrogen bonds. The histogram for the α-β pocket reflects the distances over the two α-β binding pockets present in the LS and HS of the α4β2 nAChR. These distance profiles clearly demonstrate that, as expected, the distance between the hydrogen acceptor group in isovarenicline **5** and the α4T183/α4T139/β2S133 side-chain OH group is too large, thereby preventing any direct interaction between the two.

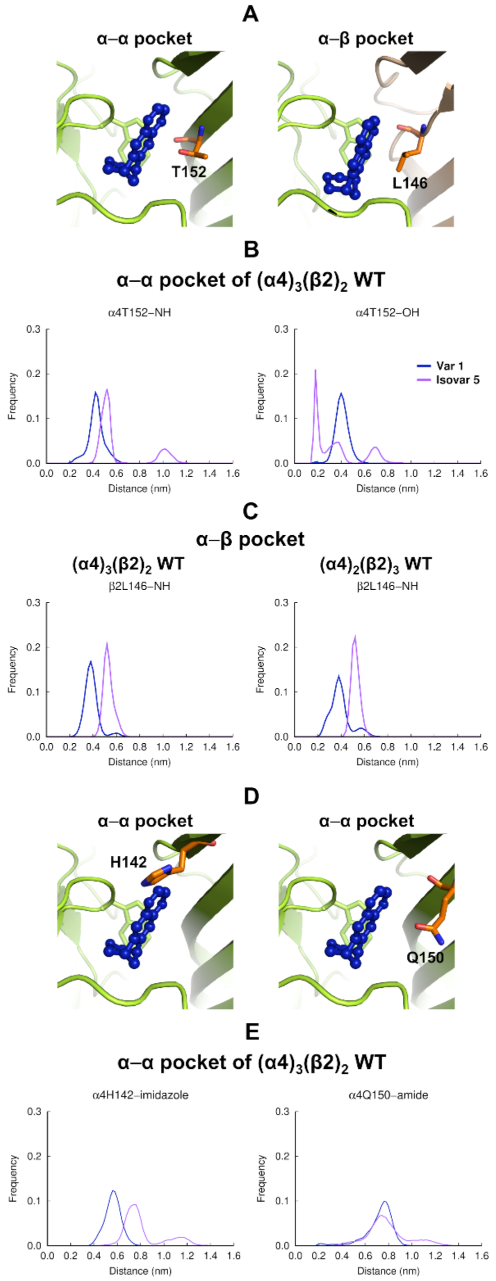

Figure S33- Varenicline **1** and isovarenicline **5** interactions with α4T152 and β2L146 in the LS and HS isoforms of the α4β2 wild-type systems. (**A**) Location of α4T152 and β2L146 in the α-α (left panel) and α-β (right panel) binding pockets of the $(\alpha4)_3(\beta2)_2$ wild-type receptor. See caption of Figure S32 for more details. (**B**) Distribution of the minimum distance between the closest pyrazine nitrogen in the quinoxaline group of varenicline **1** and isovarenicline **5** and the backbone NH and side-chain OH of α4T152 in the α-α pocket of the LS isoform of α4β2. Please note that **C_2** varenicline **4**, instead of a quinoxaline unit, possesses a naphthalene group, and is therefore unable to form hydrogen bonds. (**C**) Distribution of the minimum distance between the closest pyrazine nitrogen in the quinoxaline group of varenicline **1** and isovarenicline **5** and the backbone NH of β2L146 in the α-β pockets of the LS (left panel) and HS (right panel) forms of the receptor. The histograms reflect the distances over the two α-

β binding pockets present in the LS and HS isoforms of the α4β2 nAChR. Surprisingly, the distance profiles above show that isovarenicline **5** can make a persistent interaction with the α4T152 hydroxyl donor in the α-α pocket. (**D**) Location of α4H142 (left panel) and α4Q150 (right panel) in the α-α binding pockets of the LS wild-type receptor. See caption of Figure S32 for more details. (**E**) Distribution of the minimum distance between the closest pyrazine nitrogen in the quinoxaline group of varenicline **1** and isovarenicline **5** and the NH group in the imidazole side-chain of α4H142 (left side) and the NH2 in the amide side-chain of α4Q150 (right side) in the α-α pocket of the LS isoform. The distance profiles show that isovarenicline **5** is unable to directly H-bond to α4H142 and α4Q150.

Table S1- Summary of the simulations performed for the ECD of the human α4β2 nAChR. [a] The LS isoform corresponds to the $(\alpha4)_3(\beta2)_2$ receptor, whereas the HS to the $(\alpha4)_2(\beta2)_3$ one. [b] The simulations for the complexes between HS receptor with nicotine **2**, cytisine **3** and ACh were taken from our previous work.[7, 8]

Abbreviations used: varenicline **1** - **Var 1**; nicotine **2** - **Nct 2**; cytisine **3** - **Cyt 3**; acetylcholine - **ACh**; C_2 varenicline **4** - **C2 Var 4**; isovarenicline **5** - **Isovar 5**.

	Isoform[a]	System	Agonist	Simulation length (ns)	Number of replicates
1	LS	Wild type	**Var 1**	300	3
2	LS	Wild type	**Nct 2**	300	3
3	LS	Wild type	**Cyt 3**	300	3
4	LS	Wild type	**ACh**	300	3
5	LS	β2S133V	**Var 1**	300	3
6	LS	β2S133V	**Nct 2**	300	3
7	LS	β2S133V	**Cyt 3**	300	3
8	LS	β2S133V	**ACh**	300	3
9	LS	α4T183V	**Var 1**	300	3
10	LS	α4T183V	**Nct 2**	300	3
11	LS	α4T183V	**Cyt 3**	300	3
12	LS	α4T183V	**ACh**	300	3
13	LS	α4T139V	**Var 1**	300	3
14	LS	α4T139V	**Nct 2**	300	3
15	LS	α4T139V	**Cyt 3**	300	3
16	LS	α4T139V	**ACh**	300	3
17	LS	α4T183Vβ2S133V	**Var 1**	300	3
18	LS	α4T183Vβ2S133V	**Nct 2**	300	3
19	LS	α4T183Vβ2S133V	**Cyt 3**	300	3
20	LS	α4T183Vβ2S133V	**ACh**	300	3
21	LS	α4T139Vβ2S133V	**Var 1**	300	3
22	LS	α4T139Vβ2S133V	**Nct 2**	300	3
23	LS	α4T139Vβ2S133V	**Cyt 3**	300	3
24	LS	α4T139Vβ2S133V	**ACh**	300	3
25	HS	Wild type	**Var 1**	300	3
26[b]	HS	Wild type	**Nct 2**	100	5
27[b]	HS	Wild type	**Cyt 3**	100	5
28[b]	HS	Wild type	**ACh**	100	5
29	HS	Wild type	**C2 Var 4**	300	3
30	HS	Wild type	**Isovar 5**	300	3
31	HS	Wild type	**C2 Var 4**	300	3
32	HS	Wild type	**Isovar 5**	300	3

C. nAChR Ligand Binding Measurements

(i) Expression of human α4β2, α3β4 and α7 nAChR

Heterologously expressed α4β2 and α3β4 nAChR. HEK 293 cells were grown in Dulbecco's modified Eagle medium supplemented with 10% fetal bovine serum (FBS), 1% L-glutamine, 100 units/mL penicillin G and 100 µg/mL streptomycin in a humidified atmosphere containing 10% CO_2. cDNAs encoding human α3 and β4 or α4 and β2 were transfected into the HEK 293 cells at 30% confluency.

Heterologously expressed α7 nAChR. The SH-SY5Y cells were grown in RPMI medium (Lanza) supplemented with 10% fetal bovine serum (FBS), 1% of penicillin-streptomycin and 1% of L-glutamine. cDNA encoding human α7 was transfected into the SH-SY5Y cells at 30% confluency. The cells were maintained in an environment of 37°C containing 5% CO_2. The cell transfections were carried out in 100 mm Petri dishes using 30 mL of JetPEI™ (Polypus, France) (1 mg/mL, pH 7.2) and 10 µg of cDNAs. After 48 h transfection, the cells were collected, washed with PBS by centrifugation and frozen or used for binding analysis.

(ii) Radioligand binding assays

(±)-[^{3}H]Epibatidine (specific activity of 56-60 Ci/mmol) and [^{125}I]α-bungarotoxin (α-Bgtx) (specific activity of 200-213 Ci/mmol) were purchased from Perkin Elmer (Boston MA). Non-radioactive α-Bgtx, nicotine and epibatidine were purchased from Sigma-Aldrich.

[^{3}H]Epibatidine binding. Details of the binding experiments to the nicotinic subtypes have been previously reported by Tasso *et al.*[32] Saturation experiments were performed by incubating aliquots of membranes from HEK cells expressing α4β2 or α3β4 nAChR with 0.01-2.5 nM concentrations of (±)-[^{3}H]epibatidine overnight at 4°C. Nonspecific binding was determined in parallel by incubation in the presence of 100 nM unlabelled epibatidine. At the end of the incubation, the samples were filtered on GFC filters soaked in 0.5% polyethyleneimine and washed with 15 mL ice-cold phosphate buffered saline (PBS) and the filters were counted for radioactivity in a β counter. The affinity (K_d in nM) of [^{3}H]epibatidine for the α4β2 and α3β4 nAChR subtypes were 0.075, and 0.194 respectively and were derived from the average value of three independent [^{3}H]epibatidine binding saturation experiments.

[^{125}I]α-Bgtx binding. Saturation binding experiments were performed using membranes of α7-transfected SHSY5Y incubated overnight with 0.1-1.0 nM concentrations of [^{125}I] α–Bgtx at rt. Nonspecific binding was determined in parallel by incubation in the presence of 1 µM unlabelled α–Bgtx. After incubation, the samples were filtered as described above and the bound radioactivity was directly counted in a γ counter. Specific radioligand binding was defined as total binding minus the nonspecific binding determined in the presence of 1 µM unlabelled α–Bgtx. Nonspecific binding was ~20-30% of total binding. The K_d of [^{125}I] α–Bgtx for the α7 subtype was 1.2 nM and was derived from the average value of three independent [^{125}I]α–Bgtx binding saturation experiments.

***(iii)* Competition binding assays**

The ability of varenicline variant ligands to compete for the agonist binding sites of α4β2, α3β4 or α7 nAChR was determined by inhibition of [^{3}H]epibatidine and [^{125}I] α–Bgtx binding. Membranes from cells transfected with the appropriate nAChR subtype were incubated with increasing concentrations of test compound for five minutes, followed by overnight incubation at 4 °C, with [^{3}H]epibatidine: 0.1 nM (for α4β2 nAChR) or 0.25 nM (for α3β4 nAChR), or at rt with [^{125}I] α–Bgtx: 2-3 nM (for α7 nAChR); radioligand concentrations approximate to their experimentally determined Kd values (see below). After incubation, the membranes were washed five times with ice-cold PBS. [^{3}H]epibatidine binding was determined by liquid scintillation counting in a β counter, and [^{125}I] α–Bgtx binding by means of direct counting in an γ counter.

***(iv)* Statistical analysis**

Data from competition binding assays were evaluated by one-site competitive binding curve-fitting procedures using GraphPad Prism version 6 (GraphPad Software, Inc, CA, USA). Half maximal inhibition concentrations (IC_{50}) for varenicline variant ligands were obtained by fitting three independent competition binding experiments, each performed in duplicate for each compound on each subtype. Inhibition constants (K_i) were estimated by reference to the K_d of the radioligand, according to the Cheng-Prusoff equation.

In the saturation binding assay, the maximum specific binding (B_{max}) and the equilibrium binding constant (K_d) values were calculated using one site–specific binding with Hill slope – model.

D. nAChR Methods and Functional Studies

(i) Animals

Adult female *Xenopus laevis* were purchased from the European *Xenopus* Resource Center (Portsmouth, UK). *Xenopus laevis* toads were housed and cared for following the UK Home Office code of practice guidelines for the species. The collecting of oocytes from *Xenopus* toads was carried in a regulated room in the Biomedical Services facility in Oxford University, where the toads were housed.

(ii) Human α4β2 nAChR expression in *Xenopus* oocytes

Compounds reported were tested for effects on the function of human α4β2 nACh receptors expressed heterologously in *Xenopus* oocytes, which were isolated from adult female *Xenopus laevis* toads as previously described.[7] Human α4β2 receptors were expressed as either $(\alpha4)_3(\beta2)_2$ (low sensitivity for ACh) or $(\alpha4)_2(\beta2)_3$ (high sensitivity for ACh) receptors. Expressions in oocytes was obtained as follows. Human cDNA for α4 or β2 were subcloned into plasmid pCI from Promega and injected into the nucleus of *Xenopus* oocytes as described previously.[33] To express $(\alpha4)_3(\beta2)_2$ nACh receptors, a mixture of 10 α4 : 1 β2 cDNAs was injected into the nucleus of oocytes, whereas for $(\alpha4)_2(\beta2)_3$ receptors the cDNA ratio injected was 1 α4 : 10 β2.

(iii) Single and double mutations

Mutations were introduced in the α4 or β2 nAChR subunits using the Stratagene QuikChange Site-Directed Mutagenesis Kit (Agilent, UK). The presence of the mutation and the absence of unwanted mutations were confirmed by sequencing the entire cDNA insert (Eurofins, UK). Note that we present the numbering of the residues according to the full length of the following UniProt sequence codes for human α4 and β2 subunits, respectively: P43681 (α4 subunit) and P17787 (β2 subunit). To obtain the position in the mature form, subtract 28 from the number for α4 and 25 for β2 subunit.

(iv) Electrophysiological recordings

Electrophysiological recordings were performed 2-5 days post-injection, as previously described.[28] Current responses were obtained by two-electrode voltage-clamp recording at a holding potential of -60 mV using an Oocyte Clamp OC-725C amplifier (Warner Instruments, USA).

Concentration-response curves for agonists assayed were obtained by normalizing agonist-induced responses to the control ACh responses induced by 1 mM, a maximum effective ACh concentration at both α4β2 nAChR stoichiometries. A minimum interval of 5 minutes was allowed between agonist applications to ensure reproducible recordings. The agonist concentration-response relationship was characterized for data from each cell using non-linear regression in GraphPad (Prism 5, GraphPad, USA) by fitting the Hill equation ($Y([compound]) = Y_{max} (1 / (1 + (EC_{50}/[compound])^\wedge n^{Hill}))$, where Y is the response to a concentration of compound, Y_{max} is the maximal response, EC_{50} is the concentration producing half-maximal activation, and n^{Hill} is the Hill coefficient. Concentration-Response data were collected for an individual cell, and data were normalized to the response to 1 mM ACh. The fit was rejected if the estimated error in any fit parameter was greater than 60% of the fit value, and all parameter estimates for that fit were discarded. For compounds that elicited less than 10% of the maximal ACh response, the EC_{50} was not determined, and the relative efficacy was established by using the equation: maximal response to test compound/maximal ACh response. Data points represent the mean ± standard error of the mean (SEM) of 8-10 experiments carried out in at least three different batches of oocytes donors.

(v) Statistical analysis

For functional assays, the final data sets were assembled from a minimum of 5 independent recordings (i.e. n = 5) conducted on oocytes obtained from at least 5 different *Xenopus* donors. Data obtained from the same batch of oocytes were considered replicates. The data sets represent full concentration-response relationships obtained from individual oocytes (i.e., incomplete experiments were discarded). The data from each experiment were fitted separately and the estimated EC_{50} values were used to obtain the mean EC_{50} or IC_{50} (95% CI) reported in the manuscript or Supplementary Information. $LogEC_{50}$ values for agonist were analyzed using one-way ANOVA, followed by a post hoc Dunnett's test and/or a posthoc Bonferroni multiple comparison test to determine the level of significance between wild type

and mutant receptors. Prior to the ANOVA analysis, the data were tested for normality using the D'Agostino and Pearson normality test in PRISM and were normally distributed. Post hoc tests were run only if F achieved P < 0.05 and there was no significant variance in homogeneity.

(vi) Supporting figures and tables

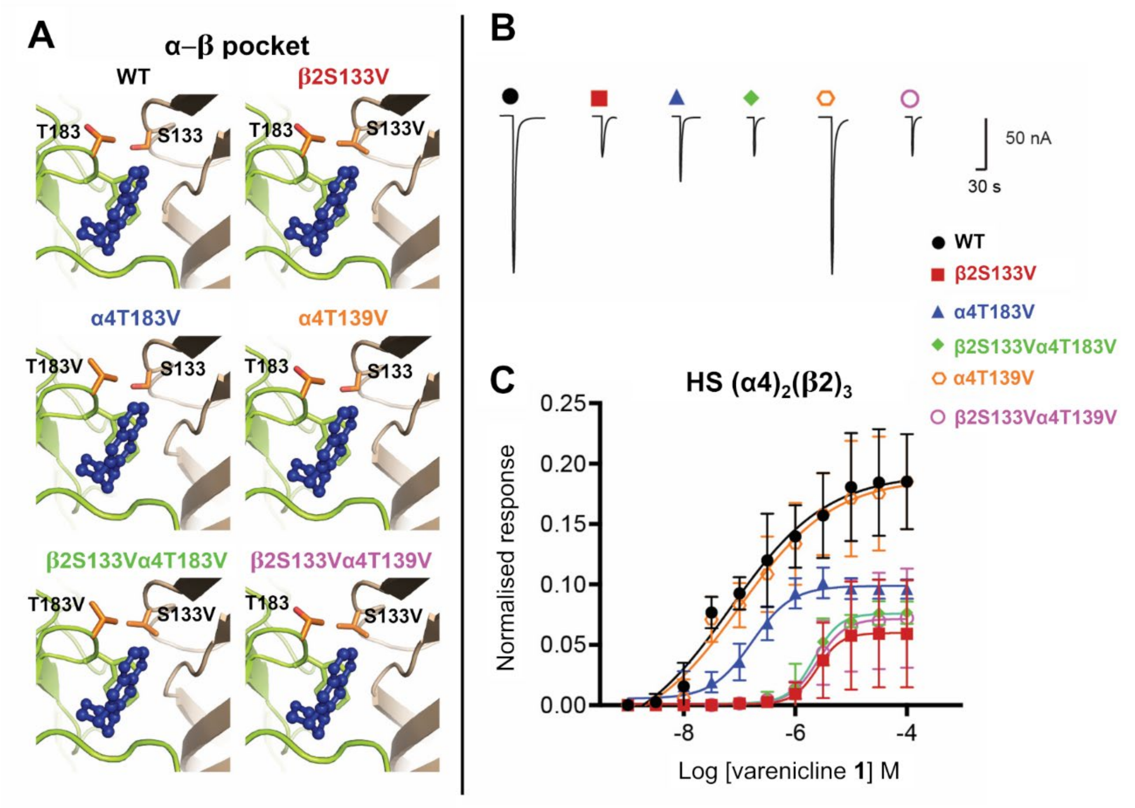

Figure S34- Effects of side-chain hydroxyl mutations on varenicline **1** agonism at the HS isoform of the α4β2 nAChR. (**A**) Location of α4T183 and β2S133 and corresponding mutations to valine in the α-β binding pockets of the wild type and mutants. The α4 and β2 subunits are colored in yellow and light brown, respectively. Varenicline **1** is highlighted in dark blue. The side-chains of α4T183 and β2S133 are represented with orange sticks, whereas TrpB is shown with yellow sticks. (**B**) Representative current traces elicited by maximal concentration of varenicline **1** (100 μM) applied to *Xenopus* oocytes expressing wild-type (WT) or mutant HS nAChR. Full concentration-responses curves are shown in panel **C**. (**C**) Concentration-response curves for varenicline **1** at wild-type (WT) and mutant HS nAChRs. Data points in the concentration-response curves represent the mean ± SEM of 8-10 experiments carried out using 6-8 different *Xenopus* donors. Current responses were measured using two-electrode voltage-clamping from *Xenopus* oocytes heterologously expressing wild type or mutant HS

isoform of the α4β2 nAChR. Peak current amplitudes for varenicline **1** were normalized to maximal ACh response (1 mM) and then fitted with the Hill equation, as described in the Materials and Methods section above. Estimated parameters EC_{50} and maximal relative efficacy (RE) are shown in Table 1 in the main text.

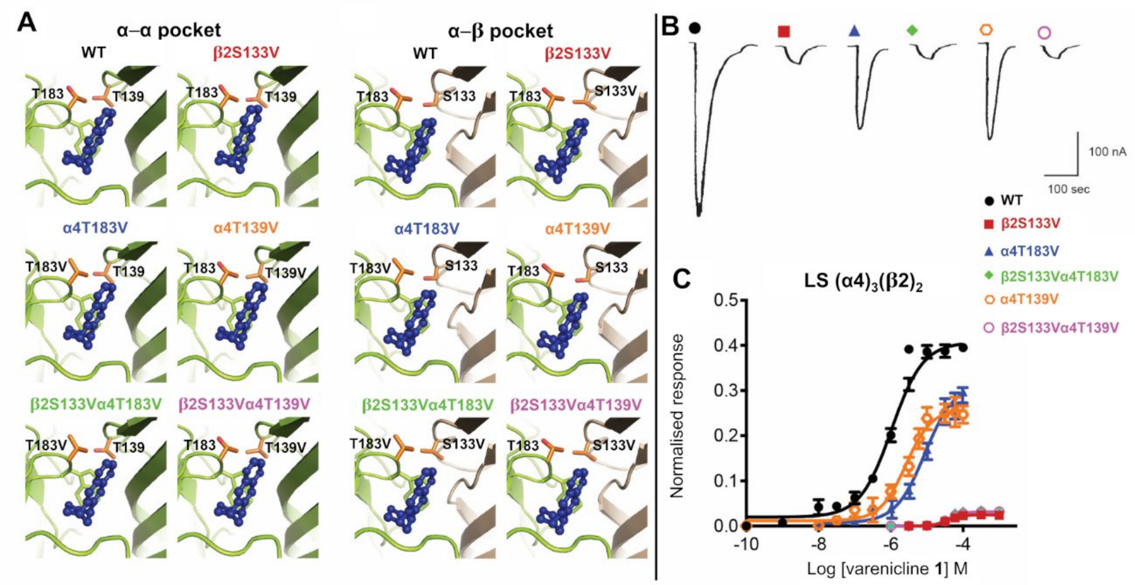

Figure S35- Effects of side-chain hydroxyl mutations on varenicline **1** agonism at the LS isoform of the α4β2 nAChR. (**A**) Location of α4T183, α4T139 and β2S133 and corresponding mutations to valine in the α-α and α-β binding pockets of the wild type and mutants. The α4 and β2 subunits are colored in yellow and light brown, respectively. Varenicline **1** is highlighted in dark blue. The side-chains of α4T183, α4T139 and β2S133 are represented with orange sticks, whereas TrpB is shown with yellow sticks. (**B**) Representative current traces elicited by maximal concentration of varenicline **1** (100 μM) applied to *Xenopus* oocytes expressing wild-type or mutant LS nAChR. Full concentration-responses curves are shown in panel **C**. (**C**) Concentration-response curves for varenicline **1** at wild-type (WT) and mutant LS isoform. Data points in the concentration-response curves represent the mean ± SEM of 8-10 experiments carried out using 6-8 different *Xenopus* donors. Current responses were measured using two-electrode voltage-clamping from *Xenopus* oocytes heterologously expressing WT or mutant LS isoform of the α4β2 nAChR. Peak current amplitudes for varenicline **1** were normalized to maximal ACh response (1 mM) and then fitted with the Hill equation, as described in the Materials and Methods section above. Estimated parameters EC_{50} and maximal relative efficacy (RE) are shown in Table 1 in the main text.

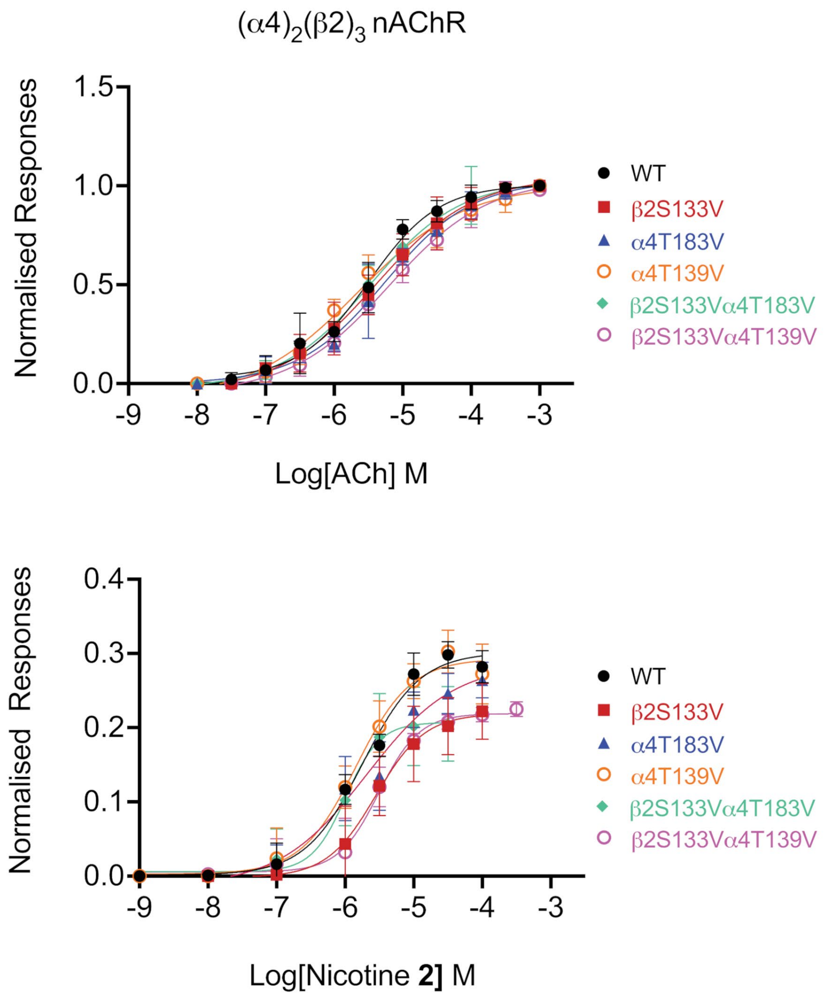

Figure S36- Effects of side-chain hydroxyl mutations on ACh or nicotine **2** agonism at the HS isoform of the α4β2 nAChR. Concentration-response curves for ACh and nicotine **2** at wild-type (WT) and mutant HS nAChRs. Data points in the concentration-response curves represent the mean ± SEM of 8-10 experiments carried out using 6-8 different *Xenopus* donors. Current responses were measured using two-electrode voltage-clamping from *Xenopus* oocytes heterologously expressing WT or mutant HS isoform of the α4β2 nAChR. Peak current amplitudes for ACh or nicotine **2** were normalized to maximal ACh response (1 mM) and then fitted with the Hill equation, as described in the Materials and Methods section above. Estimated parameters EC_{50} and maximal relative efficacy (RE) are shown in Table 1 in the main text.

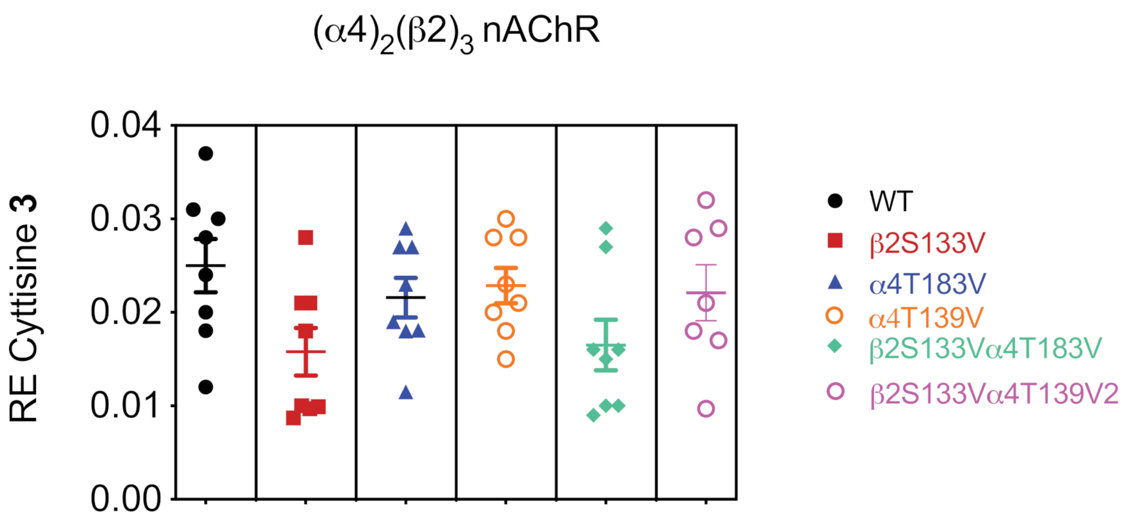

Figure S37- Relative efficacy of cytisine **3** at wild-type (WT) or mutant HS isoform of the α4β2 nAChR. Cytisine **3** displays poor agonist efficacy at $(\alpha4)_2(\beta2)_3$ nAChRs, making it difficult to generate concentration-response curves. To assess the functional effects of side-chain hydroxy mutations on cytisine agonism, we estimated the relative efficacy of cytisine using the equation $I_{max}/I_{max}ACh$. Oocytes were challenged with increasing concentrations of cytisine until the responses reached a plateau, which was considered as the maximal current response to the agonist. Data are shown as a box and whisker plot. Current responses from wild-type (WT) or mutant receptors were measured using two-electrode clamping, as described in the Materials and Methods section above. Statistical comparisons between WT and mutant receptors were performed using One Way ANOVA followed by a post hoc Dunnett's and/or Bonbeferroni multiple comparison tests. The estimated means ± SEM are shown in Table 1 in the main text.

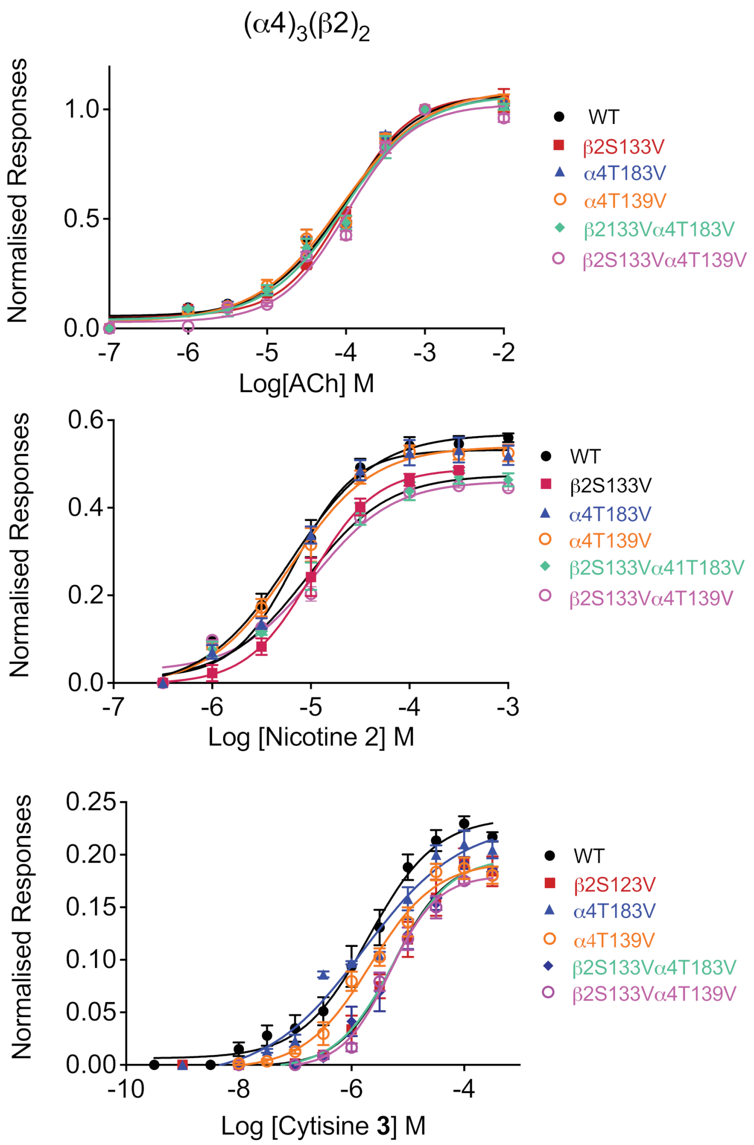

Figure S38- Effects of side-chain hydroxyl mutations on agonist sensitivity of the LS isoform of the α4β2 nAChR. Concentration-response curves for ACh, nicotine **2** and cytisine **3** were obtained at wild type (WT) or mutant $(\alpha4)_3(\beta2)_2$ nAChRs. Data points in the concentration-response curves represent the mean ± SEM of 8-10 experiments carried out using 6-8 different *Xenopus* donors. Current responses were measured using two-electrode voltage-clamping from *Xenopus* oocytes heterologously expressing WT or mutant $(\alpha4)_3(\beta2)_2$ nAChRs. Peak current amplitudes for all agonists tested ACh were normalized to maximal ACh responses (1 mM) prior fitting the data with the Hill equation, as described in the Materials and Methods section above. Estimated parameters EC_{50} and maximal relative efficacy (RE) are shown in Table 1 in the main text.

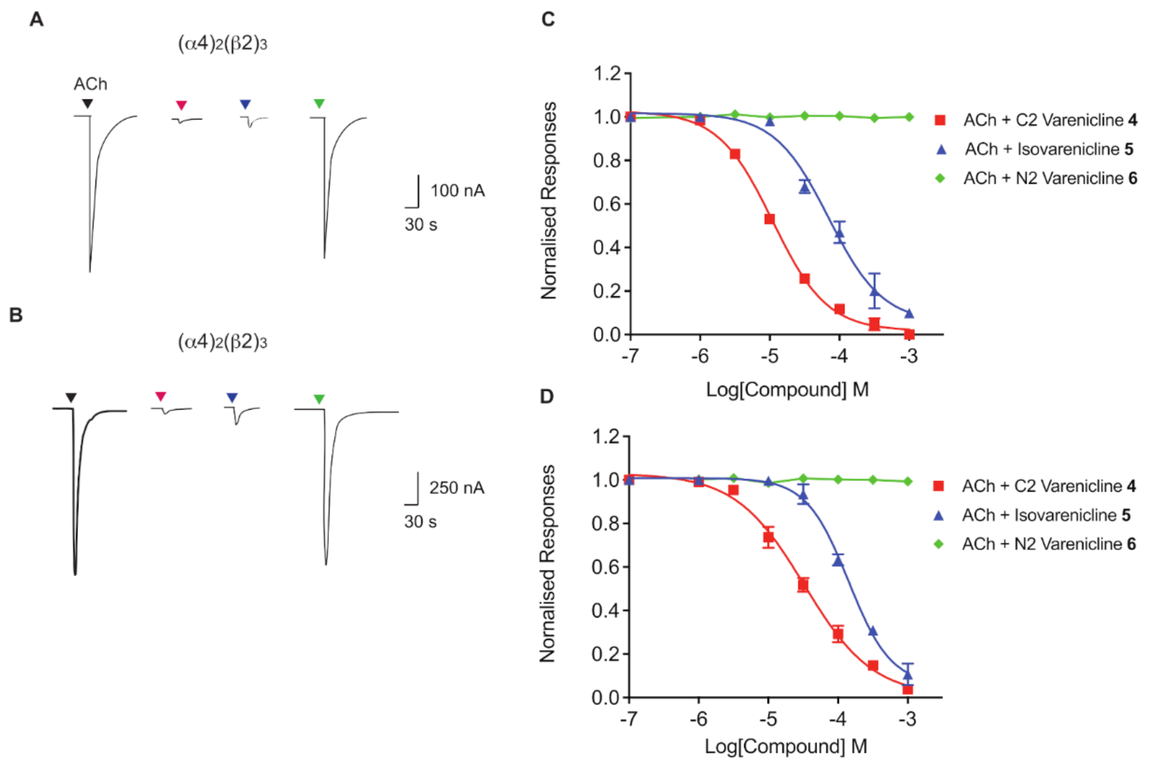

Figure S39- Inhibition of α4β2 by varenicline **1**, C_2 varenicline **4**, isovarenicline **5** and N_2 varenicline **6** on the α4β2 nAChR. C_2 varenicline **4** and isovarenicline **5** behave as partial agonist at α4β2 nAChRs and, as such, should be able to inhibit the responses to ACh. To obtain concentration-response curves data for the inhibitory effects of the varenicline ligands on the alternate forms of the α4β2 nAChR, the compounds (1 nM to 1 mM range) were co-applied with ACh EC_{80}: 30 µM for HS and 300 µM for LS isoforms of the α4β2 receptor. The peak of the current responses obtained in this manner were then normalized to the peak of the responses elicited by ACh EC_{80} alone. The normalized data were then fit by non-linear regression to the Hill equation, as described in the Materials and Methods section above. As shown in the concentration response curves for C_2 varenicline **4** and isovarenicline **5**, these ligands inhibited the responses to ACh in a concentration-dependent manner. In contrast, N_2 varenicline **6**, which had no agonist effect at α4β2 nAChR, did not inhibit the responses to ACh, indicating that this ligand does not bind the agonist sites present on the HS or LS isoform of the α4β2 nAChR.

Table S2- IC_{50} values for the inhibition of $\alpha 4\beta 2$ nAChR by varenicline variants **4**-**6**. The IC_{50} values shown were estimated non-linearly from the concentration-response data shown above, as described in the part (*iv*) of this section above. NE, no effect. [a] The HS isoform corresponds to the $(\alpha 4)_2(\beta 2)_3$ receptor. [b] The LS isoform corresponds to the $(\alpha 4)_3(\beta 2)_2$ receptor.

**Ligand**	**IC_{50} at HS isoform[a]**	**IC_{50} at LS isoform[b]**
C_2 varenicline **4**	11±1.2	33±14
Isovarenicline **5**	70±12	142±21
N_2 varenicline **6**	NE	NE

E. 5-HT_3 Methods and Functional Studies

(i) Cell culture

Human embryonic kidney (HEK) 293 cells (ATCC, Teddington, UK) were maintained on 90 mm tissue culture plates at 37 °C and 7% CO_2 in a humidified atmosphere. They were cultured in DMEM:F12 (Dulbecco's Modified Eagle Medium / Nutrient Mix F12 (1:1)) with GlutaMAX™ (Thermo Fischer Scientific, Paisley, UK), containing 10% HyClone fetal calf serum (GE Healthcare, Hatfield, UK). For radioligand binding studies, cells in 90 mm dishes were transfected with pcDNA3.1 (Thermo Fischer Scientific, Paisley, UK), containing the 5-HT_3AR subunit (Uniprot id P46098) using electroporation. For functional studies, cells were transfected as above and then plated in 96-well plates. Cells were incubated 1-3 days before assay.

(ii) Radioligand binding

This was undertaken as previously described in Price *et al.*.[34] Briefly, transfected HEK293 cell membranes were incubated in 0.5 ml HEPES buffer containing the 5-HT_3 receptor antagonist [^{3}H]GR65630 (0.3 nM; Perkin Elmer, Beaconsfield, UK). Non-specific binding was determined using 1 µM quipazine (Tocris Bioscience, Bristol, UK).

(iii) FlexStation analysis

This technique uses fluorescent voltage-sensitive dyes to detect changes in the membrane potential and has been used to examine a range of ion channels including 5-HT_3 receptors.[35, 36] The methods were as previously described.[36] Briefly, blue fluorescent membrane potential dye (Molecular Devices Ltd., Wokingham, UK) was diluted in Flex buffer (10 mM HEPES, 115 mM NaCl, 1 mM KCl, 1 mM $CaCl_2$, 1 mM $MgCl_2$, 10 mM glucose, pH 7.4) and added to transfected cells grown on a 96-well plates. The cells were incubated at 37 ^{o}C for 30 min and then fluorescence was measured in a FlexStation™ (Molecular Devices Ltd.) every 2 s for 200 s. Buffer or ligand was added to each well after 20 s.

(iv) Data analysis

Concentration response and radioligand binding data were analyzed using Prism software (GraphPad, PRISM, San Diego, CA). Statistical analysis was performed using ANOVA in conjunction with a Dunnett's multiple comparisons post test, or an unpaired t-test as appropriate; $p < 0.05$ was taken as statistically significant.

(v) Supporting figures and tables

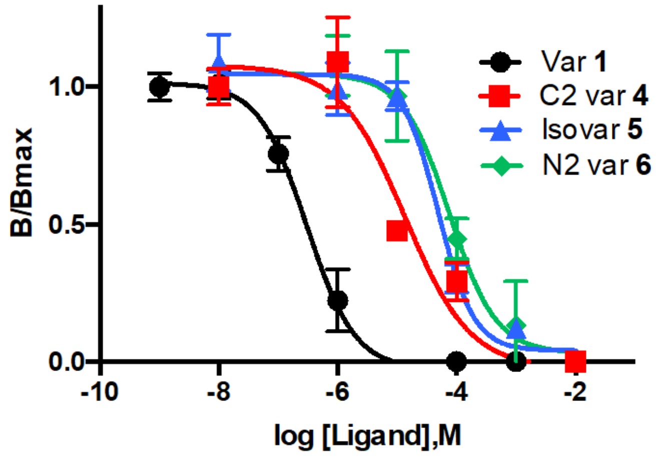

Figure S40. Inhibition of [^{3}H]GR65630 binding reveals varenicline **1** is more potent at the 5-HT$_3$A receptor than analogues **4-6** tested: IC_{50} values were 0.3 μM (pIC_{50}= 6.517 ± 0.07), 13.6 μM (pIC_{50}= 4.866 ± 0.19), 49.6 μM (pIC_{50} = 4.305 ± 0.08) and 75.8 μM (pIC_{50}= 4.12 ± 0.12) for varenicline **1**, C_2 varenicline **4**, isovarenicline **5**, and N_2 varenicline **6**, respectively (data = mean ± SEM, n=3).

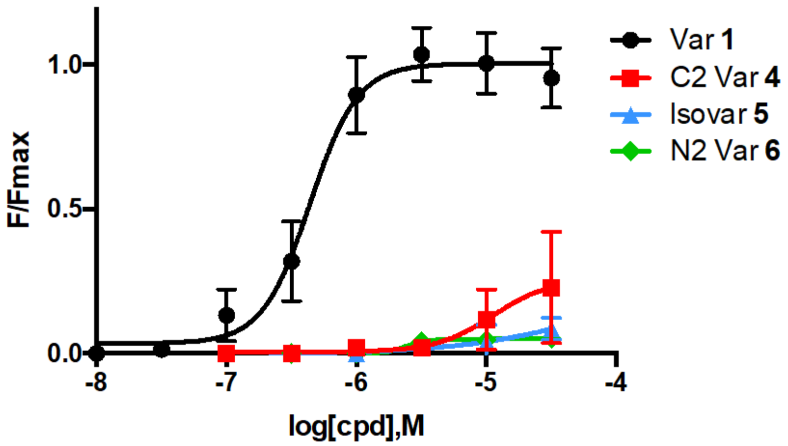

Figure S41. Functional studies using a fluorescent membrane-potential sensitive dye reveal varenicline **1** can activate 5-HT_3A receptors with an EC_{50} of 0.4 μM (pEC_{50} = 6.36 $\pm$ 0.05). No significant responses were observed upon application of C_2 varenicline **4**, isovarenicline **5**, and N_2 varenicline **6** at concentrations up to 30 μM. Fluorescent values (F) are normalized to the maximum response to varenicline **1** (Fmax), data = mean + SEM, n=4.

F. p*K*a Determination

Throughout, pKa refers to the pKa of the protonated form of the base, i.e. pKaH.

(i) Experimental description of the materials and assays

Table S3- Summary of all buffers used in spectrophotometric titrations of compounds reported here. The ionic strength of the aqueous component of each sample was maintained at I = 0.3 M with KCl. Aqueous solutions of HCl and KOH were used to access pH values outside of the range accessible by buffers.

Entry	Aqueous Solution	Total Concentration / M	%fb range	pH range
**1**	Hydrochloric acid (HCl)	0.01 – 0.3	-	0.66 – 2.08
**2**	Formic acid buffer (HCOOH/ HCOOK)	0.1	10 – 40	2.77 – 3.55
**3**	Acetic acid buffer (CH_3COOH/ CH_3COOK)	0.1	10 – 90	3.75 – 5.98
**4**	Phosphate buffer (KH_2PO_4/K_2HPO_4)	0.1	10 – 30	5.89 – 6.96
**5**	Triethanolammonium buffer ($N(CH_2CH_2OH)_3$.HCl)	0.1	10 – 90	6.96 – 9.06
**6**	Carbonate buffer ($KHCO_3$/K_2CO_3)	0.1	10 – 90	9.19 – 11.15
**7**	Triethylammonium buffer (NEt_3.HCl)	0.1	80 – 90	11.40 – 11.92
**8**	Potassium hydroxide (KOH)	0.1 – 0.3	-	12.98 – 13.39

Measurements of the pH of the buffer solutions used were performed using a Radiometer Analytical MeterLab® PHM210 Standard pH Meter with a Radiometer Analytical XC161 Combination pH electrode containing 3 M KCl solution saturated with AgCl.

UV-Vis absorbance spectra were obtained using a Varian Cary 100 Bio UV-Vis spectrophotometer with a temperature regulated cuvette holder and attached heating unit. All absorbance data were obtained at 25 °C. Spectra were obtained for each substrate for wavelengths in the range 800 nm to 200 nm at 600 nm min^{-1} (1 nm interval, 0.1 s average time, UV/Vis source change over at 350 nm) with baseline correction to account for absorbance due to the buffer present.

For single wavelength absorbance experiments, the mean absorbance at a chosen wavelength was measured over the course of 1 min, with correction of the absorbance due to the buffer

present. Margins of error reported for values of K_a and pK_a constants were taken from the standard error in the respective spectrophotometric titration.

(ii) Theoretical background

Due to the comparatively small amounts of material available (e.g. varenicline **1** and the variants described in the main text), a UV-Vis spectrophotometric titration method was employed for pK_a determination. To access the pK_a of a given compound, the change in absorbance at a chosen wavelength, λ_{obs}, is measured as the pH is changed. The observed absorbance at a given pH, A_{obs}, at λ_{obs} is determined by the concentrations of the protonated and deprotonated forms of the analyte.

Absorbance-pH data were fitted to Equation (1):

$$K_a = \frac{10^{-pH}(A_{max} - A_{obs})}{(A_{obs} - A_{min})} \quad (1)$$

Equation (2) was used when A_{obs} decreased with increasing pH at λ_{obs}.

$$A_{obs} = \frac{A_{max}.10^{-pH} + A_{min}.K_a}{10^{-pH} + K_a} \quad (2)$$

Where A_{obs} increased with increasing pH at λ_{obs}, the data were instead fitted to Equation (3).

$$A_{obs} = \frac{A_{min}.10^{-pH} + A_{max}.K_a}{10^{-pH} + K_a} \quad (3)$$

The following conditions were maintained for each titration: 25 °C, buffer ionic strength, I.S. = 0.3 M and 10 v/v% acetonitrile co-solvent in aqueous solution.

(iii) Spectrophotometric method validation: 4-dimethylaminopyridine (DMAP) and nicotine

As a control, a spectrophotometric titration of DMAP was performed under the experimental conditions described above (Figure S42) .

Figure S42- Spectrophotometric titration of DMAP

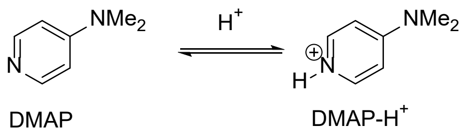

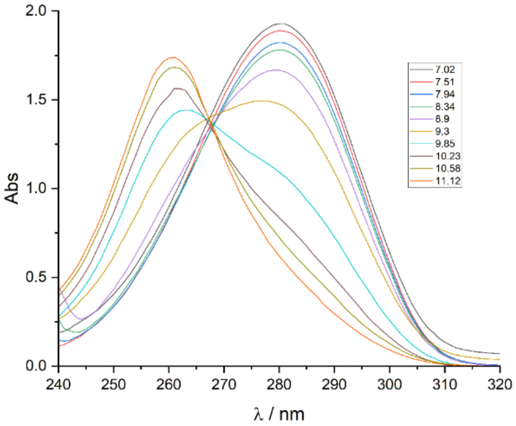

Analytical wavelengths (λ_{obs}) of 261 nm and 280 nm were chosen on either side of the isosbestic point at 268 nm. At 261 nm, DMAP is more absorbing than DMAP-H$^+$, therefore the data were fit to Equation (3). At 280 nm, the opposite is the case, so the data were fit to Equation (2). The titrations at both wavelengths are shown in Figure S43.

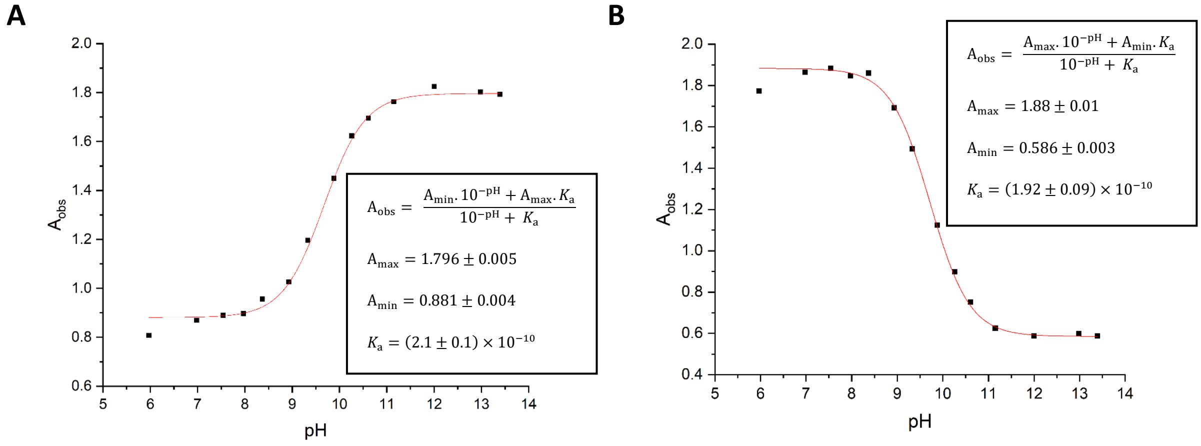

Figure S43- Spectrophotometric titration of DMAP at 261 nm (**A**) and 280 nm (**B**).

For the spectrophotometric titration of DMAP at 261 nm, the acid dissociation constant K_a of DMAP-H$^+$ was determined as (2.1 ± 0.1) x 10^{-10} M (pK_a = 9.67 ± 0.02) via equation (3). By analysis of data at 280 nm, K_a = (1.92 ± 0.09) x 10^{-10} M (pK_a = 9.72 ± 0.02) was obtained via Equation (2). The close similarity in pK_a values determined at both wavelengths and the independent literature value of 9.6, asserts the validity of this method.[37] The small 0.1 unit increase in pK_a of the conjugate acid of DMAP versus the literature value can be attributed to the presence of 10 vol% acetonitrile in aqueous solution, necessary for the solubility of the substrates in the present study. Increases in pK_a values of neutral and cationic acids are observed in the pure weak donor solvent MeCN versus more polar protic media.

The pK_a values (pK_{a1} pK_{a2}) of the conjugate acids of nicotine **2** were also determined under these experimental conditions to further validate the UV-Vis spectrophotometric method versus available literature values. The analytical wavelengths for the spectrophotometric titrations were 259 nm and 269 nm, respectively.

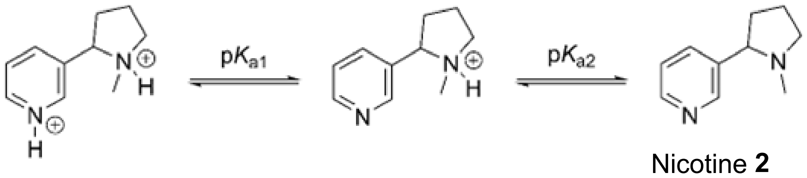

Nicotine **2**

Using the same protocol as described for DMAP, the observed absorbance change due to the deprotonation of the pyridinium moiety is larger than that for the *N*-methylpyrrolidinium component hence a greater concentration of nicotine **2** was necessary to determine K_{a2} (3.75 x 10^{-4} M) than for K_{a1} (1.67 x 10^{-4} M). Values of pK_{a1} = 3.27 $\pm$ 0.02 and pK_{a2} = 8.19 $\pm$ 0.03 were obtained by UV-Vis spectrophotometric titration under our experimental conditions. These pK_as values are closely similar to reported literature values in water which range from 3.04-3.41 for pK_{a1} and 7.94-8.02 for pK_{a2}.[38-41]

***(iv)* Spectrophotometric titration of varenicline 1, nicotine 2, cytisine 3 and varenicline variants 4-6**

The pK_a values of varenicline **1**, nicotine **2**, cytisine **3**, and varenicline derivatives **4-6** were determined. Spectrophotometric titration curves are shown in Figures S44-S49 and the resultant pK_a values are summarized in Table S4.

Figure S44- Spectrophotometric titrations of nicotine **2**

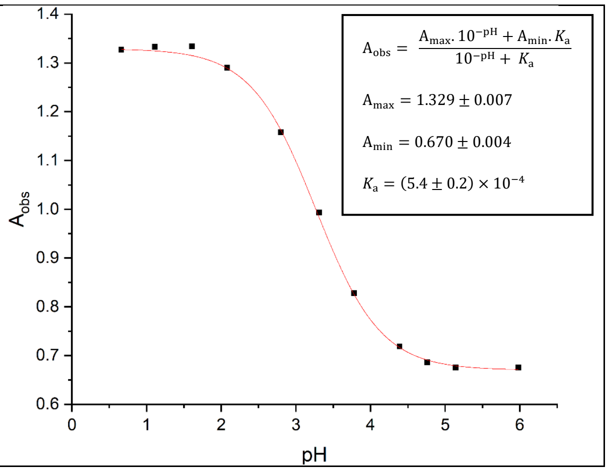

<table>
<tr><td></td><td>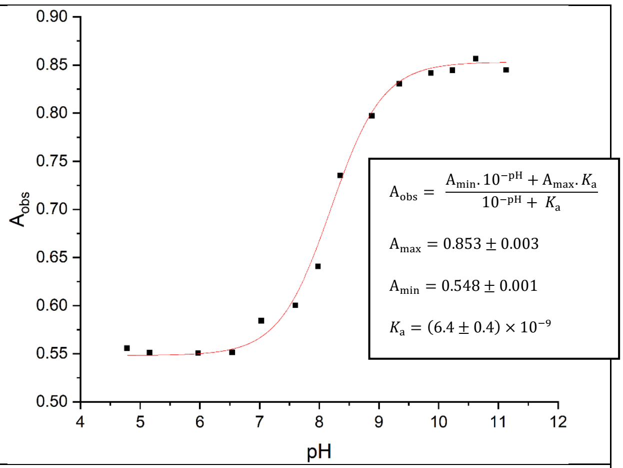
</td></tr>
<tr><td>Figure S45- Spectrophotometric titration of cytisine 3</td><td>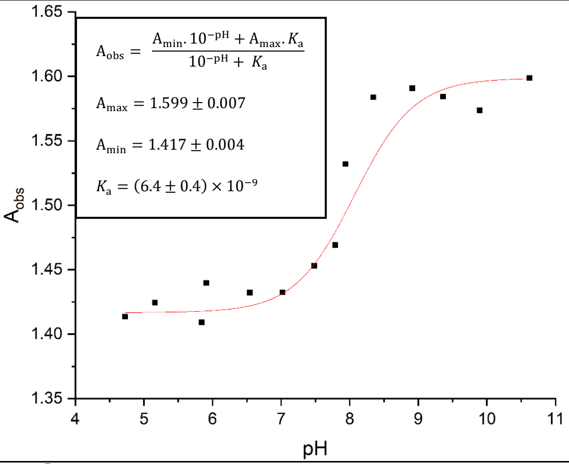
</td></tr>
<tr><td>Figure S46- Spectrophotometric titration of varenicline tartrate 1</td><td>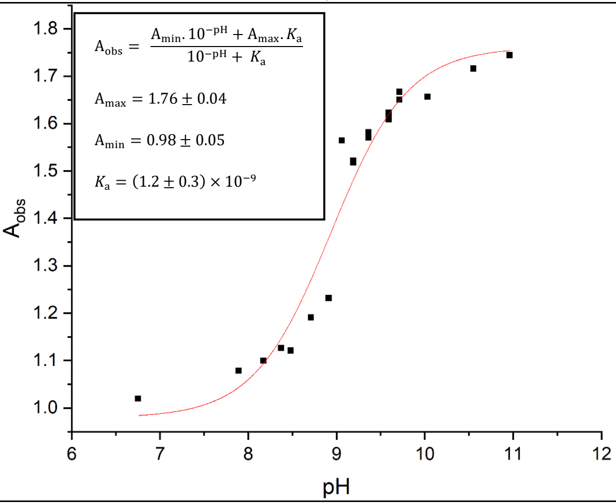
</td></tr>
</table>

Figure S47- Spectrophotometric titration of C_2 varenicline hydrochloride **4**

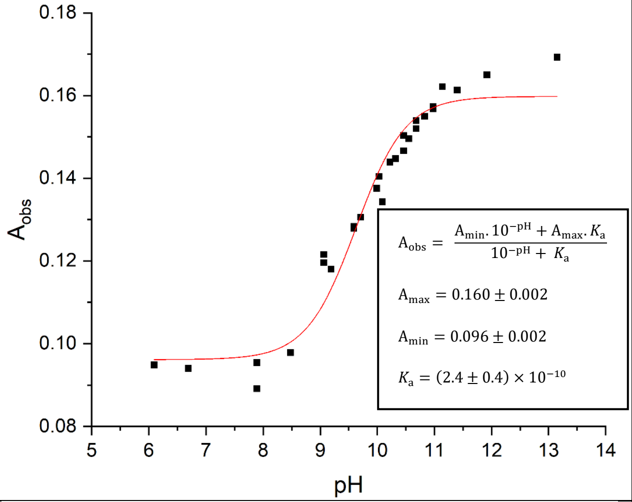

Figure S48- Spectrophotometric titration of isovarenicline hydrochloride **5**

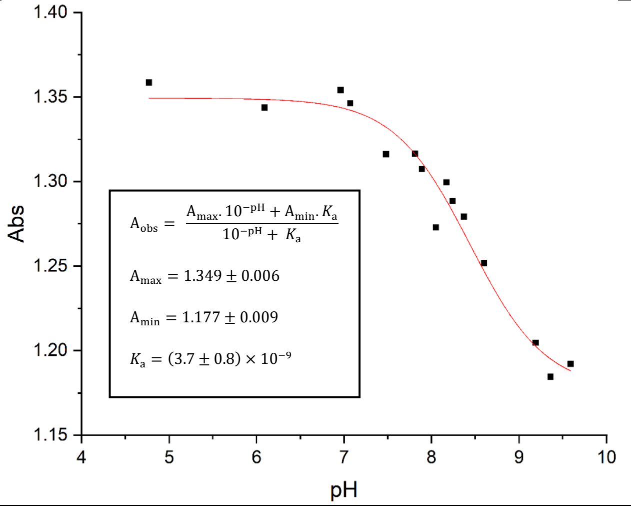

Figure S49- Spectrophotometric titration of N_2 varenicline trifluoroacetate **6**

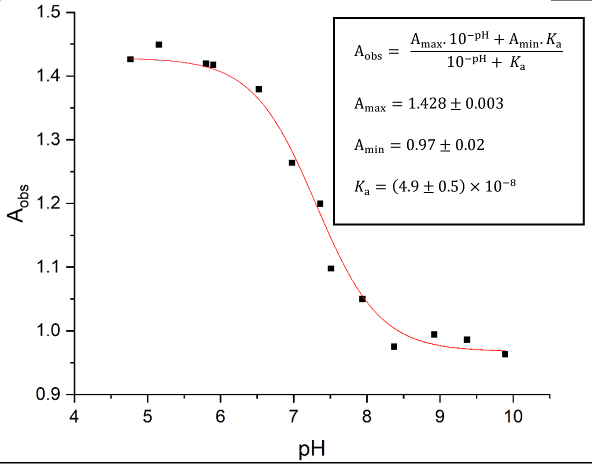

Table S4- Summary of experimentally-determined pK_a values. [a]Determined at 25 °C, buffer ionic strength, I.S. = 0.3 M and 10 v/v% acetonitrile co-solvent in aqueous solution.

**Compound**	**pK_a value[a]**	**Literature value**
4-Dimethylaminopyridine (DMAP)	9.67 ± 0.02	9.6[37]
Nicotine **2**	3.27 ± 0.02<br>8.19 ± 0.03	3.04 – 3.41[38-41]<br>7.94 – 8.02[38-41]
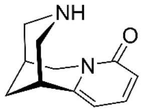<br>Cytisine **3**	8.07 ± 0.07	7.8[42]
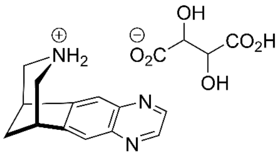<br>Varenicline tartrate **1**	8.90 ± 0.1	9.3[42]<br>9.2 ± 0.1[43]
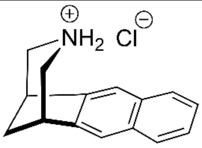<br>C_2 Varenicline hydrochloride **4**	9.63 ± 0.08	-
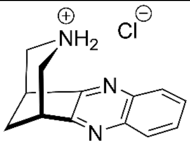<br>Isovarenicline hydrochloride **5**	8.44 ± 0.09	-
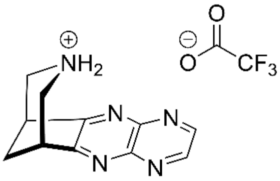<br>N_2 Varenicline trifluoroacetate **6**	7.31 ± 0.05	-

The pK_a value for varenicline tartrate **1** (8.9 ± 0.1) is ~0.3 units lower than the literature values (9.3[6] and 9.22 ± 0.13[7]). The literature value of 9.3 is provided without a quoted error or discussion of how the value was reached. The second literature value of 9.22 ± 0.13 was determined by a potentiometric titration in 100% water and at a different ionic strength of 0.15 M, which likely accounts for the observed difference of 0.29 units. The pK_a determined for cytisine **3** is 0.3 units greater than the literature value (7.8); again, this literature value is provided without a quoted error or discussion of how the value was reached.[42] The literature pK_a of the conjugate acid of piperidine is reported in the range 11.06-11.18.[38, 44-47]

G. Supplemental Methods References

(1) Paddon-Row, MN; Patney, HK. *An Efficient Synthetic Strategy for Naphthalene Annellation of Norbornenylogous Systems*. Synthesis. **1986**, *328*, 328-330.

(2) Grabowski, EY; AbuSalim, DI; Lash, TD. *Naphtho[2,3- b]carbaporphyrins*. J Org Chem. **2018**, *83*, 11825-11838.

(3) Brooks, P; Caron, S; Coe, J*; et al. Synthesis of 2,3,4,5-Tetrahydro-1,5-methano-1H-3-benzazepine via Oxidative Cleavage and Reductive Amination Strategies*. Synthesis. **2004**, *2004*, 1755-1758.

(4) Maier, L; Khirsariya, P; Hylse, O*; et al. Diastereoselective Flexible Synthesis of Carbocyclic C-Nucleosides*. J Org Chem. **2017**, *82*, 3382-3402.

(5) Muehlmann, FL; Day, AR. *Metabolite Analogs. V. Preparation of Some Substituted Pyrazines and Imidazo[b]pyrazines*. J Am Chem Soc. **1956**, *78*, 242-244.

(6) Walsh, RM, Jr.; Roh, SH; Gharpure, A*; et al. Structural principles of distinct assemblies of the human alpha4beta2 nicotinic receptor*. *Nature*. **2018**, *557*, 261-265.

(7) Minguez-Viñas, T; Nielsen, BE; Shoemark, DK*; et al. A conserved arginine with non-conserved function is a key determinant of agonist selectivity in α7 nicotinic acetylcholine receptors*. Br J Pharmacol. **2021**, *178*, 1651-1668.

(8) Campello, HR; Del Villar, SG; Honraedt, A*; et al. Unlocking nicotinic selectivity via direct C–H functionalisation of (−)-cytisine*. Chem. **2018**, *4*, 1710-1725.

(9) Mukherjee, S; Erramilli, SK; Ammirati, M*; et al. Synthetic antibodies against BRIL as universal fiducial marks for single-particle cryoEM structure determination of membrane proteins*. Nat Commun. **2020**, *11*, 1598.

(10) Morales-Perez, CL; Noviello, CM; Hibbs, RE. *X-ray structure of the human alpha 4 beta 2 nicotinic receptor*. Nature. **2016**, *538*, 411-415.

(11) DeLano, WL. *PyMOL molecular viewer: Updates and refinements*. Abstr Pap Am Chem S. **2009**, *238*,

(12) Abraham, MJ; Murtola, T; Schulz, R*; et al. GROMACS: High performance molecular simulations through multi-level parallelism from laptops to supercomputers*. SoftwareX. **2015**, *1-2*, 19-25.

(13) Lindorff-Larsen, K; Piana, S; Palmo, K*; et al. Improved side-chain torsion potentials for the Amber ff99SB protein force field*. Proteins. **2010**, *78*, 1950-1958.

(14) Sousa da Silva, AW; Vranken, WF. *ACPYPE - AnteChamber PYthon Parser interfacE*. BMC Res Notes. **2012**, *5*, 367.

(15) Jorgensen, WL; Chandrasekhar, J; Madura, JD*; et al. Comparison of simple potential functions for simulating liquid water*. J Chem Phys. **1983**, *79*, 926-935.

(16) Essmann, U; Perera, L; Berkowitz, ML. *A smooth particle mesh Ewald method*. J Chem Phys. **1995**, *103*, 8577-8593.

(17) Hess, B; Bekker, H; Berendsen, HJC*; et al. LINCS: a linear constraint solver for molecular simulations*. J Comput Chem. **1997**, *18*, 1463-1472.

(18) Miyamoto, S; Kollman, PA. *SETTLE: an analytical version of the SHAKE and RATTLE algorithms for rigid water models*. J Comput Chem. **1992**, *13*, 952-962.

(19) Bussi, G; Donadio, D; Parrinello, M. *Canonical sampling through velocity rescaling*. J Chem Phys. **2007**, *126*, 014101.

(20) Parrinello, M; Rahman, A. *Polymorphic transitions in single crystals: A new molecular dynamics method.* J Appl Phys. **1981**, ***52***, 7182–7190.

(21) Nosé, S; Klein, ML. *Constant pressure molecular dynamics for molecular systems*. Mol Phys. **1983**, *50*, 1055–1076.

(22) Kabsch, W; Sander, C. *Dictionary of protein secondary structure: pattern recognition of hydrogen-bonded and geometrical features*. Biopolymers. **1983**, *22*, 2577-2637.

(23) Oliveira, ASF; Shoemark, DK; Campello, HR*; et al. Identification of the initial steps in signal transduction in the α4β2 nicotinic receptor: insights from equilibrium and nonequilibrium simulations*. Structure. **2019**, *27*, 1171-1183.

(24) Oliveira, ASF; Edsall, C; Woods, C*; et al. A general mechanism for signal propagation in the nicotinic acetylcholine receptor family*. J Am Chem Soc. **2019**, *141*, 19953–19958.

(25) Xiu, X; Puskar, NL; Shanata, JA*; et al. Nicotine binding to brain receptors requires a strong cation-pi interaction*. Nature. **2009**, *458*, 534-537.

(26) Cashin, A; Petersson, E; Lester, H*; et al. Using physical chemistry to differentiate nicotinic from cholinergic agonists at the nicotinic acetylcholine receptor*. J Am Chem Soc. **2005**, *127*, 350-356.

(27) Marotta, CB; Rreza, I; Lester, HA*; et al. Selective ligand behaviors provide new insights into agonist activation of nicotinic acetylcholine receptors*. ACS Chem Biol. **2014**, *9*, 1153-1159.

(28) Tavares, XDS; Blum, AP; Nakamura, DT*; et al. Variations in binding among several agonists at two stoichiometries of the neuronal, alpha4beta2 nicotinic receptor*. J Am Chem Soc. **2012**, *134*, 11474-11480.

(29) Blum, AP; Lester, HA; Dougherty, DA. *Nicotinic pharmacophore: the pyridine N of nicotine and carbonyl of acetylcholine hydrogen bond across a subunit interface to a backbone NH*. Proc Natl Acad Sci U S A. **2010**, *107*, 13206-13211.

(30) Madeira, F; Madhusoodanan, N; Lee, J*; et al. The EMBL-EBI Job Dispatcher sequence analysis tools framework in 2024*. Nucleic Acids Res. **2024**, *52*, W521-W525.

(31) Valdar, WS. *Scoring residue conservation*. Proteins. **2002**, *48*, 227-241.

(32) Tasso, B; Canu Boido, C; Terranova, E*; et al. Synthesis, binding, and modeling studies of new cytisine derivatives, as ligands for neuronal nicotinic acetylcholine receptor subtypes*. J Med Chem. **2009**, *52*, 4345-4357.

(33) Moroni, M; Zwart, R; Sher, E*; et al. alpha4beta2 nicotinic receptors with high and low acetylcholine sensitivity: pharmacology, stoichiometry, and sensitivity to long-term exposure to nicotine*. Mol Pharmacol. **2006**, *70*, 755-768.

(34) Price, KL; Lummis, SC. *The role of tyrosine residues in the extracellular domain of the 5-hydroxytryptamine3 receptor*. J Biol Chem. **2004**, *279*, 23294-23301.

(35) Fitch, RW; Xiao, Y; Kellar, KJ*; et al. Membrane potential fluorescence: a rapid and highly sensitive assay for nicotinic receptor channel function*. Proc Natl Acad Sci U S A. **2003**, *100*, 4909-4914.

(36) Price, KL; Lummis, SC. *FlexStation examination of 5-HT3 receptor function using Ca2+ - and membrane potential-sensitive dyes: advantages and potential problems*. J Neurosci Meth. **2005**, *149*, 172-177.

(37) Kaljurand, I; Kutt, A; Soovali, L*; et al. Extension of the self-consistent spectrophotometric basicity scale in acetonitrile to a full span of 28 pKa units: unification of different basicity scales*. J Org Chem. **2005**, *70*, 1019-1028.

(38) Perrin, DD. *Dissociation Constants of Organic Bases in Aqueous Solution*; Butterworths, 1965.

(39) Vickery, H; Pucher, G. *The determination of 'free nicotine' in tobacco : The apparent dissociation constants of nicotine*. J Biol Chem. **1929**, *84*, 233–241.

(40) Fowler, RT. *A redetermination of the ionization constants of nicotine*. J App Chem. **1954**, *4*, 449–452.

(41) Barlow, RB; Hamilton, JT. *Effects of some isomers and analogues of nicotine on junctional transmission*. Br J Pharmacol Chemother. **1962**, *18*, 510-542.

(42) Rollema, H; Shrikhande, A; Ward, KM*; et al. Pre-clinical properties of the alpha4beta2 nicotinic acetylcholine receptor partial agonists varenicline, cytisine and dianicline translate to clinical efficacy for nicotine dependence*. Br J Pharmacol. **2010**, *160*, 334-345.

(43) Unal, G; Yeloglu, I; Anilanmert, B*; et al. pKa Constant of Varenicline*. J Chem Eng Data. **2012**, *57*, 14–17.

(44) Searles, S; Tamres, M; Block, F*; et al. Hydrogen Bonding and Basicity of Cyclic Imines*. J Am Chem Soc. **1956**, *78*, 4917–4920.

(45) Bates, R; Bower, V. *Dissociation Constant of Piperidinium Ion from 0-Degrees to 50-Degrees-C and Related Thermodynamic Quantities*. J Res Nat Bur Stand. **1956**, *57*, 153–157.

(46) Horwitz, JP; Rila, CC. *A Comparison of the Reactions of Some Amines with Nitrosoguanidine, Cyanamide and S-Methylisothiourea Hydrochlorides*. J Am Chem Soc. **1958**, *80*, 431–437.

(47) Geissman, TA; Wilson, BD; Medz, RB. *The Base Strengths of cis- and trans-1,2-Aminoalcohols*. J Am Chem Soc. **1954**, *76*, 4182–4183.
